# Evolution of NLR genes in genus *Arachis* reveals asymmetric expansion of NLRome in wild and domesticated tetraploid species

**DOI:** 10.1038/s41598-023-36302-1

**Published:** 2023-06-08

**Authors:** Muhammad Rizwan, Syed Zeeshan Haider, Abu Bakar, Shamiza Rani, Muhammad Danial, Vikas Sharma, Muhammad Mubin, Ali Serfraz, Muhammad Shahnawaz-ur-Rehman, Sidra Shakoor, Saad Alkahtani, Fozia Saleem, Hafiz Mamoon-ur-Rehman, Saad Serfraz

**Affiliations:** 1 Evolutionary Biology Lab, CABB, University of Agriculture, Faisalabad, 38000 Pakistan; 2 Biofuels Institute, School of the Environment and Safety Engineering, Jiangsu University, Zhenjiang, China; 3 Forschungszentrum Jülich GmbH, Institute for Bio- and Geosciences 1, IBG1, 52425 Jülich, Germany; 4 Virology Lab, CABB, University of Agriculture, Faisalabad, 38000 Pakistan; 5 Department of Plant Pathology, University of Arid Agriculture, Rawalpindi , Pakistan; 6 Department of Zoology, College of Science, King Saud University, P. O. Box 2455, Riyadh, 11451 Saudi Arabia

**Keywords:** Phylogenetics, Molecular evolution, Evolution, Speciation, Comparative genomics, Genome evolution, Phylogenomics, Transcriptomics

## Abstract

*Arachis hypogaea* is an allotetraploid crop widely grown in the world. Wild relatives of genus *Arachis* are the rich source of genetic diversity and high levels of resistance to combat pathogens and climate change. The accurate identification and characterization of plant resistance gene, nucleotide binding site leucine rich repeat receptor (NLRs) substantially contribute to the repertoire of resistances and improve production. In the current study, we have studied the evolution of NLR genes in genus *Arachis* and performed their comparative genomics among four diploids (*A. duranensis*, *A. ipaensis*, *A. cardenasii*, *A. stenosperma*) and two tetraploid (wild: *A. monticola* and domesticated: *A. hypogaea*) species. In total 521, 354, 284, 794, 654, 290 NLR genes were identified from *A. cardenasii, A. stenosperma* and *A. duranensis*, *A. hypogaea, A. monticola* and *A. ipaensis* respectively. Phylogenetic analysis and classification of NLRs revealed that they belong to 7 subgroups and specific subgroups have expanded in each genome leading towards divergent evolution. Gene gain and loss, duplication assay reveals that wild and domesticated tetraploids species have shown asymmetric expansion of NLRome in both sub-genome (AA and BB). A-subgenome of *A. monticola* exhibited significant contraction of NLRome while B-subgenome shows expansion and vice versa in case of *A. hypogaea* probably due to distinct natural and artificial selection pressure. In addition, diploid species *A. cardenasii* revealed the largest repertoire of NLR genes due to higher frequency of gene duplication and selection pressure. *A. cardenasii* and *A. monticola* can be regarded as putative resistance resources for peanut breeding program for introgression of novel resistance genes. Findings of this study also emphasize the application neo-diploids and polyploids due to higher quantitative expression of NLR genes. To the best of our knowledge, this is the first study that studied the effect of domestication and polyploidy on the evolution of NLR genes in genus *Arachis* to identify genomic resources for improving resistance of polyploid crop with global importance on economy and food security.

## Introduction

Groundnut or cultivated peanut (*Arachis hypogaea*) is considered as the most important oil and food legume, grown on 25 million ha with annual production of ~ 46 million tons. *A. hypogaea* was domesticated in South America ~ 6000 years ago and then widely distributed in post-columbian times^1^. The genus Arachis consists of 81 species that are mostly diploids. They are classified into nine sections, each with distinct reproductive anatomy, and shows a unique reproductive trait for subterranean fruits^2–4^. Section *Arachis* is genetically diverse and consist of 30 diploid species and two tetraploids, one wild (*A. monticola*) and the other domesticated (*A. hypogaea*)^5,6^. These tetraploids are the result of interspecific hybridization between two diploid species *A. duranensis* and (AA, 2n = 20) and *A. ipaensis* (BB = 2n = 20) that gave rise to wild tetraploids (*A. monticola*) and after subsequent domestication evolved into a cultivated species *A. hypogaea* (AABB)^1,6,7^*.* Domesticated and repeated cycle of artificial selection have narrowed the genetic base of *A. hypogaea* which rendered it vulnerable to number of biotic and abiotic stress factors. Peanut crop production is threatened by several disease from bacterial, fungal, virus and nematode diseases including Aspergillus crown rot^8^, peanut root-knot nematode^9^ and Cylindrocladium Black Rot (CBR)^10^ etc. Tapping the wild relatives for broadening the genetic base is an excellent strategy for acquisition of durable resistance against pathogens. Therefore, gaining understanding of underlying molecular mechanism of disease resistance genes, their accurate detection and characterization is vital for achieving higher production rates.

Nucleotide binding site leucine rich repeat receptor (NLRs) recognize the pathogen’s effector via direct or indirect interaction, that activates a number of defensive mechanisms, one of which is hypersensitive response also known as localized programmed cell death^11^. NLR mainly consist of Nucleotide binding domain (NB-ARC) and C-terminal leucine-rich repeats (NLRs). The NB-ARC domain is the most conserved region to determine the evolutionary relationship between plant NLRs^12^. There are four major classes of plant NLRs with distinct N-terminal domain fusion: (1) The TIR-NLR subclade containing an N-terminal Toll/interleukin-1 receptor (TIR) domain, (2) CC-NLR subclade containing an N-terminal type Rx-type coiled coil (CC) domain, (3) CC_R_-NLR subclade containing the RTP8-type CC domain and recently proposed (4) G10 subclade that contains the distinct type of CC and forms a monophyletic group. Previous genome wide analysis on *Arachis* species were reported in 2003 and 2011 using PCR and BAC library based approach^13,14^. Genome-wide identification and annotation of NLR genes from plants are challenging owing to their complex sequence diversity and evolutionary history. However, recently released tool NLRtracker identify and characterize NLR genes in high-throughput manner using canonical features of functionally characterized plant resistance genes^15^.

Genus *Arachis* provides an excellent opportunity to understand the evolution of NLR genes in diploid and tetraploid species. To this date, comprehensive understanding of evolution of resistance genes in *Arachis* is not reported. Here we have employed NLRtracker to characterize NLR genes in four diploid and two tetraploid species of *Arachis* to answer complex questions that are described as follows. What is the effect of allopolyploidy on the NLR evolution in genus *Arachis*? Whether it causes contraction or expansion of NLRome? Whether this expansion is symmetrical across wild and domesticated tetraploids. What are the suitable wild species that can utilized for introgression into cultivated polyploids to ameliorate in crop production? What are the major evolutionary mechanism employed by *Arachis* wild relatives to broadens their genetic base for combating biotic and abiotic stress factors?

## Methods

### Mining of NLR genes in ***Arachis species***

The genome assembly of six *Arachis* species were downloaded from peanutbase (Table S1). Genome, complete coding sequence (CDS) and reference proteome files for three species *A. hypogaea* (v.2), *A. duranensis* (v.1) and *A. ipenensis* (v.1) were acquired from peanutbase (www.peanutbase.org). Genome assembly of wild relatives *A. monticola*, *A. stenosperma, A. cardenasii* were downloaded from NCBI genome portal (Table S1). These three genome were annotated using augustus (v-3.4.0)^16^ with default settings except for the option of complete gene models (–genemodel = complete). The resulting gff file was parsed into amino acid and coding sequences using two perl scripts (getAnnotFasta.pl and gffread)^16^. Tetraploids species *A. hypogaea* and *A. monticola* were split in to individual A and B genomes in order to simplify the comparison between ancestral species. Reference proteomes from all *Arachis* species were subjected to the NLRtracker pipeline, which extracts and annotates NLRs from proteins and transcript files. NLRtracker pipeline uses Interproscan^17^ and predefined NLR motifs^18^ to extract NLRs and provide domain architecture analyses based on the canonical features found in reference plant NLR genes. NLRtracker annotation of CC_R_-NLR remained undetermined for this reason, manual curation was performed for each NLR gene using clustering and phylogenetic analysis.

### Clusterization and phylogenetic analysis

A library of NB-ARC domain was constructed from reference NLR genes of the PRG database^12^ and clustered using UCLUST^19^ with an identity threshold of 50%. The resulting reference genes from each cluster were classified into subgroups already defined by Eunyoung Seo et al.^20^ and considered as seed probes for phylogenetic and clustering analysis. For comprehensive phylogenetic analysis, extracted NB-ARC domains (output of NLRtracker) from *Arachis* species were aligned with seed probes of NB-ARC using MUSCLE (version 1.26, Hull, 2009). Subsequent maximum likelihood analysis was performed using IQtree v 2.0^21^, choosing the best-fit model of evolution (-m VT + F + R9) and 1000 bootstrap replicates. We further calculated number of gene cluster and their architecture in each species of genus *Arachis* by estimating the number of genes in window of 500 kb. Latter we utilized R based conventional script for overlapping visualization.

### Chromosomal localization and construction of a syntenic *R*-gene maps

Coordinates for identified NLR genes were extracted and subjected to density distribution analyses. Unplaced scaffolds were excluded and chromosomal contigs were considered for binning. The number of NLR homologs in 5 Kb bins of each *Arachis* genome was obtained using “make-windows” and “intersect” commands of bedtools program^22^. Each bin was then manually labelled with serial number. Using bin number and NLR density value in each bin, linearized version of the genome was visualized using the Rideogram package^23^. To find the syntenic relationship between NLRs in *Arachis* species, respective BED files from each species (bin size = 5 kb) were used for the initialization of genomic tracks. BLAST is performed for identification of inter-species genomic similarities, then chromosome and genomic position were retrieved from the GTF file and subsequently sorted according to BLAST output. Genomic linkage was provided on collinearity bases between the genes. The R package “Circlize”^24^ was used for the visualization of synteny plots.

### Evolutionary analysis in ***Cicer*** NLRs

Clustalw was used to align each group of paralogs' deduced protein sequences across their respective subgroups (Li 2003). And the obtained alignment was used for a guide in order to align corresponding nucleotide sequences via the usage of the pal2nal software, which is based on the language Perl^25^. After removing gaps and N-coding codons, *ks* were estimated using ka/ks calculator under the MA method^26^. We performed the Fisher test on each paralog selection value, and significant duplication events were kept, and the rest of them were removed (*P* value > 0.01). Ks-values greater than two (> 2) were eliminated from further consideration since there is a possibility that they suggest substitution saturation. Orthovenn2^27^ was utilized to study orthologs cluster NLR genes. Identified putative NLR genes from each species were queried in locally installed Orthovenn2 program using an E-value of 1e-2 with default settings. All NLR genes identified were subjected to Orthofinder for orthology analysis^28^. Output containing orthogroups families were labelled manually and species tree was modified into a ultrametric tree using R package APE^29^. Both files were utilized as input for the CAFE5^30^ and resulting files were manually parsed to evaluate gene gain and loss at each node of species phylogenetic tree. Furthermore, the ortholog sequences between A and B genome of *A. hypogaea* and *A. monticola* and their ancestral sequences *A. duranensis* and *A. ipensis* were also acquired from Orthofinder^28^.

### RNA-seq based expression analysis

Basal expression level of NLRs identified from this study was evaluated using the available datasets of *A. hypogaea* and its related species (Table S1). The genotypes utilized different species genus *Arachis* for expression were different as compared to the genotypes utilized for generating reference genome sequence. First dataset provides comprehensive collection of replicates from pod, seed and shell tissues (PRJNA847769). In the current study, we aligned the raw read sequences using the reference genome of *A. hypogaea* (v.2) with HISAT^31^. Alignments were passed to StringTie^31^ for transcript assembly. Finally, the assembled transcripts and abundance were processed using Ballgown^31^ for grouping of experimental conditions and determination of differentially expressed between the conditions. In addition, two more datasets were analyzed from project number PRJNA706902, PRNA679430 using similar approach as described earlier. Furthermore, we also evaluated the expression of common NLR genes in progenitor, *A. monticola*, *A. hypogaea* and neopolyploids using *A. hypogaea* genome as reference using PRNA380954 dataset.

## Results

### Gene mining of NLR genes in ***Arachis*** species

**Figure 1:**
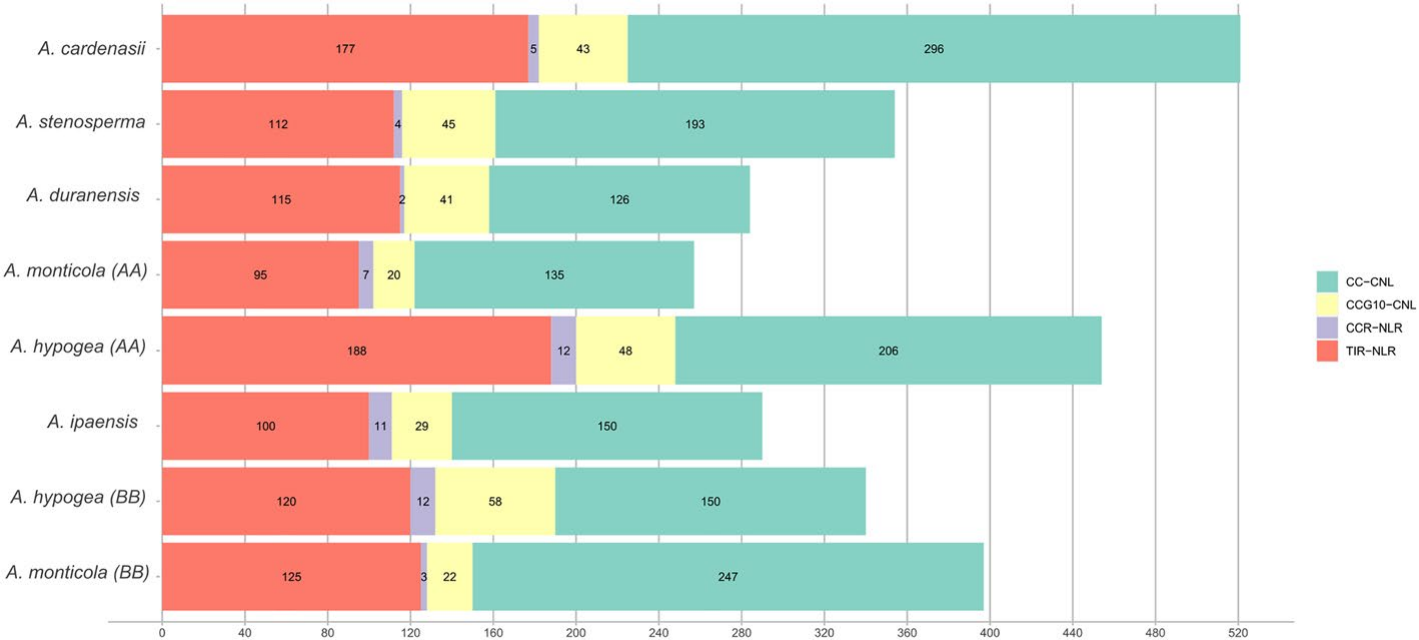
Inverted barplot represent distribution of four classes of NLRs in *A. cardenasii, A. stenosperma, A. duranensis, A. monticola, A. ipaensis* and* A. hypogaea.*

Here we utilized the NLR tracker pipeline^15^ for NLR genes mining and successive annotations. In case of wild diploid ancestral species for A-genome a total of 521, 354, 284 NLR genes were identified from *A. cardenasii, A. stenosperma* and *A. duranensis*. In total, 257 and 454 were identified from A- subgenome of wild (*A. monticola*) and domesticated (*A. hypogaea*) tetraploids (AABB) respectively (Fig. 1). Whereas, B-genomes species including *A. ipaensis, A. monticola, A. hypogaea* contain 290, 397 and 340 NLR genes in their diverse repertoire. Interestingly, A-subgenome from domesticated tetraploid species (*A. hypogaea)* revealed the significant expansion in the NLR in contrast to *A. monticola* where reduced number of NLR genes were identified. On the contrary B-subgenome of domesticated tetraploid *A. hypogaea* revealed contraction as compared to of *A. monticola.* In addition, among the wild species *A. cardenasii* shows the expanded NLRome repertoire among all *Arachis* species (Fig. 1). All four classes of NLR genes were present in all members of *Arachis* genus. Overall CC-NLR have shown the highest contribution among other classes, on average 51.35% CC-NLR, 35% TIR, 12.1% CCG_10_ and 1–2% CC_R_ -NLR genes were identified in members of genus *Arachis*. Interestingly, helper NLR were present in relatively large numbers in tetraploid species especially in *A. hypogaea* where both AA and BB genome shares 12 CC_R_-NLR and all the diploid members possess 3–5 CC_R_ except *A. ipeansis* where 11 CC_R_ helper genes were reported. It is consistent with the previous observation that polyploidization may increase or decrease the number certain genes families^32^, here CC_R_ shows symmetric expansion in *A. hypogaea* in both genomes*.* Distribution of NLR length, length of conserved NB-ARC and species wise domain organization is also provided (Figure S1, S2, S3).

### Landscape of NLR genes among genus ***Arachis***

**Figure 2:**
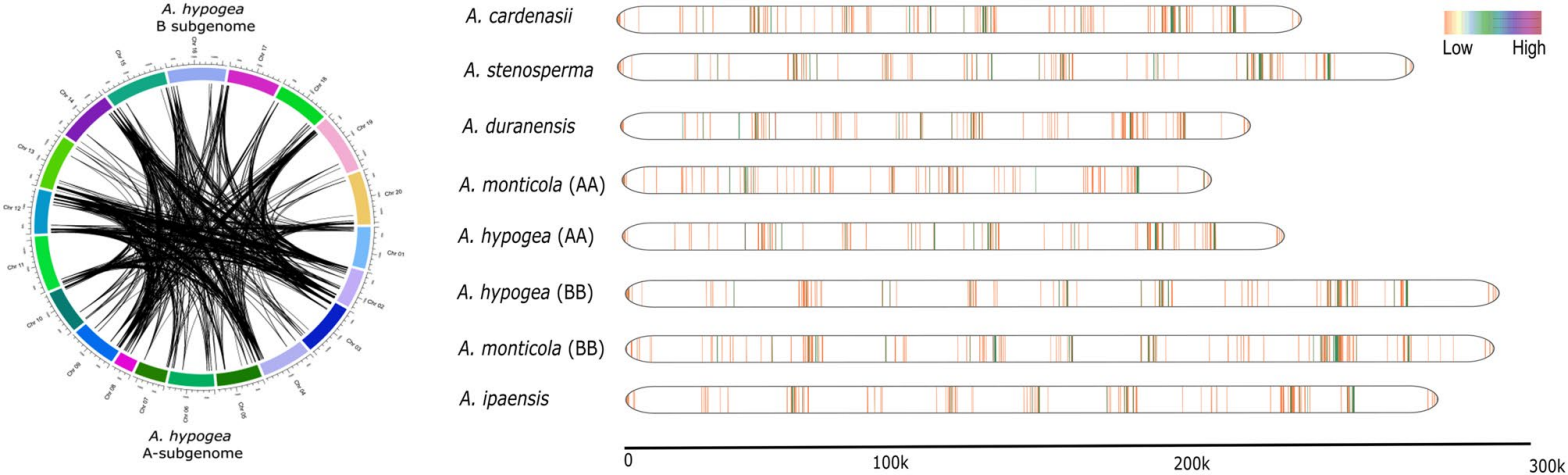
Synteny analysis and landscape of NLR genes. (**A**) Synteny analysis explores depth of evolution and conserved shared synteny between A and B subgenomes of *A. hypogaea.* (**B**) The NLR gene density map of all six species of genus *Arachis* on linearized chromosomes.

We also compared the syntenic relationship between *A. hypogaea* subgenomes and their progenitor. Highly conserved homeologoues clusters were identified in the syntenic comparison between A-subgenome and B-subgenome of *A. hypogaea* (Figure S4, A). It should be noted that less syntenic relationships were observed for comparison of each subgenome with its progenitors (Figure S4 B, C). We also studied the landscape of NLR genes in all eight genomes of *Arachis* genus by plotting the gene density of NLR genes on linearized chromosomes (Fig. 2). Interestingly, *A. cardeansii* revealed the highest gene density with respect to its size. We also observed the effect of allopolypoid in both wild and domesticated tetraploid species. Interestingly, A-subgenome have shown contraction in wild tetraploid and later on shows significant expansion upon domestication in *A. hypogaea*. On the contrary, B-subgenome of *A. monticola* expanded significantly after allopolyploidy with second highest gene density after *A. cardenasii (*Fig. 2)*.* Overall, synteny and gene density maps strongly suggest that allopolyploidization favors expansion in NLR gene density in *Arachis* species with the exception of A-subgenome of *A. monticola.*

In addition we compared the architecture of resistance gene clusters (RGCs) in each species of genus *Arachis* (Figure S10). Majority of NLR genes were allocated in the form of RGCs. Most of RGCs were allocated on Chr02, Chr04, Chr05, Chr08 and Chr09 (Figure S10). Highest number of total 29 RGCs were found in *A. cardenasii and A. stenosperma* and the least numbers were observed for *A. monticola* (A-subgenome). Interestingly, individual number of genes in each cluster were amplified in *A. cardenasii, A. hypogea* (A-subgenome) *and A. monticola* (B-subgenome) suggesting active role of tandem duplication in expansion of their NLRome. In addition, presence and absence of RGCs were variable in each genomes.

### Phylogenetic analysis and classification of NB encoding genes

**Figure 3:**
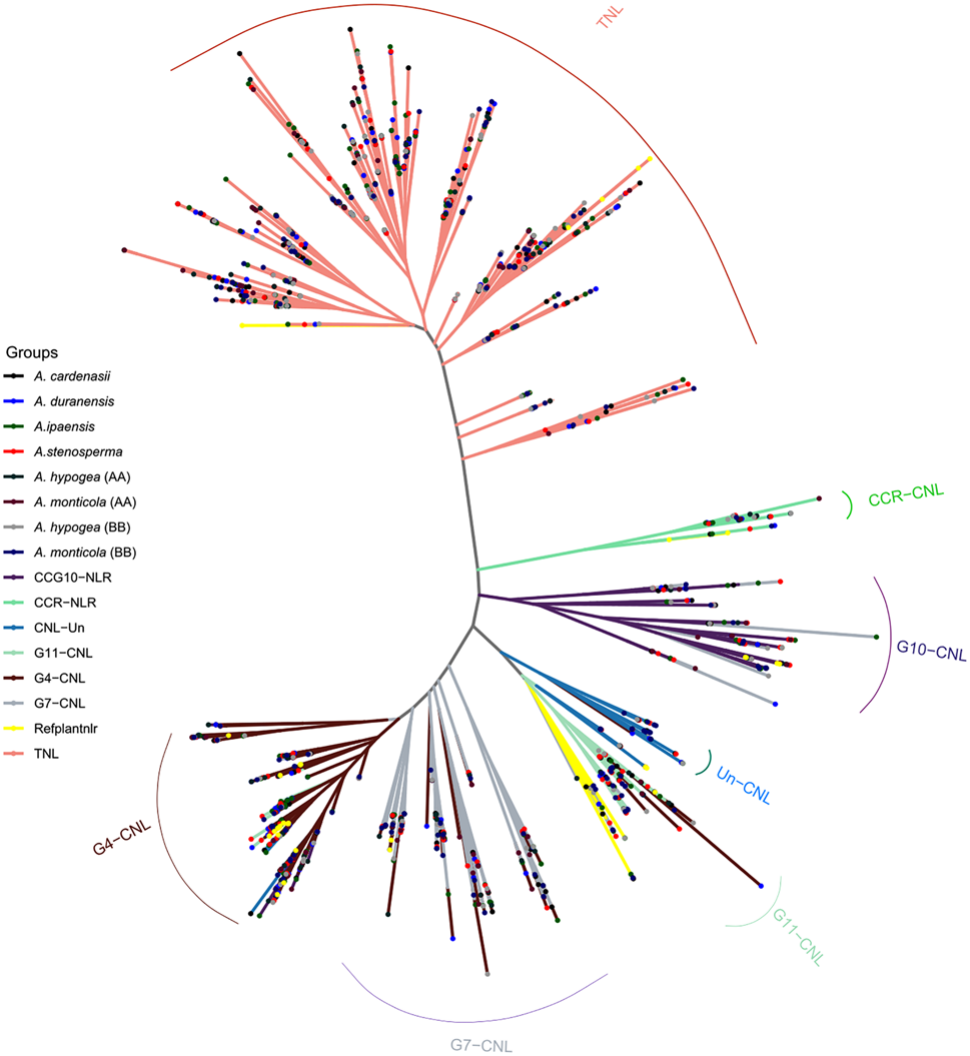
Classification of subgroups of NLR genes using phylogenetic reconstruction. Phylogenetic tree construction is based on the Maximum likelihood method on the VT + F + R9 model. The tree is divided into 7 CNL and 1 TNL subgroups. All the branches are highlighted with their subgroup-specific colors.

Conserved NBARC domain was extracted from each *Arachis* species and clustered at 75 percent sequence identity using CD-HIT^33^. Representative members from each cluster (total = 380) were utilized for reconstruction of phylogenetic relationship among *A. stenosperma, A. duranensis, A. cardenasii, A. monticola* (AA)*, A. monticola* (BB)*, A. hypogaea* (AA)*, A. hypogaea* (BB) and *A. ipaensis* (Fig. 3). TNL clade was branched out as expected, however TNL remained polyphyletic and three major radiations were observed. On the other hand CNL clade was divided in to three monophyletic major sub-clades CC-NLR, CC_R_-NLR and CC_G10_-NLR. CC-NLR was further divided in four major sub-groups CNL-Un, CNL-G11, CNL-G7 and G4. Significant expansion and diversity was observed in G4 and especially in G7 where four strongly supported polyphyletic sub-clades were observed. Interestingly, CNL groups G1, G2, G3, G4, G6, G8 previously identified from *Solanaceae* family were absent in genus *Arachis.* That is consistent with the studies from *Cicer* and *dalbergioids*, which strongly suggest that *Fabaceae* members lack G1-G8 groups^34,35^.

Phylogenetic analysis further suggest that progenitor of AA sub-genome, *A. duranensis* had less number of TIR and CC-NLR genes. After allopolyploidy significant expansion in TIR and CC-NLR genes can be observed. Highest number of these groups can be identified in AA subgenome of domesticated tetraploid *A. hypogaea.* Interestingly, among all species *A. cardenasii* has the highest number of TIR and CC-NLR genes considering its diploid nature. This unbalanced gene duplication occurrences across *Arachis* species suggest possible role of terminal duplication after the divergence from common ancestors.

We also compared the selection pressure within in the pairs of paralogs from four major subgroups (G4, G7, CCG10-NLR, TIR-NLR). G4 (Median = 0.502) and G7 (Median = 0.534) has the highest values of *Ka/Ks* as compared to other two major groups TIR-NLR (Median = 0.457) and G10 (Median = 0.427), which were evolving under purifying selection. This observation is consistent with the fact that preferential expansion of G4 and G7 is also observed in other *Fabacaeae* genus *Cicer* and *Dalbergia* (unpublished results). In case of *A. monticola* higher Ka/Ks values of 1.002 was observed for CCG10 subgroup that suggest that its evolving under neutral selection (Figure S5, S6, Table S3).

### Duplication assay

**Figure 4:**
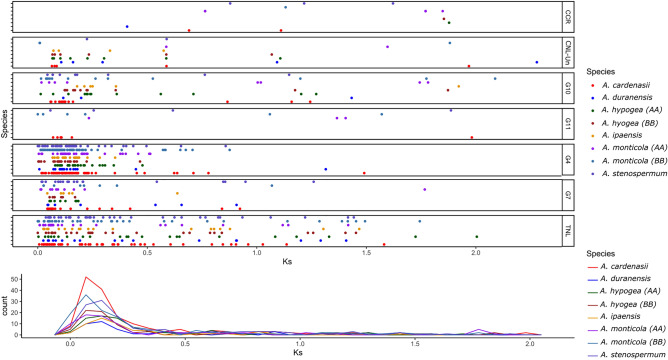
Duplication history of NLR genes in genus *Arachis.* Ks-values between paralogs of each subdivision are shown for all six species, where tetraploid species are divided into their constituent subgenomes. (**A**) X and Y represents the Ks values and frequencies, respectively (**B**). Overall duplication pattern of NLR genes in genus *Arachis.*

**Figure 5:**
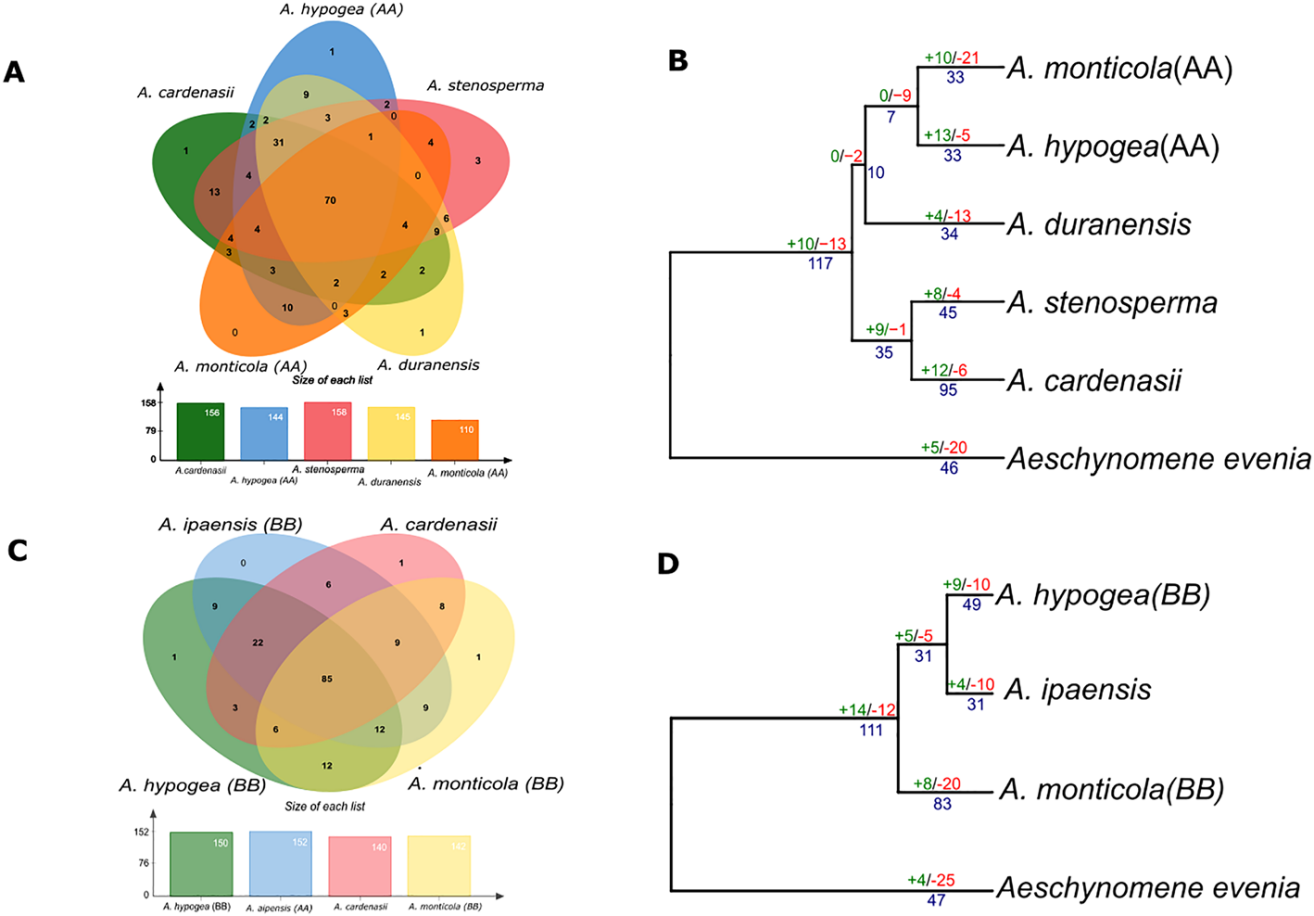
Ortholog and gene gain and loss analysis. (**A**, **C**) Venn diagram represents the shared and common genes (Orthologous clusters) distribution found between A genome related species and B genome related species respectively. (**B**, **D**) Gene gain and loss are indicated on each nodes with number of gene gain (green), loss (red) and duplication (blue) for A and B genome related species respectively.

Expanded NLRome of *A. cardenasii* could be because of multiple evolutionary mechanism including duplication, recombination and transposition. Here we explored the duplication history of *Arachis* NLRs by comparing the *Ks* values between paralogs of each subgroup. Notably the *Arachis* lineage have been rapidly accumulating silent changes (~ 1.4 time faster) since the divergence of the *Dalbergioid* clade^1^. The closest estimates for divergence between two progenitor of each AA (*A. duranensis*) and BB (*A. ipaensis*) sub-genome is recently computed as 2.12 Mya^32^. However, the precise estimate of divergence of other species from the common ancestor is still not reported. Collective *Ks* values obtained from all groups suggest one common duplication curve between 0.04 and 0.1 Ks (2.1–6 Mya) (Fig. 4, Table S3). That strongly suggest NLR gene duplication have occurred before the speciation. Highest frequency for gene duplication was observed in *A. cardenasii*, where peak value of *Ks* corresponds 0.08 (~ 4.92 Mya). TNL and subgroups G4-CNL, G7-CNL gene had been amplified dramatically through gene duplication events before speciation. Similarly other species *A. monticola* (B-subgenome) and *A. stenosperma* also revealed relatively higher frequencies of gene duplication. Interestingly, the progenitor species *A. duranensis* and *A. ipaensis* had the least frequency of *Ks* value for gene pairs. Furthermore, we also tested gene duplication using orthofinder (v 2.5.4: Fig. 5B, D). Consistent with *Ks* estimates, it suggests that in both A and B genome species highest duplication were observed in the common ancestor of *Arachis*. Furthermore, Orthofinder provides evidence for relatively higher terminal duplication in *A. cardenasii* (95) and *A. monticola* (83: B-subgenome) (Fig. 5B, D). In short, all species represents a common wave of duplication that led to major expansion in NLRome which occurred in the common ancestor of genus *Arachis*. In addition, terminal duplication was also observed after speciation in specific species that expanded the repertoire of NLR genes in *A. cardenasii* and *A. monticola* (B-subgenome).

### Gene gain and loss

A total of 70 common orthogroups were found conserved in A-genome related species whereas as B-subgenome has 85 common orthogroups (Fig. 5A, C). We constructed the phylogenetic tree for each subgenome with birth and death of genic events among members of genus *Arachis. Aeschynomeme evenia* was considered as most related outgroup for common ancestor of *Arachis.* Birth and death model of A-subgenome reveals that contraction of NLR gene families occurred in *Aesechynomene evenia* which is consistent with overall NLR contraction after whole genome duplication following diploidization. In addition, common ancestor of genus *Arachis* suggest increased number of gene duplication and gains of 10 additional NLR gene families (Fig. 5B, D). Progenitor of A-subgenome *A. duranensis* and wild tetraploid *A. monticola* has shown death of gene families except *A. hypogaea* where expansion in number of NLR families was observed. Similarly, A. *cardenasii* revealed the highest expansion of number of diverse gene families probably due to terminal duplication and gained 12 gene families (Fig. 5B, D). Similar trend was found in case of B-subgenome evolution, contraction in the outgroup species and expansion of NLRome in the common ancestor *Arachis*. Especially in case of *A. monticola* (B-subgenome) where expansion of NLR genes occurred that is consistent with expansion of other gene families including starch and sugar metabolism, linoleic acid metabolism and cutin synthesis^32^. In short, asymmetric evolution of NLR genes in A and B sub-genome was observed in wild and domesticated tetraploid species.

### Impact of natural and artificial selection pressure on NLR genes

**Figure 6:**
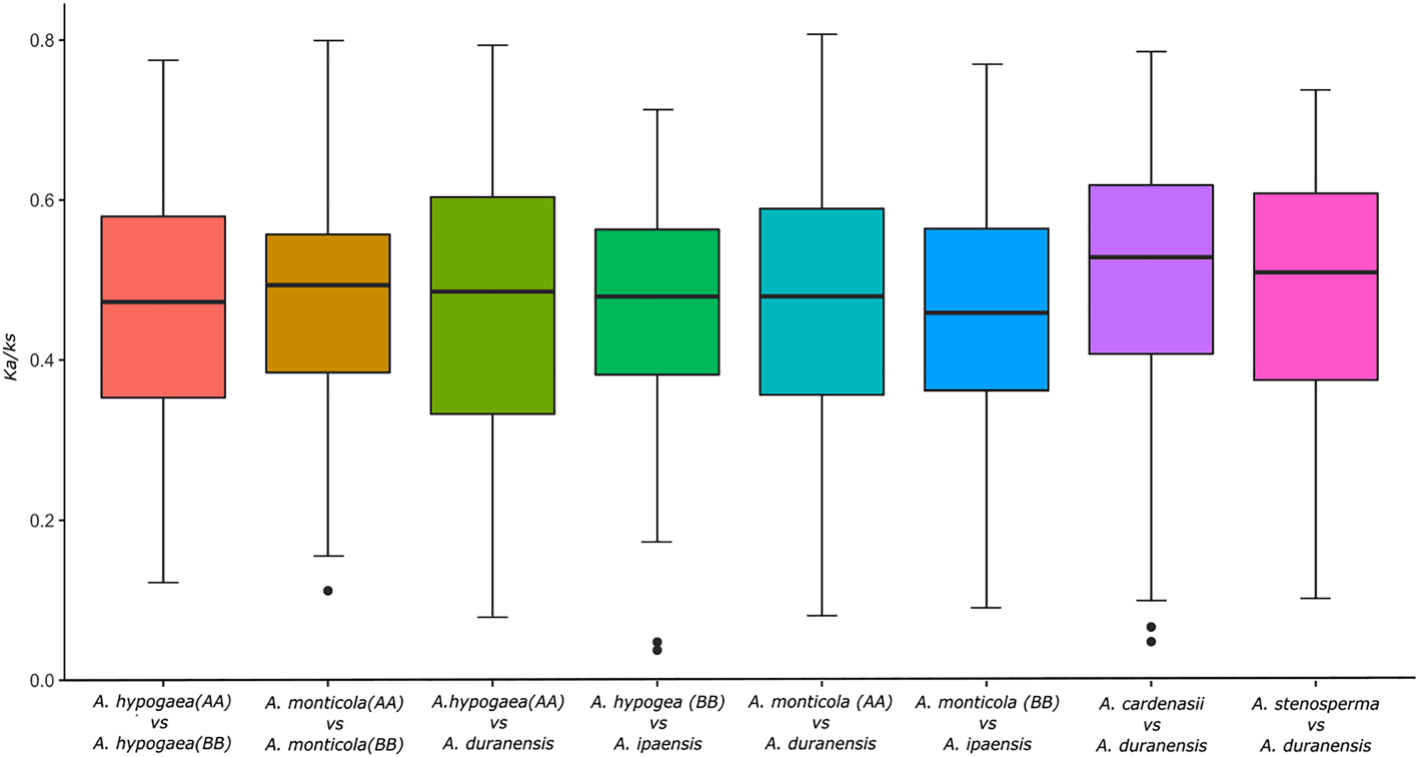
Boxplot represent *Ka/Ks* values between *Arachis* species. Middle line between each bare represent median of respective *ka/ks.*

We further studied the impact of natural and artificial selection on NLR gene evolution in both wild and domesticated tetraploid species respectively. For this purpose we compared the *ka/ks* ratio of orthologs present between subgenomes and their progenitor species (Fig. 6). *Ka/Ks* values for orthologs between A-subgenome of *A. monticola* and *A. duranensis* were significantly higher in A (median = 0.479) as compared B-subgenome (median = 0.455). Similarly, *ka/ks* values were higher in A (median = 0.488) as compared to B subgenome (median = 0.479) of *A. hypogaea* (Fig. 6). A bias was observed in selection pressure for A sub-genome NLR genes in both wild and domesticated tetraploid. We also studied the nature of selection pressure on two early diverged species of A-subgenome, for this purpose we compared *ka/ks* values orthologues of *A. cardenasii* and *A. stenosperma* with respect to *A. duranensis*. These species shows highest degree of natural selection as compared to other wild species, especially *A. cardenasii* with the selection pressure of M = 0.528. that potentially be the reason for expanded repertoire of NLR genes.

### Expression analysis of NLR genes in ***Arachis*** species

**Figure 7:**
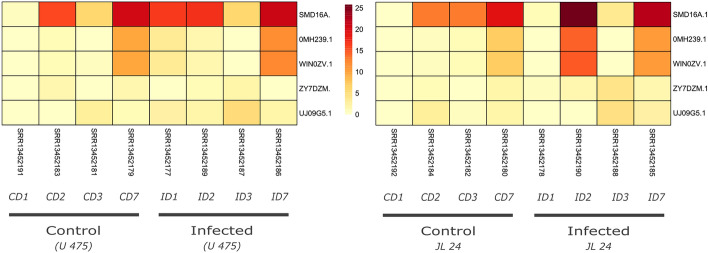
Comparison of NLR gene expression of susceptible (JL-24) and resistant (U-475) cultivar under *Aspergillus flavius* infection. Four time points including 1, 2, 3, 7 day post infection (dpi) were selected for the evaluation of their expression.

**Figure 8:**
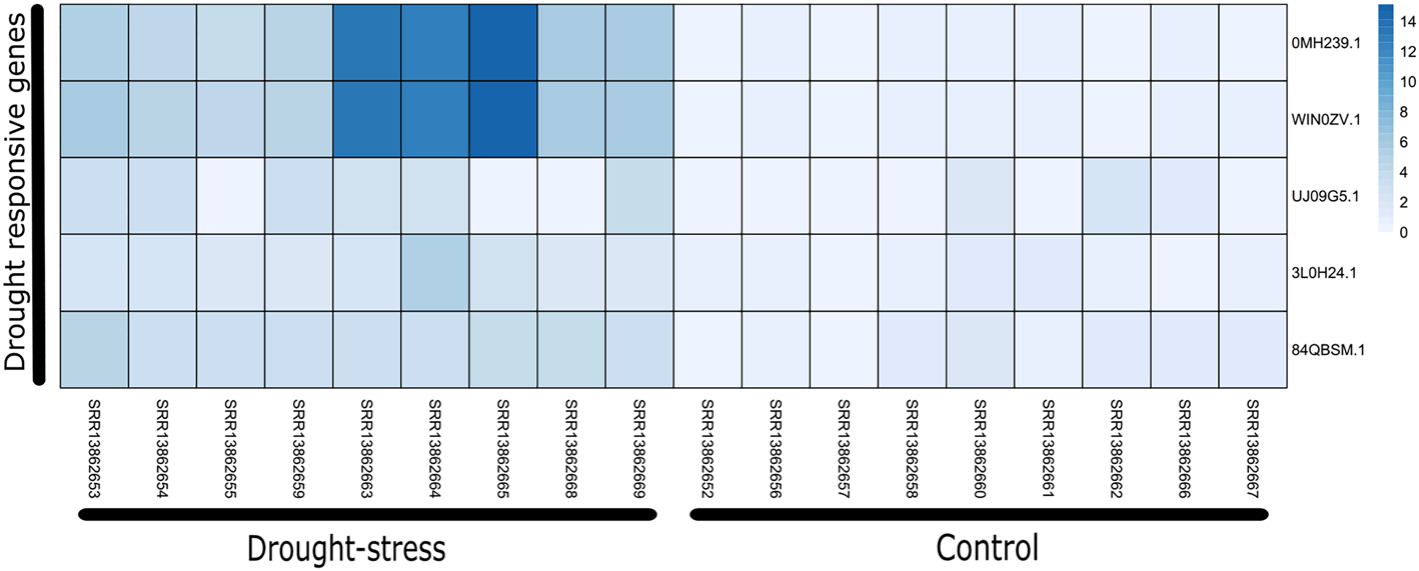
Comparison of NLR gene expression under drought conditions. Five drought responsive genes were identified indicated in left side.

We further compared the basal expression level of identified genes in *A. hypogaea* in three tissue pod, seed and shell. In total 37 NLR genes were constitutively expressed in all three tissues types, notably two genes *HV9GRN.1* and *256JRY*.*1* that belongs to subgroup CCR-NLR and CCG10-NLR respectively shows the highest expression levels in all tissue types (Figure S7). In another study, we evaluated the expression of NLR genes in susceptibe (JL 24) and resistant cultivar (U-475) of *A. hypogaea* upon *Aspergillus flavius* infection (Fig. 7). In both cultivars 12 NLR genes were differential expressed and showed strong correlation with disease progresssion. Especially three genes (SMD16A.1, OMH239.1 and WIN0WV.1) revealed higher up-regulation during 3 and 7 dpi in both cultivars (Fig. 8). All three genes are belonged to subgroup G4-CNL which is principal receptor containing coiled coil domain for recogniation of pathogens. Interestingly, no signifcant differences were observed in the expresion profile NLR genes in susceptible and resistant genotype. Presumably other resistance gene including receptor-like kinases (RLK) and receptor-like proteins (RLP) might be responsible for the difference in their genotype.

Recently, it was reported that NLR genes also plays important role under the drought stresss conditions^36^. Here we tested this hypothesis for *A. hypogaea* by comparing the expression of NLR genes under well watered versus drought conditions. In this dataset we identified five drought responsive genes (OMH239.1, WIN0ZV.1, UJJ09G5.1, 3L0H24.1 and 84QBSM.1) that were overexpressed during drought conditions. Since this dataset contains biological replicate for 5 days, 7 days and 9 days post drought situation (pds), interestingly we observed highest expression upto 14 fold in 9 days (pds) notably for two genes (0MH239.1, WIN0ZV.1) (Fig. 8).

We also evaluated the expression of NLR genes in both tetraploids and their progenitors as well as synthetic nascent interspecific hybrids and neopolyploids. Bertoili et al.^37^ reconstructed the hybrids of A. duranensis x A. ipaensis and subsequently induced polyploidy through colchicine treatment. RNA-seq analysis was performed on initial diploids, neopolyploids (1st and 9th generation). Conserved NLR genes that are common to major *Arachis* species has shown expression bias for interspecific diploid and neo-allopolyploids. Higher individual and cummulative expression levels were observed in synthetic interspecific diploid (AB, *A. duranensis* x *A. ipaensis*), neopolyploid (4x) and *A. monticola* as compared to *Arachis hypogaea* (Figure S8, S9). In short, NLR genes shows higher quantitative expression levels in *A. monticola* and neopolyploids (Figure S8).

## Discussion

Plants require repertoire of NLR genes for their consistent arm race with the pathogens. Plant genome utilized multiple genetic mechanism for the expansion of NLRome^38,39^. Tandem duplication is the major driver for their expansion. Secondly the cluster of NLR genes are quite conserved that may cause birth and death of NLR genes via unequal crossing overs or gene conversions^38,40^. Another mechanism of NLRome expansion in certain plant species is polyploidy, Genus Arachis presents a unique opportunity to understand the evolution of NLR genes, due to presence of diploid progenitor, wild and domesticated tetraploid species. Under normal conditions domestication causes narrowing of genetic base that leads to the loss of important gene involved in biotic and abiotic stress tolerance^41^. Here we have screened 4 wild diploid and two wild and domesticated tetraploid species to understand the evolution of NLR genes. We employed four major analysis to understand the evolution NLR genes in *Arachis* that includes gain and loss of NLR genes, their distribution, phylogeny and duplication assay. These analysis strongly suggest that expansion of NLRome in A-subgenome of cultivated *A. hypogaea.* Similarly, considerable expansion of NLR genes were also observed in B-subgenome of wild tetraploid species *A. monticola.* In short, our analysis provide basis for asymmetric expansion of NLRome in cultivated and wild tetraploid species. Similar trend were observed in another member species of Fabaceae family, where the allopolyploid *T. repens* have shown biased expansion of NLRome in A-subgenome^42,43^.

All diploid wild relatives has shown slightly slower rate of evolution as compared to tetraploid species with the exception of *A. cardenasii.* This wild species shows the most expanded NLRome in genus *Arachis.* It has been utilized for the development of disease resilient cultivars in Africa, Asia and Americas. The contribution of this species provide widespread improved food security, environmental and economic benefits^41,44^. Here in this article we have highlighted the evolutionary mechanism of expanded NLRome. Highest gene duplication frequency with terminal duplication, gene gain and rate of natural selection are the main reason for expanded NLRome. We observed the preferential duplication of subgroup G4 and G7-CNL. Comparative transcriptome analysis of *A. cardeansii* under infected versus non-infected conditions will allow the identification of effective NLR genes. However, limited dataset were found in databases. Therefore, it is important to generate more genomic and transcriptome resources for the identification of novel resistance genes from *A cardenasii*.

Reconstructed tetraploid species through *Arachis* wide crosses can generate spontaneous diversity. Recently generated neo-polyploids from interspecifc hybridization of *A. ipaensis* versus *A. duranensis* provides enhanced novelty that can broaden the phenotypic and genotypic plasticity through the mechanism of heterosis and gene redundancy^37,45^. We tested the expression of NLR genes in different generation of neo diploids and polyploids, our results strongly suggest enhanced qualitative and quantitative expression levels of NLR genes as compared to established polyploids. It might be due to the fact that earlier interspecific hybrids are relatively unstable and shows less regulated expression of certain genes. In addition higher genomic unstable lines tend to perish and only few lineages survives that had stronger mechanism for limiting genomic instability^37^. Earlier lineages of neo-polyploids can contain novel resistance gene combination that can be introgressed in the cultivated elite lines through conventional and modern approaches.

Polyploidy have played a major role in the expansion of NLRome in genus *Arachis*. Our results strongly suggest that NLR gene family follows a global trend of asymmetric sub-genome evolution between wild and domesticated tetraploid lineages. It could be due to homeologous sequence exchanges (HSEs) between subgenomes and high frequency of gene duplication. Homoeologous recombination does not only have altered the gene dosage due to chromosomal rearrangement but also results in novel transcript and intergenomic recombinant proteins in nascent allopolyploids^5,37^. In future HSEs should be studied in detail for understanding the expansion of both nascent and established allopolyploids of *Arachis.* In addition, structural variation (SVs) also play a pivotal role in the evolution of various gene families across different polyploids e.g. cotton, brassica^6,32^. In future, we will explore the role of SVs on the evolution of NLR genes and its related families in genus *Arachis.*

## Supplementary Information

Supplementary Information 1.

Supplementary Information 2.

Supplementary Information 3.

Supplementary Information 4.

Supplementary Information 5.

Supplementary Information 6.

Supplementary Information 7.

Supplementary Information 8.

Supplementary Information 9.

Supplementary Information 10.

Supplementary Information 11.

Supplementary Table S1.

Supplementary Table S2.

Supplementary Table S3.

## Data Availability

Library of identified NLR genes with their comprehensive classification is provided in the supplementary data file. Additional raw and refined output data will be available on request to corresponding author (saad.serfraz@gmail.com).
