# Supplementary material for: Evolution of NLR genes in genus Arachis reveals asymmetric expansion of NLRome in wild and domesticated tetraploid species
Source: Sci Rep. 2023 Jun 8;13:9305. doi: 10.1038/s41598-023-36302-1 (PMC10250334; doi:10.1038/s41598-023-36302-1)
Supplement: Supplementary file 1 — Supplementary Information 1. [file 41598_2023_36302_MOESM1_ESM.docx]

Supplementary Material

**Legends to Supplementary Figures**

**Figure S1**: NLRs organization and their distribution among the genus Arachis and its species *Arachis hypogea*, *Arachis ipaensis*, *Arachis duranesis, Arachis monticola, Arachis stenosperma* and *Arachis cardenasi*. Fig A *Arachis stenosperma* NLR length, in stenosperma NLRs coiled-coil NLRs (CC-NLR) from CNL group and Toll/interleukin1 receptor (TIR-NLR) from TNL subgroups, are most abundant. CNL initiates from 200 to 1300 amino acid sequence and TNL occurrence initiate from 250 and ends at 1200. Fig B *Arachis ipeansis* NLR length The NLRs subgroups and their occurrence in ipeansis are variable to each other. The prominent subgroups are CNL(CC-NLR) and TNL(TIR) subgroup. The CNL(CC-NLR) sequence occurrence starts from 200 amino acids and ends at 3500 amino acids. Conversely, the TNL(TIR) group also starts from 200 to 3000 amino acids. Fig C*Arachis duranesis* the pattern of occurrence of CNL is from 250 to 3000 amino acid sequence and TNL starts from 300 to 3000. Fig D *Arachis monticola*(B) NLR length in monticola the starting point of CNL occurrence is from 300 to 3500 and TNL is from 500 to 2800 amino acids. Fig E *Arachis monticola*(A) NLR length in monticola the starting point of CNL occurrence is from 300 to 3200 and TNL is from 400 to 2400 amino acids. In all species, CNL is in a wider range than TNL subgroup. Fig F *Arachis hypogea*(B) NLR length  in hypogea the starting point of CNL occurrence is from 200 to 1800 and TNL is from 250 to 1600 amino acids. Fig G *Arachis hypogea*(A) NLR length  in hypogea the starting point of CNL occurrence is from 100 to 1800 and TNL is from 150 to 1700 amino acids. Fig H *Arachis Cardenasii* NLR length  in Arachis the starting point of CNL occurrence is from 300 to 3800 and TNL is from 400 to 3200 amino acids.

**Figure S2**: NBARC organization and their distribution among the genus Arachis and its species *Arachis hypogea*, *Arachis ipaensis*, *Arachis duranesis, Arachis monticola, Arachis stenosperma* and *Arachis cardenasi*. Fig A *Arachis stenosperma* unique NB-ARC length, short and long NBARC domains, have been identified. The longest NBARC domain is CNL(CC-NLR) and it starts from 50 to 400 amino acid sequence variation there are 60 short NBARC domains from amino acid sequence variation of 350 AA. These domains are analyzed at different standard deviations Fig B *Arachis ipeansis* unique NB-ARC length one parabola of NBARC domains have been identified. In this parabola, 68 short NBARC domains from 320-350 AA have been found. Fig C*Arachis duranesis* unique NB-ARC length one parabola with 47 short NBARC from 330 to 360 AA is identified. Fig D *Arachis monticola*(B) unique NB-ARC length one parabola with 30 short NBARC from 300 to 380 AA is identified. Fig E *Arachis monticola*(A) unique NB-ARC length one parabola with 30 short NBARC from 300 to 380 AA is identified. Fig F *Arachis hypogea*(B) unique NB-ARC length one parabola with 25short NBARC from 330 to 380 AA is identified. Fig G *Arachis hypogea*(A) unique NB-ARC length one parabola with 47 short NBARC from 300 to 400 AA is identified. Fig H *Arachis* unique NB-ARC length one parabola with 20 short NBARC from 250 to 360 AA is identified.

**Figure S3**: Structural architecture of NLRs domain of genus Arachis and its species *Arachis hypogea*, *Arachis ipaensis*, *Arachis duranesis, Arachis monticola, Arachis stenosperma* and *Arachis cardenasi*. In all species, the ratio of occurrence of CNL and TNL is 60% and 30% respectively. In Fig A *CC-NLR are found*. Fig B *Arachis ipeansis* Fig C*Arachis duranesis* Fig D *Arachis monticola*(B) Fig E *Arachis monticola*(A) Fig F *Arachis hypogea*(B) and Fig H *Arachis* unique NLRs/domain architecture both CNL and TNL are found but CNL are relatively more abundant

**Figure S4**: The NLR integrated domain architectural analysis among *Arachis*. The domains are indicated in different colors. The black bar represents the occurrence of transposons or it can be another additional domain. The domain architecture is diverse. Its novel NLRs are fused with many transposons as an additional domain.

**Figure** S5, S6: Ka/Ks values plot for each subgroup of eight genomes of genus *Arachis*

**Figure S7**: FPKM value of genes in POD, Seed, and Shell of Arachis. Shell have relatively higher FPKM values as compare to Seed and POD.

**Figure S8**: Comparative expression of NLR genes in *A. hypogea, A. monticola, A. duranensis, A. ipaensis* and their reconstructed diploids and tetrploids. Higher number of genes and expression can be observed in reconstructred diploid and *A. monticola.*

**Figure S9**: Cumulative FPKM values of NLR genes in extant and neopolyploid species

**Figure S10:** Resistance gene clusters (RGCs) distribution across the genome of *Arachis* species

**Supplementary Tables**

**Table S1**: Genome Resources of *Arachis hypogea*, *Arachis ipaensis*, *Arachis duranesis, Arachis monticola, Arachis stenosperma* and *Arachis cardenasi*

**Table S2**: Selection rate in *Arachis species*

**Table S3**: Ortholog selection ratio in *Arachis*.

**NLR sequences from Genus *Arachis***

**Arachis hypogea (A subgenome)**

>arahy.Tifrunner.gnm2.ann1.00IH7J.1

MAESLLQMVIENLQAFAQDELATLWGVHSQIQELSGNLAAIHAVLQDAEEKQIRERAVKLWLQKLSDAAHVLDDILDECSIESNRLHSDQCLTRLDPVTIIFRRDIGKRMKEMVDRFRQIDEERRRFELRGRVPERQQENEAWRQTCSGITEHKIYGREQDTENIVEFLSRSADSSNDLSVYPIVGMGGLGKTTLVQWVYNDKKVIEHFHLRIWVCVSTEFNTMRILESIVESTSGHNPNLSTLEALKNKVQEILLGKRYLLVLDDMWSTDKWEDLKSVLLCGGGTKGAAVLVTTRVESVASVMGTCPAHHLSPLSQDDNWLLFKYHAFGSDKVERRELVAIGKEIVKKCGGSPLASKALGSLLRNKKEEIQWVNVLESKFWDILEDDAIIVRALKISYFHLKLSLRQCFAFCAIFPQDFRMEKEQLIHLWMANGLIKSKGKLEIEDVGNEAWEELCQRSFFQEVEIDELGRTTFKMHDLFHELAQSIMGEECRVYDESASLTNLSTRVHHVTCLKPEMEVNMDPFKKAESLRSMINLHPFDHRNLNGLPPFNSLRALRTNASQLSALKSLTHLRYLNLRRSGITTLPECVSRLQKLQILKLEDCLYLSCLPKHLTQLKDLRHLLIEECHSLVEMPPNIGELKCLRTLNLFIVDKKEGRGLSELRDLQLGGKLRIKGLENVINEGDARDANLSAKKKLENLYLSWGSSDSRRGANAERILEALEPPSNLKSFGMNGYSGVELPSWMQNTSILSSLVMVILYDCKNCKHLPPLGKLPHLTVLYVSGMKDVKYIDEDSYDGVDEKAFKSLKDLTLSKLPNLEGMLRDERVEMLPVLSKLRVSCVPKIKLPLLPSLEHIWIEGTGSDSDHGDSDSEDMALSFLEAIVENMRRVKVLHIEHFPTLKALPHELGSLNSLQELELYFCDRLESFSENVVQGLGSLRTLTINRCKELKSLSEGVRHLTCLERLDIIYCPKLVTLPSSMNQLVSLRHSFICSCDTLPEGLQHVPSLQSLDVCDIRSIPEWLGDLTSLQKLRLSSKGLRSLPSSIRNLPNLRELSIDGCHKELQKRCTKVTGQDWQAIAHIPQFKLVPIHEETFSGFSLWGGVLAQVIAIATCGLIFAECDVLIVAWFQFPIMALYIQLHTLAMQLA

>arahy.Tifrunner.gnm2.ann1.01K7X8.1

MAAHASSEIIKYDVFLSFRGTDTRCGFLSHLRKELEDKHIKTYVDNMLREGTEISHSLLAAIEQSEIALIIFSQDYASSKWCLEELAKIMECRKQNGQIVIPIFHNVDPLWVRHQKESYHHALANHEVRFADRVQIWRDALKEAANLSGFDSQSSRFKDDADLVGQIVKRVLQRLNQSPQGDLQGLVGIHGPIEKLVSLCTESEDVIIGLWGMGGVGKTTLATAVFNRLCDGFEGFCFLKNVRERAEKYGIDHLKTELLSKLLKEEDASPFVTPGGITNFAKKRLGRTKVLVVLDDVNDSDQMEDLCGGHTRFGASSKIIATTRDKHVLLRADTDHIHQVKTLNSDDSLQLFSLNAFKQNCIIKAEQVELSKRVLNYCKGLPLAIKILGSFLKGKTQQEWESELAKLEKTPDEKIQRILRLSYNELDRNDRNIFLELACFFDRNTEEEEQIKSLLDHCGYTTTIGLTNLCNKALISISNDCVSMHDLIREMGREIVRGECLDDPGKRSRLWDSCDTYEVLKYNKVSN

>arahy.Tifrunner.gnm2.ann1.04BQTE.1

MAAAIVGEALLSAAVEALVGKISTEISEFYQSKKLDESLLEKLKLTLLSLHAFLDDAEEKQIKIASVKAWLDELTQALFDADDLIDDIATEALRRKVEARYHQTVSSKVRKVLSSPFKWPYREINSKMQKLFERLEHFAQRAHNLPLEKGVSGNVWRATPTNSAFDDSAICGRNDEKQNLKEYLISEDAADDGGNKIGVLAIVGMGGLGKTTLAKLLYNDAQVNEKFDVKAWASVSKDFDVVKLAKSLLESVTSAATTLDNFDTLRAELQKKLSDKRFLLVLDDIWNARYVDWTNLMHIFNVGQMGSKIIVTTRHQNIVDIVKAMRTCNLEPLANEDCWTLLSKHAFRTPKCTELSSNLEEIGRKIAQKCGGLPLAAVAVGSLLSTKLSTEQWSKVLNSNIWHLTGDEVQPALLLSYHFLPASLKQCFAYCAIFPKNSKLQKEKLVQLWMAQGFVFVSQNEKSIEEVGGEYFDELVARSLILRSAGGEHFEMHDLINDLATMVSSPYCKRHDNEMQLRNLNKIRHLSYDKSMFNHFGELDSLHGLKGFRTLTALPFEFNLLVWGHHLANGVLHELLVALKQLRVLSLNITVLPNSIGDLKHLRYLDLSCTRIERLPSAICKLYNLQTLLLTCCKDLTELPEEMGKLVNLRHLAIDYCWALTKLPEEMGKLVNLRCLDIEGTKLQEMSVEIAKLENLQTLSDFIVSKQQHGLKLAEMRKFPHLQGQLCISKLENVIDPSDACQANLKEKNQIEELSLEWSYSILEDDSHQVVLEHLQPSTNVKKLSVECYGGSTFPSWLGDSSFGNIVSLRIEECHHCSSLPPLGRLHSLKELFISGTRSVKTIGSEFYGGNSPSFQPFPSLETLSFGSMEEWEEWNMIDGIITEFPRLSKLSLRRCPKLKGNLPSNLPCLVTLDVKDCCVLESEFSGEVDNRNIMRPLNVFNFNSLQQLSLYHIPFLMSFPNNGLPKALKTLSIEYCESLEFPTHEFLHSCKALEDLYISSSCCSLTSFPLGSLPVLKSLKLWGCKKLKSISILEEAAAIQSLMFLEHLEVYGCPELESISSTPNLSYFSVKNCDKITSLPEPINNLTGLHSLYICDVPNLESIAEEGLPINLTTLGVGNEEGVYSNTAITKWGLDRLTSLSRLWIKGEYLVKKLMEIQVPLLPNSLGMLDIEGAREIQHLDGKWLQHLTSLEDIELSECDKLKSLPTEGLPSSISRLHIWRCPIISEEPPPTPSTLLWSWFDWDDNISETITKKIQLQKGTKSEQLKEIKVNDSSKTFYNRKDALWIKGIRMNHLPAEHASYQSSLHLVPDVEKSPIPNFQTSEYV

>arahy.Tifrunner.gnm2.ann1.04X3C9.1

MAAELGGAFLSSFLNVLFDRLSDPDIINIMRGRKVNQKLLQKLKTILNVVEAVLNDAEKKQITDSAVKRWLEDLQDAIYDADDLLDEVATKAATQKNPPGNFLSRFLNLQDREMVTRIEDIIVKLEEIAKHKDILRLEKITAKNMSGRIPSTSLVKKSDIFVGRDKERHTIMKLLLDDANDGELSVIPIVGMGGIGKTTLAKLVYNDDKVKQKFHVFKAWVCVGEEEFDVLKVTKALIEKICSPCYSNDLDTAQNHLKNALAGKNFLVVLDDVWSSNRERWESFLNPFECGTEGGKILVTTRLDTVASLVKTKHTEAHNLSLLDEDQCWSVFANRAWDPTESRDHSALEEIGRKIVKKCKGLPLAAQTLGGLLRGKDNEKDWNGVLNSEFWELSEEDSGILPALRISYYHLPSYLKRCFVYCSLYPKDFEFDRDELTLLWMAEATFYGGKFFSGILELKNAAKHDTKTRHVSCARNNDDDSLMKIMEACNRLKHVRTLMQINLHKGGVIPEGDGVTVPCHLLEQLKCLRVLSFKFFSDDENLLHGSIGKLIHLRYLDLSYTSIVTLPESLSCLYNLQTLKLRSCKNLKQLPSNMQNLVNLRHLDLFDTGLKEMPKGMSKLKDLQFLSNYIVGKHEENAVGELGELTHLHGSLSIGKLENVNNSGVASNARMDQKIHLNALYLMWSSFEESEVCDSQTEKDVLDKLRPHKGLKKLFMWGYRGTMFPDWVGQPLYYNMTQLQLGGCRNCWVLPSLGQLPSLERLVISELDKLKMIGGSFYKGDGTHQHQETPFRSLKSLEFYDMGCWEEWQSYECDDYDDAPFPKLEELHILHCPKLRGDLPTFLPSLKELRINECEELGCYLPRAPIMRQLLIYGKQEARMWDLPLSLQRLIIEGNQLVEYVFEAIIHTRPTSLNYLRISNCSSAISFPGDSLPPSLKELTIENCKNVEFSIQHQQQHHSLDKIIFLQWLSDEYIDVVDSQIERGVLDNLRQWKKAGIVESWKHFLLALYIEHSLLGQRMKEMNLERAIIYGWKELDVIIDALGKMMIDELSEVIIKILHGWKEIGV

>arahy.Tifrunner.gnm2.ann1.06IH0R.1

MAAELVGGAFLSSFLNVLFDRLSDPEIINLIRGKKLSEKMIQRFKAILNGAEALLNDAERRQIREGPVKIWLDDLKDAIYEADDFLDEIATKAATKKDRGNCLTRFLNLKDRKKVTRMEDVIARLESIVNQKDTLGLKEIPMENMSWRTPSTSLVKVSDIYGRDEDRKALVKLLLDDANDGDVSVIPIVGMGGIGKTTLAQLVYNDDQVKQKFDVKAWACVGEDFNVLRLTKTVIEEVTSKSCELNGLNSVQQRLRDEVTGKSFLVVLDDMWTNHYDDWKTFLIPFQCGSQGGKILVTTRIDVVASMVKTIPAHNLSLLDDEQCWSVFANHAFFPTESRDRLALEKVGRKIVDKCKGLPLAAQSLGGLLRTKDNIADWEDVLMSEIWEFSEDECRILPALRISYHYLPSYLKRCFVYCSLYPKDYEFDKDELVLLWMAEDLLQQPKSGSILEEVGYKYFNDLAAQSFFQPSKNGYVMHDLMHDLATFYGEKFFVRISEHENVAQHDTKTRHLSYDLNDNNSVLKMLEACESSSHVRTLFQIKADLYRGRKEGIDPCGLLAQLKCLRVLSFTSFKIDRLPDSIGELIHLRYLNLSNTLVLTLPKSLDNLYNLQTLKLAECKKLKKLPSNMQNLVNLRHLDIGGTELEEMPKKMRKLKDLQFLSDYIAGKHEENGIGELGELAHLHGSLCIQELENVKNSGEASNARMDEKIHLNALHLWWSSFEEREVCDSHAEKDVLDKLRPHKDLKKLFIEGYRGTMYPDWVGQSSYHNMT

>arahy.Tifrunner.gnm2.ann1.071IAB.1

MASALGEGEASSSTATRDSCKHQVFLSFRGEDTRKGFTDHLSASLERRGITAYRDDKNLEKGDAIQDELLKAIEESMFAVVVLSPNYASSSWCLDELHKILECSKNHGLSVVPVFYNVEPSDVRHQKGNFKKALRKHQNRFGQDSDRIRRWTDALTQIASFSGWDSKNQSYFQNFHLAQKILLGFFQRWRCVGIWGMGGIGKTTVARAVYEAIQSKFQVCCFMRDVREVSKAKGFVHLQRLLLSTLNVSDEFYDIEDGKKMIRNWLCKKKVLLVLDDVSEEIQLENLAGKQDWFGPGSTVIITTRYMHLLEIHRVNGTYKVEGLVEEEALRLFCLKAFKRDQPEEGYLDLSKDIVKYTGGLPLALKVLGSYLCGRDLHFWHRTARELGSVLPSEILNTLKISFDHLEPTEKYIFLDIACFFKGMNRDEVVNILQMCDYYDGIENGIATLIEKSLITLSKDEMLEMHDRLQDMGKNIVFQEFPTNPGKRSRLWSKKDIDQVLTNDMGTEAIQGIVLHSSYEAQWSIEAFSKTSQLTFLSLHGMKLPLGLNYLPRSLKVLHWNYCPLKTLPLADQNVSENLTGTPDISGAPMLQKLNLQFCSGLSEVHPSLTHHENLVQLDFSNCTSLKTIPGKLKMSSLEELDLSNCSSFVNLPKFAECMKLSLLSLKRTSIKNLLRSVSSSLPLLCSLDLSCCNLTELAFPCNIFHLPLLTDLNLSRNKFVHVPISLHKLPKLKRLCLNNCPNLKSLPVLPSSIEILEACNSFIAYHETGFDRCESIFNFFRSSSSDRSQVFIMEFTSWIYTEEKVSPWQSFLSFNLEDGEHLSLPPQGDFAPNDRLGIILCFQTATYLNKTDLCVCNGRRWITKAIAAESCPDYTFKLSVNYLNHNMQKAWSYGSNQCWGRWVHQQDFID

>arahy.Tifrunner.gnm2.ann1.0C7DFX.2

MASTSCSASIPPPPPRSCTYHVFLSFRGEDTRTGFTSHLYAALRRKGITTYKDDNNLRKGDIISDELLKAIEESMFAVIVFSPDYASSSWCLDELQKIMECNNKLGLQIEIVFYGVKPCDVRHQIGTFQEAFKKHEQRHDTEKVQRWRDALKQVAAYSGWTSKNQDEAVLVENIAQHIFEILIPKLPSSMKNLVGIESRVEQVISQIGLGLNDVRYIGIWGMGGIGKTTIAGVVFETIRSRFEVACFLADVREQCEKKDITHIQLQLLDQINMSSNAVHNKYHGRTMIQNSLHLKKVLLVLDDVNHEKQLEDLAGEKDWFGPGSRIIITTRNVEVLKEQEVHETYKVEGLVESAAFNLFCLKAFKKPEPTEGFLDLSKKVIKYSGGLPLALKVLGSYLNGRSIAVWHSAIEKIMRFSHSEIIDVLKISYDGLDSMEKNIFLDIACFFKGCEKDYATQILEGCGYHAEIGIDILINRSLVTISDYGILGMHDLLEEMGKLIVIQESPDDASKRSRLWCYEDVDFVLTQKKETEATHSIVLHKVNMKTEGYRRDLSFSNMCKLKLLILGGLVAPILCDIPCTLKVLHWRGCPMETLSFTDQCYELVEIDLLGSKIVQLWDGKKVLKKLQHLNLSSCHKLKQTPDLSGAPNLKTLHLEHCYELNYIQPSLAHHKSLVELDLGQCKSLETLGDKLEMSSLETLDLNGCSSIEELPPTLGNLAGVSELDLTGCDKLT

>arahy.Tifrunner.gnm2.ann1.0DZS3V.1

MYIFMLSNCDIEWCRSKLRQTGCTSLSPMSSSKAFQIKLVVFVGLLFVSKFKGMEENMMSENSYSYTDKTLTFPAPQIKYDVFVSFRGTDIRQGLLSHLIKAFHLKQVFAFVDDKLERGDDISDALLGAIEKSLISLIIFSQDYVSSRWCLEELVKIVECREKDGQTVIPVFYKVDPSDVRYQKGTFANVFAEHEERYGMIKVQNWRTALKNSADLSGFHSTNFRNDAQLVDQIVDFISSRLNDMHQVKSKGLVGIHKPIAQLESLMLKESEDVRVIGIWGLGGIGKTTIAEEVYNRMCAKFESCCFLANVRKESERDGIMSLKKKLFSTLLNEQNLRIDLPKGLPDYVEKRLGRMKVLIVLDDVNDSDQLETLIGARDWFGLGSRIIITTRDKQVLVGEAEEIYQVEELDCDESLELFILNAFKQNQFEMEYCELSRRVVKYAKGIPLVVKVLAQLLRGKRKDIWESQLEKLKRMPNKKVHDIMRLSYDDLDRQEQQIFLDIACFFNGLKLKLEYINVLLKDQDYAVAAGMERLKDKALIIISEHNIVSMHDIIQEMAWEIIKDGSAEYPGNQRRLWDSHDIYQVLKNNRGNEAIRSISFNFSAIKDLQLSQEVFAKMSKLRFLNFYSNISQEATYSSDGLPSSWNKGLCLPQGLDSLSNEVRYLSWMHYPLDALPTNFSAENLVILDLSFNRMEKLWCGVKNLANLRILRLYNSLHLTMLPDLSKARNLEILVIRKSFSFLYVHPSVFCLNKLEILDLGGCVSLRQLRSDIHLSSLRSLSLAGCVRLQDFSVTSENLEELNLELTGIQQLPTSFWRQSKLETLHLGLSDIQSIPETIKNLTRFKYLSIRYCWNLRDLPELPPSLEYLDATGCASLKTVLFPSSAALQLKENRKSILFWNCLKLDQHSLGAIELNARINLMKFAYQHLSSLTHDYYKDNDNEAIYVYPGSNVPKWLEYRTSHDYVNVDISSSTAPHSPSLGFIFCFIVPRILSDGFAFRFTISVGEDEGNNVKFYLEKPLEKIVSDHVFLVYDHRLTHFLNSRSNVEVQQRLKIKVAAVTDREIRLCACEAKRVWNQSNKSIRISKFHSENGIG

>arahy.Tifrunner.gnm2.ann1.0E6JXZ.1

MAEVAVTIVVEKLAELLVQQAAEAVSEVECPEGVREQVGKLKNELAWMQGFLKDADAKQSNERVRIWVSEIRDLAFEAEELIDTYMYKATMHTRHLDKLLRPLHMYKLARRIDRIMSKIKEVSARPEAYGVRGESREDINLTSIESLRHWRQPSPYSEEEYVIELEDDIELLLSQLLTVEPRNHVVSIVGMGGLGKTTLAKKLYNHRSVVNHFECKAWVYVSKEYRRRDVLQAILRDVDASSRDEADRLERLPEEELVNKLHSVLDEKRYMVVLDDIWGMEVWDGLKSAFPRRKMGSKILLTTRKWEVALHADASSSPHHLRTLTEDESYSLLCNKVFIPPELESLAREIVVKCEGLPLALVVIGGLLSRKHKSSVEWDQVLRNISWHLLQEQERIARILELSYNDLPSHLKSCFLYLGLFPEGLNIQTKKLLRLWVAEGFLPQEGQETPEGVAHRYLNELIGRCMIQVGAVSSLGRVKTIRIHDLLRDLSLSKGKEEYFLKIFHGNMAPSSTTSQSHSQQPTRSRRLSIHSCDDRYDFLKHGAHHSRSLLFFNREYNDIVGTIWFHWNFLQEQKLNFIYRKFKLLRVLELDGVRVVSLPSTIGDLVQLRYLGLRKTNLEGKLPPSLGNLKNLQTLDLRYCCFLKRIPNIIWKMVNLRHLLLYTPFDSPDSGHLRLDTLTNLQSLPYIDAGKWIEDGALAKMSNLRQLGIYELSGKMVNSVLSTVQGFRNLHSLTLSLQSEEDEFPMFRQLSQCIHLEKLSLIGKIRKLPDPHEFPPNLLKLTLHNSHLQKESIAKLERLPKLKMLILGKGAYNVQELSFNAEGFSQLNILRLIQLKELEEWTVEERALPRLEHMLIDGCEKLRTIPEGLKTLTSLKKIKIIGMPVEFEHRLRTNDVPEFKYVTPAIESSMDILAVGMLIHSPMLLFQV

>arahy.Tifrunner.gnm2.ann1.0MD4T6.1

MLGNTKHDVFLSFRGEDTRNSFTTHLYAALSAKRIKTYMHDTRGDDDGGEEKEAIQESNIYVIIFSQHYAYSARCLDQLTKILECKERYGRDVIPVFYKMDPSNVANQTGSYADAFVKHQQRFGDKVQRWKLALTQVAGLSPPRSKINSASPDYILVEEIVQHIWRRLKSNYSTDYQGLVGIHNHIAQIHSLLRVELEAVRIIGICGIGGIGKTTIAGALYHELSSQFSFSTFAVNVQQQIENHGMQHTQSKYISELLEEKMPADYLGLRVLTEKLKVAKVLLILDDMNNSAQIRDFIGGHGIFGLGSRIIVTSRSLQTLKDAGVDEIYETKEMNFQDSLQLFSLNAFKQKNPTENYMNLSRMMLNYAKGIPLALKVLGSMLHGSREEEWERVLQKLEKIPNLKTYDLLKLSYDGLSEEQKDMFLDIACFYREGHDVNTVKQAFDSCGFNAATGIRVLSDRGLISMLRGELMMHDLIREMGQEIVHQQCANEPGKRSRLWKHEDIYHVIRENKGTDAIRCVFLDMCKIKEVQLHPETFKMMHNLRLLQFYKSSSIQALKVSLPAFLHSLPDSLWFLCWNGFPQRSLPQHFCPKNLVTLDMRDSQLEQLWEKDQKLPNLKRLDLSGSKNLVQLPDLSHCPNIEEIFLSHCKSLVRVYSSSFLNKLNCVWLNGCTELRNLNLPSNILSKSSGLIVLNDCTNLELFSISITTKDVVLHGCSRSRSIESLFRNCLPGDMIRCMMGTRGGSLFESFSDTFDPNGGAAANLDDEPMDNIHLLNLKVLREGSPSLFPSLSELCWLDLSYCESLTSLPINLFKLKWLRKLYLRGCSNLEKLPEIEEDMENLMVLILDESGIQELPSTMQNLVGLEELSLHRCRSLVFIPSSIGRLSKLCILDLTYCEALESLPSSIFNLKLTKLDLHGCSMLKTLPEIMEPAESVAHMNLAKTAIKEIPSSLAFLVGLQTLQLNLCKDLEFLPNSIGNLNHLSKLDFSGCEKLSELPRDIGNLSSLRELSLHESSIVDLPESIAHLSSLKSLDLSDCKKLENIPGLPPFLEHFVAFDCPSVRRVSSSGFDIKLPSDSKDGIFKFHLTNSQELTQSSQSNIAGDAWQRMLDTAYRSVLFCFPGSAVPHWFPNRCKGHSVTLKQSSLNWCSDNRFIGFALCVVFGLEGMHDEECKYSVFSYRFTYECDDGIHVVPSNDQLRYYFNWKDRQRFILHDHTFIWKSYLETQTINHMLSHNAHNFSFQICKYDVGRSWPNYRPRFNIKECGISPLYTK

>arahy.Tifrunner.gnm2.ann1.0MH239.1

MAESAIAFLLQRLVSVFENEVTWFPGIQEEVVHLKGHLEVIRAFLRVADAKQESDEELKVCIKQLRDIAHDAEDLLDELELVQAYDHTNGFSVILSRFSGQIRHMKARYRIASDLKGINSRMRTILGVLAKFDTASQASNYTGKAWHDQRGDALLLENTDLVGIEEPKKQLISWLIKGCPGRKVISVTGMGGMGKTTVVKKVYDDPEVIKHFKACVWVTVSQSFKTEELLRDLVQKIFSEIRRPVPDGLESMRSDKLKLIIKDMLQRRRYLVVFDDVWHMHEWEAVKYALPDNNCGSRVMITTRKSDLASACSIQSKGKVYNLQPLKEDEVWDLFTRKTFQGKSCPSYLTSICKCILRKCEGLPLAIVAISGVLAMKDKCRIEEWDMICHSLGAEIQDNDKLGNLKTVLGLSINDLPYYLKYCFLYLSIFPEDHLIERMRLIRLWIAEGFIEAKEGKTLEDVAEDYLKELLNRNLIQVAGTTTDGRVKTLRIHDLIREIIILKSKDENFATIVKEQSVPWPERLRRLSVHNTMPNGQQQRSVSQLRSLLMFGVAEQLSLCKLFPGGFRLLAVLDFQDAPLQKFPVAIGGLYCLRYLSLRNTKVNMVPGKILGKLKNLETLDLKKTSITELPADILNLKKLRHLLVYQVKVKGYGEFHSKLGFKAPSEIGYLQSLQKLCFVEANQGCGKIIRQLAELCQLRRLGIRNLREEDGKAFCLSIERLVNLCALSVTSEGENKVIALEFLSSPPPYLQRLYLSGRLLDLPDWMPSLHNLAKLFLKWSCLEQDPLEYLQDLPNLSHLELLQAYTGDTLHFQCGKFKKLKILGLDRFVELKQVILGKDAMPCLEKLIIQRCQLLKNVPSGVELLTKLKVLELFDMPDELMKTICPQGPGKDYWKVAHIPEVFSTYWRDGAWDVYPLESFKDCSPRSGTVMRSDERSTLSKV

>arahy.Tifrunner.gnm2.ann1.0QS9SD.1

MARTVAASETKTNKNYEVFLSFRGKDTRETFTGYLYEALCREGIITFMDDEYLTEGETIRPQLLKAIEDSKVSIVVFSENYATSAWCLDELVKIFQCHKEKNQLVFPIFYKVDPSDVRHQNNSYKQAMDAHEIRYCYESQKVQKWKETLAEISNMKGFHLKQGYEFEFIQDIVNKISTKVSAKQLPIEEHVVGLQYQVARLKSILDIESNDNTFMLGILGIGGIGKTTLAKALYNSLCNQFEGACFLFNVRETSSQEKGKVCLQQMLLSKILQKGKVKLGSVDEGISTLKERLCTKRVLIVLDDVDKIEQLQALAGGCAWFGSGSRIIITTRDKYLLAVHQVKRIYDMPMLNEHDSLQLFCQNAFKTSSPPTNYEDMCNRAIRYAKGLALALKVIGSNLIDKDLKEGKSALDKYEKNPHIDIQSILRISYDSLQHNEKEIFLDIACFFNGMRLDYVKRILDGCGFYSEDGIRILIDKSLIAIEHGYLRMHDLIKDMGRDIVKQEAPKEAGERSRLWFREDVLEVLTKNTGSAKIEGIKLDLPEGVNWSDTVFKKMKKLRILIVRNTSFPSWPIYLPNQLKLLDWKGYPSKFLPPGFYPEQIAAFNLRFSLLVLKKPFQKFEHLTYMNFSCCQSITKFPDVSGAKSLRELILDGCKKLVKVDDSIGFLPNLVYLSASECTQLKSFLLQISLPSLEYLSFDLCSRLAHFPDVLGKNDKPLKISLRHTALRELSDSFAELSGLGYLDMTGCKELKYLPSSLFMLPNFVTLKIGGCSQLRELLTSSKGRLSEAEHRPKLETLDFSNACLSNEDLHVIMQSFPNLKDLNVLSNYFVSCPESIKESICLRSLDVSYCLKLQEIPELPSNVQKLNARHCNSLTADTSSMLWSQVCKEINKLQVLMPQTDIPDWFDYHDQGGIPIFWARCKFPAVALAFMFGKMNYQAVELHLFIDSEHVNVQHQHKHTLNIAENHVLLCDLRAFFNDEEWESLDARIGHDNNWKQVQVVCEPDTSLREWGVYVYKKETNMNDIQFTCPYHESSRLQSLLVITNTKTISREDDMEIESARASASSVEETSASIVQALEASYSKHGVPHVNSNSWAQTCSLCCSALLSSYRRLLCCTCRCRIDDDEPCGEARMTKAQPARLMLDDNLNCCAQTCCLCFSALLSSFRRLLCSIYGRRLDNDEPYGEVKMTKAQPGPMISRPQEESTENVDEERVPSSFSQSLQTVVENLKRLTAPREDKGWSHGEDSYNSELSDVDFEDDGEGNLDMEA

>arahy.Tifrunner.gnm2.ann1.103L73.1

MAHVSPSTSSSSFFYSYTYDVFLSFRGKDTRKGFTGSLYNALRQRGIHTFIDDEGLRVGEEITPSLLNVIDDSRIAIVVFSKDYASSSFCLKELVKILECADSKGRLVLPVFYDVDPSDVRYQNGSYGVALAKHEKRHNVEEWRKALSKAANLSGWHFKQGPDSEYEYVFINKIVEEVSNNLKRVPLHIADYPVGLQPRVQEVTKLLRIGVDDDKVKMVGIHGIGGIGKTTIACALYNSIADRFEASCFLSNVREKASKNGLVSLQESLLLELVGEDVKLGGVNHGIPIIKRRLNQKKVLLVLDDVDNLQQLRAIAGGTDWFGSGSRVIITTRDKHLLRSHIDVKVKSYGVKELNKEEALELLSWNAFKGNKVDPSYADLLNRAIRYANGLPLALTIIGSNLLGKSLDEWESALQNYEQRPNRSIQEMLTVSYEGLEDIQKEIFLDIACFFVGEKLEYVSNMLLHGRGFNPEYGIGVLIQKSLLMDKYGHAAMHDLVTEMGREIVRKESEVEPGNRSRLWFYEDIIHVLEEDMGSEKTEIMIIDLPEDEEVYWTGEEFKKMKNLKILKVTNAKFSTGPKHLPNTLRVLDWEGYPSPCLPPDFRPRKLSMMNLPNSSLKLDNPLKNFRSLSHMNFEGCETLVQLPDISGVPNLTELCLDDCTNLVEVHDSIGFLHKLRKLSVKGCTSLSTFPHSINLTSLETLSLWGCSNLRNFPEILGKMEFLEGVDLGSSAIEALPHSIQNLVGLRRLDLISCNWLSQLPSSIFLLPKLESLEAEYCKGCQILINYEGQENMGSIVSSKVKDLHFRSCNVSDELLPIYFTCFPNIEMLHLGANNFTTLPSCIKDCRFLTSVCLDNCKQLQHITALPPNIKTLSARNCISLTSHSTSMLLSQEIHEVGGTHFMFPGTNIPDWFDQHKEGASLSFWFRKKFPEIALCFVFGGLDKEPRKFEVKLHVFINGKPSFYSNEGNSFKMLTDHVFLFDLQKEVELPSYQLDQVVLENEWNHVEIHCLHSPTVLESSLDMAIVKWSGVHVYKQGNNMDDISFCNPQHRTYDLVARYPWIKEPVCTLK

>arahy.Tifrunner.gnm2.ann1.104ZDW.1

MAASSSSSSSDPGATSSSHFKYDVFLSFRGYTRLRFTDALYHALINNGIDTFRDNDKLRIGKELEGALLEAIESSRMSILIPCDEYPTSKWCLDELVKIMECSENGRKRPVLPVYYYVERSDVQYQLNEYAKAMTAHEEKGRYNHKLEAWRSALSEVGKIYGQRCDQNTPFGMAINKIVEEVIKRLPPLPLYIHRPLGCDSELEEAKSLLQIGSSHACRLMLGIHGDGDLSQFVAELYNKIRCDFASASFLSGISKKTNASGGGLEDLQKTLLSEMKEKVKNKIGSTFSGSFEIKRRLGKKGVLLILDDVDNIQQLKSLAGEIDWFGPGSRIIITTRYEDVLDEHAGVDIKKYRFDEGEFEGNGGSSTMMEENVVGLEEDFNVIINQLKEEDSARNVVSIVGMGGLGKTTLARKVYNSDEARELFPCRAWATVSKECMPTEVFKELLKCLKVPEADYENAGEEKLKDMVRKRLNGKKYLVVLDDVWEANVWDKLKGPLPDNNNGSMILVTTRNDQMANYTRSKEPHHQLHLLDEDQSWEMFRNKVFGREECPPPLELIGRSIAFESCKGLPLAIKTIAGIVAKKERSEDAWEEIKNLLPYWSVAEDKEGEEMMKILKYSYDDLSEKMKPCFLYLGVFLEDAEILVRDLIQLWMSEDFIKPIQTGRRLIEPEDIGEQYLKELVDRNLVQVVKRRSDGKGVKACQIHDLIRDLCILVSNDNPDNSNNARTFTFSRSEGSYACSVTCNHSSTCSLFVYGDVDGWSHHIPEGCPVNVLYLNGFDGLPNEKNAEDLERLKSLKFLKMDCLVLHRLFKLQSLQTIQVNCIWKPKKISVDEGLKQMRHFRCPGLGGEQLLLDERVKERMQNFQTLCYVYADSQLGLLLNNGYFPNLRTLGLLISEDECQLVDENLRSLLRLSELRKLKLVFLNVFERVPLGKIRFPSNLTKIVLACKDFNDQDMNALGRIRSLQILKLHQIICEQYVLNCGGAGSFPQLQVLIMIGVSVSSVTLEAGAMSRLRSAVFRQCLGLTLQSLPERMLSLEFDLHFIEPDSDDDDERRRRLR

>arahy.Tifrunner.gnm2.ann1.10RJVF.1

MAEALLSGFINVVLERLISREFVNLVVGKKLDRKLVDRLKTAILAAKALAADAEQKQFGNELVREWLHNLKDALYTADDLLDRVFIKAQIRSKVRTRLPHFLDLSGRKMVTKIEEVVERIVDLERCKDTLGLREIPTGSSSWRPPSTSLVKGNVFGRDGDQQALIKMLNDNNHHNLSVISIVGMGGVGKTTLAQCLYNNKDLMDGEKLSEKKFFIVLDDVWSEDVDKWNSFIVPFQHGRKGSTILLTTRKENVGPTVQNFSSYSLKGLSNDYCWSIFAYNASFPESNGSSELEGIGRNIVERCDGLPLAAETLGRLLRSEHRVEEWNKILFSDIWEFPAANCKIVPALLISYYHLPAHLKHCFVYCSLYPKDHKLDKDELILLWMAQDLLRPPKKGETLEEVGCECFDHLASRLFFKQVENDDEKYFVMHDLMHDLATFLAGDLYCRFSDELGEKEEMSILTHHLSYDDSILEKTCSSSKIKSLRTLLYINQGACTWEDCATLPCYILSKNIYLRVLSFDGLNKFPDSIYKLIQLRYLDLSWSQILVLPQSLCNLCNLQTLKLKGCTFLTILPSSLGELIHLRYLDLSGSHVETLPNSLCKLCNLQTLKLEGCPGLTMLPNGMYKLVNLRHLHIRYTPLKEMPKGMGKLKQLHILSKFVVGKQEDNGIQELGGLLYLHASLEIQNLENVIDGNEARSARIIDKKHIDELLLEWFLPSGDVMASDAQTKRDILHSLQPHNGLKELRIRGYKGTIFPDWLGHSSYQNMTSVSLQYCRNCCVLPSLGQLTSLKSLRIERFDQLKSIGKEFYKNEGHQHSSPIAPFPSLETLVFDGMSCWEEWSLPDSEAFPQLKSLQITGCPMLNGDMVNQVLMRIVSSSSDVSKVRQLEIQEQRESWENKEMTLDGDSLTISGFECVAECAFKARIIHHLTSLQEIHLSWCSSVVSLGGNCLPKSLRKLTILGCRQIELLQQQHKYDLVHLQIEFSCDSLTSLSLDAFPNLQNLEIFRCSNLESVSMSEPPHAALQRLTISQCYKFVSLPSEMNSLLPNLQSLDIRGCRNICRWPEGGLPANLKELSVGECEELVRGLSWLGNLDNLTHLFIDGSNFESIIKSYPEVGWLPRLPSLTILQISWFPNLETLECSELLHLTSLQQLHISNCYMLENMAGEKLLPSLLLLQIDGCHLLGEHCKNKHQQIWSKISHIPTIKLNGRQIF

>arahy.Tifrunner.gnm2.ann1.13SC4P.1

GAYLSSFVDAISKKLSSILEDDSVLEGNDSVLELLERLDEILCDVEHVLDDAELKQFRNDRVKKWLVDLQDALYMADDFLDELSTKAAIATPGRKGKLGLEKSAKLDTSWRIPSTSLVVSSDIVGRDEDKENIIKLLLDDTCDAESHVTVIPIVGMGGIGKTTLAQLVYNDAKIMGKFDTRVWVCVAENPNPVHVTRTIIGAIDSSPCNRDNFDSLQTDLKKKLTGKTFIVVLDDVWDEQRNMWENFLKPFHYGNNGSKILLTTRSGNVASVFAPNSLHCRLSLLSEEDCWKVFLKHSSISTGSKQYAILEPIGRKIVEKCKGLPLAVKTLGGLLRNKYNEGDWENVLDSKIWELSEDVSPALRVSYHYLPSHLKRCFVYCSLYPEDYQFDKDKLILLWMAEDLLQPKENNTLENIGCAYFDELVARSFFQPVSTNRKLFIMHDLMRDLATFFAGKFYFKLKEFGNRCMIDNKTRHLSYVTKFEDSIKLFREAYNGAVHLRTFLDFTLLRRRQSIDIESDSWLLQQQLGCLRVLSFKRSSIESLPDSIGELILVRYLNLSYAPIVTLPESICKLYNLQTLKLKNCAWLKMLPSRMEDLVNMRHLDIRGTSRLKEMPKGMSKLKYLNFLSDYIIGKHEENGIRELGTLDNLHGSFCISKLENVKNSGEVLKAKMGNKKHINTLELNWLPDGDIDDVQTERDILGKLQPHQNLKKLSIVGYRGETFPDWLGLSCYSHMTKLSLDRCMNCCKLPSLGQLPSLQHLKFSNLDGLEKIDLEFYNKSNASFQQETPFKCLETLKIVNMYSWREWHFPYEFDGFPKLRILSIKSCPVLKGGLPAHLPALEKLEIVECEELACRLPRAPKLHQLLVEGSSTSYWGVAPHNIVISGTQLANSVLEWLLLHIQLPRVIGLRIINYESAISISGDYLPASLQYLEISSCSKLTFSEHVQHKSLTKIEVKGCGSLTLFPMAGFPNLTELEISECQSMEKFHVGVPQALPNLLCLRISKCPSLVSLPSLGLAAPHLQELCIEDCRKIDCFAEECLPPSFKTLEVIECEKLASWITSNVLQSECLTHLKLVSCFDTKPFPGEGCLPASLECLILREFPNLETLDCKGLHHLTSLKTLAIADCEKLENITEKHRLASIKNISIGEECPLRRKLEKMEHPRIQFIPCKCFCSMPNVFYLLLAIMDIHNLSMMIFLILILQHVVCNERESVAFGYYVFQHTLALGLPDLRSWNSICIVHLTLFICQNRMLVSQCLFMNGATCYTNESLEPPTMSIKEFASAARKANTQGYILFMAFCADIMLYLKFPSLLSLRLQEDACRFVDMIMQAFDDKLMVLASNAWLHLAKMSQTRKSPASLVCGFL

>arahy.Tifrunner.gnm2.ann1.15H21N.1

MAGALVGGAFLSGFINVVFDRLLTTDTVNRVLGKKLGPALVERLKISLYAAEAVLDDAEYKQLGNEPVRDWLNRLRDAVYDADDFLDAVLTKAATQKEVRSVLPSFFLNRHRKMVDNMEGVVSRIEFLVSQKDILGLQKSTKDNNLSSSSSSSSWRETTCLMEGNIYGREDDQQALIETINDNSESQLSVIPIVGMSGVGKTTLARWAYSVAEGFDPKAWVCISETFDVADITKKTIEEITKTTCSLGSLNLLQNELQKILSGKKFFIVLDDVWSDDADKWKQFLTPFHFGAKSSTILLTTRNQEVASVVQTCPSCPLNELSEESCWLLFAANACFPELNGNPTLEDVGRKIVKRCKGLPLAVETVGRLLRRKDDVKEWNVVLMNDIWELKNSKIIPALLISYFQLPPYLKRCFVYCSLFPKDHEFEKNELVLLWMAEDLLRLPKRGESLEEVGSQCFEELASRLFFKPADEDFPEAYVMHDLLHDLAIFLAGDFYCRIEELGEQQEKKVLTRHLSHISRGSLGPPISKVFNSHMKSESLRTSLYIKDLLSKKSRASKLKYLRVLSFRKLDVLPDSIGELIHLRYLNLSWTNVKTLPESLCNLFNLQTLILYHCYSLTMLPNDMHRLVNLRHLDLRGTSLEEMPREISKLKHVRILDYFVVGKHKDNGIQELGGLSNLEGSFEINKLENIADVRQARSARMLEKNRIDNLSLKWSSGNKMVSNIVTLRDILDNLQPHTGLKELRIEGYKGERFPDWVGHSSYNNMTRLTLLDCENCCMLPSLGQLPSLKSLCIISFDQLKRIGDEFYKNDNDHHSSPIAPFPSLEELVFHNMTCWEEWHVPHPEAFPQLRRLHITFCPMLKGDMLNDILWRRVSSLREGNEGRCDEMVGGGDALSIRPSQSFNATTINHQPHAALQRLIISGTGEGVAAPNLEALPPDVNSLLASLHSLDIHGSSNICRLPKGGLPPNLKELSGGGCEEHVRSLSWMGNLDALTHLTIHGYGCNKNIKSYPEVGSLPHLPSLTTLKIWCFDNLETLECNELLRLTSLQQLRIACCDKLENMEGEKLPPSLLQFEVDCNEVKVPNGGDFILYNILALGEKFMDVRHFIGHVYLYIWR

>arahy.Tifrunner.gnm2.ann1.15KR3N.1

MAEALLSGFINVVVKRLISPEFINLVVGKKLDRKLVDRLRTAILAAEALVADAEQKQFGNDRVRKWLHSLRDALYTADDLLDRLFIKAAIRNKARIRRPHFLDLSARKMVTKIEDVVERIVDLERCKDTLGLREIPTGSSSWRPPSTSLVKGNVFGRDGDQQALIKMLNDNNDHNLSVISIVGMGGVGKTTLAQCLYNNMDLMDGVDLKAWICVFENFDVVETTKNVIRGISSGVCSLESFDLLQQDLKEKLSEKKFFIVLDDVWSEDADKWNSFIVPFQHGRKGSTILLTSREENVGPTVQNYSTYSLKVLSNDYCWSIFADNASFPESNGSSELEGIGRKIVERCDGLPLAAETLGRLLRSEHRVKEWNNILFSDIWEFPAANCKIVPALLISYYHLPAHLKHCFVYCSLFPKDHKLDKDELILLWMAENLLRPPKKGETLEEVGCECFDHLASRLFFKRVQYGDKYFVMHDLMHDLATFLAGDLYYRFSELGEKEEMSILTHHLSYDHSIPKKTCSSSKIESLRTLLYINHRAGFLKAHTMLPCDMLSKNKYLRVLSFGRLDIFPDPIDKLIQLRYLDLSWSNIEVLPQSLCNLCNLQTLKLEGCSKLTMLPNGMYNLVNLRHLDIRGTRLKEMPKGMGKLKQLHTLSYYIVGKSEDNGMEELGGLLNLHGILGIQKLENVVDGNEARSARIIDKKHIDELLLEWSAGDDMVSDAQNQRDILQNLQPHTDLKVLRIKGYKVKTFPDWIGHSSYNNITSVSLESCKNCCVLPSLGQLPSLKSLSIKRFGELKSIGKEFYKNEGHQHSLPITPFPSLEHLEFRNMPCWEEWHLPESKAFPWFKSLRINGCAMLQGDMVNPVLMRIVFSSSNVSKVRKLEIEDLETSDKEMRLDGDRLSISGFECLVECAFKARIHHLTSLQQIHISYCSSVVSLGSNCLPKSLQMLKIDRCRQIELLQQQQKYDLVDLQIEYSCDSLTSLSLDAFPNLENLRIIGCSNLESVSMSEPPHAALQHLSIDRCDKFMSFPQEGLATPNLTYLSVSWCSKLEALPRDMNTLLPNLESLDIRGSPNICRSPEADLPPNLKHLGVGNCEEQVRGLSWMGNLDNLTYLMIVGDGRESRIKSYPEVGSLPHLPSLITLELWFFNNLETLECNELLRLTSLQHLHIVRCDKLKNMEGEKLPPSLLLLQLDDCHSLGEDCKSKHQQIWSKISHIPTIKVDDRQIY

>arahy.Tifrunner.gnm2.ann1.19V8U8.1

VLPELPSSLRELRARGCDSLVASNVNDVVSKACYGFAESSSQDREDVLQMLIHGKEIPAWFEHQEQDNGVSVSFPLNCPAPESIALSLCFLLQAIFFQFETSFLSNYVKVQLSDKRIHIGPAFNQVHLELLSEDLSVQDIASNYRGSWILTFSLLTCALFKLKEWILVLLTLLRSSEVLFDLFHHDVRLWKAYSTMLQPADFIFAWYTCFLADKVICPSGFLHLDLLESSDGLYLKHKWPMYLIVVTSKHCLYSGRVSSSPFLPPRSWTYHVFLSFRGQDTRKGFTDHLYASLQRKGITTFRDDMNLERGEVISHELLRAIEESMFAVVVLSPNYASSPWCLDELQKIVECKNNLGLQIVPVFYGVDPCDVRHQKGTFEDAFRKQEERFGGDSEKVKRWRDALIQVASYSGWDSKNQHEATLVESIAQHVHTRLIPKLPSCIESLFGMASRVEDVTTLMCIGLSDVRFTGIWGMGGVGKTTIARAIYEAIEDQFQISCFLANIRETCETNGILQLQKILGEHIHVSRCTFSNLYDGMRIIRNSLCNKKVLIVLDDVNDVSQLENLAGNQDWFGPGSR

>arahy.Tifrunner.gnm2.ann1.1EPI1N.1

MADWTYDVFLSFRGKDIRQRFIGHLYKALCRRGFHTFIDDVEIERGEDITRSLLTAVENSMIAIPVFSENYATSSFCLDELVNIMECAKTKGQIVLPVFYDVDPSDVRNLRGSFGEAMEKHEERMKLKEDKERLEKWKMAFMQAANLSGFHFKLGAESECEFTEKIVKTVSKWIHHTCLYVSDQVVGLESQIPELNLLLDVESSDRVHMVGIHGIGGIGKTTLARAVYNSIADSFEGVCFLGNVRENSITHGLVYLQQMLLSKLVGDERDIKLGDVSEGKKVIERRLNRKKVLLIVDDVDRLEQLKAVAGDSVWFGSGSRIIVTTRNKGLLTSHGIVRTYEVEKLNDKEALDLLRWNVFKTREVDPSYSYILNRTVAFASGLPLALEVIGANLFGKSKDEWESALDQYKRSPKREIQEILKVSFDGLEEEEKKIFLDFACFFNGYRSKYVEEILRAHHGFCPKNSMRVLIDKSLVKIEDDRVMLHDLIQDMGREIVRQESEDPRGRSRIWNFEDAKRVLEQDKGSHKIEIIKLAFPKADEKLNWDGVAFIKMNNLRTIDINKGDFSDSPKHLPNSLKKFMNLRVLNFRNCQHLEQIPDLSVAPHLEELSFCWCKNLTEVHKSVGLLNKLRMLDAKGCCKLRSFPDLMLPALEQLRLSSCSSLESFPEILGKMESLTKLELECTPIKEFPPSIRYITRLERLELWHSKIVLLPSGIFLMKELKCLRIRNCDGLLLHSQEKVEEQISSVVFSNQQHFDFRNCNVSNEFLQRSVPWFVNVKELNLSSNSFTILPACIEGCTFLKVLILDYCGNLREVGGIPPNIEKFSARRCISLKSLDLTLLSFTKDCYFLKELILDGCENLEEIRGIPPSIEVLHAPSSTLLTSSSRSMFSNQDLHEDVNDKESWLPMPGTKVQEWFNYSRHGSSISFWFRNKFPAISLFVINELKKTSFEPKLTINGHEMHLFSLFSLQKDHILILTLGPKQTEFKDEVNNVISKDEWNHVELSSDHAVHGVGGGTIYESMMQIGLHVFKQFSNMEDIRFTDPFLLEEVHSLEGIGYSPTQFVQRHQNLTSLERSVEQEIVSHSLLPSPSFSDNLNWESNTIVTEHKTNTISVQAYEDNLANAVGESYQTPSIANTENVHDPIPTTPSSSDKDSGQKMYTQTCSTTLLIKFDEPLINQAPLTQDDNDVEMEAFYATLDAETHDVLSPNSNDDPATTTAPSKEAKEALKTVQDFITKNDASVLLDEENYNVMKNSLHYLSNLSSKDGISGEVETLVSEASWLFNCCSVEYIESCRNIESTASELQRVDELEAGLEGNKNKHRELRQKLDWMEKRKKELEEEMNAIKAKLCDCESEKKIVVQKKKDVFEEAKTLKAQRDEWRKKVPQLRHQQSIAKSNHAKFTGEWSKLGEKFNTIVIDHLEVVKP

>arahy.Tifrunner.gnm2.ann1.1PK53M.1

MKYKHDVFISFRGTDVRRGLLSHLKKDLHREKIDFYVDDAKLKKGDKILSLLTAIEESLILLVIFSKDYASSKWCLAELEKIIECAEKNEQIAVVPVFYKLDPSEVRHQRGDYAAAFVKHEAREDKVKVQKWRYALTKAAEFSGFHYPMDSELDESDVVDEIVEDICSKLRKFSSSESDDPAKSNGLVGIDKNIGWINSLMTNNSEQVLKIGFWGMGGIGKTTIAQLVFDKFSHQYEGCYFLENIRDELKNNQLNYLRDEVVSKLLGEENHHITGASRANSSTMRRLSKKKVILVLDNVNTPKLLRDFLEPIDLGPGSRVIVTSRNQQVFTAGVFHEIHHVKELKSEESLELFCLNAFNESQPKRGFKELSREAVAIAKGVPLALKVFGSYFHSKSSDIWKCALERFKKYPDPDGEIQSVLRFSYDELHDIEKKVFLDIAFFFKGEDKDYVISQLDAWNFHGPSGIASLQEKALITISKDNKIQMHDLLQRMGWEIVRQESATPGRRSRVNDPEEVYDILKNNKGSDSVEGILLDLSQIGEVALNVDSLKKMPNLRLIKVYSPLPIEVPMQIRYFIPQDFLSVLYKRIKMDDLTEELLEESGSLNQSCDNEDIDDVVETIKGTQVDGIKILDTIIEMHILGMPKLNMPMHVEHPMPGLPIMQDLVKDTKGISTQPLMLSKLSKLGGVHKLANYNENDGVLVLATNDLSRSMPQRNPNIGSAVKELISLGSEFHVKSTHHLFMEGSSVSKGLSFSTKEIRSINSTLNRTEALNSSVELFKNLECSDAKALQCYAETRMELLNSSTGCFISHDYSDIDDLQLETTRKDELSFLRSTNYYRLSNCRENKHIGKPKLLVLFDGLRYSEQMSILVLGGSSLQWSYLWCISNNSQFMQLPVGPQLTNCVKLSLEVSPGSLPSPLLLSIMHLPPHLKSLSVRGGRSFWHKIKLPPFIPDVRTCRRMRMWRESLPSKINHHVYMEHLSLPRNLQPIMEFPTSIQSLDIDGYIMPEGLSSSFTYLPHLESLSLDYCTDLPFTSELPSFVQSLEISNGIISKSLSSNFKYLLHLKVLPWRDCSNFRFTSEFLSSIQSLKVSNCL

>arahy.Tifrunner.gnm2.ann1.1RZ0PJ.1

MGRPKEERYWNQVTKEADRTWKCNRCGRQFSGGVSRIKAHVNRIPGKGISICSASPNDNHLQPQPQETANPMNPGEVAAEDDDHEIVDAFFGGLMQHSINIISQPINLLGGSAAVAQSNNDGFVSDEIKKLTELLHDLTLEEDDIKGELEWLKSEGKQSKTQVDDWLNELQELRNEVGDCLNLAMGAVHYFNENYPHYLGQMQQLTAQVSELKKKKPVVLSNEFVGKDFEKNVKKMWELVGDEKVLMIGIHGMGGVGKTCLATYMETQIIRKGTFNHVIWVTVSRDNSILKLQEDIARRIGAKLNGDDDERTRAAHLSSALSEKGKWVLILDDVWKFIDLEKVGIPRCRINGSKLITTSRLKHVLRQMDCPRLNIITMDLLSDSEGLELFLVKLGEDHRTPANLPYRYKIFKIAMFIARECNGLPLAISVMARTMKGIDSIHQWRHALNKLERGEMGEEMKEEVFQVLKLSYDNLMDTRLQNSFLHCALYFEILDYDNMIMMLVDSGAINGRRSLNDIFDEGHTILNELEDHSLLLPFKNMQNSVKNMACHISKESQRCIVRCGKKLIGIPHMQEWTADLELVSLDTNEIEEIPAGISPKCPRLSTLIMSNNCISSIPECFFTHMRSLAILDLSFCRYLTSLPESLSDLSCLVSLLLDACKALAKVPPLGRLQKLSRLVISDTQVEVVPGLEMLTNLTWLDLSYNEKLRLESGRVLRGLTKLQYLDLFKAALLNVEIEDVQGLTTLEYLVAGFNDCKSYDNYVASIWNTGSAPKSYLLYLGSTANSQWIFDSKYDTGPRCDDHQIVHLLDCEESPHLLPKDLTKIFIERNPRWKSLCDALSNITPSSLENIQIGRCTEMKSVLCSFGNCSFCSNLNNLQSLQLYALESLTVICKEDVAVTDTTTQPLKPNAVFSHLSRLEISSCNGIETLVTAGLLPQLQNLQTLTVEGCRSLREIFAASSSGADSDDAASTIITLPNLTSLYLCDLPMLETLCKGIIISESLPKFVIRGCPKLESRSPFEVSSSSFTSYT

>arahy.Tifrunner.gnm2.ann1.1TDX9M.1

MAWPLVSAFPRKMLKNLQNQETMEFLHQSLLTVAAVADDAQENLFATTSTLSNNMVEWLCHFQDIMYLLDELLCKFDSGKAIATTVADAKREVEFVLRRFETISNQKHLLFLTEKQDQLSSSPSSQNLRRDKEMNEMVDLLLSDRENTPVIAMVGAAWTGRDTLANLVYYHGRVKACFELRGWVDFQWDLDSESLAKITLDSFNQDFHYDESLPELIDRLHKCLQGKKILLVLNGFRNLSGWESLRSCFSDAAGKGSAIILTTSELDVAFEMLSDHILSLEDSLFDLVDEKIDPQGFPIMWTGKSLYHLVRLEIVGSWISYLPDEIGELRFLEYIDLSFSEIRALPDSIGMLSRLETLKLAFCCYLRELPSTMEDLVNLRYLDLMGTKLLDMSLKLASFNNLQTLIGFTVNMNSGENLTELATISDIQMLSITELQNIVEARNAAEAELKEKRRLEDLVLRWNQLRFAECEHALEYLEPPKQLKVLEISGCPDRRFPNWLGDASFANLKVICIYDCKNCEFLPPLGQLSSLEELYIRGCGNVRSVGNEFYGQCSLHVPFKSLKILWFVDMPSWKEWILLDDESLQFPCLRELYLIQCPQLLQDLPNCLPSLKKLEIFQCDRLVSPLPKIPHQVHDLMEQKEEQEQGCTEILATSVTEVTSESSSHHATIIMPPVANGKNDELVSDDDGGMVDFESSFEIVKIADASELCNLTSRIKSLRIEGCQFLESLPDEFLKDCSNIRELFFIDCYSLKNFSDVLHPSSLRTIYIHKCPNLDLLIPLGTHKKFAFLEYLCISSSCESLASISINLFPRLRTLYIKDCPKLELFSIAEELRDRNLKLESLEIRDCPNLISFPETGLPTPYLKSISISNCRRLKSLPNHLAYLTSLQSLLIDKCPELESFPEGGLPSSLSLLSITFSDKLAPQKEWKLDTLPSLTHFEIESGCIGMKSFPDKDFLPRNLRSLRMSKLLSLRILNGTGFQHLTALETLEINCCHGLYSLPEGLPSSLTRLCIKESPILSQKLLHRAGAEWSKIAYIPNLQIDEVKKGEIPEQKQKFGEKRFVTRSTYLEGGKGILDGESLKSQKLDQRRKRMPMVSDVSYESDWSKIAHTSNLQIGDGIKEGSIELEASSLKGESVNKDEKSFTSQQWDLSLVTKKRMPPTLKGFYDLQPDDEVVKKGRGGRGRDHDPARLDDTWTSSMEAEVQTVHGISKNPFSHITSKGKDRYLEDFTLQQLLELTENFSEDKMIGRGGFGSVYHATLQDGKEVAIKRAEISPNYEDEKEYGFVNELQIHSRLHHNNLVGLLGFCRDTNERILVYEYMNNGSLHDHLHSHQTSSVLMSWPARIKVALDAARGIEYLHHYAAPPIIHRDIKSSNILLDSKWTGKLCDFGISLKGPEDEDTHLSVEVAGTPGYLDPEYYITQRLTTKSDVYSFGVVLLEILTGRKVIHRADDGERIHLASFAVPYIDQDEIFKVLDPRMPPPKAAEEYVGYLAAECVRAAPRDRPTMAEVVIHLERALAACLAVPAI

>arahy.Tifrunner.gnm2.ann1.1U71WU.1

MADALLGIVVENLHTFLRDQLATFYGVQSQIQELSSNLTASHAVKDWLNKLSDAAHVLDDMLDECSIKFNALQIPHGNCFSCFNPEMILFRFDIGKRMKAIRDRFLQIDEERRRFELQPGVVERLQEDEEWHQTSSLITEQRIYGRHQDIETIVELLSTGAGDGDGEDLAVYPIVGVGGLGKTTLARWVFNDERVIKHFDFRIWVCVSNDFNMMRILKSIVESATKKNPDLFTLEAMQKRVQEELLGKRCLIVLDDVWNEDQDKWEKLKYTLQCGSGTKGASILVTTRLESVASIMGTCPFHHLLPLSEDENWLLFKHYAFGQEREERAELVTIGKEIVKKCAGSPLASKALGSLLRFRNDVKQWLSVKISKLWDIPEGNAIMGALRISYFNLDLSIRRCFSFCAIYPQDFQIVKEQLIHLWMANGLIKARGNMEIEEVGNEVWDELCQKSFFQDVRTDSLGTITFKMHDLFLELAQSIMGEECVVSKSANLTSLTSRVHHVSCLDFYETLNLNQGALKKAESLRTFINLYPTLSNSRVLPTISSLRALTTSSSKLSALKNLTHLRYLTLYNSSITTLPKSVSRLQKLQILKLEDCDYLSCLPKQLTQLQDLRHLIVKNCESLVALPPKIGNLKRLRTLSTFIVHPKEGFTLAELRDLQLGGKLHIKGLEHVPNECDAKAANLSSKKDLNCLYLSWGSNANLQCNDDEKVLEALEAHSNIKSFGMKGYNGTKMPSWMNNISLLSRLTNVILYDCNNCEQLPALGKLPHLTILFVCGMRDLKYIDDDAYEGAEEKAFKSLKKLTLFQLPNLERVLRDERAEMLPLLSELSISCVPKLKLPHLPSVSHLQIQGLKSVFDYNGNKSIASFPEPIVQNMHHIKTLRIEYFYEKVLPDELSSLNVLQELVLFGCDELESLSENVLQGLSSLRILKIMCCDRLNNLTEGMRHLMHLERLEIWACPELVALPSSMNQLTSLLDVTIFGEDNNPIIPEGLQYIPSLQILTLYNVDSLPEWLGHMTSLQQLHIGLCPELRSLPSGFRHLTNLQKLYINECPKLAKQCKREAGEDWQNIAHIPHFELVAVQEETFCADALLGIVIGNLNTCVKNEIAALSGVDSQIQELSDNLEAIRALFQAMP

>arahy.Tifrunner.gnm2.ann1.1X0C2C.1

MLELTSRRGPIAAQSLELPVPQSAPAKIMVSCPAAFLCGTMFCSRLSPLVVPYDGKSCIFGGHYGIAKWVEIYSLKSGLSLPEIRIKVSRNARKGRKFGVIQHHLALAAISVLVSFIMGHQGHVLTTKPEHLRRKLETKVEQVQEQFDFESRESTFNGIFEALQDDSISSVAVYGMGGVGKTTLARHVGKRVKEMNMFDLVLPVHVTTAENVKRIQGDIAAGLGLRLGEESDLAERQRQLSLRLRNEEHVLIILDDLWDKLDLQKIGILNNHCKVLLTTRSQQVAYLMGCQRSFHLFLLTLEENWDLFKRCAGIHDDRFEPDLLAIAREVSAHCEGLPLTISILGSALRARTVDLWKQVLKDLMSSRRKSLNWEDDKDHEILIEDLFQQVIRLRLCSKTDVIATITSLKDSSLLMPSITSKDHVLMHDLVRYAARKIIFKEKPTNRDNISEYMNALYNEEHFQWQEALHRLLCRLFGATEYPESSSSTSSELQVAQSYSSTIPRPSGKMVDKSFLPLLKEACTKNPNLIESQRKHSEMLRQSAFDSLGRLLFLLHNVRIRDWINHKQELQMYWEQAKLMKFDLEWSSPAMERVLSSADLARIEALREEEKYWNEEASKLREKLKIVEDKAAVIRTEIVLTESKLDGFAIGYGSEAGVVSKNKHVKINV

>arahy.Tifrunner.gnm2.ann1.1ZZE7Q.1

MRNELTCAVLSSSSQFSRSNGNAPFLPIKCQRFYPHFNHNTSHQKNPNTKVQVIEENKKKWTKACFHRRTCDCWKLKRRDFPAAPIHLSSEVAFSGVLMDFVSSFAASISRDLVCGAVDELRYPCCFHDLVEGLEHEDKMLVDTKKRVQEHVHHQKRQLKKTDELMHKWLNQANNLSNDVDNLLTETRTNKIYCFGKCPNLFWRYRLGKKLVKNKGDVKKCIGEGSKYMQFERLASLPGLRYFSPERCLKFESTQSAYDQLMQALNDDTVNMVGMYGMPGCGKTTLAMEVGRKAKEEGLFGEVVFVPVSSVVEIQRIQEKIASWLQFDFPQKEEIQRAQCLDKFIGENHENVLLILDDVWHRLDFEAIGIPSFKNHRGCKVLITTRSEAVCSLNDCQKKIRLKTIELEEAWKLFQTQAQITEDTSNALKKLAQEISDKCEGLLVAIVAIANTLKGKGEADWKVASDNLKRCKPVIIEGGLQNPYKILRVSYDNLDPVEQSLFLLCSVFPEDFEIPVEDLIRIAIGVGLVEEVVTFEGARNKVIVAKDKLLSFSLLLDAVGVNCVKMHDLVRDVARWIARNDGKAIECVMENNANLVDSTPIRYLWCKQFPVELDCSSIEFLCIKTDTEVSEGIFKGIASLRVLILCWEGHQRREFSTMSLKSLTDLHSLFLSGWELFDISFVGDMKKLESLTLHNCSLPELIDVLVTQVPNLRLLDLSGCEMEGNPFEVTGKHPRIEELYINDDRPEWDLEDEDPPEFFSKFSVPQPLERYQIRLGKYFSKYQQKCLSHGRTLFLSCFDTSNVAVRNLATKAEILYIANIRGDVKSIIPGIFQIEGGGGGMNHGWIELLIKKSENIVHLVDTGKHLNEVGSLCPSCAS

>arahy.Tifrunner.gnm2.ann1.208V23.1

MIVLKLDHKYTSELRSPFIYDQNYACIESVLTHSRGIKTIGIWGMGGIGKTTIAAAIFQEFSPKYQGSCFLANVREESSRHGLNYIFNRLLSELLQEHVHITTPKIVSSAILRRLRRKKVFIVLDDVNTSELLENLLGVGQDYLGLGSKVIVTTRDKHVLLSRSVDHIHEVTEMNDGNSLKLFSLNAFNRIHPPENEYWELSKRALAYARGNPLALKVLGSFLHSKSEKEWDNALTKLKRIPNADIQKVLRLSFNELDDTEKDIFLDIACFFKGQEKEKVAMILNECGFYADIGIRNLLDKALISIATNQSIQMHDLIQEMGHKIVCEESLKNPGKRSRVWQPDEVCDILKNDKGSTTIETIYLDLTQQTEICISSNAFRKMPNLRLLAFASSNGYGQKRVDYTLSLPTNLELPNNLRYIQWDGCPLKSLSTTSWPSKLVELSMPYSDVEKLWDGPQNFPSLEKIYLNGSKQMIECPDFSGAPNLNSIWLSACESLIQVHPSIFSLPKLDYLAVHGCKKLKSLSSNNCSSSLRAVVAYDCPNLEEFSIPISKNQSNIHVHLRSTSLKQLPSSIVHLQDLTNFSFPISDLLMDLPEKYTNQIMLSDPISHKSDPVATLRTILPSPSFRYLKQLKFDGCQSLTELPDSISLLSSLLFIILHNTNIMTFPESIKTLPRLKIVVICHCERLQLLPALPPSVHDFKAWDCKSLRTVSSSTEQLQRQHGTTFMFLNCVKLDEESYFTILKDAIIRIEIRAKAQQLSPRLEENRNEECTIVDDGDGYISEHNPNVGKVCYFLPVSGRKLDDLFHHCSSQNSISIQLPLNSKFFGFIFYLVVPPIQPCSMGEVFEIRFGFECYLETSWGQRTHIASSSSIEWHCDFRPGLEMNVLSDHVLLWYDSQCCKQIMEIIGGRNVIDDDKNANLAVTFFACLPNKEEVAIKACGIRWIYTNMEKESRGCRFKRSREVFESEAIASPNEGNGVEFNDEGIELVLPAKKFKQNVLEASSILEVESIENL

>arahy.Tifrunner.gnm2.ann1.232QQF.1

MAHSVACSSLVSPKRYDVFISFRGEDIRTGFTSHLHKALTNKGIETFIDYQLERGDGIWDSLCKAIENSYISVVVFSRHYASSTWCLKELVKIMECRKHLGLLVIPVFYDTDPSQVRYQRETYQEAFSEQERNLLNDESEERRWRAALAEATDLSGFDSQSRDESLLIKDIVKDVLQKLLEFGYSNTTKRLVGIEETRKHVELLLKNARRIGIWGIGGIGKTTIAKVVFTILTPHYDSVCFLENITEESKYGELAYLRGKLLEQLKQINPIGKVVGTKLTTRSLKKALIVLDGVDTLEQLEYLCEESKVLDDLPEDSRVIITTRNRCLLTNKVDEIYEVKALSPEESLKLFSLRAFREIHPKEGYEELSKRAVEYAGGIPFALNALGSYLSSSRSLDKDFWESTMRKLENSPNVEIQEVLRGKSSCGEYLSEGLTQFRKELRCHGNVEELWQGKRILTI

>arahy.Tifrunner.gnm2.ann1.256JRY.1

MALPTPPLPDWLVQWATKLLKQELNYLLYYERNIKDLENQVNKLKLERQKLGDRVAEDEDRHGREIYDDVSKWLDGADTIIDAYEKFEKEEEEAHARCLAGFPPNLSARYFLSKKSIEIKGKAESQLQNAKFDIISRSRGPPSVALVLSNVDYQSLPSRVTAMEDITNVLKDTSARMIGVHGPSGVGKTTLVMEAVNRVQNDQEKPKLFDVVIMANVTKSPNIRKIQGQIADMLWMKLDEESEEGRASRIRERLKKEKESTLIILDDFYGKVDLNILGIPWQNGDGNQKNPKGKKSLGSSTANEETKQQALADGVMMNAQKSSDASSSSASTSIIALTTEERYKGCKVLLISEVRQVLNQMDVRPKLIVPLELLNEKDARILFNKIAGIGDKNTEFGELPAQIVEKCDGLPMSLVTTAKALKGRSRLVWQDTYQKFETQTMTGTPEHSTRVIYNLLENEELRITFLLCACMDNDALVSDLVRACIGLGFLRGIYTVRDTRTRVQVMLMKLRESGLLSDSYSSDRYTMQNLVRNAALSIAFKERHMFMLNKVRVDEWPDDDEVRRYVAISLRHCDIIDAITKRMTCARLKILEVINNDPQLKLPTKFFEQMKELKVLILTGINLSPSDSSVGCLTKLRMLCLEHCTLSSSKEELSLSEELSIIKNLENLRILSFSGSNIDCLPVELGDLSKLQTLDISNCPKLRVIPPDVISRLTSLEELYMRKTQIQWPKVINGAENDERKNASLLELGELNQLTNLDIQIQSVDQLPENLFFDKLSSYKIVIGSSNRYYLEGDFKMPEKHELSRFLAIHQKGGIHIHSHKGIKMLFERVEYLLLGKLNGVQDLFYELNLKGFPHLKYLAIQNNHDIQFLIYPKDRQEHHEKAFEKLETLELNKVTQIEGLCFSSCPLSESSFVNLKVIKINFCENLKYLFSPSLFKHLEALETIQVFDCDSLKEIVPVERPDEGEILKLLKLHTLTLQSLKDFIGFYPISTTKSTKILFHEKVEVKELERLELRWIQIDQIWNDQSCKFENLIHLDVSGCHYLKYLLPFSVAKNLKKLQSLYVSECYQMENIFPDGPVKVAKDATFPNLKNIKLSRMGSLRKIWNLNVAVEKLDTLVIEKCNQLVSVFTHDMEGIFQGLSSLTVTDCKSMKTIFDLTADQNWFARHYETLLRDVHLESLRELEEIVRCEQDQEGTLQLKSLQNITVHGCGKLQNIFPFSIAKHQLENLKCLVVSNCSELKEIVAVQNGTSDNGGSSTSNPVPLVFPELTSIKFSKLPNFKNFCPGSELQCEKLDEVSIELCGKLELFRESQLFHETFPSSQAGTSDQGTPLFHEKVMNKLRSMHIELQHTSSSTRYRRDKLEVLRLSRLKDTEILFRFLHSNPNLKSLWLDNCLFDKLVESQEKSSSDGNENIGVVPKLKTLRLTNLSCLKEIGFEHDAILQRIESLLLENCPKLDTIVPSKEGIRFNFLTNLEVVDCRCMKYVMPLSTAKSLGQLVTMKVTNCESLKEIIVSDDQIVDKDKEVNEQSRTNNKIVFKQMKALELVSLKNLESFCSSEICSLEFPLLEKFVVSACPTMEKFSEKEIKTMPTIMQKVYVVRDEEKRLCWDGDLRATVKHIYDKQKYYEGMDKISVSEHSVLEEGWKTEKALDKGWFYSLKTLTLERCKFESFAIPSFVLRCLKSLKELEVRNCKNITCIFEMNDIKGTFQLEKLTLEELPNVTHVWPQQDKQNDSRFRNLQQVFVKSCRKLKALFPVAIATNLKMLEQLEVHFCDELLEIVEKDRVGGGETKKFVFLYLTRLRLYNLPQLEHFYDGMFTLECPELNYLYPFNCIKFELFQTPQENSPPITRPGLFSNIKGISKVKTLSMKSKDTSVLKSWLQQSEDLELKYLRGLMLTFDDDVNNEYSTLQCEILGRRTPKLERMGIMNSTSLRKILFPSQNHKFLEHLDYMGLESLLELSSIGGLEYLSKLQKFGVFQCPLLRTIEQYPSSLKKLNVAGCHGLQCLFTSSAAKSLKHLKELYVYDCKSLKDIVRKEQGDETATEEIIFQQLKSISLQYLECLECFYQGNAALKLPSLAQAKIWKCPKMIIFSQQLRQEDPSEKVSVSFHSGADKSEVQLTHYHQLNLAVGAQFLNQTRLFLNDYPEMQGKWVGASGIPVEWSFNYLKYLEVEGCDFLTNAVLPSHLLPLLRNLENLTVKKCKLVAAIFDVKDTPPEHDDPNKIVMIPLKKIILKELPTLTHVWNNDSKASLSFPSLEKVIVEECKSIKSLFPASVPRDNLQHLHVRNCGELEEIVTIPQDANNKEISMLFPKLTWLVLWALPELRCICSGMHSLLDQSVVLTRLYVFGCPRLTVFPADIQNSDPRGEDCFANDDDKHSFVSSAQKVITSNFVELVLSKEDVRMIEKGLLHVDLQNLNYLGLDSFNDNESDEFPDVFFSKVSLPKLTEIQLVDCAFKDIFRPKGPDIDYSKILSQLKYLEINKLHNLYSMGFEHPWMAPLLEMLETLNVSECNLLKNLAASSAVSFFYLTRLKVENCAGLKYLFTSSTAKSLGALRELSITKCELLEAVVAHEEGDKPEDMILLSNLGTLSLNELPQLESFYTGNSTLYFPRLYSSLLCTITKCNKMKTFSHGDVLPKFMQGKIDEDPWHGDLNAAVQKQFQKASMITTTAPWTYMDLLPTERSLLLLSFITLAQSANSTIKKKQRRTLELPVSTCPPEYNENVTG

>arahy.Tifrunner.gnm2.ann1.28RSD0.1

MGNNGDTVIMAGVKVLKNANSHKVVKRKMRSRASKISVAVQGVIEDAEKNLKVRRVKSWFQGIKDLCYELIGVSEEFELLQPPKKSHFVGLRLLQRLKKGNREEASSIIRKFEALIEEGSKLHLSSTVPNEAEQLGSSSDPAPNEAEPEPLDLHSPAVPSNIIDEELMIGRDAEIQDLIRRLTKEYHRCICLVREEVGMGTTALARHVYNSTQVKSKFHFMAWVTVSQQFNVKRIVKSMLEFAPETPEKYAGNEFELLKLELHKFIKDRELLLVLEDVSHLDSNHLSDLMNVLRSSSSHSILIITRKKEVAQVAESTHTVVMTRLSPEACWSIIKHHAFGDDQDESSLEGSLGEVGRKIAEKCGGKPAVAKSFGVKLRGRSLGEWQQALMSGKLPEYDLTSCATLSENLYSRMSPALRQCLLYCSIFPTNHSIQVDELVKLWMAQGFIASHEEDKLEIQGGKYVKQLRDRSAFQESRDSGREGEGALKCKLKKEMHEFIQDLARNECRIMLPGEESGDNDAVTEPARYRHFTRHCTLYLEDQTSFLDSIDNGGKLKADKLHTLMVLSKFSDMDPTNLAKRLPRMKRIRALDLSDCPIEKLPSEATELLHLRYLNLSFNHKLKELPDAISNLLNLQTLNLNGCKSLQKLPESIGKLIKLRHLEILWTTSLSYLPKGIASLTLLRTLNRFFGSSGGASRGIACSLGDLENLNNIAGCLTIDGLGGETEISEATRADLKNKENLLGLELWFSIVGSKANDQVLLDSLEAPPQLQSLGIFDYGGSSFPNWMMELNKLTQLKLHRCSECNVLPTLGKLPFLESLEITNMPNVKMVGVEFLGIGLNHEDAVNEGSSPDAVAFPRLRKLHFKKLDEWKGWTGINVNGGDKKIMPQLSSLSVVNCKKLGSLPDYIKKKENLKPVIEGCPLLPEN

>arahy.Tifrunner.gnm2.ann1.2CTJ9J.1

MFALVVLSPNYASSRWCLDELQNIVECRKKFNQVVFPIFYGVEPSDVRYQRGTFEEAFRKHEERFKEEKGKVQRWRDALEEVASYSGWDSKDNHEAALIERIVDHIQKLLIPKLPSWVGNLVGVESRMKKLNSLIGMQLDDVRFIGIWGMGGIGKTTTARLIYESIKEQFNFSCFLANIREVSATNGIVDIQRELLSHLSVRSNYFHNLFDGVKIIANSLHNKKVLLVLDDISERSQLENLAGKQEWFGPGSRIIITTRNKHLLMTHGVHQTCELEGLVQEEALHLFCLKAFKQDQPKSKYQNLCNEVVEYTRGLPLALEVLGSHLCGRTPEAWHSALKQLRSSPHPEIQNSLKISFESLSSTEREMFLDIACFFKGMDKDEVVEVLENCGHFPQIGIEILIEKSLVTLGRVNTLEMHDLLEEMGKNIVFQESPNDPGKRSRLWSHDDISRVLTQNKGTEAIQAIVHYAQPYEARWSSEAFSKTSNIRLLKIRNACRLSHGLDCLPYALRVLDWLGCPLKTLPLTDQLDVVDINLSWSKIEQLWHGAKILHKLKCINLSFSSELNQTPDFVEVPNLESLVLQGCTSLTEIHSSVMHLKKLVQLNLKGCKRLKALPGKMEMSSLKVLNLSGCSNMNTIPDFGNCMGHLEELRLDGTAVTELPSSLGCLVGLVLLHLQNCRYLVCLPDTIHKLKSLKVLNVSNCSKLRSLPECLQEMNNLEELDASNTAIEVFLNCFRVASAVCRPSLLIRLDLSYCKLLAESVPDGFCGLSLLRDLDLSGNNFVNLPSDISKHSTLEYLCLNSCKKLQSLPELPLSIKRVDASNCASLVTCKFHPSSKCSIIASPVQWHLPRERKSLLKGICFPRTRFDMVITGNEIPSWFAPQKSSSFAEIPFPHPSPPTEWLGFALCFLLVADRPLRYYHTGITCYTAKQTSANEIMVPDKDDWRAETHVISRKVPDMEPKLPHLYILFLSIGEYLERMHTDTSIGFGLRTNSRGSPLRIVQAGCRVVCMENIQDILGNHSHSVGPNKKQKSNNNDGPSP

>arahy.Tifrunner.gnm2.ann1.2IE6SV.1

MIQENAVDYGSFLKMSDSSCIENSYHYLPSHLKRCFVYCSLYPKDYEFNRGELILLWMAEDLLQQPKSGSILEVGYKYFNDLAARSFFQPLKNAYKNSFVMPYLMHDIARFYGEKFFVRTFEVENVGKHDAKTRHLSYDLGDNDSVSKISEACDSLRHARTLLKIKKYREHKEGIDTCHLLAQLKHLRVLSFASFKIDILPDSIGELIHLRYLNLSNTLVVSLPESLNSLYNLQTLKLANCKELKTLPSNMQNLVKLRHLDISHTYLVDMPKKMSNLKDLQFLSDYTAGKHEENGIGELGELANLHGSLSLAKLENVENGGEASSARMDEKIHQNALNLEWSRSLFKDREFWNSGSERDVLDKLRPHKDLKVLFIKGYRGSMFPDWVGHSSYYNITKLELRRCRNCMMLPSLGQLPALTRSEISHCDMVNMIGGEFYKGDATHHPKTPFRSLKYLSFSDMSYWGEWESYECDDAPFPQLEELWIHKCPILRGDLPTFLPSLKSLHIVRCEELGYYLPRAPILRELTIDGKQKARMRDLPLSMLERLLVNGEQQVEYVIEAMTHTQPTSLIHLQISECSSAISFPGDSLPPSLEELSIEDCKNVEFPMQHQQHQSLRRTFNDSSGTYQFTTVRLCNSILLLLLHALSYTQME

>arahy.Tifrunner.gnm2.ann1.2IZ0XP.1

MFSQNQDPEFTFNPHRTIMYDVYISFEPHYPSHPFISLLSGALKRAGVHLFLDNYMKPKSKDLILSSVIKGSRVSIVVFTTDFACTTWSLKELEKIMEYRSSRGQQVVPVFYEVDPKEVFNQSGHFGEALLATLARTSTNQAKVVSYMTALKEAAAISPRFLTNFRDRSKTIDSIVGHVTSLLDSTALFVAEHPVGVNSHVQDLIQLLDNKKSDSVFIMAIWGMGGIGKTTVAKVLYNQISHNFDVRKFVPDIDERMEDFPRRWPLGIIEEELLLFLKEQVATKACNFDSTSVIWWEGIRCVKVLLVLDNVRSEKELEVFPVTCECFGPGSIIIITTRTKHDQFNEIGINHVYRLKEMDYNECVELFSQSAFKKATPERNYADLINRALEYSDGLPLALVAVGSVLFEKGRVEWESVLERLKRFPLQDVWQVLRKSIDSLGYDMKQMFLVLAYLSHFFIGMDQNDVTQILQKAGCDVVVALKAIKGLEDHSLLSFEKDRFRMHRLIQDIGREMYLKESSIKPQQRPYDVFLSFRGKDTRSNFMSHLHASLENASIYVFKDDDEEARGENISLSLLKAIGESRISIIILSPNYADSKWCLQELEDIMRCYNNQTQKVLPVFHHVDPSEVRNQAGRFGEAFEEFIKKNPQNKVKEQDWRKALRDVGSTAGFVVQNWRNESEDIKKIVEHVTHMLDMNELFIANHPVGVESRVKELIELLKSHQSEDPLLLGIWGMGGIGKTTIAKAVYNKICRQFDGRCFLLNIREVWDQDNGGLHLQQQLLSSVYKTTKIKIQNIESGKSILEKRLGQKRILVVLDDVDKLEQLNALAASYKWFCPRSIIIITTRDERLLSWLEIDKRYKMEGLNEEESIELFSWHAFKEPRPRKKFATLCGEVISYCGNLPLALEVIGSHLFERGIKEWKSVLNKLKSIPNKEVQKKLKISFDGLSDDKDREIFLDIAFFFIGMDKSDVTDILNGCGHSAGIGISILLERCLVTVDTKNKLRMHGLLRDMGREIIRESSPTTPEERSRLWHSEDVLDVLSKELGTKATEGIALKLSRMNPICLKTKAFKYMRRLRLLQLAGVQLKGNFKHLSTDLRWLCWHGCPSRYTEVNFDQRNLVAIDLKYSYLQHVWKKGQMMKKLKILNLSHSEHLTHTPDFSYLPNLKKLILKDCPRLCAISHTIGHLKRILLINLKDCTSLRVLPKSIYKLKSLKTLILSGCTKIDKLEDDLEQMKSLTTLMADNTAITKVPYALPRLNNIVYISLCGFEGLCRNVFPSIIWSWTSPTNNLSPQMHTNLDLSNLVSIMVSNSSSSHAGLSSIIKELPKVQNLQLECGSQLNISGNVNLVSSNDTNFKELEAPSRTSHVSNMNTSALGGCCSEGYISRSENSLNIILIQMGMNCLITKALRQSISQKLTSNLAGDFLLPGNNNPDWLTFSSEGSSVSFEVPKVNGRSLKAVILCIVYSSSSNIIASEGISLKNMMIINYTKATTHVYEGDTLSSLKGEDWQSVLSNLEASDKVQVVAVVLGYGFRVKETTVYLIYDDPIDQTMKQQDDGNDMVADENVTVHGGDEKEEFKLLRKRKFQYYDDTSSEDDRVGDV

>arahy.Tifrunner.gnm2.ann1.2IZ2Y4.1

MAAKLDGGAYLTSFVDAILEKLSLILEEDDSFLERNILLGRLEKSLYEAGPVLDDAEQRQFTNKKVKKWLLDLQDALYKADDLVDELFTKAAIPTTLRDPGISSSCSSLVDSYIEGSGGMEKIVGTLESVVAKKNRHRLKECAKVDMSSWRTPSTSLVLSSDIFGRDEDKEKIIKLLLDDTRHAESPVTVIPIVGMAGIGKTTLAQLVYNDDQVQQKFNVKAWVCVGEVFDVLKLTKTVVEKATSGSCNMNDLDSVQQRLRTQVTGRRFLVVLDDMWTNHYDDWKTFLRAFQCGSQGGKILVTTRIDAVASMVKTIPVHNLSLLDDEQCWSVFANHAFFPTESRDRLALEKVGRKIVEKCKGLPLAAQSLGGLLRSKDNVSDWEDVLVSEIWEFSEDECGILPALRISYYYLPSHLKRCFVYCSLYPKDYEFNRGELILLWMAEDLLQQPKSGSILEDVGYKYFNDLAARSFFQPSKNGYEDSFVMHDLMHDLATFYGEKFFVRISEHENVAQHDTKARHLSYDLDDNNSVRKMLEACKSLSHVRTLFPIKAYLYREGIDTCRILAQLKRLRVLSFTSFKIDILPESIGELIHLRYLNLSNTLVVTLPKSLNNLYNLQTLKLRNCKKLKKLPSNMQNLVNLRHLDIVGTDLEEMPKKMSKLKDLQLLCKYMVGKQEENGVGELGELTHLRGLLSIEKLENVNSSVEASNARMDEKIHLSTLHLMWSSDEDSDLVDSQIEKDVLDKLRPHKDLKRLSLWGYRGTMFPDWVGQSYYHNMTWLELSGCRNCWVVPSLGQLPSLERLLISGFKKVKKIGGSFYKGDGTHQHQETPFRSLKYLTFYQMSCWEEWKSYECDDDDDAPFPKLETLVIKYCPKLRGDLPTFLPSLKLLHISGCEELGCYLPRAPIIRELRIFGNQEARMRDLPLSLQRLRIEGKQLVDSLFEAMAMTHTQPNSLTVLSISNCSSVVSFAGDSLPPSLKELRIYDCKNVEFPMQHQQHDSLTRTYIDSSGTCQFIAFGCCASNLVRMNDLKSFLCTNYLLVEYMDKELCIYLNFVVSGRNFKLEWYNPALHYSCDYFLLLLHHPLTHPNLRPFITFLGVALACLLVALVGLQETNRSKLGLSLIRRPKESGQE

>arahy.Tifrunner.gnm2.ann1.2J15ZE.1

MAAKLYGGAYLSPFVDAVLDNLSSILEDDSVLNGNDSALELLERLQNCLCDVGPVLDHAELKQFTDKKVKKWLADLQDALYMADDLLDELSTKAAIAATRRDPGNSSSWSRLVDSYIEDNGAWKKRKRYLGLEKSAKVDMSWRIPSTSLVEPSEICGRKEDKEAILKLLLDDDDAADGDLSVIPIVGMGGIGKTTLAQMVYYDDKVKENFDFRGLVCVSAEFDVVKVTKTIIEAITSNSCNLTDLNLLQLDLKEKLSRQKFFIVLDDVWNENYEDWTKLLKPFQKGVKGSKILITTRSKKVAYMVQTVLPHELTPLSNEDCWLVFSKHARLSTVSVENPTLERIGRDMVKRCDGLPLAAQALGGLLRGNSDVKYWNQLMKSEIWELSDVEINVVPALRISY

>arahy.Tifrunner.gnm2.ann1.2JZ6N1.1

MAESPVSFLLDKLTTLLQEEVNLQRGVSDDIRHIKGELERHKAILRVADALEDKDHELQEWIKRVREIAYEMEDAIDEFNVRLVDQHMHGSNSSLTHKIVFTWKTLKARRQIGSHIQEIKSRLDVISMERPSMYGIGSRSSQRLSSRLDSQGDALLLEEADLVGIDTPKKQLSDLLFKDEPNRDVIAIYGMGGLGKTTLAKQVYDDPKVKKRFRIHAWVIVSQSFKLEELLRDLVQQLYNVIGKPAPEAVGQMRSDKLKEVIKNLLQRSRYLIVLDDVWHVNVWDSVKYALPNNSRGSRVMLTTRKRNVAMSSCAEFGKVYNLEFLSEHEAWSLFCRKTFQGNSCPSHLEQVCWNILKLCGGLPLAIVAISGASATRDKTNIEEWQMVCRSFGAEMEGNDKLEDMKKVLSLSFNELPYYLKSCLLYLSIFPEFHAIEHMRLIRLWIAEGFVVGEDGKTLEEVADSYLKELLNRSLLQVVQKTSDGRMKTCRMHDLIREIVTLKSKNQNFATIAKEPDITWPDKVRRLSVINTMNNIQQNKTFQLRSLLMFALSDANHDFSLHAVCSTGYRLLRVLDLQDSPLQVFPAQVVNLYLLKFLSLKNTKVKSIPASIKKLQHLETLDLKHSNVTELPVEIVELQRLRHLLVYRYEIESYAYFHSKYGFKVSAPIGKMQSLQKLCFIEVDQGSKALMIELGKLTQLRRLGIRKMRREDGAALCFSIEKMINLRSLSITAINEDEIIDIHCISKPPQYLRQLYLSGRLEKFPQWIQSLKNLAKVHLKWSRLKEDPLVYLQDLPNLRHLEFLQVYVGDKLHFRADKFQNLKVLGLDEIDGLKSMIMEEGAMPGLKKLIIQRCGALTQVPLGIEHLSKLKSIEFFDMPEELISALRPNGGKDNWRVQHVPVVYSTYWRDGGWDVYSLDTFGERETDSSAVMRSLELPTLWKV

>arahy.Tifrunner.gnm2.ann1.2ZE46Y.3

MFDAHCFIENVSKSYREGSAISVQRQILRRTLEEQDLDKYSPSEIAGIIRNRLSSRKLLIVLDNVDEREQLDQLAINTKLLGRGSRIIITSRDKHILESYGIDAIHNVSLLNSQDASELFFRKAFKSDHPSSSTCMELTPIILEYAQGLPLAIKVVGSFLDKRNASQWRAYLERLKKYPDKKLTDVLQVILEERSLITIKNQEIHMHDMLQELGKKLVWERYPEEPALWSRLWRCSDFENALMSAETGTNHVKAIVLDQREEMPECSQLRIRGLSKLRDLKLLILYHKNFLGSLDFLSPNLQYLAWHGYPFPSLPSFQPYSRLVELNLPYSNVKRLWEGNKNIPHLEKVDLSYSVDLIETPNFEWNAKLKQLDFTGCTNLIHIHPSIGLLNQLAYLSLQNCSSLSNLNLGDDCNLSSLRVLCLSGCTNLNKSPDFTGLSNLEYLDLENCTSLSTVHESIGALVMLKFLSLRGCIIISMPKEVNNMISLQTLDLSRCLRLRYPLGQIYSSYLESLISLDLGTCHFIDNIPDSIGVLTCLERLNLEECQCSHLPDTIKKLSRLAYLNLADCSYLERLPELPFDSAPSGGRYFQTVSASRNHRSGLYVTTYNKVMKKTFNYMPAALSWFARLVEVSILHAFLFAFSLIYLNFLCFMWKQQSCHFRCGFDIIIPENESSGIPGWYFNHQFQGGSTVRVADYADADDNWLGFSFCVTFEHVKNDCDSSELLRHFYLSFESEHTEEYFDMPSSVETDRDIDGTHAWIIYISRVHCHFVKTGARIRFKSCKGMKLHKWGLRMVFKQDIEKLKRRLQRVDNKKQSPISDSSRLVVTECVDEGGNSSSQSKIMLPYNWLVTDEEENEKMEAKAKEDNLSNLGFI

>arahy.Tifrunner.gnm2.ann1.332RDU.2

MAEIFISIAAKISEYAVDLAIRQGQYLFRAGRFIKNLEKEKQKLISTLGSVQKRVEATDKTEQVKDSVLKWINEAEKLVEEVENLETEIETNGSCFKGQCSIGKRYNLCKKMQQKIESLINLNKNGQFDTISFPAPIPGSEYLYPGNIVYFKSTQKASDQILEALQDDSINLIGLYGMGGSGKTILVKAVGNKAKTMNLFDRVVLATVSETPDIKEIQKEIAELMGLKFSEENKASRAIRISLGLQSKERILVILDDVWAKLKLEDIGIPCEEGNQSSCKVLLTTRLRGVCTLMNCQREIPLHLLSEEEAWMFFKEYSGIGNNSPSELLKVASNVAVECKGLPIAIEAVGSSLKKKPIEVWKAALYSLKHSKPMDVEDGVRDAFSCIELSYNHLRSKRDELMFLMCSMFPEDHEIFVEDLIRYGVGLGICGEVESIDTARNQLRASINKLVNSCLLMHSDKNKEHFSNVNRADHVKMHDMVRDTALWIASRSENKKILVNLVKDLNTLVENGDINDYFAVSSWNKKTNRIAAQVTAPKLEFLLLSSRMSLDIASASFEGLKEIKVMAIISEGYRTLLSLPHSTQSLTNLQTLCLRGWDLDDISFILNLTKLEVLDLRACRFKQIPKEIERLNKLKLLDLQGCAVLENYNSEAIGNCEQLEELYVSGPSFQKDDKCMFPCQTFLDDVISSNLQRYILELGPLQTYGHGINENSSVRALSLKEFDISKFGASKMNLLQRAEDIYLNRLRGGCKNVAPELVKAAGSMNDLTKLRLRSCSEIECIIDTSSSGSYFQLDALMPGLVKLELEEVENLKELCHGSPMHALSFFEKLEELYIRKCEQLRNIFPANCELRSLKILKIDGRGLYQPTVAISCAAVALFSMSAAKSLEQLEELSIADCKDLRCIISNEEDNGNEDISQALNNSQSMLPNLKKLCIHYCPKLEFVLPSFCVGLEKLQEIDIFKASELKYIFGNEYQNEIQTKLPNLKSLKLQNLANLIQICRGNSQPWWPCLRELRCVNCPKLSTSCVNVMVRSTMRQQHLHKGVSLEEDQGKHLASELEQVEFRGFSELRFIWSDPTNRQILGLQHLQYLKVDGCAKLKSIFSAVILRSLPELTSLVIQGCNELEEIVSENEELHHDLSNTKVVCFPKLRNLTVKNCNKLKSIFSVSVLGMPPQLSYLHISDAAELVQVFRNSSDKIVFPNLREMKLNKLPCLVDICIGFELLQPMKAVKIMVDQCPNFTSISEAT

>arahy.Tifrunner.gnm2.ann1.370D23.1

QEAQLIDEIVEAICPDLHNNCYKDELKSPFLCNRNYFSIKSLLLKFKLEKVLVIGIWGMGGIGKTTIATVLFHEYSLQYEGCCFLTFSKKLERQGLNHICTRLLSQLLDQDFHIDNIRVIHSSIIRKLKHMRVFIVLDDVTNSQIATALVEFLCNCLSSGSRIILTTRDRNVLTSGGVKEIHEVKEMNYEDSLKLFSHNAFNDSHPKEGYYELSTRVVTDYAKGIPLALKVLGSFFHTKGESEWDSALRKLRKYPNADIQQVLRLSYDALDNDEKTILLNVACFFHGHKMESVTRILNSCGFFTDIGIRTLLDKALISVASDDCIKMHSLIQQMCWKIIHEESSKSDGQQTRLWNTKEVCNILQDERDVYGVESMIVDMNQITIDPHVVIIALRKMPKLKLLALKGDINMDIEWKLPEDFQLPNDLRYIKWKKCPLKFVPSICWPQKLVQLSMPGSSVQKLWDTIQNIPRLETIDLSGCKNLIECPNLEGAVNLKSIMLYECGSLLDVHPSIFSLPKIKKLDVSYCKALKRLCSEYCSPSLRCLWASDCSKLQEFSVSLVSNLDWFGFNISYSLEKLSQNFAHKIVLMDPIKHEDDTCIILSKILASPAFLFVKRLYFYKCKSLSKLPDNIHVLQTLQVLAVEICNVITSLPESIKNLQQLIHLYITDCEMLQYVPPFPPSIIYFCVINCKYLETVSSLTSEPPMKHKALFGLHNCTKLDDHAYAAVLKDLKSRIELVASDGYRNNGANNGNISFYYLPSKESILNDWFPHYYSTEASIIVEVPSDHNISSCLVVCMLISQYQSCNIGKKEVIFGCECYLEKGCNKWEWIATSHSRASLSICFDPFIRLEMVSDHKAVWYDAEYSNKIMEAIKERKKDTTCNPILKFELSAETVDNEEVVINGCGSVGCM

>arahy.Tifrunner.gnm2.ann1.38ZPK2.1

MLGSSSSSPPPAMKYDVFISFRGEDTRDGFTSHLCTALRKRHIETYMDDRLVRGEEISPALEKAIEESTIYVIVFSQGYASSRWCLDELTKIMDCKHRFRREVIPVFFMVDPAHVRHQIGTYGQPFEKHQQRYEDKVQGWRSALNQAANLSGWDSRAFSYTRLFDDVSKKIEEYKNDGGYKDRRHMLQRLKVLLILDNVNSADQLEDLIGGRHSFGPGSRIIVTSSDKQVLNNVTDEIYEVKNMDFQDSLQLFSLHAFKQNYPIESYMEVSEKVLNYAEGIPLALKVLGYFLYGRTREAWESELQKLEKLPNPKIFDVLKLSFDALDEEQQDIFLDIACFYRGYEEHTAKQKLNDSGFSATIGIEVLKERCLISVLNREIVMHDLIQDMGQEIIRRQGVNDPGKRSRLFKHQDIYQVLKKNKGTDAIQCIMLDMCKIREVNLHAETFNMMHKLRMLQFYTSKSAIHSNVHIPEFLNILPEELRFLRWDGFPQISLPQDFCPENLVELNMRDSHLERLWDGHQDLPNLKRLDLSGSRKLIQIPDLSQSPNIEEIILSHCVKLDVVYSSSFLAKLSCLWLDGCYELRILNLPSSILLKSLGVVVLYNCHSLEMFSVSKTSEGVLPSGCSRDGTRRWKEEKKCAPDAPQRHYFDIFHPIVSAKEYENTGDNIHLLRFKVLREGSPSSFPGLKEICWLDLSNCESLTILPVELFQMKFLKRLDLCGCSSLESLPEINQTMENLTVLILDKTGIQELPSSLHHLVGLEELSLHGCRRLEFIPSSIGSLSRLCKLDLTYCELLETIPSSIFKLKLSKLDLHGCSRLRILPEIMEAAKSIAHLRLTKAAVKELPLSLDHLVGLTTLCLSLCSDLEFLPSNIVNLSLLSDLDCSGCVNLTKIPNNIDRLSSLRELSLQGSGIVNLPESIAHLSSLKSLDLSDCKMLECIPVLPPFLKQLLAFDCPSVRKVSSSRLKLPPDSKEGLFKFHFTNSQELDPNDHSIAANACDMINQDAYRSVLFCFPGSEVPHWFPYRCRGNSVTLDPGSLNPINGDRLIGFALCVVLGSSEDQRNKDREFLFGLKFEYEGMHLLANNDLLRNYFYWKGKRIFKVHHTLVWKYTLESSAINYMLSRARNFNFEICEESHGVPTSNVVECGICPLYSKEKDNDFPAGDYWVHYKVCAPGH

>arahy.Tifrunner.gnm2.ann1.3L0H24.1

MALTELISNELTGELLRNLVTISRKSILCRGSADQLITYITELLPTIQEIKYSGVELPQPRQQQLHRLSEILHSGVELAHKVLNSSRWNVYKNFQLAKKMEKLENTVSKFIEGPMQTHILADVHHTRFEMAERFDRIEASNRRLEQYFVTMKMGGMGGGGWVEEAVRSMEIAEGSLGNSRVGLELGKKKVKEMLVGRNDLWVIGISGIGGSGKTTLARDVCRDEQVQFSFKERILFLTVSQSPNVEQLRARIWGFIMGNQNLNPNYVIPQWMPQFDCKTETRNLIVLDDVWSLSVLQQLVCRIPGCKFLVVSREKFQICNATYEVELLSEEDALSLFCHHAFGQKSIPVAANENLVMQVVTECGRLPLALKVIGASLRDQNEMFWEGVKTRLSQGQSIGESYQNNLIERMAISVNFLPEKIKKCFLDLCSFPEDKKIPLDILINMWVEMHDICQTEAFTMVVELSNKNLLTLVKEARAGDMYSSCFEISVTQHDILRDLALNMSNCDSICERRRLVMPKREASGLPKEWLRYKDKPFEAQIVSIHTGEMKEKDWCNLEFPKAEVLIINFSSSEYFLPPFIQSMPNLKTLIIINYSASYACLHNMSIFEKLTNLKSLWLEKVSTPELSGIVMKNLSKLCLVLCKINNSLEGKELKEADLSRIFPNLTELTLDHCDDVTDLPSSICEIHSLQNLSLTNCHNLTKLPAELGQLRSLEILRLYACPDLKSLPLSICNMIKLKYIDISQCVNLACFPNEIGKLVNLEKIDMRECSMIRSIPKSALSLKNLRLVICDEEVQDMWIDVQKAKPKEFHIQVSEQQYDLDWLKD

>arahy.Tifrunner.gnm2.ann1.3RA443.1

MASSSFNVPLIKHDLFISFRGEIRTSFLCHLIKRLRDDGIISFFVDEENLGAGDEISSALLQAIEESSISLVIFSKDYASSRWCMEELVKIFECKEQYQRTLVPVFYNVDPSHVRHQKQTFEEAFDVHTEKYRENMAKVQNWRSALRKAADLSGIHYPSTFIRNEVELIEKIIEVVNEKLPPICPNKSKGVVGIDEQVKSIESLLAKMEPNDVGILGIWGMGGIGKTTIAQVIFNKYSSQYEGCCFLKNVREESERHGLDYLYEQLFSQLLKKQNLLVKGSANAISAIYERRLSQKEVLIVLDDVDTSDILDYLTGEQTCLAPASRVIVTTRDKQILIAARAHGIYEVKRLSFKSSLELFCIKAFNKIYPENGYEKLSQMAVNYANGIPLALKVLGSFLCSKSAVVWKNALKKLQSCPDKRIFNVLKLSYDGLDDSDKSIFLDIACFFKGECKDNVIRFLDSCSFYADVGIDNLESKALITISYNRIQMHDLIQQMGWEVVRQESNKDPTELTRINKPEDFHNLLKNSKGKNLVEGIMIDLSQIGDLNLNADTFKNMPQLRFLKFYAPRDEKQLNVYIPIPSESFSARLKYLEWNSYPLNSLSSMFCVEQLVELRMPNSQISKLWDGTQELPNLIVLYLYGCNKLVELPDFSKATKLKTIDLWNCEKLCQLHPSILSIQTLEDLYLTGCSKLKCVKSNFKSLKTFYAYNCLSLEKFSVSSEKLSDLDISLWRIKSLPNEICSFFSLEYLCLSNCRKLIDLPRNIKAQSRLRALDVSGCSSLQSIPELPPSIEELYAVDCTSLETIFNLKAVFSLNRRKISFANCVRLEQESVNDIMEDAHLTIFRNVLLLSADPDQMINYYYYHKMLGCVCYPGYKVPKWFGCETRGASIIIELHQPYYELLGFFFCCVISQNLPSYYFEELDLVNIKCEYRHFGDGVKDTFVSKNLNFVSKRRCCCDHVLIWTDPFGSENILGEIERCRLRGGSDDDDSTCNQKMSFRFSIDRPKRGKRVQIRRENDEDCFIKGCGVIPMYASTLLDAIQNLELEFNLKPHHNPIPGINLDEVRSMMIRKSKGKSKLH

>arahy.Tifrunner.gnm2.ann1.3RLS73.1

MSSKYSGDISTDLDRLSTWKSSLESKIWTLAGQLGHRDGASAPPLESDNVHGDQQVITSTLSDDEDSSSYHSVDSNISEVSSLCEEDTPPVHSLDSNNSEECVDSHKQVYRYDVFLSFRGPDVRNTFVDHLFERLERRGIFTFKDNVRLKKGKRIKAELMDAIRDSRLAIVVFSSTYPTSTWCLDEMSTIADLHRQNKQIVVPVFYDVPSSDVGSQTGPYEVHFNSERFRKFSNRVARWKVDMKYLAKVCGFSIVDSNRPETTHLEEITSFVAKELNHRFTLLASDLIGIQPRVAELERRLNLTSGNVAFQILEIWGMSGIGKTVLAMILYDRISHQFDACCFIQGVNGIYTRHANACETATTNVQKQILLQLFQEQVESDDPAEIAKMLQNRMCNRNLPKRVLIVLDDVGDPKQWDDLGIVPDSLGLGSRVVITTRFQHILNVNTAYEIHEVQLLNDDEALELFQRKAFKIDCHSLAISDVSRRIIENAQCLPSAIIKLGYYFNQKAEAHWERCFQRWREYPEEKYMNSLQEKDYEVLNEDEKMIFLDIACFFTGKNKKYVEHILGSRISDPHLAIQEIRKKSLIKIRNHEIHMHQILQDLGKKIVRGNNKDEPKCWSRLWNAEDFQEVLIDMEGNKVQAIVLDEDVSKYMKIGGLSELRDLRLLILHHHKSSSEKLTFRFNKLCYLSWHGFSYTSLSLCIWSNLAELNLPNSSIRRLWEGSQAIPNLKRMNLRNSRDLVITPNFACCQGLVRLDLTGCINLTEVHDSIGLLRELNYLSLRECSSLSLLDFGPNCQLSSLRTLLLSGCTNLQQTPDFTELSNLRYLDLERCTSLSTMHESIGTLATLKYLSLRGCKNLVHAPYILNGNSSLLILDLSGCMMITNLPRCRRKFAPSSCLESLMFLSPVFLEPQRILDFVGELMFFDGEWICSKLAYLNLAHCHELRRFPEASFHGLHEMKGTLERYPQLSGFHWIKDEGKGVWEQRTEPQRYDQNKKSRPRRSGSDFREIDMSQELKNWYTLWNKWLQLLEQSKTLSFLHHCLLHCQVNLTSPLIRWKESAPHRLSEFANTTGADTRNTFVDHLYNHLIRKGIFIFKDDKTLEQGQPISSQLMQAIRDSRVSIVVFSKEYPTSTWCLDEMAAIVDCQREFNQAILPVFYDVDPSHVRKQNGVYHDAFISHSQRQQLNKVRRWKSAMTTLANSVGWDVRNKPEFKEVENIVQKIVKTLNHKFSGFADDLIGMQPRVEELEKLLKLRSEDDGFRVLGLWGMGGIGKTTHATALYDRISYQFDASCFVENVSKLYKDGGAMAIQKQILRQALEVKRLDTKSPSEISGIIINRLHSIKVLIVLDNVDHLKQLEELAINPKLLCEGTYELFSRKAFKREGPSNNCEELIPEVLKYAQRHPLTIRVVGSFLCSRNATQWKDALDRLRNNLEDEIANVLRISYEGLQFEEKEIFLHIACFFRGEREDYVKRILDSLGLYPHIGISVIAEKSFITIRNQEIHMHEMLQELGKKIVREKYPQEPGSWSRIWSYKDFHHVLMSETGTNEIKAIVVDIKEDISDCNQMVEGLAKMKDLKMLIVHQKNNHSESRLKSLSSCMKHYLSSHGYPFPSLPSLFHPSESKLKCLSSCLKYLSWHGYPFPSLPSVFHPSECLVEMNLSGSSIKSLWEGRKTFFRLTILDLSNSKKLMETPNFEWCTSLKRLDLSGCTNLQHVHPSIGLLARLVYLNLRNCSSLVTLYFGGDECKLSSLIVLHLSGCTKLETMLDFSRILNLEYLDLEQCTSLAKVHESLWSLGKLRSLSLRGCLGLKEGPNDISWMTSLQTLDFQGCSRLLKLQLILNKYERSKLHQTLGKSIISPRISKALTFLDLGFCNLGKVPDTIGELKGLERLNLQGNKFNSLPCTIESLSSLAYLNLEHCFNLSSLPTLPFVNNSSGGRYFKTISGSRNHGSGLYMLYCPIVDIQRCLNWAFVWLTRLIKQPCHFRCGFDIVIPGSSIPWWFNRLFVGGSTIRIVDSNNMDDNWIGFTFCVAFCVTDPSAITGSSHNRLSTSLPSPFYLSFESEQAEETFCMPCRLDFKGGSQPPKTQNLEHVWIIYISRPHCHFVKTGANITFKACPGIEMRKWGLRMVFKQDIEIIQRNSQYQLEISMFVCPSEILLQDHGLVIEEVPESNNSSIGPKVQLPYNWYVTDEEEKENLEAKSKEINLANIGL

>arahy.Tifrunner.gnm2.ann1.3SI7RL.1

MMAHASFSRVSQMAYVIFVCLLLILKPKGVTSSSENTLKIKYDVFVSFRGPDIRRGFLSHLVGALSRKKINGFVDDKIEVGDEIPKSLIRAIEASLISLVIFSPNYSSSRWCLEELAKIVECREKKGQFVLPVFLDVDPSDVRHQRGSYGDAFVKHEKKYDLVKVQRWRTALQRAANLSGLHSTKYMNDYGLLEAIIKHLIERLNHEQKYISRGLIGIGKSIALLESELNPELGDVRALGIWGMGGLGKTTLALEVFNSVQSQYEGSCFLRNVRESSAREGIFSLKSKLYSELIGEPGLKIDSSNGLPPFLEKRLGRMKVIIVLDDVDNFRQLKILFGAREQFGSGSRIIVTSRDKQVLHTEVDFIYQVKPLKVDEALQLFNSIAFKHEHPDLEMEFHEQSKKVIEYAKGVPLVLEVLGHHVHGKGKEIWESLLDKLKNMPDKEVHNVMKLSYDDLDRDEKKIFLDIACSFKWLHVTEDYIKQLLKDEINSVAFGLERLKDKGLITFSKGTVVSMHDIIQGTAYEIVRQESIEDPGKRSRLWDSNDIYQVLKADKGSEAIRSISAKLPLLNKKLQLSPRVFAKMSNLQFLDILAPYTCPSYFPQDQMSLYFPEGLESLPNELRLLVWLHYPMEALPTQFSAENLQILSLPVNRVKELWHQEQNLQSLKVVVLECSTQLTESPDFSNAINLEVLGLSSSLKLIHVHPSVFSLEKLTTLYLVNCVSLTSLISDTHLRSLRYLFLSGCTALKEFSVISMNMVYLYLEHTGIKQLPASIGIQSKLEKLNLANSTIEYLPETIKHLLRLRSLNLRDCRELQTLPELPPNIEDLNVEGCISLKTVLFPVTVAEQMKEERKKVAFWNCVALDQHSLEAIGLNAQLNIMKHLSRFESDSYQDYDADYDADGATASYVYPGSRIPKWLLHRTIGNHITIDVPFGLPSSQLRFIFVFIVPKVASEGLFLNFSLSVGDDEDEGKSIKLRLSRPAQEIASDHVYLMYDVACSNYLSSKAKSQPQFEITVSVASEYTPLLLKGLGVSLVNVAEYQSFVQQIKWLDTTSITRETDSYNCELSYQNCSLEFGKFMVPSAYFNNMCTRLEQAEEGCDS

>arahy.Tifrunner.gnm2.ann1.3TW696.1

MAGAVVGGAFLSGFINIVINKCLTEDAVNKVFGKKLGSDLVQRLKTALLGAEALVADAEMKQFGNLNVRKWLDSLRDAVYCAEDLLDAVLLKATTQKNASFSWWSPSFFINQDRDDMVDKIEGVVTRIEDLGKQKDFLGLEKILTGSSSWRTPSSSLVKGSVYGREDDKKALIKMLNDNNEHHLSVIAIVGIGGVGKTTLAQWLYNNQEEFMKRFDLKAWVCVSEKFEVVETTRNVIKQIHGGTCSLDDFNSLHKALKEELSNKKFFIVLDDVWSDDGDKWSNFMTPFQYGKKGSIVLLTTREENVASAVQNCRPYFLKKLSEDYCWSVFAENASFPQSNGRAALEEIGKKIVKKCDGLPLAAETLGRLLRTKHDVEEWKKILTSHIWEFSVEKSKIIPALLISYFHLSPYLKRCFVYCALYPKDYQFEKDELILLWIAEDLLPPPKRGESLEEVGCECFDELTSRLFFTGIDDYFVMHDLLHDLAIFPAGDFYCNSEELGKEEEIKIQTRHLFVDLSHCSSKLYNSISKVESLRTLLLFANFSSPNCNTEAATCEILSKCKYLRVLLLKKFDEVPNLLIGELIHLRYLNLSWTYIKTLPESLCSLCNLQTLKLYYCSKLTTLPSGLHNLVSLRHLDIRGTSLEEMPRKMSKLKELYVLSSFVVGKHEDNGIQELGGLENLHGPLEIKKLENIVDVEQAESAKIMDKKHIDELWLEWCSGDDLVSSTETERDMLDKLQPQNGLKELGITGYKGTIFPDWVGNCSYQNMTHVSLLSCKNCCMLPSLGQLPSLKSLRIRGFDQLRSIGEEFYKNEGAHHSSHIAPFPSLERLKFDDMACWEEWHLPDSKAFPQLMILHIRNCGMLKEEMLNQVFFRIVSSLSDVSKVRKLHIGDEFYPQKKTMNLDGDRLSVTGCESVRESALKAMKSMNHLSCLQEMDISWCSSLVSFPGNCFPKSLQKLKLLKCSKLEFPEQQQHKYDLVELQIEDSCDSLTSLSLDVFPNLKNLQIERCWNLESVSMSEAPHAALQRLSIYDCPELVSFAGEGLAAPNLTYLKIYGCRNICRLPKGGLPPNLKSLGVGIGEQQMRDLLWMSNLHALTHLRIEGHYCDNIKSYPEVGWLPHLPSLTTLEIWYFHNLETLECNELLRLTSLQQLHIRFCYKLENMEGEKLPPSLLLLELRRCDLLGKHCKNKHQLIWPKISHIPDIIVMIIFLSVRPKQTLFGESLYIPISVYTGTNLLALTLIQGSANVRLS

>arahy.Tifrunner.gnm2.ann1.3WN25H.1

MAEKLYGGAYLSPFVNTVLDNMSSILEEDDSFLERNNLLVRLEKSLYDVGPVLDDAELKQFTDKRVKKWLVDLQDALYFADDLLDELSTKATIDATQREPGNSSSWSRLVDSYIEDTGDLEKIVRRLKSAVARKNYLRLKETAKVDISWRIPSTCLVEPSEICGRKEDKEALLKLLLDDDDAADGDLSVIPIVGMGGIGKTTLAQLLYHDDKVKENFDFRGWVCVSAEFDVVKVTKTVIEAITSSSCNLTDLNLLQLDLKEKLSRQKFFIVLDDVWNENYSDWDKLLKPFQKGVKGSKILITTRNKNVASLVQTVSPHELRSLSDEDCWLVFTKHARLSTVSVENPTLEKIGRDIVKKCDGLPLAAQALGGILRGNSDIGYWKHLLKSEIWKLSHDRINVVPALMISYYFLPSYLKQCFVYCSLYPKDYEFSKDELMLLWMAENFLQPVEKKTIEEVGGEYFDELIARSFLQPHSTKKNKFVMHDLVHDLAMTCAGEFYFRAEELRNAVEVDIKARHLSHNAKGNYPMSNLLGVCDRVKHTRTFLEINLNEWIPSNMENAACILLSQLKYMRALSLKNFPLVSVPDSIGELIHLRYLDLSGTDIVTLPESLGNLYNLQTLKLNWCRNLKMLPVCMKDLVNLRHLDIRETRVHVMPKGMSQLKNLQFLSNYVVGKREENKMTELGALADLQQSISIAGLENVVNSSEASMARMFDKDGICSLQLSWSPDEDENIVDSQTERDILDKLQPHTNLKELEIRGYRGTTFSDWLGSSSYRNITYVRLDGCRNCCMLPSFGQLPSLKGLSISGFESVEIVGAEFYFYQNDESCLETPFPKLKILSFSSMRCWKEWRSLEFNGFPRLRHLNISECPMLRGDLPNHLPSLQSLQIEKCDEVSCCVPRAPAINSLSISGKHLVGSVVEAITNTQLTCLTSLCISDCSSHIWFPVSAIPPSLQKLKIRDCRKLEFEMDGQHHSLQELYIRSSCDSLASFSLLDSFPNLVRVEIKYCGNMESIVVSRSLSCLRYLWIKHCGSLKSVSTIWMAAPQLERLSLLGCPEIDLCAPGVPHRSLRYLSISYCEKLVSTAAFMNSQFQGLIHLRIEGECESVKCLPKEDKGQLESTSCVKGLLLTCCLHLPDVRMMIKSTVGKVYSIPLKDTAHEIEAADKQRRECSDIGITIGATI

>arahy.Tifrunner.gnm2.ann1.3YZ3MF.1

MAAEAVLSSVLSVVFDRMSSPEVVNWIKGKKLTQNLIERLKTNLYAVQAFLIDAEQKQIKERPVKNWLDSLKDAMYVADDLLDEVFTKAATQKEPGTFLSRFLNLQDRDVANRMEEVIDRIESLVIQKDTLGLREIPKENMSWRITTSLVETSDVCGREEDKEAIVKLLLDDDGDDTGGHSDVSVIPIVGMGGIGKTTLAQLVYQDGKVKENFDFQAWICVSEEFDVFKVTKTIIEAITSSFCSLTDLNLLQHDLKEKLSRKKFFVVLDDVWSESCEDWDKLLKPFQKGVKGSKILITTRSKRVASVVQTVSPYELSLLSEENCWLVFSKHARLSTVSMENPTLRKVGRDLVKKCDGLPLAAQALGGLLRGNSDIKYWNHLLKSEIWELSDDKIKVVPALRISYYYLPSYLKECFVYCSLYPKDYEFSKDELILLWMAETFLQPAGKKTPEEVGDEYFDELTARSFFQPHKIRENKFVMHDLVHDLAMIFAGEFYFRAEELENAVEVDIKTRRLSHNAKGNYPISKLLEVCDRVKHTRTFLEINLNFLIPFKMENAPCILLSKLKYLRALSFNGFPLESLPDSIGELIHLRYLDLCYTRIMTLPDTLCNLYNLQTLKLVGCWKLTALPVSTKDLTNLRYLDITQTRLHEMPEGMSKLTSLQVLSRYVVGKREGNKINELGALANLHQTIFIDELENVVNSSEALEARMFDKDGIESLILKWSPDEYENTVDSQIERDILEKLRPHSNLKRLEIYCYRGTTFPDWLGHCSYHNITQITLGSNFWGHFRNCCMLPSLGQLPSLKHLEISMFERLAIVGAEFYRNDESCLETPFPMLETLTFESMPCWEEWRSLEFNAFPRLRELIIMDCPMLRGDLPNQLPSLQSLQIGNCKQLSCCVPRAPAITSLRVEGNNEVRIGELPPLLDKLSINGKHQAESVMEAIAHTQLTCLTSLSISDCSSHVLFPVSSIPASLQKLRILDCKKLEFEMEGQHHSLHKLMIQNSCDSVTSFSLDSFPNLVRVKISKCKKMEYLVVSRSLSCLRSLEIENCGSLKSLQTLWMASPQLEDLILLGCPEIDLSATGDPHRSLRDLTISCYEKQLSCVASQFHGLTYLCIKGENESVKCLPKEGWLPATLESLRLEFITSVKMLECKGLAHLTSLQELTMNHCYNLENIDGEKLPASLLRLIINGGRLGKRCEMKDPQVWPKISHIPAIQVDGRWIWNSQKQDNHNKVVRMAEKATEQPGTIMDTIQEIQEWHDTLIDIERNPNELHQLFSGTYPFQD

>arahy.Tifrunner.gnm2.ann1.43UGZW.1

MAAAAVGEALLSAAVEAMIGKITTEISEFYRSKNLDESLLEKLESTMRILQAFLSDAEEKQIKNAAVKAWLDDLTQALFDADDLLDDVATEALRRKVEARYVPRRSLAVKVRKVLCYPFKRPYGEINSQMQKLIERLNGFAERAHMLPLEKGVSGSDWRTTPTNSAVDDSAICGRDDERKNLKEYLLSEDAVADGGGKIGVLAIVGMGGLGKTTLAKLLYNDPHVKGKFDVKAWTSVSKDFDVVKLAKSLLESVTSAATNLDNFDALRAELQKNLSGKRFLLVLDDIWNAGYVAWTNLMPIFNVGQMGSKIIVTTRHQSVVAVVKASQICFLKPLANDDCWGLLSKHAFGAHKCNELSSNLEEIGRKIAENCGGLPLAAVAVGGLLGTKVSTEHWTKVLNSNIWNLTVKDVQPALLLSYHFLPASLKQCFAYCAIFSKNSKLQKEALVRLWMAQGFVCVSQNEKSIEEVGGEYFDELVARSLIQPSVEGQHFEMHDLINDLATMVSSPYCKRHDNEMQLGNLNKIRHLSYDKDMFNHFGELDSFHGLKGLRTLIALPFYAPYRSHYLANGVLQELLALKQLRVLSLSNYRSITLLPNSIGDLKHLRHLDLSCTGIERLPPAICKLYNLQTLLLSDCRYLTELPEEMGKFVNLRHLAIDNCGALTKLPEGMGKLVNLRCLDIDGSNLQEMPVEIAILENLQSLSGFIVSKQQHGLKLAEMRKFPHLQGKLCISKLENVIDPSDACQANLKEKKKIEELSLEWSDSILEDSHQVVLEHLQPSTSLKNLSVKCYGGSTFPSWLGDSSFGNIVSLSIEDCRHCSSLPPLGRLQSLKELFISGMRSVKRIGSEFYGSNSPSFQPFPSLETLIFASMAEWEEWNMIDGITSEFPRLSKLSLSRCPKLKGNLPSNLPCLVTLVVEDCCLLESEFSGEVDNRNIMRPMNVFNFNSLRQLYLFDIPSLTSFPSNGLPKALKTLFITNCENLEFQSHEFLQSCKALEDLDIMRSCCSLTSFPLGSLPLLKHLKLRDCKNLKSISILEEEAASQSLKFLEHLQVEECPEVESISLLDLSTPNLSYFAVVRCDKINSLPEPINNLTGLQTLYLYDVPNLESIAEEGLPINLRTLGVGNEEGVCSNTAITKWGLDRLTSLSYLSIKGEYQVKKLMEIQVPLLPNSLKILIIWSAREIQHLNGKWLQHLTSLKNLALYECEKLKSLPKEGLPSSISRLDIWRCPMLKASCERKKGKEWPKIAHIPLITITDCFSDKSEPRPPSPLFCLIWSLPLPSSPALAAGLSASPGVILSLSPSLVYSQYLSSLFASFASERHRSLCCRRGTRVISFSIVILSESASSFSIVSSLHRCTSRVSIEPQPQTIVDVFATSLRSMKEKCSEPKEDEQKLKKNKNKIKKKKKKKGETADKKGKGDRFEKLEALSRRLRHCLLKGSIH

>arahy.Tifrunner.gnm2.ann1.45SZFM.1

MENYDVKFLCSYGGEIHHRPNKKISYVGGHNKLHYVNRGIDLTAMLAELSALFDAAGDIHFKYQLPGDDFDALISVTSDSDLNSLMLEYDNLYRDSAKTARMRLFIFPGNGPDSASTQPFPAKLKSKVNGGAVVPPQITPVKFQDLPPVNNFNTSDRVLVSDPKVNRPVEDSGSSRLNSPLPALQYDVFISFRGEDTRASFTSHLFKSLSRKQIVTYTDDLLHEGDSIMSLLRAIEESCLSLVVFSENYASSKWCLQELVKIMECKKEYGRLVIPVFYNVDPSHVRYQLGSYSEAFKKHLQKNKKAEVQKWREALTAAANLEGLDSRSYRDEIEFIQNIVKDVLQKLIDHNPPNDSKSLVGIGENLENVKSLLSESVEVRMIGICGMGGIGKTTLARLIFEKYSYKFEGSCFLENVRKRSGKSGLTELVRRLYLELLQGKFWQNNTGKSTFVEDRLSKQRNFIVLDDVSSLEQLNYLVRKLQCCGAGSKIIITARDKNVLVPTVETIYEMKILDSHESFKLFSLNAFNEDCPQIGYEELSWKAVGCCKGIPLALIVLGSFLHSKSKTDWHSALQKLEKTPDPEIQNILRLSYDGLDDEAKQIFLDIACFFKGELVEYVVNLLDSCGFYAAIGMRSLLDRALIAISHNCVRMHDLIQELGWDIVCQQSSGNAENRSHLWDSNDIWDVLGNNKGTDSIESIVLDMSQIEDLQLNADTFKKMPKLRFLKLYIPSENDGRLNKLHLPVGLKPFPSKLRYLEWDAYPLPSLPSNFCPEKLVTLRIRNSKLKRLWDGVQNLVNLEEVDLTDSRKLVELPDFSKADNLKSVHLSGCRSLRHVHPSLLSLGKLELLDLLNCAKLEMLETKMHSKSLKHLSIKSRTSLIPPYVSHLSSLRKLLLDGSPVETLPVSIKHLTGLKTLSLKGCKMLQHLPELPSSIRHLTALDCIMLQTVTFSSNIPRLQEEKRINISFHNCMKLDVANCIYWYLKDIRKLAYVCESRRGGKGVVRRSDFFKICYPDYRVPEWFMHRTKGTSITFEVSSPSSYGFSSLLCVVLPKYSLDYELDIKCRCYLEDGSNMHKYSFGILFLNHIPAEGCSDHVYMAYNYGGIFDVIKLDRLNNKIASSGQNPKVTFEFFVSSGDTGSKQDDNLLIKECGVYPLNDSNFRTE

>arahy.Tifrunner.gnm2.ann1.4970S0.1

MECKKEFGRLVIPVFYNVDPSHVRYQLRSYSEPFKKHLQNNKKAEVQKWREAFTAAANLEGLDSHSYRDEIEFIQNIVKDILQKLIDHYPPNDSKSLVGISENLEKVESLLRSYGLTELRHRLYLELLQEKFWKKTGKSVFVENRVSKQRNFIVLDDVSSLEQLDYLVQKLQWCGAGSKIIITARDKNVLVPTVEIIYEMKILDSHESFKLFKSSWLLQRHPTSLNSIGFFSLFKELSYDGLDDEAKQIFLDIACFFKGELVEYVVNLLDSCGLYVAIGMRSLLDRALIAISHNCVRMHDLIQELGWDIVCQQSSGNLENRSHLWDSNDIRNVLGNNKGTDSIESIVFDMSQIADLQLNADTFKKMPKLRFLKLYIPSERLKPFPSKLRYLEWDAYPLPYLPLNFCPEKLVTLRIRNNKLKRLWDGIQRRVTSFSGQKFNGRDGKGCDNDLTTCATPSNLLNQEEERGGGQDITLGAV

>arahy.Tifrunner.gnm2.ann1.49I9B6.1

MAESFIFSIAESLITKLASRAYQEASRVVGVYDDLQDLKTSLSYVKAVLLDAEQKQEQNHELREWLKQIKLVFYDAENLLDEVDCESLRKQLRITQKDKVGGFFSSSNPLLFRYKISQQIKEIRKRLDRVAADRDKFGLQTIAVDRRVGHKREMTYSHVVESDVIGRDHDKEKIVKLLMEPSLDNNAGSKHISVIPIVGIGGLGKTTLTKLVFNDKRINESFPLKMWVSVHDDFNVRSLIIKIINSPRDFAASTDAPAGQQNLRDLEIEQLQYRLRNMLEGKKFLLVLDDVWNEDRVKWVELQHLISVGAQGNKVIVTTRSQSIASIMGTVAYSHHLKGLSPKDSLRLFVRWAFKGDEGKYPDLVMIGREIVEKCKGVPLAVRTLGSSLFSKHEIQEWESLRDKEIWNLPQKEDDILPALKLSYDEMPSHLRQCFALLSLYPRNFRFDSFGVASLWGAAGLLPLQSKDKTMVDVAHQYLRDLMARSFLHDVIDCGTFYFFGIHDLVHDLAVYVAKDVCQLVNSNTQDISENVLHLSFVENGLPYNSVKPSLRGTRSILFPVDEVGASEAFLNAWVLNCTYLRYLNLSDSTFETLPESIGKLKHLRYISLCNNTRIKRLPNSICQLQNLQVLLLGRCSNLEFLPKKLRKLISLQRLGITTKQSILPESDIAKLNCLEYLRVEDCDNLESLFAETRLPTLLTLQVIGCANLKSLPLDTHHFPQLETMLIEGVGNEDWLHRSENTNSVLRLKTISLYNMVTLPHSLQQYASTLQTLMIGDCYELEVLPEWQLNLSALKFLYIWDCPELKSLPSDIHRLTSLQVLQIINCTELYRKYKPQVGECWPMISHIKHTEIRKTWD

>arahy.Tifrunner.gnm2.ann1.4AL876.1

MRSFQVTSLLIGLKKLQNNSMKKVQKWREALTEAANLAGWVSSSCRDESELIQKIVKDVLQKLIDHNPPNDIKSFVGIDGNLKAIDSSLREVLEVQTRMIGIWDMGGIGKTTLAKLVFEKYSYQYEGSCFLENVRERSEKYGHALHELRNELYSELLQENNCKDSTRISTNAKERLRSQRNFIVLDDVSCSKQLKYLVGEHQCYGPGSKIIVTATDKSVFAQWEDEEIYEMKVLNSKDSLTLFSLNAFNQDHPKMGYEKLSWEAVKYCEGLPLALKVLGSFLRSKSETEWDSALQNIKKIPDAPIQNVLRLTYDALNYEEREIFLDIACFFKGFLKEHVVSLLESCGFDAANGMRSLHEKSLIAISDNSLRMHDLIHEMALEIVRKESIKNPEYRSRLWDYNDIRDVLGNNKGTDAIESIMLDMSHIDDLQLSVDTFKKMSRLRLLKLYDSSEKNGKLSNLQLPIGLKPLRYFEWHAYPLPTLPSNFCPEKLVTLRIQNSQLKRLWDGKQNLVDLEEVDLTGCQKLVELPDLSKAKNLKSVHLSNCRSLAHVHPSILSLGKLELLDLLNCIKLEMLGNKKHSRSLKHLCVCGCSNLIEFALSSEEIEYLDLSHTGIKVLHPSIGRFTKVKEISLCGIRLKNLPDGWSRLKSLEKLSLFKCGRVVSKQKLHDIFDGLQSLQNLSLVNCDSLFELPGNVGRLSSLQKLQLDGSHVETLPGSIKHLPNLKTLSLIGCKMLQYWPELPPSIRHLKALNCTLLQKVAFGLFSYELQEEKRVSISLQNCVNLDVENCIYSFLQHVRKQAYECEFRRRGVGRENIVVWRSDFFKICYPDSRVPEWFTYRAVGSSITFEVAPPSSYYFGSLLCIVLSSHSLDFELDIKCRCYLEDGKMHKYSLGILFLSHVPVEGHSDHVYMTYNFNGIFDVIKLDQLNNKIASSGHKPKLTFEFFVSSGMARGKKDLNLLIKECGVYPLNDSNNCVE

>arahy.Tifrunner.gnm2.ann1.4B9DJ7.1

MAISTRWTNHVFLSFRGDDTRKGFTDHLFASLERRGIKTFKDDHDLDKGGSISVGLFKAIEESMFALIILSPNYASSTWCLDELQKILHCRSNLGQAVFPIFYGVDPSDVRYQRETFGEAFRKHEERFREDKDKVKRWRDALREVASFSGWDSKDQHEAGLVETIVDHIQKMLIPKLPTCTDNLVGVDSRIKRVISLIGMRLDDVRFVGIWGMGGIGKTTTARLVYESIKEEFQVSCFLQNIREMHEKNGLVQIQKELLSHLNVRSSNFHNFFDGKKIIANSLRNKKVLLVLDDVSELSQLENLAEKQEWFGPGSKVIITTRDKHILATHGVLKTCEIGGLFSNEALHLLSLKAFKQDEPKEGYLDLCKEVVEYTKGLPLAIEVLGSHLHGRTIEVWHSALKQIRSFPLPEIQNTLKVSYDGLNSPEQDMFLDIACFFKGMDKEEVTDMLETCGGCPKIGIEILIERNLITISATNKLQMHDLIQEMGRNIVFLESPNDPGKRSRLWSQDDIDHVLTNNKGTEAIQAIVLDIVQPYEACWSTEAFSNTNQVRLLKLCEMQLPLGLNCLPCSLKVLHWEGSPLKTLPFSNQLNELVDLNLSHSKIEQLWHGKKILKKLRFINLSFSKNLKKTLDFDGVPNLESLVLEGCTSLTEIHTSLVHHKKLVLLNLKDCKRLKAFPSKLEMCSLKDLNLSGCSELKILPEFGENMELLSMLSLEGTAITKLPSSVGSLVGLTQLKLNNCKNLVCLPDTIHKLKSLKILNVSGCSKIRSLPECLKEIKCLEELCASETAIEELPSCVFYLESLRVISFAGCKGQVSNSMEMFLPFKWLFGSQQAPTGFRLPPSVSRLCLLSSVDLSYCNLSDESIPDDFCHLSSLRILDLSGNDFVSMPSSISKLPKLEYLDINWCKKLESLPELPSSIKELDASNCASLEASKLNLSKPCNLFASPIQCHLPIQEIINRLFEGLSIPKARFDMLITGSEIPSWFVPQRCISFAKIAIPQNCPVNDWVGFALCFMLVSYADPPQVCNHEVDCYLFGPKGKMLINSRNLPLMEPYCPHLYILYLSIDKFRHTIYEGGHFKEIEFVLKSYCCHSLQIVRCGSRLVCKQDVEDIFGNHS

>arahy.Tifrunner.gnm2.ann1.4FG6XW.1

MHLISIKSNLYCIVYSQCTQYIHYTMDCMRIQSTLSHRKNWKYDVFVSFRGETRNNFTDHLYAALRRHGIVAFRDDTKLKKGGLISEELLEAIEASQVLIVVFSMNYASSTWCLQELAKIADCIQVPGHRVLPVFCDVSPSEVRKQSGNYEEAFVEHEERFKGDTEMMEQVQRWRGALTQVANLSGWDLKDKSQSAEIEKIVKAVTNRLSLKPSSSVSNDIVGIHSPVQELEKLLVLDSEDDVRVVGICGMGGIGKSTLAKILYEKIFHQYDVSCFVDDVSKTYKHDGPLGVQKQLLCQAFMEESFSMWNLSLANNLIQTGLRHRKVLIVLDNVDKEIQLEKLALEREWLGRGSRIIIVSRDEHILREYEVDHVYKVKLLNDENAHQLFCRKAFKCNHVVKDYESLTDSALAYANGLPLAIKVLGSFLFGRDVSEWSSALVRLKETPTKDIMDVLRISFDGLEDPEKEIFLDIACFFPDDEEDYVKDILRIRRFHPEIGIRILIDKSLITCSRYYIVMHDLLRELGRSIIREKSPNEPRKWSRLWNYRDLSDVLWENKAVKNLEAIVLPRSLKNREELSKKATLKVEALSQMNHLKLLILKKVNFSGCLDFLSNQLRYLDWEKYPFTCLPSSFQPNKLVKLILYHSNIKELWEGTKDLHNLTHIELCHSKNLVKIPNLSQAPNLEHLDIKGCIKLVHLDASVGSLEKLSFLNLENCKSLVSIPNSIFHLNSLQHLNLSGCSKLFRYQLLEKPRQSEQLNTDQSVQSHMTSSICKTLTRPLHFFYSKRLSSSVDLLVPSLSRFPALTYLDISFCNLVQIPEAIGQLHCLESLNIGGNNIVTLPHCIKELPKLGELNLEYCNNLKWLSSTLLPIGGASRQSCYYGGLYVFNCPNLSDMEGCCLTVVSWMIKVIEVAASIPCFSFFCVNMQCSLLKCIIQVVIPGNKIPRWFNKQNTGNSVSLDPFPIVDDNNWIGIACCVTFVVHHAPIQLLKRRSRVLVGCGFRSKPLHESIYPVVPIRLEKDSITTKLDHMLIVFFSREVFINLFVSYLKKGASDVDSIELAIMSDYPEEVELKSCGYRWLYKEDVEQFNPTMMYTATSSTQQHKFLAIEHVQ

>arahy.Tifrunner.gnm2.ann1.4PE3C2.1

SIMAAKHQGRAYLSSYVDAVLNKLSSLDVNSTPEAKKLADQKLFQRLKASLRATRPVLDDAEQKQIRDQEVNKWLVDLQDALYMADDLLDELSTKAVTATPTQRDPGNSSSWSHSVDSILEDGDDDEMGVVTRMQDIVDKLESIVEEKDDLDLKQGVAKDLEDMSWRIQSSLLESPDIYGRNDDKEAIIKLLLDETCDDKLSVISIEGIGGIGKTTLAQWVYNDVRLKDKFTINAWVCVAIKFDPVNVTKAIIEDIASSPCNMVNLNSIQTELKEKLTGKTFLVVLDDVWDNQQNLWDNFLKPFLSGNKGSKILLTTRNKNVDSIVSTTNLHYKLDTLSNEDCWSLFLKHSTISTSSRQYVILEQIGKKIVEKCKGLPLAVKTLGGLLRSKDKVEDWENILRSEIWELPEDESKIVAALRVSYHYLPSNLKRCFVYCSLYPQDYQFDKDELILLWMAEGLLQPMEKNTLENIGCAYFDEFVARSFFQLSSADAGLFVMHDLMHDLATSATSFAAKFFFRVDKFGNPHMADSKTRHLSYNRGRISKFPEAYNGAIYMRTFLPIDVQSNDIESDFWLQQLKCLRVLSFCGFKILSLPDSIGELIHLRYLDLSETPIVTLPESLCRLYHLQTLKLRDCEKLEMLPSRMQDLVNLRHLDIRGAYRLEEMPKGMSKLKHLNFLSGYIVGEQVENGIRELGALDDLHGSLCISKLENLKDSGEALEAKMGNKKHINILELKWISDDDDVFQKERNILVELQPHRNLKELSIDGYRGEIFPDWLGFSCYSNITELSLIRCKNCGQLPSLGQLPSLQHLRIFELDGLERIGDEFYNNAGSSHHGTPFRSLETLEFCDMPRWREWHIPNGFDGFLKLKSLSIQDCPVLSGDLPADLPALEKLTIRNCEELACSLPRAPKLHQLNVMGSGLFWYGYAAPHDVVIKNTQLAKSILEWLPHFQSPCLERLKIHKCSSAISISGDSLPDSLQFLKISGCSNLTISEPLQHKSLTEIIVSGCDSLTMFPLGALPNLKTLYISSCRRLVSVPALGCAAPHLDILEIHECPEMDCFGEECLPPSLTTLGIRRCQKLERWITWRGLQSEGLTHSDYDSDSVMIARRDERPRLGG

>arahy.Tifrunner.gnm2.ann1.4RXW7B.1

MADGVVSFLIQNLSQLLVDEAKLLSGVEGKVKSLISELKFIDIFLKSSEGKRNNNIVKEVVNQIRDVAYEAEDVVDNYVANVSLHRSRSILGKMFHCVGQAMMLHNVDAEIENIKRSISEIYSNRDKYGIGEGEFQCGGEATPAATEALRKRRRDVEEEDVVGLVHESDTVIKKLLKTDSRLQIASIIGMGGLGKTTLARKIYNSKKVKEKYPFRAWGYVSNEYRAKELLLSLIRCLSTWKSNEESEEELKEHLRECLNGKKYLIVLDDIWKTEVWDDVKGAFPDDNNGSRILITSRIGEVAAYMGTMPPYFLPFLTEEQSWELFSKKVFRGEEDCPPDLKDLGMSIVESCSGLPLAIVVLAGHVSKKEKSLREWSRIKEHWLRVRDNNTVVVMDILKLSYDSLPQKLKSCFLYFAIYPEDYEIPARNLIQLWVAEGFIQIPETGTSDTLALEDIAEYYLEELVDRSLVQVASKRSDGGVKTCRIHDLLRDLCVSESKASKFIEIHRELDINKSNSRKMSFHHSTQFSSSPNKNNHFHTHSLFLFGEELTWDDESKGCKQFRKSFKLARVLHLNKVLLDLPPSGLKLMIHLRYLKIKTDSRSAENILDSISNLRNLETLHLSCDWVVSIPIKIWKLKRLRHFNLRIKEISQMSRVTNNERMSNLQTLCEVPLYSETISMLNNNGSFPNLKKLGLCFYPNFQQSSTGAANLSSLSMNHLSKLCTLKIRGDLFNPFSANGFKSTFFPSNIEKITLVDFKELDFKALGKLRNLRILKLRRGSTGDGIHCVAGEFPALQMLQMKEVKVERWIQDEGAMPSLSRLIIKGGAVEDLPLQLQSLASIK

>arahy.Tifrunner.gnm2.ann1.4RY6A3.1

MEIDEAKFLCSYGGEINPRGHNNKLAYVGGTNKLLYVDRRIDFTAMVAKISSLFDGACNGHYFFKYQVPGSDDLNALVSVTNDRDLHNMMLVFDQLYRDSPRFPRMRLFLFPNPNKPLDSVKPDSNGLSSNLWKHDVFISCRGKDTLTGFTSYLVKALNKKHIKTFTGDELHKGDCISDSLTRAIDNSSLSIVILSENFVTSSWCVDELLKIMACGRKVIPVFYGVDPSDVRKQLVSFNEKFKSHLQSNINNLLKWMDALAKVADLEGWDSSSCSKTYLLHLLYSYCRDDFELVDKIVKDVLRKLNNPCLPKDIKGVVGINKNLEKLELLLSSVPDEQTGIIGICGMAGVGKTTVVKKLFEKHSYQYEGSCFLEGVLERLEKYGQEIDELCNQLHSKLLEGKDFPNSMADTNSAKCCLCSRQRSFIVLDDVGSSKQLEYLVGDLQCYGAESRIIVITRNKSVLEKRVEKIYKMEVLDFQDSLTLFSLNAFNQKYPKTGYQELSWKAVTYCKGVPLALKVLGSFLHSKSETEWDSALQKLEKNPNASIQNVSRLSYDGSDYQQNHIVLDIATSFKGEQKEQRIDVLISSLLDEALLAIYDHFELLQDLNKKWGGESFVKNLSRILKIIAICGVLVIFVMFWKTARYQT

>arahy.Tifrunner.gnm2.ann1.4VC79G.1

VESYTQQIPLSTVILNDIILISHQKAGTDQFRVRGHLFPLNQAEDINRIVESITALLDNTILFVAQHPVGVESRVQHVINLLNSNRSDNVLILGILGIGGIGKTTIAKAIYNQVHGCFEGYCFLPNIREFWMQSTSQVHLQEQLLSNIFKTTKIRIHNIDSGKIILQQRLCGKKLFLILDDVDELDQLNALCGSREWFGPGSRIIITTRNEHLLKVLQVDHISRMSNMDHHESIEHFSWHAFKQKCPKREFLELSKKVVAYCGGLPLAHEVIGSLLFDRMKKVWESVLEKLTRIPNHEVQKKLRISFDSLVDDTIKEIFLDIAFFFIGMNRNDVIHILDEYNAEIGIDVLIDRSLVTVDNNNKLGMHDLLRDMGREIVREKYPKEPEKRSRLWLQKEVIEVLQTHIGTRATEGLVLKLTRTNTICLEAKAFKEMKKLRLLQLVGVLGRDFEYISRDLRWLYWHGFPLKYIPPNFSQEICLKLVWKEPQFLEKLKILNLSHSQNLRQTPNFSYLPNLEKLLLKDCSSLSTISDTIGHLKKILVINLEDCISLGNLPRSFYKLKSLTTLIISGCSMIDKLEEDLEQMESLITLIADKTAITQVPFSIVRSKSIGYVSLCGYEGFSRDVFPSLIWSWMSPTNNLSSLVSAPELVSFLDLPKLQYLQVECNSDLQLAEGVTKILEILYATYYNELEAIPTTSQVLNIETSTIIDFRSEFQINGLVKSLLIEVGAKSQVSGILKERILQKLNTNDHDRQSVLPGDNYPDWLSFNSEGSSIHFKVPHINGRDLKTMMLCVVYSSSQDTISSRCLQNVLIINYTKYTIQVYKRDTLASLQNEDWQCIISTLEPGNIVEVLVLVYGFEFVVKNTTIYLIYSESTNEEIEHSNKTACIYGRAHFFLVPQSSDLSSSHQLFHKPKNNHGYITDQNFGVFIASSLPLQFLHRYLWSTTRYHI

>arahy.Tifrunner.gnm2.ann1.4XA800.1

MVDGSLFSGGAVGAGMQEVLRLAIEMVEKGKNFKSVLKTKKETLDALTPLVEEMKQYARELDRGTDTVASLEKEINKGKEIVEKCSKFRWWRFPSFPLYHDRIEGRNKKLGRILSVNVQTQIGRDVMEVLYNVRNILDIIRDDYGGRLGEEKVLRGVSGVPEKPEFTVGLEEPLKKLKVEVLKKHGDRVLLVTGLGGSGKSTLAKKLCWDEQVKGSFFYHF

>arahy.Tifrunner.gnm2.ann1.4Y5JAV.1

MECKKEFGRLVIPVFYIVDPSHVGYQLGSYNEAFKKHLQNNKKAEVQKWREALTAVANLEGLDSRSYRDEIEFIQNIVKDVFQKLINHYPPNDSKSLVGISENLEKVESLLSESVKVRMIGICGIGGIGKTTLARLIFEKYSYTFEGFYFLENVRERSRNYGLTELRRRLYLELLQEKFWQNNTGKSTFVEDRVSKQKNFIVLDDVSSLQQLDYLVQKLQWCGAGSKLIITARDKNMLVPTVETIYEMKILDSHKSFKLFSLNAFNEDYPQIGYEELSWKAVGCCKGIPLALIALGSFLHSKSKTEWHSALQKLEKTLDPEIQNILRLSYDGLDDEAKQIFLDIAYFLRESFCGLYAAIGIRSLLDRALIAISHNCVRMHDLIQELGWDIVCQQSSENPENRSHLWDSNDIRDVLGYNKRYSLVEFLEAIIVFDMSQIEDLQLNADTFKKMPKLRFLKLYIPSESDGRLNKLQLPVGLKPFPSKLRYLEWDAYPLPSLPLNFCPKKLVTLCIRNSKLKRLWDGVQNLVNLEEVDLTDSKKLVELPDFSMADNLKSVHLSSCRSLRHVHPSLLSLGKLELLDLLNCAKREMLETKMHSKSFKHLYIKSRIKITSRWKPCEVPVSIKHLTELKTLSLKGCKMLQHLPELPSSIHHLTALDCIMLQTVTFSSNIPRLQEEKCINISFHNCIKLDVANCIYWYLKGIRKLAYVCESRRGGKGVVRRSDFFKVCYPDYRVPEWFMHRTKGTSITFEVSSPSSYGFSSLLCVVLPKYSLDYELDIKCRCYLEDGSNMHKYLFGILFLNHIPAEGCSDHVYMAYNYGGIFDVIKLDRLNNKIASSR

>arahy.Tifrunner.gnm2.ann1.4ZIH53.1

MDCMRIESPLSVEKNWKYDVFVSFRGETRFNFTDHLYAALLRHGILAFRDDTRLQKGGPISAGLQQAIEGSQILIVVFSINYASSTWCLRELAQIADCIQIPGHTVLPVFYDVSPTEVRRQSGNYEAAFIKHEQRFKDDAEMTEQVRRWRAALTQVANLSGWDMRNKSQSANIEEIVKAVTSILSPKPSSSLSNDIVGMQSPLEELKKILALGLDDDVRVVGICGMGGIGKSTLATILYEEISHQYDVSCFVDDVSETYRHYGPLGVQKQLLCQAFKEENFPICNLSLANNLIQTRLRHRKVFIVLDNVEKGIQLEKLAIKREWVGPGSRIIIVSRDEHILREYGVDGVYKVQLLNDENALQLFCRKAFKCNHVAQDYRRLTNSALTYASGLPLAIRVLGSFLFGRNVSEWSSALVRLKENPTKDILDVLRISYDGLEEMEKEIFLDIACFFPSREWDLEDILSIRGFQPDIGLRILIDKSLITNVGNMICMHDLLRDLGRSIVREKSPKEPRKWSRLWNQKDLSDVLRENKAAENLEAIVLPRFHQNREEMSERTTLRVEALSQMRHLKLLILREANFLGCPNFLSNELGYLDWKDYPFTCLPSSFQPNKLVKLILHYSNIKELWEGIKDLHNLTYLELCHSKNLVKIPNLSHAPNLEYINLKGCVKLVYLDASIGSLEKLLHLNLENCKNLVSIPNSIFHLNSLRNLNLSGCWKLLKYQLLEKPRESKQLNTGQSVQSHMTTSICKTLMRPLNFLSSRRRSNSVGLLVPTLSRFPALVFLDISFCNLVQIPDAIGQLRCLQWLNIGGNNFVTPPDCIKELPKLRVLNLEYCKHLKLLPSTFMPIGGSSGRYYYGGIYVFNCPNLSDTEGCRLTVPTSIPPFCVYYELILLIVNLQCRIQAVVPGTKIPRWFSKQKTGDSVSLDPSPIVDDNNWIGIAVCVTFVVHHTPTQLLEEAIYPPALIACGFRRKGYRGSYPHVPIRLKNDLITAELDHMLLIFVSREDFLDENTRIAKEGTSDLHGITLHTISDYPEEVVELKRCGYRWVFKEDLEQFNPTMMFSANSSAQSPNHKFLAIQDHQ

>arahy.Tifrunner.gnm2.ann1.4ZR0FI.1

MAAELVGGAFLSSFLNVIFDRLSDPEIINMMKGKKVDQKLLQKLENILSVVEAVLNDAERKQIYNPAVKRWLENLQDAVYDADDLLDEIATKAATRKDPPPGNFLFRFLNLQDREMVTRIEEIIARLQDIASHKDILGLEKISVKNMSGRIESTSLVQKSDVFVGRDQEREDIVKLLLDDSNDGKLSVIPIWGMGGIGKTTLAQLVFNDDRVQQKFDVKTWVCVGEEFDVLKVTKTVVEKATSGPCNLNDLDSVQLWLKNELAEKSFLVVLDDMWSNNYMAWKKLLTPFQCGTEAGRHGGKILVTTRDEKIANMVKSHHHQAHNLSVLNDEDCWSLFANLALLSKDRLGFEKVGREIIKKCKGLPLAIQTLGSLLSTKDDERDWNDILNNEIWDFSEEESEILPALRISYYQLPSYLKRCFVYCSLYPKDYEFDRDELILLWMAEGLLQQPRSGSTLEEVGYECFNDLASRSFLQPSNNAHKNSFVMHDLMHDLATFYGGKFYFRTFELKNVFKHDTKTRHLSYGLYNDSISKIVEICDSLKHARTLMQVNLDTYEFSKGVIDPCHLLEQLKCLRVLSFKSFYLEEDLLHHSIGELIHLRYLDLSQTLLVSLPESLCDLYNLQTLKLSKCKKLKKLPSNMQKLVNLHHLDIEDTELEEMPKGMGKLKDLQILRYYIVGKHEENGAGELAELVNLRGSFHIEKLENVINSSEAWKARMVDKKYMSDLCLEWSSGEDSDMVDSQMEKDVLAKLEPHKDLKELRIKGYRGTMFPEWVGQSLYHNMTELELSGCGNCWVLPTLGQLPALEWLVISNFDKVRRIGGEFYKDDGTDHHREIPFRSLKTLIIRRMPCWEEWESFESDDNDAPFPQLEHLRIWECPQLRGDLPTFLPSLKELWISGCEQLGCYLPRAPIIRKLRISGKQEARMQDLPLSTLEQLSICGEQQVEYVFDVMTHTQPTSLTWLEISNCSSAISFPRDSLPPSLQYLSIKNCMNVEFPMRHQQHHSLQSLRTSTDSSGTREFDAFGCCPSNFCKDPPFSQDCKKQIGSRWSKQNSWKTSSSYKIKRRIG

>arahy.Tifrunner.gnm2.ann1.53T7SD.2

MHVAMLQYNELTRSVSVEGTWALIHCSWMLDELYQTAYASKMHIVMANPVEEIETGSETISNSSSSYDVFLSFKGEDTRYAFTGHLYYALCRKGIKTFMDSEDLRVGETTRPQLFQAIEESKVSIVVFSENYADSTWCLDELAKILECQGEKGQLVFPIFYKIEPSDVRHQRNSYRLAMAAHENKFGCYSEKVQKWKSALLGVSNITGYHLKEGFEFKFIQDIVCKAATKISPKQIPLEENVVGLQFRVAELKSLLDIESNKNTFMLGIIGTGGIGKTTLAKVFYNSICNEFEGACFISNVRKASNQDKGRIFLQQKILSDALGVQKIKLDSVEKGIHTIKANLSTKRVLIVLDDVDKIEQLKELAGGCDWFGLGSRIIVTTRDKHLLVAHQIRRIYEMEMMNDRESLELFCQNAFKMSTPARNYEDLSNRAIRYGKGLPLALKVIGSELIGKDLQVWESSLDKYEKNPHGDIQNILRISYDSLERNEKEIFLDIACFFNGQRLKYVKRILDGCDFYTDSGIKILVDKSLITIENGYLRMHDLTQDMGREIVKQEAPKESGERSRLWLCKDVLEVLTENRENSKTEGMKLDCYGEVNCCDDTLEKMMKLRVLIVHNASFSSKRIRLPNTLRLVDWKGYPSTSFPAAGFNPSKIAAFNLTCSSLVLKKPFQKFDQLTYMNFSYCQSITQFPSVSGAPNLRDLVLDGCKQLVKIHNSVGFLSKLVYLSASDCTQLRSFLPEIFLPSLEYLSFDLCKRLTHLPRLRENMDKLLKISVIHTAIRELPLSFASSLCGLEYLDMTNCKQLQCVFTGWFPKLRTLKIGGCSQLRKSLNVDTFFLLEYRLMSLHLSNASLSDDDLEKIMQMFPYLKDLIVSSNCFEKIPFKHSFFWTSLDVSYCLSLKYVLALPSSIQKVDARHCNSLRTYSSSTLWSQMRKEIKGLQIVMPKTQLPKWLDYCDSGGIPFLWARGRFPVMALVLEFGKMDYQDISLHLFIDDEHIYSQRPQRHSFALAEDHVLLCDLRLLFSDEEWKRLDVRLGHDNDWKAVQVKCEAGLNLRQWGVYVYKNETNMDDIQFPCPYYHEEEEEKSFLEAVTTSPSDEMEEETLAAVSSRKEEWSSPELSDDDSNHDNRSPGTCSLCCSALLSSFKKRLCCCKVRRARSISWWIVLWNQWRRRQRRLREEERNWEEERKMERDMRRQKESMANEAMQRDMEELLENIMVEEDQLKKERSETCESQREGRERGQIEEKEDMEEEESKRWRDRRQTIEETWIEERQQKAEKETCLSQREDIKMEEIEEVEDVEEDKSKRWREGKQKMEETWIEERRQKEVVEETWMGEFDWSVMKMEVETKRRREKMREKEEEWREKQLDVTRAISSHHAPFSPNLQIVVQNLKRLITVGEDSGLDNNHDYSSEWFPDRTILRHHDGEVEG

>arahy.Tifrunner.gnm2.ann1.558TLZ.2

MTCMAFTLLAMAHPAEISSPSFTGEWEYDVFLSFRGKDTRHNFTGNLYHSLDQKGIHTFIDDEDLRKGEEIKPSLLKAIQESRTAIVVFSENYASSTYCLDELVAILEHLKAEGRLVWPIFYDVDPSVVRHQRGSYAEALAKHEERYQDDKGKVKKWRNALLEAANLSGWHFIQGSQTEYKFITNIISTVSKKLNHTPLHVADNPVGLGSPVLQVKSIMEIGSNERVKMIGICGTGGIGKTTIARAVYNLLADQFDGLCFLADIREMTIDKHGLVQLQETLLSEILGEKDIKIGCVNKGIPLIKRRLQRKKVLLVLDDVDNLKQLKAIAGGNDWFGFGSRIIITSRDKHLLSAHGVSEFYEVKELSKRDALELFSWNAFKSNKVDPGYANISSRVLLYAGGLPLALQVIGSNLYNRGIDEWKSALDKYERIPNKEVSYDGLEEDEKNIFLDIACFFNKYKMSYVRGMLHAHGFHPEHGIRVLVDKSLVSIDVNDCLRMHDLIQDMGREIVRQESTSEPGKRSRLWFDEDIFHVLQNETVCWILLDFVFLFFSDNVLIWLCLVLEKGTDKVEVIILNSSRQKEVCWNGNAFQKMRNLRVLIVGDAYFSTGPKHLPNSLRVLDWVGYPSTYLPSGFNPTELSILSMISSCLKFDKPLKVFCSLSFMNFEDCKLLRQVPVLFGLPNLRALCLDNCTNLVKVHDSVGFLKKLILLSAQGCSKLRSFVSAINLPSLETLDLRWCTSLENFPEVLGQMENLKEVYLDHTAIKELPFSIHRLIGLERLFLRRCKNINLLPVSISLLPKLEVLIDYKGRGYRLYEPHKAEEALMSKVFVSPSLEVDKGYLLPFDKAYCITMIDYNVIQEYPLTGIVLPEKHVHFGSGSSVSFWFRERFPEVTVLLIAEPSLYIDIMHSSVCEFRYSVVINGMNQFSSSCDYIISERRKLCLTIFLGDLQRLIEEGAVKRVFSDSKDERNHVEVSCELKYHMPYDPKHVSEGQNRSIEGTIKQSTVHGDVYHTLLTYPSPYCKNNFLCYYQSKALQKHIREQKLAQHVKPIKACSLLINSISFCFLTFLQCRSVFHICYFANL

>arahy.Tifrunner.gnm2.ann1.57U25Z.2

MAMKSDEVRIVGIWGMGGIGKTTIARAIFDRYASRYEGCCFLENVREKSQKSGEHSLYEKLISELLEGEHLLVKGSAHARSMNVKRRLSRKKVLIVLDDVDALDKLDYLTREPICLGAGSRVIVTTRDEQILIAKGVDKRYKVWGLSFESSLELFCLKAFHKSCPKHGYEELSEMAVNYAEGIPLALKVLGSFLHSRSIKEWESALKKLRVHSNVDIYNVLKLSYDGLDDSDKNIFLDIAFFLRGEYKDNVIRFLDCCGFFGDIGISTLQRKSLITIFSDRIEMYDLIQQMGWEVVRQESNKDPGKLTRINKPEDFCNLLKNSEEKSLVEGIMIDLSQIGDLHLNADTFKNMPRLRFLKLYAPSDQRQSKVYIPTTLDPFPAKRSYLCPLNSLPLRSCAEKLVELRMTNSQTSKLWDGPQNLVNLTELDLSGCIKLVELPDLSKATKLKRSNLLCCENLCQLHPSILSIQTLEGLFLDGCKKIEECQKQHIFEIS

>arahy.Tifrunner.gnm2.ann1.5F1M32.1

MAEAVVTVLLENLSSLFQKELGSIIGVDEELKKLSSTFTAIRAVLADAEMKQFTDLAIKDWLRKVQDAALLLDDILDEFSTHDLQNQNQQQKGGWFNKIHASCHSSFGLKNAMFRRRMANKMKRMRETLQNIAEERKMFHLCERIATTDKKAELMEWRQTSSVITQPEIYGREEDKERVVQALIQTCDTERAAVHTIVGLGGLGKTTLAQLVFNDDRITMHFDLKIWVCVSEDFSLKRMIKAILDSASVSVSGNLDIDPLQKKLQQALQGKRYLLVLDDIWSEDQEKWDRLKNSLVACGSKVGSSVLVTTRLTKVASIMGTLPPHDLSFLSDEDCWSLFKQRAFGLDEGENVELVAIGKEIVKKCKGLPLAAKSLGGLLRFKREEREWLYVKDNEIWNLPQDENSILPALRLSYLNLPLKSRQCFSFCAIFPKDAEIEKDELVHLWMANGFVSSEGTMAVEDVAEEIISELCWASFFQDIEKDDLGKVRGFKIHDLLHDLAQYVMGDLCCIVNDEMPIEHPKRIRHLTMGPCARNQIISACTFRSLRTFHFRQTNCALASTKILGPLESLRAFDLGYANVLNMLHLPLITPFNHLRYLNLRGTSIQTLPESTSSLCNLQVLNLSYCQSLRRLPRHLKYLKALRHLYLRGCRSLVYMPNEIGQLNSLRTLNIYIVGKKKGLRLDELAQLCLRGELHIKNLERVTNAADAKEANLMGKQLDVLKLSWGRNEQSKLLQNVDQILEALEPHPKLIDLGVGGYQGMYFPQWMGNPHLKNLCSIKLVDCWYSQQLPLLGRLPSLKILAICCMHNLRYVDDESYDGGVARGFQSLMCLELCCMPNLEGLSKEEGGDMFPGLSKMNVIACPRLTFPALPSVRKLCIAKGRRRSGHDLFSREILLSQCPSTTRSPNQVQMDSIHSLSCLEILEIHDDQELASFPKGTLQGLCRLRQLHIHHQSKMEVLPLELANNAALQELHIIGCHNLESLTDQVLTGLQSLQKLKIFKCDKFKGLSTGFQSLSSLKDLIISFCPQMVALAEDLQHMPPMLQSITLHELKNLECLPYCWGSTMTLQSLYIACCPKLKSLPISVGWFNGLNSLTVVHCPSIQKQFEKETGKDWKWIKHIPNVDLGENCWVIPTIAASYPDLTFSNWEGLLLQTSKQTMHFVLDRIYGDVISDDRSFS

>arahy.Tifrunner.gnm2.ann1.5JI1RR.1

MAAKPYGGAYLSPLVEVVLDNLSSMFEDDSVLNGNHSALELLGRLQNCLYNVGPALDDAELKQFSDKKVKEWLVDLQDALYMADDLLDEISTKAAIAATKRNAGNSSSWSHLVDSYIEDTGDIEKIVRRLETVVAGKISLPLKEVAKLDMSWRIPSTFLVEPPEICGRKEDKEAILKLLLDDDDAADVDLSVIPIVGMGGIGKTTLAQLVYHDDKVKESFDFRGWVCVSEEFNVVKVTKTIIEAIVSWRYDLTDLNLLQHDLKEELSRKKFFIVLDDVWDKNYDDLNRFLKPFQKGVKGSKILITTRNKNVASVVQTISPHELSPLSDEDCWLVFSKHARLSTISLENPTLEKIGRDMVKRCDGLPLAAQALGGLLRGNSDIRSWNHLLKSEIWELSYDKTNVVPALRISYYFLPSYLKQCFIYCSLYPKNYEFSKDELILLWMAENFLQPVDKKTVEEVGGEYFDELIARSFFQPHNTHEKTFVMHNLVHDLAMTWAGEFYFRAKELRNAVEVDIKARHLSHNAKGNYPMSKLLGVCDTVKHTRTFLEINLETWIPFNIENATCILLSQLKYLRALSLKRFPLESVPDSIGELIHLRYLDISETKIVTLPESLGNLYNLQTLKLNTCLSLITLPVGMKDLVNLRHLDIYGTGFQYEMPKGMSNLKSLQFLSNFVVGKHEENKIKELGALADPHKSISIWKLENVVNSSEALEARMCDKDGIDSMVLGWSWHRENRVDSEMERDILDKLRPHTNLKELQIESYRGTRFPDWVGHSSYHNITKITLDGCRSCCMLPSFGQLPSLKHLSISHFKSLESVGAEFYFNQNGESCLETPPFPMLETLKFYLMPYWKEWRSLEFNAFPRLREFSIQRCPMLIRDLPNHLPSLQSLTIETSGQLRCCVPKAPAMTSLSIFVYGNEVRIRELPPLLRQLSIGGIGQVEPGVKAIMHMQLSCLTSLCISDSSSHILFPVSAIPPSLQELTIENCGESFEFQMDGQHHSLQKLSIGNSCDSHTSFSLLDAFPNLVSVHISGCQKMESLVVSRSLSCLLSLYIFFCRSLKSVSTLWMAAPQLEKLSLVDCREIDLCDTGHPHRSLRYLEIIYSEKLVSSAAFMHPQFHGITSLIFYCEIADYSECVKSFPKEATLA

>arahy.Tifrunner.gnm2.ann1.5JTS7G.1

MAAKLEGGAYLSSFVDAVLKKLSSLDVNSTPMARKLADQDLLQRLKLSLRSVRPVLDDAEQKLIRNDQEVKKWLLDLQDALYMADDLLDELSTKAATLTPTQRDPGNYSSMCHSIVDSILEDSDDDDYEMGVADLVDKLESIVEEKNGLGLKDEPVRDPEDMSWRIESSLVESSEIFGRDNDKEAIIKLLLDDTCYAKISVIPIVGMGGVGKTTLAQLVYNDERVDEKFDIKAWVCVSDQFDIVKVTKTLIEAAGGSSYNGNSLDLLQTELKDKLMGKKFLIVLDDVWIDNSYSRQWKTLQKPFQFGKKGSKVLVTTRNDTTADVVKTISAYKLSLLSEKDCWSLFLKCVFLSSESKEYSTLEPVGRELVKKCKGLPLAVESLGALLRTNYDEREWDSVLKSELWEVFEDQNDEIIPALSMSYYYLPSTLKRCFVHCSLFPKNYLFVKHELILLWIAEELLQPKGKKDLEEIGSEYFDQLVARSFLQSSSTYNSFYVIHDLMHDLATTHAGEFYFRAEKLDEHIRINNKTRHLSRNARGNYPFAQIVAACGRVKDARTLLEISFCQEYPFVAPSTKGKNFLHDFLSKLKCLRVLSFNAFPLYSLPDSIGNLIHLRYLDLSNTTTEILPEELCCLYNLQTLKLKNCRLLKKLPCNMQDLVNLRHLDIDGAGLEEMPKGMSKLKDLQFLSDYIVGKHADNNQIKELGALANIQESLHVCQLENIEDGAQALEARIADKKHIRVLYLTWSSNSHSHIDDYQDILNNLRPHTNLRELSIKEYKGLTFPNWLGHSSYKSMTKLYLYGCSNCLELPSLGHLPSLKCLRLSNFYKLQRIGAEFYKEDGSSSRKAAFPVLETLSFEGMDCWEEWLSVSSELDAFPQLRELTVRRCPALRGDLPIQLLALQSLCIDQCGQLDFSLPRADALLELSVEGPQKVEAVLKAIAGNQLNCLQSLSITNCCSATSFLVGSMPSSLQHLVINGCPYIELPVLQQQHESLQSISISNSCHSLTSFPLASFPNLTTLRISSCINLRSLLASDPTSASTSASSLHSLTIESCPSLGSFPTLEMVAPHLEYLLLRECPEIESFFEGGLPPNLRELKIQHCEKLVKYLASMDLCDHCLTCLDIRHPYDNIKSFPMGGSLPASLETLNLRSFPCLEILDCKGLDHLQRLLIDYCPRLDVAGESFPASLVNAPLKWTFFAEETVKVTSGSKNFFLIEDKRRALPMYAALKLRGDGFDVTTSSTEVHRLTYNVYNDERVDEKFDIKAWVCVSDQFDIVKVTKTLIEAAGGSSYNGNALDLLQTELKDKLMRKKFLIVLDDVWIDNSYSRQWKTLQKPFQFGKEGSKVLVTTRNDTTADVVKTVPAYKLRLLSDTDCWSLFVKCVFLSPESKEYSTLEPVGRELVKKCKGLPLAVESLGALLRTNYDEEDWDRVLQSELWEVFEDQNDEIIPALNMSYHYLPSSLKRCFVYCSLFPKDCPFKKDELVLLWMAEELLQPNEKKDLEEIGSEYFDQLVARSFFHSSSTYNNFYVIHDLMHDLATARAGEFYFRAEKLEENVGISNKTRHLLHDAGGNYPFSQLVSACDRVKDTRTFFEINLSRKDPFNMENAPHVFLSELKCLRVLSFNTFPLDSLPDSIGNLIHLRYLDLSDTWVETLPEELCCLYNLQTLKLNNCQHLKKLPSNMQDLINLRHLDIRGASIEEMPKGMSKLKDLHFLSDYIVGKHADNNGVKELGALANIHESLHIHQLENILDGAQALEARIADKKHLKGLYLTWSYSHSYIDDSQDILNNLKPHRNLRKLSINEYKDFVEILIVLQIHFITNSTNTIHLSTMTLWKYSLFCRYTLPSYSKLLSPMPLCTS

>arahy.Tifrunner.gnm2.ann1.5L14TG.1

MANRASTSVIMATNYDVFLSFRGEETRHGFTGHLYDALCRNGINTFIDDENLRTGETIRPQLLQSIEASKISIIVFSTNYAASTWCLDELVQILRCHRERYQLVFPVFYKVEPSDVRHQRNTYKEAVDAHEIRFGCHSQKVKEWKEALAETSNMKGFHLKQGYEFKFIQEIVCKALTHIPPRQLLIEDRMIGLQTRVVEVESHLYSSYSTSKYRILSNIKSSKPKLYNNNTMLGIVGIGGSGKTTLAKALYNSICDRFECACFLFNVRKISDQEEGLVRLQQTLLSKLLGEWEIKVRSVEEGISMIKEKLGKKRALIVLDDVDKIEQLKALAGECDWFSYGTRIVITRDKYLLTAHKVEKIYKMKLLSDPESLELFCWNAFKMTRPKANYEDLSNQAIHYAQGLPLALKVIGSNLINKSLEEWKSALDKYEKNPPKDIQSVLRVSYDSLEGNEKDIFLDIACFFNGKKWEYVKNVLDGCGMFTEDGIRILVDKSLVTIKDGYLRMHDLIQNMGREIVKQEAPKEVRDRSRLWFHEDVLELLPDDKENKKIEGIKLVQCEEHDWTDNAFEKMKKLRILILRNTNLSCRTIPLPEQLRLLDWKGYPSNSIPSNLKKIVALSLRHSPLMLEKPFQNFEHLTYMNFSHCESITHFPNVSEAQCLRKLILNGCINLVRFDESVGFLPNLTYLRASKCTKLRKFLSRICLPSLEHLSFNWCRILGLFPDIVGKMDKPLKICLKATAIQELPDSFVDLVGLRYLDLTSCEKLGYLPSSLFMLPNFVTLKVGGCPQLGRSFARFRGSLPTTAESRPSLETLHFSHASLRDEDLHVIMQSFPNLEVLDVSSNNFVSIPACIHESSYLTSLDLSYCLNLQEIPKLPSSVRKVDVRHCNSLSASTTSMLWSQVCEEIYKLQVVMPTSNTQIPEWWDHCTRWKHNPSYLNLKARGKFPAVALAFVFGEMNYQSVGLHLSIDYGDVNSAYQPSHNFRVAENHVLLCDLRLWFSDEEWKRLDAHVEHGNKWKTVKVRCVPDIIPVHWGVYVYKEETSMEDIQFQERELTYQSCPDRSWKKRLSSAEKLAIASFSESLQTVVKNLKRLMAPREEEQCFCLMQHDRDKDKDEDEDEEEEGESDIEA

>arahy.Tifrunner.gnm2.ann1.5M20JS.1

MDLQKLNLDNTNETENSFVKDQNYAHIESLLRTQQREVLVIGVWGMGGIGKTTIAAAIFEEFSPKYEVSCFLANVREESSKHGVKYIFNKLLCELLQEDIHIDTPTIISSTIMSRLRHKKALIVLDDVNSSDLLDNVLGVGHDYLGVGSRVIVTTRDRHVLTCRAVDQILEVKEMNYHNSLKLFSLNAFNQIHPPENGFQELSKRAVAYAKGNPLALKVLGSFLRSKSENEWDSALTKLKGTPDANVHKVLRLSFDELDDAEKNIFLDIACFFKGEEKYKVIMILNACGFHANIGIRNLLDKTLITITKMKRIQMHDLIQEMGQEIVCEESAKIPGGQSRLWNPEEVCDILKNDKGTDAIESIFLDMTQTTDLYISSNAFRKMPNLRLLAFADSIGHGERRTNNNLYLPINLELPNSLRYIQWDGYPLKSLPSIGWTKNLVEISMPYSNVEKLWDGVQNLPSLEIIDLRGSKRLIECPNFSATPNLKEVWFNFCESLTHVHPSIFSLEKLEFLAVYGCKELKSLCSSNCSPSLHTVVAYDCPNLQEFSVPILHQDSKIHLHLRSTPLKELPPSILQLKHLVNFSFPISKNLAKLPANFANQIMLSDISEHEQDAVFTLHSVLPSPVFKHVKLLKFDNCHSLTELPDNISLLSSLGKLSLHNTNVIGLPESIKFLPQLKVLKVCHCEMLQFIPVFPPSIESLQVWDCKSLKTTLSLESETPKQPAGTFIFLDCMNLDENSCNAIMKDAIARTKCWMETILTTESEVSEEQRDNEDHNVINFGKICCFFPIRGSILQEFFHDYSAQASISTQVPPSYNLCGFMFSLVLFEAHSCIIDELVINFECECYLETSWGESIHVASSVVVEWGCDMTIGYHLNVMPDHVLLWYDEEYSKQIMETVKGREANTEKKSHFNAKMTVKFVARLPNKEEAMVKECGIRWIYSNLEEGSSREQRSKRIVGS

>arahy.Tifrunner.gnm2.ann1.5MQ2SU.1

MMLYGGTSSYGRGWSYDVFLSFRGEDTRRSFTGSLYHSLHKKGINVFIDDDKLRRGEEISPALLRAIEESRISIIVFSQNYASSKWCLDELVKILECMKTRGQLVFPVFFNVDASMVRYQKDSFGRAMSAHEVRYKGNEEKLQKWKQALFEAANLSGWSFKNGYEYELIEKIAEEVSKKINQTPLHIAEYPVGLETRISEVKTLLQIGPGEDIRVIGIYGLGGIGKTTIARALYNFIADQFEAASFLADIRESSNQRQGLVQLQESLLFDIVGDKNIKLGNIYKGIPIIKKRLCCKKVLLILDDVDKLEQLQALAGGRDWFGFGSVIIITTRDKHLLAAHQVDKTYEVKKLNHGEAFELFIWNAFKRKEPEAGYMEISNRVVLHAEGLPLALKVMGSNLIGKSVDEWTSALEKYEKIPNKEVQNVLRVTYDNLEENEKEIFLDIACFFKGETMEYVEKTLKACGFHPKFGIGVLIDRSLVSIDEYNRLRMHDLIQDMGREIVREDSPLEPGKRSRIWYHEDVIEILTESTGTYKIQGMMVDLPDQYMVHLKNDSFKKMKNLKILIVRNGQFFGSPQDLPNNLRLLDWMEYPSSSFPSSFLPKKLAVLNLSRSRFAMQEPFKHLDTLTSLDLSYCEVLTKLPDFSGVPNLTELNLDYCTNLAEVHDSVGFLEKLVELRAYGCTKLKVFPSSIKMTSLQSLILNWCSSLQSFPSILGKMDNLMSISIEGTGIEQLPPSIGNLVGLQEFSMTSCLSLKELPESFDMLQNLRNLDIEGCPQFRSFLMKLRESGKSSPTFDNVKSLNLENCGLRDEDLPTIFYCFPKLSLLVLSGNYFVTLPSCIQEIQSLELLHLDSCNLLQEIPSIPPNIQYINARNCTSLSPRSSNLLLSQEIFEACELQVMVPGRRVPEWFDYCAKGEYLTFWAHQKFPVLIFCFVIEVEGGATEDTLNCEVQFSINGEDIYEMEMPQCFSKMVTDHVWIYDLRTHPTIQWHSLDSYLVDDWNQLEISCEKISGSSNMSVSWRGVHICKQEVNMENILFTDPELDLDSNTYSEEIEADSNATSEESTKSVQDFSNNLNDNCCDFENTESCDTISRQEEEWGERSKGKENSETASHNDIDQSDKKILMHMQPLESVTEDPIAPVCLEVIDTVKVVDYGSNTIVVDHSEKVQPRSQVKSLVEIPTVDDVEMEAFYASLEAETSVLHLSNNTIDVSKFANRVPSEATQNALKTLQDFLTRDFSLLLGPDEYNAMKATLEYLTNLPPGDGISLELRSLVIEVSRHFNHWSLDYTNESKKIEAATAKLLKVDELEEGLEANKAHFREVVCLENELRNELEYLEERKKELEEQIHAIKASISASESARKMVTYRKREIFGEAKILKNQRDELKEQVSWLKDEKELARKIQTNIRAEWSKLGEKFNRKLKSEL

>arahy.Tifrunner.gnm2.ann1.5N0EYQ.1

MAEALLVGPLVSGSINLVLNRLISPEFVNSAVNKKLNRELVERLKTALLAAKALAADAEQKQFGNELVREWLDSLRDAVYTADDLLDRVFIKAQIRNKVCVPLPLRLNKSARKRVTKINEVVKRIEDLQKLKDSLGLKEIPTGSSSWKPPSTSLVKGKVYGRDGDQQALIQMLNDNNHHNLSVISIVGMGGVGKTTLAQCLYNNKDLMDGVDLKAWICVSENFDVVETTKNVIKGISSGVCSLDSFDLLQQDLKEKLSEKKFFIVLDDVWSEDAHKWNSFITPCQHGTKGSTILLTTRNENVGRIVQHYHSYTLKELSDDYCWSIFADNASFPESNGSSELEGIGIVERCDGLPFAAETLGRLLRSERRAEEWNQILSSDIWEFPMTYSKIVPALLISYYHLPAHSKRCFVYCSLYPKHYKLDKDELILLWMAEDLLQSPRRGQTLEEVGCECFDGLVSRLFFKQVENDDEKYFVMHDLMHDLATFLAGDLCCRFGEKEEVSILTRHLAYNHSIPEKTCSSSKMKSLRTLLCINDRSYIWEAPAMLLCDILSKNKYLRVLSFGRLNIFPDPIDKLIQLRYLDLSWSDIEVLPESICNLCNLQALKLKDCSRLTMLPNGMYKLVNLRHLNIRGTPLKEMPKEMGQLKQLHILSNYIVGKSEDNGIQELGGLLNLHASLEIVNLENVVDANQARNARIIDKKHIEELLLKWSSGDDTVSNTHSDEQDILQGLQPHRVLKVLEIDGYKGTILPDWMGHSLYQNMTSVSLKSCKNCCMLPSLGQLPSLKSLCIESFDELKRIGKEFYKNEGHQHSSPIAPFPSLERLQFHNMPCWEEWHLPDSEAFPQLKSLQITDCPMLKGDMVNQVLMRIVFSSSDVSKVRELEILDYEGLYKKMRLVGDSLTMSGFESAAEYAFKARIIHHLTSLQQIQISGCSSVVSLGDNCLPKSLQKLVILSCSKPEFPQQQQKCDLVDLQISESGDSLTSLSLDAFPNLQNLQIYKCSNLESVSMSEPPHATLQRLSINWCHKFVSFPEEGLATPNLTHLDVSRCSKLEALPRGMNSLLPNLESLDIRGCPNICRWPEGGLPPNLKELSVQVRGLSWLGNLDNLTRLTVSGRYSGRIIKSYPEVDSLPRLPSLATLHIQEFDEQETLECNELLRLTSLQQLHISYCKRLKNMEGENLPPSLLLLKIYDCGLLGEHCKNKHQQIWPKISHIPTIEVDHVQIF

>arahy.Tifrunner.gnm2.ann1.5VXY8C.1

MAEALVVGALVSGFANVVLDRLISSEFVNLVVGKKLDRKLVDRLKTAILAAKVLAADAEQKQFGNELVREWLDSLRDALYTADDLLDRVCTKAQIRSKVRTRLPHFLDLSSREMVTKIEEVVERIEDLEKRKDTLGLKEISTDSSSWRPPSTSLVKGNVFGRDGDQQALIQMLNDNNHHGLSVISIVGMGGVGKTTLAQWLYNNQDLMDGVDLKAWICVSENFDVVETTKDVIKGISSGVCSLDSFDLLQRDLKDKLSQKKFFVVMYGAKMLISGIVLSPLFNMGGLSDDYCWSIFAYNASFPESNGSLELEGIGRKIVERCDGLPLAAETLGHLLHSEHRVEEWNKILFNDIWEFPAANYLLRPPRKGETLEEVGCECFEDLASRLFFKQDTINGKYFVMHDLMHDLATFLAGDLYYKLIQLRYLDLSKSCIKVLPQSLCNLCNLQTLKLEDCAMLTMLPNGMYKLVNLRHLDIRGTRLKEMPKGMGKLKLLHILSYYIVGKSKDNGIQELGGILNLHASLEIRKLENVVDANQARNARIIDKKHIEELLLEWSLSSGDDMVSDAQTERDILHSLQPHSGLKKFRIEGYKGKIFSDWIGHSLYQNMTSVSLKSCKNCCMLPSLGQLPSLKSLSIKSFGELKSIGKEFYKNEGHQHSSPIAPFPSLETLEFDEMSCWEEWHLPDSEAFPQLNSLQIRECRMLKGDMVNQVLMRIVSSSSNVSKVRQLEIREGLHQKMTLDRDSLSIRIFKSVVSLGGNCLPKSLQTLKIRGRQIELFQQQHKYDLVDLRIYSSCDSLTSLSLDAFPNLENLEIIECSNLESVSMSEPPHAALQRLTISNCPQFVSFPQEGLATPNLTDLDVNGCSKLEALPRDMNSLLPNLQYLDIRGCPNICRLPDGGLPPNLKELVVGDCEEQERGLSWLGNLDNLTYLMIVGGIKSRIKSYPEVGWLPRLPSLTTLHIQDVFTLETLDCNELLRLTSLQQLHIENCWRLENMEGEKLPPSLLILEIDCCGFWIRIRWSCQKQREALRSALFPNSGSLLSSTFQPHHHVKNEQWDEENLDREGEDYYPSDAVHGYPDVDLRSPLMSTCGSSMFNSLCVKVRQTALYSKQTVGSAMVHPSQAASKGPIWKDLTEPGVKHALIVEIGIQILQQATTTHHDSCVDRLTYAFGCWKHLRVQPHHQFNSKFI

>arahy.Tifrunner.gnm2.ann1.5YD3B8.1

MAESLLQMVIENLSAFVQDELATLWGVHSQIQELSGNLAAIHAVLQDAEEKQIRERAVKLWLQKLSDAAHVLDDILDECSIESNRLHSDQCLTRLDPVTIMFRRDIGKRMKEMVDRFRQIDEERRRFELRGRVPERQQEDEAWRQTCSGITEHNIYGREQDTENILEFLSRSADSSNDLSVYPIVGMGGLGKTTLVQRVYNDKKVIEHFDLRIWVCVSTEFNTMRILESIVESTRGHNPNLSTLEAMKNKVQEVLLGKRYLLVLDDVWSTDKWEDLKSVLLCGGGTKGAAILVTTRVESVASVMGTCPAHHLSPLSEDDNWLLFKYHAFGSDKVERTELVAIGKEIVKKCGGSPLASKALGSLLRNKKEEIQWVNVLESKFWDILEDDAIIVRALKISYFHLKLSLRQCFAFCAIFPQDFRMEKEQLIHLWMANGLIKSKGKLEIEDAGNEAWEELCQRSFFQEVEIDELGRTTFKMHDLFHELAQSIMGEECRVYDESASLTNLSTRVHHVTCLKPEREVNMDPFKKAESLRSMINLYPLDDHRNLNGLPPFNSLRALRTNASQLSELKSLTHLRYLNLRSSGITTLPECVSRLQKLQILKLERCENLSCLPKHLTQLKDLRHLLIEYCHSLVEMPPNIGELKCLRTLNLFIVDKKEGRGLSELRDLQLGGKLRIKGLENVINEGDARDANLSAKKKLENLYLSWGSSDSRRGANAERILEALEPPSNLKSFGMNGYSGVELPSWMQNTSILSSLVMVILYDCKNCKHLPPLGKLPHLTVLYVSGMKDVKYIDEDSYDGVDEKAFKSLKDLTLSKLPNLEGMLGDERVEMLPLLSKLKVSCVPKIKLPLLPSLEHIWIKGTGSDSDHSDSEGMASILEAIGQNMQHVKTLCISDFPKLKALPHELSSLSSLQKLEIYGGDELESFSENVLQGLCSLQSLTIHSCKKLRSLSEGMGHLTRLESLDIMICPKLVTLPSNMNKLVSLRGVLIACCDTLPEGLQHVPSLQSLEVYESNSIPEWLGEITSLQKLELTRVRLRSLPSSFRNLTSLRELSIDGCHKELQKRCTRVTGQDWQAIAHIPQFKLIPIHEETFSDKIRSKWRSWQLRRDRHRGRHHFAKDDRFDILVERLFYWYKM

>arahy.Tifrunner.gnm2.ann1.60VTT9.1

MGNNRDTVIIAGAKVLENAESHHEVVNRRLWPRASEISHNVQNFIEDAEKKQVKHQEVKSWFQGVKDLCYELIDVSEEFELVQQAKRLRFVGLPLLQQQRKVTRIIREFETLIKEVRELKLSSSSELPVPEAELEPVNLHSNIIGKELLVGRDAEIQGLIRRLTKGYHRCICIVGDEVGTGKTALARSVYNSTQVKSKFHFMVWVTVSRQFNVKRIVKTMLEFAPEKPEYVGDEFELLELELHKFIVDRELLLVLDDVVKLDSNHLSDLMNVFRSSSCRILITTRKKEVAKVAKEVEESTHTVLMTSLSPEACWSIIKHHAFGDDLKNESTVERIFGQVGRQIAEKCEGKPVVAKSFGVMLRGRSYEEWHHVMMSEKLWWYDLTSASMLSEYLYSRMPPALRQCLLYCSIFPKNHSIQVDKLVKLWMAQGFIASHEEDKMEIQGWKYVKQLRDCSAFQEFEPDGEGAFVCKLEEGMHEFIQDLARNEYCIKFLDEGATVEESGDNDTLTKPPRFRHCTLCLEAQTSFPDSIDNAGKLHTLMVLSESSDIDPTNLASLLHSLKRIRALDLRSCAIKELPLKAAELLHLRYLNLSFNHELKKLPSAISNLLNLQTLNLNGCDSLQKLPKSIGKLIKLRHLEILWTASLSYLPKGIASLTLLRTLNRFFGSSGGCLTIDGLGGETEISEAKRAGLKNKKNLRGLELWFSIVGLKGDNQVLLDSLEAPPQLQFLGIFYYRGSSFPNWMIELNVLKHLMLVNCSECNVLPPLGKLPLLESLEIKNMPNVEKVDFQFLGIGLNHEDAGNKGSSSDAIIAFPRLRKLHFMKLDKWEEWTGLNVNGGDKNIMPLLSSLSVVNCEKLESLPDYIKSKENLKPLIKSSLVCSKSATTEESPKNVAQMCVFNFSALYIKKALKKLEIEGHRTHCQFDTVVVPLIPQLPSTQPSRWPLSSGDWTLLKLQAYSFNFSRPSIELSLITPFHKACSSAATTQRVVAVYWIGTPTDATKLQVEEGFNSGMNHDK

>arahy.Tifrunner.gnm2.ann1.61LYXS.1

MNSLYIEAVSITCTLFPTAICTHQSKTNLLSVFLSSFVAFLKMAEALLGIVLENLIPFVQTQFAAFSGIKEKAEDLSRTLQLIKAVLDDAEQKQWSNRPLKVWLQQLKDAMYVLDDILDQLPTESSQLGCLTSLNPKKVIHRRELGQKLNEIIGRLDRIAQARSNFDLRQGLRERPSELVEWRQTSSTIAVPQVYGRDEDKGRVVEFLLSPSRSSEFLSVYPIVGLGGLGKTTLVQLVYNDPEVGNNFDLKIWVCVSENFTIKSILRSILEAVKKDKSEVMDLQVMEEKVKQLLQSKKYLLVLDDVWKRSQEMELGLTQDKWDKLRSVLSCGSKGSSILVSTRDNHVATIMGTCQAHHLGGLSEEDCWLLFKLHAFGADKEERAELVAIGKEIVKKCGGSPLAALALGGVMQSRRTEKEWLEVQKSELWSLPEENDIMRVLRLSYSSLTPTQKQCFAFCAIFPKDTEIMKQELIYLWIANGFISSRPNLEVEEVGNMVWNELQQKSFFQDVRSDDFSGEIYFKMHDLVHDLTQSISEQECICLEKHNLNDFSSNSHHIVFHGIRKKQFKKIAFEKVESLRTLYQLNSDELPFSSRLIRTNHSLRVLCIHPGKIPSFGSLSCLRYLELCDLGTKSLSASICNLRRLEILKLKRLSNLRSLPKHLTRMQNLRHLIIDECYFLSSMFPEAHKLRHLRTLSVYIVKSKKGYSLAELRHLNLGGKLCIEGLANVGSISEAEDANLKDKQDLRELSLSWSRSDGKTKSVVGAEEVLEALQPHSTLELLTITWYEGLRWPTWMQNNSAIHNLVSLRLVDSLKCGHLPPVGKLPFLKKLQISNMVELRFIEEDESYDGVEAMPFPSLEKLYLSYLPNVERLMKRETTHMFPSLSILFIEDCPKLQLPCLPSVKDLTVWYCSNEQLKSISNLNALNQLHLCDSDQVSCFPEGMMDNMTSLATLEIYSFRELKELPSDITKLTALSDLTINDCGKLECLPEQGLEGLSSLRKLFIHSCKSLGSLPDGVRHLTSLQSLTIGGCPMLKERCKQGTGEDWHKIAHVPHVKLHAF

>arahy.Tifrunner.gnm2.ann1.65CWRI.1

MWAARSAAISRIASGMKSKLASPKMKLEPDISPFSASLIIAFGRSPSSLSGSLLSQYGFDMKSSKITLMSSGHLIIMAGFGNIASNLGDLATELAKQVGGEALASRLVSVRNSGENFELLKQELQGLLALKEDKEKEVQGDRHKDTSSAYRLWSAKHPILSPEIEKRLKEVQQLVDKGSSVDCTVDEPPDRILKVFYAPEIMGYKTLQNALENIVDLLKSSKIQTIGVAGMKGVGKTALMQNLNNHDVVAEIFDIVIFIRLSADHTDHELQCKIAKRLKVDTEVINDPEEVARIIHEELQTKKYLLILDGAANEINLSQLGIPCNDNHSKVIITAQHRQVCTLNGAERMIEVGLLSRDEAWKMFCNTVGPVIGPPDIPEIARHIARSFRLKKSASSWRVGLEDLEERWPDYENEGVISEILPQTHAVELVHFNDIEEISEVGKENLEQIRGLSLEQCNGIRTLIADHARLFSAYEVE

>arahy.Tifrunner.gnm2.ann1.68FRR6.1

MQASPSSSFSYIWKYDVFLSFRGIDTRHGFIGNLYKALHDKGIRTFIDDEELERGDEITPSLVKAIEESRIAVPVERADWFFRFFYDVDPSDVRHCNNSYGEALARLEKRFKDNKEKVHKWRESLHQVANLSGEGFKLYEYGLVRKKYEYVFIEKITGQIWSRISRVPLYVADYPIGLESRVQKVVMLLSDGSNGGNQMVGIYGVGGIGKTTLARAVYNLIADQFEGLCFLEHVRENSIKYGLAYLQEILLSKVLGKKDIKLASVGEGTSTIRQRFSRKKILLVLDDVDKLEQLRMIAGGLDWFGYGSRVIITTRNKHLLAAHGVERTYEVDDLNMEESLELLNWNAFRNEKVDPSYKNVLNKVVSYASGLPLALIIIGSNLFGRTIEEWMSTLCEYKKVPNKDIQRILKISFDALEEYQQKFFLDIACCFKGYEREKIETILHAHHDVNPKSSISVLLEKSLIKINEYGFVTLHDLIQDMGREIVRQESPEKPGERSRLWFSVDIMQVLQENTGTSEINMINLEVPIYEVLEWDGTAFKKMKNLKTLIIQNGKFSKGPKHLPNSLRVLEWQGYPLDSLPSDFRPKKLAILNLPNSCFTSFQLLKILKTYVNMRVLNFSGNRQTTQIPDASGAPNLEELCFADCENLIKIDKSVGLLSKLKIFDVQGCQKLRRLPSLALPSLEKLYLSGCSSLVSFPEIQGKMKKLRQLSLIDTCIKELPYSIYNLSGLQLLELRHSGIILLPSSIFMLRELNALDLWECECLLAYEGDDIEQVSLVLSSNATSLDFSTCNISDEFLQIFLPQFPNVQVLNLSYCNFTILPECISECRFLEILYLNECKNLQEIRGIPPNIEILYAMHNTSMSYSSRMTLLNKEFHEDGGDKAFILPGTEVPDWFELQSDGQPISFWFRNKFPALSLCVLFNQRLTNHRELRIGVRLIIKGNNNFEELFNETLNIVSHLHHVYICSMKQVTFAADVDEMLLKNGWNKAVISLYHYNIFVPRELEDLGSMQIGVHVIEKRSNMQDIRFSDPDTHIQYSPILLNSRWLSQGKYHLLNQETSTVTVQGLQEQKNLTRIDHKKLKGHKKVNKAKREGTMLNINSMQDIEASNDKNMTPKSWSYMSCEPWDPRHESLPLDSLDPMLWESWGPVTQPQGSWNPSVARESWNSIDCEGTDTTNQLVSSKEITMSDSDSEMTRLSHRTAKWKLWLCGHRPLGDIRIDYDSTFCLPCNVFSIIMSTLPLTSSYSSSFCYAFTYDVFISFRGSDTRYGFTNNLYNALDRKGIRTFFDDEELRKGEEITESLVKAIEESRIAIVVFSKNYASSSFCLDELVKIMECVKGNGRLVFPVFYDVDPCDVRHQKGSYAVALTKHEERFKDDMKKLQKWRTALHEAANLSGNDFKLQREYEHAFIEKIVKEISNRIGRTPLHVADYPIGLESRVQEVNKLLNGGSNSGALMVGIYGVGGIGKTTLAKEVYNLTADPFHGLCFLENVRENSNKNGLAYLQELFLSKTLGEKDIKLASVSEGISLIKQRLKSKKVLLVLDDVEKLEQLQAIAGRPDWFGPGSIIIITTRDKHLLASHMVERTYQVKKFDEKEALELLRWNAFKYENVDPSYMHMLSKVVSYASGLPLALVVIGSNLYGRSVEEWISALHEYKRIPNKDIQKKLKVSFDALEEYHQKLFLDIACCFKGYSFDDLENVLHAFYDVSPILGIRVLAERSLINIDKDGHVTQHDLIQDMGREIVRQESPKNPGKRSRSWLPEDIIRVLQENIGTNEIELIILQAPTSELIEWDGEAFKKMENLKTLIILSGNFSRSPNYLPNSLRLLEWREYPSYSLPVDFHPKKLAILKLPKNCLTLLRLVKEFVEMSVLDFSWNDWIKHIPDVSGAPNLKELNFACCGNLINVDKSVGYLSKLRSLDFQGCIKLRNLPSLMLPSLRKLTLVNCSSLVSFPEILGKMEKITYLDLYSTSIRELPYSICNLTRLQEFDLRDGGTVLLPSSIFMFGELLELKFHGYKSLLLSNEGGVEQMPSVSASSTTKRIDLSSCNMSDTFLQIFISQFSNVQELNLSESHFTILPECIKDCHLLRILYLTSCKNLQEIRGIPPNIEILNATDNTSLNCSSREALSDKKLHEEGRKRRFILPGTRIPSWFEHQRSGESVSFWFRNKFPAISLCFLFTEALEFDDQCSLFPHLKLVTHDSDNGGCIFSDQNQSCPRATRSHMRIYDLEDDEGQQFQQNEWNQAVVSLSVEDKSGERELRPEEWGSIEIGVHIIKERSSMDDIQFTLPLLDKDHHKALHMKDSHKHHMQQQQTSLPLLQPVDNLNWDPRSYIVRRRESSRTSCRGDTLNIHGSSPLSLSGKASEEDVPSISSCTASSTSTDKYLGDENVISDDDDVEMNAFYASLDVSGIPMLPCSRDKLVTTTVSKDKEAREALRSVQHVISHDASVLLHPELCSILKANLDHLCKLSADHGRISREASKVISEASRVLTHWSRDYSEASVKIDSIMSHLQKADELEMSLESNKKRFLEVGQLNVDKAMSVSQIEAGMFRKRKREIFEEGKTVKTQLDELKKNLPQWEHEHTLAKKTQATIIAEWSRLRENFQNIEKDWNF

>arahy.Tifrunner.gnm2.ann1.69WEU8.1

MATICDVFLSFRGEETRHGFTGHLYDALCRNGISTFIDDENLRTGETIRPQLLQWIEAAKISIIVFSTNYAASTWCLDELVKILQCRRERNQLVFPVFYKVEPSDVRHQRNTYKEAVDAHEIRFGCHSQKVKEWKEALAETSNMKGFHLKQGYEFEFIQKIVSKALTGIPPRQLLIGDHMVGLQTRVVEVESHLYSKYRTLSNGESPKRKHYNNIAMLGIVGIGGSGKTTLAKALYNSICDRKISDQEEGLVRLQQTLLSKLLGEGEIKVRSVEEGISMIKEKLSKKRALIVLDDVDKIEELKALAGECDWFSDKTRIIITTRDKYLLTAHEVEKIYEMKLLSDPESLELFCWNAFKMTGPKANYEDLSNQAIYYAQGLPLALKVIGSNLINKNLEEWKSALDKYEKNPPKDI

>arahy.Tifrunner.gnm2.ann1.6I5Y8L.1

MSVLLLEKVPSPTMDIQEESSTFGPLVAMSSRNMSSSSSAFFSANQSPFFSPRTSSCHLSESVRPEASSDRIHADAALPSTSAAIPELKSLANIRSNFSDVSVSVSASPAGCNSGDLQKLDRISSSVGISSSYDDSYSGSKEKGRKKGKNQRTSSTPGSRSISSYRLKSCDVFIGLHGRKPPLIRFANWLRAELETQGISCLVSDRARCKDSCKLGIAERAMSIASFGVVIITRKSFKNPYSIEELQFFSSKKNLVPIYFDLGPADCLVRDIIEKRGELWEKHGGELWLSYGGLEQEWRDAVHGLSRVEEWKLEAQDGNWRDCILSTVTSFAMRLGRRSVAEHLTKWRQKVKEEEFPLTRNESFIGRKKELSQLEFMLFGDVTGDSQQDYIELKARAKRKHLTIGRGRSYVLDERLREKQNTEVKEPVLWKESEKEIEMHSTEFSHRHYRPRVKRGGKFGRRKREMKVVYGKGIACVSGESGIGKTELILEFAYRFHQRYKMVLWLGGESRYIRQNYLNLRSFLEADVGANNSLEKTKITGFEEQEESAISRVRKELMRNVPYLVIIDNLESEKDWWDHKLVMDLLPRFGGETHVIISTCLPGIMNLEPLKLSYLSGVEAMSLMQGNGKDYPVAEVDALRVIEEKVGRLTLGLAIVSGVLSELPITPSRLLDTINRIPLKDMSQSLRHNNFLLQLLDVCFSIFDHADGPRSLATRMVLVSGWFAPAAIPVSLLAHTAQNLPEKQKGTCSWRKLLQSLTCGFASSHTKKSEIEASTLLLRFNLARSSTKEGYVQFNELIRQYARKRELTGAPEAMVQAVISQGSISQDLEHLWAACFLIFGFGHTPPVVELEVSELLYLVKKVVLPLAIQTFITYSRCTAAIELLQLCTMALEGADQAFVTPVDKWLDKSLCWRSIQTKAQLNPCLWQELALCRATVLETRAKLMLRGAQFDIGDDLIRKAVFIRTSICGEDHPDTISARETLSKLTRLISNVQIHASA

>arahy.Tifrunner.gnm2.ann1.6I7HCV.1

MSISFIPGFLQERAIDAATTYVTRQLGYVWDYEKRFVDVSKAVEALKNDRDGVRDKAEEDEGRYGRAIYDNVVEWLACVDGILAEYEKFKQEHDDNGEYALAFPFQNLDIRYHRSKTAEDIKERVEELQNEKHDSISRWQGPPSSMGYALPSVEYEELDSRKQNMEDVRKALEDSSATMVGVHGLAGMGKTTLIIKAINTVQSREPKLFDMVIMANVGKNPDIRKIQGQIADMLGITLQEETN

>arahy.Tifrunner.gnm2.ann1.6KH6YE.1

MERESTRSRSIRIWKHEVFVSFRGEDTRNNFIDHLFAAFDRKGIDAFKDDINLKQGGHISTELMEAIETSTVLMVVLSKNYASSTWCLRELEKILECAKVHKTSQTVLPVFYDVTPSEVRTQSGDYEKAFAELERRFERDLGMVQKWREAMAQVAAFSGWDVQNKPQHTEIEKIVERVIDIQHCKSSNLDGLVGMHPRVEELEKLLDFDCSSDDDDVRVTGICGMGGIGKSTLARVVFQRNLHRFSVTCILDNLSQVFHRDGLVGAQKLLVSHILKDEMQHMWSLSKAMRLMKARLCRVKALIVLDNVDSEDQQLEELGLNPEYLFPGSRIIVISRDKHILKMSGVDEIYEAQLLNKEESYQLFCRKVFRRNNFMLQHCDELLVDGVLKYAQGLPLAIEVLGSFLKERSMDSWKSILSEMRVCPPHEEIMDVLKISYDQLDCMEKQVFLDIACIFHDGSDKEEVMRILDCCGFFADIAVCNLHDKSLITINKNEGIEMHGKLRELGMEIVRKEALMEPGKRSRIWRFQDFLNVCENKAMDYVEAIRLESQTMDEKNTTLSVEALSKMNQLRLLKINDVKFSGNLSCLSSKLRYLEWAEYPYTYFPPSCEPSNLVELSLPHSSIKQLWNDIKCLYNLKSVDLHGSQNLIKIPDFSKAPNLEMLNLEGCTKLVHIHPSVRFLEKLSYLNLKNCTSLVSIPNRILSISSLQVVNLAGCSKLWKHSRKQS

>arahy.Tifrunner.gnm2.ann1.6KYT3I.1

MENFGTRAWVCVAENPNPVNVTRTIIGAIDSSSCNVDHFDSLQTDLKEKLTGKRFLVVLDDVWHDRRDMWEDFLKPFHYGNTGSKILLTTLSEKVASVFVANNLHYRLSLLSDEDCWSAIGRIYSIFPEDYEFDKDELILLWVAEDLLQPKENNALENVGCAYFDELVARSFFQPSSTNEKLFVMHDFMHDLATVFAGKFYFKVKEFGNQHVIDNKIRHLSYAATEDEDSIKLFQEANNRAVHMRNVSIYIFLFFTIVNQLILKAILASYANNWGLLLASLLDSIGELIHLRYLNLFDTPIVTLPESICKLYNLQTLKLKNCVELEMLPRGMQGLVNLRHLDIRGASHLKEMPKEITKLKHLNFLSDYIVGKHEETGIRELGTLDNLHGSFCISKLENVKNSGEVLEAKMGNKKHINTLELNWLPDGDIDDVQTERDILGKLQPHQNLKELSIVMAAPHLEELHQYDCPKIDCFADEGLPPSLKNFNSLSATNSRSEGLTHLQLYRCCDVKSFPGEGSLPASLEYLELRKLPNLETLDCKGLHHLTSLKNLRIRYCDYLHNIREEHLLASIENIYIGNECPLTRKLKAMEDLRIEFARYYLVFVILYGILQTRITLMMITGTIDSDFTPFYVNERGQRHTGIGLARDHESSMISFLKH

>arahy.Tifrunner.gnm2.ann1.6QC6B5.1

MAESTVSFLLDKMSALLQEEVNLQRGVQHEVQYIKEELERHMAILRVADALEGKDPELKVWLQRVRKIAEAMEDAIDEFNYQDNKSSSTFSKIKNMKARHEIASEIQQIKCRLEFISKERPSLYRVGSRLSPRLPSKIESQGDALLLEEADLVGIDKPKKQLCDLLFSNEAGRFVIPIHGMGGLGKTTLAKQVYDDAKVKKRFKIHAWVSVSHPFQIEELLKDLVHQLHNVIGKPAPEEVGQMKSDKLKEVIKNLLQQSRYLIVLDDVWHINVWDSVKLALPNNNRGSRVMLTTRNKNVALYSCTELGKDFHPEFLPEQEAWSLFCRKTFQDNSCPPHLEEVCQKILKMCGGLPLAIVAIGGALTTTNKANIEEWQIVYRSFGSEIEGNDKLENMNKVLLLSFNELPYYLKSCLMYLSIFPEFHAIEHMRLIRLWIAEGFVIEEGGKTLEEVAESYLKELLDRNLLQVVEKTSDGRMKTCRMHDVLREIINLKSKDQNFATVVKEQSIVWPERVRRLSLVNTTHNIQQNRTTFQLRSLLMFALSDSLDHFSIQAVCSTGYKLLRVLDLQDAPLEVFPDGIVSLFLLKYLSLRNTKVKRIPSSIKKLKQLETLDLKHSHVTELPVEIVELHMLRHLLVYRYEVESYAYFHSRHGFKLAAPIGNMQSLQKLCFIEADQGGKALMVELGRLIQLRRLGIRKMREEDGAALCSSIEKMINLQSLSVTAIEDDKIIDIHNISSPPQFLQRLYLSGRLEKFPQWISTLKNLAKVHLKWSQLKEDPLVYLQGLPNLRHLELLHVYVGETLHFRAKGFPSLKILGLDDLDGLKHMIVEEGAMPGLKKLVMQRCKSFKQAPKGIEHLSKLKTIEFFDMPEELITALLPNGGQDNWRVQHVPAVYSSYWRDRDWDVYSLETFAERAYDCSHDAAMSSHEIRTLWKV

>arahy.Tifrunner.gnm2.ann1.6SV629.1

MKSIRNNKKAEVQKWREALTVAANLEGLNSHSYRDEIEFIQNIVKDVFQKLIDHYPPNDSKSLVGISENLEKVESLLSESVEVKMIGICGIGGIGKTTLARLIFEKYSYTFEGSCFLENVRERSRNYGLTELRRRLYLELLQGKFWQNNTGKSTFVEDKVSKQRNFIVLDDVSSLEQLDYLVQKLQWCGAGSKIIITARDKNVLVSTVETIYEMKILDSHESFKLFSLNAFNEDYPQIGYEELSWKAVGCCKGIPLALIALGSFLHSKSKAEWHSALQKLEKTPDLEIQNILRLSYDGIDEEAKQIFLDIACFFKRELVEYVVNLLDSYGLYAAIGMRSLLDRALIAISHNCVRMHDLIQELGWDIVCQQSSGNPENRSHLWDSNDIQDVLGNNKGTDSIESIVFDMSQIADLHLNADTFKKMHKLRFLKLYIPHESDGRLNKLQLPNLVNLEEVDLTDS

>arahy.Tifrunner.gnm2.ann1.6TY67H.1

SDNVLILGILEIGGIGKTTIAKAIYNQAHGCFEGYCFLPNIREFWMQSTSQVHLQEQLLSNIFKTTKIRIHNIDSGKIILQQRLCGKKLFLILDDVDELDQLNALCGSREWFGPGSRIIITTRNEHLLKVLQVDHISRMSNMDHHESIEHFSWHAFKQKCPKREFLELSKKVVAYCGGLPLAHEVIGSLLFDRMENVWESVLEKLTRIPNHEVQKKLRISFDSLVDDTIKEIFLDIAFFFIGMNRNDVIHILDEYNAEIGIDVLIDRSLVTVDNKNKLGMHDLLRDMGREIVREKYPKEPENCSRLGTRATEGLVLKLTRTNTICLEAKAFKEMKKLRLLQLVGVQLGRDFEYISRDLRWLYWHGFPLKYIPPNFSQEICLKLVWKEPQFLERLKILNLSHSQHLTQTPDFSYLPNLEKLLLYDCPCLTAISETIEHLKYILLINLEDCTSLHNLPRSFYKLKSLKTLIISGCSMIDNLENDLDQMESLVTLIADKTAITQVPFSIVRSKSIGYVSLCGYEGFSRDVFPSLIWSWMSPTNNFSSQVETCMDLELVSSDVGKLRIKSLLIEVGVKNQVSNILRERKLSTNEHDNYLLPGDNYPDWLTFNSDGASIIFNVPHGNGRDLKTMTMCIVYSSSTDTISLKCLKNVLIINYTKSTIQVYKCDTIASLEDDEWQSIISSLEPGDRVEIVIVVYGTEFVVKKTSVYLIYGEPNNEDRDATDMPEDKNVISFGKAKNRFRGPLSLASVLVSPLFWIGVAGFLIWKHCLSDKRRRHGCIKFSNHEKTFPFLV

>arahy.Tifrunner.gnm2.ann1.6UQ1FU.2

MAYSSSFLLDAPRIKHDVFISFRGEDIRTSFLSHLRKELHRYHIDFFVDDEKLHPGDDISSTLIQAIEESSISLVIFSENYASSTWCLNELVKVIQCMKQDQRIVIPVFYKVVPSDVRHQNNSFKEAFDKHQHRLKGNMMKVQSWRFALKEASNLSGFHYPSKYQDESKFIEEIVNDISEKLSYIFSIESKGLVGIDDNFTSIESLLEIESGEVRIIGIWGMGGIGKTTIAEFLFDKYSSQYEGSCMLKNVREESQKFGVPHLCEKLISELLAGERLVLKGSSKARSAFIQRKLSRKKVFIVLDDMDTLEQFEHLATKWLGPGSRIIVTTRDKHVLRKVHGIYEVQGLSFKNSLKLFCLNAFDKVYPETGYEEFSEIAVNYANGIPLALRVLGSFLYSKTIEEWESALGKLKIYPNIDIFNVLKLSYDGLDDLEKDIFLDIAFFFKGEHKDVVISFLESCGFFPAIGIGNLSRKALITISNRNRIEMHDLIEQMGREIVRQESIKDPGRRSRLSNHEDVYNVLNNNKGTDSVEGIMLDLSQIKRDLHLDADTFKRMPNIRFLKFYDSWRQKESANVHVSSTFDSFPKELRYLEWSGCPVKSLPPNFCAEKLVKLSMPNSQVSKLWDGVQDLVNLKKINLRGCKQLVELPDFTRASNLEEIYLIECVRLFELHPSILSIHKLKTLSVWGCKALKSLKSNIHLKSLKELDVRCCSSLKEFSLSSEELRSLDFRGTGIDKLYSSVSHLTKLVEFDPSNVRLETLPNEVCLLVSLEVLNLEGCKQLIELPQSMKVLSRLQELNLEDCFSLQSLPELPPSLIHLSATNCRSLEKLFNIKTVFSLNLKSISFENCERLDEHSFLEYVHLTMMGVAIRDILDDMLQYKIEPHADDDDITFFPESNSQVFYPGSKVPPWFIYQTREASVTIDLPADQPLNQLVGFILCCVVYHIPSHIESPTTSRAFSSKRPPIIRCQYSGFGYSKQFAKTSRWNSDHVCIWFHAAHNRQWHGNNATFKFKAETLSEFKVETRCEDYTPWRKRIPYVWRVIEEWEVIGCGVYPIYASDILDVFQKVDPQFQFFKDRSSWERESGWTDLQSVYTLDEVKTRMIHKMEADQKRFSSVSRICQISNNRTRMSMVSSKPKEVSQSLNEEENQVDQRNSSPLPRRLVRKMFKCLFGCC

>arahy.Tifrunner.gnm2.ann1.6X03KB.1

MAAKLEGGAYLSSFVDAILEKLSSILKDDSVLEGNEPALELLRSLEETLSDVEPVFDDAELKQFSDKRVKKWLVDLQDALYVADDLLDELSTKAATANPRDPGNSSFWSRAVDSCIEDSGLNVIEKIVGTLESVVGRKGKLGLKESAKLDTSWRIPSTSLVVSSDVFGRDQDKENIIKLLLDDTCDAGSRVTVIPIVGMGGIGKTTLAQLVYNDAKVVEKFDTRVWVCVAENSDPVNVTKTVIGAIDSSPCTMDNFDLLQTNLKEKLTEKTFLVVLDDVWHDRRDMWEDFLKPFHYGNNKSKILLTTRSENVASAFAANNLHYRLSLLSKEDCWSVFLKHSSISTHLKQYATLEPIGRKIVEKCKGLPLAVKTLGGLLRNKVNKGDWENILESEIWELAEDNSKIVPALRVSYHYLPSHLKRCFVYCSLYPEDYQFDKSQLILLWMGEDLLRPKKKKNSTLENIGCAYFDELVARSFFQPSNTNKKLFVMHDLMHDLATFFAGKFFFKLEFGNPYMMDNKTRHLSCAAKYEDSIKLFREGYNGAACTRTFLDFSVLPYFESRDIEGHPRLLRQQLRVLSLTIKSMPDSIGELIHLRYLNLSKSPIVALSESICELYNLQTLLLRNCYELEMLPSRMQDLVNLRHLDIRGACRLKEMPKGMSKLKHLQFLSDYIVGKHEENGVAELGTLDNLHGSFYISKLQNVKNSGEALEAKMGNKKHINTLELNWLPDGDIDDVQTERDILDKLQPHQNLKELSIHGYPGERFPDWLGLSCYSNMTKLSLDSCMNCCELPSLGQLPSLQHLEVYELHGLEKIDFEIYNKNNASLQPETPFKSLETLKIHDMCGWREWHIPDEFDGFPELRVLEITNCGVLRGDLPAHHLPALEELTIADCEEFACSLPRAPKLHQLNVRNRFSKSKVSTGPHKVVISKTQLAKSALECLSHIQSPRVQYLDIRGCESALSISADCVPASLQYLQIQDCSKLTFSEQLQHKSLTEISIQWCHSLTSFSLGALPNLQKLTIRDCLNMEYVEVPQALPSLRYVWISNCRSLVSLPALGLVAPHLEELKIKDCSEIDCFAGKFLPPSLKKLEVCGCEKLASWITSNGLQSEGLTYLLLENWNEVKSFPSESLLPASLESLRLRCFPDLETLDCKGLRHLTSLQHLVISCSEKLENITEEHVLASIEKIYIGRESPLRRKLQEMEDLQINFGKCSCSDLP

>arahy.Tifrunner.gnm2.ann1.6X8TD8.1

MALPTPPLPDWLVQWATKILKQELNYLLYYGKNIKDLENQVNKLKLERQKLGDRVAEDEDRHGREIYDDVSKWLDGADTIIDAYEKFEKEEEEAHMKCLAGFPPNLPARYFLSKKSIEIKGKAESQLQKAKFDIISRSRGPPSVSLVLSNVDYQSLPSRVTAMKDITNALKDSSARMIGVHGPSGVGKTTLVMEAVNRVQNDQEKPKLFDVVIMANVTKSPNIRKIQGQIADMLWMKLDEESEEGRASRIRERLKKEKESTLIILDDLYGKVDLNILGIPWQSGDGNQKNPKGKKSLGSSTPNEETKQQGLADGGVMMNSQKASDASSSSSLASNSVIALTTEERYKGCKVLLISEVRQVLNQMDVRPKLVVPLELLNEKDARTLFNKIAGIGDKSTEFGELPAQIVEKCDGLPMSLVTTAKALKGRSRLVWQDTYQKFETQTMTGTPEHSTRVIYDLLENEELRITFLLCACMDNDALVSDLVRACIGLGFLRGIYTVKDTRSRVQVMLMKLRESGLLSDSYSSDRYTMQNLVRNAALSIAFKERHMLMLNKVRVDEWPDDDELRRYAAISLRHCDIIDAITKTMTCDRLKILEVINNDPQLKLPKKFFEQMKELKVLILTGINLSPSDSSVGCLTKLRMLCLEHCTLSSSKEELSLSEELSIIKNLENLRILSFSGSNINCLPVELGDLSKLQTLDISNCPKLRVIPPDVISRLTSLEELYMRKTQIQWPKIINGDENDERKNASLLELGELNQLTNLDIQIQSVDHLPENLFFDKLSSYKIVIGSSNRYMKRDFKMPEKYELSRFLAIHQKSGIHIHSHKGIKMLFERVEYLLLGKLNGVQDLFYELNLKGFSHLKYLAIQNNHDIQFLIHPKDREEHHEKAFEKLETLELHKVTQIEGLCFSSYLLSESSFVNLKAITINFCEKLKYLFLPSLLKHLTALETIQIFDCDSLKEIVPVERPDEILERLELRSIQIDQIWNDQSSNFGNLIHLDVSGCHSLKYLLPFSLAKNLKKLQSLYVSECYQMENIFPDGPVKVAKNVMVLPLFLPPLSPTRYLIRANPPVSKPDHAPNHHHHPHPFGLLPHLLSSRVDSESSSFSLHNPTKHSTASASSMTEETEDDDEKDVGRTDATFPNLKNIKLSRMSSLRKIWNLNVPVEKLDTLVIEKCNQLVSVFSHDMEGIFQGLSSLTVTDCKSLETIFDLTADQNWYARPYQTLLRDVHLESLRKLESILRCKQDQEGTLQLKSLQNITVHGCGNLKNIFPFTIAKHQLENLQCLVVSDCSTLKEIVAVQNVTSDNSSSSGTSNPVPLVFPELTSIKFSKLPEFENFCGGHCELKCEKLDEVSIELCDKLELFRESQLLHETFPSSQAATSAQRKPLFHEKVMNKLRSMHIELQHTSSSTRYRRDKLEVLRLSRLEDTEILFRFLHSNPNLKSLWLDNCLFDKLVESQEKSSSDGNEHIGVVPKLKTLRLTNLSCLKEIGFEHDAILQRIESLLLENCPKLDTIVPSKERIRFNFLTNLEVVYCKCIKYVMPLSTAKSLGQLVTMKVINCESLEEIIVSDHQTVDKEVDEQSRTNNKIVFKQMKALELVSLKNLKSFCSSKICSLEFPLLEKFVVSACPKMEKFSEKEIKTMPTIMQKVYVVRDEEKRLCWNGDLRATIEHIYNKKKYYEGMDKISVSEHLVLEEGWKTEKALDKGWFYSLKTLTLERCKFESFAIPFFVLRCLKSLKELEVRNCKNITCIFEMNDIKGTFQLEKLTLEELPNVTHVWPQQDKQNDSRFRNLQQVFVKSCRKLKALFPVAIATNLKMLEQLEVHFCDELLEIVEKGSGGGGETKKFVFLYLTWLGLFNLPRLTHFYDGMFTLECPELKILYPFNCNKFELFQTPQENSPSITRPALFSDIKDISKVKTLSMKSKDNSVLKSWSQQSEDLKLEYLRGLMLTFDDDVNNEYSTLQCEILGRRTPNLQRMGIMNSTSLKNILFPSQNRKILENLEYLALSCLFELSSIGGLEYLSKLQKFGVFQCPLLRTIEQYPSSLKKLNVAGCHGLQCLFTSSAAKSLKHLKELYVYDCKSLKDIVRKEQGDETATEEIIFQQLKSISLQYLECLECFYQGNAALKLPSLAQAKIWKCPKMIIFSQQLRQEDPSEKVSVSFHSGADKSEVQLTHYHQLNLAVGAQFLNQTFLFLNDYPEMQGKWVGASGIPVEWSFNYLKYLEVEGCDFLTNAVLPSHLLPLLRNLENLTVKKCKFVAAIFDVKDTPPEHDDPNKIVKIPVKKIILEELPTLTHVWNNDPKPSLSFPSLEKVFVEECKSIKSLFPASVPRDNLEHLDVRNCGELEEIVAKDEAFPQEVIILFPKLTCLVLRDLPKLRRTCSGMQSLLDWSAVLTRLYVSRCPMLKVFAQDIQNSNPGVTTSNFEELELSKEDVRMIEKGLLHVDLQNLNYLGLNNFNDNETDEFPDVFLSKMSLPKLKEFQLLDCAFKYIFRPKRPDMDYSKMLSQLNYLNITNLHKLNSMGFEHPWMAPLLESLETLKVSECNLLTNLAASSVVSFLYLRVLRVENCAGLKYLFTSSTAKSLGALQELVITKCKSLEKVVAHEEGDKPDDMVLFSNLYTLSLNELPQLESFYTGNSTLYFPMLYGNLLCTITKCNKMKTFSHGDVLPKFMQGKIDEDPWHGDLNAAVQKQFQKAHIITTTAPWTYMDLLPTERSLLLLSFITLAQSANSTIKKKQRRTLELPVSTCPPEYNENVTG

>arahy.Tifrunner.gnm2.ann1.7121CT.1

MSPESDITMPSPASFRLRWDVFLSFRGSDTREAFTEDLHGALEAAGVRAFIDNEGLQRGDSISPSLVEAIADSAAAIVILSPDYASSHWCLEELAKICECGWKLVLPVFYRVDPSHVRKQKGPFEKAFRLHEESERFRNKVASWRSAMNKVGGIAGWVFGDHNSNKDQLIRVLVQTVLKQMRNTPLTVAQYTVGLDDRVAELRKLVDVRSSDVKVIGLYGMGGVGKTTLAKGLFNSLVDHFDRRSFISNVREVANNEDGMVSLQGRILGDLSPGTENPINDVNAGISAIKRIVEENRVLVFLDDVDDVKQLDSLIGKREWFSKGSCVVITTRDRAVLQERYVNVKYEVKELKESQALELFCYHSMRRKVPADGFLNLSQQIVSLTGGLPLALEVFGSFLFDKRTINEWKDTLLKLKDIRPDKLQGALKISFDALDEQDKCVFLDIACLFVQMEMKRDDVVDILNGCGFRGEIAITVLTTKCLIKIIKDGVVWMHDQVRDMGRQIVQNQSILDCGSRSRLWDRHEILGGTRKVEGIVLDCVKRSLAKPRDRTAEEITWDNFRQMPGYKTASAYMKAKYKNYVEHRGEKAKKFTLDTKDFQPMVSLRLLQINYSRLEGQGKFLPQGVKWLQWKQCPLKNMSSSYYPLELAVLDLSESKIETLGGKHHNKVAECLMVLNLSSCHHLVAIPDLTGYHCLKKIVLENCTALTRIHESLGNLSTLVHLNLGTCYNLVELPSDVSGLKHLEDLILSGCWKLKALPKDLSCLVSLRKLLIDETAITVLPDSIFHLTKLEKLSCSGCRLLRRIPTSIGKLCSLRELSLNHTALDELPDSIGCLQNLEKLGLVGCKSLSIIPDSVGTLISLTHLFLDVSGIKELPDSIGSLSYLRQLSVSGCTSLGELPMSIKALFSLVELHLDKTPINILPEHIGAMKMLQKLEMANCKDLKFLPASIGDMSALTALDMYNTNITELPESIGMLENLIHLRIDMCKELKRLPKSLGNLKSLCWLQMKETAVTQLPDSFGMLSRLVKLDMERRYYLNMVGDKNTEEPNSVGILRSFCKLTLLQELNAHGWRIAGKIPDDFEKLSSLETLNLCHNNIISLPASMRGLSCLKKLLLSNCTQLIFLPSLPSSLVEVDVANCIALERISDISNLDNIEELNLTNCEKLEDIPGIEHLKSLRRLYMSGCIGCSHAAMRRFSKDLLKNLRILIMPGSKDIPDWISGESIIFSKRRNRELKGIICAGVLSFDGIPENQRDALQLVDVQAKVFNLTDNVYSTTFRLLGVPRTNEDHIFLHRFGAHTSLVHQLKDKYTLHLTKRNAPFVQELKLKSCGIHLVFEGDDDYEGDEESLDESQYSVSQKLAKFFNSCYCPRSHLHVNTSTSNEPLEIGRSITRSTVPDPWLRSILFSATFLCALSYLYLRYLNF

>arahy.Tifrunner.gnm2.ann1.714S40.1

MHTAIWLSPPVYVKCGTSLASFASLHLTQHSQHSYNFLSFFLIMPLQSSFSSFSTHSSSSSSFGYAWEYDVFISFRGEDTRYGFTGNLYKALSDKGVHTFIDDEELQRGDDITPSLLTSIEESRIAIIVLSPNYASSSFCLDELVHILHCIKGNDRLVLPVFYEVDPSDVRHQRNSFGEAMAKHEEKFKSDLNKVHKWKQALHQDGYEHKFMRNIVEEISRKIRRVPLFVARFPVGLDSLDSRVSKVISLLKTDSSDQVHMVGIHGVGGIGKTALACAVYNLIADHFDGTCFLEDVRKNSERHGLAHLQNILLSEILGKEEIKIVSFQQGTSRIQRRLCQKKILLILDDVDDHKQLQAIAGKPDWFGPGSRVIITTRDTHLLKYHGVENTYEVEGLNEVESSQLLVKHAFKNGYVSSPSYADVLTRTITYASGLPLALQVIGSHLCGKKVEEWESALNKFDRHLDDKIHEILRVSFDALGKEEKSVFLDIACCFKGYELKEITDLLQAHYGSCMKYHIGVLFEKSLIKINLYDLSVTMHDLIENMGKEIALEESPEMPGMRSRLWFYEDIVKVLQDNLGTSTIEIIYLEFPLLEREGDEDSFEKEGNKEVEVKWDGTAFKEMKNLKTLITKNGCFSECPKYLPNSLRVLEWWRFPSEWLPNDFQPKKLSILKLWNNLYLAHKLDSLSKKLVSLKVLNFDYNDSLKEIADVSSLQSLEEFSFRGCKNLVKVHSSVGFLSKLKRLNAEYCEKLRTFPAAIKLPLLKDFSLCGCSSLVNFPEILEEMANVGWLDLKGTGIKDLPCSFHNLSGLRNLEIIWNEMCKIPSIIFMMPQLSSCYIEGGGKKRRVSSEKPEEDEEEGLQGILTHSLPSQHMMKALCLRDTNLSDDFFPLAVAWLPNVRQLSLRGNNFTVLPECMQEFCFLYLLNVDDCNHLQEIRGIPPCLTNFSAVNCKSLSPRASRVLLNQELHEGRWTIFAMPGGRIPRWFEKRCSGASISFWFHGTEFPDNALFFAILLKEGLSSPVEVLPIVTVNGNQVSCRWRETPVDQLFIFHLSETIAYNVVLRFENKWNHAEISYEAHDYYTYDEVPTESIAKEIGIHLLKQKISSSIIQHIRFTDPYKMTQLIIMMMMVSIVLPNHKKQPLLLETSIANFLN

>arahy.Tifrunner.gnm2.ann1.71Y0FT.1

MACVASATTSSSYFPFTYDAFLSFRGEDTRSGFTGYLYYSLTNRGIKTFIDDEELGRGKEIASSLFTAIQQSRAAIVVLSKYYASSSFCLDELVKILDCVKGKGRLIIPVFYDVDPSDVRKQTGTYGEALAVHENKFKFERLKRWKIALEKIADLSGYHFKHGDGYEHKFVAKIVGHVSIEIKRATLPIVDHPVGLDSQVENVISLLNVGSSDGVCVLGIHGIGGIGKTTLAVAVYNWLVNHFAVLCFENICFHENTRENSNKYGLKHLQKTLLFELVGENKVTLMSVREGISMLKQKLQRKKIILILDDIDQQEQLDALAGNLDWFSPGSRVIITTRDTSLLSRYEDRITYELDKLNDKDSLELISFKALKTNKVDRSYLDILSSVVTYASGLPLTLEVMGSNFLGKSIEQWKSALDQYRKIPNKKIQNVLKVSFDGLEEFEKEIFLDIACCFKECELTDVKRILCAHHNVDSLEYGLKVLVEKSLIKMDAYRGIILHALIQDMGREIVRQESPKKPGKRSRLWLLEDIVQVFEMNMGSDKIEMIHMDFPKFEKVIRWDGEAFKKMRNLKTLFIRHTYFSQGPKYLPNCLRVLNWEEYPSPCLPLDFHPEGLVIFQLSGKSIQSYMSLTVINLDHSNVEEIPDISGVPNLVNLSLNMCLKLIKIHESIVFLDRLSVLSAQGCRRLKRFPSIKLTNLVHLCLSGCSSLEHFPKMLGKLKNILTIYSDGTLIKELPSAIKRFCLISHSSMIKHGASSPPALSSPIVMSPDEEKVTSERSEVDVEEEQGSPMAPLEAKHSACKLPDLLHESFTTYLTWFRNVEDLDLSEHNFTVLDESLKELCSLRSLSLNGCRELREIKGIPPNIKYFSARNCISLTYASKNMLLNKELHEDGAKDFVLPSSSIPNWVEHSSNNDSISFWFRNKLPEISLCVLVGPAVDFSCTHICPEFIINSSRGQAEHLESVETSNQLVDHIFITDPKLMKSKVNEVILENEWNHVVNMQQQKYMASHERRLSLDLPLGMSFSLNDHGTKFQCASKELSLRQCWQEPLDSHLRSNLMQLTSTSYNIRDEETAQAVLDHPANILAEYSQSPFLAETQNLQAPLFPSSLSGMAIREEFHHKTCCTVLPTTTDESFGNQVDVSQDDDADDLEMEGFYASLDAETNVISFSPNEETMEALKIALDFITNNNDASVFLDAQHCCIMKTSLDYLSSLSANDGLSGAMRALISEASTVFAHCSSSYIEANMKVESTASELLRADNLKSDLENNKNQFNDTVASEKELRQKLARLEEMKEELEKQIRTTNANIMASRKEQNKTRKRKRDVYVEGKALKAQMDVMKEKVPRLQHEHDLAKENQEKIKAEWSEFGEKFKKIVVKSAVQDYQEEFGFVLKQQVHDKNFNFY

>arahy.Tifrunner.gnm2.ann1.73EBH2.1

MAEALVVGALVSGSISLVLNRLISPEFVNSVVSKKLNRKLVERLKTALLAAKALAADAEQKQFGNDHVRKWLDSLRDALYTADDLLDRVFIKARIRKKVRVRLPLRLNLSVRKMVAKINEVVKRIEDLQKLKDSLGLKEIPTGSSSWRTPSTSLERGTVYGRDDDKQALIKMLNDNNNHNLSVISIVGMGGVGKTTLAQCLYNNKDLMDGVDLKAWICVSENFDVVETTKNVIKGISSGVCSLDNFDLLQQDLKKKLSEKKFFIVLDDVWSEDADKWNSFITPFQHGRKGSTILLTTRKVNVGRIVQHYNSYTLKELSDDYCWSIFADNASFPESNGSSELEEIGRKIVERCDGLPLAAETLGRLLHSERRVEEWNRILSNDIWEFPMSNSKIVPALLISYYHLPAHLKRCFVYCSLYPKDYQFDKDELILLWMAEDLLRPPRKGETLEEVGCECFDDLSSRLFFKQAEHYARKYFVMHDLMHDLATFLAGDLYCRFSKELGEKEERNILTRHLSYTHSIPEKACSSSEIKSLRTLLYINDIPYIRDERATLPYDILSKNKYLRVLSFDRLNIFPDSIGKLIQLRYLGLSRSDVQILPESLCNLSRRLFKADYAAQWHVLMRIVFSSSDVSKVSQLEIQEDGERWYKKMSLDEDSLSIRGFECVVESAFKARIIHHLTSLQEIEISGCSSVVSLGGNCLPKSLQKLKIFNCRQIELLQQQHKYDLVNLQIYESCASLTSLSLDAFPNLENLEIEWCSNLESVSMSEPPHTALQRLTIISKTFNSIVTLESLRTSLYVNDLFSMESIASKFKYLRVLSFTKLDVVPNSIGELIHLRYLDLSWTYIKTLPESLCSLCNLQTLKLYYCRKLATLPSGLHNLVSLRHLDIRGTSLEEMPGKMSKLNQLHILSYFVVGKYEDNGIQELGGLVNLHGSVEIKKLENIVDVKEAMRAKIMDKKHIDELCLEWSSGDDLVSSTQKERDILDKLQPRSGLKVLRIWGYKGTIFPDWLGNCSYENMTRVSLKSCKNCCMLPSLGQLPSLKLLRIRGFGQLRSIGEEFYKNEGDHHSSRIAPFPSLETLEFDNMACWEVWHVSESETFPQLRKLQITNCRMLKEEMLNQVFFRIVSSLSDVSKVRKLLIGDHIRRHTEAMFLDGDTLTIRGSESVMESAFKAMMSINHLRCSNLESVSMSEPPSKSLQNLRIIKCRKLEFLQQQHKYDLVGLYIFDSCDSLTSLSLDAFPNLKNLKIFWCRNLESVSMSEAPHAALQRLSIAFCNKLVSLAGEGQAAPNLTHLSLIECSKLEALPRDMNSLFPSLHSLHTYCPNICRLEEGDLPPNLKVLEVGICEEQMRDLSWMPNLHALTRLIINGSWCKSIKSYPEVGSLPHLPSLITLVIWRFHNLETLECNKLLRLTSLRQLHISYCWKLENMEGEKLPPSLLLLKVYHCHLLGEHCRNKHQQIWPKISHIPTIKVNGEQMF

>arahy.Tifrunner.gnm2.ann1.74P1QG.1

MQVVGLFYDGVTAADVRWQKEDRAYGKAITQHKERLRLGEESDKIKTWRSALSRVCDLIALNCDKKYETELISMIVKGVSVRLPPLRLQIKDVIGLDSRFEEVKSHLDIGNNDAVQMLAICGPPGIGKTTFAAYIYNNIINHQYIAASFISNIRDKPKVEDLQSTLLSEMGEKRESRRGDTDGGGREIKRKLSVKKVLLVLDGVDKIEQLKSLAGGCDWFGPGSKIIITTRDATLLNRHRVKIKRYQMTELSDDDSRKLFCWYAFDGGEPAQNFANLVPQALSIAKGIPLALKKLGSRLKDRSLDEWEMELDRYNKVPEAFEFLLKSFGN

>arahy.Tifrunner.gnm2.ann1.7657UC.1

MAGALVGGAFLSGFINVVFDRLLTMDTVNRVLGKTLGPDLVERLKISLHAAEAVLDDAEYKQLGDNRVRDWLNCLRDAVYDADDFLDAVLTKAATQKEVRSLLPSFFLNRHRKMVDNMEGVVSRIEFLVSQKDILGLQKTTKDNNLSSSSSSWRETTCLMEGSIYGREDDQQALIKTINDNSESRLSVIPIVGMGGVGKTTLAKWAYSVAEGFDLKAWVCISETVDVAEITKKTIEEITKNSCTLGSLNLLQNKLQEILSVKKFFFVLDDVWSEDADKWKQFIAPFHSGAKGSSILLTTRMKEVASVVQTCPSCTLNELSEESCWLLFAANACFPESNGNPTLEDVGRKIVKRCKGLPLAVETLGRFLRGKDDVKDWNVVLINDIWELKNNKIIPALLISYFQLPPYLKRCFVYCSLFPKDYNFEKTELVLLWMAEDLLRLPKRGESLDEVGSECFEELASRLFFKPAEDPADRYVMHDLLHDLGIFLAGDFCNRIEELGEQEKKKVLTRHLSRFPRGTVCPPITKFSNTIAKLESLRTSLYIDDLLSLKSRASKLKYFEFYPFVNLMYYQIQ

>arahy.Tifrunner.gnm2.ann1.777P8W.1

MAGALVGGAFLSGFINVVFDRLLTMDTVNRVLGKKLGPNLVERLKISLHAAEAVLDDAEYKQLGNESVRDWLNSLRDAVYDADDLLDAVLTKAATKKKVRSFLPSFFLNRYRKMVDKMERVVTRIEFLEKQKDFLGLQKTTKDNNLSSSSSSSWRASTSLVEGNIYGREGDQQALIRIINDNSDSQLSVIPIVGMGGVGKTTLAKLVYNTTEGFDLKAWVCISETFDVVEITRKTIEEITKTSCSLESLNLLQNKLQEILSEKKFFIVLDDVWSDDADNWKKFKTPFHCGAKGSTILLTTRIQEVTSVVQTCASYFLNELSENSCWLLFAENACFPESNGNPILEDVGRNIVSKCKGLPLAVETLGRLLRGKDDAKEWNAVLKSDIWEFSMKNSKIIPALLISYFQLPTYLKRCFVYCSLYPKDYLFDKDELILLWMAEDLLRPPRRGESLEDVGCKCFEELASRLFFKQHVFHYKMHDLLHDLAIFLAGDFYCLLEVHGKAKGATTLTRHLSYENLNHVLFDSISKVKSLRTFFPDRTSIHSLFNEIDGDTIGILIPKLKYLRVLSFASFRELDVLPDSIDELIHLRYLNLSGTDINTLPESLCNLHNLQTLILYVCTSLTMLPSGMHNLVNLRHLDLRGTLLEEMPTGLSKLKQLRILDYFVVGKYEDNGIQELGGLSNLHGSFEIKKLENVVDARQAMSARMLEKNHIDKLLLEWSSGEEIVSNKETERDILDGLQPHNGLKVLRIKGFKGERFPDWVGHCSYNDMTRVSLEYCKNCCMLPSLGQLPSLKSLHIEGFRQIKRIGDEFYKNDSGHHSSPTAPFPSLETLEFHNIPCWQEWHVDLEAFPQLKTLRIEDCPMLKGDMLNGIFLRMVSSLSDASKVCKLKIREDHQGTSHEMLRYEDSLSISGCESVVKSAFNVTSINHLCCLQEVHISDCSSAVSFPGNCLPKSLQKLTILTCPKFVFPEQQQHKYDLVELQIEGSCDSLTSFLLDAFPNIKTLKISRCSNLESVSMSEPPHAALQRLTISGCHKFVLFPEERLDAPNLTHLNVSWCSKLEALPRDMNALLPSLHSLNIHGCRKICRLPEGGLPPNLKQLTVGSQWKDLLWMGNLDALTRLSIHGRKCESIIKSFPEVGSLPHLPSLTTLHLFAFENLETLECNELLHLTSLQHLHIEDCWKLENMAGEKLPSSLLLLQIKKCPLLGEQCKKKHQQIWPKISHIPTIHVLVIFLCVRPSQNFLECLYIPISRTQPIEQRGKKLRGDANFAGVIMTNNKLGNTFENLDSISKVESLRTFLPTSYFSHSDNIDGVTSFLILKFKRLRVLSFFYSKGLTILPDSISDLTYLRYLNLSGLSIKTLPESLCDLYNLQTLKLNECSSLIMLPNGMHKLVNLQHLYIGGTCLKEMPRIMRELKQLHALCYFIVGKQEDNGIQELGGLLHLRGSFEIKKLENVVDAKQARSVRIIDKECIYYLLLKWSLGDDLVSSTERERDILDSLQPHNSLKYLRIKGYKGTIFPDWLGHCSYNNMTSVSLESCNNCCMLPSLGQLPSLKALSIRGFGQLKSVGIEFYKNEDDHDSSPGAPFPSLKTLEFQDMPC

>arahy.Tifrunner.gnm2.ann1.77ASM4.1

MNLFSLYFLTSLFPIIFARDDSQLIANVVNDVLQKRYLRHPIELKGLVGTEEICRNVELLMKRVRIIGIWGMGGIGKTTIAKVLFAKLFPQYDNVCFVVKEISVDRLLFELLKEEISTSNLVGLAFDMKRLNNKKVLIVLDDVDSLDQLEHLCRDFRDLSEDSRLIITTRNRQLLAGRVDWIYKVEKWKASESLQLFSLEAFKETHPQRGYEDLATMAVKYAGGIPLALKVLGSYLRSKSIKFWESTLRKLNKYPNETIVNLLKVSYDGLDDLEKKIFLDIAFFFNGEEKDHVISILDACGFEASSGIDVLEDKALITISYNNTIEMHELLQKMGFDIVRRECSGDFARRSRLRDTEVRAVLKDNKGTDAVEGITLDLSLIKDIHLSVDTFNKMNNMRFLRFYIPLGQSPGHVYLPRALKSFSNKLRYFEWNGYPLESLPSTFHAKLLVEIRMPHSRVEQLWRGKQELDNLEGIDLSDCKHLIMLPDLSKASRLKWVNLSGCESLCALHSLSTIRLRVLF

>arahy.Tifrunner.gnm2.ann1.78G47F.1

MILFRVDIGKRMKANRDRFLQIDEERRRFELQPGVVERLQEDVEWRQTSKKCLIVRVWNEDQDTWEKLKYTLQCGSGTNGASILVTTRLESVASIMGTFLFHHLLPLSEDENWLLFKHYAFGQDREECAELVTIGKEIVRLLRFRNDVKQWVIVKISKLWDIAHRIVKEQLVPLWMANGLIKARGNMEVEEFVSSLLISCHIAAYVQAMNY

>arahy.Tifrunner.gnm2.ann1.78Q89H.1

MADTVISFVLDNLSQLLAREASLLCGVDDRVRSLQRIEKEVLRQIRDVAHEAEDVIDTFVVNVAIHKRRTKLGRMLHGLEHAKLLHDVAEKIDSIKATVNDIRDNKIKLTDVVPQESGSSTSTREEEERVLLMHKKRRIVEEHDVVGFVRESKAVIQLLKEESSQSNVVSIIGMGGLGKTTLARKVYNSGELKPYFKCRTWVYVSNDCRVKDLLISLIKSLMPNLEHEHGRKKKGKKQKGTEKSGDLSSLDVDDLKLKVRDFLTMKRYLVVLDDLWKTQDWDEIQDAFPNNKNGSKILITSRLKEVASHTSPCPPYYLQFLGDDESWELFSRKVFRGEECPSDIEHLGKQMVKSCGGLPLSIVVLAGLLAKKGKSHKEWSKVVGHVNWYLTQDKTQVKDIVLKLSYDNLPTRLKPCFLYFGIYPEDFEIPVRSLLQKWVAEGFIRQTGTRDAEDVAEDYLCELIDRSLVQASRVNVNGDVKACCIHDLLRDLCISESKEDKLFEVCTNNNILERSKPRRLSVQCGMHRYVSSSDNDHSCVRSLFCLDPTRYVFTPSEMKWLFKLFKLVRVLDLGENCVRKVPSNLGLFIHLRYLRIRPERGSFKVPDSICTLQNLQTLDIYSFFEVIFSTYLASGLWNMKQLRHLNTNGSIILHGHHGSKAGDQVMWNLESMYYIKFNRQIAHMLEKGSFPKLRKLGLHISSVQKNNVHELLSSLQRLIHLNKLQISIKWKNARRRFPLNYKHMEWHVGVKPIELLQSLQQLSNLSTLKVLKAFDIATCDIAFPPCITKLTLTEISFMSDDGMNAIGNLTRLRHLRLSGSFAFDIFFDINCTANSFPQLHVFEMEWLNVDNWKLGNGAMPCLQTLLIRYCERLDDLPHELWSLTCLRQVKVVTSSQALSLALEKLEMKDGCELVIEKFLL

>arahy.Tifrunner.gnm2.ann1.79SUWC.1

MRNELTCAVLSSSSQFSRSNGNAPFLPIKCQRFYPHFNHNTSHQKNPNTKVQGELVIVGSEVAFSGVLMDFVSSFAASISRDLVCGAVDELRYPCCFHDLVEGLEHEDKMLVDTKKRVQEHVHHQKRQLKKTDELMHKWLNQANNLSNDVDNLLTETRTNKIYCFGKCPNLFWRYRLGKKLVKNKGDVKKCIGEGSKYMQFERLASLPGLRYFSPERCLKFESTQSAYDQLMQALNDDTVNMVGMYGMPGCGKTTLAMEVGRKAKEEGLFGEVVFVPVSSVVEIQRIQEKIASWLQFDFPQKEEIQRAQCLDKFIGENHENVLLILDDVWHRLDFEAIGIPSFKNHRGCKVLITTRSEAVCSLNDCQKKIRLKTIELEEAWKLFQTQAQITEDTSNALKKLAQEISDKCEGLLVAIVAIANTLKGKGEADWKVASDNLKRCKPVIIEGGLQNPYKILRVSYDNLDPVEQSLFLLCSVFPEDFEIPVEDLIRIAIGVGLVEEVVTFEGARNKVIVAKDKLLSFSLLLDAVGVNCVKMHDLVRDVARWIARNDGKAIECVMENNANLVDSTPIRYLWCKQFPVELDCSSIEFLCIKTDTEVSEGIFKGIASLRVLILCWEGHQRREFSTMSLKSLTDLHSLFLRCEMEGNPFEVTGKHPRIEELYINDDRPEWDLEDEDPPEFFSKFSVPQPLERNLATKAEILYIANIRGDVKSIIPGIFQIEGGGGGMNHGWIELLIKKSENIVHLVDTGKHLNEVGSLLSELRKLTIKRMKKLRYLYHGQHGSGLFVKLEELYIKKCPQLQGTLFDWKLNLCSLTALKLFECKKLTSVFTLAAARVSHLEILEILYCDGIKHVLEDDEEIESSNDDDGCLVFQKLKRLLVRGCKKIEYIIPATLAQGLLHLECLEIEDNNELKHVFGHSKHGAQKSQNALKIDLPRLGELALVKLPNIISICPRNCRATWQSLHQVALRNCPGFVIESANNCMADSKRRQDDQSIIEIKGNDSSLYEIRMKECELKAVLEVAERFIDKEQDPLMKCLQKLGCIYNALIQSMNPQNLEQMVVSGNTNAGEFQYLLDFHLESNQVENSPPSVSIVNYRFGPFNLCNLKSLILRSCFMLRTLFEPSTAISLTSLEELMIADCHELKYVVTSEIAHGNIEEITPEDYEPQSYASMFPRLKRINVMNCRSLEYILHVSFAQGLVELQEVEIRHAPQLRYIFGENINDRVHSSNDHQENVQIELPALEKMALFDLPNMTNICSGSYYATCSCLQQTVMDNVGLSTLSVNNRMVHSGATQSQSQKGKNSSHSSRNRGSAFDKTNGKMSGSESIWNNSEIEGNLHLDEVPINRQEMTSWYIWKGAKHFVTLQNVTLLYILGCQKLKVIFSPCVLRSLPQLTILVVIQCRELEQIIEEDENYEVAPNPESKKVSFSKLKLLLITHCNKLKHMFHLSASHEFPQLEYLIIKQNPCLEQVFRECEQVVGDRNGRVVEALLPELKHVILMQLPNLYNISQGIEFNNLDNLLVHNCPKLTLTSTTTAEEMLRSYYHDNVINFFVLSELLCINDIINKETVQHQPASESRGRANESDEILTSGRENKRELQSLEQIPQADLTQREFVNASRLEDKEHKSENINKEGSSQNPYHERCESKNLEEKEISYGQEHEEPKREELNKEDFIEPRCSYVLDRQHQEKEHSCSVCITQCSNDTSIVEELNSLVEAGELRAECVATLSSFLTAQPSMRLTVDNSSLIFKGFAYSSLHRLVSFLSTQPLVSLVGDKHAEFLDLIRLVGCFPFNKGWLSDLEDRVATSLPASSLSTLDKLFADKQQVLSKLEPLQDRVNALHSELAELSTEMEPLLRSLQETEAQEANILATLNYPLFQL

>arahy.Tifrunner.gnm2.ann1.7CH5H3.1

MMANQDRTIITNDYDPDKLAYDVFLSFRGEDTRYSFTGFLYDALFRKGVRVFMDDEELKGGDRIAQSLVNAIQQSRISIVVFSENYATSRWCLDELVEIVECMELKKRLLVWPIFYKVAPSDVRHQRKSYAEAMAAHEERFGNGSVKVHKWRSALSQVANLKGWSYQTGYEYEFIHEIVKRVTTKLQHEQVNLGEHLIGHQSRIEELRSLLDIESQRTVCMLGIYGTGGIGKTALAKALYNTIIHKFECSIFLEGVRERSNSYMGLVNLQEAIISKLYEGENIKLENVDDGITKLKDALKHKRVLLVLDDVDSEDQIRNLAGERNWFGSGSRIIITTRDKHVLNIGKVEKKFELKRLHDHEALQLFCLKAFKMNHPLPEYKNVSNHVVHYAKGLPLALKVLGSHFTNKSIRECECALKQFKRIQQRNIHDILKVSYDCLEDATKCVFLDIACFFKGASLQYVENVLEKCDLFPSYNIGRLVDKSLLAIENDCLIMHDLIQDMGIEIVRQEEPSKLGKRSRLCFYEDVLRVLREDSGSSNIEGIMLDPPEEENVIWGGTAFQKMNNIRILIVRNTHFIPEPSYLPNSLRLLEWDKYPSKSLPSNFHPKDIVDLSLPCSQLNLKMKKSFQRRRRRRAQRRKRMQWWWLCDCLILVATVAVGCGGDGGGLLDWWWWLCDCKERMDELLWKMLHLTYMNLSMCQFITHFPDASGVPNLKELKLNYCQNLIAIHPSVGFLKKLKHLSACHCMNLKHFSPTMWLPSLQYLNLDSTSIEFFPQTEKPMHEPLEITMQNDRIKDLPSSISNLMGLRKLYIRFKGLVPGGLPKDLFMLPEMVSLSIFDSPNVGESFKRFLIDKPGCYSYSPLRLLNCSACGLTDEDLHAILCCSRNLEELYVPWNEFVCLPTIIKESSKLRYLCVTKCKKLSEIPELPSSIKNVEAKDCPILSSKASELLWSQALKEVYRLEIIMPKITMIPIWFDHCNEGGVVSFWARQKFPVLAVAFILKEKASMGSANLYINGCNARQFISNNYYCPYVNAEHVLLFDLRSLFKDDEEWRFLDTFLIYEWNYVEVKYECDNLLLNDVVGMSNVSHCGAYVYKGQSNMEDIQFQCPYSTM

>arahy.Tifrunner.gnm2.ann1.7ERL5G.1

MAAEAVLSSVLSVVFDRMSSPEVVNWIKGKKLTQKLIERLKTNLYAVQAFLIDAEQKQIKERAVKNWLDSLKDAMYVADDLLDEVFTKAATQKDPGTFLSGFSRFLNLQDRDVANKMEEVIDRIESLVIQKDTLGLREIPKENMSWRITTSLVETSDICGREEDKEVIVKLLLDDNGDDDTGGHSDVSVIPIVGMGGIGKTTLAQIVYQDGKVKENFDFQAWICVSEEFDVFKVTKTIIEAITSSSCSLTDLNLLQHDLKEKLSRKKFFVVLDDVWSESCEDWDKLLKPFRKGVKGSKILITTRSKRVASVVQTVSPYELSLLSEEDCWLVFSKHARLSTGFMENPTLKKVGRDLVKKCDGLPLAAQALGGLLRGNSDIKYWNYLLKSDFWEHSDDKIKVVPALRISYYYLPSYLKECFVYCSLYPKDYEFSKDELILLWMAENFLQPAGKKTPEEVGDEYFDELTARSFFQPHKIRENKFVMHDLVHDLAMIFAGEFHFRAEELENAVEVDIKTRHLSHNAKGNYPISKLLEVCDRVKHTRTFLGLNLNSQIPFNMENAPCILLSKLKYLRALSFYRFPLESLPDSIGELIHLRYLDLSYTDMMTLPDTLCNLYNLQTLKLVGCWKLTALPVSMKDLTNLRYLDISLTRLHEMPEGMSKLTSLQVLSSYVVGKLDSQIERDILEELRPHSNLKQLQIWGYRGTTFPDWVGHFSYHNITQITLGGFFSGYFKNCCMLPSLGQLPSLQHLEISKFERLPIVGAEFYRNDESCLETPFPMLETLRFQSMPCWEEWRSLELNSFPRLRELIIRDCSMLRGDLPNQLPSLRSLRIENCEQLSCCVPRGPAITSLRIEGSNEVRIEELPPLLDKLSINGKHQAESVMEAITHTQLTCLTSLSISDYLSTALRSLTISYCEKQLSCVALLFHGLTHLYIEGECESVKCLPKEGWLPATLEYLRLDSIKSVEMLECKGLAHLTSLQQLSIHFCYNLENIDGEKLPASLLRLTIYGSPLLGKRCEMKDPQVWPKISHIPAIYVDKRWIWNSQKQDNHNNCNRGRKQLSNQGQ

>arahy.Tifrunner.gnm2.ann1.7G7GME.1

MADHDAESCSSHFMYDVFLSFRGFTRYGFTDRLYHALCERGITTFRDDENLRVGDRIRDTLLEAIERSRMSIAVLCQNYASSAWCLDELIQIMECSNKGTKRPVLPIFYHVDPSDIRHQKNQYDQDMKKHESRYGKDSHKVQAWRLALYEVSNLSGEHCKANRYESEIIESIVEEVLAKLPPEPLYIKHPIGFDSHFEAVESLWNIKSHNTICMLVIYGDGNKTTFAGELFNKFQHQFQAASFLDKVSEKSRGGADGLENLQKTLLYEMGVHEKVKLGSTLKGSFDIKQSLRNKRVFLVLDDVDSTEQLDALAGGGDWFCPGSRIVITTRDANFLSNQVLHGFKIERYCINEGEFDRMEALGSNQKQDKVVEEDMVGFVNIFNDVTKQLKENDSGVDVISIIGMGGLGKTTLAKKIYNNNEVKKLFPCCVWVTVSKDYKAKELLQSLLKGCGLSESIKGEDISADYQRSVVREFLETKKYLIVLDNIWEPEVWDEVECFFPDNNNGSAILITSRNDGVANYTGSKSYYPPLLDKDESWKLFCMKVFKSRECPSNLEHMGRLMVEKCGGLPLAIVTLAGVVAKKRRLPVEWTRIMRNVLWYVDKDDGRVTNVLKLSYDSLPQRLKSCFLFLGVYPEDYEIPVKRLKQLWIAEGLIQPPKIGISDGLEVEDIAEEYLNELVDRSLVMVVKRKSDGGVKSCWIHDLLLDLCISENRAEKGIEICTGKDIPSLDNVETCRLSLRGQYSNLLEQCDLSRVHSLMCFWDYCYFTETLWINVMSFSSARILDFGKPCFGLSPAAQNLGTHIHLRYLRVNQGACVEDHPDLICSLANLETLIVEDFSDRHFFKLPHGIWRLKKLRHLEGRLRMTSKTVPPNTSGEDCLPNLQTLQLVTLEKGLTMSSIAIGRFPKLRKFGLRWNFGSGCTEHELLQGLHHLKNLEELKLVDFHELPQAHEFPSNITKITINILSPTDWFGFNYRYSSLNTLGDLTRLHVLKVTTPVHYLEDSLRFAAGSFPNLEVLHVTMMRVREWILEEGAMPSLQHLIMDQCYLDELPEQLWSLNNLQNVRVVDPPRKLGESLLRVKPKDGCKVILEGQDTG

>arahy.Tifrunner.gnm2.ann1.7J0RKL.1

MSCSTKKYDVFLSFRGEDTRTNFTSHLYTALDQKSIRTYIDYQLNRGEDVWPPLAKAIENSHVSVVVFSENYASSKWCLEELVKILQCRKDFGQVVIPVFYETDPSHIRKQSGSYGKAFAKHERDLCAEGRYSDSNKHKVENWKAALTEAANISGWDSRNHNLYFLTSLFPIIFARDDSQLIANVVNDVLQKRYLRHPIELKGLVGTEEICRNVELLMKRVRIIGIWGMGGIGKTTIAKVLFAKLFPQYDNVCFVVKEISVDRLLFELLKEEISTSNLVGLAFDMKRLNNKKVLIVLDDVDSLDQLEHLCRDFRDLSEDSRLIITTRNRQLLAGRVDWIYKVEKWKASESLQLFSLEAFKETHPQRGYEDLAAMAVKYAGGIPLALKVLGSYLRSKSIKFWESTLRKLNKYPNETIVNLLKVSYDGLDDLEKKIFLDIAFFFNGEEKDHVISILDACGFEASSGIDVLEDKALITISYNNTIEMHELLQKMGFDIVRRECSGDFARRSRLRDTEVRALLKDNKGTDAVEGITLDLSLIKDIHLSVDTFNKMNNMRFLRFYIPLGQSPGHVYLPRALKSFSNKLRYFEWNGYPLESLPSTFHAKLLVEIRMPHSRVEQLWRGKQELDNLEGIDLSDCKHLIMLPDLSKASRLKWVNLSGCESLCALHSSILSSDTLATLILDRCTNLGTVKGEKHLKSLKNISVSGCSSLKEFAVSSDLIENLDLSNTEIETLDTSIGNLPNLIWLNLEGLKLKQLQKELCFLTSLKELKLSYSGLVIDKQQLHVLFDGLRSLQILHLKDCANLSEFPDNIGALSKLQELRLDGSSVRSLPTSIKHLLALEILSLKNCRELLSLPELPSFIKEFYAPNCTSLETVSNFKSFAMKMVGKTKHISFKNSLKLNGNSLYSIMESLHLTMLSAAFHNVLVRRFHVAIHSYNYNCMDACLPGSRVPEQFTFRITNSSSITVHLPTCSNLLGFIYCVVLSPSNGLKQCGAKIQCECNLAGGLKATWQDKAVSELNSDHVYLLYDPFHCDNILRFYEPKVYFEFSVTADTGEVDGSIAIQECGVHLISDSELQCVLPELEMDLDKRKDLEKGLEIESGKARWLYPYASEDHSQNLVAPPPPPPPPPPPSTPPLPQPKTRKKVRSHKEKSSSKRSRMQNPGTELCVQCCCDRKVATEEMKSEIIEIDPRSTDHFSDVEERMESNCKKIKNGDRKGLEENNSESTKAVKSKGNEERPIDFDAGLDLLVNSDSASKEYAPEDMPLQNFYDQPNTIGGSKHYKENPMLLRRQLLVHPEINDLTVEKPNQERKKERMGLDKPKVEDEPDEDPLAELESILLGRQKSLLKPACSASDVAIREALHNLECILEKSLENILGDIELQHKLQISLQCIEQASDEEVSPSITKLVKSMTSSVEDLIKSFASTQKVVEDHTSRLEQKEKLVQKMLDARKQQELVKERMKQYKIQAESVEREVEDLDEQIRFLVEQRKIIQMKRTKLNKDLEECDGKRRKLTDEAKDWVAESKELMLAINNSEVSYASAISKQEKLNEKWE

>arahy.Tifrunner.gnm2.ann1.7L0VU8.1

MHCLRYIPNFQYQIIIKLSKHLQLQNLITFSSSNSVPNYFEFAMANHDVFLSFRGETRYRFTDHLYGALRQNGIQTFRDNENLRVGDELEPILMKAIENCKMAVVVLCEDYASSTWCLRELVKIIDCHEKQGKQVLPIIYKVNPSNVWDQKGCYETAMAKHESREDPQKVKTWRLALSKVQKLGWVHYTDDMNQPEFIKVIVRDTAGRLPLPEPTDYVVGLDTRCEEVKSRLDIESNENVCMLGVYGPGGIGKTTLAKYLFDKIKRQFEASCFLGNVREKSESRESLESLQKSLLDDMGEETITEFGSVFKGGSEIKRRLRHKRVLLVLDDVNSITQLESLAGGHDWFGSGSRIIITTRDTDMVDKHMMGDVVTKKYKMEELNDDDSLELFCWHAFNSKEPAENFENVSRNAISYAKGFPLALEVIGSHLGGFESVDSWEEELDKYRIDPSIQGVLERSYNSLYELDRKTFLDIACFFKGEKWEYVKRVLKACDFYPVVRVFISKCLITVNQNGCLEMHDLVQDMGKEIVMSDSPTNPGDRSRLWSHKDILQGSSSIEGIMLQPATHEQVDHWITTAFDKMENLRILIVRNTIFSTAPSRLPNSLRLLEWKGYPSESFPLDFHPNRIVDLKLPHSVLKLEKSFQIFEDLTFINLSQCQSITQIPNVSGAKSLRVFILDRCHKLVKFDESVGFLPNLVYLSASECRVLRSFVPRMYLPSLEVLSFYFCKKLQNFPDVMQKMDKPLKIYLANTAIKEFPDSIGNLIGLEHIDISICRGLKDLPSSFFKLPKLFTLTMEGCYHLRRSFKRSEPEFIKVIVRDTAGRLPLPEPTDHVVGLDTRCEEVKSRLDIESYENVCMLGIYGPGGIGKTTLAKCLFDKIKRQFEASCFLGNVREKSESRESLESLQKTLLDDMGEETITEFGSEFKGGSEIKRRLRHRRVLLVLDDVNSITQLESLAGGHDWFGSGSRIIITTRDTDMVDKHVMGDVVTKKYKMEELNDDDSLELFCWHAFNSKEPAENFENVSRNAISYAKGFPLALEVIGSHLGGFESVDSWEEELDQYRIDPSIQGVLKRSYDSLYELDQKTFLDIACFFKGEKWEYVKRVLKACDFYPVVRVFISKCLITVNQNGCLEMHDLVQDMGKEIVTSDSPTNPGERSRLWSHKDILQVLKDDTGSSSIEGIMLHPATHEEVDHWITTAFNKMENLRILIVRNTIFSTAPSCLPKSLRLLEWKGYPSESSPLDFHPNRIVDLKLPHSALKLEKSFQIFEDLTFINLSQCQSITQIPNVSGAKSLRVFILDRCHKLVKFDESVGFLPNLVYLSASECRVLRSFVPRMYLPSLEVLSFYFCKKLQNFPDVMQKMDKPLKIYLANTAIKEFPDSIGNLIGLEHIDISICRGLKDLPSSFFKLPKLFTLTMEGCYHLRRSFKRFKESHSTLDDCPNIKRLNFTGANLSDEDLHPTIQVLQKLEDVNVSHNDFVSLPNCIERSVHIKCLDVSYCKNLKTIPELPSSIQKVDARYCLSLTSESSTELWSKVLLETERIQIVMPKKEIPNLFDCDSSEGIPLFWARRKFPVVALAFMFGRSTVSNSVAETRTDILGFFPQIGSSLSFIVRLHLFIGGKQIFPKDSKYFSVGEDHVLLFDLRALFSDEEWHDLDAYLGVGDDGWKAIQVQCESPLTLSHWGVYVYKQDTNSADIQFKNPNPKSPLSDLVPKRSPHEPQESMMRQVAENLNPRELLGDYLPLVELTEVPSFTSALLRSLRTGKAEATRPESSAYGASLKQEHEESNWNVSRVMDMIKDDVPTHIADAYSNEIREGRRFVEELMRARMELLKEKGQERMDIDMAIVLEQPRSGRPPSRRYWGRLHIKYEEITAKAIVRKTTQLAWRDWNPGRTTTKEKATAVLLKCTGQGESSEEENNDPVLAELLSQIEEDAMRFNTSYGKMKACIILTDDAYALISEEYVPEFMLIRGKENAEIKGMSQWGALELLIYGFMRASGENTFGSVESEPRFEKTPYGKIRVEN

>arahy.Tifrunner.gnm2.ann1.7Q6C76.1

MEQKVKELLQSKKYLLVLDDVWIRNQQLKFGLSEDKWEHLKSVLSCGSIGTSILVSTPIMGTSPAHDLSGLSEDDCWSLFKQCAIGPDKKGNEELINIGKERVKKCGELPFASKALGGLMRSRNTKKEWLELKGSSLWTLSNENHILPALRLSYFHLTPTLKQCFAFCAIFPKDTEILKQDLIHLWMASDFISSRANLDIEKVGNMIWNELYNKSFFQDVKINDESGKIYFKMHDLVHDLAQSIVEQECMFLEKSNLIDLPRNTHHIGFECGGQTETPIEKGSIFSKFLSLRNFSHLRYLALCGLDIKTIPDSLYSLHKLEILKLSNCGKLYSLPKNLTRLQNLRHLVLVVVIQYLICVHTFTNNRNPIGTSEGVGFSRKNSKPPSSKLYRTIFEVDATAIRRVFPQIYNGCNSAPVEGQRKLTTSLLHQVKGKNQVIQSAMPMNAIKSGGARHKYSFTITNYPFKCGILNDSLRRVRTKCTNSGFRSKRVLFMYLIYNRNKLVLVIHMIISEAANSYEILNFQRLGCQSSTGIPQHD

>arahy.Tifrunner.gnm2.ann1.7R2JU5.1

MSQVCSFFSKSVNPILFRAKLARKIENIQKEFNNVAEEMSKLNLNRSSVILKQDECGWRETSSSVLESEIIGREENKSDIVKLLKQTHPNQNVSLIVVVVGMGGLGKTALAQLVYNAAKDQNLFQKYIWVCVSENFEVKTVLKKILESLKKDAGDYFQKKYQRWIKRNVAGIELLDLLLVWNTSDVITSLNLVGAPFQSLQYLPRKKKKFAVQARALSGFM

>arahy.Tifrunner.gnm2.ann1.81Z5PH.1

MHSSLQELEKLLLLDSENYVRVVGICGMGGIGKSTLATILYEKISHQYDASCFVDDVSRIYGGYGPLGVQKQLLCQAFMEENFSTLNLSLANNLIQTRLRRRKVLIVLDNVDERIQLEKLAIKRKRLGRGSRIIIVSRDEHLLREHGVDEVFKVPFLNDENALQLFCRTAFKCNHIAKDYESLTDSALEYANGLPLAIKVLGSYLFGRNVSEWSSALVKLKEKPTKDIMDVLRISFDALDDMEKEIFLDISCFFLNGRDRGKYVKDILRIRGFHPDIGIRILIDKSLLIQRESWIEMHDLLRELGRSIVREKSPHEPRKWSRLWNQKDLSNVLRENKDLPNLTHIALKGSDNLVKIPNLSQAPNLEELNLEGCVKLVHLDPSVGSLQKLRFLNLKCCKSLVKIPILARTPNLEKLNLAGCDKLVQLDASVGSLQKLHSLNLNNCKSLVKIPILARTPNLEKLNLEGCDKLVHLDASIGSLEKLRFMNLENCKNLVSIPNSIFHLNSLDDLNLSGCWKLFKYQLLENPRQSEQLNSGQSVQSHMTSSICKTLTRPLHFLSSRRRSNSVGSLVPSLSRFPVLTSLDISFCNLVQIPEAIGQLHCLEYLNIGGNNIVALPHCIKELPKLTWLNLEYCKHLKGLPSTLLPIRRVYRGGIYVFNCPNLSDTESCGVTVISWMIKMIQVNLQCSFPKYSIRAVVPGSKIPRWFSEQNADSSIRLDPSPILDDNNWIGIAVCGTFVAHHASPQFVRRETGSLLECVFSRSFYLSVHYDVPIRFKQDLIATELDHVFLMFCSRENIIDFFSEVLKEGPCCRDGCELAIESDYPEVVEVKSVGYRWVFKEDVEQLNPTMMYGANSSSRHNQKHKFLAIQDEQLM

>arahy.Tifrunner.gnm2.ann1.82EEBY.1

MDLSSKSYKWKYDVFLSFRGDDTRRSFTSHLYHSLCQKGVNTFIDYKDLIKGEQITPSLLRAIEASRISVLVLSENYASSTSCLDELLKIIECKDTKGQLVLPVFYGVNPSQVREQKGSFAEDLAKHEDKFRDDVNKVKRWRAALCEASTLSGWHMGDGQESKFVQRIVEEILSKLNRTPFNVAKHPVGLDSPIEDIKTLLDTGSDDVQAIGIYGIGGIGKTTLAKAVYNHIANQFEGSAFIANVREISSQRSGLVQLQEALLSEILNHRYCKVGNKDRGINMIKNRLCSKKVLIVVDDADSLEQLESLIGEYSWFGSGSRIIITTRDEHLLVAHNVETIYKVNELNHGHALELFSWFAFKNPCPPTNCEKLSSHILNYAKGLPLALTVLGSHLCGRGKAEWISALAKLKKVPNKQIFEILKISFHGLEENEKAIFLDIACFFKGEDRQYVKMILDGCDLHSDNGFGVLLEKSLITIEVNKIWMHDLLQEMAKEIVRLESPSDPGKRSRLWFNEDVLHVLKHYKGSNNIEGIKLDIPESETEYIEAKALSRMNRLRILIINNVHVTDDIQYLPDELRLIDWPRYPSSTLLPNFHPRRLVYLNMSHSRIKHLWNGVKIFRDLKLVSFSSCEHLKEIPDFSMVPNLESLSLDNCRSLIKVHESVGTLDKLVTLNLLFCSNLKMFPSRFMLKSLHTLLLTGCSKLKKFPEIVGNMEHLEEILLQGTAIKELPQSIEYLSGLKSLFLESCQSLEHLPSSLQKLQNLTILDLSGCSKLQKIPKLPLNTRYIDLSNCRSLTSFPTLSSISNFIAEDFPRFYQIRFVNCHKLDMMLPEYISCEVVLPGSKIPDWFQYQSTNNSIYLEVSSGLYGKPMEVFFGAVFELDKGVTTTGLFAGKFDVIVNDIKINMSTSYFEALDSSHVWLSRLKLDHFMWHLKSMRQWNHFQISFSIFETSSKEKIGATLKSCGFHVWSNQEGYGIDHTTVRKSTG

>arahy.Tifrunner.gnm2.ann1.87HZXN.1

MVQQKFDVKAWVCVGEEVFDVLKVTKACLSFPSLAWVCSPCYSNDLDTTQNHLKNVIAGKKFIVVLDDVWSSNREGWESFLTPFECGSHAGKILVTTRLDTVASIVKTKHNEAHNLSLLDEEQCWSVFANRAWDPAKSRDRSTLEEIGRKIVKKCKGLPLAVQTLGGLLRGKDNEKDWNDVLNSEFWELSEEDSVILPALRISYFHLPSYLKRCFVYCSLYPKDFEFDRDELTLLWMAEDDENLMHGTIGELIHLRYLDLSYTSIVTLPESLSCLYNLQTLKLRNCRKLKKLPSKMQNLVNLRHLDDFLTDLDEMPRKMSKLKDLQFLSCYIAGKHEENGIGELRELTHLHGSLRIEKLENVKNSGEASNARMDEKIHLTTLYLSWSSFEESEDCDSQSEKDVLDKLCPHKDLKKLVIRRYRGTMFPDWVGQSSYHNVTELQLIGCRNCWVLPSLGQLPSLMRLEISHCDMVTKIGGEFYKGDATDHHQEIPFRSLQYLEFYRMGCWEEWESFACDDNNDAPFPQLEMLVIKYCPKLRGDLPTFLPSLKTLQIKGCEELDCYLPRAPIIRELRISGKQEARMRDLPLSLQQLTMEGNQLVESLFEAMTHTQPNLLVLKTSRSENLTSLEVSQSHSLQELTISECPKLDYIVRLPASLRELSIYRCPLLGEGIKGKDPHIWPSISHIPGIYVDGKQIRNDSTS

>arahy.Tifrunner.gnm2.ann1.88IWPW.1

MAFSDVYSPPTPPPPPLTKYDVFISFNGKDTRNGFTSHLHSALCRNQIETFIDYRIEKGGEIWEELVQAIRDSSVYLVIFSEHYASSKWCLRELAEIIELTNMVEKGHHHIIPVFYRIEPTHVRKQTGSYYSVFAEYDRNLDYRLVRQWRKALFHAANISGFEYNHHHSRTESDLIEGIVQMIVRKLDQKYTSELRSPFIHDQNYASIESVLINSQEVRTIGIWGMGGIGKTTIAAAIFQEFSPKYEGSCFLANVREESSRHGLNYIFNRLLSELLQEHVHITTPKIVSSAILRRLRRKKVFIVLDDVNTSELLENLLGVGQDYLGLGSKVIVTTRDKHVLLSRSVDHIHQVTEMNDENSLKLFSLNAFNRTHPPENEYWELSKRALAYARGNPLALKVLGSFLHSKSEKEWDNALTKLKRIPNADIQKVLRLSFNELDDTEKDIFLDIACFFKGEEKEKVIRILNECGFFADIGIRNLLDKALISIATNKSIQMHDLIQEMGHKIVCEESLKNPGKRSRVWHPDEICNILKNDEGSATIETIYLDMTERTEICISSDALRKMPKLRLLAFANNNGFGQKRNFPSLELIYLASSKRMIECPDLSGAPNLKEVWVNGCDKLTHLHPSILSLPKLSGLCVFRCKELKSLSCTTCSPSLRDVVAYGCPNLQEFSIPISKDNSHINLHLRSTALNQLPSSIVHLQNLDNFSFPISDLLMDLPEKYTKQIMLSDPNNHESDSVATLRRILPSPMFHYLKELKFDGCQSLTELPDNISLLSSLLVIRLHNTNIMTFPENIKTLPRLKIVLLCHCERLQHVPALPPSVHHFKAWDCKSLRTVSSSTSELKRQHGTTFIFVNCVNLDEESCNTILEDAIVRMDTRAKTQQLSPRLEENRNEECTVVDDDDGFLSEDNANVGKVCYFLPSRGSKLGGLFHRGSSQNSISIQLPQGSNFFGFIFYLVVPPIQPCNTGGDLDIRFGFECYLETSWGQRTHIASSSLIEWSCEFYYGYQMNLLSDHVLLWYDSQCCKQIMEIIRGRKAIDDDDKNANLEVKFFARLPNKEEVVIKECGIRWIYTNMEKESRVCRFKRSRQVFELEEKDLESDDEGEELVPPAKKFKNSLMEVESVENLRKKLEQLLHIQFDGGFRSAEIKLGYNV

>arahy.Tifrunner.gnm2.ann1.8AR70J.1

MAEIAVNLVVDKLIPLLKNEAKLLSGVRGQIERISDDLRLMRAYLRDVDAKAEMEAENSDQSRKEWVAQIRHVSIHIQDVIDLYLYKVANNDDDDHHNRRRGRRNAVAGVLCKICDLLKSCVGRHEIASEIGEIRESINRLKDARQLYGDSKAAAESSGGRSSSPQRHYLRLRANFAEEEELVGIEHAKKELNNWLSEGGAARTVIAVVGEGGLGKTTLVRNVYKQEQQKHSFDCYGWVDVSRSLKGVQLFKALLRSFGDMKGSNVDNNLHALIEDTREYLKEKKYLIVLDDVWETELWGAVELALPKNNGMIMITTRDTGVADSCKVSAKVHTYPLEPLEPENALRLFHSKAFQSGSKNLCEELMKLSEEFTKKCDGVPLAIVAIASLLSTKKERVSDWKVVYNSLQSKLASDSHLKGYHQALSESYQDLSYHLKSCLLYFGLFPEQYAIKRARLINLWIAEGFVECREHQTQQEVGEEYLAELIARSLVKVAYVNSYSRVRKCRVHDLMHDFIVKKCEEFNFCQVKKGSPFCFDKWSRRLSIGTNVDFADLRTSADRNQCSLLRSFLLYDIIDEEETISTFVNSLFSNFKLLVTLDFEKIPIDHVPETIGNFVHLKYLNLRYSNIEIIPESIGKLRNLETLDLKGTNVNDLPVEIYSLTKLCYLNVQTKAWKGVKLKRGVANLTALQKLAGVDASDVFGELKNLKQLRSLKIENVDRKDGMWLCNAIESMTNLCSLLINAKHNEILELESLTSPPPHLERLYLYGVMVPDWVFRLKNLIGLELIRFKFTQDQLCLVGRHLPELMRLYVRPFEGDELNIQKGWFGKLRSLTLADSTLKAIRIDEGSLPCLQEFFLALRNWNLEAVQVPDHVRKLMHVI

>arahy.Tifrunner.gnm2.ann1.8I7GER.1

MESLPFKPLDKVIDLALEASIRQLDYIINYKDHEQELKELITTLKSNKKTVDEKVKAAKDNVNQMTPTARDWLDKVATKLEESKEFHDDKMLESKTSCFSGGCSSGALPFLWHRRQLGRKAKKILAPAIKELNQEAPNILANISLPPAVTFADINPLDGDYLEFESRKGIIEKILEQLKDSTVRMVGLHGPSGVGKTSLVKRIAKQAGDSKLFDTVVMAIVKKDPDLLKVQQDIADGLRLTFGNIGENGRATLLRKRLKQENTFVILDDLWDELDLNKIGIPFDDDDVVSSHVTSSKKEEEEQTASENSPGSSGTSKRGCKILVTSRYKELLLGKMNVKEKLISSVPKLDEKDTLTLFKKVVEMSNEIPKFNPETLHDYCAGLPMAVIIVGRWLMKKNKLEWEGELERLKNQQGSNEVHRYMENSVKMGYDHLESEELKSIFLVCAQMYHQSLIVDLVKYCYGLGILNDVHTLRGARQSISQSIQMLKNSGLLDSSTSNDNFNMHDIVRDAALSIACKNQNVFTLRNKTLDVWPHKEQLERYSAIYLHKCHIVDGLPERVNCPRLTFFYIDCDDSTLKIPDTFFEEMEELRVLVLSGIDLQSLPSSIKCLPNLRMLCLEKCTLRDLSVLNHLRKLRILSLSGSRIKDWPTVLEGLRKLQLLDISDCFISSSTKSLSLSSFPNLEELYIENSLTKMEVKGQINHSQISALSELKHLHQLNTLDVYIPSAELLPADLFFHELNDYKIVIGDFETISIADFKMPKKYEASRSLALQLEPGMDIHSLKGVKLLLKGVVNFLLGELHGVQNVFYELNLDGFPDLKHFSIVNNNDIEYIVNSMELSQPHDAFPSLEFLSLFNLKNIKKICCSPITSSSFSRLKIIKVKMCPQLKSIFFFYIVEFLTSLETIDVSECDSLQAVISKEEGSKKVVLHKLCSLTLQKLPSFVSFYNNADMPLEPESMEKQARITDDIGIVPAEDEQSSTTSFSLFDENVEIPNLESLKLFSIKIHSIWSDRISNDWFQNLIKLTLIDCNLTYLCSLSMARSLNKLKSISISECSLMEKLFISEENNNEYSKDCIFPKLEEIQLSRMEMLKEIWPREDEVRADSFSSIISVDISQCNKIDKIFPSHMKCLSLKSLKVYLCEWVEFIFKSRLPSQQSDTNKSALLEVIDLGSLPYLKQVWSEDPKGVVNFTNVRSIEISSCNTLSNVLPASIAKDLGKLESFWIYSCRNLEEIIAYDGGSETSSEALEFPEVVSMSFSNLPSIRCLYKGRHIVECPKLKQLRMTGCPNLEIFKTESANEQETAFLSPEKVMSNLEHLTIDSKGLEWLMSNTRKYRFTSLKQLYLHTWQSHHDQTLYCFLHTIPNLQIFELIFNSNIREFVPSGNTAPKQRLGTVLLLKELTLWAREMEDIGFERDPALQRSLHRLKLQFCHRLRRLAHSSVSFTHLTYLQVYHCGELKTLMTCSTAKSLVQLTTLKVEYCDQLNEIVTKDEQENDEGIKIVFANLITIQLERLSNLTSFCSNWNCEFSVPLLEKLILRECPKMKTFTTKHITAPKLQSVLGEEEKGYWKRDLNATIQMYKVHLELSHHPDLKQVWCDKTWVQVNRFQNVKSLIVKKCGYLVHVIPSHLLHCFKNLEDLHVSDCHGVQVIFNMDESNNKQVTKATGLPRLKKLSLKNLPNLEHVWDKDSREIIHFSALQDLSIDKCDSLKYVFPTSIAKDLAMLNNLSIKDCEQMVKIFVELGDTKTFELPSLASLVLRELPMLKHFYPGLHKLQFPKLKELYIQVCKWMILNCQEAEIFVDQQVLLPIEKVNLLFQSLEKLSFDMRGAKLTWEVKSRKLEFEESEERVEEVLFEKKPKADYVQLLLHLALKGLSISSLYKLKSIGLDHSWIHPILDNIQTLEVKYCYDIKNLVASKVSFSSLTKLDIYRCDDLLYLFTSSAAESLSQLKHMQISDCKSMREIVSNDESSGNIIFERLQILHLEELPMLRWLYSGKRTLCFPSLQHLSIFGRILWRMTTFCPHIHINPDSVKLHSGRSKYESYGVQWEDDIIGLRENLYVQDMWRDSMPVSEAYFGNLESLIVHQCEFLSEVIPLHLLPFLNNMRILEVQKCSSVKTIFDVKCITKDKTLLPIKFSLEELVLEKLPNLDSIWNEDPDGVLDFQLIQQVRVDTCKSLTSLFPKSVAKDLVNLKNLELKHCKSLVEIIVGIATTPERAISNLIKFPRLTSLTLLDLPSFNCFYCSLHCVLLKTLNGHDPQIEDQVCFKEITPKLTNMLFGEREAKMIGHEECDRSHFGDINVSDMQSFNVELESDELSYTFLQKVSCIKTLQVKNSSIKEIFCSERPSLDCAPFLPHLKELKLASLSELIFIGFERSWIRESSILKTLVTLEVKSCSGLTSLVSSPVCFSNLTHLTVSECDSMIYLFTSSTAKSLSQLKVMKISNCKSMQWIMFNEGEELIHEEIVFEQLHELQFKSLKSLRRFYNGDFALIFPSLEELKVIKCNRMESFCEGTINTNKLSEVVFDDSFNGVRIPLEVDLNSTLRNTYKTEVATFVREVKDLKLNEHPIIHGIWNGPFQVPSLCFIRLKILIVEKCEFMCIVIPSHILSLLCKLEELVVRQCESVKTIFEVTREAKDTKINPLRSGLKKLTIEQLPNLEHVWDSDPTQQVCYFESLQEVYVHGCNNLQRLFPISVAENLNKLEKLEVTECDRLVEIVAKDEAIIEGAAKEFALQSMTSIKLRSLPELECFYPAPYKLECPKLEKVHLFHCDKLKTFQFESQEFQCPQAENQAIFLPEKVIPYLKFLAVSKEEITMMLHVNNLSKLEALQLQCFHDDSDTLPYEFLQRLPNIEKLVVCCSSFKEIICTKRPTMDCVKEIIPQVKCLQLSSLSQLNSIGLEHSWVQHISENIEKLQMDKCHSLRSIVPSEVSFSNLIELNISECNGLVNLFTPSTSRTLQQLKNMSIENCESLKEIVSEEEVEESSGHVGEEIIVFHKLKTLSLCSLPNLGRFYNGNIGLKFPSLVHLLLIDCSKMESFCAGTVSVNRWMEVQFKDEEDIEVVPFDDMGEKHAFLVEDDLNCAVRKVFEGVCI

>arahy.Tifrunner.gnm2.ann1.8IB5J3.1

MAEQIPYGVATSLINKIASLAFREIGKIYGVLDDLEKLKDTLESIKVVLSDAELKQGQNATVAHWIKRFKQVLYDADDLLDDVFIQDLRRKRMSQVRGFFSKSVNPVLFRAKLARKIENIRKEFNNVAQDMSMLNLNPSSVILKQDECAWRETSSSVLQSEIVGREENKSDIVNLLKQTHPNQNISLIVVVGMGGLGKTALAQLVYNAAKDQNLFQKFMWVCVSEDFDVKTVLKKMLESLKKDAGGDSLEALHQNLREELNGQNYMLVLDDVWNEDHSKWSDLRTHLMCGGQGSKLLVTTRSTLVSQAMGIDEPYVLSGLTDEQSWTLLKNLTFGEDSSRMSSELQTFGEEIAKKCRGVPLAIKTIGGFLRIRVEEIDQWSSLLHGDIWRLCEEEKSIMPVLNLSYRNLRPELRQCFAYCCLYPKECFAYLASPIETQSIEDVGNQYVKILLMRSFFQDASTDEYGHIESFKMHDFMHDLATLVAGNDCYLHTEGKGIVERPMHVAFETSTDCLLDVFDVCKLRTIITDTNFVAKLSFMEKLKCLRAMILSFYSMTELPKSIDKVKHLRYLDLSYSQNLRSLPESIGNLVCLQTLKLKGCESLFSLPESIGNLVCLQTLKLKGCGSLVSLPESVGNLICLQSLNLNDCGQLVFPTKMITKLINLKKLDIECCKAFEDGMPVGLGKLISLQSLSRFVVGNNKKDTSGKLNELKELDLRGRLTIRELGLVKDAASESNETNMRSKKHIQDLSLLWGIRCSDSTEYEIRKSESLVLLDNLCPHQNLRSLDVNGFPGVRLSDWLISLIHVVRISLFILPNCKHLPPLERLPCLRELKVDHMKSLEWMDYYEIIGDVFFPSLEKLVIWGCENLRGWEKNANETQNHLSLPPFPRLSLLQIHNCPKLTCMPSFPNLVVLYLGLGSSVKPMLETCMVKHDSSSISPLSHLNHLYLTEVTEIEAMAEDWMKNLTSLQSLDLGGSSAIQILSRHLQYLPSQLQQLEIGFHDDKLDLWKHTQGRGPPHALSSLQTIIFSRCRNMKALPEQIGNLQSLRLLEISYCPKLETLDEADRCLPNIHSLRIRYCPILKRKYDLEKIGPRLLTSQTYPYTEIDTLKKGILANHFLLGILSNFNRPMSAGSMQRKLHSATSQLPLASNLFRCLERFDR

>arahy.Tifrunner.gnm2.ann1.8TU2MJ.1

MAAALVGGAVLSSIFNVVFDRMSSPEVAKWIKGKKLTQNLLERLKTSLYAVQAFLNDAEQKQIKEKAVKAWLDSLKDTMYVADDLLDEVFTKAATKKHPESIVKQKETLGLREIPKENMSWRITTSLVGSSNIYGREADKEAIVKLLLDHDDTGDGDISVIPIVGMGGIGKTTLAQLIYNDDKMKGNFDFRGWVCVSEEFDVTKVTKTIIEAITKSPCNLNDLNLLQHELKEKLSTQKFFVVFDDAWNEDYEDWNRLLKPFQNGVKGSKILITTRSKKVASAAQTVSPHELSLLSDEDCWLVFSKHARLSTDSMENPTLRKVGKDIVKKCDGLPLAAQALGGLLRENSDVEYWNHILKSEIWKFSNDKIKVIPALRISYYYLPSCLKDCFVYCSLYPKDYEFDKDELILLWMAQNFLQPAGRNTLEEVGDEYFDELVARSFLQPHSTEKNKFVMHDLVHDLAMMFAGEFYFRAEELQNACEVDIKTRHLSHNANGNYPISKLLGVCDRIKHPRTFLEINLNWRIPFNMENAPCILLSQLKYLRALSFNRFPLESVPDSIGELIHLRYLDLSVTYIVTLPESLGNLYNLQTLKLYWCRYLKMLPVGMKKLVNLRHLDIRETGLHEMPKGMSKLKNLQFLSDYVVGKRRENKITELGALSNLHQSISISKLENVVNSSEASMARMFNKDGIRYLKLSWSPDEDENEVDSQIESDILDELQPHCNLKELKIGGYRGTIFPDWLGHSSHHNITKIKLGDCRNCVKLVWRHHFQCLRLSFESMPCWKEWRSMVLNAFPRLRELIIMECPMLRGDLPNQLPSLESFKIDYRNELSCCVPRAPAITTLSITGKHLVGSVVEAITNRQLSCLTSLCISHCSSHIWFPVSAIPPSLQDLTILDCREFQMDGQHNSLQKLWIRESFDSVASFSLLDSFPNLVTVEIEECEKMESIVVSRSLSSLRFLIIKNCGSMKSVSTIWMAAPQLESLSLVGCPEIDLPAPGVPHSSLRYLGISYCEKLVISAAFMNSQFHRLIGLWIHGECESVKCLPKEGWLPASLESLTLESMKSVETLECKGLAHLTSLQKLSIYKCLKLENMEGEKLPASLKQLNINGSPLLGKRCEMKDPQFSFGAREYFLCQGLVVARLYSIPLKDTAHMGNNSIFSYPKAELRLRLLISKEGSALI

>arahy.Tifrunner.gnm2.ann1.8YN2ZH.1

MAAKLEGNDSDLELLGRLVDCLCDVGPVLDDAELKQFSDERVKKWLVDLQDALYMADDLLDELSTKASTAAPRDPGNSNDWSRPVDSIIEDSGVNVIEKIVAKLESVVGRKGKLRLRESAKVDFSSWRIPSTSLVISSDVFGRDEEKEKIIKLLLDDTCDAESRVTVIPIVGMGGIGKTTLAQLVYNDAKVVGKFDTRAWVCVAENSDPINVTRTIIGALNSSPCTMDNFDSLQTVLMKKLTGKTFLIVLDDVWDDRRDMWEDFLKPFWFGNNGSKILLTTRSENVASVFAANNLYHRLSLLSEEDCWSMFLKHSSISSSSKQYATLEPIGRKIVEKCKGLPLAVKTLGGLLRNKYNEVDWENTLECEIWELSEVDCKIVPALRVSYYYLPSHLKRCFVYCSLYPKGYEFDKEELILLWMAEDLLRPKKANTLEMTGCTYFDELVARSFFQPSNTNGKLFVMHDLMHDLATLFAGKFYFKLTEFGNQHMIDNKTRHLSNTRAFVDSIELIREAEAIHMRTFLDFSLLAYCDSVNFQRFLQHLGCLRVLSFKNLSLESLPDSIGELIHLRYLDLSYIYSMTLPESIFKLYNLQTLKLRNCHMLKMLPSRMQELVNLRHLDIRGSSDLEEMPKKMSKLKHLNFLTDYIVGKHEENGIRELGPLDNLHGSFCISNLENVKNSVEALEAKMGNKKYINTLKFKWLPNGDNDDVETERDILDKLQPHQNLKELSIKGYPGERFPDWLGLCCYSNMTKLSLDSCINCCELPSLGQLPSLQHLEISKLDGLEKIDSEFYNKNNASFQEKTPFRSLETLKIEYMYSWREWHFPDEFDGFPQLRILEIRSCPVLRGDLPAHLPALEELIIVGCEELACSLPRAPKLHELHVLNSGFYTDATTHNVDITGTQLAKSVLEWLPHIQPPRVQHLFISHCYSAISISADYVPASLQYLVICDCPKLTFSEQLHHKSLTEIDVYDCDSLTLFPLGALPNLKKLAIRGCKNMEYVEMPQTLPSLCYIWITECPGLVSLPALALAVPHLQELYIRNCPEINCFAGECLPPSLKTLQVVKCQKLERWITSQGLQSQGLTRLILHEWNEVKSFPGEGSLPASLVCLKLSTFSNLETLDCKGLHHLTSLKNLAIRNCRKLENITEEHLLASIENIYLGEECPLRRKLEEMEDPHIQFVTEKNKKANLFLSLLCCLRYLHGLMQWITVNATIDSDFVPSNICPHGRLKSQHKFMAEAMFMNGATCYTNESLESPIMSSEEFASAAKKANNQELWDYLMMLLIVNLTSKYFDFLYRYWVLRDPESSVISSLKLLNLGLAACVMRKNW

>arahy.Tifrunner.gnm2.ann1.8Z23MG.1

MASLSSLSVVSESKYDIFVSFRGVDTRRGFLSHLIKALNQKQIEIYVDYKLREGTQISHSLLTAIEESEISLIIFSQDYASSKWCLDELVKIMKCRKEKGQTAIPIFYDVDPSCVRHQRGSYVDALSGHRKTSSAAQLLMWKEALNEAANLSDAALIDEIVKRVLQRLNEKCQGDLQGLVGMVCHEFEGLCFLDNVREKVQKYGINHLKKELVSKLLDIKDVEKDVVSFIMPIGITDFAKRRLRRRKVLLVLDDVSDSKQLEDLCGGHDWFGLTSRIMVTTRDKHVLNRADHIHEVKALNQDESLELFKLNAMKQNYMDTEQVEQSMRVLNYAKGIPLALKVMGSFLCGKSKQDDDLLREMGREVVREESPNDPGKRSRLWDSTDIYKVLKYDTGTETIESITLDMSKIDMLSLHPQIFARMYKLKFLNVHSWDGEHRLCAPHGIESLSDELRFFQWEDYPSKSLPLSFCAENLVEIIMPSSQLEKLWEGVQNLVNLKLVDLNYSSHLVELPDFSKAPNLEEVNVSNCKSLQQVPQSVLSSQKLGYLFLNDCNELRSFQHNIHHQSLKTLSFHSCIGLREFSLTQLNHGTDKLVLSAPLERLKLDYCSNLSLLPDNISMLSSLKDLSMCHSSVRSLPESIKHLSRLKYLNLSNCEKLQSIPELPLSILVLIAVNCTSLHTVFMAEPIKEENFGYKTFAFTNCVKLEKATIQAIMANAYVRIQQAANAWLPTAQRNEYYYANFDNNYELRVHVCLPGSQVPGWFSYRTTETCMTVELVLLASSISMFRGFIFCAVLPPREEKGLGSESLKCSYFIETSDGPSVRDQSSWVIYDRAVDKESDNVYMWYDRQCCIDMMRRTKQDKVSDEDNVHKYKVLFDFHYSLNSTGIKECGVHPIYVSDTKSKRRPEITQVDENQGDLELEEPLSSTNRNFKMEYFSSRELSATQLSSKDDQEQNGKITPFQFMYRRRVKKKIKE

>arahy.Tifrunner.gnm2.ann1.910YMC.1

MAEALVIGALVSGFANVVLDRLISSEFVNLVVGKKLDRKLVERLKTAILAAKALAADAEQKQFGHELVREWLDNLKDALYTADDLLDRVFIKAQIRSKVRTRLPHFLDLSGRKMVTKIEEVVERIEDLEKRKDTLGLKEIPAGSSSSWRSPSTSLVKGNVYGRDGDQQALIKMLHDNNDHKLSVISIVGMGGVGKTTLAQWLYNNEDLMKGFDLKAWICVSENFDVVETTKNVIKGINAESNGSSELEGIGRKIVKKCDGLPLAAETLGRLLRSKHDVKEWNKILLSDIWEFPMTDSKSFFNPERRHSVTIWHIVKNKYLRVLSFDKISILPDSIGKLIQLRYLDLSRTAVKILPESLCKLYDLQTLKLEDCSELTMLPDGMCNLVNL

>arahy.Tifrunner.gnm2.ann1.957ZEW.1

MVGIYEDPKIGKSYITTFAFELYYKIKYKFQAAGFLVNVSKQLMKITDNLLENLQKELLSDMDVETMQELEHKKMLLVLEGVDNKDHLELLVGMGIGDWFGRGSRIIIATENRYLFRYCPPVMNGVKLEKHCIHEGELVKEKIVKEKNVVGFVKDFIDVINQLREEDSKGRNVVSIVGMGGSGKTTLARKIFDSKLVTELFPYRAWVIVSKDYRENEVFLSLHRSLMPSTTPIPNNEEVLKANVRKYLNRKRRKYLVVLDDVWDTQVWDKIKDYVLPDNNNGSRILVTTRNHQVANYARSKEPHHELSRLNEEESWELFCSYVFCGEECPPDLEPIGKSIAESCKGLPLAIKTTAGIVAKRERSQDAWQKIKNLIPYWRVAEDKDGKKMMEILKLSYDDLYEKMKPCFLYLGVFPEDEEIWVRDLIHLWIAEGFIEPIQTGRSKAPPPEPEDIGEQYLKELVDRNLVQVTSRKSDGKGVKTCRIHDLIRELCISVSNNSNNARRLSFPNDVGSYACSVTCNQSRTCSLFAYGDIAGWSHHVPEDCQVNVLYLKGVDWNLSSKENSEYLMRLKSLRFLKMDYTAPRGLFNKLQSLQTLRVLTTLEYEKTIDEGLRHLRHLSCASGVHLLEKEGVKYKMQNVQTLCDVYMDSRLGFLLDNGYFPNLRTLGLIPMSEDQGQSLRNLHRLSKLHKLKLQFGVYSRRVPLDKITFPSNLSKITLSWFEDLKYQDMNALGQIPSLQILKLYQVKCTKKALNCGGAGSFPQLQVFIMISVKVTRVTLEDAAMPCLQRALFRECRGLKSKYLPERMLSLGSNLHFIEPFDDDDDDIVDEDFNFDD

>arahy.Tifrunner.gnm2.ann1.9AG3RK.1

MRLFIFPGNGPDSASTQPFPAKLKSKVNGGTVFPLQITRVKFQDLPPVNNFNTSDRVLDLDPKVNRPVEDSGSSRLNSPLPALQYDDFISIRGEDTRASFTSHLFKAMSRKQIVTYTDDLLHERDSITFFLLRAIEESCLLLVVFSGNYASSKWCLQELVKIMECKKEFGRLVIPVFYNVDPSHVRYQLGSYSETFKKHWQNNKKAEVQKEALTAAANLEGLDSRSYRAKIEFIQNIVKDPPNDSKNLVGISENLEKVESLLSESVEVRIIGICGIGSIGKTTLARLIFEKYSYTFEGSCFLENVRERSRNYGLTELRRRLYLELLQGKFWQNNTGKSTFVEDRVSKQRNFIVLDDVSSLEQLDYLVQKLQWCGAGSKIIITARDKNVLVPTVETMYEMKILDSHESFKLFSLNAFNEDYPQIGYEELSWKAVGCCKGIPLALIALGSFLHSKSKTEWYSALQKLEKTPDPEIQNILRLSYAGLYEEAKQIFLDIACFFKRELVEYIVNLLDSYGLYAAIGMRSLLDRALIAISHNCVRMHDLIQELGWDIVCQQSSGNPENRSHLWDSNDIQDVLGNNKNLVNLEEVDLTDSQKLVELPDSSKADNLKSVHLSGCRSLRHIHPSLLSLGKLKLLDILNCAKLERLETKMHSKSLKHLYIKSCTSLSYVSHFSSLQKLLLDGSPVETLPMSIKHLTELKTLSLKGCKMLQYLPELPSSIRHLTALDCIML

>arahy.Tifrunner.gnm2.ann1.9C08TH.1

MAAKLDGGAYLTSFVDAILDKLSSILEDDSVLDSVLELLERLQNSLYDAGPVLDDAEQKQFTDKRVKKWLVDLQDALYFADDLLDELSTKAAIAATQGDPGNSSSWSRHVDSYIDDSGVNVIEKIVGKLESLVKRKAKLGLEKSAKLDTSWRIPSTSLVVSSDIFGRDKEKREIIKLLLDDSESPLTLIPIWGMGGIGKTALAQLVYSDAEVVGKFDTRAWVCVAENFNPISLTRTILEKITCSSYKRDDFDSLQTHLKQKLIGKTFLVVLDDVWHDQQDIWEDLLKPFRYGNHGSKILLTTRSEKVASVVATTDLHYQLSLLSNEDCWLVFSKHARLSADSMKNPTLQKVGKDIVKKCDGLPLAAQALGGLLRGNYEVKSWNHILKNEIWKSSNDKIKIVPALRVSYYYLPSCLKKCFVYCSIYPKSYEFDKDELILLWMAENLLQPIGEKSLEEVGDEYFDELFVRSFFQPHSTNEKTFVMHDLVHDLAMIYAGEFCFRAEEHENAVEIDIKARHLSHNAKGNYPISKLVGVCDRVEHTRTFLEINLSPNIPFDMENTPCILLSKLKRLRALSFKCFPLESVPDSIGELIHLRYLDLSGTYIVTLPESLGNLYNLQTLKLIGCEKLKMLPDGMQNLVNLRHLDIRATCLRKMPKGMSKLKSLQFLSDFVIGKHEENKIKELGALANLRESISIDKLENVVDSSEAWEARMFDKDGIDSLKLSWWSHKSMEEVFAFAVKKKDAADSQIERDILDKLQPHSNLKEVEISGYRGTTFPDWLENSSYHNITTLTLQHCNKCSVLPSFGQLPSLKHLTISDFKSLKTVGAEFYKGGSCLETPFPVLETLTFCSISDWVKWDSMEFNAFPRLAELTLSDCPMLTGDLPYHLPSLQSLTIDNSKDLRCCLPIAPAMASLNIVGGGEVSISELPPLLRKLSIHGIDQVEPVVKAFRNMQLTCLTSLCISDCSFHISFPVSSIPASLQELTISGCAKLELEMDGHHKSLQSLSIIYYYDSATSFSWDAFPNLVRLNIRKCKEMESIVVSRSLSCLRSLHISSCQSLKSVSTLWMAAPQLEDLKIVDCPEIELCPTGDGHPHHSLRSLTINYSKLISCAAFMNLQFYGLTHLRILGEYKESVKCLPKEGWLPASLESLRLFNIKSVETLECKGLAHLNCLQQLSIYHCSKLKNIEGEKLPASLKQLIIKGTPLLGKRCEKKDPELIHGANVTTSAAMRGMLRSKTTNFLTRFKVPAKIETFTHNGLLTITAFIKLLSKQGRATIMDTIQDIQERHESD

>arahy.Tifrunner.gnm2.ann1.9PBB9R.1

MANDDVFLSFRGGTRYRFTDHLYHALRRNGIDTFRDDENLRVGDELRPVLMNAIENCKMAIVVLCENYASSTWCLDELVKIVDCHEKQGKQVLPIIYRMKPSDVWEQKGSYETAMAKHEDRYGKDSEKVKAWRLALSKVNTLKLIKWLHCTENTYEPEFIKNIVRDTADRLPLPEPIQHVVGLDTCCEQVKSVLNIESNENICMLGIYGPGGIGKTTLARCLFDKIKRQFEASCFLGNVREKSESRGSLESLQKTVLYDMGEETITDLGSEFKGGYEIKRRLRHKRVLLVLDDVNSITQLESLAGGHDWFGSGSRIIITTRDTDVVDKHVMGDVVTKKYKMEELNDDDSLELFSWHAFNRKEPAENFKNVSRNAIRYAKGFPLALEVIGSHLGGFTSVDSWEEELHKYRIDPSIQGVLEISYNSLYELDQKTFLDIACFFKGEKWDYVKRVLKACDFYPIVRVFISKCLITVNQSGCLDMHDLVQDMGKEIVMNESPTNPGERSRLWSHKDILQVLKDDTGSSSIEGIMLHPSTLEEVNYWINTAFDKMEKLRILIVRNTVFSTAPSHLPNSLRLLEWKGYPSESFPLDFHPHRIVDLKLPHSALKLERPFQVFEDLTFINLSQCQSVTHIPDMSGAKSLRVLTLDKCNKLVGFDESIGFMPNLVYLSASQCSKLKHFVPEMYLPSLEVLSFYFCKSLQSFPEIMQEMDKPLKINLVNSAIEEFPNSIGNLTGLEHIDISKSKRLRYLPGSFLMLPKLFTLTIDGCSLLGRSFKRFKGHSSPNIRRLNFSGANLSDEDLHPILHVHQKLEGINVSNNCFSSLPKCIEGFLHLKILDVSYCNNLMIIPELPSSIQKVDARYCLSLTPGSSTMPLSKILQETKRIEVVMPKKEIPNWLDYNCSKDIPLFWARRKFPVVALAFMFGRATGNDIDETLMKALDFWPQIASSKSYTVGLNLFIEGQQIFPEKSKYFNVGEDHVLLFDLRTLFSDKEWHDLDAYIGDDWKAIHVVCESTLTLNHWGVYVYKGETNMDDIYFKHLPNHKLSSDLVLERSPQQTRQRIGQVIDNLNPTETFDNEHLSLVDLEEDSMSFTKALVRSYKMAKANEASSSSNYGASLKKESEESNWDVLRLIELMKENVPKDIADSNPGQIQAVQEFAEEFIKARAQFMMEKDCDTLNIGMMIALEEYHIFGPPTRRYWGRIDLKHADDPTFKAVMRRAFQESWRIEERAKAHNRRMIPILLLKCEIPSIWGESSQKEESIDDPVLEELLRQIEKDAMECNKSYGKLKASITYTNELRAVSDEYLLEATYMRAHENMENEVMSEWGRLELALCDMFRSRMRRSLFGWRKLMIPGASTKKTSYGTIRVAHEDQDTHDNNRMQHNKLWRIQIPRYFRRSFFLFFYFLLRFLYSAFLFFVRALSFTFCFTVLVLLVLLRSFMFIFVFLFTFIIMVFFMHFHLILLCLVLYFIFYCLRFLFLEIWSVVLGMV

>arahy.Tifrunner.gnm2.ann1.9SS8DR.1

MGDSVVTIALENLSGLLANELISLSGVNDQIRYLCKQLEFMNVFMKSTKGMRDNESVREVVNKIRDVAHKAEDMVDTYVVNANKQRGRNMLVKYFFHRKNHVIMLPEVNDKFEAILSSILEIREKINENMFIYGIQQRNKEFAKDSLIARRRDVEEEEVVGMVRDSDEVIRQLEGGDLSREVVCILGMGGLGKTTLARKIYNNDKIKSMFPYCVWGFVSNYYSAQELLMSILKLLDLPEEEYKYLTNPEQMKRKIRESMSGKKYLVVLDDIWYTQVWDELHEAFPHDNNGSRILITTRMENISRYTNATFTYRLSFLDETQSWELFCYKVFGKAGCPLELEPAGKKMAKACKGLPLAIVVFTGMVAKKERSLREWNRIQNHISWYHAHEDYRIVTDILKLSYDSLPRTLKPCFLYLGVYPEDYEIPARTLCQLWISEGFIQATEAGPSNSQEVEDIADMYLDNLVERSLVQVASKRSDEGVKTCRVHPLLREICISESKENKFMEVCEELDANRSDSRRVSLQYKGECLPTTGDKWLARSLLFFGEETRWERESQGWKQIRNGFNLARVLDMNQVVLGLSPRGLKTLIHLRFLKVTASSRRIGNDVLASLCNLSSLETLYLWLRDEVTLPNKIWKLKSLRHVYLNFDNIILREWTRKESMPRIIRQRAKRLQIWSKGASMSEMKIGETKVENLQTLNNIYLNARTASVLNKGMFPNLTKLTLRREPTKLLEPEKEYLENSLMCLNKLRRLKLLCIAKLPLDPNAYPISLTKITIGYFGKLDAGIIKTLGQLANLRTLKLVGGSINGDVNCVAGDFPQLQVLHLSDVQLAGRWKVEEGAMPHIRYCINPHIHD

>arahy.Tifrunner.gnm2.ann1.9W4GU5.1

MVEAVVSFAIERLSDLLVEEANLLRGVKSNVEKLQTELRRMQCFLKDAERRQDQDERIQDRILEIRRLAYDAEDVIENYAINVGTKNPLYKGTYLHKVGSEIMSINSKISDLTRSLQTYGLEERDREKLHIEFENQKELRWSYSHIVEEFIVGLEEDIAQVVEWLLNQDQHSRTVFICGMGGLGKTTLAKKVYYHNAIRRHFDGFAWIYISQQCKKREVWEGILIKLTSPTKEERDEIMKLRDEELAKKLYKVQQDKKCLIILDDIWSNGDWDKMKHAFPLENTRSKIVFTSRNKAIALHVDSKGLLHEPKCLNEEASWALFQKKAFPRNDDPEFTVYNDFKRLGKEMVLKCAGLPLAIIVLGGLLATKDTVSEWETIHKYIFSYLIKGEELERQSRLAEVLDLSYNDLPYHLKPCFLYLSQFPEDSEIPTNKLIQLWVAEGVVSSQYETERGETMEDVAERYLGNLISRCMVQVGQMGSTGTIKTCRLHDLMRDLCLSKATKEQFLYIIGGVQQNSTTNSASSSSLSDARKTGGVRRLAVFLDQQVDQLIPHDELVNYRLRSLLYFHEKKCRLNDWPIIKVLFAKFKFLRVLDLEGIKGQKGQSLPKEVGNLIWLKFLSLKRTRIQILPSSLGNLENLQTLNLQTVSKVSWDSTVEIPNVISKLKRLRHLYLPSWCGNDDNQLQLEHLTNLQTLVNFPASKCNVKDLLKLKKLRKLVLNDPRYFEQFCEIFNPPNDRLECLESLSLRTDMLSFPDKAVNLEKLVLGCPSLRKLKIEGRVERLPEASLFPPHLAKLTLWGSRLMEDPMVTLEKLPNLKFLNGWEMFIGKKMACSQNGFPQLKSLVLRGLPNLEEWTVENQAMPNLHRLGISDCNKLKTVPDGLKFVSGLREIEIRWMPKSFKTRLATDGEDYHKVQHVPSIVFLN

>arahy.Tifrunner.gnm2.ann1.9YX67Z.1

MRPLYESEDLELFLARLGEDHKTPATLSPRILEIARFIARECGGLPLAISVMARTMKGVDSIRQRRHALNRLERGEMGGEMEEEVFQVLKASYDNLRHKSMQKSFLHCALYSEFWKDDKLIKKLVDSGAINGRRSLEEIFDEGHTIFNELEDHSLLSDITNMQNSVRNMACHILKEYKRLIVRCGKELTGIPHLQEWTADLELVSLDENEIKEIPAGVSPKCPMLSTLILSNNCISSIPECFFTHMKSLAILDLSHNRSLTSLPDSLSDLTCLVSLLLDECEALEKVPPLGRLQKLSRLVISDTQVEEVLGLEMLTNLRWLDLSYNKKLRLESGRVLRGLTNLQYLNLFKAALLNVEIEDVQGLTTLEYFVVGFDDCKSYNNFVTGICNTGSVPKSYLLYLGSIEGYYESIESYDDIGPSGDHQRIVRLLDCKESPHLLSPHLLPNDLTNLYIDYNARWESLCDALSNNAPSSLENVQIHSCSEMKSFNLNNLESLQLYALESLTVICKEDVGATDTTTQQPLKPNAVFSQLRHLEIRNCHEIETLVTAGLLAQLQNLQTLIVESCESLREIFAASSSGADSDDAASTVITLPNLTTLELSDLPMLETVYKGIIISESLPNLETEDCPKLESRFPFEVSSS

>arahy.Tifrunner.gnm2.ann1.A0R4E2.1

MASKLEGGAYLSSFVDAISNKLSSILEDDSALELLERLDEILCDVEPVLDDAELKQLSDKRVKKWLVDLQDALYMADDFLEELSTKAATATPRDPGNSNDWSRAVDSIIEDSGVNVIEKIVSTLESLVKRKDKLGLEKSAKLDTSWRLPSTSLVSSDVIGRDEDKENIIKLLLDDTCDVESLVTVIPIVGMGGIGKTTLAQLVYNDAKVMGKFETRAWVCVAENPDPVHVTRTIIGAIDSSPCILDNFDLLQTNLKAKLTGKTFLVVLDDVWHDQRDMWEDFLKPFRHGNNGSKILLTTRNENVASVFTPNNLHYRLNLLSKEDCWLLFLKHSSVSTNSKQYTTLEKIGKKIVEKCKGLPLAVKTLGGLLRNKHNEGDWENVLKSEIWELSNSKIVPALRVSYHYLPSYLKRCFVYCSLYPQDYEFDKDNLILLWMAEDLLQPKKNNTLKNIGCAYFDELVARSFFQPSSTERGLFVMHDLMHDLATFFAGKFYFKLEGSENLHGVDSKIRHLSFSSISWGIITSFGEACKRAVHLRTALDFSWYRQSIDVESKPWLLQQQLRVLSFPIKSLPESIGELIYLRYLNLSGACIVTLPESICKLYNLQTLILGTCYELEMLPSRMQDLVNLQHLDIRGVSRLKEMPKKMSKLKHLNFLSYYIIGKHEENGIRELGTLDNLHGSFHILNLENVKNSDEALEAKMGNKKHLNTLKLQWFPLGYIYTDHVKILEELQPHENLKELSIEGYHGETFPGWLSLSRYSNMTKLSLNDCENCCELPWLGQLPSLQHLKLSNFYSLVEIGLEFYNKNDGSFRQETPFKSLETLKFHKMFSWEEWHFPDGYDGFPKLRSLSMRYCLKLRGSLPAQLLALEELAIVECSKLVCSLPRAPKLIWHHTRRLTRLYLCEFDVKSFPRGGCLPASLESLRLWDFPNLETLDCKGLHYLSSLKYLAITYSEKLQNITEEHLLVSIKEIYIGEECPLKSKLEEMEDPRIQLGCYELDSSNGAVNGMIKYEEVVIDNCCMMFVKNPALFDVLVMPNLYGDNISDLCAGLVGAATLVRGIALAEAVYGSAPDIAGKNMGFECLFHEKFGKSNCFTAEWCSMLRHLNLHDKADLIQNAILNTIAEGKYQTADLGGKAKTIEFTNAIIDHL

>arahy.Tifrunner.gnm2.ann1.A15F74.1

MAAELVGGAVLSSILNVVFDRMSSPEVANWIKGKKLTQKLLGRLKTTLYAVQAFLIDAEQKQIKERAVKDWLDTLKDAMYVADNLLDEVFTKAATQKDPGTFFSRYLNLQDREIANCMEEIIDKIESIVKQKDTLGLREIPKENMSWRITTSLVERSNIYGREKDKEVIVKLLLDDDDTTDGDIPVIPIVGMGGIGKTTLAQLVYHDDEVKKNFDFQAWVCVSEEFDVIKVTKTIIETITSRSCKLTDLNLLQHDLKEKLARRKFFVVLDDAWNEDYDAWNSLLKPFQNGVKGSKILITTRSKKVASVVQTVPPHELNLLSHEDCWLVFSKHARLSTDSMQNTTLGKVGRNIVKKCNGLPLAAQALGSLLRGNSDVKYWNHILKSEIWEFSDDRIKVVPALRISYYYLPSCLKECFVYCSLYPKDHEFDKDELILLWMAENFLQPVGKKSMEEVGDEYFDELVARSFFQPHSTREKIFVMHDLVHDLAMIFGGEFYFRAEEVENTVEVDIKTRHLSHNAKGNYPMSKLLGVCDKVKHTRTFLEVNLDWGIPFNMENAPCILLSQLKYLRVLSFKCFPLDSLPDSVGELIHLRYLDLSKTYIVTLPESLGNLYNLQTLKLNGCANLKTRPAGMQNLVNLRHLDITGTCLHEMPKGMSKLKDWLGHSSYHNITKVLMGGFLSGGFRKCCMLPSLGELPSLKHLKIKGFERLGIVGAEFYQNHDSCVETPFPMLETLWFESMPCWKEWRSLEFNAFPRLRKLTIWGCPMLTGDLPNHLPSLKYLDIRNCEQLSSCLPRAPAITKLCLLHSNNVRILELPPLLHELLIKGKNLVEAVVPAIKNTQLSCLTSLSISDCSSHILFPLSSIPASVKKLRISSCRNLEFQIEGQHDSLHILEITNSCDLVTSFSLLDSFPNLVCADITDCEKMESVVVSCSLSCLRSLYIYNCARLKSISTLWTAAPQLENLTIMGCPEIDLSAIRDPQCSLRSLRIGYFEKLVSSAALMNLQFHGLTHLTIEGECESVSVKSLPKEVVSSSTFAARQKDVLHWVIFYGIFAGIKDGYWHKACYRCQGYRSHWVKRASDPALVGGAVLSSFLNVVFDAEQKQIQIKERAVKDWLDSLKDAMYVADDLLDEVFTKAATQKDPGTFFSRYLSLQDREIANRMEKIIDMVESIVKQKDTLGLREIPKENMSWRITTSLVDRSNVYGREDDKAAIVNILLDDDDTGDDDISVIPIVGMAGIGKTVLAQLVYHDDRVKEDFDFRGWVCVSEGFDVMKVTKNILDAITKSLCNLKILNLLLQGLQEKLSGQRFFIVLDDVWNEDYYDLNMLLKPFQHEVKGSKILITTRSKKVASVVQTVSPHELNLLSDEDCWLVFLKHASLFTDSVQTSTLEKSEIWEFFDDRINIVPALRISYYYLPSCLKECFVYCSFTSLYSKDHEFDKDLFVGKGEENKITELGALANLHKSRMFDKNGINSLKLKWSSDKDENIADSQVKRDILDELQPHSSLKELEIGTRFPDWLGHSSYHNITTLTLQVQCISSP

>arahy.Tifrunner.gnm2.ann1.A2XUUV.1

MAESLLRMVIENLQAFVQDELATLWGVHSQIQELSGNLAAIHAVVQDAEEKQIRERAVKLWLQKLSDAAHVLDDILDECSIESNRLHSEQCLTRLDPVTIMFRRDIGKRMKEMVDRFRQIDEERRRFELRGRVPERQQEDEAWRQTCSGITEHNIYGREQDTENILEFLSRSADSSNDLSVYPIVGMGGLGKTTLVRWVYNDKKVIEHFDLRIWVCVSTEVNTMRILESIVESTRGHNPNLSTLEAMKNKVQEVLLGKRYLLVLDDVWSTDKWEDLKSVLLCGGGTKGAAILVTTRVESVASVMGTCPAHHLSPLSEDDNWLLFKYHAFGSDKVERRELVAIGKEIVKKCGGSPLASKALGSLLRNKKEEIQWVNVLESKFWDILEDDAIIVRALKISYFHLKLSLRQCFAFCAIFPQDFRMEKEQLIHLWMANGLIKSKGKLEIEDVGNEAWEELCQRSFFQEVEIDELGRTTFKMHDLFHELAQFIMGEECRVYDESASLTNLSTRVHHVTCLKPETEVNMDPFKKAESLRSMINLLPLDDHNLCGLPPFNSLRALRTNASQLSELKSLTHLRYLNLRRSGITTLPECVSRLQKLQILKLESCLNLSCLPKHLTQLKDLRHLLIKECHSLVEMPPNIGELKCLRTLNLFIVKKNAGYGLSELRDLQLGGKLRIKGLENVINEGDARDANLSAKKKLEKLYLSWDSSDSRCGANAERILEALEPPSNLKSFGMNGYCGVELPSWMQNTSILSSLVMVILYDCKNCKHLPPLGKLPHLTVLYVSGMKDVKYIDDDSYDGVDEKAFKSLKDLTLSKLPNLEGVLRDERVEMLPLLSKLKVSCVPKIKLPLLPSLEYICIEGTGSESDHGDSDSDGTASIPDSIVLNMRHVKALRITDFPRLKALPHELSSLHSLQKFEIYDCDILESFSENVMQGLCSLRSLKIGSCKKLKSLSEGVGHLTCLESLDIMYCPELVLPSSMNKLVSLQRVYIYSCGTMPEGLQHVPSLQSLNVCGIPSIPEWLGDLTSLQKLRLECEGLRSLPSSFRNLTNLRELSIGGCHKELQKRCTRVTGQDWQNIAHIPQFKLFPIRQETFSDKIRSKWRSWQLRRDRRHHHFAKADTFDYLGFSLWGEVLAQVIAIATCGLIFAECDKFIWFENSKPLIFAKRNMSSAIREKRRNKVKLYENDERQLAMMDYIESGVDIVQIQTIPH

>arahy.Tifrunner.gnm2.ann1.A30FPN.1

MASVVGGAFLSGFISIVINKSLREDAVNQVFGKKLGPGLVERLKISLLAAEAVVDDAEYKQLGDDRVRDWLNSLRDAVYDADDFLDALLTKEATQKEVRSVLPSFFLNRHRKMVDNMERVVSRIEFLASQKDILGLQKSTKDSNLSSSSSSSWRETTCLMEGNIYGREDDQQALIKIINDNSESQLSVIPIVGLGGVGKTTLAKWAFSVVKGFDPKKAWVCISETFDVAEITRKTIEELAKNSCSLRSLNLLQDELQKFLFGKKFFIVLDDVWSDDADKWKQFITPFHCGAKGSTILLTTRNQEVASVVHTCPPYILNELSQDSCWLLFAANACFPESNGNPTLEEIGRKIVKRCKGLPLAVETLGRMLQGKDDAKEWNAVLRSAIWEFSVKNSKIIPALLISYFQLPPYLKRCFVYCSLFPKDHEFEKNELVLLWMAEDLPKRGESLEEVGSQCFEELASRLFFKPDDEEYVMHDLLHDLAIFLAGDFYCRIEKRSEQEKKVLTRHLSHLPYRWFDHTSSKVFKSDMKPESLRTSLYIDDLLSKKSRASKLKYLRVLSFRQLYVLPDSIGKLIHLRYLNLSGCSIGRLPESLCNLYNLQTLILYQCRFLTMLPNGMHKLVNLQHLDLRETSLEEMPRGISKLKHMPILDYFAVGKHEDNGIQEFGGLPNLEGSFEIKKLENVVDVSQARSARMLEKNHIDKLLLEWSSGNKMVSKIETLRDILHNLQPHNGLKELTIKGYKGERFPDWVGHCSYNNMTIVSLEFCKNCSMLPSLGQLPSLKSLSIEGFDQLKRIGDEFYKSDNEHHSSPTAPFPSLETLKFHNMPCWEEWHVPDPEAFPRLRTLEIEDCGMLKGDMLNGILWRRDCCLREDEEGRSDEMVGGGDALSIRPSQSFNARINCLPKSLQILTIWRCPKFEFLEQQQHKYDLLELLIDSCDSLSSLSLDVFPNLKNLVIRECRNLESVSMSEAPHTDLKEVIIDSCGKLVSFGGEGLAAPNLTHLEVKDCEKLEALPRDMKSLLPNLQSLQIYGCPNICRLPEGGLPPNLKKLTAGGCEKQVKDLSWMANLDALTRIRIYGFGCENIKSYPEVGSLPHLPSLTTLCIDRFDNLETLECNELLRLSSLQQLKISFCHKLENMEGEKLPPSLLLLQLYCCDLLGEHCKNKHQLIWPKISHIPTIQVNFEQVF

>arahy.Tifrunner.gnm2.ann1.A58C4F.1

MCGGQGSKLLVTTRSTLVSKAMGIDEPYVLRGLTDEQSWTLLKNLTFGEDSSRMNSELQAIGKEIAKKCKGVPLAIKTIGGFLRIRVEEIDQWSTLLHGDIWRLCEEENSIMPVLNLSYRNLRPELRQCFAYCCLYPKDSVIRKNECIQLWMAQGYLASLTGTQSMEDVGNNYVKILLMRSFFQDASMDENGHIRSFKMHDLMHDLATSVAGNDCYLHTEGKGIVGRPMHVAFETSTICSLDVFDVCKLRTIIHCKIATNLVAKLSFMEKLKCLRALDLSFHSLTQLPMSIDKVKHLRYLDLSYTELRSLPESIGNLICLQTLKLGHCESLVSLPESVGNLICLESLKLNDCKQLVFPTKIIIKLINLKKLDIEGCKAFEDGMPVGLGKLISLQSLSSFVVGNDKKDTSGKLNELKELDLRGRLIISELGLVKDAASESHETNMRSKKHIQDLSLLWGPSSDSSEYEIRKSESLVLLDNLCPHQNLRSLHLEGFPGVRLSDWLISLIHVVRISLHDLPNCKHLPPLERLPCLRELEVADMRSLEWMDYYENIGDVFFSSLEQLDFHYCENLRGWQMLGDETQNHLSLPPFPRLSYLQIVGCPNLTCMPSFPHLVRKLELRWSSSVKPMLETCMVKHESSSISPLSHLKLLLLWGVTGIEAMAEDWMKSLTSLKSLHLRGSSAIQILSRHLQYLPSQLKNLAISFDDDKLDLWKDTQGRGPPHALSSLQTIFFFDCKNMKALPEQIGNLQSLTLLEIMNCPKLESLDEADRRLTNIHTVGISGCPFLEHKYRAESEDRAKIAHIPHISIY

>arahy.Tifrunner.gnm2.ann1.A71BF1.1

MAAKLEGRAFLSSFVDAVLNKLSSIKSTPAEQKLLRRLRASLRAARPVLEDAELKQIKDQKVKKWLVDLQDALYMADDLLDELSTKASTQSNPSNSSSWSRYVNSILEESADDIRVSNSMQDIVDTLESVVEEKDDIGLKEEVTKELEDMSWRIQSSLIESSDIYGRDKDKEDIIDLLLDSACDSKLSMISVEGIGGIGKTTLAQLAYNDAKLEGNLILEYGCVLLPNLILLKPQRLQ

>arahy.Tifrunner.gnm2.ann1.A9ERQR.1

MEIVTAVAGKVVDLTVVPIGRQLGYLIFYRTDVNKLKTSVEKLKAKIDDTNISVEAAKQNGETTPFPSVQKWIEDAEETVADATALIQKEAQGEGSCLKWPFPNLCKRYSLSREAKKLERDVSNAMLEGKFEKLCYRSRPEMSLPPSSRDYEAMWSRTSTLDEVKQALKNPGMCMVGVYGMGGVGKTTLAKELAWQTEKECLFGVVVMAVITSTPNLRGIQGQLADGLGLKFDSETEEGRAVELRRRIHGEKSILVILDDIWGELDLTKVGVPYGDQHKGCKLVLTGRDLNVLHRMGTQTDFPLGVLSEEESWSLFETMVGDVVRHDSIKPIAVEVAKCCFGLPLLIVTVAKALRKKEDEKYWKDSLKKLRKFDPQRFHKSVYDSLEFSYDCLENEELKSLFMLNGSLVDCFSKSDGSFNTQKLLPFCWGLGLYKDVHTLLEGRNRHHALVNDLIASSLLLDSGTEGVRMHDLVRDVAKLIASRTFPTFDEQEFRNINKWPNKDQLQKYHHIILPHCRISKFPDEKLEYPNLKLFFLQSVVGKLNVPDNFFSGMRGVKVLHFDIGYYAFHPLPSSLRLLANLRSLKLSGHFEGLAMIGELTSLEILELKDKAIKELPKEIGHLTQLRLLNVRRCERLRLIPKNLLSNLKSLEELYMWDCNIKWEAEGSNKSSNNASLYELKNLHKLATLELKVEHGSLLPNDLHNLANLEGFKIIIGSSRNNYMNSYPVWPRSLILKGASTAYILQNDVVKVLLSSAEYLDIYDLECLEDIFPGLHGDGFAELRSMSIRCTGLRCIINSNFNHFQLTFPKLEKLNLDTLNNMKEISHGPFPSHSFHALKSIVVSRCDVLNHIFLWSEIGHLSQLQNMEISECQSMQEIVAGGMLEMKNIVLPQLRSVSLKSLPRLVSFCSVPMTSDDMGFVPVALFDNKVAMPNIETMVLFEINIYKVWDDGLPMHHSFMKNLTSLTVEGCENLITLFPSSVATALQKLHHLKIMSCPSLEQIFDQEEDLENHRSSLEQVMLPNLKIIEVQDLLNLKSLWANHMSPNSLHKLCKMRIMNCPKLINVFPSSVIKRLINLETLLVTKCGELQVIFEIQEPSTVTTSQQQGLPTRLRTLDLSFLPKLKYIWSKDPHGILSFQNLCEVRVFDSQSLEHVFPVSMANETHQFKVLEIYCCGVENIFDKSEFGRLKDAHIEVLTLNSCDHLKNIFPSSVNFQNLDGLYVEWCKELVNIMTPSMAASLTSLRVLNISECDMIEEIIASDHNNNADVVDDGDALSEIAFMKLEKLELSNLRSLKCFCKGTYSFKFPSLHSVTVRYCPMMETFCDGNSKLCAPRLTEVISSRGIYEYDTPQRRWDGDLNTTIRNIFIHKAQARLVA

>arahy.Tifrunner.gnm2.ann1.AJ79N4.1

MAAKLEGGAYLSSFVDAVLKKLSSLDVNSTPMARKLADQDLLQRLKLSLRSVRPVLDDAEQKLIRNDQEVKKWLLDLQDALYMADDLLDELSTKAATLTPTQRDPGNYSSMCHSIVDSILEDSDDDDYEMGVADLVDKLESIVEEKNGLGLKDEPVRDPEDMSWRIESSLVESSEIFGRDNDKEAIIKLLLDDTCYAKISVIPIVGMGGVGKTTLAQLVYNDERVDEKFDIKAWVCVSDQFDIVKVTKTLIEAAGGSSYNGNSLDLLQTELKDKLMGKKFLIVLDDVWIDNSYSRQWKTLQKPFQFGKKGSKVLVTTRNDTTADVVKTISAYKLSLLSEKDCWSLFLKCVFLSSESKEYSTLEPVGRELVKKCKGLPLAVESLGALLRTNYDEREWDSVLKSELWEVFEDQNDEIIPALSMSYYYLPSTLKRCFVHCSLFPKNYLFVKHELILLWIAEELLQPKGKKDLEEIGSEYFDQLVARSFLQSSSTYNSFYVIHDLMHDLATTHAGEFYFRAEKLDEHIRINNKTRHLSRNARGNYPFAQIVAACGRVKDARTLLEISFCQEYPFVAPSTKGKNFLHDFLSKLKCLRVLSFNAFPLYSLPDSIGNLIHLRYLDLSNTTTEILPEELCCLYNLQTLKLKNCRLLKKLPCNMQDLVNLRHLDIDGAGLEEMPKGMSKLKDLQFLSDYIVGKHADNNQIKELGALANIQESLHVCQLENIEDGAQALEARIADKKHIRVLYLTWSSNSHSHIDDYQDILNNLRPHTNLRELSIKEYKGLTFPNWLGHSSYKSMTKLYLYGCSNCLELPSLGHLPSLKCLRLSNFYKLQRIGAEFYKEDGSSSRKAAFPVLETLSFEGMDCWEEWLSVSSELDAFPQLRELTVRRCPALRGDLPIQLLALQSLCIDQCGQLDFSLPRADALLELSVEGPQKVEAVLKAIAGNQLNCLQSLSITNCCSATSFLVGSMPSSLQHLVINGCPYIELPVLQQQHESLQSISISNSCHSLTSFPLASFPNLTTLRISSCINLRSLLASDPTSASTSASSLHSLTIESCPSLGSFPTLEMVAPHLEYLLLRECPEIESFFEGGLPPNLRELKIQHCEKLVKYLASMDLCDHCLTCLDIRHPYDNIKSFPMGGSLPASLETLNLRSFPCLEILDCKGLDHLQRLLIDYCPRLDVAGESFPASLSMLHLNGPSLLRKRTLGFDGTILKLKPRMEQQSELL

>arahy.Tifrunner.gnm2.ann1.AQDT95.1

MAFSDVYSAPTPPPPPLTKYDVFISFNGKDTRNGFTSHLHYALCRNQIETFIDYRIEKGGEIWEELVQAIRESSVYLIIFSEHYASSKWCLRELAEIIELTNMVEKGHHHIIPVFYRIEPTHVRKQTGSYYSVFAEYDRNLDNRLVRQWRKALFQAANISGFEYNHHHSRTESDLIEGIVQMIIRKLDQKYTSELRSPFIHDQNYASIESVLINSQDVRTIGIWGMGGIGKTTIAAAIFQEFSPKYEGSCFLANVREESSRHGLNYIFNRLLSELLQEHVHITTPKIVSSAIIRRLRRKKVFIVLDDVNTSELLENLLGVGQDYLGLGSKVIVTTRDKHVLLSRSVDHIHEVMEMNEENSLKLFSLNAFNRNHPPENGYWDLSKRALAYARGNPLALKVLGSFLHSKTEKEWDNALTKLKRIPNADIQKVLRLSFNELDDTEKDIFLDIACFFKGEEKEKVIRILNECGFFADIGIRNLLDKALISIATNKSIQMHDLIQEMGHKIVCEESFKNPGKRSRVWHPDEVCDILENDKGSATIETIYLDMTQRTEICISSGAFRKMPKLRLLAFASSSIDYGRKRVDYTLSLPTNLELPNNLRYIQWDRCPLKSLSTTSWPNKLVELSMPYSNVEKLWDGPQSLPSLEEVFLSGSKRMIECPDFSGAPNLKTIWLNQCESLPHVHPSIFSLPKLDYLAVYGCKKLKSLSSNNCPLSLRSVVAYNCPNLEEFSIPISKNQSNIHVHLRSTALKQLPSSIVHLQDLTNFSFPISDLLMALPEKFTNQIMLSDPNNKEYDSVATLRRILPSPMFHYLKELKFDGCQSLIELPDNISLLSSLLVIRLHNTNIMTFPENIKTLPRLKIVLLCHCERLQYVPALPPSVHHFKAWDCKSLRTVSSSTSELKRQHGTTFIFVNCLKLDEESCNTILEDAIVRMDTRAKTQQLSPRLEENRNEECTVVDDDDGFLSEDNANVGKVCYFLPSRGSKLGGLFHRGSSQNSISIQLPQGSNFFGFIFYLVVPPIQPCNTGGDLDIRFGFECYLETSWGQRTHIASSSLIEWSCEFYYGYQMNLLSDHVLLWYDSQCCKQIMEIIRGRKAIDDDDKNANLEVKFFARLPNKEEVVIKECGIRWIYTNMEKESRVCRFKRSRQVFELEEKDLESDDEGEELVPPAKKFKNSLMEVESVENLR

>arahy.Tifrunner.gnm2.ann1.AR99YL.1

MHQAINILFKDISIPTTTKKEKERLDDSELIRHIVKDVLQKLKQRRPNTTKYLVGIEKTQKDIEMLLQNAKIIGIWGPGGIGKTTIGKVVYNKLSSQYDSVCLMENISEETRRYGLPRLCRNLFSDLLKEDNPTHNVRRLQTKKALIVFDDVDSFKQLEYLCEDSQVLSYLGEDSRVIITSRNRHLLANKADEIYEVKNLNLTESLKLFSLKAFSKSYPEEGYEKLSKQVVEYTGGIPLALNVLGSHLSFRLDKDFWESTMRKLENSPNEDIQKVLRVSYDGLDSLQQKIFLDIAFFFIEEEEERVKKILHASGFEPNNGLLELKDKALISTTSYDSRIQMHGLLQEMALDIVRRESELNPGGRSRLRDVKDIRDVLENNTGTNAVEGIVLDLSQIEDLQLSADVFEKMNRIRFLKFYIPRSKSSSEKYLPEGLTQFPKELRYLQWDAYPLKSLPLRLCFKFLVEIHMRHSNVEELWQGKKDLHNLEVIDLCECRKLMNLPDLSNASRLRTVNLSGCEMLHYLHPTVLSAGKLATLILDRCKNLMEVKSEKRLESLEKISVNGCLSLKEFEVWSSLIEKLDLSKTEIETLHTSIGHMENLRSLNLEGLKLKHLPNALSGLKSLKYLNLSRSGVEFDKHQLHVLFNGLQSLEKLHVTDCSNLFELPDNINVLQKLQELRLDGSSIVTLPESIKHLRVLKVLSLENCNKLVSVPELPPSITVFNASNCTSLVSVSVSTLRMMGKTKCILFKDSLKLDKYSLPYSITESLHLTMMSAAFHNVLVRVFDNKLHNYNYVKVRGCLPGSRVSRHFKNRTRGSTITIRLPSCTSVLGFLLSVVISPGVANANIYCRCYSADGTPIGEKTRWYSVIESLNSDNVFMWYDPYFSDSILKLHETHVSFKFYSEEGLELDIIKECGVHVIGPSEFQSVLGEMDLEYEQKVELGVKLGLALDIQRQVDSNVDLPNLLFAFEYGWKLQPPMQSELESRRRAMADVIRKQMWEFIFPPCPAAGMWGNITQDFEDRGLGSGHGGEKVRSKDKGIARVSLLPSEFEPFGR

>arahy.Tifrunner.gnm2.ann1.ARHM7G.1

MVAKFEGRAYLASFVDAVLNKLSSVNSTPIARKLADQKLLQKLKACLRAVRPVLDDAEQKQIKDQEVKKWLVDLQDSLYMADDLLDELCTKAATATPIQRDPGNSSSWSHYVDSIVEYCDVDEMGVVNSTQDIVDKLESIVEEKDDLGLKEEIAKDLGDMSWRIQSSLVESSEIFGRDNDKEAIIELLLDDTCDANISVIPIVGMGGVGKTTLAQLVYNDVRVERKFDTRAWGCVSDQFDIAKVTKTLIKAAGSSSCNEDDLSLLQTGLKDKLMGKKFLIVLDDVWIDNSRNWKSLQKPFQYGKKGSKVIVTTRNDNVADVVKTISAYKLGLLSEKDCWSLFVKRVFLSTASKEYSTLESVGRELVKKCKGLPLAVEALGSLLRTINYDERDWDSVLKSELWEVFEDQNDEIIPALKISYHYLPSNLKRCFVYCSLFPKDFEFDKDELVLLWMAEELLHPKGKKTLEETGCEYFDQLVARSFFQSSSTDDSFYVIHDIMHDLATIYAGEFYFRAEKREENIGISNKTRHLLHNSRGNYPFSQLVEACGKVKDVRTFLEINLGRMDPFSMENAPHVFVSELKCLRVLSFNTIPLHSLPDSVGELIHLRYLDLSCTRIKILPEELCNLYNLQTLKLKDCYLLKKLPNNMQDLVNLRHLDIQGADLEEMPKGISKIKDLQFLSDYVVGKHAEDNGIKELGALANIHGTLRISQLDNVIDGDQASGARIAGKKNIRGLRLSWSSNNHIVDSHDILNNLRPHTNLRELSINDYKGQTFPNWLGHSSYRNMTKIYLNGCSNCLELPSLGQLPSLKHLFLSEFFKLTRIGAEFYNSSSCGAPFPMLETLSFYHMYNWEEWLSVSSEFELDAFPRLRVLTMRYCPKLRGDLPIQLPALESLCIRHCDQLAFTLPKADAMLELSVEGTQRVEAVFEAIGRNQLNCLQSLSISICYSSISFPGACLPSALQNLDIHRCPYLEFPMPAQEQQHESLQSLSISSSCCSLMSFPLASFPNLTKITISSLKNMRSLEESDPTAASTSNLRSLHIDNCPSFESFQMVAPHLEYLILRNCPEIESFFDGGLPPNLRELEIQSCKKLVMYLASMDLCDHCLTHLDIEHPYDNINSFPMGGCLPPSLERLCLRSFPTLEILDCKGLDNLQKLLIDHCPNLQDVAGKSFPSSLSMLGFSGGSLLRRRWQKKDPLILSKMSHIRNINVDGYWIS

>arahy.Tifrunner.gnm2.ann1.ASR4JM.1

MDSTDRVHMVGIYGIDGIGKTTLVVAFYNLITDNFKEMPGKRSRLWFYKDIGSEDPSEKEKNDDVEVKWDGTAFKQMKNLKILIIKNEFWNGGGILQSVFPLILIQNSFPYLSCLIIPTCYPSWIAYPSDFLKEILDVSNLQTLEEFSFKRYCNLVLVHSSVEYRKLRNFSPVINLASLETLGLFGCSSLEKFLKIPKKMKNLEELSLIGTDIKDLPCSFCNLYRLHRSDLEKNEMYGIPSVIGMIPRLSWCNITLGGNKGRVSREKEKEEN

>arahy.Tifrunner.gnm2.ann1.AUU7HE.1

MLNHSKTSDCIQPEEDNTSFYVKWILWSLIIGWFGISLWYDCWLKYYKGKVKWIENKLSFLEALKDDHYQLTEEADNLRSRGREFWGSEDLSEINLWLSKEESLWVNSTKTLVSDADACIKNYKKQSQTRIPGCFTAILRLISIRDVFHQIDRVKTSVEGHLENKDKDAKDIYDSMQKSRLRNRSLLDQYAAEESQKTVKTEDDKEDQKQRKPSTSINELMRKSNDLFSGMEKRRSVHLLLPLHAFTNELSQLQLETNTENLWKGQAKMLIAEAEDLISAYSWWVSYFGSIRGIPIVGTMFKKYFNGIPIKTTQFEEDMERIVDEFHNLLDTKSKWKFDFIDSDAMESELPFQNADDDEITSTVNSIRIKLDQSGSKQEPMNSLYHQLEEMNKELRGRREETAVRKACLEHLKSISQEVDQSVSNFLKEKMNNPELQQILKATDLLQKIVKNCYTKRQNSPSIPATKNVAKELVSKLTTSSGNLLTLSIVGMKGVGKTTLAKAIFYNKDVINYFPVRVWVADGTTSKVKVLLMKQDGTIDHQALSITEVRDHLKGKRGLIVLDNISKKDDFDKYKKELISETGMTNRSRILLTTPLNNIASYADTCGGPHQIRLMTKDESWALFQKVTMTVKPKENSQEEKLAKKVVGRCGGLPLLIVSIGFLLSVKGSITEDNLRPLLRQINHGHQNIRWLQAWVNTNQELNETLSDCLYYMTLFPADFEIPVRRLVNLWIEEGLVKQRDDNQETLEATAETYLENLNKCNMIQAVALKSNGKIKTYRLPSMLREIILADKTSHSQYSGTHVERRFAYRFDDRGLDANAANVFSKKKIPLSVFFFDKREGCKPGEHIGRILSTGIANEQFMEISVLDLERVFRPQLPDTLGMLIHLKYLGLRWTYLEEFPTFICKLENLETLDVKHTCIRVFPSFIWKLKKLKNLYLNQDYRSRLEGRPSGNFQHNLYRLWGVFIYGRCPLLFDFQRLKNLGKLKLAFQLKAEEQDTLAKKIVQLNQLESLRLRSVNEMGEPEKLILRDISKLEKLASLQLYGKLENGLRMNHLPQNLINLTLSASKLQDSQDPMQLLQSLEKLESLCFYADSYHGKSMLCNPGSFPKLLVLRFWNLRNLEEWNVKEGAMPSLREFEARSCEKLAVPTGLKYLGNIQLIKLHKMSNTFMREILSSYKKKILSPDVRISPFKLPLALRYSDLYLSKKQARRDYCFDESVFSVLHVSPPSQALNNLHLLASLSSNNPKPRDGGEMTNYTLLGTFSLSSSFTLRQFKDDKDCYNKDGGVVEVGDTVNKENGYNKDGMTTKKMMWQVATL

>arahy.Tifrunner.gnm2.ann1.B2L4SY.1

MVISSSSYSQTSVTHQYDIFISHLHSALCRNRIETFIDYRIKKGGAIWNELVEAIRDSKLFLVIFSENYASSKWCLRELVQIMECKKKNENGTLFFPLENSGFMKSLTKSFYPKKKKSEPPFWDEFISYSFETSSYFWIGGSGLDIRSKARISLRRADASMWLMLINKLSKKSLLPVLFSTETFAMGVNAPARTSWKEDV

>arahy.Tifrunner.gnm2.ann1.B2M8AQ.1

MAAAIVGGALLSASVQVVLDKIISNEFLDFFRRRKLNVSLLGKMKMTLLSVQAVLNDAEEKQITNPAVKEWLNELTQAVFDAEDLLDEINTEALGCKVKARYQSPSCSAKVRNVFSSRFGRSYGMINSKMQTLFERLEHFAQQIHILQLKEGVSNSVWQGTPTSSVVDESVIYGRDDERKKLREYFLSEDVGASGNKIGVISIVGMGGIGKTTLAKLLYNDNEVKDKFDLKAWACVSKDFDLFRVTKTVLESVTSKTTNTDNLNTLQVELQQRLWRKRFLLVLDDIRGTSYVDWANLKDIFNAGEMGSTIIVTTRDENVAKAMQTFPIFHLTSLDSEDCWSLLAEHAFGANNSSERSNLEGIGREIVKKCDGLPLAAVALGVFFAPNCRKMIGISNIWDLPNLNVQPALLLSYHFLPAPLKRCFAYCSIFPKNSALEKEMVVRLWIAEGLVHVSKSKKILEEVGDEYFDELVSRSLIRRRYEDQKALENPKKVRHLSYNRSWYDYFNKFNSFYGLECLRTFLPLPLNGWSFDYYLSNKVIYDLLPKLKQLRVLSLSHYKNISELPDSIGGLKHLRHLDLSGTNIKRLPSVVCKLYNLQTLLLSDCKFLTELPEEIGMLVNLRHLDISGTQLQEIPAQIARLENLQTLSGFVVSRQQNGLKVGELQKFPNLQGKLCISKLQNIVDPCDASQANMKKKEQIEDLSLEWDSCTAQESQHLVLEHLQPSTNLKKLTIKFYGGTWFPNWLGNSSFANMVYLCIRSCHYCSLLPPVGQLQNLKELIIFDIISVKTIGPEFYGCCSPSFQPFPSLETLSFGEMPAWEEWNFMGGIATHFPRLSHLSLSDCPKLKGNLPSNLPSLTKLHLSSCSLLEVDNRNIINASNLFSELMLGLNSLEQLHIEGIPSRTAFPRDGLPETLRVLFLKNCENFEFPNHESLHSYTALESLTIWNSCCSLTSFPLGSLPVLKWLYFIECKNLKSFSISEEEDAPQCLTFLQGFYILECPELESFPHLGLPTPKLRRFWVSYCNKLNSLPEPINALVGLQELTVLNLPNMQFFANEGLPISLRTLRIGNLGGNFSNADIIKWGLDHLTCLSELEIEGGYLVNMLMKMEVPLLPSTLISLHIYHLDGIRHLDGKWLQNLTSLECLKISSCDSLESLPQEGLPSSLSVLTIGSCPLLEASCRSNGGKEWPKVAHIPCIIMDKKVII

>arahy.Tifrunner.gnm2.ann1.B32ZNM.2

LKEWEKAKPVRKRGVQKSSSNVRLSVGGRGASANQRRILITLLPTRNKLLSLSMALNGGSSGRSKWKYHIFLSFRGEDTRLGFTDHLYTALQKRSIDTFRDNEELRTGEFISQQLLHAIEDSLCAVVVLSPNYANSGWCLDELKKIVETKGRFGMIVPVFYDVDPSDVRYQKGKFAEAFEKHEERYRAQKDKVQQWRDALTFVANLSGCTSQDRYESQLIDEIAEEVWTKVEPQLPTKDDDDLVAIESKVNDVRSCLSLEPKGDVLFLGIWGMGGLGKTTLASVVCKRIRREFEDYCILRVGDVSKEGDLVNLQNQLLSHLKLRSRVIETLVQGRDNIRNLLYKKRILIVLDDVRTIEQLENLVGNKEWFGPGSRIVVTTRDKNLLSSHGVFKIYEMEVLNTDESLQLFHQEAFKGELPKEEYLELSKRFVSYTGGLPLALKVLGSNLRRRSIDEWEDALDEIRKDPDGGIMNLLRISYDMLKEGYKTIFLDIACFFRGWYRDKVTKILTNCGLNPTKGISELIGKSLITCNKGVLGMHDLLEDMGKQIVFQESEKDPGKRSRLSSLDDIDQVLEENTGTENIEGMVLKQQFESYSKAANWHPEAFSKMRNLRLLIVLCDLHLSHGFKCLPRSLKVLIWTECPLKALPLDIELRRLVHLQMNNSKLEKPWNGSQVFEGLKVIDLSYSKDLIQTPDISEVPLLEELFLDGCVSLVELHLRILQC

>arahy.Tifrunner.gnm2.ann1.B5ASP3.1

MSSLSNKEVLIILDNVKDSDQPLLEAVCQGYKSHSRESKLIITTTHKHLLENRVDWIFEVKQWDDSKSIELPSLKAFEESIPAKPYEILVNKVVKYAGGIPLALNQVFMLSYLLRFACRTAMSRNSGRKSRFSILMTQYFVQLEQELNNLEGIDLRECKELEELPDLSEAKRLRWVSLSGCETLPILHSFVFSSNTLVSLILDRCTNLQCVKAERHLNSLQHVSVKGCSNLRICVSSDLIENLDLSNTKVEKLDKSIGKLLKVECLILEGSRVKHIPKELSALKLLKKLKHLYSGLEIDKHQLQGLFNGLSSLQILHLKDCSHLFEFPDNIDSLSKLRKLRLDGSNVTWLPATIKCLQELEILSLNNCKFLETLPELPSSIKEFSADNCISLESVSSLNTLATKMVGKTKCISFNNSLKLLAGHTLHSIMESIHSTMVSAVSSNVTVRSYTIDVHSYNYNSVEVCLPGDTIPKQFAYKTEKSSSITIELPESPSNFLGFIYSVVLSPRHGREKHGAKIQCEYNFAGGKNSSWEDITISELNSYHVYIWYDPFLTDKILGQYGPSFHLKFSVATDTGN

>arahy.Tifrunner.gnm2.ann1.B5WJWN.1

MAEALVVGALLSGFANVVLDRLISPEFVNLVVGRKLDRKLVERLKAALLAAEALVADAEQKQFGNDRVSKWLDSLRDALYTADDLLDRVFIKAEIRNKVRTHLPSFLNLSDRKMVTKIEEVVKTIEDLEKLNDTLGLEKIPTSSSSWRPPSTSLVKGNVYGRDGDQQALIKMLNDNNDHTLSVSSIVGMGGVGKTTLAQWLYNNKDLMDGVDLKVWICVSENFDVVETTKNVIKGISSGACSLDSFDLLQQDLKEKLSEKKFFIVLDDVWSEDVDKWNSFVTPFQHGRKGSTILLTTRKENVGRVVQHYNSYTLKELSEDYCWSIFADNASFPESNGSLELEGIGRKIVKRCDGLPLAAETLGRLLRSERRVEEWNKILSNDIWEFPMSNSKIVPALLISYYHLPEHLKRCFVYCSLYPKDYQFDKDELILLWMAENLLRPPKRGETLEEVGRECFDDLVSRLFFKQGEDYFRKYFVMHDLMHDLATSLAGNLYCKFSEELGEKEEMSILTRHLSYGDSIPEKICSSNKIESLRTLLYLEHGASASKGPATLPGDILSKNKYLRVLSFGRLNIFPDSIAKKLIQLRYLDLSWSDIVVLPESLCKLCNLQTLKLEYCFKLTMLPNGMYKLVNLRHLDIRGTPLKEMPKGMGKLEQLHILSNFVVGKQEDNRIEELGGLLNIHGSLEIENLENVIDANQARSARLIDKKHIEYLSLKWSVSPGAHTDEEDILGGLQPHTGLKKLSVEGYKGKIFPDWIGHSLYQNMTSVSLDYCWNCCVLPSLGQLPSLKSLSIRRIEEVQSIGKEFYKNEGHQHSSPIAPFPSLERLEFYNMPCWEEWHLPDSEAFPQLKSLQITYCGKLKGDMVNQVLMRIVSSSSDVSKVRQLEIQEQRESWGNKEMRLDGDRLSISGFECVVECAFKARIIHHLTSLQEIQISWCSSVVSLGGNCLPKSLQKLKIFNCRQIELLQQQHKYDLVHLQIEFSCDSLTSLSLDAFPNLKNLEIRWCSNLESVSMSESPHASLQRLSIKWCDKFVSFPEEGLATPNLTHLDVSRCSKLDALPRGMNTLLTNLHTLKIQGCRNICRWPEGGLPPNLKELRVGKCEEQVKGLSWLGNLDNLTHLFIDGSNFESIIKSYPEVGWLPRLPSLTTLKISCFDNLETLECSELLRLTSLQQLHISNCYMLENMEGEKLPPSLLLLGIYDCGSMCTYRDVILYIGYTSFNSKFI

>arahy.Tifrunner.gnm2.ann1.B6JPF5.1

MAVALVSGAFLSGFINVVFDRLLTKDTVNLVLGKKLGSRLVERLKISLYAAEAVLDDAEYKQLGDKHVREWLNCLRDAVYDADDLLDAVHTKAATQKEVRSSKLSFFLNRHRKMVDYMEGVVSRIEFLVKQKDDLGLLKSTKDNNLSSSSSPTPSSWRETTCLIQGNIYGREDDQQALIKTINDKSESQLSVIPIVGMGGVGKTTLAKWAYSVVKGFDPKKAWVCISETFHVAEITRKTIEEITKTTCSLASLNLLQNELQKILSGKKFFIVLDDVWSDDADNWKQFITPFHCGAKGSTILLTTRNQEVASVVQTCPSHFLNELSEEYCWLLFAANACFPESNGNQTLEEIGRKIVKKCKGLPLAVETLGRLLRGKGDAKEWKAVLRSDIWEFSTKNSKIIPALLISYFQLPPYLKRCFVYCSLFPKDYNFEKTELVLLWMAEDLLRLPKGGESLEEVGSECFEELASRLFFKKLQDNDKYFKDLASWLFFERSRFFSERFVMHDLLHDLAIFLAGDFYCRIQELREQEEKKVLTRHFSYFPLGRLDHPISKVFKSIVKPEESLRTSLYIDDLLSMESRASKLKYLRVLSFRELDVLPDSIGKLIHLRYLNLSRTDVKTLPESLCNLFNLQTLILYACYKLSMLPNDMHKLVKLRHLDLRGTSLEEMPRGISKLKMPILEYFAVGKHEDNGIQELGGLPNLEGSFEIKKLENVVDARQARSARMLEKNHIDNLSLEWSSGNEMVSNIETERDILDDLQPHNGLKELRMKGYKGERFPDWVGDCSYINMTSVTLESCKNCCMLPSLGQLPCLEYLSIKGLDQLRIIGEEFYKNDSGHHSSPIAPFPSLEELVFANMPCWEEWHVPDPEAFPRLRKLRIKECPTLKGDMLNGISLRMVSSLSDASEVRKLEIRGDHEERYDEMLYRDTLSIGGCESAVKSALNLLACFPEICIVGCSSAVSFPGNCLPKSLQKLIINDCRNLEFPEQQQQKYDLVELQIHNSCDSLTSLSLDVFPNLKNLEIVRCRNLESVSMSEAPHAALQRLSITFCEKLVSLAGEGLAAPNLTFLNVSKCYRLEALPQDMNNLLPSLRSLDIYSCSKICSRLPEGGLPPNLKSLTVGGCEEQVRSLSWMGNLDALTHLTIHGSDCDSVTSFLGSLPHLPSLTTLSIEYFQNLETLECNELLRLTSLQQLRISICHKLGNMEGEKLPPSLLLLKIQYCGLLGEHCKNKHQLIWPKISHIPTIKVDSKQIS

>arahy.Tifrunner.gnm2.ann1.B7APXK.1

MENYDVKFLCSYGGEIHHRPNDKKISYVDGHNKLHYVNRGIDFTAMLAELSALFDAAGDIHFKYQLPSDDFDALISVTSDSGLNSLMLEYDNLYRDSPKTARMRLFIFPGNGPDSASTQPFPAKLKSKVNNGVVVPPQIMPVKFQDLPPVNNFNTLDRVLDSDPKVNRPIEDSDSSCLNSLLPDLQYDVFISFRGEDTHASFTSHLFKALSRKLIVTYTDDLLHEGDSITSLLLRAIEESCLLLVVFSENYASSKWCLQELAKIMECKKEFGRLVIPVFYNVDPSHVRYQLGSYSEAFKKHLQNNKKADVQKWREALTAAANLEGLDSRSYRNPFLFNFVDDRKLIDHYPPNDSKSLVGISENLEKVESLLSEYVEVRMIGICGIGGIEKITLAILIFEKYSYTFEGSCFLKNVRERSGNYGLTELCHRLYLELLQGKFWQNNTGKSTFVEDRVSKQRNFIVLDDVISLEQLDYLVQKLQWCGADITARDKNVLVPTVETIYEMKILDSHESFKLFSMNAFNEDYPQIGYEELSWKAVGCCKGIPLALIALGSFLHSKSKTEWHSALKKLEKTPAPEIQNILRLSYDGLNDEAKQIFLDIAFF

>arahy.Tifrunner.gnm2.ann1.B9AAYW.1

MVASAIGNTITEITRLSTLRVFGKKTTIIIDNIKRHLLSIKEMVELEHGDAAAPTPAWLMDIPLADVATDLWDLLENQKQSNKSSSFLSIEKRLQRIVDDLTKQPLLKIDRNMKDWEMEIPIGFKDNKEAIIERMLALTKAKGAVAVVAISGIRGTGKTTLAKFVCDDDEKVKNHFDMVFWIDNIHGESYADYVVQQMVHQLATKKKKVENNGSIENVNLQESIDGNKFLLVLDGLHNENRDEWFKLKEKLKEAASSSSAAVLVTTQNYLLRNAIDGDYYTTPKLSEEDSWTLFKQVVREDPSESNIKNAQKKMLSKCGGVPLGILTMARLWKSRGDITEDSETGDLEELFMQEMQLIYYNELPSWHLKQCFAYLSFIFPCEYPSVEENELTRLWMAEGFLGAVHVDSNSSSSSCSPQPQPEDLAHNCIQELGRRSILSVYTDKRDNMTECYLDNELISGLSRFVAGKDRFCMDDGNADVKETIPRVALTSKNFYVSNGTLSSLIENNKSLRTLLWDGQLSKFPNGVNLRFSACDAIFCAFKSLRVLKLRDLGIKILPVSIGELKSIRYLDLSRNNMKTLPSSIGKLKLLQTLKLSHCLQLRELPNEVKYLVSLRHLEIDECLHLSHLPATLKKLTKLETLSNFVVSTLNNHKRILGFKELVNLNNLSGQLEISHLERLKFVDGHHDAAAYLKEKQQLKRLILNWNHDNDHEDNKRDDERSLEQLEPHPNLQQLHIVGYSGVKYSSWLSSLNNLVDLSLNNCSKCESLPLEQFPKLKYLHLRRLDSLKYVIDQDDRCSWLEPEHLQSLSITDCPNLMSWWKGNTHNKALLFGTIKEMEIKYCPKLNSLPLFPNLEESLELEGSSVKPLLDTINYSGMASNSSSDWPLSRVKYLRIKNVEQHSLLPEDWLRNFVSLKYIRISERMSLIKGFKHLHSLSIMTIENCTLVDLSRDEWEGLQSLELLLLYQLAKLESLPKGIKHLTSLTYLDIETCPELKTLTEEIGHLKSLKTLCIIDCHKLASLPKGMINLKSLNWLLIRSCPLLLPRCQQGTGDDWPKIKHIKHIQLFFNYINFRPSFEEACFMELVMVHS

>arahy.Tifrunner.gnm2.ann1.BB34IZ.2

MSLSVAAAASSSQAPATYRYDVFISFRGEDTRKGFLSHLQVALRQNQIQTFRDDDGMDKGGLIWDELLHAIRNSNLFLVIFSENYASSRWCLKELVEIMERKNNKNVVIPVFYGIEPTHVRKQSGSYRRAFDKHERSSEDRCHVQQWRTALTHAANLSGITFDDHRDDEAKLISEIVKAILAHCLKNKCFIDGLNRPFICNTNYTRVESLMRLKMEKVLVIGIWGMGGIGKSTIAEALFNNYSFQYEGSCFLSNSRELGKPCLSDICSRLLSQLLNQDLHNTNIGVLDSRTQSKLKKKRVLIVLDDVVDSPIATDLVPGLRTCLCSGSIVILTTRDRSVLTSGGVEQIHEIKQMSNGDSYKLFSQYAFSDPHPKAGYGELTARVIDYANGIPLALKILGSFLRGKSVAEWDSALKKLRKYPNADIQKVLRLSYDALDDVERNILLDVACFFHGHEMEETTRILNSCDFFGDIGIKSLLDKSLISKYKYIKMHSLIQKMCWKIIHEESSENRDQQTRLWNAEEVCNIFQHKRDIHGIESMIVDMNQITIDPCLILIALRKMPKLRLLALRGDINIDLERNRVHLEDFQLPNDLRYIEWDKCPLNFVPSICWPQKLVQLSMRHSNVQKLWDTVQNLPSLEFIDLFGCKRLIECPNLAGATNLKIISLSRCESLQDVHPSIFSLRKIEQLKVSYCTSLKRLCSDYCSPSLRALWATGCSNLEEFSIPIITEHSKIQLYLESPTLSEVPSTIVHLKDVEWFGFNISYSLQKLPQNFTSRISLMDPIKHEDDTCIMLSRIFSTPAFLSLKVLVFYKCKRLSKLPDNIHVLQSLQVLQLQHCYVITSLPKSIKNLQQLTHIYIGNCEMLQYVPPLPPSIIYFYALNCKSLKEVSSRLTSEPPKKHYSWFGFHNCTKLDDDAYEAVLKDLKFRIELVANNDGYPHNEANNNGNRFFYYYLPSKESIINKWFPDYYSREASIIVKVSPDHKISSCFVGCILISQYQSFNLAKKKVIFGWKCYLEKGCNEWEWIATSANRAPLTGSNVPHEIAKEMVSDHKIVWYDGESSNKITEAIEERKKGTTCNPILKFEFYADTEDNEEIVIKGCGIRWMHVHVNHDGEISPDATDDEGYESHDVESRRKENQKKSEFTSWFQRLLLLVSCPIMLAKLLIILDEITIS

>arahy.Tifrunner.gnm2.ann1.BBI6IF.1

MIMTEGGQEEERHRRGGGELTSQRRGGKGNGPWLYDAVLSFTEDIPTTFISDLYTFLTSAGICVFMDCNHETRRVDRISLSLLQAIGRSRISIVVLSTQYANSRWCLQELEKIIECHRKIGQVVVPVFYGIDPSEVLNETGVFGRVVENLFKRFSVEGDMENRWRVAILEIGRIEGITIADFGNKCEDITKIVKHVTHHLDMFIVDHPVGVEARVQDLIQLLNCEKSKDVLLLGIWGMGGSGKTPIAKVTCNKLCRDYKGRGIFLQNISEVWKKDNGQVYLKEKLLSGIHETTQENNKSGGKKLMERLRNERVFVVLDDVDRLEQLNSLCGSREWFGPGSVIIITTRNKRLLNELGVDYVYQMKEMDKSESLMLFSWHAFKQPCPRDDFRALSRDVVAYCGGLPLALEVLGSELYGEREKVEWRRTLQGCKIIPHPTIYKVLKRSFDGLSDSRVENIFLEIACFYIGLDQNEQRIYDVFLSFSKDTPPSFISRLTDSMKKAGMSFYKNDDSLSGDDQISRSLLAVIQKSRIFIIVLSKNYARSTRCLQVLEQITGYRRIGGQFILPVFLDVDPKDLRLQREFTQAFRDLKENVLDKAAVDRWKIAFARIFQIRSFKFVYEAANGTNRSKDDDVALRVVERAKDLLDKRNLFIVEFPFQVDFHVTEATQLLTHRKSRGALLLGIWGMAGIGKTTIAKAIYNKIGGDFEGRSFLPGIREMWEFEKRHAYFQERILSDIFMLPKIKIDNVKIGKKLLKEKLSHRKVLLVFDDVNKLEQLNALCESHEWFGPRSVIIITTRDRALLRVHHVDHVYNIKEMDESKSLVLLIKILKGNFKIDSNTSSQKLVACAARGLPLALEILGSGVSSTSDMVNMSLESPHPIVRNVLERCINDLSCLQKSMFLDIACFLIGMDREEVIQALNVRRNLAEDGINTLEDQSLVTFDKQNKIRMNLVTTSKAVDFMCKMINGSINFCIVFLFQPWIYDVFLSFRGEDTRASFTSHLSTYLANAGFHVFMDDEKIRRGDWISISLLQAIKKSRIFVVVLSRRYSNSRWCLQELESIMVCSEANDRVILPVFYGVDPSTVRKQNGEFGEAFQDLLNRFSVDRYKEESWRKALRHIGGIAGIEIIGSRNESEDINRIVEHITGFLGKTFFFVAQHPVGVNSRVDHVVKLLNHRRSKDVEVLGISGIGGVGKTTIAKAIYNQIHRDFEACCCLLDIREKWKQITCQVDLQNQLLSDVYTTTKIKIRDIELGKTILKERLQHKRILLILDDVDDLSQLTALCGSRKWFGPGSRIIITTRDEGLLRILKVDHVSRMSEMDDDESIEHFSWHAFKKASPQEDFAQISRDVAAYCKGLPLALEVIGSLLFDKEIEEWQSVLEKLQIIPNRQVQKKLRISFDSLSDDTEKEIFLDIAFFFIGMDQNDVLHILDGANHPALGIKTLKDRCLVTIDNNNKLGMHDLLRDMGREIVREESIKEPEKRSRLWLQKDVLKVLQKHMGTRAVEGLALKLTRNNTVCFKTEAFKEMHRLRLLQLASVQLDGNFEYLSQELRWLNWHDFPLKYMPPNFYQEGLAAIELESSCLTQLWTEDKLLVKLKFLNLSHSHHLTQIPNVSYMPNLEKLLLKDCPNLTVIPDTIGDLKKILLINLEDCTSLSNLPRSFYRLESLETLIISGCSKIDKLEEELEQMKSLVNLIANRTSITKVPYSIIRSKSVRYISICGYEGFSRDVFPSLIWNWMSPTNIISFSTLIQTSACPEFVPMDVIINLHSLRPSKNKQGVKRVLDILDAAYCKQVEAKRTASQVANIESCSETIFDFPNQFYISGSAKSLLIEVGIKIQITNILRERIIQKLTAGGPDDCLLSSDNYYSLTFNCEGSSVIFEVPHVNGGKLESMMLCIVFSSSLETEILKCLQEVLIKVYTETSIEVYNYKCNRKDRSAEFLDWQSLTSKLEPGTKVEVHVIPGVGFTVKKSEIYLVYNAAPTYFNMENCQADDMAIDENPNVSGDDNMATEIMDDAGSSGVEEVSDVIIVSHTPIML

>arahy.Tifrunner.gnm2.ann1.BC3717.3

MASGSSSGSIPPPTRSWTYHVFLSFRGEDTRTGFTGHLYAALNRKGITTYKDDQNLRKGDVISKELLKAIEESMFAVIVFSPDYASSSWCLDELQKIIECKNQLGQQIEAVFYGVEPSDVRHQRGSFEKALKKHEKRHDSEKVKRWRDALTQVAAHSGWTSKNQDEAVLVENIAQHIFEILIPKLPSSMKNLVGINSKVEQVITLMGLGLNDVRFIGISGMGGMGKTTIARAVFETIRCGFEVTCFLANVRENCEKKDITDMQKQLLDQMNISSNAVYNQYDGRTIIQNSLRLKKVLLILDDVNQENQLENLAGEQAWFGPGSRIIITTRDVEALKGPEVHETYEVEGLVESEALNLFCLKAFKQQKPTEGFLDLSKEVVKYSGGLPLALEVLGSYLNGRPIAVWYSAIERIKKTSHSEIIDVLKISYEGLEDTEKDIFLDIACFFKGDEKDGVTKKLKRCGHDAEIGIDILINKSLITIDIHGCLGMHDLLEEMGKQIVIQESPNDACNRSRLWCSEDVEFVLAQKEKTKATHGIVLRKWYSEAEVIQRDLSLSKMCQLKLLILDDVKAPILCYIPCTLKIFRWRHCPLKILPLTDHQSYELVEIDLPYSEIVELWDGQKVLEKLEYLNLVGCKQLKQTPDLSMAPNLKELDLRECEELDYIHPSLAHHKRLVELNLGCCERLETLGDKLEMSSLEELYLDWCSSLRRLPDLSGAPNLKILDLRGCKELDYIHPSLAHHKRLVELNLGYCERLETLGDKLEMSSLERLDLYSCSSLRRLPEFGECMKQLSKIILAYTDIESLTVRADYDDSDGSSREESTLSYDIAHLASLTDLDLSENMFLRVPISIHQLTRLTRLQLWDCSELEVLPELPSSLRELGAGGCYSLAAWNVDEVISKACCGFAESASQDREDFLQMLITGEEIPAWFEHQEEDNGVSVSFPQNCPSIETIALALCFVIEIEEDIYPIMPSVICNGKEFINASLYPFDGSDNLFIVCVNGYYCSKLLCQHNRFQILSPDDDNIDIRVKRCGARWVFKQDMQDFKKRKATLELNMDISHSSASRNKMLVVDSPIYEEEIEPAATAEASICHLASRKSSDPPQLLPPFPLQEVHETYNVEGLMESEALNLFSLEAFNLPKPSEEFLDLSKEVVKEVVKYSGGLPLALQVLGSYLNGRPIVVWHSAIEKIKQFSHSEIIDVLKISFDGLDDMEKNIFLDIACFFKGYEKGNVTRILEGCGYQAELGLHILINRSLVTINKYDQLEMHDLLEEMGKRIVIQESPNDPSKRSRLWCYEDLNSVLAQKKGTEAIQSIVLNSSSEKHLRFEAFSKLKLSKLLILCGVEVEVEPPSDLPCLPSTLKVLHWWRCPLKTLPLAETEHEFAEIELRFSRIEELWLGKKFLGNLKYLNLSHSWYLKETPDFSGTPILETLILGDCPDLREVHPSLLLHENLVLLDLSKCISLKTFPGILYMTSLKELILKDCRSFENRPEFGECMKQLSRLCLEGTAVRELPSSLFRLPITINKRKSREPALLLDSGQHLASKRKISSSLSLDKISHN

>arahy.Tifrunner.gnm2.ann1.BIGY5Q.1

QAFCSINFCRNEAEEINSVVKEITRFIGNTLLFVAQYPVGLESRVEHVISLLNLQQSEKVLILGILGIGGIGKTTIAKAIYNQIHRRFEGHCFLPDIRESWKQSTNRVYLQERLLSDIYKTTKIKIHNIESGKFILQQRLPGKRLLLILDDVDELDQLRALCGSRQWFSPGSRIIITTRNEHLLKVFEADHISRMSIMDDDESFEHFTWYAFKEKSPQKDFLELATKVVAYCGGLPLAHEVIGSLLFNKKKKLWESVLEKLTRIPNHEIHRKLRISFDNLSDDTEKEIFLDIAFFFIGMDRNDVINILDEYDAEVGISTLVDRSLVTVDKNNKLGMHDLLRDMGREIVREKYPKEPEGRSRLWLQKEVLEVLQKHIGTKAIEGLTLKLTRTGTICLKTEAFKEMKKLRLLQLAGVQLGGDFEYISKDLRWLYWHGFPLKYMPSNFSQTSLVSIELESSSLKLVWKEPKFLERLKILNLSHSQHLTQTPNFSYLPNLEKLLLNDCPRLTAISETIEHLKYILLINLEDCTSLHNLPRSFYKLKSLKTLIISGCSMIDKLEEDLDQMESLITLIADKTAITQVPFSIVRSKSIGYVSLCGYEGFSRDVFPSLIWSWMSPTNNFSSQVETCMDLELVSSDVGKLRSLQVECNSDFQLAEDTISLKCLKNVLIINYTKSTIQVYKCDTLACLEDDEWQSIISSLEPGDRVEIVIVVYGTEFVVKKTSVYLIYGEPNNEEMENSHVKGDRDAIDMPEDKNVITFGKAKNRFRGPLALASVLVSPLFWIGVTGFLIWKHCLSDKRRR

>arahy.Tifrunner.gnm2.ann1.BJ32EB.1

MSVVIDALVGGAIEALLDTVIEIKEKNDKFKPSLEKLETTLKSLKPFIKQIEGIDAKLDRPKSETERLIKRMESGKILVLKCSKLQWWDCCGKANRQEELEELYDSICEFFKLELQAINTRDVKEALVGVRDIQGGVREIQVEIGKLSKMVPRNERVELRGVCSPPKPPAFIVGFDVPLKQLKLKLMDDRIGDHPVVLTVTGAGGSGKSTLAKMLCWDDEVKDKFNDNIFFVTFGKKPKLSTIVQKLFQHTGYETLEFQSDEEMLNQIENLLNQLVRKNPILLVLDDVWPGSESLVDKFEFHIQDYKILVTSRVVIGRFGNPIVLKPLGDADAIKLFQHSASLNHSSSDVPDDVVKEIVRGCSGSPMALRVNGRSLSQQQRVVWQERAKELSRGGSVLHSSSDVLDSLQKCFDLLDPMGIECFQDLGLFPEDQRIPAAALVDMWTELYGDDDTSALANIYKLVNWNLADIVVTRKVESETVDYSYHYVMQHDLLRELAILQTSQKPEAERNKLILDISGNDLPKWWTTEKEYHIKARVLSISTDEVFATEWCNLEPSEVEALVLNIRAAKFTLPMFMKKMRKLKVLIVSNYDFFQMELNNSELLGYLSELKRIRFEKVSVPFLGKAGIQLKNLQKISLFMCNVNEAFENCTTEVSEMLPSLLEINIDYCNMVALPNGISNIVSLKKLSITKCHKLSQLPEGIKNLVNLESLRVSSCASLAELPESITRLRNLKLLDISECISISNLPEKVGELCSLRKLNVRGCSNLSELPSSIMDLGNLRDVICDEETKELWEDLKTTLNGLKIEVVQADINLHWLHH

>arahy.Tifrunner.gnm2.ann1.BPD95K.2

MEFVALAVDKTLGFVAQPVFRQLGYIFFYNSNVKNLVQGVEELEAERIAVQHQVDEALRKGDEIEQRVENWLKQASDIISKTEEFQQDGGHKGTRSWLFPNSMRLRYRLGKQAKKMKTDVDGLLPKADFNGASYRLGPRSMDAALSNIGYESFKSRDETMKSVKAALEDPTVRMIGIYGPGGVGKTTLVKEVAKQAMDENLFYPVILTSVTRNPDPKKIQGEIADMLGLRLEEESEIGRADRIRQRLKREKKNTLVILDDLWEGVDLNRLGIPFDDDGFSQMTIKDIPDFDNSMMKNEKATGEYKGCKILLTSRSKEVLSTQMDVKKNTIFSVGILDKKEAEKLFKKVAGVQGKNPKFERLTTEIVRKCGGLPMAIVAVARALTSMGYLDWNNAMDQLKRQEFMGLQNPMEFSVKLSYDHLGSENLKSVFLLCAQMGDRALIMDLMKYCIGLGIFQGVHTIREARNRTDTLLQKLKDSSLLLDTDSNDHFNMHDMIRDVALSIASKEQNVFFLRNGKIDEWPDMDELQRYTAISIHNSEIIDELPEDINCPLLKVFHIDSDDPSLRIPDKIFEGMKNLRILILTGVHLPHLPNSIKCLKNLKMLCLERCQLGENLSIIGELKKLRILSFSGSEIQNLPNELGNLGKLQFFDISNCTKLKGIPPNIISRQSSLEELYMRNSLSQWETEGQTSQTQSAILGELRHMHQLTTLDICISNAELLPTNLFFDKLINYKIVIGDSNMLSLGDFKMPHKYEVSRTLALQLKEPTDIHSQKGIKMLFKRVENLLLSDIYGVQNIFDELNLDGFPELKHLSIVRNSEIEHIIIESIDFSHPHNAFPKLESLCLYELNKMEKLCSAKLTTNCFSNLKSIKISCCGQLKSLFAFCMVQSLTKLEAIDVAECCSLEEIVGGGTDESNNNSVKVNRLKLPALRSLTLRSLPRLTGFLTNDNIYSEPQLVEEQVSDGGFNEITVVEESASHYLLNDQVAIPNLESLELLSINIHKIWSEQSPSLPLTHFCFQKLIKLNVKDCNRMAYLFTYSMATSLPNLKALSASGCKRMQEIFIYDNSNKDETKISIFPKLEEIQFSGMERLTKIWPSRVTSKSFGSLTTVVIEKCKNLVTVFPGHTVGRFQKLISLKVFDCESVKEIFDIQNSTPQGLSGYETYLQFFYIKELPSLKILWNKDPEGILSIKNLQILEVYGCHNLKYLFPFSVAKDLDKLQCLTVLNCNEMEEIIPMGKKSTASSTLKLPQLSFLQLQQLPKLKNFFGGGAIGLPELKQLAVCYCENLEAQPIFGDEKVISKLEMLTMTMTQKGSEWLNQWISKCRMNSLKDLRLALLQNIKILYEFLHKIPNLEDLTLYSCQFKEVLPHGSLAAPKKIGTVVQVKGLVLWDLPDLQKIGFERDPVFQMIERLRVHRCPRLIDIVPASVSFTYLTLLEVSDCNSLINLMAFSTAKSLFQLTTMKIIKCNKMEEIVRKDGTEEETEDVVFSKLMTLEIASLEKLKSFCCSRNCSFKFPSLEKLILRECPSMKVFSAGATSTPKLLKVQVAEEKEKWRWVGDLNGTIGNIFTEAFATGVEHLKLGDQSMLEEIWKGQVTVPNKWFNNLKSLVIKKCKFLSIVIPSNLLPFLSKLEELEVQDCNSVKVIYDMKDVTEETRSMKNMGQVFPCPFPLKKFILKELPNLEHVWNRDPQGIVSLKNLQDVHVERCKSLRSIFPESVAKDLAQLENIVVRYCEEMVEMIARDGESSSEATKEFVFPSLSSLILWQLPKLKWFPRELKIKFPQLKSLDVHHCLLTSALEIYQTACLEDHASTSVAKVFPNFERLSLNKEETMMICHGRQLEVNLLQKLKCLILQCFHSRCSMFPYGFLQKFPHIEEVEVRCCYFKKIFSMDRPDMNYGGVPSKLKQLKLNTMPELNSIGFEHSLADSILKNLEKLEVSGCPRLVNLTPSTVFFSKLAELTVSQCHELDYLLTYSTAKSMHMLEKISISHCQSLKVVVAREIDELDENEHHEIIFEKLKALYLDSLTNLACFYDGISSLNFPLLEQVSVFECPMMESFCQGNAVSPNLSGVKFHGHMSKLKLFGVEYEDHADSHWGTDLKTIISEAFKETAAEFLSHVNHLKLGDHSELKLKDIWFGVVQVPDTCFNNLKSLILENCRFISNVIPSNLLSFLSSLEELEVRNCELVNVIFDVKDITGDKSNMNIGQAAPLQLALKKLKLENLPNLMHVWSKDPHGLFRLPALQVMHVNGCSSIKSLFPVSVAQDLLNLEIIEVSYCGSLEELFQPDIGAEEGITIKFLFPCLTVLILGNLQNFKYIYPGKYEVEFHMLKSLKVYLCKKFTPIFRIDHQKHPDYEHHQNHPDGEFQEFQDNQQALLSIEQVAASLEQLSLNTEDIVMIDQSISQFDLLNKLKALELQCFEYESTFPFGLLQKLPNTEKLQVSHSSFKMIFPPEAGPWLSSCIDDTSYNHEDHSNFLSFTDQIRYVLAALQHAGIRPIDNEHDAKALVPKLKELYLNALPELRAIGLENTWVDVLSRGLKTLEICRCPSLVNLAPSTVLFSSLTNLTVKECGGLQYLFTLSTAKSMHLLENMCICQCESMTEIVDKEEDESDLQVDITLGRLNTLYLDSLENLVSFYAGNSILKFPSLEQVSVFECPKMKVFCQSGVDTPKLMGVEFSNHMDEPCWFGDLNITTWKQSEQMITKFACEVQHLELSEHPELQMFWNNVVSVPDTCFENLESLVVESCEWSSTAIPSNIHSLLSSLKELQVGNCNCVNAIFDGRDIRGDRRMKDLEPALFLSRMILNQLPNLEHIWNTGLQGVAGQQQSEEVYIDGCDKLRSLFPASIEKYLMKLEKLDVKRCLRLEEIVGRDKVCTGAIMECILPAVSFSSLKELCLSDCPRLIYVFSSSTAKSLVQLEKMHINKCKSIKEIVAKEVNDAIQRQIIFGQLKVLSLNSLPSLASFYEGNSTLKFPELVEAIIYECPKMKIFSPGLETPKLEAIQISPQASDKRWEHDLNHTIKAFIAWKVTKFACEVQFLKLIDHPELEDIFCGVALAPFESYFSNLKILVVEGCEFLSSVIHFKLFPFLNNLEELQVRHCDSVKEIFDVEGLMNNVVPSASQSFPRIKKLTLENLPDLQHILRITDHQGIFTFPDLEKVEVRECKSLISLFPAKIAKGLVKLEMLHVIHCAGLVEIAAKDEAATEAKNELFEFPNLKWVILLELPELRHFYPGPHNKECPKLKELDVSCCRNPKIFSTAQDFGENYQLFSWAKRDFSNAGFAIVKISIRADHHPVAVIGRFELYFRSPDPVR

>arahy.Tifrunner.gnm2.ann1.BW2M8B.1

MEEQTHHHQYHRKQTSSFGDSKIVPPLVLVQHCCSSSLWFLFFFVTITTFFITTTNAQQAFDYGDALSKSLLYFEAQRSGRIPYNQRVSWRHHSGLTDGLEQGVDLVGGYYDAGDHVKFGLPMAFTVTMLSWGAIEYLQHISDAGELQHTLEAIKWGTDYFIKAHTSPNVLWAEFGTSLVQEIQDCCCWVLKVGDGDTDHYCWQRPEDMTTSRQAFKIDEEKPGSDLAAETAAAMAAASIVFKNTNPHYSHLLLHHAQQLFEFGDKHRGKYDESVGVVKSYYASVSGYKDELLWAAMWLYKATDSEMYFQYFISKAHSFGGTGWSISEFSWDVKYAGLQLLASKFLNQEKHKKHSDILEQYRSKAEYYICSCLNKNNNDSNVQRTPGGLLYVRQWNNMQYVSTASFLLSVYSDFLKSTNQNLNCHGGTVDHEEILSFAKSQIDYILGSNPMNMSYLVGYGPRYPKRVHHRGASIVSYKENKGFIGCTQGVSSPAPSSTSNNNRRWSYHVFLSFRGADTRKGFTNHLYSALKDAGIIVFRDDKILEVGDVISDELPRAIKDSLSAIVILSPKYATSTWCLEELHCILESKLEVFHIFYDVEASDVRYQKGSFAEAFEKHEKRYDGDKVQKWRDALTQVAGLSGWGSKKRLEAELIEEIVTTVWTRLQPKLPTFRDGLVGIDKKIEDMNSLLRPDVKDVRFIGIWGMGGIGKTTLAKVLYMKIRRKFEISCTLDNVREASGQRHGLLNLQRELLSKLKIMDTKIEDEYQGIDTIRNFLFNKKVLLILDDVSDMSQLENLAEKEWFGPGSRVIVTTRDMQLLTSHGISEKYEIDFLNPEESLQLFSRKAFKRDEPPEHFLKLSKAVIKYAGGLPLTLKLLGSLLCERSVSQWKEVLEQIKEVPESDSLHRTLGISYDGLPRRYKGLFLDIACFFKGWTKDQVKQILNDCGRYPPIGIEILIEKSLVTYDSGVLGMHDSLQDMGRSIVFEESPEIAGKRSRLWSLDDIDHVLRKNKVNESTQGIVLELWHLHHEARWHPEGFSNMEILRLLILSSNLHLPLGLKCLSSGLKVLVWRECPLNALPLGVPLDELVRLEMPHSNLKQLWNGMQYFAKMKSIYLSHSLSLTSTPDFTGIPNLEILYLDECINLFEVHPSLGKHRKLVKVSLADCRNLKKLPRKLEMESLKCLDLHGCTNVRKLPEFGENMIHLEELDLRNIAIAELPPSLGHVTALRTLNLESCQNLICLPKTFGNLKSLTKLNICCCSNFSKLPENLNENEALEYLNASLTALREIPSSIVHLKNLMWLIIAGCKVQETSNSWNIIQPIAQIFGFKSYHPPISMSLVLPPSISGMRMLKELSLRNCNLHDGSIPYDLGCLSSLELLDLSKNNFVNLPDGCFSKLFKLAELYIYDCQSLVSLPDLPPNAAHVYLYNCPRLESLPKLPPTVQRVDACQSVSLKPLSDPEQIWSLLEAIDFEEVEDPDSSMKFSRFRPILEIPGTELPACFENDYFVPDKQFLEHFGIQFESAVSIILEIPESCSQSEFWGIAVCLVIEGNPESAPLQYYDGLYCFSQVHSANSEEKQEINIEDAEWIKWIPNYKCPHILMYYYPVNFWQHDENKVKLMFYAINNNGTGNKMMIEGGSSKKCSKIKKCGARLFRTLAKMGSELRRWRWWCAVFVGMLAVTLTSSEQQQQTVSRIAFGSCSNQSAPQPIWDAVVDFHPQVFIWLGDNIYGDIKHPFKLFGRERTIGPWKNVPRFVPSSEQEMEAKYKKAKSHPGYTQLRQNAKVIGTWDDHDYGLNNAGKEFDRKVTNQKLLLDFLDEPQDSPRRKQAGVYTSYTYGSAGRDVKIVLLDARYHRDPVGSYGTMLGDSQWSWFEKELRGPPTAITIIASSVQVIPNHTAITLPFFDMESWTRYPKERDHLFKLIADSKRSGVFFISGDVHFGEISRYDCAGDYPLYEVTSSGITQSVEGTLPDFFHFVVRLVSWLVPSTMRVKNKNCRYKSCIYGQPNFGAIEINWESQPVTLKLEIRDKNGRPVSGVNTSLRELQASNSQTCSTEKAAGCNCTPESSLPWIVRYRLAILVFVSLALLLLVLVLLLYAVIKIARQGICSKRKPD

>arahy.Tifrunner.gnm2.ann1.BXSS78.1

MQTAGMATQQASNSCSFPSLTERDREWEFDVFINFRGPETRYGFTGYLHKAFCEKGIRTFMDAENLISGNKILETFDRAIESSRIGIIVFSAHYADTDFLLRELVKLLECSRRKGQFILPIFYYMDPGDVRHQRGSYEKAMAVHEERFKDEVPIWRAALRDAANLCGLHFKGDEFEYEFIERTSKQVLAILKEDTLPVVADYPVRAESQVKSFSNPRQYQHHVFLNFRGCDTRYRFTGSLYKALQDKKIHTFMDDVGLHRGNDISRTLIQAIKGSRIAIIVFSENYADSSYCLDELVKILECHESDGQFVLPVFYDIYHDHVRYHTGSYGKAMAKHEEKFKDDLSKVEKWKQALFQAANFTGFVFEGKQYEHEFIGKIVEVVSREIKRVALPVADYLTGLESQVSVVKSLLFSDGYDGVQVVGIHGNAGSGKTKVAHAIYHLISNGFESICFLENVRENSYKHGLVYLQNMLLSNVFERKNLKATSFEQGISTIKHWLQQKKFLLILDDVDKPEQLQALAGKPDWFGLGTRVIVTTRDKCLLESHGIKRIYEMENSNSESNINASPGKDNREQLGEKSTSGDQVNGSLEPLVSEDISLERSKQASLIQAESNTEVIGCNENQADICHTHFPERIDNGKKEVPNGNKEVDMAEKCFSAQLESLEEKKRELEEEIRAIKAEIAGFQRRDTVAKEKKRSV

>arahy.Tifrunner.gnm2.ann1.C2BH8Y.1

MATHALLGILIGNLYTFVQNEIAALSGVDSQIQHLSENLSAIRALFQDAAEEQFTSHVMKDWLNKLSDAAHVLEDILEDCSTESNRLQSEGWSARFHPKTILFRHAISKRMEDMVKRFQRIDDDRRRFQLPLGVRQRQQEDDDLRLTISAITEHQMYGRDQDKHKIVDFLTEHASSIHGLSVYPVVGMGGLGKTTLARWVFNDDRVIQHFDLRIWVCVSTNFNMMKILQSIIESSTGVNPNLSSLAAMQNKVQQVLLDKRCLLVLDDVWENDKWEDLMSVLNSRDSETKGVSILVTTRDQTVASAMETCPTQSHHLQPLPKDDNWSLFTHYAFGPNKEQPAKLVEIGKEIVRRCAGNPLASKVVGSLLRNKKEEKQWLNVLESKFWDIDAVMGALRLSYFHLNPSSRQCFCFCALYPEDFRISKEQLIHLWMANGLIKSRGNLEVEDVGNQQWEELLQRAFFQEVSIDKYGNTTFKIHDLFLDLARSIVGEEYKAYGESESLINFSRRVHHVSYSGLPELNQKTLKNIESLRTLIDLEPAISNTFFGHPVLVLHKVQLCNSLRALRTRSSELSALKSLTHLRYLNIYNSYITKLPKCVFRLQKLQILKLEQCYFLTCLSKHILKLKDLRHLLIEGCRSLVEMPPKMGELKQLKTLNIFIVDSKAKHGLAELHDLQLGGRLHIKGLENVQSEDDAREANLMNKKELSYLYLSWNSDSNCISPERVLDALEPPPNLKNLGINGYRGSQFPGWVRNTNIFSSLVNVILFECNNCEQIPPLGKLPHLESLYVYSMKDVKYIDEDSYDGEEEKVAFKSLKELTLIELPKLERIIRDEGVEMLPLVSKLTISCSPNLKLPLLQSVEVLQIQGLESDNEVVASFPEEIFLSMRYVKQLRISIFPKLKVLPQELGTLSSLQELDIVGCDELESLAENVFQGLSSLRRLDIYDCPRLKSLSSVVEYLTCLESLRILFCPELTTLPTNMNKLTALHDVVICAGEDNGRVPEGLQCIPSLKYLMLDEVDSLPEWLGDMTSLERLVIRLSPRIKSLPSSFRNLTNLRSLTIEKCDGLEQRCQRETGEDWPNIAHVPHVELIPTQQQKHTSWVQDKVAVYACIHIGRNLLLLIARKHQPTHILPLNKTWLCCVFGNVLLT

>arahy.Tifrunner.gnm2.ann1.C4WT98.1

MIFLREEESQDDHQLIRKVVNDVLKMRILKHPILPTDLVGIEEIRKIVKVNMKQHRVIGIWGMRGIGKTTIAKMLFAKSFPHYDHVCYAENAKEYTPQRLLSELLRERITIDATGFVNSMSSLSNKKVLIILDNVKDSDQPLLEAVCQGYKSHSRESKLIITTTHKHLLEKRVDWIFEVKQWDDSKSIELLSLKAFEETIPPKDYEILVNKIVKYAGGIPLALNLLGSYLRSRSIEFWESTLEKLQKHPIKRIQAAFTESYDELDDLDKEIFLDIAFFFHGEKKDLVTSILKACEFSPRRGIEILQDKAMITTLPYKETIEMHGLLREMSYEIVHEENSKDPRKRSRLKDTKEKTFKPLDAIEGIILDLSQIKDLRLSPDTFKRMNNLRFLKLYIPSGQSSGNMILPTDLEPFSDKLRYFEWHQYPFISLPPSFYAKLLVEIRMPHSHVKKLWEEKQELNNLEGIDLSECKELEELPDLSGAKRLRWVNLSGCETLPILHSSVFSSNTLVSLILDRCTNLQCVKAERHLNSLQHISVKGCSNLKEFVVSSDLIENLDLSNTKVEKLDKSIEKLQKVECLNLEGSRVKHLPKELSALKLLKELKHSYSGLEIDKHQLQGLFNGLSSLQILHLKDCSHLFEFPDNIGSLSKLRELRLDGSNVTWLPATIKCLQELEILSLNNCKFLETLPELPSSIKEFSADNCISLESVSALNTLATKMVGKTKRISFNNSLKLSAGHTLHSIMESIHSTMVSAVSSNVTVRSYAIDVHSYNYNSVEVCLPGDTIPEQFAYKTEKSSSITIELPESPSNFLGFIYSVVLSPRHGMEKHGAKIRCEYNFAGGKNSSWEDITISELNSDHVYIWYDPFLTDKILGQYGPSFHLEFSVATDTGEVDDSIIIKECGVHIINELELQRFLLELDKKKKDMEEESSAQHLHFHPQHGSQDHSHVQTPPQDKKKERFDEKQSNEIENQISKQKGEMNEQSSFDRKIGKGNEETPTEPDAALPLCIDSVKNNVFQDISNENYYDLPDSMEEAKHLEEKLQEVHHNVHDMTADEPYKEKQGEPMDLETSESQDHTSNVEQTIELAQPINSCIPPHGKKRAPKLTVKELRHKKAKVEGNPARATQATNLAMETSDFSRSNIKGKRIEDIHLNQVPAQNASTSIAQTHDKITIPSLDDPQVALELLKHFDASAKRLQITEFVDNHVLHPYDHSITKDLDSALCRWLQVQGLRSVSVARYAEMKFEAAKREREEELRLAREENAKFEEALKRMEDRISHVESLEKRVEDLNSEVASWRSKYEETEKSLKETEKKLEDEKDVGAQKETRWKRRESELITEAATCSFENCRSQVSILYPDIDLSRLGPFKEIQNGQIVSPSDTEETESEEDTRAFDDDAHGDAGV

>arahy.Tifrunner.gnm2.ann1.C6P5C3.1

MADNGAALSSSSSSSSLSEGMYDVFLSFRGEDTRYISSDSLYRKLAENGNLKVFRDCPGLKLSDPIKSTLVEAIKGSRMFIVMMSSNYVSSSWCLVELVEILKYSNNGRNRAVFPVFYHVEPSEVRYQNSVKSKEAMRKHEERYGKDTVAAWESALSTICGLCGQHIVKNKGYETEVIDKIAEQVLAKNREIKQLLNRFYSQFEEVESLLDLKSRNTLRMVGIYEEDAEIDKTTFTLELYYKIKHEFKEASFLLGVSKTLEESADGLKNLQKAILSDMGVKVSTVDCTSTGSSEIKRRLRHKRVLLVLNDVDSKMHLELLARSGDLFGPGSRIIITTEDKDLLDNYPVIDGVETKTYCIRECDEFEGNKSSNHVVKGEYVVGLKKDFNDVIKQLMEEDSRDGNIVSIVGMGGIGKTTLARKIYNSDEVKKLFACRAWATISKDCREKEVFKSLLNCLKSSTSKHEDSSSEEELMQKVRKCLTGKKYLIVLDDIWDTKAWATLKGCFPENNDGGMILITTRNDQVAYFLRSKKPHHKLSFMDKEESWKLFCNEVFCREKCPPKLERIGRSIANTCSGLPLAIKITAGFVAKTKRSGDEWKRIKKLLPHLRIVEDKECKKMKERLMLSYDDLPENIKPCFLYLGVFPEDDQICARDLIRLWITEGLIEEPIQSGRSKATPAELEDIGEQHLKELVDRNLVQVSQRRSDDKGVKTCQIHDLIRELCISESKNPDNNNNNARRLSFPKDIGSYACLVTCSQSRTCSLFVYGDAEGWSHHIPKGYRVIVLYFKGSKMDIISGKNAGFLKGLKSLRILRVEFPDPYRFYPLHRMHILHILEHSAEDINIEGLKQLRHLRSRYAVHLLVDEEGVKEKMQNLQTLCYVHLDSRLEFLLDNGYFPNLRTLGLHSAERKLSCLRRLSNLRELKLEHMTSKYVPLDKNVFPSNLTKITLSWYVGFSTKAMNALGQIPTLQILKLYEVQCMEGILNCGTERSFPRLQVFIMKKVQVKGLTLEGGAMPCLQRAVFHECPGLKLEYLPEQMRSSGCNLEFSEHVEQRRSWVVKTWSFQNMNKNKKVMMIPIMMMMMMMIPMMMKMMMID

>arahy.Tifrunner.gnm2.ann1.C8YHPQ.1

MASSPSITYDVFLSFRGTDTRRGILSHLVKAFNQKQIETYVDYMLREGTEISHSLLTAIEQSQISLIIFSQDYASSRWCLDELVTIMKCRKQNGQIAIPVFYEVDPSWVRHQKGSYQDAFANHEKTSSQDKVQIWRQALNQAANLSGLHSSNFGNDAEFIEEIVKRVLQRLNQAYQCDPKEFVGIHEPIAELESVLCRESKAVLVVGLWGMGGIGKTTLASNIFNRLQYEFQGVCFLENVRERVQRYGMTHLKKELLSKLLEEKDVVPFIMPDGITNFAKRRLSRKAILLVLDDVNDPDQLEDLCGRGFEWFGPTTRIIVTTRDKHVLLVKQVDHIHEVKPLNDDESLKLFNLNAFKQNYDIERDQDHAELAMKLVTYANGIPLALKVLGSSLYGRSKEEWESQLSKLEKIPHVKIQNILRVSYNDLDRHDQNIFLYIACFFETDNAEQIKCLLDSCGYSTAIGLRNLHDKALISIAPKSMAMHDLIRDMGREVVREESPSNPGNRSRLWDPVDIYEVLKYNRGTETVESITFDMKNIDMYLSLHPRAFARMYKLKFLRISCSWTRHGECLLRVPQGIESLSDELRLLEWNACPLKSLPSSFCAENLVKLMMMDSGLEKLWDGVQNIVNLKEVYLQGSRKLMELPDLSRALKLQVLNISECVSLRQVPSSIASCHNLLELNLSRCYELGSFHHCIYLESLKILFLDSCINLREFSLLLSTDSDTLPAKATAAMALENISLVDCCNLSLLPHNIGMLSSLKYLSLCRSYITCLPESIKHLSLLKSLNLRGCQMLQSLPELPSSITELDVIDCTSLETTTPSTFAYSDEQIETEIFAFANCIKLDHLTKKGIMEDACMRIKRAANTCLAAIKENEDDDRRALLVNPHYHYKVQGCLRENEIENEDGREYQYHHRVQFCLPGKQVPSWFTHRSRNKETMMTIELVLPHRASNDRFLGFILCVVVPKKGSSGILGCKYCIESVEYSSWWNPSPRVGVIDNVFLWYVGECCIDIVREMKENVCKLKVSFQFSIGGKIWTRDISQCGVHPIYASNVFKQGVVVTHMDDDNKNECDIEEQEQLP

>arahy.Tifrunner.gnm2.ann1.CA7JWP.2

MAAELVGGAFLSSFLNVLFDRLSDPAIINMMKGKKVDQKLLQKLETILSVVEAVLNDAERKQISNPAVKGWLENLQDAVYDADDLLDEIATKAATRKDPPPGNFLSRFLNSQDRKMVTRIEEIIARLQDIASHKDILGLEKISVKNMSGRIESTSLVQKSDVFVGRDQEREDIVKLLLDDSNDGKLSVIPIWGMGGIGKTTLAQWVFNDDRVQQKFDVKTWVCVGEEFDVLKVTKTVVEKATSGPCNLNDLDSVQLWLKNELAKKSFLVVLDDMWSNNYMAWKKLLTPFQCGTEAGRHGGKILVTTRDEKIANMVKSHHHQAHNLSVLNDEDCWSLFANLALLSKDRLGFEKVGREIVKKCRGLPLAVQTLGSLLSTKDDERDWNDILNNEIWEFSEEESDILPALRISYYHLPSYLKRCFVYCSLYPKDYEFDRDELMLLWMAEGFLQQPRSGSILEEVGYEYFNDLASRSFFQHSNDGYENSFVMHDLMHDLATFYGGKFYFRTIELKNVLKHDTKTRHLSYGLRNDSVSKIMDVCESLKHARTLMQINLDRRDDFSVRIIDPCHLLEELKCLRVLSFKSFSHEEDLLHDSIGELIHLRYLDLSYTSVVTLPDSLCDLYNLQTLKLSNCKKLKKLPSNMQNLVNLRHLDIKGTELEEMPKGMGKLKDLQILRYYIVGKHEENGVGELGELVNLGGSFRIEKLENVVNSREAWRARMVDKKYMSELCLEWSSGEDSEMVDSQIEKDILAKLEPHKGLKELRMKGYRGTMFPDWVGQSLYHNITELVLSGCENCWVLPSLGQLPSLEWLEISDFDKVKMIGGEFYKDDGTDHHCEIPFRSLKTLFISGMPCWEKWESFECDDDDAPFPQLEVLSIWDCPKLRGDFPTFLPSLKDLGIARCEQLGCYLPRAPIIRQLRIFDIQEARMRDVPLSTLESLSISGEQQVEYVFNAMTRTQPTSLTWLKISNCSSAISFPGDSLPPSLLSLYIIDCKNQDCKKQIGSSWSKRNSWKTSFSTKSKEEMDDVEQNFARIDRPIKMQQEHLQLFFFFFFL

>arahy.Tifrunner.gnm2.ann1.CD0F2D.1

MAETLIEIVLKNLITLVQSEFAAFSGIREKAEELTLTLELIKAILDDAEEKQWSNRPLKVWLQQLKNAMYVMDDILDQLSTQSSQPGCFSSLNPKVIHQRELGKKLNKMIVRLDRIAQRRSNFDLRQVVRKRSSEVAEWRQTSSTIALPQVYGRDEDKKQVVDFLLSPSRSSESLSVYPIVGLGGLGKTTLVQLVYNNQQVANNFDLKIWVCVSENFTLVSILRSILKAMEVDKSEVMNLEEMEKKVKVLLQSKRYLLVLDDVWKRSQEMELGLTQDKWDKLRSVLSCGSKGSSILVSTRDKDVATIMGTCQAHHLDRLSDDACWSLFKLRAFGADKEERAELVAIGKEIVKKCGGSPLAALALGAVMHSRSTEKEWLEVQKSELWSLPDENDIMPVLRLSYSSLTPTLKQCFAFCAVFPKDTEIEKQELIYLWMGNGFISSRPNLEAEEVGNMVWNELYQKSFFQDVRSEDFSGNIYFKMHDLVHDLAQSISEQECICLEKQNLNDSSRNPHHIVFHDIDKEQFKKRAFEQAESLRTLYHHQLNSDEFPSVSRLIPTNHSLRVLSIYGRKIPSFGSLSCLRYLELCYLDIKSLPASICNLRRLKILKLKDLWSLRRLPKHLTRVQNLRHLIIDECGSLSRMFPEAHKLCDLRTLSVYIVKSEKGHSLVELHGLNLGGKLRIEGLGNVGSISEAEDANLKRKQDLTELSLSWDKRKSVVGAEEVLEALQPHSTLKLLTIEYYEGLHWPTWMQNNSATHNLVSLRLEYCGYCRHLPPLGKLPFLKKLVVICMKDVQYIEEDESYDGFEAKAFPSLEELEVSNLPNMERLLKRETTHMFPSLSKLEITDCPKLQLPCLPSVKDLTVLYCSNEQLKSISNLNALNQLHLFQSDQVSCFPEGMMNNMTSLATLLIVSFSELKELPSDITKLTALSHLTISNCGKLECLPEQDWEGLSSLRKLSIDNCKCLGSLPNGVRYITSLEYLSISGCPMLKERCKKGTGEDWHKIAHVPHCVLVKRSELGNSM

>arahy.Tifrunner.gnm2.ann1.CDY884.1

MSLAASKRAESSASMAVNRGIDFTAMLAELSALFDAAGDIHFKYQLPGDDFDALISVSSDSGLNSLMLEYDNLYRNSPKTARMRLFIFPGNGPDFASTQPFPPKLKSKVNGGAVVPPQITPVKFQDLPPVNNFNTLDRVLDSDPKVNRLVEDSGSSRLNNPLPALQYDVFISFRGEDTRASFTSHLFKALSRKQIVTYTDDLLYEGDSITSLLLRAIEESCLFLVVFSENYASSKWCLQELVKIMECKKEFGRLVIPVFYNVDPSHVRYQLGSYNEAFKKHLQNNKKAEVQKWREALTAVANLEGLDSRSYSLIAFNEDYPQRGYEELSWKAVGCCKGIPLALIALGSFLYSKSKTEWHSALQKLEKTSDPEIQNILRLSYDGLDDEAKQIFLDIAYFFKGELVEYVVNLLDSCGLYAAIGMRSLLDRALIAISHNCVRMHDLIQELGWDIVCQQSSRNPENRSHLWDSNDIQDVLGNNKGTDSIESIVFDMSQIADLQLNADTFKKMPKLIFLKLYIPSESDGRLNKLQLPVGLKPFPYKLRYLEWDAYPLPSLPLNFCPEKLVTLRIRNSKLKRLWDGIQVCTQCMFCHLFDGWV

>arahy.Tifrunner.gnm2.ann1.CFS3CL.1

MSQVCSFFSKSVIPILFRAKLARKIENIQKEFNNVAEEMSKLNLNRSSVILKQDECGWRETSSSVLQSEIIGREENKSDIVNLLKQTHPNQNVSLIVVVGMGDLGKTALAQLVYNAAKDQNLFQKYIWVCVSENFEVKTVLKKILESLKKDAGDYFQKMYQRWIEINVVGIELLDLLLVWNTSDVITSLNLVGAPFQSLQYLPRKKKKFAVQARALSGFM

>arahy.Tifrunner.gnm2.ann1.CN3IG1.1

MSHEVVKYTGGLPLALKVLGSYLYGRNVSAWHSAVKKLRSVPDAKILETLRISYDGLDSMQKEIFLDIACFFKGKPKDKVLDLFEKRGYNPQIEIDVLIERSLVTVKQDIDVFKKKFDVLEMHDLLQEMGRSFVIQESPNYPSKRSRLWSPEDLDLMLTQNKGTETIQSIVLPPIGNGTYYVQRWRDKAIPNMSQLKFLNFDFLRAHIHINIPSTLKVLHWELCPLETLPLVDQRYELVEIKISWSNIVQLWHGFKFLEKLKHLDLSCSGLEQTPDLSGVPVLETLDLSCCHCLTLIHPSLICHKSLLVLNLWECTSLETFPGKLEMSLLKELNLCDCKSFMSPPEFGECMTKLSRLYFQDMTISELPISLGSLVGLSELDLRGCKKLTCLPDSIHELESLRILRASWCSSLCDLPHSVSVIPFLSILDLRDCCLTEESFPCDFGQFPSLTDLDLSGNHFVNLPISIHELPKLKCLSLNGCKRLQSLPELPSSIRELKAWCCDSLDTRSFNNLSKACSVFASTSQGPGEVLQMVIPGANIPSWLVHRQESNCLSVPFPHHCHPSERLGIALYFIVRPSERWFSSSLRLAVGNGDRVITSSIPIWYHQGYHLCMFCMTNDCLSDQETRKAIHFELSFEYINVEYPPEILSSAACWVHTDEIEHLNKGETERPNKKRQKMLEEKH

>arahy.Tifrunner.gnm2.ann1.CQ0716.2

MKLPSSMENLIGIDSRLEEVVRHIGLGENDVRFIGICGMGGVGKTTIARRVYEAIRSEFKASCFLSNVRETCKRSSIVQIQKELLARMNINLDTTIHDEFHGRAAICDSLCHRKVLLVLDDVDDGSLLKNLAREQNWFSLGSRIIITTRDRHVLVRHGAVNRIYKVEGLKQNEALELFCLNAFKRPKPEEGYMDLSTEVVKYCGGLPLALEVLGSHLCGRPSDVWHRAIEKIKSFPDDKIFNTLKISYDGLGHMERNIFLDIACFFKGRKKDYVTNILNRCGYHAEIDIATLIDKSLLTIINDEYGNIFLGMHDLLEDMGKHIVKQESPNNPSKRSRLWSYKDVDLVLAQNKEIEAIHSISLYNMDWEIEGSWRDLDIRDLSFSNKCQLKLLILDGVEAPILSDIPCTLKVLTWRGCPMKTLPFTDHQRYELVEIDLSHSSIVQLWDGKKFLKKLELLNLSYCKLMKQTPDFSGAPNLKTLHLEECKGLNYIHPSLAHHKSLVELNLRECGSLETLANKLEMRSLEKLDLNWCQHLRKLPEFGECMTKLSILSLSYTDIKELPRTLENLVGLSELHFRVWEYIPVSLGCFVGLKKLELSGCSELSCVPYSTHGLESLAVWGWHDRLLGSLSLLPSLSSLQLRGRFFGSKESTPYYDLSHLTSLTDLDLSVNDLLRVPINIRELPRLIRLKLNRCYKLEVMPELPSSLRELNAEDCHLLDASNVNDVISKACCGFAESASQDREDVLQMWIHGKEIPAWFEHQEEDNEVAVSFPSTENIALALCFLLDSYAYEVKPSVICNGEKFINKSFLEIGFHTCPQHLFIVCLNGYYLSNLLCQYNCFQMLFPRIDNTAVQRSGARWVCKQDIQDFKKRKSQNREKKSNSLN

>arahy.Tifrunner.gnm2.ann1.D6W861.1

MENYVMKFLCSYGREIHHRSNDKKISYVGGHNKLHYVNRGIDFTAMLAELSALFDAANQVLDSDPKVNQPVKDSGSSHLNSPLPALQYDVFISFRDSITSLLLRAIEESCLLLVVFSENYASSKWYLYQLGSSNEAFKKHLQNNKKAEVQKWREALTAATNLEGLDSHSYRVSKQRNFIVLDDVSSLEQLDYLVQKLRWCGAGSKIIITARDKNVLVPTVETIYQMKILDSHESFKLFSLNAFNEDYPQIGYEELSWKAVGCCKGIPLALIALGSFLHSKSKTEWHSALQKLEKTPDPEIQNILRLSYDGLDDEAKQIFLDIASIGMRSLLDRALIAISHNCVRMHDLIQELGWDIVCQQSSGNPENRSHLWDSNDIWDVLGNNKGTDSIESIVFDMSQIADLQLNADTFKKMPKLIFLKLYIPSKSNGKLNKLQLPVGLKPFPSKLRYLEWDAYPLPSLPLNFCPEKLVTLRFWNIKLKRLWDGVQVCTQCMFCHLFDGWV

>arahy.Tifrunner.gnm2.ann1.D8E7V4.1

MAEKLYGGAYLSPFVDAVLDNMSSILEDDSVLYGNESSLELLGRLQNCLYDVGPVLDDAELKQFTDKRVKKWLVDLQDALYFADDLLDELSTKAAIDATQRELGNSSSWSRLVDSYIEDSGDLEKIVRRLESVVARKNYLRLKETAKVDMSWRIPSTCLVEPSEICGRKEDKDAILKLLLDDDDASDGDISVISIVGMGGIGKTTLAQLLYQDDKVKENFDFRGWVCVSEEFDVVKVTKTIIEEITSSSCNLTDLNLLQLDLKEKLSRQKFFIVLDDVWNENYDDWNKLLKPFQKGVKEHARLSTVFVENATLKKIRRDIVKKCDGLPLAAQALGGILRGNSDLRYWNHLLKSEIWELSSHRINVVPALRISYYFLPSYLKECFVYCSLYPKDYEFSKDELMLLWMAENFLQPVGNQTMEEVGSEYFDELIARSFFQPHSTREKIFVMHDLVHDLAMTCAGEFYFRAEELRNAVEVDIKARHLSHNAEGNYPMSNLLGVCDRVKRTRTFLEINLWSWIPFNMENAPSIMLSQLKYMRALSLKRFPIESVPDSIGELIHLRYLDLSWTDIVALPESLGNLYNLQTLKLYWCTNLKMLPVGMKDLVNLRYLDIRGTRSLHEMPKGMSKLKNLQFLSDYVVGKREENKITELGALANLHESISIVKLENVVNSGEALEARMSDKDGIDSITLTWSLNEEENRVDSEMERDILDKLRPHANLKELHINGYRGTTFPDWMGHSSYHNITSITLIHCRSCCMLPSLGQLPSLKHLRIIGFESVGIVGAEFYFYENGESCLETPPFPMLETLKFYSMPYWKEWRSLEFNAFPRLRELSISSCPMLSGDLPNHLPSLQSLKIENCVELSCCVPRAPAITTLSISGKHLVGSVVEAIIYMQLSCLTSLWISHCSSHIWFPVSRIPTSLKKLTIESCGELEFEMDGQHHSLQKLSIRNSCDSVTSFSLLDSFPNLKDVEIDKCEKMESIVVSRSLSSLRSLYIYECRSLKSVLTIWMAAPQLEHLTLLGCPEVDLSPTGDGVPHCSLRSLEISYSKKLVSSAAFKNSQFHGLTYLSIHGEYCESVKSLPKEAVSGRSRNPFRGDDRATSMSSVHHV

>arahy.Tifrunner.gnm2.ann1.DEFX9H.1

MAAELVGGAFLSSFLSVLFDRLSDPEIINMMRGKKVDQKLFQRLENILNVVEAVLNDAEKKQITDPAVKRWLENLQDAVYDADDSLDEVATKAATQKDPPGNFLSCFLNFQDREMVTRIEEIIDRLEDIAKHKDILRLEKIAAKNMSGRIPSTSLVKKSDIFVGRDKERDTIVKLLLDDSNNGELSVIPIVGMGGIGKTTLAKLVYNNEKVQQKFHVKAWVCVGEEFDVLMVTKAVIEKTCSPCNSNDLDTVQNHLKNALVGKNFLVVQDDVWSSNCEGWESFLIPFECGSEGGKILVTSRLDTVASMVKTKHNEAHNLSLLDEEECWLVFANRAWDPAESRDCSDLVEIGRKIVEKCKGLPLAAQTLGGLLRGEDSEKVWNDVLNSEIWEFSEEKCGVLPALRISYYHLPSHLKRCFVYCSLYPKDFEFDRHNLMLCGWQRLPKSGSTLEEVGYGYFDDLVLRSFFQHANSNENSFVMHDLMHDLATFYGGKFFSGAFGLKNAAKHDAKTRHLSYDLIDEDSIPKIWEACSSLKHARTLLKTSLIAYETFPDERVDSSHLLEQLKCLRVLSFKFFCYEEDLLHDSIGELIHLRYLDLSGQPVMMLPESLSNLYNLQTLKLRDCSNLKKLPTNMQDLVNLCHLDIHGTDLEDMPKGMNKLKDLQFLSDYVVGKHEENGIGELGELANLHGTFYVQKLENVISSVAASNARMDEKIHLNDLSLKWSSGEDSDIDDSQVEKDVLDKLRPHKGRKKLKIEGFRGTMLPDWVAHSFYNNMTFLELRGCKTCWMVPSLGQLPSLMELRLDGFDMVKIIGAEFYKSDRTHHHHHHQTPFRSLKALFISHMRWWEEWESFECDDAPFPQLKQLSIWECPKLRGDLPAFLPSLKSLYIEDCEQLGCYLPRASIIRELRIYGKQEARMQDLPLSLQQLSIEGNQLVESVFGAMTRTQPTSITGLWISNCSSAISFPGNSLPPSLKELGIMNCKNMEFPMQQQHHHESLIMSFGEKKSETIFTMTSLNTFIGFYYGTAMQHMKKIFQVLDVVILCWLSTWTRSYAYIVESWTTFRVTFAPCLVSHPNLRPFITFLGVALACLLMALIGFASWSKHNLESKGTWARMISQGAVSVSMRKQNAWKTSSLNESNIKGYFAWPSRARIYQNQIGPLLKTA

>arahy.Tifrunner.gnm2.ann1.DFN1EJ.1

MLNLNPKKWKQELEKRKKLPQDVVVALDCSMNRAHWLNNYAITIVRSTKNWSHDAESCSSHFIYDVFLSFRGFTQYGFTDRLYHALCERGITTFRDDENLRVGDRIRDTLLEAIERSRISIAVLCKDYASSTWCLDELVQIMKCWNNGKHQPVLPIFYQVEPSDVRRQRNQYEKNMMKHEDRYGKDSHHIKAWTLALHQVADLSGVTCKVKSYESEIIKKIVEEWNLFGSDNTICMLVIYGDGNKTTFVGELFNKFRLQFEAASFLDKVSEKSVRSADGLENLQETLLYEMGVHKKLKLGSTLTGSSDIKQNLRNKRVLLILDDVDSTEQWFCPGSRIIITTRDANFLNNQVLNGFKIEKYCINEGEFEGMEGARSNQKRDMVVQEDMGISSNPSQWLQLVQVYLKEKKVKEYLKEKKYLIVLDDFWEPKVWDEVQCLFPDNKNGSAILITSRNDEVANYTESKSLSLPFLDKDESWKLFCKKVFRGGECPSVLESIGRSMVEKCSSLPLAIITLARVVAKKKQLTVEWMRIMRNVI

>arahy.Tifrunner.gnm2.ann1.DIS81S.1

MASASSSASIPPPRSCTYDVFLSFRGEDTRKGFTGHLFAALNRKRITTYKDDKNLRKGDVISKELLKAIEESMFAVIVFSPNYAYSSWCLDELQKIMECNKKVGQHIVPVFYGVEPCDVRHQRGTFEEAFKKHEQRHDNLQRNSLSLNRDEAVLVENIAQHIFEILITKLPSSMKNLVGIESRVKQVITLIGLGLNDVRFIGIWGMGGMGKTTIARAVFEIIRSRFEVTCFLADVREHCEKKDITHIQKQLLDQMKINSNSVHNKYDGRTIIQNYLRLKRVLLVLDDVNHGKQLEYLAGEQAWFGPGSRIIITTRDLKVLKEQEVHETYKVEELVESEALNLFCLKAFKQQEPTEGFLDLSEEVVKYSGGLPLALKVLGSYLYGRPIAVWHSAIEKIKKSSHSEIIDTLKISYEGLDDMEKNIFLDIACFFKGDPQYYVRKILEECGYQAKIGLDSLINRSLVTINKYDELGMHDLLEEMGKQIVIQESPNDACNRSRLWCSEDVEFVLAQKEKTKATHGIVLPMDYWYSETKVNWRDLSFSKMCQLKLLILDGVKAPILCDIPCTLKVFRWRHCPLKTLPLTDHQRYELVEINLSHSQIVELWDGKKVLEKLEHLNLSECKQLKQTPDLSGAPNLKTLDLGGCERLETLGDKLEMSSLEILYLSYCSSLRRLPDLSGAPNLKEVHFDGCEELNYIHLSLAHHKRIFFLDLSGCTSLETLGDKLEMSSLRMLDLNSCSSLRRLPEFGECMKQLSILDLRDTGIEELPPTLGNLAGVSELNLSGCDKITGLSSDIGSSREEATLSYDIGQLASLTDLDLSESSFLRVPVSIHELPRLTRLALSDCSKLKVLPELPSSLRKLDAQGCDSLDASNVDDVISKACCGFAESASQDRQDFLQMLITREEIPAWFEHQEEDDGVSVSFPQNCPSIETIALALCFLIEIEEDIDSIKPSVICNGKEFINASLHVASSGDSLFIVCVNGYYFSKRQHNRFQLLFPDDDDLDDDIRVKRCGARWVCKQDIQDFKKRKSQTGKRKATLELNMDMILHSSASTNKMLVVDSPVYEQEIEPAATAEGAISHLASGKSSDPPQLLPLFPLHPSSHSS

>arahy.Tifrunner.gnm2.ann1.DK2LRN.1

MVAALVGGAVLSSTFSVIFNRMSSPEFAKWIKGKKLTQKLLERLKTNLYAVQAFLIDAEQKQIKERAVKDWLDSLKDAMYLADDLSDEVFTKAATQKDPGTFFSRYLNLQDREIANRMEEIIERIESIVKQKETLGLREIPKENMSWRITTSLVERSNIYGREEDKEAIVKLLLDDDDIGDSDISVIPIVGMGGIGKTTLAQLIYNDDKVKENFDFRGWVCVSEEFDVIKVTRTIIEAITSSSCNLKDLNLLQHDLKERLSRQKFFVNGVKGSKILITTRSKKVASVVQTISPHELSLLSDEDCLLVFSKHARLSLDSMENPTLRKVGKDIVKKCDGLPLAAQALGGLLRGNSDVEYWNHILKSEIWKFSNDKIKVIPALRISYYYLPSCLKDCFVYCSLYPKDYEFDKDELILLWMAQNFLQPAGRNTLEEVGDEYFDELVASSFLQPHSTEKNKFVMHDLVHDLAMMFAGEFYFRAEELQNAFEVDIKTRHLSHNAKGNYPISKLLGVCDRLKHTRTFLEINLDWQIPFNMENAPCILLSQLNYLRALSLKSFPLESVPDSIGELIHLHYLDLSETKIVTLPESLGNLYNLQTLKLHWCSNLKMLPVGMKKLVNLRHLDIRKTGLHEMPKGMSKLKHLRFLSDYVVGKHRENKIRELGALANIHQSISISKLENVVNSNEASMARMFAKDGIRYLELSWLPDEDENIADSQIERDILDELQPHCNLKELQIEGYRGTTFPDWLGHPSYHNITKITLGNFMFGPGFRNCCMLPSLGQLPSLKHLQISNFESVEIVGAELYFNQNGEFCLVTPPFPMLETLSIVKMPCWKEWRSSGHNAFPRLRVLDIGFCPMLRGDLPIHLSSLQSLDIMGCDELSCCVPRAPAITRLSISGKHLVGSVVEAITNTQLTCLTSLCISDCSSRIWFPVSAIPPSLQNLRIVDCRELGFQMDGQHNSLQNLWICESCDSVASFSLLDSFPNLVRVEIEKCEKMESIVVSRSLSSLYFLAIKNCGSMKSVSTIWMAAPQLERLDLVGCPEIDLSAPGVPQRSLRYLRISFCEKLVSTAAFMNSQFQGLIQLSIEGECESVKSLPKEGWLPASLESLTLRSIESLETLECKGLAHLTSLHELYIYECPKLENMEGEKLPVSLIQLCISDSPLLGKRCEMKDPQLWPKISHIPAIQVDDRTEIEAADKQRRECSDIGITIGATI

>arahy.Tifrunner.gnm2.ann1.DN45ZN.1

MAGSLVGGAFLSGFINVVFDRLLTKDAVNLVLGKKLDPDLVKRLKISLHAAEAVLDDAEYKQLGNESVREWLNDLRDAVYEADDLLDAVLTKEAIQKEGSSYWPDYFLNREREMVDEMERVVTKIEFLEQQKDFLGLQITMDNNILSSSWRESTSLVEGNIYGREDDQQALLKVINDSSESELSVIPIVGMGGVGKTTLAKWVYNTTEGFDLKAWVCISETFDVVEITRKTIEEITKTTCTLGSLNLLQNKLLEILSGKKFFVVLDDVWSDDADNWKKFKTPFHCGGKGSTILLTTRIKQVASVVQTCSSYFLNELSEDSCWLLFAENACFPESNGNQTLEDIGRQIVNKCKGLPLAVETLGRLLQGKDDAKEWNAVLSSDIWEFPMKNSKIIPALLISYFQLPAYLKRCFVYCSLYPKDYLFEKDELILLWMAEDLLRPPTRGESLKEVGCKCFEELISRLFFKQDLYSYYKMHDLLHDLAIFLAGDFYCSLEEHDKAKDVTTLTRHLSYEDLNHVLSQNFDSITKVKSLRTFLPGSFNIYSLFNEVDGDTINILIKKFKYLRVLSFDSFKNLDFFSFGSFSKLDVLLDSIGELIHLRYLNLSWTDITTLPESLCNLHNLQTLILYECTRLTKLPSGMHSLVNLEHLDLRRTCLEEMPGGIGKLKHLPILDYFVVGRHEDNGIQELAGLSNLHGSFEIKKLENVVDARQARGARMLEKNQINNLLLEWCSDDEMVPNTETLTDILDGLQPQNGLKELKIKGYKGTIFPDWLGGCSYNNMTSVSLESCKNCCMLPSLGQLPSLKSLRIEGFDQLKRIGDEFYKNERDHHSLPTAPFPSLETLEFHNIPCLQEWHVIDPEAFPQLKRLRIKDCPMLKEDMLNGIFLRMVSSSSDSSKVLKLEIRGDHEKRSNEILPRGDALSIRGCESLVKSAFNARSINHLCCLQEVHIYYCLSAVSFPDNCLPKSLQKLTIYWCPKFEFPEQQQHKYDLVELQIEASCDSLTSFSLDAFSNLKNLEIRECRNLESVSMSEAPHAALQRLIISGCSKLVSLAGEGLAAPNLTHLQVTGCENLEALPRDMKSLLPSLQSLQIYGCPNICRLAEGDLPPNLKSLGVGGCEEQMRILSWMGNLDTLTHLTISGSGCVSTIKSYPEVDSLPHLPSLTTLHINNFDNLETLECNELLRLTSLQQLHIEYCYKLENMEGETLPSSLILLQIEGCHLLEEHCKNKHQLIWPKISHIPTIKVLVIFLCGRPKPKLSEVSLHSR

>arahy.Tifrunner.gnm2.ann1.DN6KXW.1

MAAELVGGAFLSSFLNVLFDRLSDPEIVNMMRGKKVDQKLLQRLKTILNVVEAVLNDAEKKQITDSAVKRWLEDLQNAVYDADDLLDEVATKAATQKDPPGNFLSRFLNLQDREMVSRIEEIIARLEDIAKHKDILRLKKIAAKNMSGRIPSTSLVKKSDIFVGRDRERDTIVKLLLDDVNNGELSVIPIVGMGGIGKTTLAKLVYNDDMVQQTFNVKAWVCVGEEEFDVLKVTKAVIEKTCSPCYSNDLDTAQNHLRNGLAGKKFLVVLDDVWSSNREHWESFLNPFECGSEVGKILVTTRLDTVASVVKSKHNEAHNLSLLDEDQCWSVFANRAWDPAESRDHSALEEIGRKIVEKCKGLPLAAQTVGGLLRGKDNEKDWNDVLNSEFWELSEEDSVILPALRISYFHLPSYLKRCFVYCSLYPKDFEFNRDELTLLWMAEGLLLQPKSGNTLEEIGCEYFDDLVSRSFFQHSNSDDNTFVMHDLMHDLATFYGGKFFSRIFELKNTEKHDTKTHHLSYALNIDNDSFMKIAEVRERLKHARTLLQINSATYGRLFTRVTVPCHLLEQLKCLRVLSLQFFLADENLLHGSIGELIHLRYLDLSYTSIAALPESLSCFYNLQTLKLRDCDKLKKLPSNMQNLVNLRHLDIEGTGLGEMPKKMSRLKDLQFLSCYIAGKHEENGIEELGELTHLHGSLRIEKLENVKNSGEASNARMDEKIQLNTLYLSWSSFEESEVCDSHTEKDILEKLRPHKDLKELFIRGYRGTMFPDWVGLSSYHKITKLELRRCGNCWMLPSLGQLPALTRLEISGFDMVKKIGGSFYKGDGTHQHQETPFRSLKSLEFYNMGWWEEWESYEYDDDDDAPFPKLEELRIYNCPKLRGDLPTFLPSLEELWIEGCEELGCYLPRAPMIRSLRIHGKQEARMRDLPLSMLEKLAINGEQQVEYVFEAMTHTQPTSLSYLQISNCSTAISFPGDSLPPSLKELAIHDCKNSLLRRGRIVPQAPQAAFKFGNYTVEAKSYSYSGYQMKIVMWLIPKLKLERDVLDKLRSHKELKELRIKGY

>arahy.Tifrunner.gnm2.ann1.DX2UEH.2

MTTNNQNFVGLNAGEAVIVSEGEKNVWPAIAEGKIQLRQFELTSEAFPESARYIFDFEMAINIVGAITGVMSCLCTQNCLQESLGHHIRHLRRPKRNLNKLKSLLEELDARKVDMCAKLNQMWLQKGMNPKKEVELWLKNVDEIANEVNKIIREVEQESGSTSVLSPFYYSSRIKRGELIEKKMEEVRELLEKGRFADDSLAETSPQKGQTLPTTKLMEHETTMKNLTKILNFILDDRVRIIGVHGMGGVGKTTIMAEINNRLLRENRLFDSVIWVSSSKDMKTEELQKVIAGKFDIDLSGFQDETSGAATLFEAFQRRKKFALILDDLWEPFSLERVGIPIPTVENGCKLLITTRRVSVCRGMETDRDVKVKALTENEAWDLFRDKVGDEALASPDIKLIAKDVAKECMGLPLGIVTVGRALRNATDISEWQISLMGLKASALNIEQMEESVFSRLKFSFTKLKDDTSRSCFLYCALYPEGNHVDANELIEYWMWEGLLGAVDSISASKQKGKIILNELKYACLLENAADNEMECVKMHDLIRDMALTIMKTDP

>arahy.Tifrunner.gnm2.ann1.DX3GFU.1

MAAAIVGEALLSAAVEALVGKITTEISEFYRSKKLDESLLKKLNVTLLSLHAFLNDAEEKQISNPAVKLWMDELTQALFDADDLIDDIATEALRRKVEARYHQSATDKKKLFVVVRKVFRVRKVLSSAFKWSYREINSKMQKLFEELEHFAERAHNLPLKKGVSGSDWRTTPTNSAVDDSAICGRDDERKNLKEYLLSEDAVTDGGSKIGVLAIVGMGGLGKTTLAKLLYNDAQVKEKFDVKAWASVSKDFDVFKLAKSLLESITSAATNLDNFDALRAELQKNLSGKRFLLVLDDIWNAGYVDWTNLMHIFNVGQMGSKIIVTTRHQDVVDIVKAMRTCRLEPLANEDCWSLLSKHAFGAHKCNELSSNLEEIGRKIAEKCGGLPLAAVALGGLLGTKLSSEDWTKVLNSNIWHLTVKDVQPALLLSYHFLPASLKQCFAYCAIFSKNSKLQKEALVELWMAQGFVNVSQNEKSIEEEGGEYFDELVARSLIRRSVDGQHFEMHDLINDLATMVSSPYCKRHDNEMQLGNLNKIRHLSYDKSMFNHFGELDSLHGLKCLRTLTALPFEFEFWIRGHYLANGVLHELLVALKQLRVLSLSDYRNITVLPNSIGDLKHLRYLDLSCTGIERLPPAICKLYNLQTLLLSDCRGLTELPEGMGKLVNLRRLDIDGTNLQEMPVEIAKLEYLQSLTRFIVSKQQHGLKLAEMRKFPNLQGKLCISKLENVFDPSDACQANLKEKNQIEEILLEWSDSILEDSQQVVLEHLQPSTSLKKLSVKYYGGSTFPSWLGDSSFVNIVSLRIEDCHHCSSLPPLGQLQSLKELFISGTRSVKSVGSEFYGGNSPSFQPFPSLETLSFEWMEEWEEWNMIDGITTEFPCLSKLSLRRCPKLKGNLPSNLPCLVTLDVEDCCLLESEFSGEVDNRNIMRPLNLFNFNSLQQLSFFGIPSLMSFPSNGLPKTLKTLSIRFCENLEFPSHEFLHSCKELEELKIWFSCWSLTLFPLGSLPVLKRLELSGCKKLKSISILEEAAASQSLMFLERLSIHRCPELESISLPDLCTPNLSSFWVRRCDKINSLPEPINNLTGLQTLWIRDVPNLESIAEEGLPINLRTLGVGNEGVYSNTDITKWGLDRLTSLSKLSIKGDYLVKKLMEIQLPLLPNSLVMMEIQGARGIQDLDGKWLQHLTSLKTLELVACDKLKSLPREGLPSSISRLQMSGCPMLKASYERKKGKEWPNIAHIPLIIFD

>arahy.Tifrunner.gnm2.ann1.E2W0NT.1

MAAKLQGRAYLSSFVDAVLNKLSSLDVNSTPDARKLADQKLLQKLRKSLRATRPVLDDAEQKQIRDQEVKKWLVDLQDALYLADDLLDELSTKAATATPTQRDPGNFSSQCHSAVNSMVEYSDDDEMEVVDNMQDIVDKLESIVEEKDVLGLKQEIAEDLEDISWRIQSSLVESSDIYGRDSDKEAIVEWLLDNTCNDKLSVISIEGIGGIGKTTLAQWVYNDARMKEKFAIKAWVCVATKFDPVNVTKAIIEDITSSPCNMVNLNSLQTELKEKVNLNPIQTELKEKLTEKTFLVVLDDVWDNQQNLWDNFLKPFLSGNKGSKILLTTRNKNVDSVVSSTNLHYKLDTLSDKDCWSLFLKHSSISTSSRQYVILEQIGKKIVEKCKGLPLAVKTLGGLLRSKDKVEDWENILKSEIWELPEDESKIVAALRVSYHYLPSNLKRCFVYCSLYPQDYQFDKDELILLWMAQDFVQPIENYTLENIGRAYFDELVERSFFQPSSKNVSFFVMHDLVHDLATYFAGKFYFRVTEFGDPKKVCSKTRHLSYMVTRYDSILRLGEAYKRAIHMRTFLDVHFSHQPYGSIKLESDFWLFQLHMRCLRVLSFKSFSIESLPDSIGELIHLHYLDLSYTPILTLPESLCKVYNLQTLKLRYCDKLEMLPSRIQELVNLRHLDIRGTYCLKEMPKGMSKLKHLNFLSGYIVGKHEENGTRELGALDNLHGSLCISKLENVNNSREALEAKIGNKNHINALELKWLPKGDIVDVETARDILDKLQPHQNLKGLSIVGFRGEIFPDWLGLPCYSNLTELSLKDCKNCGQVPSLGQLPSLQHLMIWGLDVLERIGGEFYNNSESSHEGTPFGSLQTLEFLDMPRWREWHIPDEFDGFLKLKSLLIEDCPVLSGDLPAHLPALEELTIDRCEELACSLPRAPKLHQLTVNDSVFFGNAAPHKILIKKTQLAKSILECLPHVQSPCLQRLEIQQCSSAISISGDCLPDSLQFLRIWDCSKLTFSEPLQHKSLKEVVVSKCDSLTLFPLAALPNLKTLYISDCPEMDCFGEEGLPPSLTTIAISNCQKVERWITSKGLQSEGLTYLSLQQWNEVKSFPREGCLPASLKFLMIQTLKYKRDFFAGIAESMTGTAIHSSFMETFQVLQFSFVLEWRMK

>arahy.Tifrunner.gnm2.ann1.E34FZW.1

MYDVFLNFRGEDTRFLFTDYLYHGLADVKKLQVFRDDPALELGDAIKPTLMEAIKRSRIHIVVMSENYVSSSWCLLELEQMLKYSKNNGNERSMVPVFYHVKPAEVRYQISAKYKEAMEKHEKREGKDKGEAWKSALFTVCDLSGQHIVEERDQQQLNRFDSQFGVVESLLNLESCDTVGVVGIYEDPKIGKSYIRVFAFELYYKIKYKFQAAGFLVDVSELLRKTTDDDRLEIFQKELLSDMDVKTMHELKHKKMLLVLEGVDSEEHLELLVGMGISDWFGGGSRILIATENRNLFQDCPAVMNGVKLEKHCIREGEFGKEKIVKEKKVVGFVKEFIGVINQLKEEDSNRRNVVSISGMGGSGKTTLARMIYDSNEVREVFPCREWVTVSKQCIEKEVYRNCAYH

>arahy.Tifrunner.gnm2.ann1.E5IT1X.1

MVVEPTIPIPTIPYSLRGVCKICLLAWLDLISLITWGMMDIEEESPMSGSLKAMTLRNMSSSSSAFFSANQSPFFSPRSPSLIPDAAPGPSNRVVHLDVASPSTSSIIQEPKFDVNVGCTFPDASGNSGDLQKLDRISSSVGISSSSISSSCYHRDDRYFAKKERRDKKDRCHRTSSIPDSTSCSSYRLRNCDVFIGLHGSKPPFVRFVNWLRAELELQGISCFVSDRARCRSSRKLGIVERAMNAASFGIVIITNKSFKNPYTIEELHFFSSKKNLVPIYFDLSPADCLVRDIIEKRGELWEKHGGELWLMYEGLEQEWRDAVHGLSRVDEPKLEAQDGNWRDCILRAVTLIAMRLGRRSVGENLTKWREKIEKEEFPFTRNESFIGRKKELSELEFMLFGDVSGDAEKDYIELKAKPRRKNVTIGWGKSNMLDEREKHAGNGCKKQKEPVVWKESEKEIEMQHYHSRLKRGKYARRKRGRNMLCGKGIACVSGDAGIGKTELILEFAYRFHQRYKMILWIGGEGRYIRQNYLNLRSFLEVDVGIENSLDKNRIKGFEEQEITAISRVRKELMRNIPYLVIIDNLESEKDWWDHKLAVMDLLPRFGGETHVIISTRLPCIMNLEPLKLSYLSGVEALSLMLGIGKDYPVTEVDALRTIEEKLGRLTLGLAIVSAILSELPITPTRLLETINRMPLKEMSWSGKEDRWWRKNTFLVQLFEVCFSIFDHADGPRSLATRMVLASGWFAPGAIPVSLLAVAANKIPEKCQGRCLFRKLLQALTCGFTSSYIKKSELEASSLLLRFSIARSSTKEGYIQFNELIKVYARKRDVPGAAQAMIQAVIIQGSIAQTLDHLWAACFLLFGFGRDPVVVELKVSELLYLVKRMVLPLAIHTFITYSRCTAALELLRLCTNALEAADQAFVTPVDKCLQKSLCWRSVQTNAQLNPSLWEELALCRATVLETRAKLMLRGAQFDIGEDLIKKAVFIRTSICGEHHPDTISARETLRKLPRLISNVQVHASTT

>arahy.Tifrunner.gnm2.ann1.E8NTQN.1

MADDGAAGVASSSLPAPWLWATSSLSSEGGMYDVFLNFRGEDTRFLFTDYLYHGLADVKKLQVFRDDPGLELGDAIKPTLMEAIKRSRIHIVVMSENYVSSSWCLLELEQMLKYSNNGTKRPVFPVFYRVKPAEVRYQISTKSKEAMKKHEARENKEKADAWKLALSTVCGLSGEHIVEKGDHYETAVVGKIAEQVSAKLLEIRQQLNRFDSQFGVVESLLNLESCDTVGVVGIYEDPKIGKSYITTFAFELYYKIKYKFQTAAFLVDVSTLLRKTTDDNRVEIFRQELLSDMVGKKMHELKHEKMLLVLEGVDSKEHLELLVGMRMGIGDWFGGGSRIMIATENRNLFEDCPAVMNGVKVEKHCIREGEFGKEKIVKEKKVVGFVKEFIDVINQLKEEDPKGRNVVSIVGMGGSGKTTLAKKIYDSNEVSEVFSFRAWVTVSKDYTEKEIFSTLFRSLKPSKPIPEEEGKLKKEVRSCFTKLNEESTKYLVVLDDVWDTQLCHNLKDYLLPNNNNGSRILVTTRNHEVANRARSKEPHSKLLRLNEEESWRLFCNEVFCGEDCPPDLEPIGKSIVESCKGLPLAIITIAGVVAKKEEEEWGDIEKLIPHWDVDDDIIRMKNILKTSYDDLHENLKPCFLYLGVFPEDYNINVTELIRLWMAEGFIQVRETGTSKAPPQPEDVGRKYLKDLVDRNLVQVTKRKSDGKGVKTCQIHDLFRDLCIEQSNAQSIRRLSFTRKGKYYACSVICNQSSICSLFVYGIDVDWSSDTPENLQSINVLHFETVGGKDPRVNFKELIHLKYLRIYKPVVPIRIGEYKELMHLNCRGGVKLQADREGPNNILQNLQSLSYVRPDSVLESLIKNGCFPNLNTLGLLLYSEDKQGGRTEVLKKLGDLRNLRNLKLHFDHMMKYYNTPALLMDEMEFLSNLTKITLEGAWGFNSSDMAALGRIANLKTLKLNGGLVSPNRLISCGDAGSFPKLEMFHMIDMYFLQCLALEEGVMPCLQRVVISHCGDLREIPERLLSLSNLRHLHDDIWEEADNASNKYELRRTSTSS

>arahy.Tifrunner.gnm2.ann1.E8QTMY.1

MAEDLLRPPKRGESLEEVGHECFVELAFRLFFKQDGDGYVYKMHDLLHDLAIFLAGDFYCRFEKVADAEYMSSHIRHLSCESLNHLNLDSFSKIKSLRTLLQINFSSLSDNIDAVTCILMLKLKYLRVLSFKMLDVLPDSIGGLIYLRYLDLSWTNIRTLPESLCDLCNLQTLKLHNCTSLIMLPNGMHKLVNLRHLDIWGTCLKEMPRKMSKLKHLHIFSYFTVGKHKDNGIQELGELSNLHGSFGIKKLENIVDVKEAKSAKIMDKMHIDCLSLEWFSSDDMVLDTQIERDILDSLRPHNGLKELTIEGYKGTIFPNWVGHCSYQNMTSVSLKSCNNCCMLPSLGQLPSLKSLHIEGFGQLKYISDEFYKNEDNPSFHIAHFPSLEKLEFRDMPCWEMWHLPDSETFPQLKRLLIRDCPMLKGDVFSQAFLKILSSLSDVSKVRKLDISERCSQEMSLTGDNLSIGGCESIVKSAFEAISINHLTCLQEIQISGCLSAVSLPGNCLPESLQKLAICSSGKLEFPEQHQQKYDLVELQIHSSCDSLTSLSLDAFPNLKNLEIHWCENVESVSLSQPPHTALQHLSIIGCPKFVSFPGEGLAAPNLTSLHVKRCSKLEELPRDMNTLLPNLESLEMQHCQEICMFPEGGLPPNLKSLTVGGCKQQLRSVSSMGGNFESLTHLEISGVGCESVKSFHEIGSLPHLPSITTLVIRGFSNLETLECNELLGLTSLLQLRIGGCPNLENMVGEKLPPSVLTFQISHCGLLGEHCKNKHQQIWPKISHIPTIQVNGRQVL

>arahy.Tifrunner.gnm2.ann1.EFNA3N.1

MAAELVGGAFLSSFLNVLFDRLSDPDFINMMRGKKVDQKLLQRLKTILNVVEAVLNDAEKKQITDPAVKRWLEDLQDAVYDADDLLDEVATKAATQKDPPGNLLSRFLNSQDREMVTRIEEIIARLEDIAKHKDILRLEKIAAKNMSGRIPSTSLVKKSDIFVGRHKERDTIVKLLLDDANNGELSVIPIVGMGGIGKTTLAKLVYNDDIVQQKFHVKAWVCVGEEEFDVLKVTKAVIEKTCSACYSNDLDTAQNHLKNGLVGKNFLVVLDDVWSSNRECWESFLTPSECGSEGGKILVTTRLDTVASVVKTKHNEAHNLNLLNEEQCWSVFVNRAWDPLESRDRSTLEEIGRKIVKKCKGLPLAAQTLGGLLRGKDNEKDWIDVLKSEFWELSEEDSGILPALRISYYHLPSYLKRCFVYCCLYPKDFEFYRDELTLLWMAEGLLLQPKSGITLEEIGYEYFDDLVSRSFFQHSNTYVNSFVMHDLMHDLATFYGGKFFSRILEFKNAAKHDAKTRHFSCACKNEDDLLMKITEACDKLKHARTLLQIFNKYGVFSAGDRVAVSCDLLEQLKCLRVLSFNFFSDDENLMHGSIAKLIHLRYLDLSYTSIVTLPESLSCLYNLQTLKLRSCVKLKKLPSNMQNLVNLRHLDSGGTDLEEMPKKMSKLKDLQFLSDYIVGKHEENGIGELGGLTHLHGSLWIPKLENVKNGGEASSARMDEKIHLNALYLSWSSFEESEVCDSQSEKDVLEKLRPHKDLKELFIWRYRGTMFPDWVGHSSYHKMTWLELWGCRNCWVVPSLGQLPSLERLVIAGFNKVKKIGGSFYKGDGTHQQHQETLFRSLKYLSIKGMPCLEEWESYECDDDDDDAPFPKLEYLSIWDCPKLRGDLPTFLPSLKSLHIDECEELGCYLPRAPILRELRIYGKQEARMRDLPLSMLERVWVNGEQQVEYVIEAMTHTQLTSLIELQISECSSAISFPGDSLPPLLKELRIYNCKNETVSMRIQHLKPGQRIPSNASLQDVSRVKYLHACIGATLNIFSYEISVDT

>arahy.Tifrunner.gnm2.ann1.EFT9AB.1

MALPLASSSSFSPLTTRYDVFISFRGEDTRNSFTSHLHSALLRNQIETFIDYRIPKGGVVWNELVEAFRDSKLFVIIFSENYASSSWCLRELVEIMECKKKNEQVIVIPVFYKIEPTHVRKQSGSYRRAFDEHERSSNRKHVHQWRTALTEASNLSGFTCDHHRQEAKLIDEIVEAIFPNLNNNGFRDDLKSPFICNRNYTSVKSLLKFKSENVLVIGIWGMAGIGKTTITTTLFHECFSEYEGQCFLTFSKKLEGKGLNHICAKLLSQLLNQDLRIDNIRVIHSSIIRKIKHRKVFVVLDDVTNSQIAVDLIQLFCNCLSSDSRIILTTRDRNVLTSGGVEEIHEVTEMNFEDSLKVFSHNAFSGSHPKEDCYELSTRVVADYAKGTPLALKILGSFLRTKEKSEWDSALRKLRKYPNPDIQQMLKLSYDALDDDEKNILLDIACFFHEHEMEMVTRILNSCGFFADIGIKGLSDKSLVSIYSNGGCKYIKMHSLIQEMCWKIIHKEYSKNGGQQIRLWNTEEVCNIFQDERVRESCFNGLVFKKNDVHGVESMIVDMNEITIDPRIIIIALRKMPKLRLLALRGNINIDLERNRVLLEDFQLSNELRYIEWNKCPLNFVPSICWPQKLVQLSMQGSNIEELWDTAQNLPNLKIIDLYGCKGLIKCPNLAGATNLKEISLWGCESLQDNVDPSIFSLPKIEEVYVSCCTSLKRLCRDYCSLSLRRLWATGCSNLEEFSIPIMRDHSKINLHLSLTALNEVPSTIMHLKNLQCFAFNISYSLQKLPLNLAR

>arahy.Tifrunner.gnm2.ann1.EH4SXH.1

MSSKNDSTLLSASSSDKSGNKSKKSSLRRKFRALLGFPKPSDSSPLGHPHLASTSANVFETAALSSRPVFKYDVFLSFRGTDTRNTFVDHLYNNLIRKGIFTFMDDKRLHKGEPISSQLIQAIRDSRVSIVVFSKDYAGSAWCLEEMATIAECKKELGQKVFPIFYDVDPSHVRNQSGAYEFAFLLALKYNVDRVARWKSAMTSLANSVGWDVRNKPEYTEIKEIIQKVVKTLNHRFSGFDDDLVGIQPRVERLEALLKLSSKDDNPRVIGICGMGGIGKTTHAKVLYDKISYQFDGCCFIENVSQSYRNGGAVIIQKQILNQALDEQNLEMFGHFEISGILRDRLSCIKVLIVLDNVDELQQLEELAISPKLLGRGSRIIIVTRNEHILKVYGADEVDKVALLDDDEALKLLLRKALGDDGSSSSKYMHLIPKILEYAQNLPLAVKIVGSLLHSRDVTQWTDALKRMRKIPNKNIMDVLQISFEGLELYEKEIFLHIACFFHGEREDYVKQILDSCGLHPHIGIPTIMEKSLITIRNQEIHMHEMLQELGKKIVREKSPEEPGSWSRLWLYHDFYRVLTSATGSYSVKAIVLDQKEDVSKYSQLRIEGLSKMMGLKLLILHHKNFSGSLYFLSDNLQYISWHGYPFSSLPVNFDPHSLVELNLRDSNIKLLWESPKSFPCLKKMDLSNSKDLVKTPNFEWIPNLKWLDLSGCTNLSQVHPSIGLLTQLAYLSLRNCSNLVSIDLNTEYKLYSLKVLHLSGCTKLENTPDFTGLSNLEYLDLEKCVSLSAVHESIGALVMLKFFSLRGCINLVQLPASVNNLTCLQNLDLHDCFELMNLPLRQISMLSPYLRYLIFLDLGSSKIKVIPDAIGALKCLERLNLQGSRIQSLPETIKSLSCLAYLNLSGCHELVELHDLPFKSASSGGRYFKKVSEARNHRSGLYIFNCSRVMKCHWMLVAISWLARLVKEPGHFRCGFDIIVPFMGIEIPKWFFHHRFVGGSIIRILSIDTDVNDWLGFAFCVTFEVNKNSRANPGSSSKLPRPLYLSFESENTEECFDLPACLEMEKEHVGPISTYCWIIYISRAHCHFVKTGTCITFKACPGLQVKEWGLRMVCMEDIVLIRREFRKMDCEGYDPWFRRGIQEISDPIICLPHNCFYTTDEHENEGYNRFGPKIQLPYNWYVTDDEEKENMDAKAKEINLYNLGHSSIEEVQVVNGHSSSEEEESMKMKGKALQVAFEEESDCSYMSDISPR

>arahy.Tifrunner.gnm2.ann1.EI55Q9.1

MAHTTQIAVLASLLQQASESILEMLQTARKSNQNRGFLRSVILTELTPLFNEIKQYNYDDEHLDRQREQITALITETDDDGASLCNCSCSSWSHLWENCFSWLVRHIKNNNNNNNDDDCDYFDEALREDLNETLEKLREIIEVLKCGCVSERRGVCGVPEKRGFTVGLEESMRKLKAEVMRERDGVSVIVLTGLAGSGKTTLATSLCWDQQVKGKFRENILFITCSKTFKIKIIVERLFEHSGYRVPEFQNEEDAINRMGVLLRHIGKSSPMLLVLDDVWPGSESLVEKFKIQTSSDYKILVTSRVAYPRFGTPCIVLEPLNHEDALTLFSHFAQLEDNYFSNFQNDEDILQKVVRGCKGSPLAITVIGRSIRNQPYEFWLKMVEKLSQGRFIFDSSEELLKCLENILEILEDKPIIKECFMDLGLFPEDQRIPVTVLLDIWTELYGMDDDGIEAMTIINTLNSMNLANVLVARKNACDVENYYYNNHFIVVHDLLRELAIYENNQEATEHRHRMSIGMNEQNGEFGLGEKQRGIIAEIFSKCLRWCINKQMPQQIHARTLSIAIDETCPSYWSNMQTADVEVLIFHLRAKFFSFPMFMQKMSKLKVLIVTNYGFYPSELNNFKLLDSLPSLKRIRLERISVPSFGQLKNLRKLSLYMCQTSHAFEFDNFKFSEACPNLVELNIDYSKDMVELPNGICEIPSLKKLCVTNCHKLCSLPKEIGNLKNLEILRLNSCTDLQGLPESIGMLSNLRLLDISNCISLPNLPEEISNLCGLRKLYMTSCANCELPSLISNLENLKVTCDEETATLWEAFITMIPNLRIEVPQVDVNLNWLHTVN

>arahy.Tifrunner.gnm2.ann1.ETG8N4.1

MNDDLRGESESNLEKLCAIAQSRLLEKESEQEPSKSPLSKTTLQLRKQCQCQRRVKVIGDEVEGGFRVETIVLDAFVEGVAESMEVASASSSIGERKRTESNLIEGIIQMILRKLNRKYRSEVRSPFIYDQNYACIESVLTHSKEVRTIGIWGMGGIGKTTIATAIFQEFSPLYEGSCFLANVREESERHGLSYIFTRLLCELLQEEVYISTPKIVSSAIVRRLRRKKVFIVLDDVNTSELLENLLGVGHDYLGAGSKVIVTTRDKHVLLSRSIDHIHEVMQMNDENSLKLFSLNAFNRIHPPDENGYLELSKRTLAYAKGIPLALKVLGSFLHSKSEKEWDNALEKLKEIPNADIQKVLRLSFNDLDHSEKDIFLDIACFFKGQEKEKVTTILNKCGFYADIGIRNLLDKALISIAMNQSIQMHDLIQEMGHKIVWEESVENPGGRSRVWNADEVCDILKNDKGTDAIESIFLDLTQIKDLEISTHAFRKMLNLRLLAFASGNISFGGKRITNTSSIPTNMVLSDNLRYIQWDGCPLKSLSSSCWPTKLVELSMTYSDVEKLWDGAQNLPSLEVIDLRCSKRLKECPNLSGATNLKKIWLVGCDSLRHIHPSIFCLQKLDGLYVYHCKGLKSICSNKCSPSLRSLVAYGCPNLQEFSVPMSRDRSGINLHLRLTAVERLPPSVFHLRNLQHFSFAISDGLMDLPSNFAYQIMLSDPTKHEYNTAITLHKVLPSPIFQSVRRLIFDNCCSLTEIPENISLLSSLVHLNLYHCTNVINLPESIKYLPRLQVLSVYQCEMLQLIPILPPSLKRLRVWDCQALKTVLISESQRKDEGTFIFLNCINLDEDSYSAILRDAIVRPEVGTQSPLASKLEKQEDACIILDRDCYVNVGETCCFLPVRHNKLLDELFHEHSTEASISVELTSSSKLSGFLLYLVLSQAQLCDIEGQILYNVDDFLSFGCECYLETSCGEKLHTRSSSVIQWHWDQLYHRVNIKSDHVLLWYDEKCCEKIIETINGKRSSYSAKLAFKFFAGIANKMETVIKGCGIRWIYQNVEEESRERKSKRNREVYESVDIAFQIGQGSESHQQEPIPPTKKFKQSFLLTSASSIVEAEAAEDLSLHKTNVIGLPESIKFLPQLKVLKVCHCEMLQFIPVFPPSVECLSLESETPKQHAGTFIFLDCMNLDENSCNAILKDAIARTTCWMETILTTEWKNNERMKIIITQVPSSSNLCGFMFYLVLSEAQSCIIDELVINFVCECYLETSWGESIHVASSVVVEWGCDMTIGYQLNVMQDHVLLWYDEECSKQIMETVKGREANTEKKSHFNAKMTVKFVARLPNKEEAMVKECGIRWIYSNLEEGSSREQRSKRIIGS

>arahy.Tifrunner.gnm2.ann1.EZ6YKV.1

MGYKTLQSALENIVDLLKSSKIQTIGVAGMKGVGKTALMQNLNNHDVVAEIFDIVIFIRLSADHTDHELQCKIAKRLRVDTEVINDPEEVARIIHEELQTKKYLLILDGTADEINLSQLGIPCNDNHSKVIITAQHRQVCTLNGAERMIEVGLLSRDEAWKMFCNTVGPVIDLPDIPEIARRVCDKCSCLPLLIQKIARSFRLKKSASSWRVGLEDLEERWPDYENEGVSELYSFLTFCYDELKDENKQKCFLYASLYPANCKVYTDYLVECWAAQNFLGDVNNTRKYQKARDRGQAILEHLTDVSLLDRGKQMIYVSMNDCMQQLALHISSKHPECSSYVQTREKLGDAQESLSWEKARWVSMIDTNLKNLPTNQDCSMLLMLLLQKNPDLSTIPQLFFKKHMRSLLVLDLYGTGIRWLPSSMSKLTGLKGLYLNHCKHLRELHPAIRTLELLEFLDIRGTQISFIPSLIGYLINLRCLRVPYIRSGDQNMDQTSDLDPCAILRLTRLEELVIEVVSFEDWCSNAENVMAMLTSLEHLTNLQCSFPSSNILDRFLRRRSGRQFTSFQFFVGCPNSKRPQILESFEYRISKYIRYDNSKHENTFSISEILPQTHAVELVHFKEIKEISDLGKENLEQIRGLSLEECNEIHTLIGGNANGSRDEIGLPNLEQLLLNKLLELNCVFRGPLNPGSLSKLKVLTLKNCPFLITIFCNGAIQYLSELQKLEIHSCFRLEELIPIIEVGEDVLPKLEVLLLANLPRLRFVTPGMTLRWSSLERVNIYRCPLLKSLPFCKDKETNLRSIRGEQGWWNELKWKHKAQYQDIFLPSNELTF

>arahy.Tifrunner.gnm2.ann1.F1BSAS.1

MATKLDGGAYLTSFVEAVLDKLSSILEDDSVLEGNYSAQELVGRLEKSLYDVGPVLDDAELKQFTDKRVKKWLVDLQDALYMADDLLDELSTQAAIAATQTDPGNSSSRSRVVDSYIEDSGDMEKIVGTLESVVAKKNYHRLKECARVDMSSWRTPSTSIVVSSDIFGRDKDKEEIIKLLLDDTRHAESPVTVIPIVGMGGIGKTTLAQLVYSGVQVVEKFDTRLWVCVAENSDPVHVTRTIIAALDSRPCSMDNFDFLQSDLKKTLSGKTFLVVLDDVWHDQLDTWEDFLKPFRVGNTVSKILLTTRSEKVASVFAAPNRHYQLSLLSDEYCWSVFLKHSCIFTNSEQYATLEPIGRKIVAKCKGLPLAVKTLGGLLRNKYNVRDWENILESEIWELPEDESKIVPALRASYHYLPSHLKRCFVYCSLYPEDFQFDKDELILLWMAEGLLQPMEKNTLEDIGCAYFDELVARSFFQLSSAGVGLFVMHDLMHDLATFFAGKFLSRVNKFGNPHMADSKTRHLSYNRGRISKFPEAYNGAIYMRTFLPVDVQSNDIECDFWLKQLKCLRVLSFRGFKILSLPNSIGELIHLRYLDLSKTPIVTLPESLCRLYNLQTLKLRDCYDLEMLPSRMQDLRCHKLKHLSFLSGYIVGEQVENGIRGLGALDNLHGSLCISNLENVNHSSEALEAKMGNKKHIIILELRWLLEDDDDNDEDDCGDGGGDTVDVEKERDILEKLQPHRNLKELAIYSYRGKTFPDWLGLPCYSNMTKLSMHFCKNCRQVPSLGQLPSLQHLVIGGLDGLERIGGEFYNNAESSHQGTPFRSLETLVFGLMPRWREWRIPDDFDGFSKLKTLSIEECPVLRGDLPAHLPALEELEIWECPEMDCFGEKCLPPSLTTLRISKCEKLERWITSNGLQSEGLTHLIVEKWKEVKWFPGEGCLPASLQSLELSYFSNLETLDCNGLHHLTSLQQLTISDCPKLENITQQNLPASISNLLIKGKCPLTRKLEEMNDDPRIQCQTDPEIRYTFLLWNCPSFKLRDQTPQVPKFVLFSDWLGCISLKASSILHGLQSFRLLKEAFIILLVFYAAIHMLILRIHSDRKPIETEASKPLPKHFKKTCCASSVSKVDFKGSVAATGFGLPNKTKKK

>arahy.Tifrunner.gnm2.ann1.F56ZG1.1

MTHQGWFYHVFLSFRGEDTRKTFAGNLYSALDQRGINTFIDNEALRVGEEISPSLLKAIEESRISIIIFSKNYASSRWCLDELVKILECRKEKGQMVCAVFYSVSPADVRHQKGSYGEAFVKHEERFKHDMERVRKWRSALSEAANLSGWHFTNGYEYKFIQTIVDEVSQKLNRIPLNVARHPVGLQARVSEVYSLLEPGSDDVRMVGIYGIGGIGKTTIVKAVYNTLCDQFRYASFLANVRENTSHRSGLVKLQERLLYEILGEKATKLGNVDIGINIIKDRLCRKRVLLVIDNVDDVDQLQALAGGLDWFGPGSRIIITTRDKHLLTAHQVDLTYEVKKLNHHEALQLFSWNAFKRSEPDASYFHIANRAVAYAEGLPLALTVLGSDMCGRSIRQWESALDKYKRSPNRKVQNILRISFDGLDENEKEIFLYIACFFKGQIMEYVVKALRACDLHPAIGIAVLVDKSLITLDEKFVLSMHDLVQDMGREIVRQESPLDPANRSRLWYYEDVLQVLTEGMGSEKIQGIMLDLPEKQEVQLSDQDFRKLKNLRMLIIRNAEFSGGHVHLPRNLRLLDWKEYPSPSLPSDFLPEKIVMLELRHSHLYTLEKSFKKYAYLTSLNFSSCESLSKIPDVSGIPNLEQLILEDCTSLVDIHESVGSLDKLVYLGVERCTELKNLPSVLKLPSLGCIVLNGCSQLEKFPELLGEMENLKFIEVEETAIQELPSCIINFSSLEVLVLKCCSNLKELPINIDMLPNLQLLDISGCPQLQLFTKKLRRFSTQNCSTMPAESDKGSPNLELLPSPPCLDPISPSIHSSYGFPLLENLELSDCNLSDEDLHILSCFSNLASLDISRNHFLTLPKCFNRLCSLQELYMANCMKLQHISGIPPNLEHIDATSCTRLESQSLEFLLSQGFSKAFKFEVIAPRPKMKMPFNYQSEGGSISFWIGQKFPRIALCLIFGLGNKITGFFSCEVQLSINGQKASNRVERFLSVIGDLTWLYHQDVMDFNTYLLHEQNYVEVTCEIIDASKDAEVTVYYCGVHEYKDDEDVKTQNLMLHSSSNSSDIREGNVNDPLDNTGSDCCSLARNLRLYEIPDKQWKRHPASINAEALEGDGCMAKENGKATGIISADVVHQSKELPLLQLKQYDDVVWDPMLLECQLNSMNENPLLSHHDYHTSKSKEVGPMMVRALDSPKEMKENAFDNVNKEDLVSQKKVGLKMDNVLKEPKEEPEVPIVTRKMERELRYETKSNKLDVFPMAHNTSQFDFNDNMEEFYATLRAETFALSSLTSRNGRDDFKLAYPEISEETEKALETLKEFLSKQFHQLLSLGSFSSMKAALESLSTLSTDADVSLCLKSLLLQLSTDFDQWSCDYIDASMKLESSTAGLSNLDTLEDGLIANKNQFSEFSSIEIDLCSQLVYLEQRKKELEQQLEAIKYSISISEVTRDTALSKKRETFEEGKMLKAQRDQLRKQRPRLRSEQESAKATKANIEDEWSKIREKFDGILNIFGSKYCN

>arahy.Tifrunner.gnm2.ann1.F5G8NI.1

MVEVVPHIITTLMERMRCLDAFGKKITKIKDSIIRHLLPIKMEAERSDGPALPLIPNWLNDVMDALTDLRDLLDDHGIPADATPLPKKRNICFSSCHLASCGLLHNLQKVEKWLQPIVVYVTPMEIYKRSMLTKTVVEEGERKIAGRENENEEIINRILSLTTVEGVVSVLRIVGMKGIGKTALAELVCCDIRVTTKFSVIWISGIHGNSYVESVKKETIRELEIEGKGMKKIEDLNPEEATRRSRFLLVLDDLRSENHEELLSLRRELREVPGSSGGVILVTTQNSLGPPKLVRYTLRLDILGEENCWALFEKVIGGVSSESKTSDAQKKLIKKCRGVPAAITKLATMLKTREAISEADIKNLELEFMQEMKTMYYDELPSWHLKQCFAYLPFIFPKAGQAVKVETLVQLWMAEGFLGPFNSSSQPEELGISIIKEFCRRSILSVREDQFGSITDCGMYNPLLLDLSRFVAGEGHCYMDDRGESVMQTVRRVFLTQDFDFSNGIPEPLNKTKKHLRTILFPLPGNNDWSSRIPHDVTLSLSACDAIFRAFESLHVLGLADLGMRMLPSSIGELKLLRYLNLSHNNMDKLPASIGKLKRLQTLKLSHCHQLKKLPDEVQHLVNLKHLELEGCLHLAHMPSTLGKLTKLEKLSHFTVSNNNCKHKQLQGFAELMNLKNLRGKLEILHLERFKFEEPRHVGRAYLKDKEHLWDISLKWSHDDDNNNKDNEKSLLDRLEPHPNLQGLSIKGYRGTAFSTWLSSLKNLVSFTLNNCSMCTSLPALNGFPNLKVLRLERLDSLKYITDGTKGSPELGLGMLDYLSISDCPELTSWWRPSETAHNNAILFSSLSILDVKYCPKLSSMPLYPNLDVRLILEGSSMRTLLDTINYRSTSDPAPPLSRLIRLKINNVEEESYPPNNWLENFVSLQYLWISEKMSVVKSFRHLRSLHTMTITNCTGVDLLSEEWDGLKMLRHLTIQEVAKLKSLPSGIKHLTFLAALWIVGCPELETLTEEIGNLKSLNNLYIENCHKLRSLPKTMIQLESLKNLVIRSCPLLLPRCQEGTGDDWPQIRHIKDIYLAGTSEVFE

>arahy.Tifrunner.gnm2.ann1.F5GB60.1

MAEALVAGAFLSGFINVVFDRLISREFVNLVVGKKLNRQLVEKLKTALLAAEALVADAEQKQFGNELVRKWLDSLRDALYTADDLLDCVLIRAEIRKKVRIRLPRFLNLYNRKMATKIEDVVKRIEDLEKRKNSLGLKQIPTGSSSWRPPSTSLVKGNVFGRDGDQQALIKMLNDNNHHNLSVISIVGMGGVGKTTLAQWLYNNKDLMDGVDLKAWICVSENFDVVETTKNVIKGISSGVCSHVSFDLLQQDLKKKLSEKKFFIVLDDVWSEDADMWNSFIVPFQHGRKGSTILLTTRKENVGPTVQNYSSYSLKGLSNDSCWSIFAYNASFPESNGSSELEGIGRKIAERCDGLPLAAETLGRLLRSKHDAGEWNKILSSDIWQFSMTDSKIIPALLISYYHLPAHLKRCFVYCSLYPKDYQLDKDELILLWMAEDLLKPPRRGQTLEEVGCECFDGLVSRLFFKQVYDVEKYFVMHDLMHDLATFLAGDLYCRFGEKEEMSILTRHLSYNHSISEKTCSSSKIESLRTLLYINDVSSIGKAPATLPRDILSKNKYLRVLSFGRLDIFPDSIDKLIHLRYLNLSWSNIEVLPESLCKLYNLQTLKLEDCSRLTMLPNGMYKLVNLRHLDIRGTPLKEMPKGTGKLKQLHILSKFVVGKQEDNRMEELGGLLNLHGSLEIENLENVIDGNEARSARIIDKKNIEELLLKWYVSSGDDMVSNTHTDEQDILQGLQPNRVLKVLEIDGYKGTIFPDWMGHSLYQNMTSVSLKSCKNCCMLPSLGQLPSLKSLWIESFDELKSIGMEFYKNEGHQHSSPIAPFSSLEELIFFNMPSWEEWHLPDSEAFPQLKRLQIRECRMLKEDMVNQVLMRIVSSSSDVSKVRQLKIQENDKGWGKEMRLDGDRLSISGFKCVVEYAFKAKIIHHLTSLQEIEISECSSVVSLGGNCLPKSLQKLKILWCRQIELLQQQHKFDLVHLQIYQSCDSLPSLSLDAFPNLQNLEIVRCSNLESVSMSEPPHAALQRLSIKWCDKFVSFPEEGLATPNLTHLDVSWCPKLEALPRGMNTLLTNLESLHIVGCRNICRWPEGGLPPNLKELRVGGCEEHVRGLSWMGNLDNLTHLTISGFGCESIKSYPEVGSLPRLPSLTTLHIQDFDNLETLECNELLRLTSLQQLHISYCKKLKNMEGENLPPSLLLLKIYDCGLLGEHCKNKHQQIWPRISHIATIEVDDDQIF

>arahy.Tifrunner.gnm2.ann1.F7SKJA.1

MSGGSTSLSCSFSSFSYGWTYDVFISFRGTDTRHGFTGHLYSALSHRGIRTFIDDQDLQRGHEITPSLLKAIQESRIAILVFSANYASSSFCLDELATIINCVKSKGRLVLPVFYGVDPSDVRHQRGAYGEALATHEERFKDNLDIVHNWRNALHQAANLSGWHFKQGDGYEYKFIGKIVEVVSRNIRRGVLPVADYPVGLESKVVQVNSLLEVGSNDGVRMFGIHGIGGIGKTTLALAVYNCIADHFEGLCFLQRVRESSSKHGLVHLQNILLSELLGENKFKSTSVQQGISIIQHRLQQKKILLILDDVDRQEQLQALAGNPDWFGPGSRVIITTRDTHLLSCHGVERSYELEGLNYADAVELLSWKAFKTIDVSPSYVDVLNHAVTYAHGLPLALEVIGSNMFGKSIGEWKSAINQYERIPNKNIHEILKVSFDALEEEEKSVFLDIACCFRGYTLAEVEDILHAHHGACMKYHIGVLVEKSLIKIQELGEITLHDLIEHMGKELVRQESPKVLGKRSRLWWHEDIVQVLENNQGTSEIEIIHLNFPSTEDEEEEKVEWDGKAFKKMKNLKTLIIKNGHFSEGPRHFPNSLRVLEWWRYPSECLPSNFHPKKLSIFKLPNSCFLSLYLASLLKATKFATLKVLNFDNNKYLTQLPDISGLPNLQKLSFEHCENLISVHNSVGFLKQLKSLSAYCCGKLSSFPCINLPSLEELRLSGCSSLENFPEILEKMEKITGLHLEHTGIKALPLSFHKFSRLQRLTLSENKYCKIPSAIVMMPELVMLTFTPLENERLSIEGEETELHEAGNTRFALPGSRIPAWFEHHSKGASISFWFRNKFPAIALFLAIGLTDKDTVFVSPDVTINGKKGFPEFWTEMEQIFLFDLQMIQCDLGETLSENEWNHAEISCTASDHFLFIGEKFPVEPFAKEIGIHVFRQKSTMEDIRFTDPYKRRKLDEDSILMAYGPFSVLLTRRLLNPKPSSFSLSHLLTFTNSFSTSQDPNPKPSTLSARLSFVFDQIDAIEKERSEKNQALQRIRAWRQSKNPQSPLNDAVPPSSESAKDDKPNSADAAAAEPAVAATNLEEVKKEVELVHPWPEWIQLMETLVHQNYFDHRRKDEDKMVQDVGFDAPGDVADDSDIDFTKDFKSVHDACLNFGKDRFDILRSLSRQDIQVLVGFGCPNVDKKVVFSAKLLRKHAHLDEGDVCSSCSLRNNCDRAYLLTNKEDDARTLDVMRILLSFGFDPVNGPITNKSLLKQKSVKTVVRKLLHEVVKLSSVPIDPNLPPPIIRKPPPKVKQPPPPPKKRVGRDDVEMKKGDWLCPKCDFMNFAKNTICLQCDAKRPKRQLLPGEWECPQCNFLNYRRNVVCFHCECKRPHDEFMESQMQDTKLSSKPRFNNNKVSRPEVSNAWNFDFDDNESDGADVAAFEYADAKAIDEDFPSDNLARQGNYRGWEGDFGKNNRVQGSQDEEYANPGALKPGVGFDDFEDEDDDDIDSYELESKTHSSSTRVQPSRNHFSEVEGSSDLDDVEDTYDKKHARNRTGSRKNMRSRDPFSGSEDDELDLDTEQRAIHSNFKSSHSYSANQKRKGRGPTKKLSFGSDSDEDDVGAGGLFSDDDDDLDDVYSSRKNKGNKHDSSRPNKGTRPDSGKRSFTEYRKSGSTGGRSQNKFRDDYGGSSQHSYRNGRGSQGNDRSWKKFEDFDKSTSYGNGRGKSFGNGRGSRGSDRNSRRFEDRGRSAGQFNKYGMDEKDFGEFKNSRRVIER

>arahy.Tifrunner.gnm2.ann1.F923VL.1

MSTILTKHNTAKKNIHTTKHNLLLHLPIYMAMASLAAEASSFSTPPSPRSWTYHVFLSFRGEDTRTGFTDHLCASLERKGITTFRDDKDLERGQVISLELLRAIQESMFAVVVLSPNYASSAWCLDELQKIVECKHNLGLQIVPVFYGVEPSDVRHQKGTFAEAFRKHEHRFGEGSEKLRRWRDAFTQIASYSGWDSKHQLEARFVESISEHIHRKLIPKLPSCTKNRVGIASRLEEVINLIGIGLNDVRFVGIWGMGGIGKTTIARAVYEAIRGEFKVCCFMRNVRELSAKNGFVQLQRDLLACLNISSYFHDIEDGKTTIKSALCNKKVLLVLDDVSELNQLENLAENQDWFGQGSRIIITARDMHLLDIHGVHGTYEVKGLDQEEAYNLFCLKAFKQLEPKEGYSSLCKEVVKYTKGLPLAVEVLGSYLYRRNADFWHSTIREIMNFPHFEVLNALKISYNHLMTTEKNIFLDIACFFKGMKKDEAIHILRMCDFYVGVGSDIGSGIVTLIDKALVTLDQNNKLEMHDLLQEMGRHIVYEESPSNPGKRSRLWSKDDIHQVLTNDLGTEAIQSMVLNFGHDKFYWFRSKPFSAHWSMEAFSKTTQLRYLSLPYMELPLGLNHFPSSVRVLHWDFCPLETLPLLNQQYQAVEIKMQRSNLEQVWHGKKFLEKLKYLDLSSSRNLKQTPDISGVPILETLDLQGCDSLTEVHISLIHHKNLVHLNLSYCEMLKTLPGKLEMSSLKELIIEHCQSFENPPEFGECMRKLSRLSLSGTPIGKLPSSLGNLVGLEDLNIKGCGKLDSVPDTIHRLKSLKNLDLGSCFNLHGLPSSMSSLPLLSNLNLSGCYQSEISFSHDLFCYLPSLMHLDLSGHWFANIPISIHELSKLRSLKLNGCCRLQFLPKIPSSIRELEAYGCRSLNIFESNVLSTIFTAFKYSSGQDQENQGVVLEMLIPSTEIPSLFGHFPLKDYHSAIVPYPSVCRWIKNIKGIAVCFLFYTKFWGFDKSVKLNLSVSNGNRCIIPWRTYRMCDGYHLYILCLTNDYFGEEFQQDMVFKLLLRPEVEYGEYDSEEFEHIPCYQAKVLSTGLACINEIEDLNQSEIERQRNEGQSLFDLNKSIEIMDICE

>arahy.Tifrunner.gnm2.ann1.F9MP7L.1

MLCLDPPLFFPLPSYCRDEIEFIQNIVKDVLQKLIDHYPPNDSKSLVGISENLEKVESLLSEYVEVRMIGIYGIGGIEKTTIARLIFEKYSYTFEGSCFLENVRERSGNYGLAELRRRLYLELLQGKFWQNNTGKSTFVEDRVRKQRNFIVLDDVSSLEQLDYLVQKLQWCGAGSKIIITARDKNVLVPTVETIYEMKILDSHESFKLFSLNAFNEDYPQIGYEELSWKAVGCCKGILLALIALGSFLHSKSKTEWHSALQKLEKTSDPEIQNILRLSYDGLDDEAKQIFLDITFFLRESFCGLYAAIGMRSFLDRALIAISHNCVRMHDLIQELGWDIVCQQSSGNPENRSHLWDSNDIRDVLGNNKGTDSIESIVFDMSQIADLQLNADTFKKMPKLRFLKLYIPSKSDGRLNKLQLPVGLKPFPSKLRYLEWDAYPLPSLPLNFCPEKLVTLRIRNSKLKRLWDGVQNLVNLEEVDLTDSQKLVELPDFSKTDNLKSVHLSGCRSLRHVHPSLLSLGKLELLNLLNCAKLEMLETKMHSKSLKHLYIKSRTSLSYVSHFSSLRKLLLDGSPVETLPVSIKHLTELKTLSLKGCKML

>arahy.Tifrunner.gnm2.ann1.FDM1EJ.1

MAEKLYGGAYLSPFVDAVLDSLTSILEEDDSFLERNNLLGRLQNCLYDVRPVLDDAELKQFTDKRVKKWLVDLQDALFMADDLLDELSTKAAIAATQMDPGNSSSWSRLVDSYIEDTGDIEYIVRRLESAVARKNYLRLKETAKVDISWRIPSTCLVEPSEICGRKEDKEAILKLLLDDDDDADGDLSVIPIVGMGGIGKTTLAQLLYHDDKVKENFDFRGWVCVSEEFDVVKVTKTVIEAITSSSCNLTDLNLLQLHLKEKLSRQKFFIVLDDVLNENYDDWNSLLKPFQKGVKGSKILITTRNKNVASVVQTVSPHELSLLSDEDCWLVFSKHARLSTVSVENLTLEKIGRDIVKKCDGLPLAAQALGGILRGNSDIRDWNHLLKSEIWELSNDRINVVPALRISYYFLPSYLKQCFVYCSLYPKNYEFSKDELILLWMAENFLQPVGKKTIEEVGGEYFDELIARSFLQPHKTREKNFVIHDLVHDLAMTCAGEFYFRAEDLRNAVEVDAKARHLSHNAKGNYPLSNLLGVCDRLKHTRTFIEINLNKWIPFNMENAPCIMLSQLKYLRALSFDRFPLESVPDSIGELIHLRYLDLSRTDIVTLPESLSNLYNLQTLKLYWCTHLEMLPVGMKDLVNLRHLDIRVTQLREMPKGMSKLKNLQFLSSYVVGKHEENKMKELGALVDIQQSISIDKLENVVNSNEASMARMFDKDGIDSMMLSWSWNKDENTVDSEMERDILDKLRPHTNLKELQIRGYRGTRFPDWVGHSSYHNITKIKLYACRSCCMLPSLGQLPSLKILLISGYRSLGSVGAELYFNQNGESCLETPPFPVLETLNFESLDGWKEWRSLEFNAFPRLRELTIRRCPMLRGDLPNHLPSLQSLQIWNCEQLSCCVPRSPEMTSLCIEGGNEVRIGELPPLLRELSIAGNYHVESAVQAIMHMQLSCLTSLSIADCSSHISFPVSAIPASLQQLTILRCRKLKFQMDGHHHSLQKLSIEKSCDSVTSFSLLDSFPNLVQVDIKGCGNMECIVVSRSLPCLRSLRIIGCRNLKSVSTLWMAAPQLELLTLLGCPEIDLSPTGDGDPHRSLRSLEIRNIERVVSSAAFMNSQFHGLTHLSIHGEYGESVKSLPKEGWLPASLESLTLTSIESLETLECKGLAHLTSLQRLTISNCWNLEYIQGDKLPASLTQLCISVSPLLAKRCEKKDPQLWPKISHIPAIQVDHKWIW

>arahy.Tifrunner.gnm2.ann1.FHX76J.1

MSAERIPDPSWKYDVYLSFRGQDTRSGFTSHLYDALRRAGIHAFRDDEALGRGDQMTYILVQAIEASRLFVVVFSESYAASTWCLNELMKIMECRSTRGQTVIPVFYNVDPSDIRHQSGPFEKAFQSHRFSYENEIVHAWRAALTEAANLLGFLITGSRTRSEAEIIGEIVEHITTLLASEDLFITKHLVGVQSRTEDVIQQLHSHTPEDVVLMGIWGMGGVGKTTIAKSVYNQIHRHFDRTMFLPNIREMWENNQQVFLQEQLLNGICKAQINIQNIGSGVALLRERLRLERVFLVLDDVNKIDQLNALCGSREWFGAGSIIIITTRDRRLLRMIGVDYVYQKWITMNLLNFFCWNAFGKATPLTEFVRLAEDVVAYCGGLPLALETVGCQLFGKMKDEWENVLGGLKRFPHPDVHQVLKISFGGLNDDREKEIFLDIASFCIGMERGEVLETLNYGFGISFLEERSLITFDEKDRVRMHPLLRDMGREIRRTYDVFVSFRGKDTRPTFTSHLHTSLQNAGIAAFKDDDDVDGLQRGERISIALLKAIGLSACSVIVFSTHYADSKWCLQELENIMVCHRTKNQVVYPIFYEVHPSDVRYQKKDTDFGKAFESLISRRSVEEDKVQSWRTDLREVSSFSGMTVINSRNKSEDIKRIVEHVTRLLDKTELFVAAHPVGVEPRVQKLIEESNIQKTKDVLILGIKGMGGVGKTTIAKAIYNKIGRDFDGRSFLLNIRETWQQDNGKVLLQQQLLEDIFIATRIHIRNIDSGMQILKERLGAKRVLIVLDDVTDLYQLNALCGSGEWCGPGSVVIITTRHDDLLRVCKHTFNLEEMNGAESLELFSWHAFKQACPKEEFAKLSTDVVEYSNGLPLALEVLGSHLFNMGIKEWQSALNTLKLIPHDKVQKKLQISFDGLSKNYEKEIFLDIACFFIGMDRNDVVKILNGCGLHAEIGISALKERNLITVDNNNMLGMHDLLRDMGRAIICEQSPELEERSRLWYDEKVLELLENHEGTDLKAKGLSLKLPMTNSICLSKEAFKSMTKLKLLQLAGVQPNGEFKHVSRYLRWMSWHGFPLRHTPKDCYQSNIISIELENSKLKLLWKESRLLKQLKILNLSHSHDLIKTPDFSYLPNLEKLVLKDCTELSSVSYTIGTLQNILHINIEGCTNLCVLPRSIYKLTSLETLILSGCSKIDKLEEDVEQMESLAVLKADNTAIAQVPSALARLRNLGHISLCGYEGLARDVIPLIMFWLWTSPNNMLSSVMQKCSSNVFTYANWGPRLQIKGDVSLDADNVANFNELGMDISESKSALNSLLIQMGVENSTTEILQKTISQNNELGDYLLPYDNNNPGLLTFSGEGSFVTFQVLHINGRNLKSMMLHIVFYSTSSTFITTTEGLILENVRILNLSRDTSNVFKGDKLASFKDEDREILLSSLEPGDTVRIVVALGSGFIVKKTIVYLIYDDEPPIEENLEHFNDDEDDIVCSTGDDVVASVNEGTLGIDVIAADDDEDVTVFGVHDAVAGMNETVSGVDAMAEDKDGATVANVDDDMVVNLDRNASTSGTDIMPPITANGFGANYVVGTDMNENSSSVDDNNDDDNAAIGDKNVSDNLYVASESKSTALIPLVQVPLDANTYSELTLTMMVSEDELHEDSDLLIAELDKTLSESYFVYPSPGSLELQSLSLISIFQKMQLLLDNELEALVGDVNIKNQLLGYVAQLGQIIESSQVPKDLHSLVIEISHFYEDFLNDFPPVQEVLDNHQRLIDSKNGVQEKLKAAKAKQRHFSASISKGKERVNEMSKEINELESKLKALHEKRNRLQFNVKRCEFESININKNLETLIKENEEVVSSLKESESAFRKAELSKQSYERKLAVLKQALYGNTRH

>arahy.Tifrunner.gnm2.ann1.FV5VRM.1

MDILIAVAAKIAEYTVAPIGRQVGYLIFHKAKFKELRDRVSDLEYKRDEIKQRVEEERRNGKTTFDVVQNWLKNVDDAIGEATQLQNDPRRAKAGCSRWSFPNVVTRHQLSRKATKVAQKVVEVRGGGDFSQVAYRPKLDAVSTFTTRSRQNLESRKPIMENIMHALRDPKVSMVGVYGLGGVGKTTLVEEVYQIAKENQLCDEVVMATISKTPDIKTVQEEIADQLGLRFEEVTVAGRAPRLYERIKKEKTILLILDDIWEALDLKKVGIPSDGQHNGCKLLMTSRTLDLLRQMGVKEHFRLEVLNEEESWSLFQSEANNLDKEPDKHQIAFQLAKRCAGLPILIVTMARSLIDQNIHAWKDALSQLEKVDNEELQEITYSALELSYKRLKGNEMKAFFLLCATNGKNPSVNDLFKYGMGLGIFSSTNTMEGARNRLHNMISALKASCLLLEDDTITRVKMHDVVREVAISIAYRDYHILAKYGDGDKLREFPNMDILSNCSQIILQNCGFRQLPEKLDGHNLKFFHLSNFDLSLKIPNFFFADTKFLEVLDLTGLNLSLLPTSFLSLTNLKTLCLDQCVLENIDAVGALKNLEILSLLQSSMIKLSSEIGKLSHLRMLDLGDSGIEIIPAGVISSLIKLEELYMGNTSIKWKIDNLDNQDKNASLDELRQLSSLTSLELQIQEAWMLPRDLMFDKLERFKIVIGDVWEWADIDDVTWKTLKLKLGTNIHLEHGIKGLIRRAENLYLDEVEGISNVLYQLNRDGFPQLKHLHIQNNALIQHIIDFTERSHIPTPFPNLEKLVIQNLGKMEKICHGPLAVSFFSKLRAIKVEKCNKVKYLLSVSMVKGMPQLSELEISQCNLMEKVVFEDDDASAMNDETIQFPLLHSLTLQHLDALECFYSHQPTSSPISLFNNQVRVSASILVLMRISGLLHNSYKGWNTIECKVAFPNLDTLKLSSLNLNKIWKDNRHYFCKLRNLIVENCDGLKYLFSPAMVESFSSLIKLEISECHIMEEIIAVEEDSDNNIVTLEEVRFFKLQTIILKNMKSLKKIWHKEFSKVKTLQVKNCEKIRAIFSSSMQKVYNDLETLMVTNCASVEEIFQLSSDENYSTEQTQLKKITLKGLSKLKQIWSKDPERALNFYNLEEINVESCINLEFVFPCSVATSCSHLKELIIKWCENMKEIIAFKEEPMFLSISFEFNHLNTLVLWDLLKLKGFYARNYTLSCPSLRKLDITGCVTLNLYRTLSTSSHQKLSDDEYIISTQQHLVAEQVMPNLEHLRIDEKDAANILQTQNIGSFFDKISFLALSNYKTEGSAFPDQVLQNICSLKRLHVERSSFKKIFHDKRLTNEKNCTKLQSLILYQLPNLQHICEEGLQIDPVLELLEDLYVYGCSSLINIVPSSVTFCHLTYLEVVNCNSMINLLSPSTARSLAKLTVMKVKQCDSLEEILSKEREEITNDIAFFSLRTLELDSLPRLGRFCSQKCFLRFPLLRKVVVRKCARMKYFSEGDKVSTPKLGKVLTAENRKEFYWKGDLNDTIKHMFENKLTESKPTAQV

>arahy.Tifrunner.gnm2.ann1.FXRP5B.1

MAGAVVGGAFLSGFINIVINKFLTEDAVNKVFGKKLGSDLVERLKTALLGAEALVADAELKQFGKPLVRKWLDSLRDAVYCAEDLLDAVLLKATTQKNASSSWRFPSFFINRDRDDMVDRMEGVVSRIEDLGKQKDSLGLEKIPTGSSSWRSPTSSLVRGNVYGREDDKKALVKMLIDNSEHHLSVISIVGIGGVGKTTLAQWVYNNGDLMKGFDLKAWVCVSENFDIVETTKNVIKEISPDTQGLEHFNSLHRALKEKLSKKKFFIVLDDVWSDDGDKWSNFMTPFQQNGNKGSIVLLTTRGKNVALAVKNCRPYFLKGLSEDYCWSVFADNASFPESNGSAALEEIGRKIVKKCDGLPLAAETLGRLLRTKHDVEEWNKILMSDIWGFSVEKSKIIPALLISYFHLSPYLKRCFVYCALFPKDFKFEKDKLILLWIAEDLLPPPKRGESLEEVGCECFDELTSRLFFTKSRDFGVYFVMHDLLHDLAIFLAGDFYCNSEELGKEEEIRSQTRHLCVDLRHYSSKLYNSISKVESLRTLILFGNFSSPNCNIEAATCEILSKCKYLRALSFSKLNVVHDSIGELIHLRYLDLSCTNIKTLPESLCKLCNLQTLKLNHCSKLTTLPSGLHNLVSLRHLDIRGTSLEEMPGKMSKLNQLHVLSFFIVGKHEENGIQELGGLVNLHGSFEIKKLENIVDVNEAKSAKMMDKKHIDELCLEWSPSNDLVSSTQKERDMLDNLQPHNGLKDLRIKGYKGTIFPDWLGNCSFNNMTSVSLECCKNCCMLPSLGQLPSLKSLRIKSFDQLRSIGEEFYKKEGDHHSSHIAPFPTLESLEFDNMPCWEVWHLSESETFPQLRKFEITNCPVLKEDMLNQVFFRERHTKAMYLDGDTLSIRGSESVKESAFKAMISINHLSCIQEISIQRCRKLEFPQLQQQKYDLVELTIYSSCDSLTSLSLDVFPKLKNLGISGCRNLKSVSMSEAPHTAIQHLSITECPELVSFAGEGLAAPNLTSLHVSSCSKLEALPLDMNSVLPSLLFLEIYGCPNICRLPQGGLPPNLEWLDLGIYAKQYSCASWMPGNFESLTHLTIKGDGCKRVKSFPEARELSLMCRDRLALNVLLILVFFVKQGGTKDKIWWTQECEQREQKLRGVANFATATCSGNLIGINLSGHKQLSKRRKVERRCYFHPCRAATYFHQVNDRMGKHCDIELKF

>arahy.Tifrunner.gnm2.ann1.FZ3B5X.1

MMRILQSIVESSTGVNPNLSTLEAMQNKVQQVLLDKRCLLVLDDVWDNIKWEDLKSVLNSRGSETKGVSILVTTRDQIVASAMETCPTHHLQPLPKDENWSLFTHYAFGPNKEQPAKLVEIGKEIVRRCVGNPLASKVVGSLLRNKKEEKECLNVLESKFWDIDGVMGALRLSYFHLKPSSRQCFCFCALYPEDFRISKEQLIHLWMANGLIKSKGHLEVEDVGNQQWEELLQRSFFQEVSIDKYGNTTFKIHDLFLDLAHSIVGEEYKAYDESASLTNLSRRVHHVSYSGLPELNQNTLKNIESLRTFIDLDPAISNTLFGYPALVLCKVQLCNSLRALRTRSSQLSALKSLTHLRYMNIYNSYITKLPKCVSRMQKLQILKLEQCYYLTCLSKHILKLKDLRHLLIEGCRSLVEMPPKMRELKQLKTLNIFIVDSKAKHDLAELHDLQLGGRLHIKGLKNVQSEHDAREANLMSKKELSYLYLSWNSDSNSNSLCISSERVLEALEPPPNLKNFGINGYRGSQFPGWVRNTGIFSSLVNVILFDCNNCEQIPPLGKLPHLESLYVSGMKDVKYIDEDSYDGVEEKVAFKSLKELTLIELPKLERIVRDEGLWVIVDLWSRKIFLSLKNV

>arahy.Tifrunner.gnm2.ann1.G02YMJ.1

MKRDKRIVIPVFYNVVPSDVRHQNNSFKEAFDKHQHRLKGNMMKVQSWRFALKEASNLSGFHYPSKYQDESKFIEEIVNDISEKLSYIFSIESKGLVGIDDSFTCIESLLEIESSEVRIIGIWGMGGIGKTTIAEFFFDKYSSHYEGSCMLKNVREESQKFGVLHLCEKLISELLAGESLVLKGSSKARSAFIQRKLSRKKVFIVLDDMDTFEQFKHLATQWLGPGSRIIVTTRDKHVLGKVHGIYELQGLSFENSLKLFCLNAFDKVYPEAGYEEVSKMAVNYAKGVPLALKVVGSFLYSKTIEEWESALEKLKIYPNIDIFNVLKLSFDGLDELEREIFLDIVFFFKGKNKHVVVPFLESCGFFPATGIGNLSRKALITILKDGTIEMHDLIEQMGREIVRRESIKDPGRRSRLSDPEDVYNVLNNNKGTDSVEGIMLDMSQMKRDLQLDADTFKRMPNIRFLKFYDSWWRHKESANVYVSSTLDSFPNELRYLEWSGCPVKSLPPSFCAEKLVKLSMPDSQVSKLWDGVQDLVNLKRMDLMRCEQLVELPDFTRASNLEVVRLDDCVRLRQLHPSILSIHKPEVLVVDNCKELKSLKGKVHFKSLKTLRVSGCSSLKEFSVSAEELTRLDFNGSIIDTLHASVGRLSKLVELDLSNVRLETLPNELCLLVSLMELNLQGCKQLIELPDNMKALSRLQNLNLTDCCSLRSIPELPSSITHLSATNCTSLEKLFNAETVFSLNLKSISLENCERLVDHSFLEYVHHTIMGVAIRELYDGMLHFKYLYRHLDPDSRPESPRQVFYPGRKVPSWIKYQTRESAVTVDVVADHPHYLDTLVGFILCCVVDHIPSQRGFPPIGPVGFAKWPRVIRCHSDFGYSSQFAKTSRWSSDHVCIWFTDLGQWFTELRNYNNLDGKNVTFEFKAESPEFEPLLCGWEILGCGVCPKSLILISEGREITKFSSIAFCEERIEMHDLIEQMGREIVRRESIKDPGRRSRLSDPEDVYNVLNNNKGTDSVEGIMLDMSQIKRHLQLDANTFKRMPNIRFLKFCNSWRHKISASTLELFPNELRYLGWGGCPVKSLPPTFCAEKLVELYMPDSQVTKLWDGVQDLVNLKRINLRGCKQLVELPDFTQASNLEKVYLDDCARLRQLHPSILSIHKLETLSVHNCKKLKSLKGKVHLKSLKTLHVRYCSSLKEFSVSSEELTSLNFNGTIIDTLHSSVSHLSKLVEFDLSNLRLETLPNELCLLVSLEELNLEGCKQLIELPHNMKALSRLQNLNLEDCCSLRSIPELPPSIIHLSATNCTSLEKLLNAETVFSLNLMSISFENCERLDKHSFLEFVHHTMMGVAIRDLLEQMLQYRFIGYDEGWDPDSHPESNGQVFYPGSKVPSWFTYQTRECSVTVDLLADQPYRTLVGFILCCVVYHIPSQRRVRPIGPVRSAKWPPVIRCLSDLGYTKQFAKTSRWSSDHVCIWFAEVKRYNILDGKSKNVTFEFKAESFMKYYEEGRPRYEK

>arahy.Tifrunner.gnm2.ann1.G06RN1.2

MSSQNQEYDAYISSGLQFLHPFISGLYDALKRVGLHVLADRTEPKTNKILLSGAIERCRVSIIVFTTAYAESTLKLQDLVKIMECHRRKDQKVVPVFYCLDPSQVCNLSGYFGEILSRTLQGILRDENRMLSYETALRQAASILPRFLSDIWKNRNVESHIVGRVTSLIDSTKLFIAKHPVGVDSRVKDLIQLLNNQKADGVLIIAIWGMAGIGKTTIAKALYNQISHNFEVRTFVPDIQDSVADYTRRRLHFILRDPDETQAHNFDSTRKLWWEGLRCIKVLLILDNVRSDLELGFLAVTREIFGPESIIIITTRTKHDRLQEIGVNHIYRVKEMDYNECVELFSWSAFNKATPERSFSGLINYAIEYSDGLPLALVAVGSAVSEKSIEEWENVLDSFKRFPFQDVWQVLKENIDSVGSEEKKIFLELAYLSHLFIGVDRNDICQILQGAGHHDASRAIKGIEEHSLVWFDKDKLCMNRLLQDIGREIMYMKESSIEPQQRPYDVFLSFRGKETRSKFISHLYVSLENAGIYVFKDENRLARGENLSISLLKAIGESKTSIIILSPNYAFSRWCLQELEDIMICCRNKTQKVLPVFYHIDPSEVRNQTGKFGQAFDNLMKRYPDKIKGKEQSWRKALREVGCIAGFVIRKSRNESEDIKNIVEHITHMLDMKELFVANHPVGVESRVEEVIELLKDQQQENPLLLGIWGMGGSGKTTISKAIYNKLFREFEGRCFLLNIREVWDQDNGILYLQQQLLSSIFKTTKIKVENIESGKSILEKRLGQKKILLVLDDVDKLEQLNSLAASRKWFCPGSIIIITTRDEHLLRCLRIDKLYSMKELNDKESIELFSWHAFKEPCPKEEFDSLANEVISYCERLPLALEVIGSHLFNRKVYEWRSVLNKLKTIPNNDVQKKLKISFDGLSDDRDREIFLDVAFFFIGMDKNDVIHIINGCGHSAEIGINVLMERCLITVDTKGKLGMHGLLRDMGREIIRESLPMKPEERSRLWNPDEVLNVLSKDMGTKGIEGLALNLPKSLNPTQLKTEAFKEMKRLRLLQFANVQLVGDFKYLSTDLRWLCWHECPPEYTTAKFYQGNLVAIDFKYSKLDLVWKKGQMMKNLKILNLSHSQHLTQTPDFSNMPNLEKLILKYCLKLTSVSHTIEHLKQVLLINLKGCSGLRLLPSSIYKLKSLNFHSIRMFID

>arahy.Tifrunner.gnm2.ann1.G21QXD.1

MLRNQNTKDPLLLGIWGMGGIGKTTIAKAVYNKICREFEGRCFLLNIREVWDQDNGGVHLQEQLLSSIYQTMKLKIQNVESGKVILEERLGQKKILFVLDDVDKLEQLNALAANRKWFCPGSVIIITTRDQRLLNWLGVDKVYRLEKLSDRESVELFSWHAFKEPFPIEEFVGFTCKVVSYCGRLPLALEVLGSHLFKRGIQEWISVLEKLKNIPNKQIQKKLKISFDGLNDDEDREIFLDIAFFFIGMDKDEVIDILNGCGYSTEIGISVLRERSLVTIDSKNKLRMHYLLRDMGREIIRENLPLPEERSRLWNPEEVLDVLSKDMGTKAIQGLALKLSRMKPIHLKTNAFRKMKRLRLLQLGGVQLDGDFKYLSTDLRWLSWHGCSSEYTTIGFDQGNLVAIDFKYSNLQLVWKKRQMLMKLEILNLSHSQYLIETPDFSNIPNLKKLILKYCSRLSLFSDTIGDLKKILLVNLKGCTSLRILPRSIYKLKSLKTLILSGCSEINKLEEDLEQMESLTTLMANKTAITQVPNALIRLKSIVYVSLCGFEGLSREVFPSIIWSWTSPTNNLSPQMQTYVNVSCLVSLTVPNNSFHGTSSIIGELPNIQGIRLESGSQIQTTGDFTLDAINIKNCKELEAQSAISHVSNMDTSALVDFHSQGDMSRSKATVSFVLIQMGMKCPVTKILRESISQKLADSTSEVGECLLPGDNNTDWLSFNGEGSSVIFEVPKVKGHNLKAVTLCIVYSSPDIMTYEGLILKNLLIINHTKTTPYLYEGDTLLSLRDEDWQSVISNLEAGDKVQVVVVLGDGIIAKNTAVYLIHDESIDQNIDECQEDVIVNSMVVGKKEDASNVDDMRDKNFSVPAVDATPAISNKISVSSTDKKVSVMDLGKPLAESPWAPCLIALLRVQHGSCIMDFRYESNNK

>arahy.Tifrunner.gnm2.ann1.G3725Q.1

MAGAVVGGAFLSGFINIVINKFLTEDAVNKVFGKKLGSDLVQRLKTALLGAEALVADAEMKQFGNLNVRKWLDSLRDAVYCAEDLLDAVLLKATTQKNARSSWWSPSFFINQDRDDMVDKIEGVVTRIEDLGKQKDFLGLEKIPTGSSSWRTPTSSLARGNVYGREGDQKALVQMLNDNNEHDLSVIAIVGIGGVGKTTLAQWLYNNEEFMKGFDLEAWVCVSEKFEVVETTRNVIKQLHGGTCSLDDFNSLHNALKEELSNKKFFIVLDDVWSDDADKWSNFMTPFQYGNKGSIVLLTTREENVASAVQNCRPYFLEKLSEDYCWSVFAENASFPESNGRAALEEIGKKIVKKCDGLPLAAETLGRLLRTKHDVEEWNKILMSHIWEFSVEKSKIIPALLISYFHLSPYLKRCFVYCALFPKDYEFKKDELILLWKAEELLPPPKRGESLEEVGCECFDELTSRLFFTKIKDGDDYFVMHDLLHDLAIFLAGDFYCNSDELGEEEEIRIQTRHLRVDLYRCSSKLYNSISKVKSLRTLLLFGGSSYSNCNSEAATCEILSKCKYLRVLSFHTLDVVPNSIGELIHLRYLDLSWTNIKTLPESLCSLCNLQTLKLRGCYELTMLPSGLHNLVSLRHLDIRETALEEMPRKMSKLNQLHVLSSFVVGKHEDNGIQELGGLVNLHGSVEIKKLENIVDVKEATSAKIMDKKHIDALCLEWSSGDDLVSSTQKERDMLDNLQPQNGLKELRIWGYKGTIFPDWLGNCSYENMTRVSLKFCKNCCMLPSLGQLPSLKYLRILGLDKLRSIGEEFYKNEGDHHSSHIAPFPSLETLEFDSMACWEVWHVSESETFPQLRNLEITNCPMLKEEMRNQVFFRIISSLSDVSKVCTLRIGDHYIKCQTETMFVDEDTLRIKGSESVMESALKALISINHLRCLQEIHILNCRKLEFPQLPQHKYDLVDLQIHNSCDSLTSLSLDVFPNLKNLDIHHCRNLESVSISEAPHAALQCLKIYRCFKLVSFAGEGLAAPNLKSLEVGICEQQTRDLSWMPNLHALTHLIIYGFECENIKSYPEVGSLPHLPSLTTLHIKLFDNLETLECNELLRLTSLQQLHIHWCPKLENMEGEKLPPSLLLLTMVNCGLLGEHCENKHQLIWPKISHIRTIEVDSKQIV

>arahy.Tifrunner.gnm2.ann1.G9PN1T.1

MVDEMEKVVTKIEFLEQQKDFLGLQKTTNILSSSWRESTSLVEGNIYGREDDQQALLKVINDSSESELSVIPIVGMGGVGKTTLAKWVYNTTEGFDFKAWVCISETFDVVEIIRKTIEEITKTTCSLGSLNLLQNKLLEILSGKKFFVVLDDIWSDDADNWKKFTTPFHCGGKGSTILLTTRIKEVASVVQTCSSYFLNELSQDSCWLLFAENACFPESNGNPTLEDIGRKIVNKCKGLPLAVETLGRLLQGKDDAKEWNAVLSSDIWEFPMKNSKIIPALLISYFQLPAYLKRCFVYCSLYPKDYLFEKDELILLWMAEDLLRPPTRGESLKEVGCKCFEELASRLFLKPAKNFSVRYVMHDLLHDLAIFLAGDFYCRIEEHGEQEKKKVLTRHLSHLSYRSLDNPIAEVLESIVKSESLRTSLYIDDLLSMESRASKLNYLRVLSFRKLDVLPDSIGKLIHLRYLNLSWTNVKTLPESLCNLFNLQTLILHQCRSLTLLPNDMHKLVNLQHLDLRGTLLEEMPRGIGKLKHLGTLSSFVVGKHIDNGIQELGGLSNLKGSIEIKKLENVVDVRQARSAGMLEKNHIDNLSLKWCSVDWMVSNTETERDILDSLQPHNDLKVLTIEGYKGTIFPDWLGHCSYNNMTYVSLLSCKNCCMLPSLGQLPSLKYLRIKGLDQLRSIGEEFYKNEGDHHSSHIARFPSLERLEFDNMACWEVWHIPESETFPQIRNLRIRNCPMLKEEMLNQVFLRIVSSLSDVSKVRKLHITVSSIVSHFEGISLNGDTLSIRGRKSKKESALKAMLSINHISNCFPKSLQKLKIDGCRKLEFPEQQQHKYDLVELKIQHSCDSLTSLSLDFFPNIKNLEIEGCRNLESVSKSEAPHAALQRLSITYCSKLVSFAGEGLAAPNLTHFQVTCCGNLKAFAGEGLAAPNLTHLHVTYCDKLEALPRDMKGLLPSLHSLQIFDCPNICRLAEGGLPPNLKSIKVGIGQQQMRDLSWMANLHALTHLIIEGDYCHNIKSYPEAGSLPHLPSLTTLKIRRTLECDELLRLTSLQELTISVCSKLENMAGEKLPPSLLLLYIFHCRLLEKHYKSKHQLIWPKISHIPTVEVEYYQSSGHFPVW

>arahy.Tifrunner.gnm2.ann1.GA50RN.2

MSYQSAERIPTRSWKYDVYLNFRGQDTRHGFISFLYQGFKNAGIHAFPEDEELMSGEEIAYTVLQAIEGSRVSVVVLSEGYASSRWCLDELSKIIECRATIGQIVLPVYYNINPTYVRHQRGSFEKDFAKHEERLSSSLEKVQRWRSALTQVANIGGFEINKGSNRNEAEVVGKIVERVTSLLVSEELFITKHPVGVKSRMQDVVQILDCHMRKDDNVVLIGLWGMGGVGTTTFAKTVYNKYCHMFECPKFLPNIREMWKNNQQVFLQEQLLNGKDLQGIKIKNIESGVAILKRRLCTKKALVVLDDVNNIDQLNALCGSREWFGAGSRIIITTRDRRLLCMVGVDHVHRVTEMDYNESLQLLCWNAFTQATPLEEFARLAKDVVAYCGGLPLALVTIGCQLFGKTIEEWETVLDGLKRFPHPDVHKVLKMSYDDLNDDTQKEIFLDIASFCIGMESGEVLKTLNYGFGLGKVAGIGFLEEQSLITFDDKNRVRMHPLLRDMGREIVREQSQTQAQGRMYDVFLSFRGKDTRSTFTSHLHASLQNASITVFKDDDELQRGERISISLLKAIGLSACSVIVISTHYADSRYCLQELENIMWKKIRCKVGEQIFVKNESEDIKRIVEHISRLLDKTELFIAARPVGVLSRMQKVIEKLHNQQRKDVLILGIKGMGGIGKTTIAKAIFNKLGRNFEGRSFLLNIRETWQQDNGKVSLQERLLHDVFRSTTRKIHNIDSGKQTLMESLRTKRVLIVLDDVDNLDQLNALCGSRDRFGPGSTILVTTRDDHLLRLCGVNHTFDIEKMNTDESLELFSWHAFKQAYPKDEFFKLSRDVVEYSNGLPLALEVLGSYLFDREIKEWQSALDRLKSIPNNRVQKILQISFDGLSDDNEKEIFLDIACFFIGMDRNDVVKILNGCGFHAEIGIRVLIERNLVTVDNNNMLGMHELLQEMGRAIICEKSPELEERSRIWYNETLLQILENHEGTNLKAVKGLSLKLPTTNSICLSTEAFKTMSRLKLLQLASVQLNGEFKHVSRYLRWMSWHGFPLTYTPKDCYQPNIISIELENSKLKVFWKEAQV

>arahy.Tifrunner.gnm2.ann1.GALA63.1

MSIISSFIPEFLQERVFDATTGYVTRQVGYVWDYEKRFKDVSDAVKALKNDSDGVRYKADEDEGRYGRIIYENVLEWLGRVEKIVAEYEKFKEEHDNTAGYALAFPLQNLDIRYHRSKTAEDIKERVEELQNEKHDRISRWQGPPSSMGYALPSVEYEELDSRKQNMEDVKKALEDSSATMVGVHGLAGMGKTTLVIKAINTLQSREPKLFDMVIMANVTKTPDIRKIQGQIADMLGITLQEESECLSIYILYL

>arahy.Tifrunner.gnm2.ann1.GBJR32.1

MALATPALPDWLVKWVKETLKQELSYLVYYQSNINDLENQVNKLKQERQKLGDRVAEDEDRHGKEIYDDVSKWLDRADTIISDYEKFRKEEDHAHAVCFSGLPPNLLARYLLSKKSIQLKKEAESQVQKAKFPCISHGGPPSVGLALSNVDYQSLPSRATAMEDIMNALKDSSARMIGVHGPSGVGKTTLVKEAVNRIQNDQEKPKLFDVVIIANVTKTPDIRKIQGQIADVLWMKLEEESEEGRAGRIRERLKKEKESTLIILDDLYGKVDLNILGIPWQSGDGNQKNPKGKKSLGSRVPVPEEKKTDQQVIKQGVMKEQKDPDGSSPLTTEERYKGCKVLLISEVRQVLNQMDVRPDVVVAVNLLNEKDARTLFNEMAGIGDKSHELGELPAQIVKKCDGLPMSIVTTAKALKKRSRLVWEETHHKLETQALVGTPEHSTRVIYDLLENEELKITFLLCACMDHDALVSDLVRLCIGLGFLQGIYTVKDARTRVQVMLMKLRESGLLSNSYSSDRFTMQNLVRNAALSIAFKERHMLMLTKGKIDEWPDDDELRRYIVISLRHCDVNEAITKSMTCDKLKILEINNNDPQLQLPDDFFKQMKELKVLILTGVHLSLLNSAIGCLTKLRMLCLEHCTLDSSSEELRIIENLKNLRILSFSGSNIDCLPVELGKLSKLQTLDISNCPKLRVIPPNVISRLTSLEKLYMRKTPIQWPKVINGAENDERRNASLLELGGLNQLTNLDIQIQSVDHLPENLFFDKLSSYKIVIGSLSRYLEKDFKIPEKHELSRFLAIHQKGGIHIHSQKGIKMLFERVEYLLLGKLTDVEDLFYDLNLKGFPHLKYLAIQNNHDIQFLIHPKDRQQHLEKAFEKLETLDLYKVTQIEELCFSSCLLSEPSFANLKTIKVNFCEKLKHLFSTPMLELLYALETIQASDCDSLKEIVPVESTHESKMLKLPKLRTLTLQSLPEFSGFDPISTTEGIKILFHEKVEVSELERIELKFIQIDQIWIGQSPNFGNLIHLDVIGCHNLKYLLPFSLATNLKKLQSLYLGECYKMENIFPDGPVKVAKGVSFPNLKNIKLSRMSSLRKIWNLNVPVGKLDTLVIEKCNQLVSVFSHDMEGIFQGLSSLTVTNCKSMETIFDLAADQNRYAFETVLRDVHLESLPKLETILGCKEDQEGTLKLKSLQNVTVHDCDKLENIFPFSVAEHQLKKLQCLVVSDCFKLNEIVAAQKGTSNNSSSSSNLVLLEFPELTTIKFSKLPKFQNFCPGHCELKCRKLHDVSIEFCGNLELFREETSQATTSAQGKPLFHEKVMNELRSMHIELQHTSSSSRYRRDKLEVLRLSWLEDTNILYRFLHSNPYLKSLWLDNCYFSRLVEPKSDGIENIGVVPKLKTLKLTNLPSLNDIGFEQDAILQRIESLTLENCPSLETIVPSKERVCFNFLTSLEVVDCKCMKYVMPLSTAESLGQLVTVKVANCESLVEIVSDNHQGGEEHKKESIIVFKQMKALELVSLNNLVSFCSSKSCSFEFPSLKKFVVSACPKMETFSEKEIKTTPTIMQKVYVVGDEEKRMCWDCNLQDTIKYIYDTKKYYEGMDKISVSEHLVLEKGWKNETALDRGWFYSLKTLKLERCKFESCAIPSFVLRCLKNLKELEVRNCKSIAFIFDMNDIKGTFQLEKLTLEELPNVTHVWTQQDKQKDSRFRNLQQLTVKFCRKLKVLFPMAIARNLKMLEQLEVSSCDELREIVEKDGGGGGGTENFVFPYLTKMDVHNLPQLAHFYDGKFTLECPELNYLYPFLCDKFELFHTRINRPALFSNIKDISKVEVMSMRSKDTWVLKSWLQESKNVELEYLGGLMLDFVDDVNNEYSTLPFEILTRTPKLERMGMMRSTSLKEISFRSQDPYREKDKFLGHLEYLLLGSLFELISIGGLEPLSKLEQLEVHHCPLLKRIEQYPSSLKILNVAGCHGLECLFTSSTARTLKHLEELDVSGCKSLKDVMRKEQDDETATEEIIFEQLKTISLQYLESLECFHSGNAALKLPSLARVTIWKCPKMTIFSPQLTDGEDPPRQISVSFHSRDDKSEVQLTQTRLCLNDYPEMQGKWVGASGIPVEWSFNYLKYLEVEGCDFLTNAVLPSHLLPLLSNLEELTVKKCKRVAAIFDVKDTPPEHDDPNKIVTIPVREIFLKELPTLTHVWNNDPKASLSFPSLEKVFVEECKSIKSLFPASVPRDNLQLLVVRNCGGLEEIVAKDEAFPQEVIILFPKLTCLVLRDLPKLRCTCSGMQSLLDWSAVLTRLYVSRCPMLKVFAQDIQNSNPGVEDCFATDDDKHHLVSSAQKVTTSNFEELELSKEDVTMIEKGLLHVDLQNLNYLGLNNFNDDETDEFPDVFLSKMSLPKLKEFQLLDCAFKYIFRPKRPDMDYSKMLSQLNYLEITNLHKLNSMGFEHPWMAPLLESLETLKVSECNLLTNLAASSVVSFLYLRVLRVENCAGLKYLFTSSTAKSLGALRELVITKCKSLETVVAHEEGETPDDLILFSNLYTLSLNELPQLESFYTGNSTLYFSSIYYVSFTVTKCNKMKTFSHGDVLPKFLQGEIDEDPWHGDLNNAVRSSFKKQAVSHNKDDMYVHIYEVCVVLKLE

>arahy.Tifrunner.gnm2.ann1.GG3E31.1

NIFFQTVSKSPNLKSIVQTLFEHCGHRAPEFQTDEEAINRLGNLLRHVGDKGRPILLVLDDVWSGTDSIVEKFKFSTIPGYKIVVTSRFAFRRFHTQFHLKPLGDDDAVSLFHHFTQAKDTNSYKPDENLVHEIVRGCNGSPLALKVIGGSLCHHPYEFWQTMKERLKSNKDLDSHLQNCLDIVEDDKEKECFMDLGLFPEDQRIRVPLLNDMWTELHELDEDGVKAMNIIHSLDSKNLANVIVTRKVATDVEMYYNNHFLMQHDLLRELANHQSNQEPIERRKRLAIDLTQNGKNCPNWLVGQNQPGIVSRTLSFLSVRGTQQQQKQEQVTARIMFVSTDETFTPDWYNMNLAETEVLILNIHSNKYTFPDFIQKMRKLKVLIVTNQGFHPCELNNFEVLSSLPCLKRISLEKVSVPSLCKLRKLRNLSLYMCTTKQAFGSSDIKISDALPNLVELNIDYCMDMVELPSDFCNIMTMKKLSITNCHKLSKLPPEIVMLKNLEVLRLSSCSDLKEMPESVERLQQIWCLDISECISLTQLPADIGELHSLRKLYMWGCSGLNELPHSVTSFENLKHVIHVICDDEAAALWEHFKEFTSLPNLMIVKVRADINLRWLPGFH

>arahy.Tifrunner.gnm2.ann1.GJLC5G.1

MLAKLSALFDAAGDIHFKYQLPGDDFDALISVTSDNGLNSLMLENSPKIARMRLFIFPGNGPDSASTQPFPAKLKSKVNGGAVFPLQITRVKFQDFPLVNNFNTSDWVLDLDPKVNRPVEDSSSSCLNSPLPALQYDDFISFRGEDTRASFTSHLFKALSRKQIVTYTDDLLHKGDSITFLLLRTIEESCLLLVVFSENYASSKWCLQELVKIMECKKEFGRLVIPVFYNVDPSHVRITRRRRFRSEGKLSLQQQILKAWTPVLIDRVTLLLMLCLDPLPFPLTSYCRAKIEFIQNIVKDPPNDSKSLVGISENLEKVESLLSESVEVRMIGICGIGGIGKTTLARLIFEKYSYMFEGSCFLENVRERSRNYGLTELRRRLYLELLQGKFWQNNTGKSTFVEDRVSKQRNFIVLDDVSSLEQLDYLVQKLQWCGAGSKIIITARDKNVLVPTVETIYEMKILDSHESFKLLSTNWIRGAIMKSGWLLQRHPTSLNSIGKVEWHSTLQKLEKTPDPEIQNILRLSYDGLDEEAKQIFLDIACFFKRELVEYVVNLLDSYGLYAAIGMKSLLDRALIAISHNCVRMHDLIQELGWDIVCQQSSGNPENRSHLWDSNDIQDVLGNNKNLVNLEEVDLTDSQKLVELPDFSKADNLKSVHLSGCRSLRHHLYIKSRTSLSYVSHFSSLRKLLLDGSPVETLPVSIKHLTELKTLSLKGCKMLQHLPELPSSIRHLIALDCIMLQTVTFSSNIPRLQEEKHINISFHNCMKLDVANCIYWYLKDIRKLAYVCKSRRGGKGVVRRSDFFKVCYPDYRVPEWFMHRTKGTSITFEVSSPSSYGFSSLLCVVLPKYSLDYELDIKCRCYLEDGSNMHKYSFGILFLNHIPVEECSDHVYMAYNYGGIFDVIKLDRLNNKIVSSGQNLKVTFEFFVSSGDTGSKQDDNLLIKECELNRRYYVRVGHEKQGIGMLDFVAHGTMRLELEALGLQLKLLFPSLKTKGNTVLTKPNMEPSW

>arahy.Tifrunner.gnm2.ann1.GK1TXU.1

MAEALLSGFINVVVKRLISPEFVNLVVGKKLDRKLVEKLKTAILAAEALVADAEQKQFGNDRVRKWLDSLRDALYTADDLLDRLFIKAEIRNKARIRRPHFLDLSVRKMVTKIEDVVKRIEDLEKLKHTIGLEKIQTGGSSWRPPSTSLVKGNVFGRDDDQQALMKMLNDNNHHNLSVISIVGMGGVGKTTLAQWLYNNKDLMHRVDLKAWICVSDNFDVVETTKNVIKGISLGVCSLDSFDLLQQYLKEKLSKKKFFIILDDVWSEDAHKWNSFITPFQHGRKGSTILLTTRNENVGPTVQNYSSYSLKGLSNDYCWSIFAYNASFPESNGSSELEEIGRKIVERCDGLPLAAETLGRLLRSERRAEEWNNILWSDIWEFSPANSKIVPALLLSYHHLSTHLKHCFVYCSLYPKDHKFDKDELVLLWIAEDFLQPPRRGQTLEEVGCQCFDDLVSRLFFKLVENDDEKYFVMHDLMHDVATFLAGDLYCRFSEELGEKEEMSILTRYLSYDHSIPEKTCSSSKIESLRTLLHINHESRFWKARATLPCDILSKNKYLRVLSFDRLNIFPDSTDKLIQLRYLDLSWSDIEVLPESLCNLCNLQTLKLEGCSKLTVLPNGLYNLVNLRHLDIRSIRLKEMPKRMGKLKQLHILSKFVVGKQKDNGIEELGGLLNLHGSLEIQKLENVVDANQARSARIIDKKHIQELLLEWCLDDEMVSNTHTDEQDILHGLQPHRVLKVLRIKGYKGKIFPDWLGRCSYSNMTRVSLLDCKNCCMLPSLGQLPSLKSLRIERFDELKSIGMEFYKNEGHQHSSPIAPFPSLERLVFDEMSCWEEWHLPDSEAFPQLKSLQITDCRMLKEDMVNQVLMRIVFSSSDVSKVRQLEIREYHEGWGKHMRLDGDRLSISGFECVVEYAFKSRIIHHLTSLQEIQISRCSSVASLGGNCLSKSLQKLTISNCRELEFLQQQQKYDLVELHIVNSCDSLTSLSLDSFPNLENLEIRGCSNLESVSKSEPPHAALQHLTITSCPNLTHFDVSYCDKLVALPRDMNTRLPNLHSLHIQGCPNICRLSGGGLPSNLKELRVGDCEEQVRGLSWLGNLDNLTHLTISGIRCESIKSYPEVGSLPRLPSLTTLHISKFHNLETLECNELLRLTSLQQLHIHWCPNLENMAGEKLPSSLLLLKIERCPLLGERCKKNQQIWPTISHIPAIEVID

>arahy.Tifrunner.gnm2.ann1.GP7DSL.1

MAATLVGGAFLSGFINVVFDRLLTTDAINLVLGKKLGSDLIQRLKTALLGAEALVADAEMKQFGNPLVRKWLDNLRDAVYCAEDLLDTVLIKGTTQKKESSCCSLSFFINRHRDDMVEKMEGVVRRIEDLGKQKDFLGLEKIPTGGSSWRTPTSSLVRGNVYGREDDKKALVKMLNDNNEHHLSVIAIVGIGGVGKTTLAQWLYNNQEEFMKGFDLKAWVCVSEKFEVVETTRNVIKQLHGGTCSLNDFNSLQNALKEELSNKKFFLVLDDVWSDDGDKWSNFMTPFQYGNKGSIVLLTTRGKNVALAVQNCRPYFLKGLSEDYCWSVFANNASFPESNGRAALEEIGRKIVKKCDGLPLAAETLGRLLRTKHDVEEWNKILMSDIWGFSVEKSKIIPALLISYFHLPPHLKRCFVYCALFPKDFKFDKNELILLWIAEDLLPPPKRGESLEGVGCECFDELTSRLFFTKNNDRDDYFVMHDLLHDLAIFLAGDFYCNSEELGEEEEIRIHTKHLFVYLSHRSSKLYNSISKVESLRTLLLFNNFLSPNFNVEAATCDILSKCKYLRVLSFCKRGVVTWHMRDSSFSILVVVPDSIKELVHLRYLDLSWTNIKTLPKSLCNLSNLQTLKLYHCSNLTTLPSGLHNLLKLRYLDIRGTSLEEMPGKMSKLNQLLILSYFVVGKHEDNGIQELGGLENLHGPLVIKKLENIVDVKEAESAKIMDKKHIDQLWLEWSSGEDMVSDTQREKVMLDKLEPQNGLKELKIKGYKGTTFPDWLGRCSYTNMTSVSLKSCKNCCMLPSLGQLPSLKSLRIEGFNLLRSIGEEFYKNEGDHHSSHIAPFSSLETLVFDNMACWEVWHVSESETFPKLRKLQITNCPMLKEDMLNQVFFRIVSSLSDVSKVRKLHIGDLIIFRQTEAMYLDGDTLTIKGSESVMESAFKAMTSINHLRCLQQIYIWRCRKLEFSQQQQQNYDLVELLIRHSCDSLTSLSLDVFPNLKNLEIEGCMNLESVSMSEAPHAALQRLSISECSKLVSFAVEGLAAPNLTHLQVRNCEKLEALPRDMKSLLPTLQSLQIYGCPNICRLAEGGLPPNLKELHVGIGEQQMRDLSWMGNLHALTHLKISGDYCDNIKSYPEVGSLPHLPSLTTLEISGFNNLETLECNELLRLTSLQQFRIFECKKLENMEGEKLPPSLLLLIIQDSGLLGEHCKNKHQLIWPKISHIPDIKVF

>arahy.Tifrunner.gnm2.ann1.GSP809.2

MANPASTSETMATNYDVFLSFRGEDTRHGFTGHLYDALCRNGINTFIDDENLRTGETIRPQLLQSIEASKISIVVFSTNYASSTWCLDELVQILRCHRERNQLLFPVFYKIKPSHVRHQKKTYKEAMDAHEKRFGCHSQKVKEWKEALEEASNRKGFHLKQEYEFEFIQKIVSKALTHIPPRQLLIGDHMVGMQTRVEEVESHLYSWYRVLSNGESPKPKLYNNNTMLGIVGIGGSGKTTLAKALYNSICDRFECACFLLNVRKISDQEEGLVRLQQTLLSKLLGEWEIKVRSVEEGISMIRKKLNKKRALIVLDDVDKIEQLKALAGECDWFSYETQIVITTRDKSLLEAHEVEKIYETKLLSDLESLELFCWNAFKMTRPKANYEDLSNQAIHYAQGLPLALKVIGSNLINKSLEEWKSALDKYEKNPPKDIQSVLRVSYDSLEGNEKDIFLDIACFFNGKKWEYVKNVLDGCGMFTEVGIRILADKSLVTIKDGYLRMHDLIQNMGREIVKQEAPREVSDRSRLWFHEDVLELLPDDKENKKIEGIKLVQCEEDDWTDTALEKMKKLRILILRNTNLSCRTILLPEQLRLLDWKGYPSKSIPSNLKKIVAFSLRHSPLTLENSFQNFEHLTYMNFSHCESITHFPSVSKTRSLRKLILNGCKNLVSFDESVGDLPNLTYLRASKCPKLRKFLSRICLPSLEHLSFNWCTSLGLFPDIVGKMDKPLKICLKATAIEELPDSFFDLVGLRYLDLTSCEKLGFLPSSLFMLPNLVTLKVGGCPQLGGAFARFRGSLSTTAESGPSLETLHFSHASLCDEDLHVIMQSFPNLEVLNVSSNNFVSIPACVQESSYLTSLDLSCCLKLQEIPELPSSVRKVDVRHCNSLSANTTSMLWSQVRREINKLQVVMPTSNTKIPEWWDDRTSWKYYSQDLNFEARGKFPVVALAFVFGEMNYQSVGLHLSIESGDVNSAYQPSHNFTVAENHVLLCDLRLWFSDEEWKRLDAHVEHGNTWKTVKVRCVPGIIPFHWGVYVYKKETSMKDIQFQKEEWEDVPCGAGRSNWKKSAVELAIACFSESLKTVVKKLKRLMAPREEEQSFCLMQQDEDEEEEGDSKVVSR

>arahy.Tifrunner.gnm2.ann1.GT0Q5X.1

MINVLLKTSEGKTKNGIDKEALRQIRDAAHEAEDVIDTFVVNVAMHKRRTKLGRMLHGFEHAKLLRDVAEKIDSIEATINKIRDNKIKLTDVVPQESESSTSTREEEERILLMHKKRRIVEEHGIVGFVGESKAVIQLLKEESSQSNVVSIIGMGGLGKTTLARKVYNTDEVMSYFHCRAWVYVSNDCRVMELLIGLIKSLMPDEMKRIDLFSLSVDELKLKVQDFLSMKRYLIVLDDLWNTRDWDEIQDVFPNDNNGSKILITSRLKEVASHTSPYPPYYLQFLGDVESWELFSRRVFRGEECPSDIEQLGKQMVKSCGGLPLSIVVLAGLLAKKGKSHKEWSKVEGHVNWFLTQEDKTQVKDIVLKLSYDNLPTKLKPCFLYFGIYPEDFEIRVRPLLQKWVAEGFIQQTGTRNAEDVAEDYLYELIDRSLVQASRVDFNGDVKACRIHDLLRDLCISEGKEHKIFEVCSDVNTLDRSKPRRLSIQCPMHRYASSSNDDHSCVRSLFHFGPRGYCDFTPGEQKWLFKSFKLVRVLDLGYNYFSKVPSNLGLFIHLRYLRIYIMTAISPQHIADSICKLENLQTLDIGGVPIPIVFPKGVWNLKQLRHLNTAGWVLLEDHYGSKAHDQVMLNLQTISFIAFTEQIADMLKKGRFPNLRKLGLNILPFLRDDMHEMLSSLQQLTHLNKLRLFFDRERQIGCKPIDLSQSLQHLSNLSTLKVDEAFNLATFDIAFPPCITKLTLTGISIRNDDGMNAIGNLTTLRLLRLYGTGKFDDSFEINCTANSFPQLQVFEMEKLNVDNWKLGNGAMPCLQTLLIKCCERLDDLPDELWSLTSLRQVKVVEPSQALSLAVANLEMKDGCELIITEGYQYRFRV

>arahy.Tifrunner.gnm2.ann1.GT1121.1

MARPCTYKVFLSFRGEDTRKGFTSHLYTALVNRGITTYIDDKNLRKGDLISDELLTAIEESMFAVIVLSPNYASSTWCLDELHKIVECNNNLGLQIVAVFYHVKPCDVRHQIGAFEEAFKKHELRFGKEGGRVRRWRNALTQVVSYSNWDSERFENEAILVESITQHIHERLIMKLPSSMKNLVGIDSRLEEVVRHIGLGGNDVRFIGICGMGGIGKTTIARRVYETIRSEFKVSCFLADVRERSEKRGIVQLQKQLLDCMNINSDTRFDDEFEGKGIICHSLCHKKVLLVLDDVDDGGLLENLAGEQDWFGPGSRIIITTRDTHVLEVHGAAYRICKVEGLEQDEALELFCLRVFKRSKPEEGYMDLSQEVVKYCDGLPLALVVLGSHLCGQTIDVWRSAIEKIKSFPHDKIFNTLKISYDGLDHSEKNIFLDIACFFKGREKDYATNIWKRCGYHVEASIAALINKSLLAIGEYYKETLEMHDLLEDMGKYIVIKECRDDPSKQSRLWSYKDVDLVLAQNKEIEATHSIVLYNKMEWGNNLGLQIVAVFYHVKPCNVRHQIGAFEDAFKKHELRFGQESDRNLVGIDLRLEEVVRHIGLGENDVRFIGICGMGGIASKKVRALESVQLLQAEANDRSFWGSQS

>arahy.Tifrunner.gnm2.ann1.GXWQ1S.1

MTSSSSSSSTAPQFKYDVFISFRGSDTRRGFFSHLLTALRQKKIHVYVDYNLKEGNEILPALFAAIEQSQIALVIFSKDYASSKWCLEELVKITECQKEKGQIIIPIFYNVEPSEVRHQKRSYAEAFVKHENDFKDKVNKWRAALKGVSDLSGFHSKNFRSEAGLIEEVVKHILKTLDDMHPAYDFQGLVGINKPVDELESIVFANGSKDIRVIGIWGMGGIGKTTIATVLFNKLYSQYESRHFLANVRKESQKWGIIQLRDKILSVFTDDKDLNIGVPNGILKHVLRKLRCRKILVVLDDVSTSDQIINLVGGHTWLGPGSRIIVTTRDKHAICKEADDIYKVKALESSDARQLLNLHAFDGTDGHCLELRWHNLVSKMVEYAKGIPLGLKVLGSFLYGKCFEDWGCQLKKLQEMPFPEIHNVLRLSYEGLDHQEKSIFLYVACFFKDDEGKDTVENLLDACGYSTSIALKTLQDRALISIEHCVSMHDLIREMGRWIVRQQSINKPGEQRHLWDPHDIHRVLKHREGTDHIESLTWNMSSVSADISLSPHLFARMENLKLLRFYYNNNNNNSSDYYYYYYCSDNNTCQVHLPEGLEILPQKLRLFHWDNYLAKSFPTTFNAECLVELRMKESRVEKLWDGVQDIPNMKRIVLNGSKQLIEIPDLSKALSLQELELMGCINLLSVHPSIFSLPKLVTVTLWNCRRLEKLPGWCEVKKEVLSEASASSTSSCSGSSHNSAPSFST

>arahy.Tifrunner.gnm2.ann1.GZWC57.1

MAAALVGGAFLSSFLNVLFDRLSDPEIINLIRGKKLSEKMIQRFKAILNGAEALLNDAERRQIREGPVKIWLDDLKDAIYEADDFLDEITTKAATKKDQGNCLTRFLNLKDRKRVNRMEDVIARLESIVNQKDTLGLKEIPMENMSWRTPSTSLVKVSDIYGRDEDRKALVKLLLDDANDGDVSVIPIVGMGGIGKTTLAQLVYNDEQVQQKFNVKAWVCVGEVFDVLKVTKTVVEKATSASCNMNDLDSVQQRLRTEVTGKSFLVVLDDMWTNHYDDWKTFLIPFQCGSQGGKILVTTRIDTVASMVKTIPAHNLSLLDDEHCWSVFANHAFFATDSRDRLALEKVGRKIVEKCKGLPLAAQSLGGLLRTKDNIADWEDVLTSEIWEFSEDECRILPALRISYHYLPSYLKRCFVYCSLYPKDYAFDKDELVLLWMAEDLLQQPKSGSILEEVGYKYFNDLAARSFFQPSKDGYLDSFVMHDLMHDLATFYGEKFFVRISEHENVAQQDAKTRHLSYNLMDNDSVPKMLEACESLCHVRTLFQIKVYLYGYKEGIDPCRLLAQLKRLRVLSFPSFQIDRLPDSIGELIHLRYLNLSDTLVVTLPKSLNNLYKEKHEENGIGELGELAHLHDSLCIQELENVKNSGEASSARIYEKIHLNALYLRWSSFEESEVCDSESEKDVLDKLRPHKDLKKLSIRCYRGTMFPDWVGHSSYHNMTWLELRGCRNCWVLPSLGQLPSLERLLIGELDKVKKIGGSFYKGDATHQHQQTPFRSLKFLVIEELPCWEEWESYECDDDDHAPFPKLETLIIQNCPKLRGDLPTFLPSLKSLDIYGCEEIGCYLPRAPILRELRIYGKQEARMRELPLSMLERLVINGEQQVEYVFDAMTHTQPTSLRELRISNCSSAISFPGDSLPPSLKELAIYDCKNVEFPMQHQQHESLRGLTIDNSWTFNDSSGTCQFNAVPLCTSILLLLHHHALSYTQMEARNKLKQSWSKLNLESRGTWSRMISRGVVSASIRKLNSWKTPSSYKIKRRSK

>arahy.Tifrunner.gnm2.ann1.H1MVFQ.1

MATHALLGILIGNLYTFVQNEIAALSGVDSQTQDLSENLSAIRALFQDAAEDQFTSHVMKDWLKKLSDAAHVLEDILEECSMESNRLQSEGWLARFHPKTILFRHAISKRMKDMVKRFQLIDDDRRRFQLPLGVRQRQQEDDDLRQTSSAITEHQMYGRDQDRHKIVDFLTQHASSIDGLSVYPIVGMGGLGKTTLARWVFNDDRVKQHFDLRIWVCVSTNFNMMKILQSIIESCTGVNPNLSTLEAMQSKVQQVFLDKRCLLVLDDVWENDKWEDLKSVLNSRGSETKGVSILVTTRDQIVASAMETCPTQSHHLQPLPKDENWSLFTHYAFGPNKEQPAKLVEIGKEIVRRCVGNPLASKVVGSLLRNKREEKQWLNVLESKFWDIDAVMGALRMSYFHLKPSARQCFCFCALYPEDFRISKEQLIHLWMANGLLEVEDEGNRQWEELLQRSFFQEVSIDANGNTTFKVHDLFLDLARCIVGEEYKAYDESASLTNLSRRVHHVSYSGLPELNQNTLKNIESLRTFIDRYPAITSVLVQYQALVLSEVQLCNSLRALRTRSSQLSTLKSLTHLRYLNIYNSYITKLPKCVSRLQKLQILKLEQCHFLTCLSKHILKLKDLRHLLIEGCQALVEMPPKMGELKQLKTLNIFIVDSKSRHGLAELHDLQLGGRLHIKGLENVLSEHDAREANLMSKKELSYLYLSWNSHFNFNSLFICPERVLEALEPPPNLKNLGINGYRGSQFPGWVRNTNIFSSLVNVILFDCNNCEQIPPLGKLPHLEGLYVCGMKDVKYIDEDSYDGVEEKVAFKSLKELTLLKLPKLERIVRDEGVEMLPLVSKVTISCSPNLKLPLLQSVEALEI

>arahy.Tifrunner.gnm2.ann1.H79UNX.1

MAENFLQPIGEKTMEEVGDEYFDELIARSFLQPHSTEENKFVMHDLVHDLAMLIAREFYFRAEKFQNTIEVDKIRHLSHNAKGDYPMSKILKICDRIKHTRTFLEINLESHIPFNMENAPCILLSQLKYMRALSFKCFPLESLPDSLIHLRYLNLSETYIVTLPESLGNLYNLQILKLTLCCNLKMLPVSMQDLVNLRYLDIWGTMLHEMPKGMSKLESLHFLSHYVVGKDEENKIKELGALTNLEQSISITKLENVVNSSEALEARMFDKDGIDSLWLSWSWYEGENMVDFEIERDILDKLQPSIGLKELAIDGYRGTTFPDWLGHSSYHNITEVTLDGCSNCCRLPSLGQLASLKHLTISKFVNLGIVGAEFYFCQNDESCLETPFPMLETLEFSSMPWWEEWHSSEFSAFPRLRVLTINWCPMLKGDLPNHLPSLQSLEIQNCELLNYCVPRTPGMTSLSIQGSNEVRIGELPPLLGNLSIERSDHLVQSVVEATTLTQLTCLTSLRISGCSSQIWFPVSAIPPSLQKLTIEDCRELEFQMDGQHHSLQQLLIEKSCSSLTSFSLLDSFPNLVRVEIKYCGKMESVAVSRSLPRLRSLEIDDCWSLKSVSTLWMAAPQLEQLSLLKCPEIDLSPTGDPHRSLRSLTISYYQKQVISAAFMNLQFHGLTHLRIEGGYRESESVKCFPKEGWLPASLESLGLVGMKSVETLECKGLAHLTSLQRLRIDGCAKLENIEGEKLPASLIRLTIVRSPLLSKRTHSFGPKFPTSPAFKLMIDGFGNPTTSTEFLYHRLSESKLKTVPRSFKSVDKKRKHT

>arahy.Tifrunner.gnm2.ann1.HD1GNJ.1

MCLCFCGCFSFWNHQLLYQNLHTDTMDSNPSSNQPKPRAVPKKVIPPDTHETLPPRVGNISRDLADHIPIHSRKSFPAIRDRLGLSPRSLSDATMPFPSTTGPRSRKAVKLTQLKVNLDKHISPSASSPSFAIHSERSTTEQHVDLLPFIFHCSLFSLCCEYSKKELISLWAAQGLFTISSRQVEDQVFDSLLSDQVVVPYRLDPSVGVFRTLLYKINQSILKFSAEYARVFDGDIDNVPDDVKAFTLRLKNCFKLDGLPEKIGKLTSLKHFVFDILGRSGSMPHGIGNLTSHQTLSNFFVGKDDGCRIVELKDLVDLTGSLCILSMENVSNFEEAREIDLSKLEIASYGGFRLPNWFSDPSYVRLVDMKLYNCMNCECLLPIGKLPALRYLLIDGMDKLERINEHFCRDGEDQQYHAFSKLEKLSIQYMRQLKEWTGVQNGDFPWLNKLQITFCPELHSLPVLSSLDSLKHLKLSCCPNIKYWREMAHVPNVAKDWLVVTC

>arahy.Tifrunner.gnm2.ann1.HE3JP9.1

MAGVLVGGAFLSGFINVVLDRLISVDAINLVAGKKLSSDLVERLRNALMDAGALVDDAELKQLDNHDVKEWLNCLRDALYTADDLLDRVCTKAATQKGVCNFLPSFLNSEERQMVKEIERVVTRIEDLEKRKGKLGLEKISTASFSWKTTSTSLVKGNVYGREDDQKALVQMLNDNNEHHLSVISIVGIGGVGKTTLAQWMYNNAELMEGFDRKAWVCVSENFNIVETTRNIVKEISTNTQDLDSFNSIQDALKKGLSEKKFFIVLDDVWSNDHHQWKDFLAPFQYGVKGSTILLTTRKEDVGSVVQTNYHPHYLIPLSEDYCWSVFAANASFPESNGSPTLEGIGKKIARKCDGLPLAAETLGCLCRRHDAEEWEKILRSDIWGFSTIDSKIIPALLISYFHLPAHLKRCFVYCALYPKDYLLDKDELILLWMAQDLLRLPNRGESFEEVGCKYFEELASRLFFKPSKYNFKRFVMHDLLHDLAIFLAGDFYCRIEELGEQEEKKVLTRHLSHISRGSLGPRISKVSNSIAKLESLRTSLYIDDLFNLESIASKFKYLRVLSFHKLDVLPDSIGELIHLRYLNLSWTDINMLPELLCNLYNLQTLILYGCTKLTMLPSGMHNLVNLRHLDLRKTSLEEMPGGISKLKHLRVLDFFIVGKHEDNGIHELGGLSNLHGSFQLKKLENIVDVKEAENARMINKNLISKLYLEWSSGGDMVSNTQTEREILHSLEPHNGLRELTIRRYRGTIFPDWLGHCSYNNMTHVSLVCCMNCCMLPSLGQLPSLKSLYIRDFGQLSSIGIEFYKNEDNPSLHIAPFPSLETLQLQDMPCWEVWNLPDSETFRQLKSLQITDCPMLKGDMLHHVLMRIVSSSMDVSKVRKLEILQDDKERFQQMLLSGDTLLFSGSESMVESAFKAMISINHISCLHEIIIRRCRKLEFPQLQLQKYDLVELLIEESCDSLTSLSLGVFPNLKNLQIKRCRNLESVSMSEAPHAALQRLSIYDCHKLVSFAGEGLAAPNLIHLQVAWCSKLEALPRDMNSLLPSLEFLDIRGCRNICRLAEGGLPPNLKSLEVGICEQQMRDPSWMPNLHALTHLRIEGHYCNNIKSYPEVGSLPHLPSLTTLEIWHFHNLETLECNELLRLTSLQQLHISFCRKLENMEGAMLPSSLLLFKMEYCPLLEEHCKNKHQLIWPKISHIPTIQVKINLVLGKKLGSDLVERLKISLHAAEVLVGDAEDKQLDNQPVRDWLHSLRDSVYWADDLLDAVFTKAATRKEVDSFWPISLLNRDKEMIDKMERVVRRIEFLEKQKDFLGLEKTTKKNFLSWRIPSTSLVEGNIYGREKDQQEIIKMLNDNREHQLSVILIVGIGGVGKTTLAQWLYNNENLMEGFQVKEWVCISEDFNIVEATKNIIGQTARNIEDFNSLQLKLKEKLSEKKFFIVLDDVWSDDGDVWKKFKTPFQYGAKGSIILVTTRVKEVASVLQTCPPYILSELTEECCWSVFANNACFPESNGSSTLEEIGRKIVNKCNGLPLAAETLGRMLRTKHDVKEWEAALKSDIWEFSIKNSKIIPALLISYFQLPAHLKRCFVYCSLYPKDHHFHKDELILLWMAEDLLRPPKRGESLEEVGCECFEELASRLFFKQCEYFDKCFVMHNLLHDLALFLAGDFYCRFEEHGKVESVTTYTRHLSYDSLSHLISEHFDSINKVESLRTLLPNNFFSHSDNIDSITCTLILKLKYLRVLSFLSFEGLNVLPESIGELTYLRYLDLSETSIRTLPESLCDLYNLQTLKLNECSSLTMLPNGMHKLVNLRHLSIRRTCLKEMPEEMSKLKHLHFLSYFMVGKHEDNGIQELGGLLNLHGSFEIKKLENVVDAKQARSARMLDKKHIDNLLLEWCSGDDMVSNTQTERDILDSLQPHHGLKELRIKGYKGTIFPNWVGHCSYQNMTSVSLYSCNNCCMLPSLGQLPSLKSLLIQGFNQLKSIGMEFYKNEGDCHCSPIAPFPSLETLEFWDMPCWEVWHLSDSESFPHLKKLQITDCPMLKGDILNQVFLRFISSLSSDVSKIRKLSIQKDHKGRSQEILLKGDSLSIKGCESVMGSAFKAMSINLLTYLQEIQISGCSYAVSFRGDCLPKSLQKLTILNCSKLEFPQQKYNFMELYIKDSCDSLSSFSLDDFPNLNNLKIEKCETLKSLSLSQPPHAALKHLFIDDCCKFVSFPVEGLAAPNLTHLRVTYCYKLESLPANMNTLLPNLQSLEIQRCSEICKLPKGGFPPNLKELSVGGCEEQLKGLSSMCHFNALIELTIEGYFGVTSYPKVGSLPQLPLLTTLRLWGFLDLETLECDQLLHLTSLQQLHIQWCVKLENMEGQNLPSSLLLLQIIECPLLGEHCKKKDEQYWSKIFYIPTIQVDGSMYRGDDMVSDTQTERDILNSLQPHNGLKKLKIKGYKGTIFPDWLGFCSYNNMTSVSLESCKNCCMLPSLGQLPSLKSLRIRGFDQLRSIGDEFYKNEGDHHSSPIAPFPLLESLEFDNLPCWDVWHLSESETFPQLRKLEIRYCPMLKGHMLNHVFLRMFSSLLDVSNVRKLDILEDDKKRSQKMLDNGETLSIRGCKYILEYAFKATIVHHLTSLQELQISGSKLEFPQQEQKYDLVELKIENSCDSLTSFSLDAFPNLKNLEISWCWNLESVSMSERPHAALQRLTIHQCSKFVSFPREGVDAPNLTHFNVTGCSKLEALPCHMNSLLPNLQSLNIRDCQKMCRLPEGGLPPNLKELTIGKQWKVLSTMGNSDALTHLSIYDCDWYNKSTRSFPEVGLLPPLPSLTTLCLYYFPNLETLECIELLRLTSLQQLSIMSCPKLENMVGEKLPPSLSLLQIKGCPLLGEHCNNKHQLIWPKISHIPNIQ

>arahy.Tifrunner.gnm2.ann1.HEBF57.1

MQQQRQEQIFIFVIAFQVPLIDSSMAEVVSSVASTLLANLATKSFQEIILAYGLKDEIKKFESSLRTIDAYLIDAENKQAKNHSIDEWLKQLREAFDDAGDILDEIEYEAKRNEVVKIYGSISIKVRRFFSYTSNLLAFRIKMAHKIKDMKQKMDEKIKEGRNLGIIEQHVNTPALEHNLPWRETASSLSFRVCGRLEEKEEIINSLMTQKLEANSIDVISIVGIGGLGKTTLAQMVYNDTQVKANFDTLMWVCVSDDFDVKKLIQKIIHAASKRENVVDANSSLEYMISLLNQNLHGKKFLLVLDDVWNENHNKWDELRTHLLEAAGDKNSKIIVTTRSQKVVDIVGSNLVMKLEGLPENECWRVFVKCAFQEEKEEEKYPRLKQIGEQIVKKCKGVPLAITTLGCLLRSKFHDENEWRKIRDNEVWNLNQEETDIFPSLKLSYNHLPPQVKQCFSYCSCFSKDYDFHVIELIMFWMAHGLLQPTREDEDAEDIGELYIKKLVSTSLLQIHDGDDLFRLFDFQNLMAFKRLKMHDLVHDVAQLTMKESSKTRTIVQEGQQEASIEWASNKFNYLRVLHLRKDMELMSSLPDDCFVKMKKHLRYLYLENCPSFKKLTDSICKMQNLQSLYLDEFPKNMKNLIYLQYLFLMGIKITSLSSMNIGRFQQLKFLYLQCPKFGPSKHRCVRDKKHQRHRHLLLSHTTFISLVHHPLVTSLNSVAHNLSFPV

>arahy.Tifrunner.gnm2.ann1.HEJ4FG.1

MAAKLEGGAYLSSFVYAISKKLSSILEDDSILEDDYSTQKLLEKLDDYLYDVEPVLDDAELKQFGNDRVKKWLVDLQDALYVADDFLDELSTKTATATPRDPGNSYDWSHSVDSIIEDSGVNVIENIVGTLESLVERKDKLGLDKSVKLDTSWRIPSTSLVVSSDIFGRDEDTKNIIKLLLDDTCDAESLVTVIPIVGMGGIGKTTLAQLVYNDAKVMGKFDTRAWVCVAENPDPVHVTRTIIGAIDSSLRDQDNFDLLQTNLKEKLTGKTFLVVLDDVWHDQRDMWEDFLKPFRYGNNGSKILLTTRNENVASVFAPINLHYGLSLLSNEDCWSVFLKHSTISTNSKQYTTLEIIGRKIVEKCKGLPLAVKTLGGLLRNKHNEGDWENVLKSKIWELSNSKIVPALRVSYHYLPSYLKRCFVYCSLYPEDYEFDKDELILLWMAENLLQPNENNTLKNTGCAYFDELVARSFFQPSSSERGLYVMHDLMHDLATFFAGKFYFKLEGSENLHGVDSKIRHLSFSSSTSFGEACKRAVHLRTALDFSWYRQSIDVEGKPWLLQQQLRVLSLPIYSLPESIGEMIYLRYLNLSKAWIVTLPESICKLYNLQTLKLRDCRHLEMLPSRMQDLVNLQHLDIRGVSLLKEMPKKMSKLKHLNFLSNYIIGEHEENGIRELGTLDNLHGSLHILNLEKVKNSDEALEAKMGNKKHLNTLKLQWLPLGYIYTDHVKILEELQPHENLKEISIKGYRGETFPDWLSLSRYSNMTKLSLNDCENCCELPSLGQLPSLQHLKFSQLHGVEKIDLAFYNNSGSFEQETPFKSLKTLEIWHMKHWREWNFPDEFDGFPKLRKLSVRDCWKLRGSLPAHLPALEELCIFNCSGLVCSLPRAPKIQQLQVYSNPVLSVSRTLEISETQLAQPLLEWVPHLQSLRAECLEIFRCQSLKSISADYLPASLTEIRVRECDSLSSVQLGPLPNIKKLTIAHCPSMECVDLPQALPGLRYLLISGCPSLVSLPALGLAAPHLEELHLWDCPEIDCFADQCLPASLKTLKVVQCEKLARWMTSKGLQSQRLTRLDIREWFDVKSFPSEGCLPASLEYLELWEFPDLETLDCKGLHHLVRLKHLQIGDCEKLANITQECLLPSIANIRMGTKCPLSRKLKEMEDLRNRFDTFHDYSGHEYDSNLE

>arahy.Tifrunner.gnm2.ann1.HI7B1K.1

MAAKLEGGGAYLSSFVDAVLEKLSSVLYDDSSFERNNLLGRLEKSLYDVGPVLDDAEQKQFTNEKVKSWLDDLQDALYKADDLLDELSTEAAIATQRDPGNSYSWSHAVDSYINYSSGKEKVVQRLEYVVARKNLLHLKESAKVDMSSWRTPSTSLVVSSDIFGRDNDKEEIIKLLLNDTCHAQSPVTVIPIVGLGGIGKTTLAQLVYNDAKVVKNFDLKVWVCVAENFDPVNLTRIILKKITSCSCDTDDFESLQTDLKDKLEGKRFLVVLDDVWDDREDIWEDILKPFRYENNGSKILLTTRSEKVASVFTATNQHYQLRLLSDQDCWSVFFKHSSLSINSKQYATLEPIGRKIVEKCKGLPLALKTLGGLLRHKYNVEDWENILESEIWELPEDKSKIVPALRVSYHYLPSSLKRCFVYCSLYPQDYQFDKNELILLWMAENLLQPREKHTLEDIGCAYFDELVARSFFQPSSTDPRLFVMHDLMHDLATSFAKKFYFRVKEIDSPQKICIITRHLSYTTRDRILSLGEDYNRAEDVRTFLNVDVHFGKIDIDNEVSPLLLQKRCLRVLSFKCFSIDSLPKSIDKLIHLRYLDLSYTPIVRLSESLCKLYNLQTLKLSYCKLEMLPSRMQDLVNLHHLDIRGNSLIEMPKGMSKLHNLNFLSGYIVGEHEENGIEGLEALDNIHGSLCISKLENVNNSRKAFEAKMGNKKHVDSLELIWRPFRDAVDVQTERDILHELQPHRNLKELSIQNYGGEIFPNWLGLSCYSNITTLRMNGCKNCRQLPSLGQLPSLQRLEFSELDGLEIIGCEFYSSDESFQQETPFKSLETLIFKYMSSWREWHFPDKFDGFPKLRSLSMRHCPVLTGDLPAHLPALEELLIVRCEELACSLPRQPKLHRLLVKGTVAFMGAEPNEAVIEETRLAQFVLECLPHVQPPGPQTLNIKECSSAISISVDYLPPSLQYLTISDCSKFTFSDQLQHKFLTEIVVDGCESLPLFPLAALPNLKRLSIESCENMEYIEVAEALPRLYYLQIAVCPRLVSFPALGLAAAAPHLEELEIWHCPEIDSLGEESLPLSLASISIINCQKLGGWITSRGFQGQGLTQLILKQWNDVKSFPREGCLPASLQSLHLSEFPNLETLDCKGLHQFTSLQQLTIVLCPKLENITDEKLPDSITKLRIKGECPLRSKLEEMNDPRIQSETDDDTERGAYPDYESVSDYESS

>arahy.Tifrunner.gnm2.ann1.HIUY82.1

MDNLDPNPIYDVFLSSEIEPDSNQSYTSCLCSCLQKAGISVFRDDDALPRNEDNHHISPSQLQAIQNSRISVVVLTEGYAGSERCLQVLEQILECHRIAGQFVLPVFLGLYLDAVRFQIGAFGRAFEDLTKRISDKDQNVGRLLSQEFVESDPTTGLARATATIHMSDNDKDIKEHVELVRGMLKRTDLFIAEYPMGAYRAYLVPLLLEPKPTGVVLLGIWGMVGIGKTTLAKSIYNQIGDNFEGKSFLPNIKHVWEQQTRRAYLQERLLMDICIPSERQVKIDSIENGKKKLKEILSHKKLLVVLDDVNKLEQLNALCGSRGWFGPGSMIIITTRDRGLLRNVDMMVGVDHVHEMKEMDENESFEIFNRCAFKSHGFDYRKDKRDISAFNTQAAVAYAGRLPLALEVLGSKIFTSFNLDSALDRCTESLHPVLKKVFKRHIDDDLIGTEKEIFLDAACFLNGMNRDEVVEALNAFGDFAKIGIRNLEDKCFVAFDHMNNLGMHVLLRDFGREILGEQIRVEPAFSTAAIIAVTIVNDHGFLSKDHGLFLTK

>arahy.Tifrunner.gnm2.ann1.HVB0T8.1

MGEWTYDVFLSFRGKDIRQRFIGHLYEALRGRGIHTFLDDEELERGEDFTRSLLTAVHESRMLSLSSLPTMQLRLSAWMNFIMDCAEAKDQIVLPLFYEVDPFHVRRLRGSYGEALTKHEERFRSGEGSSMVKNMEKLEKWKMALEQAANISGYHFKIGDESERMFIKKVVAAVSKWTHRGSLHITDHLVGLELHVRDVMKLLKVGFDDEVCIVGIYGIGGIGKTTLARAIYNSIAGSFEDTCFLGNVRESSAAYGLVYLQKILLHKLTGDTTIELGDVNEGISVIMRRLRQKKVLLIVDDADNVKQLRAIVGDSKWYGSGSRIIVTTRNKGLLASHGVERTYEVGKLNEKESRDLLIWNAFRTNEVDPSYVDILNRIVTYASGLPLALEVIGSNLFGKSNEEWESALQQYKRIPNKEIQQILKVSFDDLEEDEKNCFLDIACFLNGNKVEYAKIILQALHGVCPKSSIRVLVDKSLVKIDDDIVTLHDLIQDMGKEIVRQESPEEPKRRSRLWLFEDIKHVLEENKGSNKIKIIDLVLPNSREKIKWDGEAFAKMNNLKILFIHQHCCLEGPKKLPNSLRVLKWRYYPSRSLPSDFCPKKLVMLDLSYSLLNLIQLQKKFMNLQFLKIDGCELKHVPDVSFAPNLEELSFRQCHKLIDVHESVGLLEKLKVLDADGCSRLKRFPSLMLPTLEEFYLSNCSSLETFPEILGKMENLTTLELINTRIAEFPSSIRYITRLQGLVVRFSGIFKLPSSIFVLTKLKYLSIQNCDGLLLCEHEGEEQISSMAISNQLHLDFSKCNVSDEFLQIGVPWLSNVKKLDLSINKFEVLPASLEECFFLKELILDGCWNLKEIRGMPPNIEELSFRKCKSLIEVHESVGLLGKLRVLDAHGCCSLKHFPALMLPSLEELYLSNCSSLQTFPQILGKMENLTKLELINTQIAKFPSSIQYITLLQELVLRFSGIFELPSSILVLPELKYLSIQNCDGLLLCEHEGEEQTSSMAISNQLHLDFSRCNVSDEFLQIGVPRFSSVKKLDLSINSFKVLPASIKECFFLKELILDGCWNLKEIRGIPPNIEKISASRCRSLRDLDLTLVPACTKECHFLEELILDGCKNLKEIRGIPPNIQILHVPTCTSLTSSCRSMLLNQGLKHSCMGSSISFWFRNKFPAIYLCVIAEQSCAPFFLKMIINGHDVPQFSMFFLEKDMLIMALSQKLIKINDEVHNLLLQNEWNHVELFNDPVMQSLGGVEPDHSLRLGFNVFKQSDVMEDIQFTNPLLKKEEDKVSLPLYPHLSLNDDVIQINGTTIVQASKDHPAKAQDVEIIGGSENGQESPLIQDDDDNDDDAEMEAFYASLNATNGLSHSHDKFTINAPNEEVREALKTVQDFITNNDASVLLHDEHYNVINNSLHYLSGLSSKDGISGEVETLILEASWLFNHCSREYIESCMKIKSTASKLQRVDELESALESNKHRFRENLALENELRQKLDWMEKRKKEMEEEINAIKAKLCDCESEKKVVVQKKREVFQEGKTLKAQLEEWGEKVPQLRHQESVAKSNHAKFTELRDYIGWPIQMSLSSDAISLFREMEKNNLDLNIFGDRWDVPSGKLNDARELLSASKRLET

>arahy.Tifrunner.gnm2.ann1.HZ3BCY.1

MYEWILLSILQLKIRCREHLKNGKMKVKGKKHMKSTFFAELLPLPYSECKQKLLPIPSLSYFLALVNMAASLVGGAFLSSFINVLFNRLSDPMIINMMRGKKVDRKLLQKLETILNVVEAVLNDAEKKQITDPAVKRWLENLQDVVYDADDLLYEFVTKAATQKDPGNFLSRFLNLQDREMVNRIEEIIARLQDIANHKDIIGLKKIAANNLSRRIPSTSLVKKYDIFVGRDKERYTIVKLLLDDANDGDLSVIPIVGMGGIGKTALAKLVYNDDRVQQKFHVKAWVCVGEEFDVLMVTKAVIEKTCNTTQNHLKNALAGKNFLVVLDDVWSSNREAWESFLSPFECGSEDGKILVTTRLDPVASMMKTKYNKAHSLSLLDDEQCWSVFASRAWDLAESRGHSALEKIGRKIVEKCNGLPLAAQTLGRLLRGKDNEKDWNDVLNSEIWEYSEEESGLLPALRISYFHLPSYLKRCFTYCSLYPKAYELDRDELMLLWMTEGLLQQPKSGNILEEVGYEYFDDLVSRSFFQHSNYDENLFVMHDLMHDLATFHFSSGEEHLFHDSIGELIHLRYLDLSETLVMTFALFRSLTQLYISNCSSVISHIIFREFFSPFIAMVIYQELQELRIPNATPTTFLT

>arahy.Tifrunner.gnm2.ann1.I08KEP.1

MLLRGGEPGEAPRCCAMVKQERLAGGELWFEEIRDREEEKERRGSRLIWKGERQRLQRRDGETAAAGWRLPFPFYCHHHQSEKLGPDLVERLQTALLAAEALVLDAEQKQLENEFVRKWLHSLRDAVYKADDLLDSVFTKAATRKEVPISTFLPRFIMNYKDREMIIEIKRVVRRIVDLEKCKESLGLEKIPTRSSSSWRTPSTSLVRENVYGREDDQVALIKMLYDNNQHQLSVIFVVGIGGVDNASFPKISNGSSELEGIGRKIVKKCDGLPLAAETLGRLLRTKHDVKEWSKILMSNIWGFSVTDSKIIPALFISYFHLPAYLKRCFVYCSLYPKGYQFDKDELVLLWMAEDLLRPAKRGETLVEIGRECFDDLASKLFFKQVQDNGGYFVMHDLLHDLAKFLAGDFFCCLEEHGEEEEIRTMTHHLSYRSLRHTMPKYFSSIDKAKSLRTFLHINHLPRRKMSQLNLLPPSFGMENAARDVLSKFKYLRVLSFHKLDVLPDLIGELIHLRYLDLSWTHINVLPESLCNLYNLQTLKLQNCLNLTMLPNGMHNLVNLQHLDIRRTPLEEMPREMGKLKYLQHLNYFVVGKQEDNGIKELGGLSNLHEALEILKLENRSNGRRISDKIHIDELSLRWSLDGDVVSNTQVERNILENLHPHYGLKKLIIEGYRGTMFPNWIGHCLYHNITSVSLVSCNNFCMPPSLGQLQSLRSLDIEGFGQLDSIGMEFYKNRDNDSLCIAPFPSLEFLKFCNMPCLEVWHSFDSYAFPQLKKLQLQNCPLLKGDLPNHLPALERLDITGCQRFVSPLGFGNSHPGFSIVIYETTEGSQELLFDMKNLLIRGCHPEVESMSKAMSINHLAFLKVMHIFNYSSAVAIPSNHLPKSLARLRIDNCSKVEFPKQQQHQYDLVELVIENSCDSLTSFSLELFPKLELLQIRKCASMESLSMSLSQHTALHRLVISKCPNFVSFPGEGLAMPNLAYFVVMKCYKLKSLPCHMNVLLPSLEFLNIYGCPGIQAFPEGGLPPNLKKLYVGGCDMQLSSLSCHRWETLRPSLIKSFPQVGSLPRLPSLNRLGLHHFHNLETLECNELLHLNFLQQLIIEECPKLENMTGKKLPSSLLQLQIDYCPLLLQLCEMKHPKVWAKISHIPIIEVNGKQIL

>arahy.Tifrunner.gnm2.ann1.I28IWP.2

MEIREAKFLCSYGGEIKPRGRNNKLAYIDGTNKLLYVDRRIDFTAMVSKISSLFDGASNRDNFFKYQVPGSDDLNALVSVTNDRDLHNMMLEFDQLYRDSPRFARMRLFLFPNPNKPLDSVKPNSNRLSSNLWKYDVFISFRGKDIRTGFSSHLVEALNQNQIKTFIDDELHKGDCILDSLTRAIENSSISVIILSENFVTSSWCLHELLKIMECGRKVIPVFYGVDPSDVRKQLVSFNEKFKSHLQSDINNLLKWMEALAKIADLDGWDSGSCRDDFELVEKIVKDILRELNNHCLHKDVKSLVGVDKNHEKLEMLLSSVPDEQTGIIGICGMGGLGKTTLARLLYQELSHQYEGSCFLGNVRERSEKYGRGLDELRNQLYLELLQGKDCENTMANTTSKCRLSRQRNFIVLDDVSSSKQLEYLAGDLQCYGAGSRIIVITRDKSVLKKGVEKFHQMEVLDFQDSLILFSLNAFNQDYPKRGYQELSWKAVTYCKGVPLALKVLGSFLCSKSETEWDNVLQKLEKNPNAAIQNVLRLSCNGSDYQGNHIVLDIASVFKGEQKEQLVNVFDSCSFYTARGMSSLLDEALIAMFDHFGLSHVLYKKWGRESFVRELSRIVKVIAICGMLVMLVMFWKIISFPPFNIYVFLDTSTLLLLLNNNA

>arahy.Tifrunner.gnm2.ann1.I2M58K.1

MAEKLYGGAYLSPLVDAVLDNTTSILEEDDSFLERNNLLERLQTCLYDVGPVLDDAELKQFTNKRVKKWLVDLQDALYIADDLLDEISTIAAIDVTQRDPGNSSSCSRLVDWYIQDNGDMEKIVGKLESVVRRKHYLGLEKSAKVDMSWRIPSTSLLEPSEICGRKEDKEAILKLLLDDDDAADGDLSVIPIVGMGGIGKTTLAQLLYHDDKVEKNFNFQAWVCVSEEFDIVKVTKTIIEAITSSSCNLTDLNLLQHDLKQTLSRKKFFVVLDDVWNENYDDWNRLLKPFQKGVKGSKVLITTRSKKVASVVQTVLPHELSLLSDDDCWLVFSKHARLSTVSVENPTLEKIGRDIVKKCDGLPLAAQALGGLLRGNSDVRYWNHLLKSEIWELSDEKIKVVPALRISYYYLPSYLKECFVYCSLYPKDYKFDKNELILLWMAENFLQPVGKKTLEEVGVEYFDELIARSFFQPHNTLEKVFVMHDLIHDLAMIFAGGFYFRAEELENAVEVDIKTRRLSHNAKGNYPMSKFLGVCDRVKHTRTFLEVNLESWIPFNMENAPCIMLSQLKYMRALSFKSFPLESVPDSICELIHLRYLDLSETYIVSLPVSFGSLYNLQTLKLFRCKKLKMLPVGMKDLVNLRHLDIRKTCLSEMPEGMSKLKSLQFLSDYVVGKCEENKITELGRLANLQQCISIAKLENVVNGSEASMARMFDKDGISSLLLRWSLDKDENTADSQIEREILNKLQPHSNLKGLEIVGYRGTTFPDWLGHSSYHNITKITLHSCRNCRMLPSLGQLPFLKNLYISEFDSVGIVDAEFYFNQNGESCLETPPFPMLETLWFQSMPHWKEWRSLEFNAFPRLRELTIRECPMLRGDLPGQLPSLQSLTIFNCEQLSCCLPSAPAVSTLYIDGGNKVRIRELPPSLRRLSIEGNHQVESVVDVITHTKLIYLTSLSISCCSSHIWFPVTAIPPSLQHLAIVDCRELEFQMDGQHHSLQELYIRSSCDSYKSFSLLDAFPNLKDVDIMECEKMESIVVSGSLSSLRSLFIKNCRSLKSIWTIWMATPQLDDLRIVGCTEMDLSATGDTHRNLRSLAISYCEKLVSSAAFMNSQFHGLTDLWISGEHDESMKCIPKEGWLPASLESLTLFRMKSVETLECKGLAHLTSLQRLTIDECPKLENMEGEKLPSSLIKLSIYKSPLLGNRCQKKDPQVWSKISHIRAIKVDSRWIW

>arahy.Tifrunner.gnm2.ann1.I8BVUC.1

MASTSSTSKRSCKYHVFLSFRGEDTRAGFTSHLYAALTRKGITTFIDDSNLRRGDVISDELLTAIEESMFAIVVLSPNYVSSTWCLDELQKILECKHKLGQHVEAVFYGVEPSDVRHQKGTFGEAFWKHEYKFGQESDKVRRWRDALTQVAGYSGWTSKNQNEAILVENISQSIHKKLIPNLPSSMNKLVGIDSRVEQVISHIGIGLSDVRYIGICGMGGIGKTTIARIVYEAIQSEFEVSYFHASVRETCEKNGIVQAQKELVGHINGSSSNFNNEYDGRRIIQASLYRKKVLLVLDDINEEKQLKNLAEEQDWFGSGSRIIITTRDMHLLKIHDANKIYNVEGLGESEAFDLFGLKAFKQRKPAEEYLDLSKQAVQYCAGLPLALEVLGSHLCGRPVKDWHSALEKLMSFPHVGIFDTLKISYDGLDTMDKDIFLDIAYFFKGRSKDGVIKILERCGYHVEIGIATLIDRSLLTMNEKGRLEMHDLVEEMGKHIVIQESPNDPSKRSRLRGYEDINLVLTQNKGTEATRSIVLRDQDFYQEQDIMRWRDLTFSDICQLKFLILDGVEAPILSYIPCSLRVLRWRRCPMETLPFMDQGCELVEINLSDSSSIVQVWHGKKFLEKLKYLYLKCLYRLKQIPDLSEAPNLEILDVQCCDELNDFPSYLTRHKSLVKLILYRCSSLETLASKLEMNSLKELDLGFCTSMRKPPEFGECMKHLSVLSLWGTAIEELPTTVGCLVGLKDLRLQHCQRLTCLPDSIQKLKFLTFFNLSYCPNVFQSLHSLFGLTSLDTLILSGCFVISQESWSYNLGNLVSLTDLDLSHNNFVRVPINIHKLPRLRHLNLDYCPSLKVLPELPSSIRELNARDCASLDTWHSNVISKVCCGFAASANHDSDGLLHMWVAQNEGEEIPLWFVHQEEGNGVSVTLPHNETMALALCFRLCPRRSSRNYVVNLPVICNGKEFIKKHLTVLRETKNSQHFILCLSSDYFVDQFCQDYRFELVFPSNVEMKAHSSGARWVCKQDIQDLKNSGTETSKRKATFDLNMNITPPSSPCRKKMFMVTPSSVSPLEEEEEE

>arahy.Tifrunner.gnm2.ann1.I91QXC.1

MAEALLGIVLENLIPFVQTEFAAFFGIKEKAEDLSRTLELIKAVLDDAEQKQWSNRPLKVWLQQLKDAMYVLNDILDQLPTESSQLGFLSSLNPKKVMHRRELGQKLNEIIGRLDRIAQARSNFDLRQGLRERPSEVAEWRQTSSTIAVPQVYGRDEDKGRVMEFLLSPARSSEFLSVYPIVGLGGLGKTTLVQLIYNDPKVGNNFYLKIWVCVSEHFTMESILRSIVEAITDEKYELKALDVMEKKVKELLQSKKYLLVLDDVWKRSQEMELGLTQDKWDKLRSVLSCGSKGSSILVSTRDKHVATIMGTCQPHYLDRLSDDDCWSLFKLRAFGPDREERAELVAIGKEIVRKCGGSPLAAMALGGLMQSRSTEKEWLEVQKSEVWNLPDENDIIPVLRLSYSCLSPTLKQCFAFCAIFPKDMEMVKQELIYLWMANGFISSRPNLEVEEVGNMVWNELYGKSLFQDVRADDFSGEIYFKMHDLVHDLAQSISGQECICLEKQNLNDSSRNPHHIVFHHIVKRQFKKRSVEKAESLRTLYQLDSYGFPFSSRLIRTNNSLRVLCIYARKIPSFGSLSCLRYLELRGLDIKSLPGSICNLRRLEILKLIGLNNLRRLPKHLTRMQNLRHLVIDGWNIPSSMFPDAHKLRHLRTLSIYIVKSKKGHSLTELHALNLGGKLYIKGLENVGSISEAKNANLKGKQDLRQLILSWRKSGKRKSVLGAEEVLEALQPHSTLKLLTIEYYEGLQWPTWMQNNSATHNLVSLRLVKCGKCGHLPPVGKLPFLKKLFISGMDDVQYIEEDESYDGVEAMPFPSLEELEVARLPNVERLLKRETTHMFPSLSTIKVINCPKLQLPCLPSVKNLTVWECSNEQLKSISNLNGLNQLHLWVNDEVSCFPEGMMSNMTSLATLEIKYFSELKELPSDITKLTALSNLTIYDCGKLACLPEQGFEGLSSLQRLSINKCTSLGSLPDGVRHLT

>arahy.Tifrunner.gnm2.ann1.I9PUIS.1

MAEDLLRPPKKGETLEEIGCECFDHLASRLFFKQVENDDEKYFVMHDLMHDLATSLAGDLYCRFGKKEEMSILTRHLSFRNSIPKKTCSSNKIESLRTLLYLEDGAGFWKVGATLPCDMLSKNKYLRVLSFDTRNIFPNSIAKKLIQLRYLDLSWSDIVFVVGKQEYNGMEELGGLLNLHGSPEIEKLENVVDANEARSARIIDKKHIEELFKKLSGRNGSDIFISKGFRNWKKVNDGKNCAFLKHIGDPCSPHNNAVGACLDLLNQASHIQNVIEDHTSEKIQKNRLRLKSSIDATRWLTFQACAFRGHDETPE

>arahy.Tifrunner.gnm2.ann1.ICL5HQ.1

MKRNPDKIKGKEQSWRKALREVGCIAGFVIRKSKNESEDIKNIVEQVTHMLEMKELFVANHPVGVESRVEEVIQLLKDQQQENPLLLGIWGMGGSGKTTIAKAVYNKIFREFEGRCFLLNIREVWDQDNGILHLQQQLLSAIYKTTKIKIENTESGKSILERRLGQKRILLVLDDVDKLEQLNSLAASRKWFCPGSIIIITTRDEHLLRCLRIDKLYKPCPKEEFASLANEVASYCERLPLALEVIGSHLFNRKVYEWRSVLNKLKTIPNNDVQKKLKISFDGLSDDRNREIFLDVAFFFIGMDKNDVTDILNGCGHSAEIGINVLMERCLITVDTKGKLGMHGLLRDMGREIIRESLPMKPEERSRLWNSDEVLNVLSKDMLPL

>arahy.Tifrunner.gnm2.ann1.IEL5NN.1

MAIPIPIPEFLQEYTVNLVAEYVTNHLDYVWNYEKKFDDLSRVVKELQEERDRVHDKAEEEEDRYGREIYNDVKVWLDRIDEVISEYEKFKEEHRKNGEYPLSLSNLETRHRRSKTAQDIEEKIRELQQEKHDSISHCQGASSMGFAFANVDYEAFDSRKEITRNITRALEDSRATAIGIHGPAGVGKTTLVIEVANKAWKDKLFNVVIMVNVTKSPDIRKIQGQIAEMLGMKLEEESEHVRALRIQKRLKKEKENALIILDDMSVKVDLDMLGLGIASETYSDDIDCQKNLIVPEGRKSSAADNFPPSKNKTKQSPKDGSAEEIEKTSVGSGKLKTEERHKGCKVLLISEMRQVLSQMGVKPSLIFSVNVLSDKEAETLFKKRAGIVDKNFELGKIAAEITKKCHGLPMSIVTTAKALKNQSPSVWEDTRLKLHRQSLTGTPEYSTRLSYNLLENEELKLTFLLCASMGHDALIADLVKRCIGLGFLQDIYTVREARDRVQSLLVKLKQSGLLSDSYSSDHFSMQNLVRNAALSIASADNHVFRLTKGKLDEWPDEDKLEKYTDIFLQHCDFIEEFPRRIRCPRVRVFHIDNNDPHLKIPDNFFQEMKELRVLILTGIHLLPLPSSIGYLTKLRMLCLEHCKIDEKSLCIIGELKNLRILSFSGSEIESLPVELKNLSKLQIFDISNCTKLGGIPPKVISSMTRLEELYMRNTSIQWKINEEQENQSEIASLSELGHLNQLSNLDLQIPSAAHLPKNLFFDRLQNYKIIVGSSERYSKQEFKMPEKYELVRFLAIQQKYGFDIHSQNEIKMLFERVENLLLEKFNGVQDLFYELNLKGFPCLKELFIVSNSDICSLINPKDRKRPEMAFPKLELLDLYKLKNMKEICSSSCELSKPSFGKLKIIKIMLCSALKNVFQISLVGFLTALETIEVSECSSLKEIVHVENTSQIGTLGFPELRHLSLRSLVEFVGFDPILLEDSRILFHGRVGVTKLERLELSAIQIDCIWNDGQSSHFGNLIHLDVNNCGNLKYLLSLSVAKGLVNLQSLFISECEQMRRIFNQEQCRDSSKKDRIFPKLKNIKLSSMKRLSEIWSSGVRKDSFGKLDTLIIEKCDKLVNVFPSYLVGIFRSLSSLRVTSCNSMEAIFDLPFKNKYAKYVSRLQEVHLETLPKLEHVLRWRENQEGIFYFNHLQKMYVQDCENLENMFPVFVAENLENLEYLVVLDCIRLREIVAKGEEEDIKRGREFKLPKLTTLNFSKLPKFKSFYPGVNGLSCPLLNELSIELCDNLELFTEEKVDALSGKERILFPEEVINNLKSMQIEWQHAKSSTRYRRDNLEELRLSRLKKTEVIYSFLHSNPNLKSLWLNDCSFKVLMPLEKPANFESLGVVPKLKSLKLTNLSRLEKIGFEQDAILQRIEFLILKNCHGLSTIALSSVSLAYLTNLEVVDCQGLKYLMSLPTARSLSQLNVMKVIDCKSLTEIVSEQGKEEENALCEVNILFKQLKTLELVSLKSLESFCNSESCVFEFPSLEKLVVSACPKMESFCREVKSTPILQKIYVVHDKEKKRWCWNGHLQATIQDMFKNKKYFEGMDKISVHEHPYLQEVWQGGKNDLQKDWFYNLESLTLSGIEFELYAIPSNVLCCLKRLKELTVRDCDKIKSIFEMNDTVFRGTFQLKKLYLDWLPNMTHVWEMDNQGISCFQNLQEVIVESCDKLETLFPTALARDLRMLEQLDVSFCDELLEIVGREDGEKEKKEAEEGTTGKSLFPRLTKLKLNILPQLTCFCSTTFTLGCPELHVLDVIGCNKLQLFQGQLKAEDSTSITIHPTFSSIQVISEVEHLCLNWKDTSVLCSLLSQVADDVKLQYLNVLELYLDDDVNEKSTLPLQLLEKTPNLEKLEIYNCIIQEIFSQLDTPKIINSNGTLGNLKQLHLFDLSELSSISGLEHLPKLQLLYVSECPSLTSLVVQSGSNLKELKISRCHRLACLFTSRTARMLKNLKEMCIYSCESMKEIVGEDEQDETQENQEMIKFERLERIRLEDLESLDCFYPGNATLQLPSLIQVEILECPKVKIFSCGPINAESFRGIRYSYYQDDDLVFHSDLNSSIGRLFLYQGHLALGDFPQLEEIWLGAQEIPSDFSFSKLKSLKVEGCEFLSDAVLSSHLFPLLENLEELCVQKCEHVKAIFDLKDTSTRDPNMIVTLGLKTLILKQLPTLRHVWNKDPEGYLSLPSLCKVIVDECKSIKTLLPSSNTEKEEPCAILPSHLLSFLSKLEELQVRKCDSVGAIFDMIDTPKHDADMIIIPLRTLILERLPNLCHVWNSDPKGSLSLALEKVTVNECKSIKSLFPASVAKDNIQKLDVRCCAELVEIVANNEATIKEANKEVTIFTKLTSLTLCDLPNLKSICSEMQIIDSSKLDGCEKSSSSAILPSYLLPCLSKLEELQVQKCDSVEAIFDVIDAPTHDANMIVIPLKTLILEQLPNLSHVWNKDPKGSLSLSLEKVTVNECKSIKSLFPASVAKDNIQRLDVRSCVKLVEIVANNEAAKEEANKEVTLLTKLTSLTLCDLPNLKSICSEMQIIDSSDVDGCEKLSSSAILPSYLLPCLSKLEELQVQKCDSVEAIFDVIDAPTHDSNMIITIPLKRMTLEHLPTMRHVWNKDPKGSISLALEAVTINECKSIKSLFPASVAKDNLQKLEVRNCVELVEIVARDEAATKEASKELAMFPKLTLLVLCDLPNLGCICSGMQILDWPLLEKLNVYHCENLKVLAANSPNSPRSYPEDQDTITIESHGILSTGSVAPQLVKLSLNKEDIIMIEQELPHVDLQKIKTLTLQSFKDDSDTFPNDFFTKVPLPNIKKLRLIECAFEALFHSQRPETEQHTKILSQLKKLELKNLYKLKSIGLEHSWVVPLLENVKVLKVLECCCLTNLVPSTVQSFSCLTELHVEGCARLQYLFTSSTAKRLVALDEISVSNCELLETVVAHEESDKPDDQVIFPELWNLSLSKLPRLRSFYTGNSTLKFRWLMNVTITECKCMETFSHGNLVAHKLRTVDIDEEHWSKVDLNTVIQQQFEKGKEATLS

>arahy.Tifrunner.gnm2.ann1.IFV7W8.1

MSYQNQEYNAYISSGLSFRDPFFSGLYHALKSVGLHVLADHTDPKTNKLLLSGAIERCRVSIIVFTIAYAGSTFTCKNSSKYWSVIGGKIRSLSGYFGVILSRTLQGIPRDENRMLSYETALRQAASILPRNNRRVMSHIIGHVTSLIDSTKLFIAKHPVGVDSRVQDLIQLLNNQKADGVLIIAIWGMAVIGKTTIAKALYNQISHNFEVRKFVPDIQDREGDYPRGELLSFLGDQVKTKAHNFDSRNIWWEGLRCLKVLLILDNVRSERELEVLPVTHEVFGPGSIIIITTRTKHDRLQETRVNHIYRVKEMDYNECVELFSWSAFNKATPERSFSGLINYAIEYSDGLPLALVAVGSAVSEKSIEEWENVLDSFKRFPFQDVWQVLKENIDSVGSEEKEIFLELAYLSHLLIGVDRNDICQILQGAGHPDASRAIKGIEEHSLVWFDEDKLCMNRLLQGIGREMYMKESSIKPQQRPYDVFLSFRGKETHSKFISHLYASLENAGIYVFKDENGLARGENLSISLLKTIGESKTSIIILSPNYAFSRWCLQELEDIMICCKNKTQKVLPVFYHIDPSEVRNQTGKFGQAFDNLMKRYPDKIKGKEQSWRKALREVGCIAGLVIRKSKNESEDIKNIVEQELFIANHPVGIESRVEEVIQLLEDQQQEYPLLLGIWGMGGSGRCFLLNISEVWDQDNGILHLQQQLLSAIYKTTKIKIENTESGKSILERRLGQKRILLVLDDVDKLEQLNSLAASHKWFCPRSTIIITTRDEHLLRCLRVDKLYSMKELNDKESIELFSWHAFKEPCPKEEFASLANEVVSYCERLPLALEVIGSHLFNRKVYEWRSVLNKLKTIPNNDVQKKLKISFDGLSDDRDREIFFDVAFFFIGMDKNDVTDILNGCGHSAEIGINVLMERCLITVDTKGKLGMHGLLRDMGREIIRESLPMKPEERSRLWNSDEVLNVLSKDMGTKAIEGLALNLPKSLKATHLKTEAFKEMKRLRLLQFANVQLIGDFKYLSTDLRWLCWHECPPEYTTANFYQGNLVAIDFKYSKLDLVWKKGHMMKNLKLLNLSHSQHLTQTPDFSNMPNLEKLILRYCPKLTSVSHTIEHLKQVLLINMKGCSGLRVLPRSIYKLKSLKTLILSGCSLIDKLEEDIEQMESLATLMADKTAITQVPHALLRLKSIVYISLCDFEGLWRNVFPSIIWSWTSPTNNFSPQVQTFLDLSNIVSLIVPNRNSEGLSSIIRGLPQVQNVGLECGSQLQIADVASDTFKVTNCNEMIVTSSASNVSKRSTLSLAGCCSKHDIIEAEISLNLILIQMGMSCAVTAVLRDNIFQKFSARVPRDCWLPGNKNPDWLAYSSEDSSVTFHVPQVNGRKLKTVLLCIVYSSSPINVPSEAHIVKNLFIINHTKTTPYVYDGDTLASLKDEEWQKVTSNLEAGDKVQIVVVAGLGFTVKKIAVYLVYADQQAEGIVCADDMVAYANFTVHGGDNNECLKSQESLSNIEND

>arahy.Tifrunner.gnm2.ann1.II44X3.1

MAASYFKYDVFLSFRGHTRREFTDALYHALVNKRIETFRDSENLRIGEELEGALLEAIERSRMSILILCDEYPTSKWCLDELVKIMECSGNGTKRPVLPVYFRVAKSDVQFQKNKYETAMAAHQAKGRNNHKLEAWKSALSEVGKIYGQLCDQKTAWGEAIDNIVEEVTKRLPPLPLYIDRPLGCDSELEEAKSLLEIGSHATCFMLGIHGDGDEINKFVAELYNKIRPHFVTASFLSNISEKTNESGGGLEHLQETLLSEMGEEVRTKIGSTFKGSSEIKQRLGQKRVLLVLDDVDNIQQLDSLARRMDWFGPGSRIIITTRYEDVLDDHILNNCVEVKKYCIAEGSSSTVKEENVVGLEKDFEIVINQLKEEDSPGNVVSIVGMGGLGKTTLARKIYNSDEVKMLFPCRAWATVSKDYSGKEVFKSLFKCLKPSASKFEDSSSEEELKQKVKKCLEGKKYLVVLDDVWDSKAWRTIKNCFPENNNGGMILVTTRNDQVAYVSESKKPHHKLSFMDKERSWELFHKEVFCRRNCPPELESIGRSIVETCKGLPR

>arahy.Tifrunner.gnm2.ann1.IS7D7R.1

MAGVVVGGAFLSGFINVVLDRLISADAVNLVVGKKLSSDLVERLRNALTDTGALVDDAELKQLDNHDVKEWLNSLRDALYTADDLLDRVCTKAATQKVTLLGRIFNSEDRQMVNEIERVVRRIEDLEKRKGKLGLEKISTASFSWKTPSTSLVKGNVCGREDDKKALIKMLNDNNEHHLSVISIVGMGGVGKTTLAQWMYNNAELMEGFDQKAWVCVSENFNIVETTRNIVKQISTNTQDLDSFNSIQDALKKGLSEKKFFIVVDDVWSNDHHQWKDFLAPFQYGVKGSTILLTTRKVDVGSVVQTNYQPHYLIPLSEDYCWSVFAANASFPESNGNPTLEGIGRSIARKCDGLPLAAETLGCLCRRHDAKEWEKILRSDIWGFSTNDSKIVPALLISYFHLAAHLKRCFVHCALFPKDYHFKKDELILLWMAEDLLRLPKRGESLEEVGCKCFEELVSRLFFKKLQDNDEYFVMHDLLHDLAIFLAGDFYCRIEELGEQEEKKVLTRHFSYFPPGRLDRPISKVFNSNAKLESFRTSLYIDDLFSMESVASKFICLRVLSFHKLDVLPDSIGESIHLRYLNLSSTDINRLPESLCNLYNLQTLILYGCSKLTMLPINMHNLVNLRHLDIRKTCLEEMPGGISKMKHLHTLSSFVVGKHEDNGIKELGGLSNLHGSLELKKLENIVDVKEAENARMTNKNLMNELYLEWSSGDDMVPNTKAERDILDSLQPHNCLRELTIKGYKGTIFPDWLGNCSYNNMTSVSLESCNNCCMLPSLGQLPSLKALRIKGFGQLKCVDMEFYKGIGDPSFHIAPPFPLLESLEFYNMPCWEEWHLPDSKAFPQLKSLQIRDCPILNGDMLHQVFMRIVSSSLDALKVRKLVIIGFREGWLSARIPGMSLNGDTLSIMGSESVVESAYNEMMSIKHLPSLQEVEIIECSFAVSWPNNCLLPKSLQKLTIRQCSKVEFPEQKYDLVEVLIDSCDSLTSLSLDVFPNLKNLDIHHCRNLESVSMSEAPHATLQRLSITFCFKLVSLAGEGLAAPNLTRLQVAYCDKLEALPRDMNSLLPSLQSIEIFCCPNISRLAEGGLPPNLKELHVGFCEQQMRDLSWMGNLHALTHLSIYGDYCDNIKIEELGEQEEKKVLTRHFSYFPPRRLDHPISKVFNSNAKLESLRTSLYIDDLFSMESVASKFKYLRVLSFHKLDVLPDSIGESIHLRYLNLSSTDINRLPESLCNLYNLQTLILYRCTKLTMLPINMHNLVNLRHLDLRKTCLEEMPGGISKMKHLHTLSSFVVGKHEDNGIKELGGLSNLHRSLELKKLENIVDVKEAENARMTNKNLMNELCLEWSSGDDMVPNTKAERDILDNLQPHNSLEKLTIKGYKGTIFPDWLGHCSYNNMTSVSLKSCNNCFMLPSLGQLPSLKALRIQGFGQLKCVGMEFYKDIGDPSLHIAPPFRLLESLKFYDMACWEEWHLPDSKVFPQLKRLQITDCPMLKGDMLHQVFMRIVSSSSDALKVRKLFINKYEAGWIPGLSLNGDTLSITGSESVVESAINEMMSIKHLPSLQEVEIIGKCSKVEFPQHKYDLVELLIYSCDSLTSLSLDVFPNLKNLEIRGCENLESVSMSEAPHAALQRLSISGCHKLVSFAGEGLAAPNLTHLNVSRCSKLEALPRDMKSLLPSLQSLQIYGCPNICRLAEGGLPPNLKVLDVAIGEQQMRGLSWIPNLHALTHLTIYGYYCHNIKSFPEVSSLPHLSSLTTLHIQDFYNLETLECNELLRLTSLQQLHIYDCSKLENMEGEKLPPSFLLLQLTGCGLLGEHCKNKHQLIWPKISHIPTIQRFLSRKYFEDQAAIVLRACSSGMDLECQFQSKQAKEMKNHFPPLLLFFQRKNKIER

>arahy.Tifrunner.gnm2.ann1.IV9S0P.5

MGSKENIQLHVLPEEEAWDLFRDMAGVDTTDKANIKHIARKVAKECGGLPLAIVTTGSGLRNKAKPQWEYAFEQLQHSKLLNKVQSSIELSITSLGTEEHKYCLFLCALFPEDFDVPVECVLRHAVGMRLINLAGALGKARNMVDTLVDDLKRCFLLLESGKRDCVKIHDVVRDVVLSIASRKEHGFEVRQGNDELKQLKKQDKVDHFKALSLILADAITNGIDYPMLELFQARSEREEAILWPENFFQRMSKIRVLTMNKLRIPTMPSLFQTPVTLRTLQLEDCDVGDVSVIGNILVELEILSLSRSNIKELPTEIGQLSSLKLLDLTECNELAIISDNVFARLSQLEELYFRVSNFPWMLNKPILGELIELSQHLKVFEIRVREVEILPKDLSFKNIERFWIYVAQYVYWGDDRGFGYLEPNKLVFRHTSYKKSIKCSPVLMQLIKRCEVLKLEGVKDMKNVICELDDNGFQCLTNLELYSCPDVMYVVDCITATSYTAFPLLKSLSIMNLPMLKEICKASDNHHKVNNPLISEFLNLEKLYLERLPLFTGFSNAIDSTEPPSSTDSSHRGSSCTNKRVNASQTKEDESMSKSNCQNKLFSSIWMLQFPVLETITLEICHSLEEVFDLREYLKSSNTQMFPQLREINIYRLPKLKYVWGNVPHSVDGFHNLRSIQISSCDSLSHVFTPATVKAMVNLETLKISYCNSMVALVADEEEGDLETKGREHTIIFNKLCSLSLYRLPNFENLYTDSAKLEWPSLRTFYFRYCPKLKISLIPTQLKTENRDFSNSNSSSSSHRPLIGCMPWPLTFVRHRSQETTTSMEASLAQDQEEPAISEIKGKAEISHVPILEDLTLIHSDSVEEVVLLEGTHNSSIDICDYDNLEKRTSMMVTFTHLVSLQLFGLPNLENFCSFAINGKQEGRDSNEGSERILREKPFINGLLVPNLTSLHMSKCDRIKILFSFSTFGRLQNLRVSNCKNIEEIVSKDNINTSEDKVMFPKLEKLSLKSLPKLKAFCQVSYSFELSSLQEVMIKDCMNMEVFSRGSCHTTKLKNVTMNSKATYFKCNISVQKEDQLNSTIEGFRTFMELQEEKMIRWSDLHDKDIIKNLFEISVMNISGYHELSMLVQLKEMGMLRHVRELSITSCDSLEEVFEARGEMLTKEGYDQSINYGLESIKLQDLPKVRSIWGLNIVRHVSFTKLTSIEIARCNNLKCVMSYSVAKSLVEIKELEVKNCEMIEEIVKKEEEEKIMNMGAGCKDKTLFPKLEKLSLENLPNLRCFCYGDYDYDIPLSNENEDKKKQEQVQVSFPQLKEISLTGVPNFQCFCGGSYDYDLMLLSPSSQIETFSNGNGKVIVSTPNLHKVNNDTLTLGDMNLTLYYLHNYSGKYKVELQEVDTFECVDEKPQYKHLVGYMKRVSRLEVESCNKLLTCVQSNTIHSLLFQHLKELHVRQCHIMEVVFEGTGTGIHKSQLVRMELHSLPKLNHIWRNNNSVIGFENLNILIISRCHDLRFVFPNVSAARSLSNLEALDVQECKEMEEIIGNNNSNKSVQHKGAKIIFPNLYSIRLSKLPKLNSFCSISSFYFDLPYCDG

>arahy.Tifrunner.gnm2.ann1.IYM9J9.1

MAGALVGGAFLSGFINVVFDRLIKREFVNLVVGKNLHRQLVEKLKTALLAAEALVADAEQKQFGNELVRKWLDSLRDALYTADDLLERALIRAEIRNKARIRLPRFFLNLYDWKMETKIEDVVKRIEDLEKHKDTLGLKEIPTGSSSWRPPSTSLVKGKVFGRDADQQALIRMLNDKNDRNLSVISIVGMGGVGKTTLAQWLYNNKDLMDGVDLKAWICVSENFDVVETTKNVIKGISSGVCSLDSFDLLQQHLKDKLSKKKFFIVLDDVWSEDADKWHSFIAPFQHGRKGSTILLTTRMVNVGRIVQHYNSYTLNQLSDDDCWSIFADNASFPESNGSSELEGIGRKIVERCDGLPFAAEALGRLLRSERRVEEWNKILLSDIWEFPITDSKIVPALLISYYHLPAHLKHCFVYCSLYPKDYKLDKDELILLWMAEDLLQPPRRGQTLEEVGCECFDGLVSRLFFKQVENDDEKYFVMHDLKHDLATFLAGDLCCRFGEKEKMSILTRHLSYNHSIPEVTCSSSKIKYLRTLFYINDGSHIGKAPATLPCDILSKNKYLRVLSFGRIDIFPDSIDKLIQLRYLDLSWSDIEVLPESLCKLCNLQTLKLKNCFSLTMLPNGMCKLVNLRHLDIRGTPLKEMPKGMSKLKQLHILSKFVVGKKEDNGIQELGGLLNLHGSLEIERLENVVDANEARSARIIDKKHIEELLLKWSLSSGDDMVSNTHTDEQDILGGLQPHTGLKELTVEGFKGEIFPDWIGHSLYQNMTSVSLECCWNCCVLPSLGQLPSLKSLSIRSFDELKSIGKEFYKNEGHQHSSPIAPFPSLETLEFDDMSCWEEWQLPDSEAFPQLKSLQIRDCPMLKGDMLSQVLVRIVSSSLDVSRVHELKIKEDAERWDKKMRLDGDRLSISGFECVVECAFKARIIHHLTSLQEIQISYCSSVVSLGGNCLPKSLQKLKIFNCRQIELLQQQHKYDLVHLQIYQSCASLTSLSLDAFPNLENLEIEWCSNLESVSMSEPPQAALQHLTISNCPQFVSFPEEGLATPNLTHLNVSRCSKLEALPRGMNTLLPNLESLDIAGCPNICRWPEGGLPAKLKELRIGECKEQLKGLSWMGNLDNLTHLTISGHGSDNIIESYPEVGWLPRLPSVTTLHIQDFHNLETLECNQLLRLTSLQQLHISYCPKLKNMEGEKLPSSLQQLHISWCEKLENIAGEKLPPSLLLLQLDFCGLLGKHCKNKHQQIWSKISHIPTIQVACAHIEMKYILRFGNHIKIIIIQSKVHQKRCSFFNCCQSGYNAKTANQWWSLSQPSPSLMIPQKIEPLPALPSAVWAPVECLLQQHYIPLLQWVELFQLQHASQVRTLLFLLHQETPLEAPSYVPYLCHSQI

>arahy.Tifrunner.gnm2.ann1.J2JVS2.1

MIDSVVNFALDNLSRLLVSEVTLLSSVKDQIRSLNDELKFMNIFIKSSEGKREDPFVKEVVNQIRNVAYQAEDVVDTYVVNVNNQRSRNMLGKFFHSKDHVMMFHEVSDQINSIKRRIDEIYQNKSKYGIQQGDFESHNGNKEFAKDSLIAKRRNVEEEEVVGLVHDSDEVINHLARRGDSSRRVVCILGMGGLGKTTLARKIYNNKKIKSMFPCHVWGFVSNHYRAQELLLSLLKLLGLSSEEYKDLNDKEKMKRKVRECMSGKKYLVILDDIWNIQVWDELQEAFPDENNGSRILITTRIEDISHYTRAIFTYELPFLDESKSWELFCKKVFGKEKCPHELELPGKEMANACKGLPLAIIVLAGMVAKKERSPREWLKIKNNVSWYLVQEEEYRIVTNILKLSYDDLPQTLKPCFLYLGVYPEDYEINVRRLCQLWIAEGFIQKKEVGPSNSPEVEDIADMYLDKLVERSLVQVASRRTDGGVKTCRVHDLLRDLCISESRENKFMEVCTNLDAIKCNSRRMSLQYRGELRLTEDNQSSARSLLLFGENTYWENESEGWKQIKNGFKLARVLDMNQVRLCLSPRGLTTLIHLRFLKVTASSRTIGDEVLASICNLWNLETLYLWIKHSITLPNKIWKLKSLRHVYLNCDDIFRELTQIESIMQRMKMEEATKIFKGWSGGASMSKMKIGETKVENLQTLHNICLNARTASVLKKGMFPNLTKLTLRGEVTELPEAPEKEFLENSLQCLNKLRTLKLLGIAILPLDRNAYPTSLTKITISFGQLDARIIKTLGQLANLQILKLGGRGIYGDVDCVAGDFPQLRVLQCGADMVDGRWKVEKGAMPQIRYCNIPHIRD

>arahy.Tifrunner.gnm2.ann1.J2MEI5.1

MLCITSNFISTLSSSSTTQNRQRNSTTASAASFLIISKMDAFAGKIIEGIASLAVEEFRFIWNLKADQERMKRITTSIKAVLLDAEAKATNNQISNWLEQLKDVLYDADDLLDDFSTEALRSKMIKEMKTLFSWKNKMVYAHKMGRKMKEIRKRLDDIANDRHVLQLRDCSIEPPVSYRGRRQTYSFVGEDEVFGRQKEKLLIKSYLLGNNSVTSNLSVIPIVGIGGLGKTTLAQLVCNDKAVRNYFELIMWVCVSDEFDIRRIAQKMIGHNNNKDCEVEEVQQELRKKIERKKYLLVLDDVWSEDRELWLQLNSLLMEGAKGSMVIVTTRNQKVAKIMGTEPPLFLKGMDVETSWKLFCRVAFDREKEPNDLELVAIGRDIVKKCSGVPLAIRTIGSLLYLRNLGRSDWIYFRDVEFSKIDPQKDEIFAILKLSYDHLPSPLKNCFSYCSLFPKGFMFEKNTVIQLWVAEGFIRTTDDIRSAEDIGHEYFMNLLSMSLFEAATTNDCGDVTAYKMHDLIHDLSLLVVGREYAVVGEKEVNVGKKTRYLSYNIPLFSAATLSSYLLNGDKLRTFHVPRQPIHSSRDLVFSFGSLISLRCLRVLTLRALDITVIPKGIEELKHLRYIDLQGNANIQSLPSEITSLYNLQTLKLSNCSNLKELPADINKMMSLRHIELDSCVSLTCMPPGLEQLTGLQTLTLFVLGHGSKLSLSELSGLNNLRGKLKIKGLNSLRGIAASVESAKVLLQKPHLQELELQWQWQCEEEAISLVSDPLFNRGFKTTIQQEEDGLKDEVILEGLQPHQSIKRLVINGFCGKKLPDWIGNLTELAVLEINNCDGLTSLPEGLRNLKSLQRLCIYSCSQLEESCARNHGDEWNKISHIPKVLILPLPPSLLKYIN

>arahy.Tifrunner.gnm2.ann1.J4BTEJ.1

MKRIGRMYWKVKFGNFQKMTAKFYHYLPSHLKRCFVCCSLFPEDYLFDKDELILLWMAEGLLQPIGKSTLEKIGCAYFNELVARSFFQPSNTNGSLFVMHDLIHDLATVFAGEFYFSAKNHGNAPEISIKTRHLSYIAKPWHPISKLPEVGNGATHMRSFLSICLFVDYPLTRIKCDSGHFLLHLRFLRVLSFKCFTLKSLSDSIGELFHLHYLDLSFTLVVTLPESLCKLYNLQTLKLRNCIKLKMLPSRMHDLVNLRHLDIEGASCLKEMPKGFSKLKNLNFLNHYIVGKHEENGIRELGTLDSLQGSLCIAKLENINNSSEALEAKMGNKKHINILTFKWLPGSDIVDFQTSRDIFNKLQPHGDLNELSIVGYRGETFPDWLSLGCFSYGNMTKLSLSRCTNCQTPFKSLESLTFESLPSWREWLFSDEFDAFPQLKKLSIRECSVLTGDLPNHLLALEQLSIVECEQLACSLPRVPRLHQLDVKSVYHIRMEEPLHWVVIEGTQLSLGSVLKTYHSRDYLFTSLQELKIWDCPKLIFLEKLQHKSLTEINLYKCGSITSLPLAALPNLRTLTIDACKNIECVVVPMDVEDTLPCLRYLDIKYCPSFVSFLTISNCPEIDSFPEGGLPPSLTTLWIKNCQKLA

>arahy.Tifrunner.gnm2.ann1.J60GAE.1

MPSKSTSSSSSERRWIYDVFLSFRGTDTRTIFISHLYNSLTNAGIYVFKDNEELRKGGQVSSLLMQAIQVSRISIIVFSKNYANSKSCLKELQRIMDCRRSIGQVVVPVFYNVEPSHVRHQRGAFGRSFQDLKKTISKNKAQLWGTALRDAASLTGFHLNNSRNESEDINKIIETITESLDNTVLFVAQHPVGVQPRAQHVIKLLNYQKSKDVMLLGILGIGGIGKTTIAKAIYNQIHRHFESYCFLSNIRELWEQSTTSQVYLQERLLSDIYKTTKIKIHNIESGIFILQQRLRYKRALLILDDVDKIEQLKALCGSREWFGPGSRIIITTRDEHLLKLLQVDHISRMPEMDQDESIQHFSWHAFKQPCPGEDFDQLSRNVVAYCGGLPLALEVIGSFLFDKKVREWGSVVDKLKRIPNDQVQRKLRISFDSLNDDTEKEIFLDIAFFFIGMDRNDVIHILDEYDAAIGISVLVDRSLVTIDNNNKLGMHDLLRDMGREIVREKSPKEPEERSRLWLQNDVYHVLSNHMGTKAIEGLTLKLTNSVSLEAKMFKMMKRLRLLQLAGVQLDGNFEDLPKHLRWLQWHECNLKYIPSNSDQASLVAIELEFSNLKFVWKEAKFMEKLKVLNLNHSHNLTQTPDFSYLPNLEKLLLKDCSRLSTISDTIGHLKKILVINLEDCTSLGNLPRSFYKLKSLTTLIISGCSMIDKLEEDLEQMESLITLIADKTAITQVPFSIVRSKSIGYVSLCGYEGFSRDVFPSLIWSWMSPTSKYNLSSMVQASSSALIPLDLPKLRSLWVKCDSDLQLNGGVARILATLFATYNKELEATPTTSLVMENIKGSEIFDLSNQSHNSESLKCLIIEVGMKSHATNALKDKILQGLMTTTNEFSDKYWLTFGSKGSSVVFEVPQMNGCNLKAVTLRILYSSSSETILSECLKNVLIINYTKSTIQVYKQDTLASLEDEEGKTITSHIEPGNKVEILLVGYKFIVKRTTVYLIYDEPVDHYAEHYQADVIVSSDNYIISSDNNDMEIDECSSVSGGSHDRSKKKYSCFYWCWR

>arahy.Tifrunner.gnm2.ann1.JAA9B4.1

MAETLLEIVLEKLTPLILNEFAAFFGIREKAEELTLTLELIKAVLDDAEEKQWSNRPLKVWLQQLKNAMYVMDDILDQLSTQSSQPGCFSSLNPKKVIHRRELGKKLNKMIGRLDRIAQRRSNFDLRQVVRKRSSEVAEWRQTSSTIAVPQVYGRDEDKKRVVDFLLSSSQSSESLSVYAIVGLGGLGKTTLVQMVYNDQQVGNNFDLKIWMCVSEDFTRETILRSILKAMEVDKSEVMNLEEMEKKVKVLLQSKRYLLVLDDVWKRSQEMELGLTQDKWDKLRSVLSCGSKGSSILVSTRDEDVATIMGACQAHHLDRLSDDACWSLFKLRAFGADKEERAELVAIGKKIVKKCGGSPLAALALGAVMHSRSTKKEWLEVQKSELWSLPDENDIMPVLRLSYSCLTPTLKQCFAFCAVFPKDTEIEKQELIYLWMANGFISSRPNLEVEEVGNMVWNELYQKSLFQDVKSDDFSGKIYFKMHDLVHDLAQSISEQEYICLEEQNLNDSSRNPHHIVFHGIYKDQFKKRAFENAESLRTMYQLDSAPFPFNSRLVPTNHSLRVLCIYPTKIPSFGSLSCLRYLELRDLDIKSLPASICNLHRLEILKLIQLPFLSRLPKHLTRMQNLRHLVIDPCYSLSEMCPDIKKLCELRTLSKYIVKCKKGHSLKELRHLNLGGKLIIEGLGNIGSISEDEHANLKGKQDLRELTLSWSKSGDKTKLRVGAEEVLEALQPHSTLELLTIQDYEGLDWPTWMGNNNLVSLRLVYCGKCKRLPPVGKLPNLKKLVVQSGMKDVQYIQKDESYDGGEAMPFPALEELYLSYLPNVERLMERETTHMFPSLSKIEISDCPKLQLPCLPSVKDLTVSYCSNEQLKSISNLNSLNQLRLYRSDEVSCFPEGIMNNMTSLATLEIKYFRELKELPSDITKLTALSHLTIFDCEKLECLPEQGFEGLSSLQRLSINKCTSLGSLPDGVRHLTSLQYLSIVGCRKLKERCKEGTGEDWHKIAHVPHVDNWIWKGCETHLKFSVAIFKLQK

>arahy.Tifrunner.gnm2.ann1.JACZ02.4

MAEIAVSFVLDQLFPLLRDKARTLQGAHKEFEDVKDELESICAFLKDADTRAAKDNNALDDDGIKSWVRQLREAAFQIEDLVDEYLIYQQQWPSHSGIAASLCKAASLIKNLKRRHRLASDAQDIKSRVCGIKERSERYGFHCSSRESHSAKSHDTRMASLFMEEAEVVGVEAARDELIGWLVEGTTDRTVISVVGMGGLGKTTLAKKVYDDTNVIANFECRAWITVSQSYTIEGLLRNMLDQFYKERREAPVPAAVDLSRMDQESLIEEVRSYLQQKRYVVVFDDIWNVNFWGQIEFAVYDNKKGSRVMITTRSMNVAEFCKRSAFVNLHNLRPLSQEKSWELFCKKAFRFDLDGRCPEELADISFEIVKKCKGLPLAIMAIGGLLSTKEKKAIQWRKLSENLSLEFEKNPHLSGITKILALSYDDLPYNLKACFLYFGLYPEDYLVNSERLIWQWIAEGFVKHEKGKTLEQVAEQYLTELIHRSLVQASIEVDGKVESCQVHDLFRDLIVRNMEDLSFARFVTKEDQSPIVGITRRLAIANSPDDFIGSLSSSTRSLHVFRVEELSETFLKSIVKKCRLLRVLNFRDAPLGQVPENLGKLLHLRYLSLRNTDIRTLPKSIGKLQNLETLDLRQTRVREIPKEINKLKKLRHLIAYHLNFKVDFRMSWDWEKGVQMNGAVGSLTSLQTLCLVKANHGGVQLLSELGKLNQLRSLALSHMKREYGSVLHVSIREMHHLETLEITAIDENEVIDFPLVSSVPQLQHLRLRGKLEKLPDGIQQLWYLVRLSLSYSKLGDDPLEVLQDMPNLMHLLMYDAAYEGKSLHFREKRFLKLNELTLKNLYGLNSILIDKGSLPNLKRVKLENIPQMKKVPFGIRHLVNLEDLYFVDMQNELVESIDPIGGQQHWIIEHVPIVYVRHKVGPKFAVFDTRVIRHSRRKISDSPRGPFTTMALLLFLNALVNPSGYLISICGSLSYFLDFIFDEYLN

>arahy.Tifrunner.gnm2.ann1.JDC98L.1

MAARFVGEAFLSSALGTIYNMLISPLLVNFIQRKKLNRKLVEKLETVLKAAHSVINDAERRQIEEEAVKDWLDRLKDAVYDAEDILDEITTKAAIHKEDPGNSLSRYVNLHGDGEIVTKIEEIIAELESTVKEKDSLDLKEIPVEDMSWRIPSTSLVDVGKIYGRDKDREAIVNLLLDDTNDRGISVIPIVGMGGIGKTTLAQMVYNDDRVKQKFVVKAWVCDGQEEFDILKATKMVMEKVTSSSCNSTELNTIQESLKKVLAGKKLLVVLDDMWSNNYDAWISFLKPFKSSNRGVKILVTTRLDSTADMMKTTPSYHLSLLGDDHCWSVFANHACLNSAEPYIHSGLEVVGRKIVKKCNGLALAAQTLGGLLRARKEAADWEFILKSEIWELPKKNSGILPALRISYHYLPSHLKRCFVYCSLFPKDYRFKRDELVLLWMAEGLLQQPNSSGSTLEEVGYNYFNDLASRSFFQHSKADVNSFVMHDLMHDLAIFYGGKFFFRTFELKTSDQHDACPRHLSYDLRINDSFSKILDACDNLKIARTLLEINFDEWYYSPMEIDPCRLLAQLKHIRVLSFKFFPLDGLDSIGELIHLRHLDLSGSPVSVLPESLSKLYNLQTLKLNRCKVLEKLPTSMQDLVNLRHLDLSGALIMVLPESLSKLYNLQTLKLSRCERLKKLPTNMQNLVNLRHLHVEDTNLEEMPKGMSKLKDLQILSYYIVGKHEENGIGELGELVNLQGSFRIEKLENVVDSSEAWKARMVDKKYISHLCLKWSSGEDSDIVDSQIENDVLAKFEPHNDLKNLTIEGYRGSMFPDWVGQSSYHKMTKLELRGCRNCRELPSLGQLPSLMELRLSGFEMVKKIGGEFYKGDGTHQHQKTPFQSLKTLSINRMPCWEHWESYECDDDELFPSLEVLMILDCPKLRGDLPTSLPSLKNLWIDGCKQLGCYLPRAPIIRQLEIDGKQEARIQELPLSMMETLRVNGEQQVEYVFDAMTYTQSTSLRWLQISNCSSVISFPRDSLPPSLRDLHIIDCKNVEFPMQHQQHHWLEKLKLSPRE

>arahy.Tifrunner.gnm2.ann1.JFQB2B.1

MDCIIGFASSIARDLACGAVDELRYPCCFNNFVDDIQRKEDALIRTRNGVKSRVKHAKKQAIKNTEVFDAWLEGSSPLKNNIEDLLKKARTNKSCCFGLCPNWIWRYHLAKKLSKEKQEIEKCIEEGKEYIQFERVASLPPTLHLSAAKRLKFDSMQYPYEQLMEALKDDKVTTIGLYGMGGCGKTTMALELMRTAETEHLFDKVLFVPVSSKVEVRKIQDKIASMLQFEFPEDGDRERAQRLSQRLTLDDESTLVILDDVWQLLDFGEIGIPSGEDHGNCKVLITTRSGTVCGLMDCQRIIHLATLTDEEAWFLFQKQANISLDMDNNLKQLGRLISDECKGLPVAIAAMAGTLKGKAEDDWRVALDRLRRSIPVNIEKGLQNPYNCLRVSYNNLDTEEAKSLFLLCSVFPEDFNIPVETLIRIAIGLGIVGEVRSLEGARNEVSAAKNMLISSCLLSLSHDNDDGECVKMHDLVRNIALWIAKEENKMVKCALEKDVTFESNSIKYLWCKEVPIDLDCSSIEFLCIKTDLHISDEIFERMESLKVLSLCWEGTHRRALSIASFKSLLNLRCLFLEWWELGDISSIGCMKKLESLTLCLCSFFELPDVVTTQLTNLRLLHLSKCDIETDPLGVIGRQQLLEELYFYDYEWGFDIGEHAEFLKKFSVPQALRRYQIKLGPEFEDYEKEILSGHRTLVLSYFDTSNAAVVDLAKKADVLLLANIKAGGKNIIPDIFQIESGGAMNYRWIELWLRDSETIECLVDTRSHYRQVGALFSELRKLAIERMEHLGALYHGMPPAGLFKNLEQLYIYKCPVLTCLFVHAVARNLIQLEKLEIVSCDGLKYIMADDDTEVKEINREDDILVFSKLKQLRINGCSMLEYTIPVAFAQSLVQLESLEISRCGELKQVFGQSMHQGHNQSEPDIELLALEKLQLTSNSNMVSIFPQHCSATLPSLYEFYLWDCPKFDVAVNSSKANRWKDVRIIEKEFLTSKSLVINNSNMESIFNLDRVDTHGQPITLSLQHLRVLYLCRMKYICVGLENSFAFQHLKKLEIYGCEKVEVIFPVSALRYLPELERLEITECQALKQIIEEDVESKRSSNRFHRQPCFPKLAALFVNNCHNLKRLFCVSASNDLPNLKLLIIWGASELEELISDEIGKAKVELPKLKLAVLTYLSSLCQEIELPSLMLRVVHDCPKLSLTSSFPTLEEFEGKLSELNKDLEIDEFYRWKLVKMVGEIIKSDSRVEDPTKEVTTYFPVGAEDETRSTMEIHQSIKRDIGEGAASHNGKTVTLSNHSQSARPKSGQLVNSRHKSYSHPTNKNNDNRPVIVRDSEYDVPISFVQIGEEDNNDRKIAPSVSVLAEKHPVTEENLVEKVVADIEESLKMPLKDIASSEANCLRLLTALKFLSHLPLEDETLSSGLKAVIDSLHQEFPSILWSFKQAFAATHRFAVLEEKKRCIMEEPSYQNEKAYEMEETVKKGIIRLGKEIEDCEAEPSSLEEEKKQCIFESTRMKKESESVRRDIPQMVEYQRKAKQQLFGVNYKWSALCSQFQLNHMVARRPS

>arahy.Tifrunner.gnm2.ann1.JIJ5SX.1

MSSTASSSSQNNEPGLMKFTTSSYRNWIHDVYVSFTKEDSSTKELVSDLYARLREAGVRVFKDEMEPRSGDQILFKAIEDSRVSIVVFSRSYGDSTWWLKELEKIMECRSSKGQKVVPVFYGVDPSEVRDLTAQFRTAFAYRGPLREAARLPGFATDIRETGEAVDNIVEHITAFLCGEDNWFTVPNVEKLQSRVQDLIQVQLLNYHKVQILGIWGMAGIGKTTIATALYNQICYRFERRKFVDNISKRCEQHSMIFLQQELLSCILKTTEEATKETIKEAVEEAREETAEKTKINNIESGKLLLKKNLSNKKTLLVLDDVTELDQLEALCGSREWFGPGSTIIITTRDRNMLKGVDDIYTVKGMDYDESIELFCWHAFKNKEIPTDDYRKPIESLVDLCEGVPLALEVVGSHLYGKAIEEWESALEVLEEHPPLKWQRFRRVSFDSLRDDSLKKIFLDIVCFFVGMNRNHVVQILNGTGHNGEKALTALEDKCLITVDGNQLQMHPLIEEMGTDIVDEESRLTPKLTTYDVFLSFRGKDTRSTFTSHLHAALENAGFYVFKDDVQLPRGEWISISLLEAVKDSRLSIIVLSTNYAGSKWCLEELENIMQLQRTTAHAVVPIFYDVDPSEVRHQTGAFGQKFNDLLRSHPVEEDKEKSWRTSLRQVGSLSGIVVVNSRNESEDIKKVVEDVTHLLDKTELFVADYPVGVTSRVQNVVNLLMEQQSKDVLLLGICGMGGIGKTTTAKALYNQLSRKFDCKCFLLNIREFWEQDNGQVSLQDRLLSGIYKTTKIPIRSTESGKLELKNRLCHKKVLLVLDDIDKVEQMNALCGSHEWFGSGSCIIITTRDEHLVKMRGVEPIIYRLDEMDDSEAVELFSWHAFKQAYPDTNFSVLTKDVVAYSSGLPLALEVLGSYLCDREIEVWKNALEKLKRIPNHKIQEKLRISFDSLSDDTEKEIFLDIACFFIGMDRSDVIQILNDCGLFAEIGISVLRERSLVTVDSTNKLGMHNLLRDMGREIIRKESPDEPEKRSRLWLHEEVLHVLTEYKGTTTITGLALKSPRTNPISLSTKAFEEMNKLRLLQLEKVQLDGDFKYISRDLRWMSWHGFPLSHIPKSCCQKGLVAIELEYGNLKIAWKQAQLLNKLKILNLSHSHQLRETPDFSYLPNLEKLILEDCSSLSSVSHTIGHLNQLLLINLKDCKSLRSLPRSIYKLKSLKTLILSGCLMIDKLEEDMEQMESLTTLKAEDTAITQVPNALVRMKNIGYISLCGFEGLVRDVLPSIIWSWTYPTNNVSSLFQNTLYHLSSMISKLPKRSSLLLESGLQLEIPEDVVLKTLDATNCKITNLDENASVHDITEDENVTVFDSDDMETDMNENVSDFEDMAVDRNAVVSDMLTDKSIDIISNGGESRISRFSRLRSLVHVVLKARLFWFGFIAILIWITCSYSENQECSTSFQVCQHTPTFKEAEVIPNKQVVKLLEEESKRKSKLSYNRRVVEPPPESKNIEASATIKKSRPKSKGDMPLVKTLFASNPKDDELALIVDPISLITTQEDNDLLAELEQILSESYVLSTNKVASATSVSELQSLPLIAVIQKVQLLLDNEVEALVADNDIRKQLLDYLAHLVQMKSQVPTNIQKLVKEIKEFYEGFLGNFPPIQEVLDNHQRLIDTKNRLEGELEIAKAKQAHFSTSISKGKVRMNEMSKEINELEVKLKALYDKRDKLKSAVKICEVETININRKAVTWVKETEEVVSTLKASESAFKNAESSKHNYERKLSELKRALGKIKH

>arahy.Tifrunner.gnm2.ann1.JK0RBR.1

MERTLYFEAISVFLPFFLSFFFFFFIRCISEMAEALLGIVLDNLIPFVQSEFAAFFGIKEKAEELSRTLQLIKVVLDDAEEKQWSNRPLKVWLQQLKDAMYVMDDILDQLPTESSQLGCLSSLNPKNVMHRRELGHKLNEIIGRLDRIAQARTNFDLRQGVRESPSEVVEWRQTSSTIAVPQVYGRDEDKGRVVEFLLSPSRSSEFLSVYPIVGFGGLGKTTLVQLVYNDPEVGNNFDLKIWVCVSENFTMESILCSIVEAITNAKSERMTLDVMEKKVKELLQSKKYLLVLDDVWKRSREMELGLTQDKWDKLRSVLSCGSKGSSILVSTRDKHVATIMGTCQAHHLNRLSEDDCWLLFKLRAFGADKEEREELVAIGKEIVKKCGGSPLAALALGGVMQSRSTEKEWVEVQKSEVWSLPDENDIMPVLRLSYSCLTPTLKHCFAFCAIFPKDMEMVKQELIYLWMGNGFISSRPNLEVEEVGNMVWNELYQKSFFQDVRADDFSGKIYFKMHDLVHDLAQSISEQECICLEKQNLNDSSRNSHHILFHRIDKKQFKERAFEKAESLRTLYQLNSHEFPFSSTLIPTTHSLRVLCINDRKIPSLGSLTCLRYLELRRLDIKSLPASICNLRRLEILKLKSLENLRRLPKHLTRIQNLRHLVIHECRWLSHMFPDAHKLCHLRTLTVYIVKPEKGHSLAELRSLNLGGELRISGLGNVGSISEAENANLKGKQDLRQLMLSWSNSGKSKSVVGAKEVLEALQPHSTLKLLTIQGYEGTQWPTWMENNSATHNLVSLRLVDCGKCRHLPPVGKLPFLKKLVVSFMDDVRYIDMDESYDGVEAKAFPSLEELKVSYLPNMELLLKRETTHMFPSLSILYIYKCPKLQLPCLPSVKHLTVWHCSNEQLKSISNLSSLNELYLRHSDQVSCFPEGMMKNMTSLATLEIDYLSELKDLPSDITKLTALTHLSISNCGKLECLPEQGWEGLSSLRHLPIHNCEGLESLPDGVRHLTSLQSLTIVKCPVLEKRCEKGTGEDWHKIAHVPHITSSVVVSSAMAEDAKVAREAKLAQLLNQTFEHNNQISSTPKIHRVPLFWHTQNDIIWSHSSWQPKSEATRTTLEISMGELRSAGVIQNLLQNDEELAKFLNDIGHDLPTKMFNQLFMSDAVPISKKYIQVRRQIEKHYSASSKLALRYHNNSIFSLHYCCFGSSVSSLSGSSSGSSFFSSSSSSSSSGGSAGNGGGNGVFIGLN

>arahy.Tifrunner.gnm2.ann1.JKXA5P.1

MAIQAFLTHSLSPLSHEDTWKLFSGCSFKHGNPDEYPILTRIGMEIVRKCNGLPLAAKVLGSLVRYKEDVEEWESVSQSHIWELPSARSSILPALILSYCHLPPLLKRCFAYCSVFPKGHEIKKWDLIYLWMAEGILPQPDTEKRMEDIGDECFQELHFRSFFHESTLDKSHFMMHDLISDLAQFVAGDFCYMLGDNNTRRIKNWARYLSFSQDKYEALDNLDSFTECQQLRTFLPFRSSKSGSKYAFTRMVQDLLEKQNPLRVLSLSYYEITKFPVSIGNSLHLRYLNLANTTVECLPPDVCSWYNLETLILSGCRRLSKLPDNMFKLIDLRHLDISGSKVMEMPAEFGRLNSLQVLTDFIVSNGRGSKISELGSLLELRGALSIGNLQNVADAIEASNARLRSKKYLHELEFKWTTTAHNVDSETAVLNMLVPHQNVKRLKIQNFGGSMLPNWLGSSAFSRMVVLQLVDCKTCFSLPSLGQLPSLEKLSIAKMKGLQRIGPEFYGYVTEPFKTLKILRFEDMPNWEEWSSSKPGHAARFPSLEELHITRCPKFTGKLPDQLPSLVKLVITTCQSWNGRCIRFSSMVVLELVDCESCLSLPSLGQLPSLEKLYVSKMRGLQKLGLEFYGSITESFKSLKIMKFEDMPNWEEWSTGGAKQESRFRSLEELEISKCPKLIGKLPRRLPSLRKLVITACQALTSSMPWVPKLTTLELTGCDALESLSERMMEENECLETIIIRNCSSLTTVFREGLLPSSLRSLEIYECRNLELFVSPSSTHESHQYPALKRLHLRFCCSSLISFPMSLFTNLEELQVQGCSNLKKISSPPNCLPYLRKLELKDCSKLVLFPEGGLPAPKLESLSIRSCTELSPDTAWGLHAMTSLTSLYISGIPSLTSLENTGIQFLASLRTLEIEACDKLAFLPLDRLVHSLSHLTIRGCSLLKDKCERDGGEYRSLVSLVPYGVIED

>arahy.Tifrunner.gnm2.ann1.JT86R1.1

MVKLKIHDTIKILEALVTRKDLLGLTAAGATSREKPLQQQGWWPPSPAVLHSYLDGTFPKQCFVGRDDEIKSVLGKLSSATQEDGKHVKVINIVGKDGAGKTALSGVIYDSDNVTQLFERKAWITVPPKATVNTVAKKFLRNSRLLLV

>arahy.Tifrunner.gnm2.ann1.JUY39I.1

MAGALVGGAFLSGFINVVFDRFLTTDAVNLVLGKKLGPDLVQRLKTALLGAEALVADAELKQFGNPSVRKWLDSLRDAVYCAEDLLDTVLTKSPTQKEVRPFLNTINFFINRDREMVDNMERVVRRIEDLGKQKDNLGLEKIPTGSSSWRIPSSSLVRGKVYGREDDKKALIKMLNDNSEHNLSVIAIVGIGGVGKTTLAQSVYNNEEEFMKGFDLKAWVCISENFDIAETTKNVLKEISPHTQCLDHFNSLHHALKEKLLNKKFFIVLDDVWSDDGDRWINFLTPFQYGKKGSIVLLTTRGKNVALAVQNCHPYFLRGLSEDYCWSVFADNASFPQSNGNAALEGIGREIVKMCGGMPLAAETLGRLLRTKHDVEEWNKILMSDIWEFSVEKSKIIPALLISYFHLPPHLKRCFVYCALYPKDYKFKKDELILLWMAEDLLPPPKRGESLEEVGCECFDELTSRLFFTKLQIAHDYFVMHDLLHDLAIFLAGDFYCNSEELGKEEEIRMQTRHLCVNLNHRSSKLLNLISKVESLRTLLLFGDLSSPNCNIEEATCDILPKCKYLRVLSFDKLDVLPNSIGELIHLRYLDLSYTNIKTLPESLCNLYNLQTLKLYYCSKLTPMPSGLQNLVSLRHLDIRETSLEEMPRKMSNLNHLHILSYFVVGKHEDNGIQQLGGLVNLHGSLEIKKLENVVDVKEARSARIIDKKQIDSLLLEWCSGDAMVSNTQIERDILNNLQPHNGFRELTIDGYRGTIFSNWVGHSSYQNMTRVSLMCCKNCCMLPSLGQLSSLKSLLIEGFDQLRSIGNEFYKNEEDHHSSHIPPFPSLQSLEFFDMACWEEWNLPDSEAFPQLKILRISECPMLKEEILHEVFLRIISSLSDVSKVRELRIDDYFIGEGIGDMFLGGDTLTIRGCESVKESAFKAMISTNHISCLQEIHIAGYSSYVFLPGNCFPKYLQKLQIWRCRKLEFPEQQQQKYDLVELLIEESCDSLASLSLDVFPNLKNLEIRGCRNLESFSMSEPPHAALQHLTIEYCNKLVSFAGEGLAAPNLTHLELRYCNILEALPHDMNSLLPSLQSLDIYGFSDTWRLPEGGLPPNLKSLAVGICEQQMRDLSWMGNLDALTHLKISGYKCENIKSYPEVGSLPHLPSLTTLEIWLFNNLETLECNELLHLASLQQLHIYCCPNLENMEGEKLPPSLLLLQIYSCDLLGEHCRNKHQLIWPKISHIPTIQVEQFLNRDF

>arahy.Tifrunner.gnm2.ann1.JW22BI.1

MAEALVVGALVSASISLVLNRLISPEFVNSVVSKKLNCKLVERLKTALLAAKALAADAEQKQFGNELVREWLDSLRDALYTADDLLDRVFIKAQIRQKVRVRLPLRLNLSAWKMVTKINKVVKRIEDLQKLKDSLDLKEIPMGSSSWRTPSTSLERGTVYGRDDDEQALIKMLNDNNDHNLSVISIVGMGGVGKTTLAQCLYNSKDLMDGVDLNAWVCVSEHFDVIETTKNVIKGISSGVCSLDNFDLLQQDLKKKLSEKKFFIVLDDVWSEDADKWNSFITPFQHGKKGSTILLTTRKVNVGRIVQHYNSYILKQLSDDDCWSIFVDNASFPESNGSSELEEIGRKIVKRCDGLPLAAETLGRLLRSERRVEEWNRILSNDIWEFPMSNSKIVPALLISYYHLPAHLKRCFVYCSLYPKDYQFDKDELILLWMAEDLLRPPRKGETLEEVGCECFDDLASRLFFKQVKNDDEKYFVMHDLIHVLATFLAGDFYYRFSKELGEKEERNILTRHLSYTHSIPEKACSSSEIESLRTLLYVNDKPYIRGERATLPCDILSKNKYLRILSFDRLNIFPDSIGKLIQVRYLGLSRSDVEILPESLCNLCNLQILKLQDCSKLTMLPNGMCNLVNLRCLDIRGTPLKEMPKGMGKLKQLHILSNFVVGKQEDNGIQELGELLNLHGSLEIEKLENVVDGNEARSARIIDKKHIEELLLKWSVSSGDDDMVSNTHTDEQDILHGLQPHRVLKVLRIKGYKGKIFPDWLGRCSYSNMTRVSLLDCKNCCMLPSLGQLPSLKSLCIERFDQLKSIGKKFYKNEGHQHSSPIAPFASLTCHVGRSGSYLTQKPFLSLRALL

>arahy.Tifrunner.gnm2.ann1.JX3LEM.1

MPLIDQIFYRSCRVQIQTIIQFHAAAFGVQHIYMASTSFTSKRSCKYHVFLSFRGEDTRPGFISHLYAALTRKGITTFIDDTNLHKAIEESMFAIIVLSPNYASLTWCLDELQKILECKHKLDQHIEAVFYGVEPSDVRHQQGTFGEAFRKHEHRFGQESGKVRRWRDTLTQVAGYSGWRSKNQNEATLVENISQRIHKNLIPNLPSSMNKLVGINSRVKQVISYIGIGLNDVRYIGICGMGGIGKTTIARIVYEAIKSEFEVTYFLANVSETCEKNGIVQAQKELVDHINGSSRNCKNEYDGRRIIQASLFHKKVLLVLDDINEEKQLKNLAEEQDWFGSGSSIIITTRDMHLLKIHDAHGIYNVEGLGESEAFHLFHLKAFKQRKPAEEYLDLSKQAVKYCAGLPLALEVLGSHLYGRPIKDWHSALGKLKSLSHADIFDTLKISYNALDLMDKDIFLDIAYFFKGWPEVDVIKILEGCGYYVEIGILL

>arahy.Tifrunner.gnm2.ann1.JZL3JJ.1

MLEYQLPSDDFDALISITSDSGLNSLMLEYDNLYRNSPKTARMRLFIFPGNGPNSASTQPFPAKLKSKVNGGAIVPPRITPVKFQDLPPVNNFNTSDRVLDSDPKVNRPVEDSGSSRLNSLLPALQYDVSISFRGEDTRASFTSHLFKALSRKQIVTYTDDLLHEGDSITSLLLRAIEKSCLLLVVFSKNYASSKWYQLGSYSEAFKKQLQNNKKAEVQKWREAFTAAANLEGLDSHSYRDNIEFIQNIVKDILQKLIDHYPPNDSKSLVGISENLEKIESLLSESVEVRMIGICGIGGIGKTTLARIIFEKYSYTFEGSCFLENVRERSGKVSKQRNFIVLDDVSSLEQLDYLVQKLQWCGAGSKIIITARDKNVLVPTVETIYEMKILDSHESFKLFSLNAFNEDYQQIGYYELSWKAVGCCKGIPLALIALGSFLYSKSKTEWHSALQKLEKTPDPEI

>arahy.Tifrunner.gnm2.ann1.K0Q4EJ.1

MEIDEAKFLCSYGGEINPRGRNNKLAYVGGTNKLLYVDCRIDFTAMVAEISSLFDGACNGDYFFKYQVPGSDDLNALVSVTNDRDLHNMMLVFDQLYRDSPRLPRMRLFLFPNPNKPLDSVKPDSNGLSSNLWKHDVFISCRGKDTLTGFTSHLVKALNQKHIKTFIGDELHKGDCISDSLTRAIDNSSISIAILSENVVTSSWCVDELLKIMECGRKVIPVFYGVDPSDVRKQLVSFNEKFKSHLQSNINDLLKWMDALAKVADLEGWDSCSCRDDFELVEKIVKDVLRKLNNPCLPKDIKGVVGINKNLEKLELLLSSVPDEQTGIIGICGMAGIGKTTVVKKLFEKHSYRYEGSCFLEGVLERLEKYGQEIDELCNQLHSKLLEGKDFPNSMADTNSAKCCLCRQRSFIVLDDVGSSKQLEYLVGDLQCYGAGSRIIVITRNKSVLEKRVEKIYKMEVLDFQDSLTLFSLNAFNQNYPKTGYQELSWKAVTYCKGVPLNLKVLGSFLHSKSETEWDSALQKLEKNPNASIQNVLRLSYDGSDYQQNHIVLDIATFFKGEQKEQRIDDLNKKWGGESLVRNLSRILKIIAICGVLVIIVMFWKTARFYNLLSVEHVLFEDINKAN

>arahy.Tifrunner.gnm2.ann1.K1XC5T.1

MGRPKNPIWNEVTIIGESERERKWLCNRCGKIYAGGATRINEHLGNTKGKGNITLCPRRHDLLANEGVNNSMLPGSSNQVEAPNEFGLNNGVLAASSNQLEDPNRLDSLPEPGFELVDNPYHEEAEGHPNTISACFDGPIDQNDVAGPSNMVEGLSDSDEVLESDNSDFETNIEKLRELVDELTGWEEEVKEELQWLEYCGKKHKREEVDDWLKKAADFKKEAHEIFDSFPRDALQDLQMKTDDLLCRFNWLEDEIPFHYDEDPMHVYFAKMREFLRNNNVFLIGVRGMGGVGKTWLVTYFRNQIRRNKSFNFEDVFWVTVSQDFSILKLQNSLAKRIGEKLDDDDEIIDRAEILSSALEKIDRSVLILDDVWNYIDLQKVGIPLRINGIKLILTSRLKHVFRQMDCPTDNVIEMKPLLQYTDWELFLLNLGCDGRPANLSSEVENTAQSVVEMFGGLPLGIKVMARLMKGVNDDSNIWRYTQNKLEDSSMGEDMEEKVIKVLKRSYDHLQDKTIQNGFLQHALYSKVSDEVFIMNLVDEGLIQSTSSLEKILVQGKAILAKLESHSLIDWLDPGWCMHGLLREMASHIMKGRFMLRCGKRLDNIPEMQEWAPLLEKVSLMDNRIEEIPEGTSPECPQLSTLNFSKNMIRCIPDCFFYRIKALTLLDLSHNKKLTSLPNSLSSLRCLKSLLLKGCDALESVPPLGNLQELSRLVISGTSIKEAPDGLEVLTKLRWLDLSYNMKLVCVEWSVICGLTNVQYLNLGQTSITGEVEILHGMMSRLKCFVGSFQVAKNLADFVENILNRDGGLEYYQIGFRYPDVFWEHDFTGRKRKLIIVQDFEGYELLLPSDLDQLVIRNNKQWSGDLCGAFSFKAPSSLREIQVRDFPKLERLCCMSGRCSFCQHLQNLQSLDLYRLERLDAIWSEEDEFIGLPNLSQLKISYCDTIERLMGAAALAKFPKLEGIDVSSCKLMKEVFATGMSEDDPDHTITFPNSFKYLYLLNLPELVSVCGSIIVSESPPVVTAYCCRQLRQSHLRHKQSPSASESNIKIRY

>arahy.Tifrunner.gnm2.ann1.K3ZAF5.1

MNDSCNARKMLNTVILSLEIDSTDRVHMVGIHGIDGIGKTELALALALYNLITDNFKGVCFLENMREILHKIWERIQIIGVKEGISQIQRRLSRIKVLLVLDDVDEHEQWIAIAEKSDWFHLVSRIIITTQDKYLPTPHDNERTYKIQGLNKEGSLYLLYWKAFKTDIVNQRYENILTCGVNNLCFWTSIGFGSFNLFGKDLELQKSAMDLYEKVSFDALGEDVQSVFLDISCCFKDILQAHYGSCMKYYIGILVEKSLKKNDQFRITLHDLMEDLSKKYISKKNHQKCLESVADYGSTRI

>arahy.Tifrunner.gnm2.ann1.K48XAH.1

MAAALVGGAVLSSIFNVVFDRMSSPEVANWIKGKKLTQKLLERLKTTLYAVQAFLIDAEQKQIKERAVKDWLDSLKDAMYVADDLLDEVFTKAATQKDPGTFFSRFLNLQDREIANRMEEIIERIESIVKQKGTLGLREIPKENMSWRITTSLVERSSIYGREEDKEAIVKLLLDDDDTGDGDISVIPIVGMGGIGKTTLAQLVYNDEKVKENFDFRGWVCVSEEFDVIKVTKTIIEAITSTSCNLKDLNLLQHDLKERLSRQKFFVVLDDAWNEDYEDWSRLLKPFQNGAKGSKILITTRSKKVASVVQTISPYELSLLSDEDCWLVFSKHARLSTDSMENPTLKKVGKDIVKKCDGLPLAAQALGGLLRGNFDVEYWNHILKSELYPKDYEFDKHELILLWMAENFLQPAGRNTLEEVGEEYFDELVARSFLQPHSTEKNKFVMHDLVHDLAIMFAGEFYFRAEELQNAAEVDIKTRHLSHNAKGNYPISKLLGVCDRIKHTRTFLAINLNEWIPFNMENAPYILLSHLKYLRALSFNSFPLESVPDSIGELIHLRYLDLSETHIVTLPESLGNLYNLQTLKLYRCTNLKLLPVGMKDLVNLRHLDIRGTWSLQEMPKGMSKLKNLQFLSDYVVGKREENKITELGALADLHRSISIGKLENVVNSSEALEARMSDKDGIDSITLSWSSNEEENTVDSEMERDILDKLRPHTNLKGLFIYGYRGTTFPDWLGHSSYRNITTITLYGCRNCCMLPSLGQLPSLKHLEIGFFGSLGIVGAEFYFYENDESCVETPPFQKLETLFFESMPCWKEWRSMVLNAFPRLRELTIRKCPSLRGDLPNHLPSLQSLQIEKCDEVSCCVPRAPAINSLSISGKHLVGSVVEAITNTQLTCLTSLCISDCSSHIWFPVSAIPPSLRVLMIQGCRELEFQMDGQHHSLQKLYIGSSCDSVASFSLLDAFPNVKNVDIKYCGNMECIVVSRPLSSLRYLCITHCGSLKSVSTLWMAAPQLEQLSLVACPEIDLSDTGHPHRSPRYLCISYSEKLVTSVAFMNSQFHGITSLSIEGGYNESVSVKCLPKEVNALYILIQHIHGANNVRYNGKFKVIENAKWKLSWRLENGSLTATASKEVVRMP

>arahy.Tifrunner.gnm2.ann1.K4R7NK.1

MATDALLGILIGNLNTFVKKEIAALSGVDSQIQELSNNLQAIRALFQDAAEEQFKSHAIKDWLKKLSDAMHVLEDILEDCSMESNRLQSEGWLARFQPKTILFRHAISKRMKDMVKRFQRIDDDRRRFQLPLGVRQRQQEDDDQRLTGSAIPEHQMYGRDQDKHKIVDFFTEHASSIDGLSVYPIVGMAGLGKTTLARWVFNDERVIQHFDLRIWVCVTTNFNMMRILQSIVESSTGVNPNLSTLEAMQNKVQQVLLDKRCLLVLDDVWDNIKWEDLKSVLHYGGTKSVSILVTTRDQIVASVMETCPTHHLQPLPKDENWSLFTHYAFGPNKEQPAKLVEIGKEIVRRCVGNPLASKVVGSLLRNKREEKQWLNVLESKFWDIDAVMGALRISYFHLKPSSRQCFCFCALYPEDFQISKEQLIHLWMANGLIKSKGHLEVEDVGNQQWEELLQRSFFQEVSTDKFGNTTFKIHDLFLDLAHSIVGEEYKAYDDSASLTNLSRRVHHVSYFGLPELNQHTLKNIESLRTFIDLDPAISNLLDGFPALVLRKVQLCNSLRALRTRSSELSALKSLTHLRYLNIYNSYITKLPKCVSRLQKLQILKLEQCHFLTCLSKHILKLKDLRHLLIEGCRSLVEMPPKMGELKQLKTLNIFIVDSKAKHGLAELHDLQLGDRLHFKGLENVQSERDAREANLMSKRELSYLYLSWNSDSNSNSLCISPERVLEALEPPPNLKNLGINGYRGSQFPGWLRNTNIFSSLVNVILFDCNNCEQIPPLGKLPHLEGLYVCGMKDVKCIDEDSYDGVEEKVAFKSLKELTLIELPKLERIVRDEGVEMLPLVSKVTIPCSPNMKLPLLQSVEVLAIEGLKSDNEGVASFPEEIFLSLRYVKQLRISSFPKLKVLRQELGTLSSLQKLDIFGCDELESLAENVFQGLSSLRRLGIYHCPRLKSLSSAVEHLTCLESLRIMFCPELRTLPTNMNKLTALHDAFINGRVPEGLQCIPSLKILILDEVDSLPEWLGDMTSLQRLVIRLSPRIKSLPSSFRNLTNLRSLTIEKCAGLEQRCQRETGQDWPNIAHVPHVQLIPTQQLKHTCWEIAKFKWSLRRLNISKPPNFAFDEMVEDLHKQKQD

>arahy.Tifrunner.gnm2.ann1.K5EKT0.1

MLSKFVFMITTVVPRHRMASEIENVTASIRKLYEDKQALGLSPSSEQNVAERTCYDLRMGAHFIRDDELVGVDYAKKLLTEWLIHREARRTIISVVGEGGLGKTAIVRNLYNKHKEDFDCYAWITVSHSFKEEHLLTTIQTLYENDGKKYLVENREKEGDKCNLTQKLRDYLQGKSYMIVFDDVWEINFWECMEYALPDDTNIRSRIIITTRDRGIAEFCRRSAPVHIHELKPLPADDALKLFLLKTFQFDHHGCPEDLNELSQRFVKRCEGVPLAIVVISGLLSTKKKTISEWQKVYDSLRSKFSSDPHLRSFYLVLLESYHDLPYHLRLCLLYFGLFPQDYSIKCSTLIQLWVAEGFVNENEMFGAQTLEEAAEDYLAELIRRSLVKVSNVFVNGKVKSCRVHDLMHDFIVRKCEELNFCQVVSKQQFGFHEWTRRISIQNIDKSAFIGNDQSFVRVRSCISCGIEELSESVVKSLFSGFNLLVTLGLEDFPLDHLPEVVGNLLNLKYLSLRKTKIKTIPKSIGKLQKLQTLNLRDTQVRELPMEINKLVKLRHFLSYSFASKEHRQHLLGVRLNGGIGGLTDLQSLAMVDTSTANGDDIIQEMENMINMKKLGIVGISEVNGNGVCNAIKNMPNLCSLSIKAAKNCGFLALQALVDPPANLQRLYLNGTLQRLPEWIPKLKHLIKLSLIGSRLAEDPLPKLEGLPELLELHLDHYCWNSKVHFKCGWFEKLKELTLQRMNTLRTLKIDKGALPKLEVLRLGSCPQIIEGADAIQNLEALKNLYLIDMPIKFKDDIALNIPYNIDMPIQLSNDIALNNPKDIDMPRQFANDISTPLKENTTLDISPPLLPPPQLTPPCLPPRRGQLKVKVLQDLASAAIRSLSAA

>arahy.Tifrunner.gnm2.ann1.K6KWHM.1

MDCISGFASSISRDLVVGTTNQLRYPCCFNDFIEDLEQEEEKLITTRNSVEDRVKHARKQVIKNAEVVNAWLEKSNPLKDKAEDLIRKARTNRSCCFGYCPNWIWKYHLAKKLAKRRKEVEMCVQEGKMYIQYERIASLPSKQHFLENCLKFDSREGDYEELLEALKDDEVTTIGLYGMGGCGKTTMAMELMRTAEAENLFDKVLFVSVSNIVDVRKIQDRIASALQFQFPEGGESERAQRLRARLNLDDQRTLVILDDLWQSLDFGVIGIPSGQNHRNCKVLITTQYETVCSLMNCQRMIYLSTLTDEEAWELFQNQAQLSGSTSDNLKHLGRLISDECKGLPVAIAALASTLKGKAEDDWNVALVRLRNSIPVNIERGLKDPYKCFRVSYDNLDTKEAKSLFLLCSVFPEGYEIPVETLIRFAIGLGMVGEVWSYEEARSEVCAAKNKLISSCLLSDMGGGKRVKMHDLVRDVALCIASNDNKVIKCVLEKSAILESRSIRYLLCKEFPSELNCSSIELLCLETNSEVSDEMFRTMGRLQVLFLSCQGLGTSSLSTMSFKWLINLRCLWLKMWKLGDISSMGYMTKLESLTLLGCSFLELTEDVMTQLTKLRLLHLSECKMERNPFQVIGRRPSLEELYIDGETSKWDNGNGDQTEFFRNFRVPQALARYHIQLGHKFKGHGDKILSCRRTLILSCFDTSNASAVALAEKAEVLLLANIVGAAQNITPDIFQIEREGFMDYGWIQLWLCDSDEIKCLVENSNHPQQRLRNMFSRLRQLRLERMKKFGALYHGVPPSGLFEKLEHLYISKCPLKTRLFTPAIARGLQQLEKLEIFSCDELKHILEDEEISGQDHRVIFSKLKKLHIMGCQMLEYVIPVTFSQGLVQLESLDIDYCGELKYVFGECSTDGDTSHQNGINIEFPALQDTVAIEKDFLSWETICIHKSKVETIFSLEQAEIIEKPVRLQLRHLELSHLRQMKYICVGLKNFFVFQNLKTLEIKRCEKLEVTFPASVMRCLPELKHLKIIKCRELKLIIEEGDAENHRLSNCVPPQPCFPKLSELIITDCQNLESLFLVPESNDLSNLEVLIIVGAGKLKELIRCEERQSDQIRNVQVKLPKLKLLMLMSMSNLCQEIELSSAALCVIDECPKFPLTSSVATFKEFERKISELDIDLEDLEIAGIDQWKVLDRVREINKSDQIEEEEAEIKIVGKSSYFDIPSTSTSPLDIAVHKAHSHELMDGQSVSELSFTNQQKPLGEIRTVQIPQSIEGIEKTCVGEVPASLKPISPIFGSEVVAKPLYKIPSMNVEDKIKEGQKMQDVKDSIQEQEQPHLSDKQVVSNSNIEVHGDSSMITKLEAFKQCSDLDDAQIALLDEAIAVYPHLWKVVEDFSMRFQAWMLKTLVDILFFLRNESPASVTPQGKKDFQKLCDEAIQLGFDKSWIHEMHQRVMVMVKDTNNNNKVDHAQEQLGELLKKHDHLTEQLQSIKAEIVSLREFVDSHKRCFDFL

>arahy.Tifrunner.gnm2.ann1.KENS9L.1

MMRGKKVDQKLLQRLKTILNVVEAVLNDAEKKQITDSAVKRWLEDLQDAVYDADDLLDEVATKAATQKDPPGNFLSRFLNLQDREMVTRIEEIIARLEDIAKHKDILRLEKIAAKNMSGRIPSTSLVKKSDIFVGRDKERDAIVNLLLDDAYNGELSVIPIVGMGGIGKTTLAKLVYNDDKVQQKFNVKAWVCVGEEEFDVLKVTKIVIEKTCSPCYSNDLDTAQNHLKNGLAGKNFLVVLDDVWSSNRERWESFLTPFECGSEGGKILVTTRLDTVASVVKTKHNEAHNLSLLDEEQCWSVFANRAWGPTEFRDHSALEEIGRIIVKKCKGLPLAAQTVGGLLSGKDNEKDWNDVLNSEFWELSEEDSGILPALRISYYHLPSYLKRCFVYCSLYPKDFEFDRDELTLLWMAEGLLLEPKSGNTLEEIVSLNSRMQQSMIRKLATRFAKFFMKIMEACNRLKHVRTLLQLNLHKGHGTREGDRVAVPCDLLEQLKCLRVLSFKFFSDDENLLHRSIGELIHLRYLDLSYTSIVTLPESLCFLYNLQTLKLKLCRNLKKLPSNMQNLVKLHHLDIGGTDLEEMPKKMSKLKDLQFLSFYIAGKHEENGIGELGELPHLHGSFCIEKLENVKNSGEASNARMDEKIHLKKVRFVIPTLKKMYLTNYVLTKT

>arahy.Tifrunner.gnm2.ann1.KHQI9Q.1

MASTSSSSSTLSCKYHVFLSFRGEDTRSAFTSHLYAALTRKGITTFIDDTNLRKGDVISHELLTAIENSIFAIIVLSPNYASSSWCLDELQKILECKHKLGQHVEAVFYGVEPSDVRHQKGTFEEAFRKHEHRFGQESDKVRKWRDALTQVAGYSGWTSKNQNEAALVENISQSIHKKLIPNLPSSMNKLVGIDSRVEQVISHIGIGLNDVRYIGICGMGGIGKTTTARIVYEAIQSEFEVSYFLANVRETCEKNGIVQAQKELVGHINGSSSNLINEHDGRRIIRASLCHKKVLLVLDDINEEKQLKNLAEEQDWFGSGSRIIITTRDMHLLKIHDAYEIYNVEVLGESESFDLFHLKAFKQRKPAEEYLDLSKQAVKYCAGLPLALEVLGSHLCGRPVKDWHSALGKLKSIPHVDIFDTLKISYDGLDTMDKDIFLDIAYLFKGRSKDGVIKILEWCGYHAEIGISTLIDRSLLTIKNGILEMHDLVEKMGKHIVIQESPNDPSKRSRLRGYEDINPVLTRNKGTEATHSIVLDNMKVYEEQNEVHWRDLTFSNICQLKLLILDGVKAPILSYIPPSLRVLSWIECPMETLPFMDHYYELVEISVSYSSSIVQVWHGKKFLEKLKYLYLSYCHRLKQIPDFSEAPNLEILHVEWCGELNDFPSYLTCHKSLVKLILFCCSSLETLGSKLEMSSLEELDLSFCTSMRKLAEFGECMKHLSVLSLSETAIEELPTTVGCLVGLKELHLDGCKRLTCLPDSIQKLKSLTLLDLSGCPNVLQSLHFLSSLTSLDTLGLSGCFVTSQESWSFDLGNLASLTDLDLSYNDFIRVPINIHELPRLRCLDLDYCPNLKVLPELPSSIRELYARHSESLDTWHWNVISKVCCGFAASANHHSYGLLQMWVAQKQIKIFGLTIGEEIPLWFVHQEEGNGVTVTLPHNETMALALCFRLRPTNSRPKFGRDLSVICNGKEFIKQEHLTAACETKNSQHFILCLTSDYFVDQFCQDCRFQLVLPWDVKMKVESCGARWVCKQDIQDLKKSGTQTSKRKATFDLNF

>arahy.Tifrunner.gnm2.ann1.KNN53R.1

MVKNFLAMKRYLIVLDDLWKTQDWDDVKDAFPNDNNGSKILITSRLKEVALHMSSCPPYYLQFLGDDESWELFFRKVFQGKECPSDLEHLGKQMVKSCGGLPLSIVVLAGLLKKKEKSQREWSRVVGNVNWYLTRDETQVRDIVLKLSYHNLPRRLKPCFLYLGIYPEDCEISVRPLLQKWVAEGFIQQTGTRDVEDVAEDYLYELIDRSLVQASRVNVNGDVKACRIHDLLRDLCITESKEDKLFEVCTNSNILETSKPRRLSIQCGMDRYVSSSKNDHSCVRSLFCLDPTRYGFTPNEMKWLFKLFKLVRVLDLGKNYFLKVPSNLGLFIHLRYVRINLARGESGQVIPDSISTLENLQTLDIYGMPYPIYLPRRVWNLKQLRYLRSSGTIILRGYHGSKAGDQVMWNLQTISLIKFNSQIARMIENGRFPKLRKLGLHIHSVQKKNVHELLSTLRRLTHLNKLELSFRRKNDYGYMDWHIGLKAIELLQSLQHLSNLSTLKVDGALDLATCDIAFPACITKLTLTGISFMNDDGVNAIGNLTTLRLLKLHGAYKFDDPFEINCRAGSFSQLQDFEMEWVNVENWKLGNGAMPCLQTLLIKCCERLDDLPDELWSLTSLRQVPIL

>arahy.Tifrunner.gnm2.ann1.L0P1GP.1

MAGVLVGGAFLSQLIDVVLDRLMSADAINFVVGKKLSSDLVEKLRTALKDAGALVDDAELKQLDNHDVKEWLNCLRDALYTADDLLDRICTKAATQKVTLLGRIFNSEDRQMVNEIERVVRRIEDLEKRKGKLGLEKISTASFSWKTPSTSLVRGNVYGREDDEKALIKMLNDNNEHHLSVISIVGIGGVGKTTLAQWLYNNAELMEGFDRKAWVCISENFDIVETTKNIVKEISTNTQDLDSFNSIQDALKKELSEKKFFVVLDDVWSNDHHQWKDFLAPFQYGVKGSTILLTTRKEDVGSIVQTNYHRHYLNPLSEDYCWSVFAANASFPESNGSPILEGIGKKIARKCNGLPLAAETLGCLCRRHDAEEWEKILRSEIWRFSTNETKRFSKLYVMHDLLHDLAIFLAGDFYCRIEEPGEQEEMKVLTRHLSYLPRQSLGPPITKVSNSIAKLESLRTSLYINDLFSVESVLSKFKYLRVLSFWKFDELPDSIGELIHLRYLNLSCCEINRLPESLCNLYNLQTLILYRCTKLTMLPSGMHNLVNLRHLDLRETSLDEMPGGISKLEHLHTLSSFVVGNNEDNGIQELGGLSNLHGSLKIKKLENVVDVRQATSARIRDKKHIDSLWLEWSSGGDMVSNTQTEREILHSLEPHNGLKELTIWGYRGTIFPDWLGNPSYSKMTHVSLVCCMNCCMLPSLGQLPSLKSLDIQDFGQLSSIGTEFYKNEGNPSLQIAPSFPLLERLKFDKMACWEEWHLPDSKAFPQLKSLQIIDCPVLKGDTLYQGDMFKAMISSNHLSCLQEIEISECSSLISLSLDAFPTLKVLEIKWCSNLESVSMSEPPPKSLQNLRIMNCRKLEFLQQQHKYDVVNLYMFDSCDSLSSLSLDVFPNLKNLEISLCRNLESVSMSEAPHAALQRLSITFCSKLVSFAGEGLAAPNLTHLNLVFCSKLEALPLDMKSLLPSLQSLEIYGCPNICSLAEGGLPPNLKSLGMGISEQQMRDLSWMGNLHALTHLEIAGYDCNNIKSYPEVDSLPHLPSLTTLYIEYFDNLETLECNELLRLTSLQQLHIKYCNKLENMEGEKLPPSLLRLEVYSCDLLGKHCKNKHQLIWPKISHIPTIQVDF

>arahy.Tifrunner.gnm2.ann1.L22RAT.1

MVEDDGLHKLIASFESAAARGSALILTSTPHDDNPELFMIPASHVVLLDSLSTKNMGSIFSAHASSRQMNRPKLQKVAAQAGYEIIRNLGNLPLAARMIGSLVQDKLSVNKWVEMSRLLDAGDDIDVNLHPIPLFLALCYLDLPAQIKWCFAYLSLFPKGYQFKQTEVVLLWMAQGFLNMNTSGDKSMEDIADEYFGYLVMRSLLQPCSSGSGVSFTMHNLVHDLATYAFGESYKHHLSYSEDIEDFPEQSIVENRRTLRTILPLYLSLEQALTKFDIKLLEFVVKQLNPRVFRVLSLSRHYVTDLPASIGRLKHLCYLNISYTAVKTLPDSICDLLNLQELITTGCSSLKSLPERISNLVNLRHLDVRHSGLQEMPLGMHKLTSLRTLTDFVVSADGPGLADLAELSNLKTLSISKLQNVACAKDPSDAKLSKKILDDLMLQWGNGNGNKWRAMEVLENLKPHKDLKKLTLEYYDGPRFPNWLGDPSYRALQLVILRHCGDCNSLPTLGMLPFLKDLFIEGFTQVSSIGAEFSGEVRPSWKPFQSLESLQFRDMFQWRVWNILEGIEFPRLTKLYIIRCPKLVGYLPKQLVSLQKLEIIGCSNLVPPLPIVDVTCKVLVHESNEILMTSVARSSEGTLYSKDLHEIFLGSSSRFTITNPRCEIEEISLERSLETESLDSPSIPDSTMIQDLKEASTKMLSDQVKDDIPKGKVDILSTPHPDTPETMAPVKIENLSNQDSDDQRSSFEVLKVSTVSQLKSLPPTLHSLKIKGCESLEVLPDDLLAGLTALSEFYLISCSSLTSLPSLGSVITLYIRNCRRLENLSSLASRKQLAFLRHLSIGSSCDSLTTLTLDLFPELKVYGIVPIFSHFVLPKRSRVISHRLSP

>arahy.Tifrunner.gnm2.ann1.L48NEE.1

MAGALIGGAFLSGFINVVFDRFLTKDSVNLILGKKLGSDLVERLKISLHAAEALVGDAEYKQLDNPSVKDWLNCLRDAVYMADDLLNVVLMKAATPKEVRSFWPISFLNPDRDMVDKMEGVVRRIEFLEKQKDFLGLKVIPKDNNLAWRASTSLVEGNIYGREDDQQALLKIISDNSESQLSVIPIVGMGGVGKTTLAKWVYSVIEGFDLKAWICISETFDVADITKTIIEEVTRSPCTLQSLNSLQLELKRILSEKKFLIVLDDVWSDDADSWKKFKTPFHGGAKGSTMLLTTRIKEVASVVQTYPSYFLNELSEDYCWLVFADNSCFLKSNENLTLEEIGRKIVKKCKGLPLAAETLGRLLRGKEDVKEWNVVLTSDIWEFSMKNSKIVPALLISYFQLPAYLKRCFVYCSLYPKDYEFVKDELILLWIAEDLLRPLKKGETLEEVGRECFDELASRHFFKQHDHFYKMHDLLHDLAIFLAGDFYCRLELGNVEAMTTLTRHLSFESLSHLISENFGAIEYLRTFMRINFFSHLDNNDGVTFILMSKLKYLRVLSFSLFKGLDVLPNTIGELTYLRYLNLSKTSIKTLPESLCDLYNLQTLKLDECSSLTMLPNGMHRLVNLRHLCIWRTCLKEMPGGMSKLKQLHILHHFIVGKHEDNGIQELGGLLNIHGSFGIKNLENVVDVRQARSARILDKKHIDRLVLKWSSSDDMVSNTQTERDILGTLQPHNGLKILKIKGYRGTIFPNWVGHYSYQNITGVCLESCNNCCMLPSLGQLPSLKALRIEGFGRLKSVGMEFYKNEGDHHSSPIAPFPALETLEFWSMPCWEVWHLSDSETFPQLKSLQITNCPMLKGDMLNHLFLRIVSSLLDASKVHKLDISEDHEGWSRLVSLKGDTLSIKGCETVVESAFKAVSINRLTCIQEIRISHCSFAVSFPGNCLHKSLQKLTIWHCEKLEFPQQQQQKYDLVELQIHSSCDSLSSLSLDAFPNLKTLKISGCENLESVSISQPPHTALQHLSIHQCPKFVSFTGEGLAEPNLTHFEVNLCDKLEALPSDMNSLLPNLDSINSRKICRLPDGGLPPNLKQLIVNKQLRGLSSMGNFDTLTHLTIQGYVGVRSFPEVASLPHLPYLITLRLYWFPDLETLECNQLLCLNSLQKLKIGWCPKLENMTGEKLPPSLSLFKIKHCPLLGEHLKNKHQQLWPKISHILTIKVDDEQIF

>arahy.Tifrunner.gnm2.ann1.L4U9CB.1

MVVQKLNHKYTNELRSPFIRDQNYACIEALLKMQSREVQIIGIWGMGGIGKTTLAAAIFKEFSSKFEASCFLENVREESSNHGLNHIFKKLLSDLLEEKVHISTPKVISSAIISRLRRKKVFIVLDDVHTSELLETLLGVGHDYLGFGSKVIVTTRDKHVLQGRVIIHHIHGVKEMNFENSLKLFSLNAFNKHCPETEYLDLSMIAVAYASGIPLALKVLGSFLRSKSYHEWESALAKLKEVPNGDIQKVLRWSFHELDDTEKNIFLDIACFFKGQNRYKVTRLLNACGFFAEIGIRTLLDKALIRITINDSIQMHDLIQEMGHKIVHEESIKNPGGRSRLWKSEEGTDAIETIFLDMTQNTDELCISSQAFRKMPNLRLLAFADNKAFQRKKKRTNETLDLPTNLELPNNLRYIQWDGCPLKSLTTSWPSKLVQLSMPYSNVEKLWDGEQNLPSLEVINLECSRRMKECPDLSGCPNLKLVRLTGCNSLTHVHASVFSLQKLESLHVYACNALTTLSSEYCSPSLQSIVAYDCPNLQEFSVPMIGDHSGIHLHLRSTALKKLPSSIFLLKDLQHFSFPISESLMDLPTNFAFQISLSDPVEHKYDTAITLHKVLPSPVFQSVVRVVFDNCCSLTEIPESFSLLSSLAHLNLYRCIHVRTLPQSLKCLPRLEVLNIFQSDMLELLPMLPPSLKRLRVWDCKLLKTVLSTISEPTRKDEATFLFLNCNNLDEDSYGIILKDVIVRTELLITPPSSSGTEFENQEKERVFDCYTDFGEICYLLPVRGSNIHDLFDVHSTEEATYKLSVEVPRDSNLVGFLFFIVVSEEQWSCIEEHTWYNVSDLLGFGCECSLETCWGEKVHTESFSLLQWHWDQFYHHLNITSDHAFLWYDEKCCKKIMEPIRGRKGKDINDEKGSTSTCNNANLTLEFFAGVVNKLDAVIQECGIRWIYQNVEEEEGPRGRKSKRSIEESYEEEGSDDDEQEESSIPPTKKFKQSLLEPSLMLEGEEVVEDLR

>arahy.Tifrunner.gnm2.ann1.LD8LM0.1

MAESLLQMVIENLQAFVQDELATLWGVHSQIQELSGNLAAIHAVVQDAEEKQIRERAVKLWLQKLSDAAHVLDDILDECSIESNRLHSEQCLTRLDPVTIMFRRDIGKRMKEMVDRFRQIDEERRRFELRGRVPERQQEDEAWRQTCSGITEHNIYGREQDTENILEFLSRSADSSNDLSVYPIVGMGGLGKTTLVRWVYNDKKVIEHFHMRIWVCVSTEFNTMRILESIVESTSGHNPNLSTLEALKNKVQEILLGKRYLLVLDDMWSTDKWEDLKSVLLCGGGTKGAAVLVTTRVESVASVMGTCPAHRLSPLSEDDNWLLFKYHAFGSDKVERTELVAIGKEIVKKCGGSPLASKALGSLLRNKKEEIQWVNVLESKFWDILEDDAIIVRALKISYFNLKLSLRQCFAFCAIFPEDFRMEKEQLIHLWIANGLIKSKGRLEIEDVGNEAWEELCQRSFFQEVEIDELGRTTFKMHDLFHELAQSIMGEECRVYDESASLTNLSTRVHHVTCLKPEAEVNMDPFKKAESLRSMINLLPLDDHNLCGLPPFNSLRALRTNASQLSALKSLTHLRYLNLRRSGITTLPECVSRLQKLQILKLEDCLNLSCLPKHLTQLKDLRHLLIEECHSLVEMPPNIGELKCLRTLNLFIVDKKEGHGLSELRDLQLGGKLRIKGLENVLNEGDARDANLSAKKKLENLYLSWDSSDSRRVANAERILEALEPPSNLKSFGMNGYSGVELPSWMQNTSILSSLVMVILYDCKNCKHLPPLGKLPHLTVLYVSGMKDVKYIDDDSYDGVNQKAFKSLKYLTLLKLPNLEGMLRDERVEMLPVLSKLKVSCVPKIKLPLLPSLEYIWIEGTGSDSDHSDSEGMASILEAIGQNMQHVKTLRISGFPKLKALPHELSSLSSLQKLEIYGGDELESFSENVLQGLCSLQSLKIHSCKKLRSLSEGMGHLTRLESLDIMICPKLVTLPSSMNKLGSLRRVYIYSCDTLPEGLQHVPSLQSLEVYKSNSIPQWLGEITSLQKLELSCVRLRSLPSSFRNLRNLRELSIYGCHKELQKRCTRVTGQDWQAIAHIPQFKLVPIHEETFSDKIRSKWRSWQLRRDQRRHRFAKDDTFDYLVSRLFCWYKM

>arahy.Tifrunner.gnm2.ann1.LDQ9TP.2

MANADAGESSETNKSNYDIFLSFRGEDTRHTFTGYLYEALSRKGIKTFMDNENLRIGDSIGPNLLKTIEHSRISIVVFSENYAASKWCLDELVKILECRNEKNMLLFPIFYKVAPSDVRYQRNSYEKAMAAHELRYGCGSEKVKKWRSALFDVSQISGHVLREGSLKSLEFRVYGSRGTPEMAIRSAAWLPAIFLSRTIFAYYICRYEYKFIQDIVCKADAKLLSSKQLHIDEDMVGLQFKVEEVKSLLDLKSSDSTSMVGIYGIGGIGKTTLAKALYNTICNQFEGACFLFDVRKTSNQEKGQVHLQQTFLLELLEEGKIKFSSVEKGISILKDRLAGKKVLVVLDDVDNMEQLRALAGKCGWFGSGSRIIITTRNKYLLTAHQVKSIYEMKLLNVYESLELFCLNAFKMSSPATNYDDLSNRAIGYAKGLPLALKVIGSNLIGKDLNEWKSALKKYKRNPHRDIQSILRISYDSLESNEKDIFLDIACFFNGQRLEYVKRILDGCEFYTEDGLRILIDRSLITVEDGHLRMHDLIQDMGRDVVKQEAPKDAAERSRLWLREDVLQVLTENRGSSKIEGIKLDCLEENLISRAFEEMKKLRILIVRNTSFSFGRIHLPNQLRLLDWKGFPSKSFPPDFYPKKIAAFNLRCSPLVLKKPFQKFEHLTYMNFSYCQSITRIPDVSGAKNLTELILNKCKKLVKVDESVGFLPNLVYLSASGCIQLRSFLPRIFLPSLEFLSFDMCRRLAFFPDIVGTMDKPLKICMKDTAIRELPPSFANLSGLGYLDMSSCKQLQKLPSELFVLPNFVTLKIGGCPQLRESFTQFKGCDSRAECWPNLETLHFCNACLSDEDLYMIVHSFPKLIELNVSSNYFVSIPAYIKESINLTTLDVSYCLKLQQIPALPSSIQKVDARHCNSLTADSSSTLWSQMPREIDRLEVAMPETEIPEWLDYHGHGESPIFWARGKFPVVALAFVVDEINYQVVNLHLFIDNEHVTVQSQHLIFNIAEDHVLLFDLRVLFRVEEWKRLDARFQHDNCWKSIQVKCEPDIILKDWGVHVYKKETNMDDIQFSCPYPATLGSGSCMGLNLIWVPRDWVEEGSATGTDSSSDVEELDTILQVKEASCSSPCAVSCSCYCSAGSSSFKKLLGFKFRRRLSINMISRWPEDDVDDIDFRNQEPLSFSENLQTVMENLRRLAAPRQKKGMSNGDHDNDSLKWLAETEILSDGEYEDEEEESDSEA

>arahy.Tifrunner.gnm2.ann1.LF97TK.1

MEPGETPSVSTNASFISGIVFGLFISGILLNLRPLLGSKFRALLGYQDSDSNNIKDEPVGKEGSRLSQEDSASLHSLDSNVSQGSSLSQQEDSTCDHPPPESKSTMTEGSSSSPRGYKYDVYLSFRGADTRKSFIDHLYHRLMEEGILAFKDDVHLDIGEIISSQLLQAIKDSRISIVVFSRGYASSTWCLDDLVAIVDCHKEMKQELLPVFYDVDPRDVRYQSGAYEDAFDSHRERFKEEPDKIYKWKSAMTYLADLSGFALRDPAEAEGIERIVEAVRDTLVYKFQISRSTHKNPESTERRPSLQESNKYDVFLSLRRTHTPYTFIYYLFHYLTKKGFSTFKSEEETERGDSIPSQDLQVIKDSRILIVVFTRDYADSTCSLEEMATIVDCHRELNQTVIPVFCGVDRRDVQRQTGPYEKAFVSYTKEFKQDPLKVQKWREALKYMTNLQDISLKDWSEIEAIGNIIQIVERLCPKFSRLSSHSPVGIQSSLAMLEKLLKLEDDCVLVLGVWGMGGSGKTTHALALYDRIVQEFEGACFIEDVSQVYRFGGATALQKQVLCQFLNEELSDMYSPFEMSGFLRRRLYGKKILIVLENVDVPRQLESLAINRNMLGNGSRIIITTRDRHILTAYGVDEIYEIPLLNEDEARELFLSACPDVTNDEGYTELIPRVLEYAGGLPLAIRVLASFLRYRNVTFWEDTLNRSRRVPSFEIIKILQISFNGLDQEEKEIFLHIACFFHGKKLDFVKEVLNCCGYFAGIGIHALIDKSLIKNEEEEIHMHDLLQEMGKEIVRQECLGNPVSWSRLWRYEDFSFTLKSKNEASEVKAIVLYEDISISKHEDLNIEGLSKMTNLKMLILFHEHFSGKLTSLPNKLRYLLWDGYPFSSLPSFEPYNSLVQLNLPNSRIKRLWHGRQVV

>arahy.Tifrunner.gnm2.ann1.LFE0TK.1

FLLLSCVKIFVSLFFFFLVLSRSHSAEIEKIVKTVTKILSPRPSSSLSNDIVVGMEFSLQELGKLLVLDSDDDVRVVGICGMGGIGKTTLACRLYEKISHQYDVSCFMDDVSKTYRDCGSLGMKKQLLCPAFMEEDSPICNILMADNLIQNMLHYRKVLIVLDNVDQRIQLEKLALKREWLGRGSRIIIVSRDEHILREYGADHVYKVQLLNDENALQLFCRKSFKCNHVAKDYESLTDSALAYANGLPLAIKVLGSFLFGRDVSEWSSALVRLKETPTKDIMDVLRISFDGLEDDEKDIFLDIACFFPNDIEKYVKDILRIRGFHPDNGIKVLIEKSLITRDRWKITMHDLLRDLGRSIVREKSPKKPRKWSRLWNHKDLSNVLRENKDLHKLTHLELCYSKSLVKISNLSQAPNLSHINLKGCVKLVHLDTSIGGLEKLSLLNLENCKSLVSIPSNVFGLRSLCDLNLSGCSKLIGYRLLESEQSVASSICKALTRPLNFLSSRWRANSVGLLVPSLSHFPLVTLDISFCNLVKIPDAIGLLRCLEDLNIGGNNIVTLPHSINELPKLRRLNLQYCKQLKWLPSNLLPLGGTSGRVHYAGIYGAICVFNCPNLSDNVGCCLAVISWMIKMIQVNMQSSSPRCVIQVVIPGTEIPRWFNNQNEGSSMKLDPSPILNDNNRIIGIACCVTFVVHDTPTQLPEEPKHAGDIGCGFRLRSGGTYFALPIRLKKDFITTELDHMLLIFFSRDKIIDHYTSHLKEGRCALDGIELATSSEYPEEIIQVKSCGYRWAITSAKDLTGETTKFLRYEFKDSLNSAASLKMLLKVLNHIRGKRAKEWVPYYSNYPMECVRIQSTLSHSHRRNWKYDVFVSFRGETRFNFTDHLYAALRKHGIVAFRDDTKLEKGGPISAGLLEAIEGSQVLIVVFSINYASSTWCLQELASIADCIQIPGHTVLPLFYDVSPTEVRRQSRNFEKAFMKHEERFKDDAEMMEQVARWRGALTQVANLSGWDLKDKSLSAEIEKIVKAVTNILSPKPSSSLSNDVVGMYSHLEELEKLLVLDSHDDVRVLGICGMGGIGKSTLATILYEKISHQYDISCFMDDVSKTYADCGPLGVKKQLLCQAFMEEEFLLCNLSAATNLIQKYLCHRKVLIVLDNVDQGIQLENLALKREWLSRGSRIIIVSRDEHILREHGVDDVYKVQLLNDENALQLFCRKAFKCNHVIEGYESLTNSALTYANGLPLAIKVLGSFLFGRSISEWSSALVRLRENPTKDIMDVLRISYDGLEDMEKEMFLDIACFFSNEDEYYVKNILCIRGFHPDIGIRILIDKSLVTIVDGWKIVMHDLLRELGRSIVREKSPKEPRKWSRLWNQKDLSNVLRENKIAENLEAIVLPRFLEIREELSVEALSQMSCLKLLILREVNFSGCLNFLSNELGYLEWKEYPFTCLPPSFQPNKLVKLILHHSNIIELWEGIKDLHNLTHMELCYSKSLVKIPNLSQAPNLLRLDLKGCVKLVRLHPSIGCLKNLGYLNLENCRSLVSIPNNIFDLNSLYDLNLSGCWKLLKDQLSEKPRQSKQLNTGQSVQRHMTSSICKTLTRPLHFLSSRRCSNSIGLLVPSLSRFPALVSLDISFCNLVEIPDAIRQLCCLERLNIGGNNIVTLPHCIKELPKLRELNLQYCKQLKWLPSTLLPIGGGTEIPRWFNKQNTGNSMKVDPSPILNDNNWIGIACCVAFVVHDAPTEYPPILFGCGFRRNPDKAVCSVAPIRLKKDWITTESDHMLLMFLSREVFINNFVSVLKEGVSDLDGIELVALTRYPEEVVEVKSCGYRWVFKEDLEHLNPEKMYSSNSLAHNRKHKFLEIQDDQ

>arahy.Tifrunner.gnm2.ann1.LJ4C01.1

MGGLGKTTLARKIYNSKEVKKLFPYRAWSYVSKDYNKKEVLLRILDCLMSSTSKFKDSSEEVLKNEIAEGFIQPHESGTPNAPEPEDVGEDYLVELVDRSLIQVTSIRSYGGVKTCKIHDLLRELCILERKANNSLEVFTESNIHANNTSNPHRLSFLCNAQSYVSSVKPDTRSLSYEVPLSCVGGGFSRLQVLKLEYSSGYNRGVLDGGAIMTPLRCSVMKQCLRVTALPKQLWSLTTLQKVDVVGPSDELEKSL

>arahy.Tifrunner.gnm2.ann1.LM1XTI.1

MSSSCTIIYDVFISFRGEDTRETFTVHLFSALEDKTIRAYMDCLLQRGDEVWPALEKAIESSLISIVVFSENYATSKWCLEELVKIIQWREDHGQVVIPVFYRTDPSDIRNQSGSFEKAFAKYERDLAESESNRDKISKWRQALKKSANISGWHSRNYADDHELIRKVVDDVRKMRILKHPIVPTGLVGIEEIRKNVKFYMKQHRVIGIWGMVGIGKTTIAKMLFAKSFPHYDHACYAENAKEYTPQRLLSVLLKEEISTDVSGFMRSISSLSNKKVLIILDNVNDYDQPLLEAVCEGYKNHSRESKLIITTTHKHLLENRVDWIFEVQQWNDSKSRELLSLKAFEEKIPPKPYESLVNKVVRYAGGIPLALNLLGSYLRSKSIQFWESTLEKLEKHPDQRIHTAFRKIYDELEDLDKEIFLDIAFFFHGEKKDLVTSILKACDLSPIRGIEVLQDKALITTIPYKETIEMHGLLRKMAFEIVRKEEKDPRKRSRLRDTEDIRVVLKDNKESLDAVEGIILDLSKIKDLRLSPGTFKRMNRLRFLKLYIPSGQSSGKMILPAHLEPFSGKLRYFEWHQYPFNTLPPSFCAELLVEIHMPHSRVKKLWNEKQELYNLKGIDLSECRELEELPDLSEAISLKWVNLSGCETLPTLNSSVFSSNELVSLILDRCTNLQCVKAKRHLKSLQHISVKGCSNLNEFAVSSDLIENLDLSNTKVEKLDESIGQLQKLKCLNLEGSRVERLPKQLSTLKSLKELKLSYSELVIDKHQLQELFNGLSSLQILHLKDCSHLLEFPDNIDALSKLRELRLDGSNVTRLPATIKRLQELEILSLKDCRFLETLPELPSSVKEFSADNCISLESVSALNTLATKMVGKTKRISFNNSLKLSAGHTLHSIMESIHSTMVSAVSSNVTVRSYATEVHSYNYNSVEVCLPGDTIPEQFAYKTEKSSSITIELPDSPSNFLGFIYSVVLSPPHGMKMHGAKIRCEYNFAGGKNSSWEDITISELNSDIVYIWYDPFLTDKILGQYGTSFHLEFSVATDTREADDSITIKECGVQIINESELQRFLLELDKKKKDLEEESSKQQLHAPHGYQNHSDSHVQTPPQYLKKERLDEKQSNVLGAEEKMKFAPNENKTSGGESHEENNIVKGKGNVESPTESEAGPPLSIDSVNKNVSKDISIENYYDQPNGMEEVKDLKEKLQEVHHNMRDMTADEPYKEKQKEPNAEQTKNLGQQNIDAKKLQELHKEEMIDHAQPINSSIASHGVIMAPKLPLKELRRHLKAKVEANPERVTPATNLAMVTSLSSRSKIKGKRVEDIHLNQVPTENASTSIAQTHDKIAIPSLDDPQVALELLKHFDASGKRLQITEFVDDHVLYPYDHSITKDLDVSALCRWLQVQGLRSVSVARYAEMKFEAAKREREEELRLAREENAKFEEALKRMEERVSHVESLEKRVDDLNTEVASWRSKYEETEKSLKETQKKLEDEKDVGAQRESYWKRRESELITEAAIQLVWSFENCRSQVSILYPNIDLSRLGPFKEIQNGQIVSPSDTEETESEEDTSNFDNGGHDDARGD

>arahy.Tifrunner.gnm2.ann1.LT5Q2Z.1

MPITEYLQALLQYCSLCYILKIQSFVAAVCCDHHTMAEALVAGAFLSGFINVVFDRLISSEFVNLVVGKKLDRKLVEKLKTALLAAEALVADAEQKQFGNELVRKWLDSLRDALYTADDLLDRVCTKAEIRSKVRTHLPRFLNLSDRKIVTKIEELIERIEDLEKLKDTLGLKEIPTGSSSWRAPTTCLVKGNVYGRDGDQQALIKMLHDNNDHQLSIISIVGLGGVGKTTLAQWLYNNEDLMKGFDLKVWICVSENFDVIETTKTVIKGISSGVCSLDDYNSLQLDLKGKLSEKKFFIVLDDVWSNDSEKWNSFITPFQHGIKGSTVLLTTRTENIGPVVQNYSSYFLKGLSDDYCWSIFAENASFPESNGSSELEEIGRMIVKKCDGLPLAAETLGRLLRSKHDVEEWNKILSSDIWEFPVTVCKIIPALLISYYHLPAHLKRCFVYCSLYPKDHEFDKDELILLWMAEGLLRPPKRGETLEEVGRECFDDLASRLFFKQVVNDDESYFVMHDLMHDLATFLAGEFYCRLSEDIGEKKEMRILTRHMSYCYPISKKSSNKIEYLRTFLCIHYSPYFIEKASATLTHGILSKNKYLRVLCSHKLGIFPYSICKLIHLRYLNLSGSFIKTLPESLCKLYNLQTLKLEDCSELTMLPNGMYNLVNLRHLHIRNTPLKEMPKAMGKLKQLHILSNFVVGKHKDNGIQELGGLLNLRGSFEITKLENVVDVRQARNARIIDKKHIDELGLKWCSGDHIVLNTQTERDVLNSLQPHNCLKELTIEGYKGTAFPDWVGRSCYHNMTCVSLVSCKNCCVLPSLGQLPSLKSLRIERFDQLKCIGDEFYKNEGDHHSQPIALFPSLERLEFKAMPCWEVWHLPDSEAFPQLKWLEIGFCQMLQGDMLNHVLMRIVSSLSDASKVHKLEIREDHRGWSRGMSLNGDSLSIKGCESVVEFSFKAVISNNHLSCLQEIHITECLSIVTFSSDSLPKSLQKLRITQCRKLEFPEQQHQKYDLVELQIENSCESLTLLSLDAFPNLKNLDLDTCPNLESISMSEPPHTALQRLRIFGCDKLVLFAGEGLNAPNLTHFQARSCDKLGALPRDMNSLLPSLQSLDVRGCPNIASWPEGGLPPNLKKLKVGGCEQQLSSLSWMGNLDSLTHLTVFGFGLRCVSVNSYLELGSLPLLPSLTTLKISWFLNLETLKCNELLRLTSLQQLHISDCYMLENMAGEKLPPSLLLLQIKQNYLLGEKCKNKHQEIWHKISHIPTIVVDGKKIL

>arahy.Tifrunner.gnm2.ann1.LV2887.1

MRSFQVTSLLIGLKKLQNNSMKKVQKWREALTEAANLAGWVSSSCRDESELIQKIVKDVLQKLIDHNPPNDIKSFVGIDGNLKAIDSSLREVLEVQTRMIGIWDMGGIGKTTLAKLVFEKYSYQYEGSCFLENVRERSEKYGHALHELRNELYSELLQENNCKDSTRISTNAKERLRSQRNFIVLDDVSCSKQLKYLVGEHQCYGPGSKIIVTATDKSVFAQWEDEEIYEMKVLNSKDSLTLFSLNAFNQDHPKMGYEKLSWEAVKYCEGLPLALKVLGSFLRSKSETEWDSALQNIKKIPDAPIQNVLRLTYDALNYEEREIFLDIACFFKGFLKEHVVSLLESCGFDAANGMRSLHEKSLIAISDNSLRMHDLIHEMALEIVRKESIKNPEYRSRLWDYNDIRDVLGNNKGTDAIESIMLDMSHIDDLQLSVDTFKKMSRLRLLKLYDSSEKNGKLSNLQLPIGLKPLRYFEWHAYPLPTLPSNFCPEKLVTLRIQNSQLKRLWDGKQNLVDLEEVDLTGCQKLVELPDLSKAKNLKSVHLSNCRSLAHVHPSILSLGKLELLDLLNCIKLEMLGNKKHSRSLKHLCVCGCSNLIEFALSSEEIEYLDLSHTGIKVLHPSIGRFTKVKEISLCGIRLKNLPDGWSRLKSLEKLSLFKCGRVVSKQKLHDIFDGLQSLQNLSLVNCDSLFELPGNVGRLSSLQKLQLDGSHVETLPGSIKHLPNLKTLSLIGCKMLQYWPELPPSIRHLKALNCTLLQKVAFGLFSYELQEEKRVSISLQNCVNLDVENCIYSFLQHVRKQAYECEFRRRGVGRENIVVWRSDFFKICYPDSRVPEWFTYRAVGSSITFEVAPPSSYYFGSLLCIVLSSHSLDFELDIKCRCYLEDGKMHKYSLGILFLSHVPVEGHSDHVYMTYNFNGIFDVIKLDQLNNKIASSGHKPKLTFEFFVSSGMARGKKDLNLLIKECGVYPLNDSNNCVE

>arahy.Tifrunner.gnm2.ann1.LVW2ZC.1

MAGALVGGAFLSGFINVVFDRLLTMDTANLVLGKKLGPDLVERLKTALLGAEALVADAEMKQFGNPSVRKWLDSLCDAVYCAEDLLDTVLAKAATQKELSSSWWSPSFFTNREREMVEKMEAVVRRIEDLGKQKDFLGLEKIPTGSSSWRTPSTSLVRGNVYGREDDKKALVKMLNDNNEHHLSVIAIVGIGGVGKTTLAQWMYNNEEFMKEFDLKAWLENMEGEKLPPSLLLLKIHDCYLLKEHCKNKHQQIWPKISHIPTIEVDGQQIFRIEISEQVAYTEVGEYGKEKNFLPLCYYFKFKTVLCLENPARRVVAKLCMKGTEEIDESVFFIDSTMKMCIRQLLWPLQLGEHNFDIVETTKNVIKEISPDTQGLEHFNSLHRALKEKLLNKKFFIVLDDVWSDDGDKWSNFMTPFQQNGNKGSIVLLTTREENVASAVQNCQPYFLRKLSEDYCWSVFADNASFPQSNGRAALEEIGRKIVKKCDGLPLAAETLGRLLRTKHDVEEWNKILMSHIWGFSVEKSKIIPALLISYIHLPPYLKRCFVYCALYPKDYKFVKDKLILLWIAEDLLPPPKRGESLEELGCECFDELTSRLFFTKSEDFGDYFVMHDLLHDLAIFLAGDFYCNSEELGKEEIKIQTRHLYVDLSRCRSKLYNSISKVESLRTLLLLDDFSSPNSNTEVATCEILSKCKYLRVLSLDKHDVVPNSIGELIHLRYLDLSWTDIKTLPESFCNLCNLQILKLRRCYELTTLPSGFHNLVSLRHLDIRETSLEEMPGKMSKLNQLHVLSYFVVGKHEDNGIQELGGLVNLHGSIEIKKLENIIDVKEAKRAKIMDMKHIDELCLEWSSGDDLVSSTQRERDILDNLQPQNGLKELKIKGYKGTIFPDWLGNCSYENMTRVSLKFCKNCCMLPSLGQLPSLKSLSIGGFDQLRSIGEEFYKNEGDHHSSHIAPFPSLESLKFDNMACWEVWHLPDLKAFPQLKRLQIRNCRMLKEEMLNQVFFRIVSSLSDVSNVRKLHIDNNFIERHIFLDGDTLTIGGSESLMESALKAMMSINHLRCLQEIHIEGCRKLEFPQLQQHKYDLVELQIVDSCDSLTSLSLDVFPNLKNLELEGCMNLESVSLSEAPHAALQRATIFDCPELVSFAGEGLAAPNLTHLSITWCSKLEALPRDMNSRLLDLQSLEIYGCPNICRLPEGGLPPNLKSLDVGFCEKQMKNLSWMANLDALTHLTIEGHYCDNINSYPEVGSLPHLPSLTTLYIEYFDNLETLECNELFCLTSLQQLHIVYCSKLENMEGEKLPPSLLLLKIERCRLLGEHCKNKHQLILPKISYIPDIRVVASTEHKYDLVELLIHSCDSLTSLSLDVFPNLRNLEIYYCRNLESVSMSEAPHAALQCLSIRGCSKLVSFAGEGLAAPNLTHLQVAYCDKLEALPRDMKSLLPSLHTLEVYCCPNICRLAEGGLPPNLKSLDVGIGEQQMRDLSWMPNLHALAHLIINGLGCENIKSYPEVGSLPHLPSLMFLYKSSA

>arahy.Tifrunner.gnm2.ann1.LWFC3J.1

MAEALLGVVLEKLTPLILREFAAFFGIKEKAEELSRTLELIKAVLDDAEEKQWSNRPLKVWLQQLKDAMYVLDDILDQLPTEFSQLGCLSSLNPKNMIHQCHLRHKLNEIIGRLNGIAQARSNFDLRQGVRGRPVDEWRQTSSTIALPQVYGRDEDKKQVVEFLLSPSRMSEFLSVYPIVGLGGLGKTTLVQLVYNDPDIGNNFDLKIWVCVSENFTIKSILRSILEVTKKDKSEVMDIEVMEVKVKELLQSKKYLLVLDDVWKRSQEMELGLTQDKWDKLNSVLSCGSKGSSILVSTRDKQVATIMGTCQAHHLDRLSDDDCWSLFKLRAFGADKEERAELVAIGKEIVKKCGGSPLAALALGGLMQSRSTEMEWLEVQKSKLWSLPDENDIMRVLRLSYSSLTPTLKQCFAFCAIFPKDAEIMKQELIYLWMGNGFISSRPNLEVEEVGNMVWNELYQKSLFQDVRSDDSSGKIYFKMHDLVHDLAQSISEQECICLEKQNLNDSLRNSRHILFHRIDKKQFKERAFEKAESLRTLYQLNSHEFPFSSTLIPTNHSLRVLCINDRKTPSLGSLTCLRYLELRRLDIKSLPASICNLRRLEILKLTRLSKLRRLPKHLTRMQNLRHLVIHECDSLTGMFPDAHKLSHLRTLSVYIVKSKKGHSLAELHHLNLGGGLNIKGLENVGSISEAEDGYLKGKQDLRELSLSWGKSGKSKSKSIVEAEEVLEALQPHSTLKLLKIEGYEGMHWPTWMENNSDTHNLVSLGLEDCGKCGHLPPVGKLPFLKKLVVSGMDDVQYIEEDESYDNVEASRFPSLEELRVKRLRNVERLMKRETTHMFPSLSTIEITDCPKLQLPCLPSVKHLTIWNCSDEQLKSISNLNGLNQLRLYISEEVWCFPEGMMNNMTSLATLHIYSFRELKELPSDITKLTALSHLRISNCGKLECLPEQGWERLSSLRQLSIIECKSLGSLPDGVRHFTSIFDY

>arahy.Tifrunner.gnm2.ann1.M0V26Z.1

MGVCTKGISAICTGYETIFGETFYDNNNSGCDRQGDHQEESSELVAIGKEIIKKCGGSPLAALALGGAMNSRSTEKEWVEVQRSDLWSLPEENDIIRVLRLSYSCLSPTLKQCFGFCAIFPKDEEIMKQDLIHLWIANGFISSRLNLEVEEVGDMVWNELCQKSFFQYVRSDDSSGNIYFKMHDLVHDLAQSVSEKECIYLESEKQSLGDFSRNLHHIGFDLNRSQLKKRDFEKVESLRTLYQLNIDQPTPTNHSLRVLCTYGGKIPSFGSLTCLRYLELSWLRDIESLPASICNLRKLEILKLIGLWRLHHLPKHLTRMQNLRHLVIERCSFLSGMFPDAHKLRDLRTLSVYIVKSEEGHSLAELRDLNLGGKLNISGLENVGSISEAEDANLKAKQDLRELCLSWDRSSKTKSAVGAEEVLEALQPHSTLKLLTIHAYEGLHWPTWMGNNSATNNLISLHLYKCQNCHLPPVGKLPYLKKLVVSGMDDVQYIDEAESYDNVEATPLFRSLEELKVHELPNMERLLKRETTHMFPSLSKLEIEWCPKLQLPCLPSVKDLTVRKCSNEQLKSISNLNALNVLSLNLNDQVSCFPEGMMSNMTSLATLQIYFFRELKELPSDITKLTALSDLTISDCGKLECLPEQGLEGLSSLRKLSIHFCESLGSLPDGVRHLTSLELLSIGVCPMLKERCKQGTGEDWHKIAHVPHEMKLTSSSLLEFLQLSHPFFFMQNDIVVYY

>arahy.Tifrunner.gnm2.ann1.M159XN.1

MADSVVSSIVSFAVDNLSRLLVGEVKLLSSMEDDITSLRNDLKFMVAFLKNSEGNRNNESVKEMADQIRQVAFQSEDAIDTYVANVARHKSLNKLRMCFYCTGHVAILHELNAQVKAIKSTLKGIYRNKVMYGIDEHSGTTKGRSRWCGVSPDQRRRRDVEEREVVGLVHEFNVVMEKLMEPDPNLNVVSIIGIGGLGKTTLVRKIYNKDEVKSMFDCCAWKSSIDECKDLSVDELKKRLSECLKGKKYLVELDDIWKTQCGMS

>arahy.Tifrunner.gnm2.ann1.M2PLEH.1

MASSTNVVSNTITKITERFNTVRVSYHETMPIIDNITRHLSTINGMTDKCHGDDASLHIHRHDWITDIMDVLTNLWELLTNTTSLHPKQGTIATSFFSCFFSRIPRGLVLHLEQIEKSLENLAADSTKLSLKDRTKLRRSEARGNETKVQGRREDKEVIIGKILISTKVNGIVSVAAIVGMQGMGKTTLAKFVCDDDRVKDNFVVIWVDAGIHGEFYADSVKRSMIQELDPEKETVAIDENLDLGAAIHGRRFFLVMDDLRSENREEWVKLYEMLMKAATSSGGAVLVTTRNSHVANVVDPNAWRLFRLRKEDSWSLFENLAGGNSSASKIRGGHQKLEKKCKGVPLALVTMARMLESKSIAELKQDHLEEEFMQEMKSIYFNDLPSLHQKQCFAYLSLVFPTQFVSVKAETLIQLWMAEGFLGHVNLNSPSPQHSQPEDLGLDCIQEFSRTSFLVLHDSKIMTYKMANELIWELSRFVATKDRFCFCVDNRVETIKNTVSRVALSPSLNVTYGIPKFLTNSEKTLHTLLFPMPSYDWSSRIPYEVKLSWSACHELFRSFKCLRVLNLTDLGMKNLPDSIGELKSLRYLDLSHNNMKKLPKSIGKLKHLQTLILSHCHQLRKLPNEFQHLVNLRHLVMDECLQLEHVPLALKKLTSLLTLSHFTVSTRNNKSKHILGFRELVNLKNLSGELEISHLEQLKLKKSEQGLAYLKEKQHLKHLTLKWNHDDNDNHNNHDENDETSLGHLEPHVNLQGLDIVGYKGAKFSDWLQSLENLVTFSLYNCSSCKSPPPLDRFPKLKSLRLERLDSLEYINASQHELRLELLQELSIADCPKLKSWWQPGTEDTTAIFPSISNLKVRYCPKLECMPLYPNLDGNLLLEGSSMKPLMHTIDYSSSNMSSSTSSLPPLYKVKRLTITNVEGKEESPLPENWLESFISIHFLCISENMQRMRGFRHLTSLSTMIVTKCSRDDLPHDKQWQGLQSLRRLELQEVDKLKHFPEGVKHLTSLTKLSILSCSALTSLGEGIGELKSLEILLIKDCPKLGALLGIGKLESLKELSITDCRLLLPRCQRETGDDWPQIKHIKRIRLAGASEIYE

>arahy.Tifrunner.gnm2.ann1.M35DT6.1

MGGATAQQQELENIIEKVKTAQRCKQTRRILKSTLKGSKSLVYEIKQYNNDLDQPRDEIKALIKENGAAQDSESPCCNCFSSCSLWFGKCFFHIPFYRIRSVRSERNSYCDAKEMLSNVRDVLELLSRQNIEKRLSGGSIKRRPCGFPEKPGFTVGLDEPLRRLKVEVLMEGVSVIVLTGLGGSGKTTLATMLCFDEQVKDKFDENILFVTFSKNPKLKTIVERLFEQCGYQVPEFKSDNDAVNQMVVLLRHIGTRPTLLVLDDVWPGSEALVEKFQIQMSQYKILVTSRVAFPRFASHFILKPLNQEDALTLFRHYALLERSCSDIPNEDIVQKVVSGCKGSPLAIMVMARSLSRQPYGFWLKIVEKLSQGHSILDSNAELVNCLQKILEVLQNKPFIKECFMDLGLFPEDQRIPVTALIDIWKELYGLDDDGQEAMTIINILDSMNLVKLSVARKSVCGVDNYYYNNHFLVLHDLVRELAIYQSNQEPIERRQRLMIDVNENASQRWLGKKQKGMMMARISSYLTRCVKQKAVQVTVRTLSISTDEAYPSLLSNVKTDETEVLIVDLRTKQYSFPESIEKMNKLKVIIVTNYGFHPSELNNFEKLSSLSNLRRIRLERISAPPLVILKNLRKISLYMCNMTRVFQNGNIPISEAFPNLVELSIDYCKDMVKLPNELCDITPLKNISITNCHKFNALPQDIGKLSKLELLRLSCCTDLQGLPNSIGRLSILRLLDISNCMNLPDLPEDIGNLRSLRNLYMTGCSNCELPFSVANLENLKVICDEETAASWEAFIPIIPNLKVEVPQVDVNLNWLHPLSS

>arahy.Tifrunner.gnm2.ann1.M38AUV.1

MATLQESGNSSSTLKEWEFDVFLSFRGPETRYGFTGYLHKALCEKGIRTFMDLEDLISGNKIQQTFARAIESSRIAIVVFSEHYADTSFLLRELVKLLECSQRYGQFVLPIFYLVDPGDVRHQRGSYEKAMAVHEERFKDKAPIWRAALRDAANLSGLHYKGDEFEYEFIEKITKQVLSIIKEDMLPVVADYPVRPESQVQASFDSASSFSVSRQYQYHVFLSFRGCDTRYRFTGSLFKALRDNKIHTFMDDVGLHRGNDISRTLIEAIKGSRIAIIVFSENYADSTYCLDELVKILECHESDDQFVLPVFYEVDTNDVRRQTGSYGEAMAKHEEKFKDDKSKVEKWKRALHEAANFTGFCFKGKKYEHEFIAKIVGVVSKEIKRVALPVADYPIGLESQVSKVKSLIFSDGYDGVHMIGIHGNGGSGKTTIAHAIYHLIANGFESICFLENVRENSYKHGLVHLQDILLSNIFERKNLKSTSVEQGISNIKHWLQQKKILLILDDVDKIEQLQALAGKPDWFGRGTRVIITTRDKDLLVCHGIQRIYQLENSNPESNITASARKDNTEQVVEKIIGGQVNGSLEPLFSEDTNVDRSKQVSVIQAQSNAQVICQFNENQAEFCHTCSLERIESANITLKVKEELEVKDLSLCAKLASLEEKKRELEEQIRAVKAEISDSTAERQLLREKELAMEKS

>arahy.Tifrunner.gnm2.ann1.M4G710.1

MGDGASFRIPSGKQIKSVIFRHQKGSQKENSEYEDLLEKSNIVGAIHREKEEKEIIEILLKEPAGENGVPPVVAITGIAGIGKTTLACLVCENDKVKDRFGSPIWLNAAGKTFDDLASIAGPARNPSLLVLDDLRAEISKSKVSELRKHGVSVGAGAIIVTTRRTWQSFDDKAMHVLPLKGLDEEQSWSLFTSLGSSNKHKEEIKEILHNCLEVPPLATKLVSKFLESRAGTLEKDKLSKWLQFCLQPLAVQLVAYLSLFPDDYLIHADRLIHLWIAEGFVPPEKSCRQVFDDFVDSGIFQDVKREEDGDGVVKSCRDMQELACFVAKKKKDEFISTGVEEEPLLRASFDMRLADSVSKLELFLKEEKRAKDLKTILFHGMVESPLVLGNQERMNEYTCDKVLSTCKALCVLDLQHLGMNMVPSSIGKLKGLTYLDLSHNNTEKLPNSITKLSQLKTLKLSQCHLLKELPKDLKDLTKLTHLHIDGCVNIQSMPRGINKLTSLQTLSHFVVGKNEPMACEELRNLNELRGHLEIMYPERLKFQGSSSEWMNKKEHLRRLTFRLDQHDKGNEQDQKKALQALEPHTNLTELHVVGYKGNEFPSWIPSLDCLVKLSLYNCSTPEPLPWLDKLPSLEVLELRRMHSLRFVAEKCDDSVHPFFPSLKKLTLWDCPKLQSWWRNNNNTDGKSRNSIIFSCISSLQIHYCPNLTRMPLYPTLDEILELVDSSVEPMRDTIYYGESKMGFKEAKVVPFSKLKCMFIASIQKSPPELWLVNFTSLEKLHIRDCSQLEALPSGFKHLRSLQSLTIETCPVLDLDQSPDEWEGLEKLNCLIIKEIPKLKSLPMGLEKVKSLREIRLHDCAGLTSLPEAIGNLTSLVRLEICQCEHLASLPEGIKELICLNILIIKDCPLLMPRCQPDTGNDWPKIAHVKNIVVKQTSQRLWAGKLRM

>arahy.Tifrunner.gnm2.ann1.M55R6K.1

MAAVLVAGAFLNGFINTVFERLLTTDAVNFVLGKKLGSDLIQRLKTALLGAEALVADAEMKQFGNPSVMKWLDNLRDAVYCAEDLLDTVLIKATTQKKESSCCSLSFFINRHRDDMVDKMEGVVRRIEDLGKQKDFLRLEKIPTGSSSWRTPTSSIVRGNVYGREDDKKALVQMLKDNNEHHLSVIAIVGIGGVGKTTLAQWLYNNKDEFMKGFDLKAWVCVSEKFEVVETTRNVIKQLHGGTCSLDDFNSLQNALKRELSNKKFFLVLDDSDDGDKWSNFMTSFQQNGKKGSIVLLTTREENVALAVQNCRPYFLKGLSEDYGWSVFANNVSFLESNGRAALEEISRKIVKKCDGLPLAAETLGRLLRTKHDVEEWNKILMNDIWGFSVEKSKIILALLISYFHLSPHLKCCFVYCALFPKDYEFEKDELIPLWMAEDLLPTPKKGESLEEISCEYFDELTSRLFFTKNNDGDDYFVMHDLLHDLAIFLAGDFYCNLEELGKEEEIRIQPRHLCANLCYCSSKLHNLISQVKSLRTLLLFGYCSSSNCNIEAATWDILSKFKYLRALSFCKCELVHLCYLDLSWTNIETLPESLCKLSNLQTLKLYHYSNLTMLPSGLHNLLKLRHLDIRKAAFEEMPRKISELKELHVLSSFVVGKHEDSGIQELGGLENLHGPLEIKKLENIVDVEQAESAKIMDKKHIDELWLEWSSGEDMVSDTQREKVMLDTLQPQNGLKELKIKGYKGTIFPDWLG

>arahy.Tifrunner.gnm2.ann1.M6Z2HS.1

MDVKANLIISVDIINSDDAKKLFKKKVGITDDKNSDLEPLAIDIAKKCHGLPMSIVTTAKALKNQSRSVWEETLKTLERQKLTGTPEYSTKLSYQLLENEELKLIFLLCACMGQDAFVSDLVRLCIGLGFLEGVYTVREARDKVQMLLMHLKESGLLSNSYSNDRFTMQNLVRNAALLIAMEEKDVFVLTKRKLDEWPDDDKLKRYTSIFLHHCDVNAEEFPQSVICPKLKVFHFHNNHQHFEIPKDFFQEMKELRVLVLIGIDLSALSSSMECLTKLRKLCLEQCINLDEELCINIGKLMKNLRILSFSGSDIKSLPIELKHLSKLQILDLSNCSELKKIPPLLISSLTSLEELYMRNTSVEWRIDNRQNNKTKNASLSELGHLNQITNVDLQIPSVAHLPKNLFFDKLYSYKIVIGTSSTHLEPDFKMPEKYELLRYLAIQEKGGAFDIHSQKGIKMLFERVENLLLEELNGVQDIFYALNLKGFPCLKTLSIASSSGIRSLIKPQERKHPENAFPKLETLHLYKLNNMEQLSFCESLSPSSFRKLKVVKIKLCGLLKNVFLISMVKLLVVLETIEVSECNSLKEIVCVKKNASENLVFQELRTLTLKSLPEFHGFYPISSTAQQKILFDEKVGFSKLERLELSSIQIHQIWKGQNPPFAKLVHLEVNGCGNLENLLSLSMARNMLNLQSISVSECDKMRYIFFKEQGSSDTKKKQVTIFPNLKNIKVSSMKTLSGICNGFEFPIDSFVKLETLVIDECDKLGYVFTSHVGIFQHVSNLRVTNCKSIKAIFDPAWENKPASSKDATTNLQDVHLESLPKLEHVFKFNRKKNQLDGILSLNNLHKIWVQDCERLENIFSVPVAKTLENNLEDLVVSDCSQLREIVAKEEDVDRKISSLTAFNFLKLATIKFLRLPKFKSFYPGAEEIKFPALNNLSIEECEKLEPFREEIIDTQTKPILFPDMVINNLKSIQIESRHATSSTNYDYRRDNLEELHLSKLKDTKILYSFLHSNPNMKNLWLNDCSFEELVPLERLAKIESLGVVPQLKSLKLTDLPNLRRIGFERDPILQRIESLVFQNCSILKTIAPCNVFLSHLTKLEVVDCERLEYLMSPSTARSLGQLNTMKVINCESLKEIVSEKGQGRKEEDNKDNGDIIFKQLGTIELVSLKHLKSFCSSKNCAFQFPTLEKFVVSACPKLKSFSQQEGMNTPMLQKIYVVHEKEKMRSYWTNNLQQTIQYLFKKKMFFEGMEEVSVSDHLAHLQQLWQSKGVGQQENVFKKGLREELFNNLKTLKLSGCEFEPYAIPSNVLFTFKNLKELEVDNCEKITGIFEMNDTKMMGTSFQLKKLTLEALPNVKHVWPPKKKGILSFKNLQIVTVNECKDLRTLFPIALARDLKKLEELDVRDCDELSNIVEAEEAGTVDEHLVFPCLTTLALCYLPSLTDFCSQKFTLECPELNCFDVYDCNDQLELFQSHPEENQNNTSTTKQPLFMNTKDISKMENLTLNWRHTQALGSWLRKFKDENLESLNELYLLDDDGKSNCNVPVELFQKTPNLETLEVSYYCDETLKNILPSHDDEANNNKEILGKLKELYLFKLSELQSMSGVENLSKQLRLLDVSDCPKLTTIVLQSSSNLKELHIESCEALLRLFTSSTARMLIHLEELEVENCESLEEIVGEEQQNATTEDDVIEFKQLERITLRSLESLECFYSGNATLKLPSLIQLDIVDCSKMKVFSHGNVSVSRRIQVSYNSSDDLFFHHHLNAAAVWQFLSEERIVLSDVPDLQEIWAGKVPIPVNCPSCKFQYLEVDGCDFLSDAIIPSHLLPLLTNLEELVVRNCDSVEAIFDVRVKDTPTSDSSTVVIPLKKLTLEKLPILKHVWNNDPIKGGLNFPYLEEVTIDECKRIKNLFPASIAGDNLRELDVGNCEEMEEIVAKDANKELIKFHKLVKLMLFNLPKLRSVISSMMHIPEGCDISSNALLPSHLLSSLNDLKELLVIDCDSIEAIFDVKYSSPTQDTNMIDIPLKTIILENLPTLRHVWNEDPKGGFRLPLLNEVIVKECKCITSLFPASVFKNKVRKLDVRNCVKLEEIVGKNEAITAGKENDEELLMFPCLTTLTLWELPALRCIWSGRQILDWPELKELDVYRCPELKMFAADSENSPYSKAEDQDGIATNDRNDDANEGDNDDANDGDNDDDDDDDDDDDDDDDDDEDGDGDNNDNDDSVVSPSKIVAPRLQRLSLNKEDIIMIEQGQLQVDLQKILFLKLHSFHDDLHSFPDDVISKIQLTAIEQIAVVDSGFKQIFNSRRPENDTENYTKILSQLKGLVLRDLYKLESIGFEQTWMAPLLENLEYLKILACNCLMNLAPSTVSFSKLTQLNVKDCARLEYLFTSSTANSFAELKRMSISNCELLETVMVGDGDNDNEDVTIPKLSFIYLGNLPKLESFFKGKPNLNFPQLHKIFITECKGMQTFYQGNVVSPELQGVKDTEGEFCQCDDLNTAIKHYLRGEEDDNDSTKQEEEGEEEEEGSN

>arahy.Tifrunner.gnm2.ann1.M73JGF.1

MAAKLEGRAYLFCFVDAILNKLSSLDVNSTPTAKKLADQKLLQRLRNSLRATRLVLDDAEQKQIRDQEANKWLVDLQDALYLADDLLDELSTKAATATPPQRDPGNSSYHYRYAVDSVVEYSDGDEIRIVESMQDIVDKLESIVEEKDDLGLKQELVEDPKDMSWRIQSSLVESSDIYGRNDDKEAIIKLLLDDTCDDKLSVISIEGIGGIGKTTLAQLVYNDARVKEKFAIKAWVCVATRFDPISVSKAIIEEITSLCNMVNLNSLQTQLKEKLTQKTFLVVLDDVWDNQQNLWDNFLKPFLSGNKGSKILLTTRNKNVDSVVSNANLHYKLDILSPNDCWSLFLKHSSLSTNSRQYVILEKIGKKIVEKCKGLPLALKTLGGLLRNEENEEYWEKTLKSEIWELPKDKSEIIPALRVSYHYLPSHLKRCFVYCSLYPEDYQFDKDELILLWMAEDLVQPIENYTLENIGCAYFDELVARSFFQSSSKNVSLFVMHDLMHDLATFFAGKFYFSVREFGDCQKICSKTRHLSYMVNPRDPILRLEEAYKGAIHMRTFLDVHFSHQPYGSIKLESDSWLFHLHMRCLRVLSFKSFSIESLPDSIGELIHLHYLDLSYTPILTLPESLCKVYNLQTLKLRYCDKLAMLPSRIQELVNLRHLDIRGTYCLKEMPKGMSKLKHLNLLSDYIVGKHEENGIRELGALDNLHGSLCISKLENVNNSREALEAKMGKKKHVIILKLKWRREGDIVDVETARDILEELQPHRNLEELSIDGYRGEIFPDWLGLSSYSNVTILTLNRCKNCRQVPSLGQLPSLQYLEILGLEGLERIGGEFHNNAESSHQGTPFRSLLSLRFDKMHRWREWHILDEIDVFPKLENLSIRDCPVLSGDLPAHLPVLEQLSIVGCEKLACSLPRAPKLHQLTVKGSGFYRNAAPHEVLIKETQLAKSVLECLPHIQSPCFQDLEIHECWSAISISGDYLPDSLQFLRILDCSKLTFSEPLQHKSLTQIDVTRCGSLTLFPLGALPNLKKLSISKCRHLVSVPELGFAAPHLEELYMQDCPEMDCFGKECLPPSLTTLWINNCEKVERWITSKGLQSEGLTHLMLHKWNEASHYPDPEIQYTFLLWNCPSFKLRDQTPQVPKFVLLSDWLGCIPLKASSIEHGLQSFLLLKEVCCLT

>arahy.Tifrunner.gnm2.ann1.M76BEQ.1

MGLAAEPDSSNGDKTPLLLSHPLKRTAISIRTILVSICYHTNEEEVACEFVDAYYMLIFGAIQVVLSQIPNFHNIEWLSVVASIMSFAYSFIGMGLSILQIKEKGYVEGSIEGIRSRSRTEKLWLVAQALGDISYSYPFSTILLEIQDTLKSPPPENQTMKKASVISVAITTFFYLGCGGAGYAAFGNDTPGNLLTGFGSSKYYWLVNFANVYSQPLFAIVENWFRFQYPDSEFVNHSYFLKLLFLPDFELNFLRLSFRTAYVASTTVIALMFPYFNQILGVLGSLIFWPLTIYFPVEMYLGLSNTEAWSAKWIMLRSLSILGFVFVAVDSLIMKLARLLNEEVTLLRDVRTKVEGVRDELCLIQAYLKDADAKADTADTSSHAVKTIIQTLYENDGKKYPVEGIREKEVDKCNLTQKLRDYLQGKSYMIVFDDVWEINFWECMEFALPADTNIRSRIIITTRDRGIAEFCQRSAPVHIHELKPLPADDALKLFLLKTFQFDHHGCPQDLNDLSQRFVKRCKGVPLAIVVISGLLSTKKKTVSEWQKVYDSLRSKFSTDPHLRSFYLVLLESYHDLPYHLRLCLLYFGLFPQDYSVKCSTLIRLWVAEGFVNENEMFGDQTLEEAAEDYLAELIRRSLVKVSNVFVNGKVKSCRVHDLMHDFIVRKCEELNFCQVVSKQQFGFHEWTRRISIQNIDKSAFIGNDQSFVRVRSCLSCGIEELSESVVKSLFSGFNLLVTLGLEDFPLDHLPEVVGNLLNLKYLSLRKTKIKTIPKSIGKLQKLQTLNLRDTQVLELPMEINKLVKLRHLLSYSFGSKEHWQHLLGFRLNGGIGGLTDLQSLAMVDTSTANGDGIIREMENMINMKKLGIVELSEVNGSSVCNAIKNMPNLCSLSIKAAENCGFLVLQAIVDPPANLQRLYLNGPLQRLPEWIPKLKHLIKLRLIGSRLAEDPLPKLEGLPELLELHLDHYCESEEVHFKCGWFEKLKELTLQRMNTLRTLKIDKGALPKLEVLRLGSCPQIIEGADAIQNLEALKNLYLIDMPIQFSYDIALNNPYDIDMPRQFANDISTPLKENTTLYISPPLLPPPQLTPPRLPPRRGQVKVKVIQNHSTQCMYLTFLYIQIDVEKKGYVEGSIEGISSRSRTEKLWLVAQALGDISYSYPFSTILLEIQDTLKSPPPENQTMKKASVISVAITTFFYLGCGGAGYAAFGNDTPGNLLTGV

>arahy.Tifrunner.gnm2.ann1.M9HD5W.1

MAEIAVNLVVDKLIPLLKSEAKLLSGVRGQIESISNDLRLMRAYLRDVDAKAEMEAQNSDQSRKEWVAQIRQVSIRIQDVIDLYLYKVANNDDDDHHNPRRGRRNAVAGVLCKICDLLKSCVPRHEIASEIGEIRESINRLKEARQLYGDSNAAAESSGGRSSSPQRHYLRLQANFAEEEELVGIEHAKKELTNWLSEGAAGRTVIAVVGEGGLGKTTLVRNVYKQEQQKHSFDCYGWVDVSRSLKGVQLFKTLLRSFHDMKESNVDKNLHALIEDTREYLEGKKYLIVLDDVWETDLWGVVELALPKKNGMIMITTRYMGVADSCKVSAKVHTYPLKPLELKNALRLFHSKAFQSGSKNPSEELMKLSEEFTKKCDGVPLAIVAIASLLSTKKERVSEWKAVYNSLQSKLANDSHLKGYHQALSESYQDLSYHLKSCLLYFGLFPEQYAIKRARLINLWIAEGFVECREHQTQQEVGEEYLAELIARSLVKVADVNIYSRVRKCRVHDLMHDFIVKKCEEFNFCQVKKGNPFCFDKWSRRLSIGTNVDFADLRTSADRNQCSLLRSFLLYDIIDEEEMISTFVNSLFSNFKLLVTLHFENIPLGHLPETIGNFVHLKYLNLRDTNIETIPKSIGKLRNLETLDLKETNVSDLPVEIYSLTKLCYLNVRTKRREDVKLKRGVANLTALQKLAWVDASDVVEELKNLKQLRSLKIENVVRKDEMGLCNAIESMTNLCSLLIIAKDNEIIELESLTSPPRHLERLYLFGGYPEKKVPDWVFRLENLIKLELVRFKFTQDQLCFVGCHLPELMRLYVQSFEGDELNIQKGWFRKLQSLELGGPILKTIRIDEGSLPCLQHLALPVGTLKSKTVQVPGHVRREWIIQPLIPIISMMSCS

>arahy.Tifrunner.gnm2.ann1.MA81EW.2

MASKLEGGAYLSSFVDAVSKKLSSILEDDDFVLQGNHSARKLLEKLDDYLCDVEPVLEDAELKQFGNDRVKKWLVDLHDALYMADDFLDELSTKAATATPRDTGNSSPWSHSVDSTIEDNDVNIIEKIVGTLEFLVGRKDKLGLIKSAKLDTSWRIPSTSLVVSSEIFGRDEDKENITKLLLDDTCDAESPVTVIPIVGMGGIGKTTLAQLVYNDIKVVAKFETRAWVCVAENSDPVNVTRTIAGEIDFPPCNMDNFDSLQTDLKMKLTGKTFLVVLDDVWQDQRKTWEDFLKPFQHGSNGSKILLTTRSEKVASVFATNNLHYRLSLLLEEDCWSVFLKHSSISTNSKQYTTLEPIGRKIVEKCKGLPLAVKTLGGLLRNKYYEGDWENILESEIWELSEDDSKIVPALRVSYHYLPSHLKRCFVYCSLYPEDYEFDKDELILLWMTEDLLQAKGNNRLENIGCAYFDELVARSFFQPSSTNKKLFVMHDLIHDLAIFIAGKFHFHLNEWENLHMIDIKTRHLLVTEYKESLPDSIGELIHLRYLNLSRTPIVALSESICKLYNLQTLKLRNCTKLEMLPSCMHDLVNLRHLDIRGASCLKEMPKRMSKLKHLNFLSYYIVGTQEENGIRELGTLDNLHGSFCISKLENVKNSGEALEAKMGNKKHINTLKLKWVPDGDIDDVQTERDILDKLQPHPNLEKLSIEGYRGETFPDWLGLSCYSNMTKLSLDRCMNCYELPSLGQLPSLQHLEFSDLDGLEKIGLEFYNKNNASFQQETPFKCLETLKIVNMSRWREWHFPDEFDGFPKLRILSIKSCPVLKGDLPAHLPALEELTIVECEELACSLPRAPNLHQIHVKGNRSSTYSTRGHKVVIEETQLAKSVMECLPHIQPALIQHLEISNVWSAISISEDYLPASLQSLEIFYCSKLTFSEQLQHKSLTKISVVGCHSLKLLPLWDFPNLKNLKVSVCQSMEYVEVPHALPSLRYMCISGCPSLASLPALGLAAPHLEELDIRNCPKIDCFVEECLPPSLEKVVVIGCEKLARWITSKGLQSEGLTHLWLGGCFDVNSFPREGCLPSSLESLELWNFPNLETLDSKGLHHLASLKRLAIGDCRKLENVTEEHLLASIANIYIGEECPLRRKLEEMEHPPIQFVCYKCDSDYSDDDYCYD

>arahy.Tifrunner.gnm2.ann1.MGS08G.1

DGSAQDIPNNYTEPPFSYSSFYPTNSFALHSNPSSPMSYYQSAERIPDPSWKYDVYLSFRGQDTRYRFISDLYEALTRAGILALLDDEEIQLGDQISYILVQAIEASRLFVVVFSESYAASTWCLKELTKIMECRSTRGQTVIPLYYNVDPSDVRHQRGPFEKALLRHMDRYENEIVHAWRAALTQATELSGFLITGDSNEVELVGVIVEHITTLLASKDLFIARHPVGVQSRTEYVIQMLHSRKPEDVVLMGIWGMGGVGKTTIAKSVYNQIHRHFDRPMFLPNIWEMWENNQQVFLQDRLLNGICKAQINVQNIASGVALLRERLRLERVFLVLDDVNNIEQLNALCGGREWFGAGSIIIITTRDRRLLRMIGVDYVYQVKEMDYNESLQLFCWNAFGKATPLTEFARLAEDVVAYCGGLPLALETIGCQLYGKMKDEWENVLGGLKRFPHPDVHQVLKLSFDGLNDDREKEIFLDIASFCIGMERGEVLKTLNYGFGISEHGISFLEEQSLITFDEKDRVRMHPLLRDMGREIVREQSQTDAQRRTYDVFVSFRGKDTRPTFTSHLHTSLRNAGIAAYKDDDDVDGLQRGERISIALLKAIGLSACSVIVFSTHYADSKWCLQELENIMVCHRTKGQVVYPIFYGVDPSDIRYQKKETDFGKAFESLISRRSVEEDKVQSWRTDLREVSSFPGTTVINSWNESEDIKRIVEHVTHKTELFVAAHPVGVEPRVQKLIEESNIQKTKDVLILGIKGMGGVGKTTIAKAIYNKIRRDFDGRSFLLNIRETWKQDNGKVLLQQQLLEDIFIATSIHIRNIDSGMQILKERLSAKRVLIVLDDVTDLDQLNALCGSGEWCGPGSVVIITTRYNDLLRVCKLTFNLEKMNRAESLELFSWHAFKQACPKEEFVKLSRDVVEYSNGLPLALEVLGSHLFNMGIKEWESALDTLKSIPHDKVQKKLQISFDGLSKNYEKEIFLDIACFFIGMDRNDVVKILNGCGLRAEIGISALKERNLITVDNNNMLGMHDLLRDMGRAIICEKSPELEERSRLWYDEKVLELLENHEGTDLKVKGLSLKLPMTNSICLSKEAFKNMTKLKLLQLAGVQLNGEFKHVSRYLRWMSWHGFPLRHTPIDCYQPNIISIELENSKLRVLWKRSLLLKQLKILNLSHSHDLIKTPDFSYLPNLEKLVLKDCTELSLISYTIGTLKNILHINIEGCTNLSVLPRSIYKLTSLETLILSGCSKIDKLEEDVEQMESLAVLKADNTAIAQVPGALARLRNLGHISLCGYEGLARDVIPLIMFWLWTSPNNMLSSVMQKCSSNVFTYANWGPQLQIKGDVSLDTGNVSNCNELVMHISESKSSLNSLLTQMGVDNSFTEILQKTISQRMIYQYAIAQDEFTKNENLIMDVSGNFLTRAEAAAAAPGSWIVNNESLASKFEEFVAGEYIHLNSIIGDNPYWKENSIRDQNLLMGAILWKHHWWCYALDRSQKKLYVLDSIHCEPPNEDRRKLDKLVTYQSRATVSQSRLPFTEVDVSPTERTKKWFEEGDTSFKKKGKISSSKLFVNLVPESKRFKNSTKIFKPKSKGFIPPGQSPFVSNTEEVEPVRVEETNGSELLANLPEDVDVLLSPTKSASDGSGFESQSLSVIDILYKIQQLFDNQLQVLVADGGIRRQFVHFLAQLEQMKSQVPTNLKPLVNDIKKFYEDVLNYFPSIQKVFGNHQRLIESKNQLQEKLETAKSRQAHFYSSISKGKERINGMSNEINDLELKLKALYEKKDKLESTVKLCEVETLSINTKAATWVKESEEVDSKLKASESAFRNAESSKQNYESKLIELKRALGNIKH

>arahy.Tifrunner.gnm2.ann1.MR6FSP.1

MAGALVGGAFLSGFINVVFDRFLSSEAANLIIGKKLGPDLVERLRISLHAAEALVDDAEYKQLDNPSVKDWLNSLREVVYVADDLLDSVLTIEATRKEVRSFWPISFLNRDREIVDKMEDVVRRINFLEKQKDFLGLEKTTKKNFLSWRIPSTSLVEGNIFGREKEQDEIIKIINDNREHQLSVIPIVGIGGVGKTTLAQWLYNNDKLMEGFQVKEWVCISEHFDIAQVTKNIIGQNARCIDDFNSLQLELKEKLSAKKFFIVLDDVWSDDGDVWKKFKTPFQYGAKGSTILVTTRVKEVASVVQTCPPYVLSELSEDCCWSVFANNACFPESNGSSILEEIGRKIVKKCKGLPLAVETLGRMLRTKHNVKEWEDVLISDIWEFSVKNSKIIPALLISYFHLPAHLKRCFVHCSLYPKDYDFDKAELILLWMAEDLLRPPKRGESLEEVGCECFDELASRLFFKQHKDSSKNFVMHDLLHDLALFLAGDFYCRFKELGDAEHICPQTRHLSYESLSHLISNDFDSISKVESLRTFLPTKIFSCSDNIDCVTCILISKLKYLRVLSFLWFTKLDVLPDSIGELTYLRYLDLSRTSIKTLPDSLCDLYNLQTLKLDDCSSLTMLPNSMHKLVNLQHLYIKRTSLKEMPGRMSKLKKLHILSNFVVGKHEVNGIQELGGLLNLHGSFEIKKLENVADVKEARSARITDKKNIDKLQLEWSSSDDMVSNTQIERDILHSLQPHKGLKALTITGYKGTTFPNWVGNRSYNNMTRVSLVSCTNCCMLPSLGQLPSLKSLRIGGFDQLKSIGMEFYKNEDDHHSLHVAPFLSLETLEFSDMPSWEVWHLTNSETFPQLRKLEITDCPMLKEDMLNQVFLQIIFSLSEVSKIRKLLVGEDHFGSSQRMILEGDTLIISGYKSVVESAFEAMNINHLACLQELRIIQCWFAVSFQGNHLPKSLQKLTIENCGELEFGDHHQLHYNLVDLSIEAGSCSSLTSFSLDAFPNLKTLKISECKNLKSLSLSQPPHTALQHLTIYSCPEFVSFPGEGLAAPNLTHLEVTDCNKLEALPSDMNTLLPNLKSLHMLHCLGICRLPEGGLPPNLKELTIGVCEEQLKDLSWMGNLDSLTHFTILGFDCDSLKSFPEAGSLPQLPSLTTLKIIKASFSERRSAQSIGLIGAVV

>arahy.Tifrunner.gnm2.ann1.MR8NY8.1

MEKLEKTVSRFVQGPMQAHILADVHHARVEMAERFDRVDASNQRLEQYFSAMKIGVGGGGWIEEAVSSMEVDESGVEGLELGKKKVKEMVVTECGRLPLALKVIGASLRDQTEMFWASVKNRLSQGQSIGESYEINLIDRMAISTNHLPEMIKECFLDLCSFPEDKKIPLDVLTNMWVEIHDIDQKEAFAIVVELSNKNLLTLVKEARAGGMYSSCFEISVTQHDILRDLALNLSNRSSIRERRRLVMPKREDHGRLPKQWLRYKDRPFEAQIVSVHTEKIDMRECPMIKSLPKSTVSLKNLRLVICDEEVQEVWNEVEKAKPNLHIKVSEQYYDLDWLKE

>arahy.Tifrunner.gnm2.ann1.MS9PXH.1

MEDRRLSLALSSDYGWDYEVFLSFRGLDTGKHFAGNLYYALKQRGIRTFYADRELEGGEELEPTLLKRIQDSKTAIPVFSPGYADSAFCLLELAAIMDNSKAKGRLVFPVFYGVSASDVRRQTGTYEEAMAKHQRRNDHHVVQKWREALQQAANLSGSSFKFQNEYEFEFIEKIIEVVSKNVNRTLLCVAKHPVGLESPVQEVCKLLDVRPGCNKNQVCMVGIHGIGGIGKSTLAKAVFNYIADQFDSSCFLENVRENSSKHGLEHLQAKLLFDIVGEKNISLIGPSEGVPPIKHRLRQKKVLLILDDVDEEKQLKELAGGLDWFGSGSRVIITTQDQQVLRLHAIKTKYELNGLKFEDARKLFELKLKKKADPHFDDVINRAVTYCAKHPLALELISSDLGSINADEWESALGHYERNLGKEIYDKLKRSFDRLEKEVQSVFLDIACCFKGLSRTEVNNMLRAHHGFCPIYAIRLLEEKSLIMIEDNEVRLHDKIHEMGRKIEQEGKHGHRYLLSSYEAIVQFFKHKGIHDKIEMIILDLSSSEEQVVEWDGEGFKDMESLKTLINRNVYFSQDPNHLPNSLREFVNMRVLNFDDAECVKAIPSLSSTPNLEELSFSNCESLTEIDESVGNLRKLKILNAFGCSKLRSFPPLKLPSIEELNFTSCSNLENFPKILEKMENLTKLELDCTAIKAIPDLFSAENLVELSFSYCVNLIEIDESVGFLIKLRLLNAFGCCKLRSFPSLLLPSLEELDLSWCSSLENFPEILDKMEKITRLRLQYTPIKELPNSIQNLTRLRDLEMLECGMLQLPSNITLLPELRHIVFCRTPNQDEGEEKLSWMESSNSTLHASQCTISHGIFPNLFGWFSMYMEELDLFRVNFRFLPDCIMECLLLKELNLDQCHNLQSIRWLPPNLETLSVTCCRCLKDLDLTILPGSTKEYYNFRRLIVDNCENLQIIKGIPPEGSGNCIPEWFHPCNNGNSSVSFWFRNKFTAISLCVFLGALGKHQIAFYFCPKLEINGNTVNKWLLENKKYWFVQEAEADHIFILHEKQMKYENSVNEALVRNKEWNHAKIFVDVYSPRGWTEIDMQIGIHVFKEKNSMKDVQFTNPYNNVTGESNSVDSTQQSGTSIVQEPSYF

>arahy.Tifrunner.gnm2.ann1.MTL5BG.1

MELLFDDTTDAKISVIPIVGMGGFGKTTLAQLVYNDESLEQIFDLKLWIYVSENFDIQKITKTMIEAVTSSSCDMKDLGFLQLELKEKLIGKKYLVVLDDVWNENYGVWNDFQKPLQHRAKGKLPSSMQNLVNLRHLDIGESSLLELPKGMSKLQNLQHLTDFIVEENMIAELGGLANIHGPFGICKLENVRNSSEPSEARMMEKKHISTLFYHERDIFDKLQPHNEVKELLTYSYRGTTFPNWLGHSSYHNMSSIVLDSCRNCCILPSLGQLPNLKSLIIVNFNSSQTIGAEFYRNDSSQVVQSMFKAIANAIPTHLQSLSISGCWSAVSFPGDYLPASLKALHIKD

>arahy.Tifrunner.gnm2.ann1.MUV7N5.1

MASKLEGGAYLSSFVDAISKKLSSILEDDSVLEGNDSALELLERLDEILCDVEPVLDDAELKQFGNDRVKKWLVDLQDALYVADDFLDELSTKAATATPREPGNSYDWSRPVDSIIEDSGVNVIEKIVAKLGSVGCQVGHIMENSIHITDVFGRDEDKEKINKLLLDDTCDAESLVTVIPIVGMGGIGKTTLAQLVYNDPKVVEKFGTRAWVCVAENPDPVNVTRTVLGAIDSSPCNMDNFDLLQTNLKEKLTGKTFLLVLDDVWDDRRDMWEDFLKPFHYANNGSKILLTTRNENVASVFAPINLHYGLSLLSKRLLVGHSTISTNSKHYATVEPIGRKIVEKCKGLPLAVKTLGGLLRNKDNKEDWENILECKIWELSEDGCKIIPALRVSYHYLPSSLKRCFVYCSLYPEDYEFDKDELVLLWMAEDLLQPKENNTSKKIGCAYFDELVARSFFQPSSTKRGLFVMHDLMHELATFFARKFYFKLEVSENLHMVDSKIRHLSLFSNYRDTITLFGEACERAVHLRTALDFSSYRPSIDVESNPLLLQQFRVFSFRVKSVPDSIGELIHLRYLNLSGTCIVTLPESICKLYNLQTLKLRGCVELEMLPSRMQDLVNLCHLDIRGASRLKEIPKGMNKLKHLHFLSDYIVGKQENGMRELGTLDNLHGSFCISKLENVKNSGEALEAKMGNKKHINTLELNWLPDGDIDDVQTERDILDKLQPHQNLTELSINGYPGERFPDWLGLSCYSNMTKLSLDSCMNCCELPSLGQLPSLQHLEISKLDGLEKIDLEFYNKNNASFQQETPFKCLETLEIEYMYSWREWHFPDEFDGFPQLRILEIRNCPVLSGDLPSHLPALEELTIDGCKELACSLPRAPKLHKLHVKCDMFYGNLELHKVTISGSQLAKFVWEWLLHIQPPYVQYLYIDDCQSAISISANHLPASLQRLEIIDCSKLTFSEQLQHNSLTKILVEDCDSLTLFPLGDLPNLKKLTISECKNMENVEVAHALPSLRRLNISDCPSLVSLPPLGSAAPRLQELDIRNCPEIDCFAGECLPPSLEKLVIVECQKLASWISSNVLHSEGLTHLWLGSYFDVKSFPREGCLPASLKSLQFWDFPDLETLDCKGLHHLTSLTYIAIRYSEKLENITEEHLLASIKKIYIGEECPLRSKLEEMEDLRIQLGCDESESCDEYAWNDEDAASDSNSD

>arahy.Tifrunner.gnm2.ann1.MY0F6Q.1

MIPISTHIPTKALKKTIFILLTIASKSKSTLESAMITDVPSSSSSSSPSVKTRRWINHVFISFRGEDTRKGFTDHLYAALERRGCIKTFKDDHDLESGEIISEGLIKGIQESMFALVVLSPNYASSRWCLDELQNIVECREKFNQVVFPIFYGVEPSDVRYQRGTFEEAFRKHEERFKEEEGKVQRWRDALQKVASYSGWDSKDNHEAALIERIVDHIQKLLIPKLPSWVGNLVGVESRMKKLNSLIGMQLDDVRFIGIWGMGGIGKTTTARLIYESIEEQFNFSCFLANVREVSATNGIVHIQRELLSHLSVRSNYFHNLFDGVKIIANSLHNKKVLLVLDDISERSQLENLAGKQEWFGPGSRIIITTRNKHLLMTHGVHQTCELEGLVQEEALHLFCLKAFKQDQPKSKYQNLCNEVVEYTRGLPLALEVLGSHLCGRTPEAWHSALKQLRSSPHPEIQNSLKISFESLSSTEREMFLDIACFFKGMDKDEVVEVLENCGHFPQIGIEILIEKSFVTLGRGNKLEMHDLLQEMGKNIVFQVSPNDPGKRSRLWSQDDTSHVLTQNKGTEAIQAIVHYAQPYEARWSSEAIQAKVRYTRPYEARWSSEAFSKTSNIRLLKIRNVCRLSHGLDCLPYALRVLDWQGCPLKTLPLTDQLDVVDINLSWSKIEQLWHGRKILHKLKCINLSFSDNLNQTPDFVEVPNLESLVLEGCTSLTEIHSSVMQLKKLVQLNLRGCKRLKALPGKMEMSSLKVLNLSGCSNMNTVPDFGNCMGHLAELHLDGTAVTELPSSLGCLVGLVLLHLQYCRYLLCLPDTIHKLKSLEVLNVSYCSKLSSLPKCLQEMNNLEELYASNTAIEELPLCLCYLENIKVVSFAGCKESTSELNCFRVASAVCGPSPLIRLDLSYCNLLAESIPDGFCGLSLLRDLDLSGNNFVNLPSDISKHTALEYLRLNSCKKLQSLPELPLSIKMIEASNCSSLVTSKFHPSSKCSIFASPLQWHLPREQKSLLKGICFPRTRFDMFITGNEIPSWFAPQKSSSFAEIPFPHPSPPTEWLGFALCFLLVADRPLKGYHAEIGCFTATQTSLESCILVPDIQANKIMVPYKDDWRAESPVITRHLPDMEPKQPHLYILFLSIGEYLERMHTGYGFGLMSWSICSLRIVQAGCRLVCKEDVQDIYGNHSHTSSVGPKEKQTKNKQQRWTKLITFLSFPSTPTAHTNTSLQQYTHTSHRQCYSLISLPHVTSSTIILYTFITINFITLLLF

>arahy.Tifrunner.gnm2.ann1.MZFR22.1

MTGAVVGEAFLSGFIKVVFQRLLTTDTVNQVLGKKLGSGLVERLKISLHAAEAVLDDAEYKQLGDDRVRDWLNCLREAVYDADDLLDAVLTKAAIQKEVRSWLPSFFLNRHRKMVDNMEEVVERIEFLVSQKDILGLQRSTNDLKTKDNNLSSSSSSWRETTCLMEGKIYGREDDQQALIKIIHDRSESQLSVIPIVGMGGVGKTTLAKWAYSVAEGFDLKAWVCISETFDVADITKKTIEEITTDSCSLGSLNLLQNELQKILSGKKFFIVLDDVWSDDADKWKQFITPFHCGAKGSTILITTVAANACFPESNGNPILEEIGRKIVKKCKGLPLAVETLGRLLQGEDDAEEWNAVLRSDIWEFPMKDSKIIPALLISYFQLPPYLKRCFVYCSLFPKDHEFEKNELVLLWMAEDLLRLPKRGESLEEVGSQCFEELASRLFFKPAKYPSERYVMHDLLHDLAVFLAGDFYCRIEKLGEQEEKVLTRHFSYFPPAILYHSLSKLFKSTVKALRASESLRTSLYIDDLLSMESRASKLKYLRVLSFLDLDALPGSIDYFVVGKHKDNGIQELGGLSNLEGSFEIKRLENVADVRQAKSARMLEKNHIDNLLLEWSWDREMVSNTETERDILDGLQPHTGLKELRIEGYMGKRFPDWVGHCSYNNMTSVRLASCNNCCMLPSLGQLPSLKSLRIEGFDQLKRIGDEFYKSDNEHHSSPIAPFPSLEELVFHNMPCWEEWHVPHPEAFPQLKILQIERCPMFKGDVLNGCTTTINHQPHAALQRLIISGTGEGVAAPNLTHLSLIQCYKLEALPPDMNSLFPSLHSLDIRGSSNICRLPKGGLPPNLKELTVGDCEEQMRDLSWMGNLDALTHLTINGSRCKNIKSYPEVGSLPHLPSLTTLEIGGFDNLETLECNELLRLTSLQQLHIHWCPKLENMEGEKLPPSLLLLQLTMCGLLGEHCKNKHQLIWPKISHIPDIQVNGIKIS

>arahy.Tifrunner.gnm2.ann1.N06FBV.1

MAGALVGGAFLSGFINIVINKSLREDVVNLVLGKKLGSDLVERLKISLLAAEAVLDDAEYKQLGDDRVRDWLNCLRDAVYDADDFLEAVLTKAATKKEVRSLLPSVFLNRHRKMVDNMVGVVARIEFLVKQKDILGLQKNTKDNNLSSSTLSSWRESTCLMEGSIYGREDDQRALIKTINDNSESQLSVIPIVGMGGVGKTTLAKWAYSVVEGFDLKAWVCISETFDVAEITKKTIEEITKTTCSLGSLNLLQNELQKILSGKKFFIVLDDVWSEDADKWKQFITPFHCGVKGSSILLTTRMKEVASVVQTCPSHFLNELLEEYCWLLFAANACFPESNGNPTLEEIGRKIVNKCKGLPLAVETLGRLLRGKDDAKEWNAVLRSDIWEFSTKNSKIIPALLISYFQLPPYLKRCFVYCSLFPKDYYFDKDRLILLWMAEDLLRVPKRGESLEEVGSECFEELASRLFFKKLQDKYKYFEELDSMFFFKPEYPADTYVMHDLLHDLAIFLAGDFYCRIQELREQEEKKVLTRHFSYFPPGRLDHPISKVFKSIVKPESLRTSLYIDDLLSMESRASKLKYLRVLSFRRLDVLPDSIGKSIHLRYLNLSGTDVKTLPESLCNLYNLQTLILYFCSKLTMLPDGMLKLMKLRHLVLRGTCLKEMPRGISKLKHMHILDYFVVGKHKDNGIQELGGLSNLQGSFEIKKLENVVDVRQARSARMLEKNHIDKLWLEWSSDDEMVSNTETGRDILDGLQPHNGLKELRMKGYKGETFPDWVGRCSYNNMTTVWLESCKNCCMLPSLGQLPSLKSLYMEGFLELKCIGDEFYKSDNDHHSSPIAPFPTLEELVFDNMRCWEEWHVPDPEAFPRLRRLEIRDCEMLKGDMVNGIFGRRDCFLREDEEGRCDEMVGGGDALSIRPSQSFNATTINHLCCLQELLISGCPSIVSFPDNCLPKSLQKLKIWRCPKFEFPEQQQRNYDLVQLQIQDSCDPLSSLSLDVFPNLKNLEIEGCRNLESVSMSEAPHAALQRLSISWCFKLVSFAGEGLAARNLTHLQVSFCSKLEALPRDMKSLLPSLHSLKISGCKNICRLPEGGLPPNLKELYVGGCEEQLWDLSWIANFHALTHLRIEGYYDCDNIKSYLGSLPHLPSLTTLEIWGFDNLETLECNELLRLTSLQQLHIHWCPKLENMEGEKLPPSLLLLQLTMCGQVLKSEELGSNLFSSSERSERQT

>arahy.Tifrunner.gnm2.ann1.N0928S.1

MAHASFFRVSQKAYVVFICLLLILKPKGVASSSENTLKIKYDVFVSFRGPDIRRGFLSHLVDALSRKKINGFVDDKIEVGDEIPNSLIRAIEASLISLVIFSPNYSSSHWCLEELAKIVDCREKEGQFVLPVFLDVDPSDVRHQRGSYEDAFVKHEKKYDLVKVQRWRTTLQRAANLSGLHSTKYRNDYGLLEAIIKHLIKRLNHEQKYISRGLIGIGKSIALLESELNPELGDVRALGIWGMGGLGKTTLAWEVFNSVRSQYEGSCFLRNLRESSAREGIFSLKSKLYSELIGEPGLKIDSSNGLPPFLEKRLGRMKVIIVLDDVNDFRQLKILFGAREQFGSGSRIIVTSRDKQVLHTEVDFIYQVKPLQVDEALRLFNSIAFKHEHRDLEMEFREQSKKVIEYAKGVPLVLEVLGHHVHGKGKEIWESLLDKLKNMPDKEVHNVMKLSYDDLDRDEKKIFLDIACFFKWLTVTEDDIKLLLKDEINSVAFVLERLKDKGLITVSERTVSMHDIIQETAYEIVRQESIEDPGKRSRLWSFNDIYQVLKGDKGSEAIRSISAKLPLLNKKLQLSPQVFAKMSNLQFLDFLAPYTCPSYFPQEPMSLYFPEGLESLPNELRVLLWVHYPMEALPTQFSAENLQILFLPVNRVKKLWHQEQNLQSLKMVLLECSTQLTESPDFSNAINLEVFALSWSLKLIHVHPSVFSLEKLTSLYLVNCVSLTSLISDTHLRSLRYFFLSGCTALKEFSVISMNMVYLYLDHTGIKQLPASIGIQGKLEKLNLANSTIEYLPESIKHLLRLRILDLRDCRELQTLPELPPNIEDLNVEACISLKTVLFPVTVAEQMKEERKKVAFWNCVALDQHSLEAIGLNAQLNIMKHLSRFESDSYQDYDAHEATASFVYPGSRIPKWLLHRTIGDHITIDVPFGLPSNQLDFIFAFIVPRVASEGLFLNFSLSVGDDEDEGKSIKLRLSRPAREIASDHVYLMYDVACSNYLRSIAKSQAQFEIKVSVASEYTPLLLKGLGVSLINVAEYQSFVQQIKWLDTTSDTIFNWSITREMDSYNCEPSYQNCSLEFGKFYGTQCLFQQHVHALGAGRGRDMLVL

>arahy.Tifrunner.gnm2.ann1.N9YI8E.1

MITDVPSSSSSPSPTVKTRRWINHVFISFRGEDTRKGFTDHLYAALERRGCIKTFKDDHDLENGEIISEGLIKGIQESMFALVVLSPNYASSRWCLDELQNIIECREKFNQVVFPIFYGVESSDVRYQRGTFEEAFRKHEERFKEEKGKVERWRDALQKVASYSGWDSKDSHEAALIERIVDHIQKLLIPKLPSWVGNLVGVESRMKKLNSLIGMQLDDIRFIGIWGMGGIGKTTTATLIYESIKEQFNFSCFLANIREVSAKNGIVHIQRELLSHLSVRSNYFHNLFDGVKIIANSLHNKKVLLVLDDISERSQLENLAGKQEWFGPGSRIIITTRDKHLLMTHGVHQTCELEGLVQKEALHLFCLKAFKQDQPKSQYQNLCREVVEYTRGLPLALEVLGSHLCGRTPEAWDSALKQLRSSPHPEIQNSLKISFESLASTEREMFLDIACFFKGMNKDEVVKVLENCGHFPQIGIEILIEKSFVTLGWGNKLEMHDLLEEMGKNIVFQESPNDPGKRSRLWSHDDISRVLTQNKGTEAIQAIVHYTRENEARWSSEAFSKTSNIRLLKIRDPCRLSHGLDCLPYALRVLDWLGCPLKTLPLTDQLDVVDINLSWSKIKQLWHGKKILHKLKCINLSFSQNLNQTPDFVEVPNLESLDLGGCKRLTKIHSSVMHLKKLVQLNLEGCKRLKALPGKMEMSSLKVLNLSGCSNMNTIPDFGNCMGHLEELRLDGTAVTELPSSLGCLVGLVLLHLQNCRYLVCLPDTIHKLKSLKVLNVSYCSKLRSLPECLQEMNNLEELDASNTAIEELPLFLCYLENIKVVSFAGCKESTSVLNCFRVPSAVCRPSLLIRLDLSYCNLLAESIPDGFCGISLRDLDLSGNNFVNLPSDISKHSTLEYLCLNSCKKLQSLPELPLSIKRIDATNCSSLVASVQWHLPRERKGICFPRTPFDMLITGNEIPSWFAPLKSSSFAEIPFPHPSPPTEWLGFALCFLLVADRPLKGYHAEIGCFTATQTSLESCILVPDIQANKIMVPYKDDWRAESPVITRHLPDMEPKQPHLFILFLSIGEYLERMHTGYGFGLMSWSICSLRIVQAGCRLWDAVENKSTVLLYGGGAIVAVWLSSILVGAINSVPLLPKIMELVGLGYTGWFVYRYLLFKSSRKELAEDIDGLKKKITGTE

>arahy.Tifrunner.gnm2.ann1.NB3YGV.1

MAGALVGGAFLSGFINVVFDRFLTTDAANLVLGKKLGPDLVERLKTALLGAEALVADAELKQFGKPLVRKWLDSLRDAVYCAEDLLDAVLLKATTQKNACSSWSLSFFSNRDRDDTVDKMEGVVRRIEDLGKQKDFLGLEKIPTGSSSWRTPSTSLVKGSVYGREDDEKALVQMLNDNNEHDLSVIAIVGIGGVGKTTLAQWLYNNQEEFMKGFDLKAWVCVSEKFEVVETTRNVIKQIHGGTCSLDDFNSLQNALKEELSNKKFLIVLDDVWSDDGDKWSNFTTPFQYGKKGSIVLLTTRGKNVALAVQNCRPYFLKGLSEDYCWSVFANNASFPESNGSAALEEIGRKIVKKCDGLPLAAETLGRLLRTKHDVQEWDKILISDIWGFSVEKSKIIPALLISYFHLPPYLKRCFVYCALYPKDYEFEKDKLILMWIAEDLLPPPKRGESLEEVETLRTLLLFGDFSSPNFNIEAATCEILSKCKYLRVLSFDKLDVLPNSIGELIHLRHLDLSYTRIETLPESLCSLCNLQTLKLRHCYELTTLPSGFHNLLSLQHLDIRGTSLEETPRKMSKLNQLHVLSFFVVGKHEDNGIQELGGLVNLHGSVEIKKLENIDDVTEAKSAKIMDKKHIDELCLEWSSGDDLVSSTQKEKDILDNLQPTNGLKELKIRGYKGTIFPDWLGNCSYQNMTSVYLESCKNCCMLPSLGQLPSLKSLSIEGLDQVRSIGEEFYKNEGDHHSSHIAPFPSLESLLFYNMPCWEEWHLPDSKAFPQLKSLEISNCQMLKEDMLNQVFFRIVSSLSNVSKVRKLRIDDDDDLRHIEAMFLVGDSLSITGRESVKESAFKAMISINHLRCLQEIHIQGCRKLEFPQLQQHKYDLVDLQIYESCDSLTSLSLDVFPNLKNLQISWCRNLESVSMSEAPHAALQRVIIYQCNKLVSLAGEGLAAPNLTHLQVAWCSKLEALPHDMKSLLPSLQSLEIDGCSDMCRLADGDLPPNLKELHVQIGEQQMRDPSWMHNLDALTHLTINGYKCDNIKSYPEVGSLPHLPSLTTLKILGFNNLETLECNELLHLTSLQQLSIHWCPKLDNMEGEKLPPSLLLLKIQDCPLLGEHCKNKHQLIWPKISHIPTIQVDFKQIV

>arahy.Tifrunner.gnm2.ann1.NDAQ9Q.1

MAAKLQGRAYLSSFVDAVLNKLSSLDVNSTPEAKKLDDQKLLQKLRKSLRATRPVLDDAEQRQIRDQEVNKWLVDLQDALYMADDLLDELSTKAATATPTPTQRDPGNSSSWSHYVDSILEDSDDDEMGVVTSMQDIVDKLESIVEEKDDLGLKQGDAKDLEDMSWRIQSSLLESSDIYGRNDDKEAIIKLLLDETCDDILSVISIEGIGGIGKTTLAQLVYNDARVKEKFAIKAWACVATKFDPVIVTKSIMEDIASPCNKVNLNSIQTELKEKLTEKTFLVVLDDVWDNQQNLWDNFLKPFLSGNKGSKILLTTRIKNVDSVVSTTNLHYKLDTLSNEDCWSLFLKHSSISTSSRQYEILEQIGKKIVEKCKGLPLAVKTLGGLLRSKDKVEDWENLLRSEIWELPEDESKIVAALRVSYHYLPSNLKRCFVYCSLYPQDYQFDKDELILLWMAEDLLQPMEKNTLENIGCAYFEELVARSFFQLSSKDAGLFVMHDLMHDLASFFAEKFYFRVRELGDPQKICSKTRHLSYMVNPDDSRFGEAYKGAIHMRTFLNVCRGPLSITLESDSLLLQLQMRYLRVLSFKHFSIESLPDSIDKLIHLHYLDLSYTSILTLSESLCKLYNLQTLKLSYCKKLEMLPSRMQDLVNLRHLDTRGSNSLKEMPKGMSKLKHLNFLSGYIVGEQVENGIRELGALDNLHGKLCISKLQNVKDSGEALEAKMDSKEHISILELKWRREGDTVDVETARDILEELQPHRNLKELSIDGYRGEIFPDWLGLSCYSNLTDLSMYRCKNCRQLPSLGQLPSLKDLRIFEFDGLERIGGEFYNNAESSHQGTPFRSLQTLEFAHMPRWREWHIPHDFDGFPKLKSLSIVNCPVLSGDLPAHLPALEKLNIVDSDELACSLPRAPKLHQLLVMGPLYESEGYKHLSKPHEVVILESLLTKSVLECLPHIQCSCVQRLTIENLWPAISVSGDNLPDSLQYLQITGCSDLFSGPLHSKSLTEILVQECDSLTLLPLGALPNLKTLSIIDCPEMDCFGEECLPPSLTTLRIRRCQKLERWITWRGLQSEGLTRLFLEDWDEVKSFPREGCLPASLQWKQEFQFMDVVRVECSKKFIDMIMQARCTSVPRLTQGPGKGRNQGCNVRSAFNAGSGKGPQPRFLMIQTLKYNMDFFAGIAEIQYDWDCHSFKFHGQPLLVH

>arahy.Tifrunner.gnm2.ann1.NFL8TR.1

MKHTKHIDDYETEYTNLIEGHDDSFVMHDLLHDLAIYLARGFYCNLEDLEEEEIMIQTRHLCGILRYCSLKLYNSRSKVESLRTLLLFNDQIWYHEFNIETAICDILSKCKYLRALSFHKLNVLPNSIGELIHLRYLNLSGTSIEALPESLCNLYNL

>arahy.Tifrunner.gnm2.ann1.NJ02RL.1

MAAELVGGAFLSSFLNVLFDRLSDPEIINMMRGKKVDQKLLQRLENILNVVEAVLNDAEKKQITDPAVKRWLEDLQDAVYDADDLLDEVATKAATQKDPPGNFLSRFLSLQDREMVTRIEEIIARLEDIAKHKDILRLEKIAVKNMSGRIESTSLVQKSDLFVGRDQDREDIVKLLLDDTNDGELSVIPIWGMGGIGKTTLAKLVFNDDRVQRKFNINAWVCVGEEFDVLKVTKTVIEEITSSPCDMNSLNLAQQHLRSKLTGMKFLVVLDDLWSNNYTAWANFLIPFRCGSEGSKILVTTRSEKIANMVKGFHYQAYNLSALNDEDCWVVFANHAFLSGERLAFEKVAREIVKKCKGLPLAAQALGSLLRSKDNEKDWNNVLNSEIWEFSEEEIEIIPALRISYYHLPSYLKRCFVYCSLCPKDYEFDRDELVLLWMAEGLLQRPRGRSTLEEVGYEYFSDLASRSFFQPSNNAYNTSFVMHDLMHDLATFYGGKFFSRTFEVKNAAKHDVKTRHLSCARKNDDDSFMKIMEACKRLKHVRTLLQLNFRKDGGIPEGDSVAVPSDLLEQLKCLRVLSFKFFPDDENLLHHSIGELIHLRYLDLSDTSIVTLPESLSCLYNLQTLKLTYCKKLKKLPSKMQNLVNLRHLDVSETGLEEMPKKMSELKDLQFLSSYIVGKHEENGVGELGELAHLHGSLWIEKLENVKNSGEASNARMDEKIHLNTLYLWWSTFEESEVCDSQSEKDVLDKLRPHKDLKKLSISCYRGTMFPDWVGQSLYHNMTWLELRGCRNCWVLPSLGQLPSLKRLLISGLDKLKKIGGSFYKGDGTHQHQETPFRSLKFFIIQRMPCWEEWESYECDDDKDAPFPQLDELHILQCPKLRGDLPTFLPSLKSLFIYECKELGCYLPRAPILRKLIIRDKQEARMRELPLSMLETLVINGEQLVDSLFEAMTHTQPTSLIELDISECSSAISFPEDSLPPSLEYLGIFNCKNVEFPMHHQQHHSLQRLEFEWIKHKGCYIAGKHEENGIGELRELTHLHGSLRIEKLENVKNSGEASNARMDEKIHLTTLYLSWSSFEESEDCDSQSEKDVLDKLCPHKDLKKLVIRRYRGTMFPDWVGQSSYHNVTELQLIGCRNCWSDATHRHQEKPFRSLKYVSFRDMGCWEEWESFACDDDDAPFPQLEKLVIKHCPKLRGDLPTFLPSLKSLVIEKCEELACDLPRAPIISQLEIYGKQEARMRDLPLSVQHLSISGKQLVEYVFEAMTHTQPTSLSYLFISKCSSAISFPGDSLPPSLRNLSIIHCKNVEFPMQQQQQHHSLQTLFIDNSCGSLTSFALPAFPKLKHLTIARREDLTSLGNITLPASLHSLQIFECPLLGEGIERKDPHIWPSISHIPYISVDRTLIHNDSTS

>arahy.Tifrunner.gnm2.ann1.NJ1UU6.1

MELFPSSPKKYDVFVSYKGGDTQINFINNLNSALTQAGIKIYIDSYLRTGDAIWPPLREAIDNSSVALVIFTKGYASSKWCLEELGKILECRRTEGMGVIPLFYNVDPCDVRNQSGTYGEAFAKHERCLLLDEKDNIINKEDVQKKLSQWKGALTEAANISGWYTRSNKNQSQVIEKMVGDVLEMLELRFPKELKSKDLVGFDKILKEMQLLLSRNRGNLLVGNVQVIGICGMSGIGKTTIAKILFSQQFPLYDSVCFIENMKEASQNIESLKEKMVSELLKDENQNSKESTLLIGKRLSNKKIFVVLDDVDNLEQLEVLCEEFKYASPESKLIITTRNKDLLRGIMVDEYDVYEIKTWSFEESLELFCSSAFKSKHPKKGYEDLAKRAVDYAGGVPLALNVLGSSLGDRSIEFWERELDKVENYSCDRIQKVLRVSYEGLHDLEKKIFLDIAFFFKDESKDFVERVLNACGFYATGGLKVLEDKALITISDGNKIKMHGLVQDLALSIVREGIEDHGKRSRLRDIAEISSVLECKNKGSDAVEGIKLDLSQIDDLHLNANAFSIMTELRFLKLHTPCGKISGNMDYPIVLDQFSSKLSYLEWNGYRLKSLPESFCATKLVEIRMVQSHITELWHGVKNLVNLEGIDLSECKYLENLPDLSKASKLKWLNLSDCESLVELHPSVLSLDTLETLILNGCKKLKSLKSEKHFRSLKKVSVEGCTSLKEFALSSDKMESLELKNAGIEQLHSSIGLSSNLRYLNLEGLRFQNVPNELSSLTSLAELRISDCKELVLEKQKLQALFDGLRSLRLLHLKDCSNLLELPENISVLSKLCELRLDGSGVETLPESIKHLSLLEILSLENCLNLRQLPELPTTIKELTAANCKSLVTVPTLKTLATMMTGREKFVSFENCTKLDGSSLCRIAEGAELSSMNAAFQNVFVRSKGASGASCHNYNFVKVCSPGSRVPEQFTYRSRESSFKVKIPSHSNILGMVLSVLISPSEGIKNHGARIFCQCYDADGRKVGYATRWFHEATKDLKGDHVFVWYDALHFDSIVKSLEEQEIIFEFFVANELGERDVLNVLPKECGVRLIFDSELHNLLRNLELDFESKWELGLRLGSELGLEMGSELRLELESQQHRPISMATERFYFNEDESDGLYNEEHVHQSERKGIHNIKMFSVEDMIVSMTSSNATRIKY

>arahy.Tifrunner.gnm2.ann1.NKM1DI.1

MDSSNRIESSSATAPRRICKCDVFISFRGPDTRNTLVDHLYNHLIRKGIFTFKDDKTLEQGQPISSQLLQAIRDSRISIVVFSPDYAASTWCLDEMAAIVDCQREFNQAVFPVFYDVDPSHVRKQNGVYQEAFISHSQKFTQDPAKVQRWKSAMTTLANSVGWDVTNKPEFGEIEKIVQKIVKTLNHKFSGFVDDLIGMQPRVEGLEKLLKLRSEDDGFRVLGLWGMGGIGKTTHATALYDRISYQFDASCFVENVSRLYNDGGAMAIQKQILRQALDEKNLDTYSPSEISGIITNRLHSIKVLVVLDNVDHLKQLEELAIKPKLLCEGSRIIITTRDAHILKVYEADEVHNVPLLSDKEARELFGRKAFKREGPSNNCEELIPEVLKYAQRHPLTIKVVGAFLCSRNAAQWRDALDRLRNNQEDEITNVLRISYDGLKYEEQEIFLHIACFFRGEREDYVKRILDSLGLYPIIGISVIAQKSLITIRNQEIHMHEMLQELGKKIVREKYPQEPGSWSRIWSYKDFHHVLMSETGTNKIKAIVVDIKEDITDCSQMVEGLSKMKDLKLLIVHQKNNHSESRLKSLSSCLKYLSWHGYPFPSLPSVFHPSECLVEMNLSASSIKSLWEGHKVLLDY

>arahy.Tifrunner.gnm2.ann1.NUHQ9Q.1

MAGSSSSDQGVSHFKYDVFLSFRGYTRLRFTDALYHALINNGIDTFRDNDKLRIGKELEGALLEAIESSRMSILIPCDEYPTSKWCLDELVKIMECSENGRKRPVLPVYYYVERSDVQYQLNEYAKAMTAHEEKGRYNHKLEAWRSALSEVGKIYGQRCDQNTPFGMAINKIVEEVIKRLPPLPLYIHRPLGCDSELEEAKSLLQIGSSHACRLMLGIHGDGDLSQFVAELYNKIRCDFASASFLSGISKKTNASGGGLEDLQKTLLSEMKEKVKNKIGSTFSGSFEIKRRLGKKGVLLILDDVDNIQQLKSLAGEIDWFGPGSRIIITTRYEDVLDEHAGVDIKKYRFDEGEFEGNGGSSTMMEENVVGLEEDFNVIINQLKEEDSARNVVSIVGMGGLGKTTLARKVYNSDEARELFPCRAWATVSKECMPTEVFKELLKCLKVPEADYENAGEEKLKDMVRKRLNGKKYLVVLDDVWEANVWDKLKGPLPDNNNGSMILVTTRNDQMANYTRSKEPHHQLHLLDEDQSWEMFRNKVFGREECPPPLELIGRSIAFESCKGLPLAIKTIAGIVAKKERSEDAWEEIKNLLPYWSVAEDKEGEEMMKILKYSYDDLSEKMKPCFLYLGVFLEDAEILVRDLIQLWMSEDFIKPIQTGRRLIEPEDIGEQYLKELVDRNLVQVVKRRSDGKGVKACQIHDLIRDLCILVSNDNPDNSNNARTFTFSRSEGSYACSVTCNHSSTCSLFVYGDVDGWSHHIPEGCPVNVLYLNGFDGLPNEKNAEDLERLKSLKFLKMDCLVLHRLFKLQSLQTIQVNCIWKPKKISVDEGLKQMRHFRCPGLGGEQLLLDERVKERMQNFQTLCYVYADSQLGLLLNNGYFPNLRTLGLLISEDECQLVDENLRSLLRLSELRKLKLVFLNVFERVPLGKIRFPSNLTKIVLACKDFNDQDMNALGRIRSLQILKLHQIICEQYVLNCGGAGSFPQLQVLIMIGVSVSSVTLEAGAMSRLRSAVFRQCLGLTLQSLPERMLSLEFDLHFIEPDSDDDDERRRRLR

>arahy.Tifrunner.gnm2.ann1.P0VEPY.1

MAAKLVGGAYLSSFVDTVLNKLSSIDVNATPTARKLADKRLLQKLRKSLRATRPVLDDAEQKQIRDQEVKKWLVDLQDALYMADDLLDELSAKAATPTQRDPGNSSSWSHYVDSILEDGDDDEMGVVTRMQDIVDKLESIVEEKDDLDLKQGVAKDLEDMSWRIQSSLIESSDIYGRNGDKEAIIKLLFEETCDDKLSVISIEGIGGIGKTTLAQLVYNDARVKEKFAIKAWVCVATRFDPISDSKAMIEEITSSPCDMVNLNSLQTQLKEKLNEKTFLVVLDDVWDNQQNLWDNFLKPFLSGNKGSKILLTTRNKNVDSVVSSTNLHYKLDILSHNDCWSIFLKHSSLSTNSRQYVILEKIGKKIVEKCKGLPLAVKTLGCLLRNKDSEEDWENILEKDESKIVPALRVSYHYLPSHLKRCFVYCSLYPEDYQFDKEELILLWMAEDFVQPIENCTLENIGRAYFDELVARSFFQPSSKDVGLFVMHDLMHDLATFFAGKFYFKLREFEDSQKICSKTRHLSYTANPDDRILKLGEAYKGATHMRTFLDVHLFHRYGSINLESDSWLLQLHLRCLRVLSFKYFSIESLPDSIEKLIHLHYLNLSHTPIVRLSESLCKLYNLQTLKLCYCKKLEMLPSRMKDLVSLRHLDIKGADSLKEMPKGMSKLNHLNFLSGYVVGKHEENGIRELGALGNLHGSLCISNLENVSNSREVLEAKMGNKKHINILELKWLPDGDIVDVETERDILDKLQPHRNLKGLSIVGFRGEIFPDWLGLGLSCYSNMTKLVLSSCKNCCQVPSLGQLPSLQHLEFFEIDGLERVGCEFYKNNESFQQDTPFKALQSLKFKRMRGWRKWHIPDEIDVFPKLKSLSIRDCPVLSGDLSPHLPALEQLTIWNCEELACSLPRAPKLHQLTVKGSGFYLSQEKGVLISETQLAKSVLEWLPHIQSPRLQHLEIKKCWSAISISGDYLPDSLQILRIQDCSKLTISEPLQHKSLKEIHVSRCDSLTLFALGSLPNLEALFISKCHRLISMPGLGFADLEKLVIAACPEMDCFGEECLPPKLTTLHIAGCEKLERWITSKGLHSEGLTDLTLGQWNEVKSFPKEGCLPVSLQSLQLLFFPNLETLDCKGLRHLTSLQELSITGCSKLENITEERAPASIAKFVIGKECPLSCKLEEMNDPRIQLQSSFIGSLIRQAYRQMTEDRRKHINAKVQFPHPSEEVSHYPDPEIQYGFFLRIAESMNCPSFKFHDQPSLVPTLQLFP

>arahy.Tifrunner.gnm2.ann1.P4Q5K4.1

ISSLLFSNMAAEAVLSSVLSVVFDRMSSPEVVNWIKGKKLTHKLIERLKTNLCAVRAFLIDAEQKQIKERAVKDWLDSLKDAMYVADDLLDEVFTKAATQKDPGTFLSRFSGILNLQDRDVANRMEEVIDRIESLVIQKDTLGLREIPKENMSWRITTSLVETSDVCGREEDKEAILKLLLDDDSDDDTGGQSDVSVIPIVGMGGIGKTTLAQLVYQDDKVKENFDFQAWICVSEEFDVFKVTKTIIEAITKSFCSLTDLNLLQHDLKEKLSRKKFFVVLDDVWSESYEDWDKLLKPFRKGVKGSKILITTRSKRVASVVQTVSPYELSLLSEEDCWLVFSKYARLSTCSMENPTLKKVGKDLVKKCDGLPLAAQALGGLLRGNSDIKYWNHLLKSEIWELSDDKIKVVPALRISYYYLPSYLRECFVFCSLYPKDYEFSKDELILLWMAENFLQPAGKKTPEEVGDEYFDELIARSFFQPSKIRENKFVMHDLVHDLAMIFGGEFYFRAEELENAVEVDIKTRHLSHNAKGNYPISKLLGVCDRVKHTRTFLEINLEREIPFNMENAPCILLSKLKYLRALSFKCFPLESLPDSIGELIHLRYLDLSWTDIMTLPDTLCNLYNLQTLKLFGCWKLNALPVGMKDLTNLRYLDITHTGLHEMPEGMSKLTSLQVLSNYVVGKREGNKINELGILANLHQTIWIEKLENVVNSSEALEARMFEKDGIESLILMWSPDEYENIVDSQIERDILEELRPHSNLKKLEICGYRGTTFPDWLGHCSYRNITQITLGDLLSGYFKNCCMLPSLGQLPSLKHLDIAKFERLPIVGDEFYRNDESCVETPFPMLETLTFQSMPCWEEWRSLEFNAFPRLRKLIIWDCPMLRGDLPNQLPSLEDLTIHNCEQLSSCVPRASGITCLRIEGNKEVRIGELPPLLDKLSITGKHQAESVMEAIAHTQLTCLRSLIISNCSSHVLFAVSSIPASLQELTISNCKKLEFEMEGQHHSLHYLTIRNSCDSVTSFSLDSFPNLVSVHIRECEKMEYLVVSRSLSSLRSLHIHDCGSLKSLQTLWMASPQLEYLSLRGCPEIDLSAIGDPQRSLRSLSISYCEKQLSCVASQFDGLTHLYIKGENESVKCLPKEGWLPATLESLTLDGIKSVEMLECKGLAHLISLQQLSIYECYNLENIDGEKLPASLLRLSISESPLGKRCEMKDPQLFQQEITSAVTILLMKATKQSGTIMDTIQEIQKWHDTVIDIESSLNELHQVLLDMTVLVQSQGKQLNDIESHESKANSYVCRGSGSFSLQGSTRRIPEVHLHCHHNIDLLY

>arahy.Tifrunner.gnm2.ann1.P5WFZY.1

MTGVLVGGAFLCGFINVVMDRLISADAVNLVVGKKLSSDLVERLRNALTDAGALVDDAELKQLDNHDVKEWLNSLRDALYTADDLLDRVCTIAATQKGVRNFLPTFFNSEDRQMVNEIERVVRRIEDLEKRKGKLGLEKISTASFSWKTPSTSLVRGNVYGREDDKKALIKILNDNNEHHLSVISIVGMGGVGKTTLAQWMYNNAELMEGFDRKAWVCVSENFNIVETTKNIVKEISTNTQDLDSFNSIQDALKKGLSKTKFFIVLDDVWSNDHHQWKDFLAPFQYGDKGSTILLTIRTLLLVSVCSQTNYHPHYLIPLSEHYCWSVFAANASFPESNGSPTLEEIGKKIAKKCNGLPLAAETLGCLCRRHDAEEWEKILRSDIWGFSTNDSKIVPALLISYFHVAAHLKRCFVYCALFPKDYHFKKDELILLWMAEDLLRLPKRGESLEEVGCKCFEELVSRLFFKKLQDNDEYFVMHDLLHDLAIFLAGDFYCRIEELGEQEEKMVLTRHLSYFPRGRLDHPISKVFNSNAKLESFRTSLYIDDLFSMESVASKFICLRVLSFHKLDVLPESIGESIHLRYLNLSSTDINTLPESLCNLYNLQTLILYRCTELTMLPVNMRNLVNLRHLDLRKTSLEEMPGGISKMKHLHTLSSFLVGKHEDNGIKELGGLSNLHESLELKKLENIVDVKEAENARMTNKNLINELHLEWSSGDDMVPNTKAERDILDSLQPHNSLKVLTIKGYKGTIFPDWLGNCSYNNMTSVSLESCNNCCMLPSLGLLPSLKALRIQCFSQLKCVGMEFYKGIGDPSLHIAPPFPLLGSLEFGNMPCWEEWHLPDSKAFCQLKSLQITDCPMLKRDMLHQVFMRIVSSSSDALKVSKLVIQSQAAGFPGMSLNGDTLSITGCESVVESAFNEMISINHLPSLQEIQILWCSFAVSWPNNCLLPKSLQKLTISNCNKLEFPQHKYDLVELLIHSCDSLTSLSLDAFPNLKNLQISGCKNLESVSMSEAPHAALQRLIIGGCSKLVSFAGEGLAAPNLTHLKVKYCSKLEALPRDMKSLLPTLQSLEIYGCPNICRLAEGGLPPNLKSLDVGICEQQMRDLSWMSNLPALTYLKFYGYDCDNIKSYPEVGSLPHLPSLTTLVIYWFDNLETLECNELLRLTSLQQLHIYDCSKLENMEGEKLPPSLLLLKIEYCHLLGEHCKNKHQLIWPKISHIPTIEVNHNNHNHWRFLSRYIVMKEKCNSNILYPTFENFYILYPIAIIYS

>arahy.Tifrunner.gnm2.ann1.P78B16.1

MGRGKRLLSLLPRRMLGLFHNPQIKPNFLTSLMHQSLLTLVAVADDAEAKLFPLSGSASCNVAEWMHHFHDVMYRLSMLLWDRLFANAFADFEPDWEFILQKIEVMTKQKHLLLLTEKLQGEKDQEQQQRSSYLKADVYGREKEKNELVDYLLSDRSSGTVGNTSVIAIVGVEGIGKTTFAEIVYNDDRVKASFELRGWVDYQEGIDAVSLGNIVLDSFDEDFLCSDGLKEVIGRLRRCLNGKKFLLVLDRTQSLTAWETLWACFADAAVGSAVIFTTSKLEAALEVRSNQVLHLGLLSLEDSCSLFSDHAFGDGNQNEHLEFVTVTKQVVRKLGGLPIAVKKLGSVLYDYQDLYAWRELLKINRSHLLDLSDFSLPIQFSRCRPYNFHNMVRLEIVGAWRLNLPNEFGELRCLEYLDLSFCEVRRWPNSVTPLSRLHTLKLSFGEYPPILGVILPFVNLHYLDITGRSNFPIWLPIELGEMKILQTLLGFAANKDSGSNLVVLASLPSIRTLSITELQNVVDATCASLANLKGKRYLENLTLRWNQLCAPELKQAPEHLQPPGQPKFLETLGLSECERSLEYLEPPEQLKMLEILGCPDRIFPNWLGDSSFRTLEVLHLRDCRNFPFLPPLGQLSSLKELSIQGCDNVRSVGSEFYGHCTSYVPFKSLEVLWFVDMTNWKKWILLDDGRLHFPCLRELYLIHCPELQQDLPKHLPSLKKIEIIKCDRLVAPRPKILDKHELLVQEQEKGKEKDCDDIVATSAAECEKEKDCDGIVATSAAEPNEFNFIMEPIIREVDEPVVDDDEDDGNNDVMLDFEYSFNILKVADASELCNLPGELKSLRIEGCQFLESLPNQFLKYCPDIFELFLVDCCSLKHFADVLHPKSLRTLHIRKCPVLEFIIPLGVHKKFKLLKHLFISSSCESLTSISINLFPRLQTLYIKDCPNLKSFSVAQGLRDQNLKLESLEIRDCPNLISFPERGLPTPSLKTMTISNCRSLKSLPNRFLTLTSLQSLLIDKCPELESFPDGGLPSTLSLISIAFCDKLTPQKEWKLNTLCSLTCFEIEGGCIGMKSFPEKDLLPRNLKSLRISKLSSLRVLNGTGLQHLTALETLEINCCHGLISLPEGLPSSLTCLCIKESSILIHKLSYKAGEEWSKVAHIPNIQIDDDAGHGVNKETIIPGKEVEAASLSFKQPQPKRGF

>arahy.Tifrunner.gnm2.ann1.P92HK0.1

MENYKAKFMCSYGGKIQRNTLDHTKLSYVGGHTKILYVHRTTITFPAMLAKLATLCNAASASADVSFKYQLPGEDLDSLISVTDDDDLENMMIEYDQLHSASPKTARMRLFLFPNLPLDSAAASSEPEPDPAVRQLQLPPPPPPPPSSSLSSRLVGPLSTHGKYDVFISFRGEDTRTNFTCHLHEALCRKRIKTFIDYELHKGDEISPSLFRAIEDSYVSVIVFSENYADSKWCLEELVRILECRKEYGQVVIPVFYEIDPSHVRKQNGSYKEAFEKKHMQNSMLDINKIQKWKDALAEAADLAGWDSHSRSYRDDTELIQRIVKDVLQKLIYHYPPNDFNGLVGIQEKSAPLESLLREARSVGIWGIGGIGKTTIARYIFDKYSHGFEGSCFLENIRERSGDHVQGLHDLRDQLYSVLLNEKVRQSSTAKSTFVECRIRRQSNFIVLDDVSSSKQLKYLVGELESYGPGSKIIITTRDKSVLQNRRVEKIHEVEGLDSPTSLTLFSLNAFNEDSPEVGYKELSRRAVNYCKGVPLALVVLGSFLHSKTEAEWGSALNKIEKIPNEEIQTVLRLSYDELDYEEQQIFLDIACFLKGELKENIVSLLDSCSLYPVIGMRSLLDKALITISNDSVGMHDLIQQMGWEIVRQESIENPEDRSRLWDLDDTCDVLKNNKGTGAIQGMKLDTYQIRQNLSLSVDTFKKMPNLKYLKFFISIRDHGKLSGLQLPEELESFSEKLRHLEWHAYPLPSLPSNFCPEKLVTLRMPNGQFRRLWNKVQDLVNLKDVNLAGCQELVELPDLSKAKNLRNVDLFGCRSLSNIHPSILSCSTLERLDLTGCSKLETLESQTHFKSLWHLNVSGCKSLAKFSVSSEEVEVLDLMMGVKVLHPSIGRFSKARILHVDGHRLENLPKELSCLKSLETLSLHRCSHVSSKENLHLVFNGLQSLRELYFMDCHYLFELPDNINQLSSLQKLALDGSYVVRLPETIKHLSALETLSLKGCRRLQSLPELPSSIIRLEADNCTLLPIASSSLTNFRPKEDGRSDDSFHNCVNFHVQKHTDSFHQYLRDLAHRYELRRIKRRGGGGRRAMFADINFRIFYQDHRIPKWFTYQTKGASITFELDQPYDLCSSFVLCVVIAPGWPSPIKYGLILQYQCHLEDSDMNKYSTSKILLDDVPAERDFDHIYMSLDRGGIIEAIKAYKLKYGSHSVSECYNNLKVTIEFYFYCCTFQWNQDHDWLIRECAVYPLVAPDSRLKQVELELELGMENKRPRGILEMEHTEGGVGVGSSSDRGPLPSTKKLKELC

>arahy.Tifrunner.gnm2.ann1.P98XPP.1

MEILSSVAGKVAEYTVAPIGRQFSYLIFYKANFKELSKRVTDLEGKRDEIKQRVEEERRNGKTTFDVVQNWLNNVDDAIGEANQLQNDPRHAKVGCSRCSFPNVVTRHQLSRKATKIVKNVVDIKGEGNFSEVAYLPALDVVSTSTTRSNKNLESRKSITENIMHALKDPKVSMVGVYGLGGVGKTTLMNEVAQIAKHERLFDEVVIATVSKTPDIKTIQQEIADQLGLHFRDESVACRGKRLHDRIKTERSILIILDDIWARLDLEMLGIPSGSEHGSCKLLMTSRNKDVLQVMDVQKDFKLDVLSEEENWIMFESLAGDTVHDINIKGTAIEVAKRCAGLPLRLVTVARALKNKKLYAWKDALTRLDGSNSGDMDAVTYSALELSYNWLESDEMKDVFLLCSVMRSFLLPEYIVTCAMGLDLFKGINTLGAARNRVYRILDTLKASSLLYEGRMYSCLLYMHDIVREVAVSIAKRDRHVFTTKYGDALKEWPTEGFLRSCSQIVLDNCIINKLPEKLDCPNLLLFVLNSADRFLEIPDSFFEGMKSLKVLELRFINLSLLPTSIFGLISLQTLNLFICVLQSMDGIGALKNLEILSLCCSSMINFPSEIGQLTKLKMLDLCYTGIEVIPQNFISRLISLEEFHMTDISIDWNDETSLDEFRQLLNLTTLRLQVREACILPSDLMFDKLEMYNIAIGDVWEESDIDDGTWKTLKLKLGTNIHLEPGIKGLIRRAENLYLDEVEGISNVLYQLNGDGFPQLKHLHIQNNALIQHIIDFKERTHVPSPSFPNLEKLVIQNLDKMEKIYDGPLANNSFAKLQAIKVENCNKVKYLVKSMSQLSELEVSRCKLMERIVFEDGDASAMNDETVETIQFPLLHSLTLQHLDELKSFFSHQPTSSAIPLFNNQVSVLPSILVSNLYAHTVYYITPTNFEIALNAKVAFPNLDTLKLSSLNLSKIWKDNRHSFYKLTNLIVENCDGPKYLFSSTMVESFSNLIKLEISECHLMEEIIDPVDRDNNIITLEEVRFSKLQTIILKDMKSLKKIWYKEFSKVKTLQVNNCEKIRVIFPSSMQKAYNDLEALMVIDCVSVEEIFQLSSDENCNTERTQLKKITLKKLPKLKQIWSKDPEGTLCFCNLEEIYVETCENLEFVFPCSVATSCSHLKELSIKWCGNMKEIVALKDEPTVSTTSFEFNHLNTLLLWRLYNLKGFYAGNHTLTCPSLRKLDVTLCVKLNLYRTLSTIKSILKL

>arahy.Tifrunner.gnm2.ann1.P9Q3PR.1

MLVSSGNSQRHRQISCPPRMAYRTLPSMAASSSSSSSKKHDVFISFRGEDTRNNFTSHLCAALNQSKIQVFIDNRISKGDENSSSIFKAIRDSNVSVVVLSKDYASSTWCMRELTKILEQKRHGKGHIVVPVFYKIDPSHVRKQIGTYKKAFEKHERDVKHNILLKWKAALTEVANLVGWDSQNYRCYAKTELHISPEVKDLVGIDQNLAPIKSLLRIRSREVRIIGIWGMGGIGKTTIAKALFSKLSSQYEGSCFLENVREESEQHLLNKLLSEALEDANHYFGTPIARSTFVMRRLSRKKVLIVLDDVDNLKKLEDLAAKHNFLGPGSRVIVTTRDKHVLSKGVDEIYEVKGLPLHHAIQLFSLNAFDKSYPESGFVMLSKMVVDYANGNPLALKVLGSFLHSRNLQQWNSALRKLTKFPNTEIQNVLRWSYDGLDDDEQKNMFLDIACFFQGEKKETVTRLLDICGFYADIGIRILQDKALITFSDANELHMHDLIQEMGLEIVRQESTKDPGRRSRLWDPEEIYDLLKNDRGSDAVEGIMLDVSQIRVLNLSYESFSRMTSLRFIKFYMGRGRTCNLFLLSGLESLSNGYPSKSLPSTFSPDNLVVLSMMGSHVEKLWDGVKSLPSLKEINLRACSNLTNLPDLSLAPNLERVDLSLCTSLLCVPTSIKYTNKLLLFNLESCKNLKSLPRNIHLFSLQMFIRKGCSSLDEFSLTSENMTRLDLRGTDIEHFPESLWQLLNQLVYLNLESCAKLKSLTSNIHLVSLQRLNLRDCSSLEVFSVTSENMEYLNLGGTSIKGLPTSIWRNNKLFTLVLHSCKRLVNFPDKPKLGELSLTFNEKNSFEGPTVDEPWNLSSLSDLSLKGSGIVNLPASIKDLPRLKKLTLMDCKKLRSLPSLPSSLEDLSLDERNIDFLPVNIKDLSHLKKLTIINKKRQFSPPELPPSLKDIFLNESKVDSLLLSMKDLSHPQKFPLIKCNWLHPLLEFPPCLEDLSINESNIECLPVSIKDLSHLRKLSLIECQRLRYLPELPRSLEHLSVNGCNIESLPRNIKDLSHLQTITLVDCKRLRSVPELPPCLQSFCAADCRSLEIVPSSKTILMEERIAFYYNCINLDQNSRNNIIADAPFEAAFASLKERTPLGPLISICLPGIEIPDWFSYQTPNSALNIEIPQRWFIDSKFLGFALCLVIGGSHQNSNEGYDPDINCYHFVKPAGNSDPNDRFLGHCTTIMQVPWGFNSDHIFICYYPAFNVPIMQDFKDLAKYYDANSLNLRVIFKFKGPSQRLDLVKKCGVRPLLIANTKRFHIET

>arahy.Tifrunner.gnm2.ann1.PJ1Z6Q.1

MATPQAFPSSSFPSFRDREWEFDVFLSFRGPETRYGFTGYLHKAFCEKGIRTFMDLEDLISGNKIQQTFARAIESSRIAIIVFSEHYADTDFLLRELVKLLECSQRCGQFILPIFYLMDPGDVRHQRGSYEKAMAVHEERDEFEYEFIERIAKQVLAIIKEDSVPVVADYPVRAESHVKALSSFSFPRQYQYHVFLSFRGCDTRYCFTGSLFKALRDNKIHTFMDDVGLHRGNDISRTLIEAIKGSRIAIIVFSQNYADSTYCLDELVKILECQESDGQFVLPVFYDVYHSDVRRQTGSFGAAMAKHEEKFKDDLSKVEKWKRALHRAANFTGFVFDGKKYEHELIGKIVEVVSREIKRVALHVADYPIGLESQVSEVKSLIFSDGYDGVHMVGIHGNGGSGKTTIAHAIYNLIANGFESICFLENVRENSYQHGLVYLQNMLLSNVFERKNLKATSFLQGISTIKHWLQQKKILLILDDVDKPEQLQALAGKPDWFGRGTRVIVTTRDKYILESHGIERIYEMENSNSESNISASDGKDNGDQLGEKNHFPRSSQ

>arahy.Tifrunner.gnm2.ann1.PQX2LZ.1

MRQVYSRALVGIGKSIARVEFLLREEPENVRVIGIWGMGGIGKTTIAEEVYNLLCDEYESVVFLGNVREESLRHGIIYLKSELFSKLLGENLEIYTQNGLPTYVEKRIGHMKVLIVLDDVAESEQLKILVGNPQWFGSGSRIIVTSRDRQVLANYASDNAVYKVEPLDFDEALQLFNLIAFQQNQVEKYRVLAERVVNYAKGIPLVLKTLGHLLHGKDKRIWERELERLGKISNKKVFDMMRLSYDDLDRQEKSIFLDIACFFDGIKLEVNYLKTLLKDGEYQVHAALKRLEDLAFITISKEDVVTMQYIIQEMAWEIVHQESVEEPGKHSRLWNPDDIYHVLKYNQLHLDLLRRFIKTTHNKHPSI

>arahy.Tifrunner.gnm2.ann1.PSU6NZ.1

MAEKLYGGAYLSPFVDAVLDNLTSILEEDDSFLERNNLLVTLQNCLYDVGPVLDDAELKQFSDKKVKKWLVDLQDALYMADDLLDKLSTKAAIAATQRDPGNSSSWSRLVDSYIEDTGDMEKIVGTLESVVRRKNYLLKESAKVDMTWRIPSTCLVEPSGICGRKEDKEAILKLLLDDDDAADGDLSVIPIVGMGGIGKTTLAQLLYHDDQVKENFDFRGWVCVSEEFDVVKVTKTVIEAITSSSCNLTDLNLLQLDLKEKLSRQKFFIVLDDVWNENYDDWNTLLKPFQKGVKGSKILITTRNKNVAFVVQTVSPYELSLLSDEDCCSVFSKHARLSTVSVENLALEKIGRDIVKKCDGLPLAAQALGSILRGNSDIRYWNHLLKSEIWELSNDKINAVPALRISYYFLPSYLKQCFIYCSLYPKDYEFSKDELMLLWMAENFLQTVEKKTIEEVGGEYFDELIARSFLQPHSTKENKFVMHDLVHDLAMTCGGEFYFRAEELRNAVEVDIKARHLSHNAKGNYPMSNLLGVCDRLKHTRTFLEINLNKRIPFNMENVPCIMLSQLKYLRTLSFKSFPVESMPDSMGELIHLRYLDLSWTDIVALPESLGNLYNLQTLKLYSCSNLKMLPVGMKDLVNLRHLDIRVTQLREMPKGMSNLKSLQFLSDYIVGKHEENKIKELGALADLHESISIGKLENVVNSSEALEARMSNKDGIDSITLTWSSNEEENTVDSEMERDILDKLQPHINLKELQIKGYRGTRFPDWVGHSSYHNITKITLVGCRSCCMLPSLGQLPSLKHLEILYFESVGIVGAEFYFYLNGESCLKTPPFPLLESLSFYSMRCWKEWHSLEFNAFPRLKHLSISECPMLRGDLPNHLPSLQSLEISQCKQLSWCVPRSPGMTSLRIKGPNEVRIGELPPLLRELSVAGNHHVESVVEAIMHMQLICLTSLSILGCSSHIWFPVGSIPASLQKLTIQNCRKLEFQMNGQHHSLQELFIENSCDSVTSFSLLDSFPNLVRVNINFCDYMECIVVSRSLPCLRSLCIFGCGRLKYVSTLWMAAPQLEHLTLLGCPEVDLSPTGDGVPHCSLRSLEISYSKKLVSSAAFKNSQFHGLTYLSIHGEYCESVKSLPKEDYPSTQQFQSLQYTG

>arahy.Tifrunner.gnm2.ann1.PT8T2W.1

MAASPSDHDSTALSYFKYDVFLSFRGHTRCEFTDALYHALVNKRIETFRDSEKLRIGQELEGALVEAIERSRMSILILCDEYPTSKWCLDELVKIMECSGNGTKRPVLPVYFRVAKSDVQFQKNKYETAMAAHEEKGRNNHKLEAWRSALSEVGKIYGQRCDQKTAWGEAIDNIVEEVTKRLPPLPLYIDRPLGCDSELEEAKSLLEIGSHVTCFMLGIHGDGDEISKFVAELYNKIRPHFVTASFLSNISEKTNESGGGLEHLQETLLSEMGEEVRTKIGSTFKGSSEIKQRLGRKRVLLVLDDVDSIQQLDSLARRTDWFGPGSRIIITTRYEDVLDDHILNNGVEVKKYCIAEGSSSTVKEENVVGLEKDFEIVINQLKEEDSPGNVVSIVGMGGLGKTTLARKIYNSDEVKMLFPCRAWATVSKDYSGKEVFKSLLKCLKPSASKFEDSSSEEELKQKVKKCLKGKKYLVVLDDVWDSKAWRTIKNCFPENNNGGMILVTTRNDQVAYVSESKKPHHKLSFMDKERSWELFHKEVFCRRNCPPELESIGRSIVETCKGLPLAIKTTAGLVAKRERSEDAWEEIMNLLPYWSVADEDSSEEMMELLKFSYDDLPNKMKPCFLYLGVFPEDEEIWVRDLIRLWIAEGFIEPIQTGRSKSPPQLEDIGEQYLKELVDRNLVQVANRRSDGKGVKTCQIHDLFRELCISESNKPDNNIKSAGVGKSQVLENVPL

>arahy.Tifrunner.gnm2.ann1.Q19R73.1

NKKRLKKQERSKQMENKGSIVLLTTREENVASAVQNCRPYFLRKLSEDYCWSVFAENASFPQSNGRAALEEIGKKIVKKCDGLPLAAETLGRLLRTKHDVEEWNKILMSHIWEFSVEKSKIIPALLISYFHLSPYLKRCFVYCALYPKDYEFEKDELILLWKAEELLPPPKRGESLEEVGCECFDELTSRLFFTKSEDFSDYFVMHDLLHDLAIFLAGDFYCNSEELGKEEEIKIQTRHLFVDLTHCSSKLYNSISKVESLRTLLLFGDFSSANCNIEAATCEILSKCKYLRVLSLTKLDEVPNLIGELIHLRYLDLSWTDIKKLPESLCKLCNLQILMLYGCSKLTTLPSGLHNLVSLRHLDIRKTSLDEMPRKMSKLNQLHVLSSFVVGKHEDNGTQELGRLVNLHESFEIKKLENIVDVEEAKRAKIMDKKHIDELCLEWSSGDDLVSSTQKERDILDNLQPHNGLKYLEIKGYKGTIFPDWLGNCSYENMTHVSLECCKNCCMLPSLGQLPSLKSLSIVGFDQLRSIGEEFYKNEGDHHSSHIAPFPSLETLVFDNMACWEVWHVSESETFPQLKKLEITNCPMLKEEMRNQVFFIIVSSLSDVSKVRTLRIGDYFIERHTEAMFLDGDTLSIRGSESVMESSFKAMISINHLTCLQQIHICECRKLEFPHLQQQKYDLVELLIQHSCDSLTSLSLEVFPNLKNLEIINCWNMESISMSEAPHAALQWLSIVLCEKLVSFAGEGLAAPNLTHLQVRDCEKLEALPRDMKSLLPTLHSLQIYGCPNICRLPEGGLPPNLKELEVGIGEQQMRDLSWMGNLHALTHLSIFGYECKNIKSYPEVGSLPHLPSLTTLHIEHFDNLETLECNELLRLTSLQQLHIYWCPKLENMEGEKLSPSLLLLKIEDCPLLPKHQLIWPKISHIPTIEVASTEVGEYGRRKASFFCYYFKFNTVLCPENATRRRVEAKLFMKGHQEIDNCYGRGSTFFLNSTIIMCISSFCGNCNLVSM

>arahy.Tifrunner.gnm2.ann1.Q1NA6L.1

MGGLGKTTLASTVYKKIRSQFEEHCILCVGEVSREGDHGLVNLQNQLLSHLKPKCMVIKTLNQGKDAIENLLYKKKVLIVLDDVRTTRQLENLAGSRVWFGPGSRIIVTTRDQQLLSSHGVTLFKLYEMKALDTNESLQLFHKEAFKGEKKQEEYLDLSRKFVEYAGGLPLALKVLGSNLCTRSIDEWDEALDKMRKDQDGGIMNILRICYDMLEEGYYKTIFLDIACFFKGWYKDKVIRILKNCGLHPAIGISVLVEKSLITCKKGVLGMHDLLEEMGRMMVMQESKQHLGRSRLSSLDDINQVLKENTGIEKIEGVVLKQQIESYNKDILKDHRAFSKMCNLRLLIILCELHLSRGFKCLPSSLKVLIWVECPLKTLPLGLQLRELVHLQMNNSKVEQLWNGSQVFEKLRVVDLSYSSNLIRIPNISNVPLLEELILDGCVSLVEVDQSVGKHKKLAVLSLIGCIKLETLPRKFEMSSLKRLILCGCSNVKSLPDFGESMEYLSVLNLMKCSSLLSIPETVTNLKSLKILNLSGCSKICRLPDNINENWALEDLDLSETSIRVMDSSLFQLENLKKLSFRGCSGPVSSTTTSDSSVSRLTLPPRIPGLPSLTIFDLSYCNLRHELIPDLRHLSSLETLILSGNTELILSVAKLPMLRFLEAEGCRQTFDPYVLNFHGEAGLLLDFWKLWKLFKTDYNKLLCQVVDHSYPITYQEIPPNFGKEIFFPIGTRLSELESSASITVDIPNECCEGKWWGIAVFLAFKPLESSTNFPSLKFGWSFGASSTDPEVGSSFLYLSSNAVKAHYDCCLVTMIVSENYIYIQLHHRSYDNVSESKPFSKHRKPNFSENSRVRFSVQGEEINMKECGYHVFGNEDFRNKFSFKLNRESTVTPNSSDSNSTEEMDIILEDETETTSLCDDCIDEESKLVSVNSDHMEADIRIKDSTRGRWKKERILSSSQWNKANSTWLQHIHS

>arahy.Tifrunner.gnm2.ann1.Q2Q4I2.1

MTKIVIEKTGSSCHSNDLDTVQNHLKDKLEGKKFLVVVDDVWSSNREGWESFLTPFECGSEGGHILVTTRLDSVASMVKTNHIQPLNLSLLHEEDCWLVFAKHAFFPTESRDRSALEIIGRKIVQKCKGLPLAVQTLGGLLKTKDHEREWIDVLNSDIWEFSEDESSILPALRISYYYLPSYLKTLLCLLFFIPERL

>arahy.Tifrunner.gnm2.ann1.Q5HR98.1

MFIRFLFSFLSHLGTCVIFDNFCKEEDMAAHASSEIIKYDVFLSFRGTDTRCDPSWVRHQKEIYHHALANHEVRFADRVQIWRDALKEAANLSGFHSPSSSFRDDADLVGEIVKRVLQRLNQSPQGDLQGLVGIHGPIAKLVSLCTKSEAVIVGLWGMGGVGKTTLATAVFNRLCDGFEGFCFLNNVRERAEKYGIDHLKKELLSKLLKEEDASPFVTPGGITNFAKKRLSRTKVLVVLDDVNDSDQMEDLCGGHTWFGASSRIIVTTRDKHVLVKADADHIHEVETLNSDDSLRLFSLNAFKQNCTIEAEQVELSKRVLNYCKGLPLALKILGSFLKGKTQREWESELLKLEKMPDEKIQSILRLSYNELDRNDRNIFLHLACFFYTNTEEELIQSVLDSCGYATTIGLTNLHNKALISVSNGYVSMHDLIREMGREIVREESLNDPGKRSRLWDPQDICKVLKDNKGTDAIESIELNMSEIDRMSLHPETFERMAGLKLVRFHAADSKNKLYAPEGIRSMSNELRYFHWDEFPLKSLPPSLSVERMVEIIMPNSGLQKLWEGEQNLVNLRKVDLDGSSSLMELPDLSKASNLREVSISGCKSLCQVPPSAVCSQKLKYLNVSNCESLESIAELPASVVCVIARDCRSLHTVSVSALDSVAEEAIQQQQFEDITFNFSNCYKLEENAKNSIMEHAYGRLKRPLAAAPTCATTAWLDKEDKHPYRYLSGKQHNHPKLNVLSGADPGWFRYINPQNGAVTLDVAPGDSLLGFIFWVVFPAYSGSYQVSATSVSYTYCVEVRNGPSFSYDGHWMPDEDIWLNDYYFWYDGQCCIDMIKVMEENTNTNNNNIQLKVSFKFFYNRLILSQPIEEDDDKEVEYWGVHPIYASDVRGGMSMESVNDVNLNMNVPSIEEGNGGSIDDSDEDDEGSERGGVSELIALMHGALIARLDSIVYPSLYSRCFNLLQPQPPIFIISHQLP

>arahy.Tifrunner.gnm2.ann1.Q7VTCQ.1

MGRPKEDRYWNEVTEQADGTWKCKRCGDQFSKHISVTRIKAHVDRIPGKGISPCSASLHDNHPQPQTANPMEGDGEDHDHEMVDAISGGLTQHPVNVISEPTNYLLPEGFAVVAPSINDGFVSDEIKKLNELRPDLIHEEDGIKGELEWLKSEGKQSKKRVDDWLNKLQQLRNRVVDCLNLVMGAAHNFNENVAHYLRLQIPQLTEEASKLKKEKPVVLSNEFVGEYFEKNVKKMWELVGDEKVLMIGIHGMGGVGKTCLATYMETQIIRKGTFSHVIWVTVSRDYTISKLQEDIAEAIGVKLGGNERTRAAHLSSALSEKGKWVLILDDVWKFIDLQKVGIPLGRINGSKLITTSRLKHLLRQMDCPTLNIITMHPLSNKEGLELFLLRLGERHKTPATLPPKILEIAGIIARECGGLPLAISVMARTMKGVDSIRQWRHALNKLERGEMGGEMEEEVFQVLKASYDNLTHKSMQNSFLHCALYSGLEKDDKMIMKLVDSGAINGRRSLDEILDESHTIFNELQDHSLLLFFPHFWRVKMQDSMRKIACHILKESQRLIVRCGKKLRGIPHVQEWSADLELVSLDKNEIEEIPAGISPKCPRLSTLILSNNCISSIPECFFTHMKSLAILDLSQNDSLTSLPDSLSDLTCLVSLMLHECKALAKVPPLERLQKLSTLVISYTAVEVVLGLEMLTNLRWLDLSFNGKLRLETGSVLRGLTNLQYLDLFDATLSNLEIEDVQGLTTLEYFVVGFYDCKSYNNFVTGICNGSVPKSYLLYLDSIEYNDEIIESYDHIGPSGDHQRIVHLLYGKESPHLLPNDLTNLYIHYNARWESLCDALSNNAPPSLKNIVIGTCTQMKSVFCSFGNCSFCSNLKNLQSLQLYGLESLTVICKEDVGATDTTAQSLKSNAVFSQLRHLEIRDCDGIETLVTAGLLPQLQNLQTLIVESCESLREIFAASSSGANSNDAASPITLPNLTSLYFSDLPMLETLCKGIIISQSFPKLQIRDCPKLESRFPFEVSSSVT

>arahy.Tifrunner.gnm2.ann1.Q8CPXC.1

MAAHTSSEIIKYDVFLSFRGTDTRRGFLSHLRKALEDKHIKTYVDYMLREGTEILHSLLAAIEQSEIALIIFCQDYASSQWCLEELAKILECRKQNGQIIIPIFHNVDPSWVRRQKESYHHAFANHEVRFADRVQIWRDALKEAANLSGFHSPSSSFRNDADLVDEIVKRVLQRLNQSPQGDLQGLVGIHGPIEKLVSLCMESEAVIVGLWGMGGVGKTTLATAVFNRLCDGFEGFCFLNNVRERAEKYGIDHLKTELLSKLLKEEDASPFVTPGGITNFAKKGLSRTKVLVVLDDVNDSDQMEDLCGGHTWFEASSRIIVTTRDKYVLATADAYHIHEVKTLNPDESLRLFNLNAFKQNCNVYDDPGKRSRLWDSCDTYIVLKYSKETQAIQSITLNMYEIDTIKLHPETFERMPELKLVRFHAPDFRRKLCAPEDITSLPKKLSYFHWDEFPLKSLPSSFGVERIVEIIMPDSGFKRFGKVNRYGKRQHCSSFVIPTRL

>arahy.Tifrunner.gnm2.ann1.Q935U8.1

MAEPVPFGIMERLIAELASPALDEIRLKDTLLLVKSVLLDAERKQENNRPLTMLTTSLMIFKPKSRETTSPVVADVNVIGREIDKEFIIDLLMQQNPEDDDERIPVIPIVGTGGVGKTTLAQLVFRDERVIQSFPLKLWVCVSLDFDIQQLIVKIINSASPHLRQLNLKELDMEPLIGRYACWSKVPACLGQCME

>arahy.Tifrunner.gnm2.ann1.QAH2M2.1

MGGLGKTTLARKIYNSKEVKKLFPYRAWSYVSKDYNKKEVLLRILDCLMSSTSKFKDSSEEVLKNEIAEGFIQPHESGTPNAPEPEDVGEDYLVELVDRSLIQVTSIRSYGGVKTCKIHDLLRELCILERKANNSLEVFTESNIHANNTSNPHRLSFLCNAQSYVSSVKPDTRSLSYEVPLSCVGGGFSRLQVLKLEYSSGYNRGVLDGGAIMTPLRCSVMKQCLRVTALPKQLWSLTTLQKVDVVGPSDELEKSL

>arahy.Tifrunner.gnm2.ann1.QC354N.1

MAAKLDGGAYLTSFVDAILDKLSLILEDDSVLEGNYSAQELLGRLEKSLYDVGPVLDDAEQKQFTDKRVKKWLVDLQDALYFADDLLDEISTKAAIAATQREPGNSSSWSRLVDSYIEDSGDMEKVVGTVESVVAKKNYHRLKECAKVDMSSWRTPSTSLVVSSDIFGRDNDKKEIIKLLLDDACHAESPVTAIPIVGMGGIGKTTLTQLVYSDVQVVEKFDTRVWVCVAENSDPVHITRTIIVALDSRPCGMDNFDILQSDLKKRLTGKTFLVVLDDVWHDQRGTWEDFLKPFRDGNKGSKILLTTRSEKVASVFAAPNRHYQLSLLSDEYCWSVFLKHSCISTNSKQYATLEPIGRKIVAKCKGLPLAVKTLGGLLRNKYNVGDWENILESEIWELPEDENKIVPALRVSYHYLPSHLKRCFVYCSLYPEDYQFHKNKLTLLWMAEDLLQPIGNNTLENIGCAYFDELVARSFFQLSSADDELFVMHDLMHDLATFFAGKFFFRVDEFSNPHIADSKTRHLSYNRGRISKFPEAYNGAIYMRTLLPVDVQLNDIECDFGLQQLKCLRVLSFRRFEILSLPDSIGELIHLRYLDLSYTPMVTLPESLCKLYNLQTLKLWNCEKLEMLPSRMQDLVNLRHLDIRGADSLKEMPKGMSKLKHLNFLSGYIVSEQVENGIRELGALDDLHGSLCISNLKNVKDSREALEAKMGNKKHVIILDLKWLREDDDYDDSDDDLKWLREDDDDDDDNQDEDQDEDNEEEEEEEEEEEEEEEDEDEEDDDDDDDDVEDDCGGGETVDVEKERDILDKLQPHRNLKKLSIYSYRGETFPDWLGLPCYSNIIKLILFVCKNCGQVPSLGQLPSLQHLVICGLYGLERIGGEFYNNAESSHQGTPFRSLETLKFVGMARWREWHIPHDFDGFPKLKSLSIANCPVLSGDLPAHLPALEELDIRECPEMDCFGEECLPPSLTTLRIYKCKKLARWIISKGLQSQGLTHLFLSFWNEVKGFPGEGCLPASLKEESRVNINTWLRQVENWGALISKSLKLKEIHRYGNACIH

>arahy.Tifrunner.gnm2.ann1.QE3TA0.1

MAGALVGGAFLSGFINVVFDRFLTMDAVNLVLGKKLGPDLVQRLKTALLGAEALVADAEMKQFGNPSVRKWLDSLRDAVYCAEDLLDTVHTKASTQKEESSSWSLSFFINRDRDDMVDKMEGVVRRIEDLGKQKDFLGLENIPTGSSSWRTPSTSLVKGSVYGREDDKKALLQMLNDNNEHHLSVMAIVGIGGVGKTTLAQWLYNNEGEFMKGFDLKAWVCVSEKFEVVETTKNVIKQIHGSTCSLDDFNSLQTALKGELSNKKFFIVLDDVWSDDGDKWSNFMTPFRQNGNKGSIVLLTTREENVASAVQNCRPYFLRKLSEDYCWSVFAENASFPESNGRAALEEIGRKIVKKCDGLPLAAETLGRLLRTKHDVEEWNKILMSDIWEFSVEKSKIIPALLISYFHLSPYLKRCFVYCSLFPKDFKFEKDKLILLWIAEDLLPPSKRGESLEEVGCECFDELTSRLFFTKIQDDDDYYFVMHDLLHDLAIFLAGDFYCNSEELGKEEEIKIQTRHLFVDLSHCSSKLYNSISKVESLRTLLLFSNIFSSPNWNIEAATCKILSNLSLTKLDEVPNLIRELILLRYLDLSLTDNETLPESLCNLCNLQILMLYGCYKLTTLPSGLYNLVSLRHLDIRGTFFEEMPGKMSKLNQLHVLSYFVVGKHEDNGIQELGGLVNLHGSLEIKKLENIVDVKEAKRAKIMDKKHIDKLWLEWSSGDDLVSSTQKERDMLDNLQPQNGLKELKITGYKGTIFPDWLGNCSYQNMTRVSLECCKNCCMLPSLGQLPSLKSLSIKSFDQVRSIGEEFYKNEGDHHSSHIAPFASLETLVFDSMPCWEVWHVSESETFPQLRKLQIRNCRMLKEEMLNQVFFRIISSLSDVSKVRKLLIGNNFMKRHIEAMFLDGDTLSIWGCESVEDSALKAMISINHLRCLQEICISRCRKLEFPELQSHKYDLVELKIQDSCDSLTSLSLDVFPNLKNLKIEGCRNLESVPMSEAPYAALQRLIISGCSKLVSFAGEGLAAPNLTHLQVAYCDKLEALPRDMKSLLPTLQSLQIYGCPNISRMAEGGLPPNLKSLGVGGCEEQLRDLSWMANLHALTHLIIRGSGCDNVKSYPEVGSLPHLPSLTTLEISWFDNMETLECNELLRLTSLQQLHISECEILENMEGEKLPPSLLLLKIERCRDTFEDQAAIVLRALQFWNGS

>arahy.Tifrunner.gnm2.ann1.QFLR8D.1

MATDALLGILIGNLNTFVQNEIAALSGVDSQIQDLSENLSAIRALFQDAAEEQFTSHGMKDWLNKLSDAMHVLEDILEECSMESNRLQSGGWLARFHPKTILFHHAISKRIKDMVKRFQRIEEERRRFQLPVRVTQRQQEDDDQRQTISSIPEHPMYGRVKDKEKIVDFLTKHACCNDGVSVYPVVGMGGLGKTTLCRWVFNHDKVIQHFDLRIWVCVSTNFNMMRILEYILESTTGQNPNLSTLEAMQNKVQQVLLGKRYLLVLDDVWDNVKWEDLKSVLNCRGSGIKGVSVLVTTRDPSVASVMTPCNSHHLQPLTENDNWSLFTHYAFGLNNQQPAKLVEIGKEIVRRCVGNPLASKVLGSLLRNKKEEKHWLNVLESKFWDIDDVMGALRISYFRLKPASRQCFSFCAIYPEDFQISKEQLIHLWMANGLIKSRGNLEVEDVADEVWEELCQRSFFQEVRIDVYGNTTFQIHDLFLELIQSVVGEECKVYDKSASLINLSRRVHHISYSGFLEELNQSTFKNVESLRTFINLDQPKGNFLTTQTLILHDMPICNSLRALHTRSSQLSTLKSLTNLRYLNIYDSYITTLPKCVSRLQKLQILKLEHCRYLTCLPKHLTKLKDLTHLIIERCWSLVSMPPKMGELKHLKTLNTFIADSKSRHCLAELHDLQLGGRLHIKGLKNSVRERWNSMLTIPSNASDLMIRKCFFMNGNQFELWDVSNGLPILSCYFGASLLQLLMAPINR

>arahy.Tifrunner.gnm2.ann1.QIQ4VJ.1

MAESLLQMVIENLQAFAQDELATLWGVHSQIQELSGNLAAIHAVLQDAEEKQIRERAVKLWLQKLSDAAHVLDDILDECSIESNRLHSEQCLTQCLTRLDPVTIMFRRDIGKRMKEMVDRFRQIDEERRRFELRGRVPERQQEDEAWRQTCSVITEHNIYGREQNTQNIVEFLSRSADSSNDLSVYPIVGMGGLGKTTLVRWVYNDNKVIEHFDLRIWVCVSTEFNTMRILESIVESTSGHNPNLSTLEAMKNKVQEMLLGKRYLLVLDDVWSTDKWEDLKSVLLCGGGTKGAAVLVTTRVESVASVMGTCPAHHLSPLSEDDNWLLFKYHAFGSNKVERTELVAIGKEIVKKCGCSPLASKALGSLLRNKKEEIQWVNVLESKFWDILEDDAIIVHNALLFVPFSPKIFRMEKQQLIHLWMANGLIKSKGKLEIEDVGNVAWEELCQRSFFQEVEIDELARTTFKMHDLFHELAQSIMGKECRVYDESASLTNLSTRVHHVTCLKPEMESLTHLRYLNLRSSCITTLPECVSRLQKLQILKLEDCLDLSCLPKHLTQLKDLRHLLIEECSSLVEMPPNIGDLKCLRTLNLFIVDKKEGHGLSELRDLQLGGKLHIKGLENVINEGDARDANLSAKKNLENLYLSWDSSDSRCGANAERILEALEPPSNLKSFGMNDYPGVELPSWMQNTSILSSLVMVILCDCKNCKHLPPLGKLPHLTVLYVSGMKDVKYIDDDSYDGVDEKAFKSLKHLTLSNLPNLEGMLRDERVEMLPVLSKLKVSCVPKIKLPLLPSLEYIWIEGTGSDSDHSDSEGMASILEAIGQNMQHVKTLCISSFDKLKALPHELSSLSSLQKLEIYGGDELESFSKNVVQGLCSLQSLTIHSCKKLRSLPEGVGHLTRLESLDIMICPKLVTLPSSMNKLVSLRRVCIAHCDTLPEGLQHVPSLQSLEVYESNSIPEWLGEITSLQKLELTRVRLRSLPSSFRNLTNLRELSIDGCHKELQKRCTRLTGQDWQAIAHIPQFKLVPIHEETFSDPSKWRSWQLRRDRRRRTQDDILFAINMLNQQPLPFRQSSRDDLWMGFSLWGEILAQVIAIATCGLIFAECDKFIWFENPKPLIFAKRNMSSAIRNITLVMAKKLYKKYLIANRICLFHRERYCKQEIRIFLASNYGKGKEDHA

>arahy.Tifrunner.gnm2.ann1.QMVD72.1

MSTILTKHNTAKKNIHTTKHNLLLHLPIYMAMASLAAEASSFSTPPSPRSWTYHVFLSFRGEDTRTGFTDHLCASLERKGITTFRDDKDLERGQVISLELLRAIQESMFAVVVLSPNYASSAWCLDELQKIVECKHNLGLQIVPVFYGVEPSDVRHQKGTFAEAFRKHEHRFGEGSEKLRRWRDAFTQIASYSGWDSKHQLEARFVESISEHIHRKLIPKLPSCTKNRVGIASRLEEVINLIGIGLNDVRFVGIWGMGGIGKTTIARAVYEAIRGEFKVCCFMRNVRELSAKNGFVQLQRDLLACLNISSYFHDIEDGKTTIKSALCNKKVLLVLDDVSELNQLENLAENQDWFGQGSRIIITARDMHLLDIHGVHGTYEVKGLDQEEAYNLFCLKAFKQLEPKEGYSSLCKEVVKYTKGLPLAVEVLGSYLYRRNADFWHSTIREIMNFPHFEVLNALKISYNHLMTTEKNIFLDIACFFKGMKKDEAIHILRMCDFYVGVGSDIGSGIVTLIDKALVTLDQNNKLEMHDLLQEMGRHIVYEESPSNPGKRSRLWSKDDIHQVLTNDLGTEAIQSMVLNFGHDKFYWFRSKPFSAHWSMEAFSKTTQLRYLSLPYMELPLGLNHFPSSVRVLHWDFCPLETLPLLNQQYQAVEIKMQRSNLEQVWHGKKFLEKLKYLDLSSSRNLKQTPDISGVPILETLDLQGCDSLTEVHISLIHHKNLVHLNLSYCEMLKTLPGKLEMSSLKELIIEHCQSFENPPEFGECMRKLSRLSLSGTPIGKLPSSLGNLVGLEDLNIKGCGKLDSVPDTIHRLKSLKNLDLGSCFNLHGLPSSMSSLPLLSNLNLSGCYQSEISFSHDLFCYLPSLMHLDLSGHWFANIPISIHELSKLRSLKLNGCRRLQFLPKIPSSIRELEAYGCRSLNIFESNVLSTIFTAFKYSSGQDQENQGVVLEMLIPSTEIPSLFGHFPLKDYHSAIVPYPSVCRWIKNIKGIAVCFLFYTKFWGFDKSVKLNLSVSNGNRCIIPWRTYRMCDGYHLYILCLTNDYFGEEFQQDMVFKLLLRPEVEYGEYDSEEFEHIPCYQAKVLSTGLACINEIEDLNQSEIERQRNEGQSLFDLNKSIEIMDICERINFES

>arahy.Tifrunner.gnm2.ann1.QP8IW7.1

MFQNPRTLACKMKYIGLIVCVIAICIAIIAQKFDAETPVEKNTPPSYPEASSPTHDTKFEVFISFSGKDIREGLLSHLTKALRQKQIFTFVDTKLEQGGEISQELLQAIEKSLISLVVFSENYAFSTWCLDELVKIMECRREKGQIVLPVFYRVEPTHVRHQKGVFSTAFAKQERRFGKEKAQTWRSAFQEAANISGFHSAKFGNDAKLIEEIIQSVNTRLKNMRQFSSKGLFGIAKSISRVESLLRQEPESVRVIGIWGMGGFGKTTIAEVVYNLLRDEYESVVFLRNVREVSLRHGIIYLKNELFSKLLGENLEIDTQNGLPTYIETRIGRMKVLIVLDDVNQSEQFEILVGTPQSFGSGSRIIVTTRDRQVLAKYAHANDTYKVEPLESDEALQLFNLIAFQQNEVVEKEYSVLAERVVDHAKGIPLVLKTLGHLLHGKEKWIWESELEKLGRIPNKKVFDMMRLSYDELDRQEKSMLLDIACFFDGMKLKVKYLESLLKHGDFPVPAALKRLEDISFITISKEDVVTMHDIVQEMAWEIVRQESIEDPGNYSRIWNPEDIYQVLKNNQGSEAIRSINFSYSKATVRDMQLSPQVFSKMSKLRFLDFYGEQHLLHFPEGLQQLPSRLRYLRWTYYPLKSLPKKFSAEKLVILELPYSQVEKLWYGIQAPYSSQLKEFPDLSKATNLEILDFKYCLRLTRVHPSVFSLNKLETLDLSWCSQLARLETNAHLKSLRYLSLYHCKRLNKFSVISENMTELDLRHTSIRELPSSFGYQSKLEKLHLANSEVKKMPDSMKLLTSLKYLDISDCKNLQTLPELPLSIETLDADNCTSLKVVSFPNASEQLKENKKKAVFWNCLKLENQFLNAVALNAYINMVRFSNQYLSTIEHDNVDNSNEDPEASYVYPGSKVPNWLEYQTNMDHLTVNLSSAPYAPKLGFILCFIVPAVPSEGFRLMFTISGDDQEEDDVNEVRLYVDRPRKEISWDHVILIYDQRCSSFLNNRGQNRRMFNIKVSVVSLSMTSEYVAVELKGFGVHPVNPLEYPSFISFIKRMEQLGYYTTPATVNPVSRWNGIRSWFGAHNWG

>arahy.Tifrunner.gnm2.ann1.QVS58C.1

MGFALPTVDYEELQSRVETMNEITKALEDSSATMIGIHGLAGMGKTTLVIEAANRVQNRESKVFDVVIMANVGKILDIRKTQGQIADMLGIILQEESEYARAIRIKEKLKKEKNALIILDDVYAKIDLDMLGIPSQSPDDKQKNLLLKEGKSFSNVDQTKARQTKPMDPLL

>arahy.Tifrunner.gnm2.ann1.QXFT5L.1

MSSQNQEYDAYISSGLQFFHPFISDLYDELKNVGLHVLKDRTEPKTNIYGAIERCRASIIVFTIDYAESTLYLQELVKIMECHRRKDQNVVPVFYCLDPSQVCNLSGHFGEILSYTLQGTDENMMLSYATALRQAAFISPRNDREVMSHIVGHVTSLIDSTKLFIEHPVGVESRVQDLIQLLNNKKADGVLIIAIWGMAGIGKTTIAKALYNQISHNFEVRRFVPEIQGREADYLRELLLFLKDQVKTKAHNFDSTRKIWWERLRCLKVLLILDNVRSERELEVLPVPVECFGPGSIIITTTRTKHGVNHIYRVKEMDYNECVELFSWSAFNKATPERSFSGLINYAIEYSDGLPLALVAVGSAVSEKSIEEWENVLDSFKRFPFQDVWQVLKENIDSVGSEEKKIFLEIAYWRHLFIGKNRNDISQILQEAGRNAAPRAIKGLEEHSLVWFDEDKLCMNRLLQDIGREMYMKESSIEPQQRPYDVFLSFRGKETRSKFISHLYASLENASLEPQFIYDAYISSGLQFRHPFISGLYDALKGVGLYVLADRTEPKTNIYGAIERCRVSIIVFTIDYAESTLYLQELVKIMECHRRKDQKVLPVFYCFDPSQVCNLSGHFREALSLTLQGISTDENRMLSYETALRQAASILPRFLPDICNNREVMSHIVEHVTSLMDSAKLFIAKHPVGVESRVQDLIQLLNNKKADGVLIIAIWGMAGIGKTTTAKALYNQISHNFEVRKFVEGIQDVKGDYPIWLASKFIEERLLLFLKDQVKTKAQNFDSTTNIWKEGLRCLKVLLILDNVRSERELEVLPVTTECFGPGSIIIITTRTKHDRLHEIGVNHIYRAKEMDYNECVELFSWSAFNKATPERSFSGLINYAIEYSDGLPLALVAVGSAVSEKSIEEWENVLDSFKRFPFQVVWQVLKENIDSVGSEEKKIFLELAYLKHLFIGMNRNDISQILQEAGHNAAPRAIKGLEEHSLVWFDEDKLCMNRLLKDIGREMYMKQSSIEPQQRPYDVFLSFRGKETRSKFISHLYASLENAGIYVFKDENGLARGEELSISLLKAIGESKTSIIILSPNYAFSRWCLQELEDIMICCKNKTQKVLPVFYHIDPSEVRNQTGKFGQAFDNLMRRYPDKIKGKEQSWRKALREVGCIAGFVIRKSKNESEDIKNIVEQVTHMLEMKELFVANHPVGVESRVEEVIQLLKDQQQENPLLLGIWGMGGSGKTTIAKAVYNKIFREFEGRCFLLNIREVWDQDNGILHLQQQLLSAIYKTTKIKIENTESGKSILERRLGQKRILLVLDDVDKLEQLNSLAASRKWFCPGSIIIITTRDEHLLRCLRIDKKVYEWRSVLNKLKTIPNNDVQKKLKISFDGLSDDRDREIFLDVAFFFIGMDKNDVIHIINGCGHSAEIGINVLMERCLITVDTKGKLGMHGLLRDMGREIIRESLPMKPEERSRLWNPDEVLNVLSKDMGTKAIEGLALNLPKSLNPTQLKTEAFKEMKRLRLLQFANVQLVGDFKYLSTDLRWLCWHECPSEYTTANFDQGNLVAIDFKYSKLDLVWKKGQMMRNLKILNLSHSQHLTQTPDFSNMPNLEKLILKYCPKLTSVSHTIEHLKQVLLINLKGCSGLRVLPRSIYKLKSLKTLILSGCSSIDKLEEDIEQMESLTTLMADKTAITQVPHALLRLKSIVYISLCDFKGLSRNVFPSIIWSWTSPTNNFSPQVQTFLDLSNLVSLIVPNSNSQGLSSIIRELPQVQNVRLECGSQLQIIGDVVSNTFDVTNCNEMKVTSSASNISKGSSSSLIGYCSEGDIIESENSLNSILIQMGMSCSVTELLRENIFQKFNARVPGDCLLPGNKNPDWLTFSCEGSFVIFDIPQVNGHKLKSVMLCIIYSSSSNIVPLEGPIIKNLCIINHTKTTPFLYDGDTLASLRDDEWQKVIANLEAGDKVQIVVASGLRFTVKKTAVYLIYAEQQAEGIVCGDDMVADGNIIVHDDEENDLLQGVNKNFSKKNLKSHTKRKFKEYDDTNH

>arahy.Tifrunner.gnm2.ann1.QYBR1Q.2

MAAEAVLSSVLSVVFDRMSSPEVVNWIKGKKLTLKLIERLKTNLYAVQAFLIDAEQKQIKERPVKAWLDSLKHAMYVADDLLDEVFTKAATQKDPGTFLSRLLNLQDRDVANKMEEVIDRIESLVIQKDTLGLREIPKENMSWRITTSLVETSDVCGREEDKEAIVKLLLDDDSEDATGGHSDVSVIPIIGMGGIGKTTLAQLVYQDGKVKENFDFQAWICVSEEFDVFKVTKTIIEAITSSFCNLTDLNLLQHGLKEKLSRKKFFVVLDDVWSESYDDWNKLLKPFRKGVKGSKILITTRSKRVASVVQTVSPYELSLLSEEDCCLVFSKHARLSTGSMENPTLKKVGRDLVKKCDGLPLAAQSLGGLLRGNSDIKYWNHLLKSEIWELSDDKIKVVPALRISYYYLPSYLKECFVYCSLYPKDYEFSKDELILLWMAENFLQPAGKKTPEEVGDEYFDELIARSFFQPHNFHENKFVMHDLVHDLAMIFAGEFYFRAEELENAVELDIKTRRLSHNAKGNYPISKLLGVCDQIKHTRTFLGLNLNSQIPFNMENAPCILLSKLKYLRALSFNCFPLESLPDSIGELIHLRYLDLSWTYIMTLPDTLCNLYNLQTLKLVGCRKLKALPVSMKDLTNLRYLDISGTGLDEMPEGMSKLTSLQVLSKYVVGKREGNKINELGTLANLHQTIFIDRLENVVNSSEALEARMFEKDGIECLLLEWSPDESENTVDSQIERDILEKLQPHSNLKQLQIWGYRGTTFPDWLGHCSYHNITQITLGGFFPGYFKNCCMLPSLGQLPSLKHLEISKFERLAIVGAEFYRNDESCLETPFPMLETLRFYSMSCWEEWCSLEFNAFPRLRELIIWDCPMLRGDLPNQLPSLRSLTIQNCEQLSCCVPRAPAITSLRIEGSNEVRIGELAPLLDILSITGKHQAESVIEAITQTKLTCLRSLSISGCSSHVLFPVSSIPASLQELTILDCKKLEFQMEGQHHSLHKLMIHNSFDSVTSFSLDSFPNLGRVEISKCEKMESLVVSRSLSCLPYLEIENCGSLKSLKTQWMASPQLEDLGLLGCPEIDLSATEDPHRSLRSLTIGYCEKLLSCAASQFHGVTHLCIEGENESVKSLPKEAFPTRDNFCSYGFAFDASLTINHCNGEEF

>arahy.Tifrunner.gnm2.ann1.R4GVP5.1

MAEALVVGALVSGSINLVLNRLISPEFVNSVVSKKLDRKLVERLKAALLAAGALAADAEQKQFGNDRVRKWLDSLRDALYTADDLLDRVFIKAEIRKKVRVRLPLRLDLSVSKMVTKINEVIKRIEDLQKLKDSLGLKEIPTGSSSWRTPSTSLERGTVYGRDGDQQALIQMLNDNNHHSLSVISIVGMGGVGKTTLAQCLYNNKDLMDGIDLKAWICVSENFDVVETTKNVIKGISSDADKWNSFISPFQHGTKGSTILLTTRNENVGRLVQHYNSYTLKELSDDYCWSIFAENASFHESNGSSELEGIGRKIVERCDGLPLAAETLGRLLHLRVSC

>arahy.Tifrunner.gnm2.ann1.R8GYE0.1

MHSTLIRLKSELSKSTHGKKVDEGMKERLLTVCNILLGVAEDAEVKPENEGLRSRIEEVKEKCYDVMDLLEEVKVARVELCFCNPVPPSRRRLKRMVEEFEGLSRHRDLFRSGVVDFFKLGPVADMETGFQPRSHALYGRDYETEFLWIKLFDPFGNNNSFEDIRVISVVGAPGIGKTAVAKYAFQFPIVRYHFEIRAWISASEDLDLIKFLKVFIEIFTNDVVYNVETEVLQLRFRQIVEKKRCLVVLDDVSQENPQHWLHLKKLFKFCDPRSRILITTRNQEVAHNAGSLSTDILELRGLSEEACLSIFKDCALVAPEAKIPNELRSNITNICNGSPLNAESLGRFIYRRPVEEWPEALKGIDIIHKLSYYPGQSTFFSVSPILRQCLLYCCIFPKNYSIDADTLIKLWMAQGFISSSDQDEEDMEKQAWRCIKELLDRYIFEESKIDDNGNFKFRLEGRMGDFVKFYVGGEYQSRFLDYYYDKEPTQKGLITRHYTLNVGVQASLPESKRLRALHLSSCSIKELPQNIAHLLHLRYLDLSFNHDLKKLPKAICSLLHLQTLNLNGCDSLQKLPKDIGKLVNLLYLEILWTTSLSYLPKGIATLTMLRRLNRFFGNSSANNSKACNLGDLENLNKIRGHITIDGLGAEADVSGAKKASLKNKKDLLGLELWFSFVGSQRKDEAVLEALEAPPGLQGLGIFYYQGESFPNWMTALQNLTHVMLADCSNCSDLPPLGKLLALQSLTIKNMSKVNMVGSGFLGIQLNNAAAGAESSGSSSSQAFPKLQELCFEKLNNWENWIGIGDDGDCAKVIMPSLSSLSIINCSKLRTIPNYIKLKTNLSGGIKRCPSLDLSQHQLSSIFQGTTTTDKVRSS

>arahy.Tifrunner.gnm2.ann1.RBTF4J.1

MAPKLYSGAYLTSFVDAILEKVSLILKDDSFPERKILLRKLEKSLYEARPVLDDAEQKQFTEEIVKKWLLDLQDALYMADDLVDELSTTKAAIPTTLRDPGISSSLGSFVDSYIEDTGDMEKIVGTLESVVAHKDLLCLEKSAKVDMPRTPSTSLVVSSDIFGRDKDKEKIIKLLLDDTLHAESPVTVIPIVGMGGIGKTTLAQLVYNDDQVQQKFNVKAWVCVGEVFDVLKVTKTVVENATSASCNMNDLDSLQQRLRNEVTGKRFLVVLDDMWTNHYDDWKTFLGPFQCGSQGGKILVTTRIDTVASMVKTIPAHNLSLLDNEQCWSVFENHAFFPTDSRDRSALKEVGRKIVEKCKGLPLAAKSLGGLLKTKDNVSDWEDVLVSEIWEFSEHECGILPALRISYHCLPSHLKRCFVYCSLYPKDYEFEIDELILLWMAEDLLQQPKSRSTLEQLGHKCVNDLVARSFFQPSKNAYKNSFVMHDLMHDVATFYGEKFFVRICEHENVAQHDTKTRHLSYDVSDNNSVPKMLEACESLSHLRTLFQIEAYLYRGHKEGIDPCGLLAQLKRLRVLSFASFKIDRLPDSIGELIHLRYLNLSNTLVVTLPESLNNLYNLQTLKLRNCEKLKKLPSNMQDLVNLRHLDIAGTDLEEMPKKMSKLKDLQFLSGYIAGKHEENGIGELGELAHLHELLFIQKLENVKNSGEASNARMDEKIHLNALELWWSSYEESEVCDSQSEKDVLDKLRPHKDLKMLRLSGYRGTMFPDWVGQSSYHNITELYLRECRNCWVLPSLGQLPALTRLGISDCEKVKKIGGSFYKGDGTHQHQETPFRSLKILVIQTMPCLEEWESYECDDDDDAPFPKLEELSIEDCPKLRGDLPTFLPSLKSLYIRGCEEIGCYLPRAPILRDLRICGKQEARMRELPLSMLRTLWVNGEQQVEYVFEAMTHTQPTSLIHLHISECSSAISFPGDSLPPSLQDLRINNCKNVEFPMQHQQHHSLQTLEIHNSSTFAPPCLVLHPNGRSFITFLGVAPACLPVPFVGFARNKLKQSWSKLNLESRGTWSRMISRGVVSASIRKLNSWKTPSSYKIKRRSK

>arahy.Tifrunner.gnm2.ann1.RE92TF.1

MRSFFDRALIAISHNCVRMHDLIQELGWDIVCQQSSENPENRSHLWDSNDIRDVLGNNKGTDSIESIILGVGCLPFAISPLNFCPEKLVTLRIWNSKLKRLWDVVQNLVNLEKVDLTDSQKLVELPDFSKADNLKS

>arahy.Tifrunner.gnm2.ann1.REWL7K.1

MAGSSSDHDETPLSYFKYDVFLSFRGHTRREFTDALYHALVNKRIETFRDSEKLRIGEELEGALVEAIERSRMSILILCDEYPTSKWCLDELVKIMECSGNGTRRPVLPVYFRVAKSDVQFQKNKYETAMADHEEKGRNNHKLEAWKSALSEVGKIYGQRCDHKTLPPPPLYIDRPLGCDSELEEAKSILEIGSHATCFMLGIHGDGDEISKFVAELYNKIRPHFVTASFLSNISEKTNESGGGLEHLQETLLSEMGEEVRTKIGSTFKGSSEIKRRLGQKRVLLVLDDVDSIQQLDSLARRTDWFGPGSRIIITTRYEDVLDDHILNNGVEVKKYCITEGSSSTVKEENVVGLEKDFEIVINQLKEEGSPGNVVSIVGMGGLGKTTLARKIYNSDEVKMLFPCRAWATVSKDYSGKEVFKSLLKCLKPSASKFEDSSSEEELKQKVKKCLKGKKYLVVLDDVWDSKAWRTIKNCFPENNNGGMILVTTRNDQMAYVSESKEPHHKLSFMDKERSWELFHKEVFCRRNCPPELESIGRSIVETCKGLPLAIKTTAGLVAKRERSEDAWEEIMNLLPYWSVADEDSSEEMMELLKFSYDDLPNKMKPCFLYLGVFPEDEEIWVRDLIRLWIAEGFIEPIQTGRSKSPPQLEDIGEQYLKELVDRNLVQVAKRRSDGKGVKICQIHDLFRELCISESNKPDNNNNNARRLSFPGDTGSYSCLLTCNQSCTCSLFVYGDDTQEWPHHIPEDCRVNVIYFADGFTDLNEYFKELKSLKFLKMFYFESYELCKLQSLKTCHVMMIIEKDLSIGRLKQLRHLRCENGLNLSVDEEVVKDKMQNLQTLCYVSADSQLGSLLDNGCFPNLRKLGVSINKDQGSPEENLRSLHCLNNLRKLVLMFEECWVPLDRIAFPSNITKISLSSFKDLKSKDMNTLGQIPSLQILKLRFGSCEEETLNCGAAGSFLRLQVFIMDGVNITCLTSEEGVMPRLRRAVFYNCPDLKEVTKQMRSLGSNLEFIEYDVNHGLSLG

>arahy.Tifrunner.gnm2.ann1.RL0N5R.1

MASKLYGGAYLTSFVDAILDKLSPILEEDDSFLERNNLLRRLEKSLYEAGPVLDDAEQKQFTDKKVKKWLVDLQDAFYKADDLVDELSTKAAIATTLRDPGISSSCSSLVDSYIENSGDMEKIVGTLESVVAKKNHHGLKECVKVDMSSWRTPSTSLVVSSDIFGRDEDKEKIIKLLLDDSRHAESPVTVIPIVGMGGIGKTTLAQLVYNDDRVQQKFNVKAWVCVGEDFNVLRLTKTVIEEVTSKSCELNGLNSVQQRLRNEVTGKWFLVVLDDMWTNHYADWKTFLGPFQFGSQGGKILVTTRIDAVASMVKTIPAHNLSLLDDEQCWSVFANHAFFPTESRDRLALEKVGRKIVEKCKGLPLAAQSLGGLLRTKGNIADWKDVLMSEIWEFSEDECRILPALRISYHYLPSYLKRCFVYCSLYPKDYKFDKDELVLLWMAEDLLQQPKGGSILEEVGYKYFNDLAARSFFQPSKNGYEYSFVMHDLMHDLATFYGEKFFVRISEHENVVQHDTKTRHLSYDVNDNNSVPKMLEACESSSHVRTLFQIKADLYGEHKEGIDPCRLLEQLKRLRVLSFTSFKIDMLPDSIGELIHLRYLNLSGTLVVTLPESLNNLYNLQTLKLENCRKLKKLPSKMQNLVNLRHLDFSGTDLEEMPKKMSKLKDLQFLSFYIAGKHEENGIGELGELAHLHGSFCIEKLENVKNSGEASNARMDEKIHLNALLLRWSSFEESEVCDSESEKDVLDKLRPHKNLKKLVIRRYRGTMFPDWVGQSWYHNMTELELSRCRNCWVVPSLGQLPSLERLVIAGFDKVKKIGGEFYKGDGTRQHQETPFRSLKYLSIKRMPCWEEWESYECDDDDDAPFPKLETLEIWDCPKLRGDLPTFLPSLKSLLIFRCEELGCYLPRAPILLRINIFGKQKARMRDLPLSLQLLRIEGKQLVDSLFEAMTHTQPTSLIDLHISECSSAISFPGDALPPSLKYLHIDDCKNVEFPMQHQQHESLTRTYIDSSGTCQFIAFGCCASNLVRMNDLKSFLCTNSFLVEYMDKELCIYLNFVVSGRNFKLEWTALRVLLLHHPFTHPNLRPFITFLGVALACLLMALVGLQETNRSKLGLSLIRRPKESGQE

>arahy.Tifrunner.gnm2.ann1.RX2NL5.1

METPNFEGSPSLKRLDFRGCTNLVRVDSSIGLLKELAYLSFRNCCRLVILDLDVGCNYDCKKPETKPDFTGVGLPNLEHLDLGECTSLSTVHYSIGTLKLKFLNLQGCINLVEKPDFSVFLRDEPENEVIDHSQVGIRSNLAVLENPGSSQRLSQEYSASDLAPQKSKSIMTEGSSSSPRAYKYDVYLSFRGADTRKSFIDHLYHRLMEEGVFAFKDDVHLDRGESISSEPLQAIKGSRISIVVFSKGYASSTWCLDDLVAIVDCHKEMKRELLPVFYDVDPCDVRYQIGAYEDAFDSHRERFKEEPDKIYKWKSAMTYLTDLRGLILRDRAEAEGIERIVEAVRDTLVYKFQISRSTHKNPESTERRPSLQESNKYDVFLSLRRTHTPYTFIDYLYHYLTKKGFSTFKSEKETERGDSIPSQDLQVIKDSRILIVVFTRDYADSTCSLEEMATIVDCHWELNQTVIPVFCDVHPRDVRRQTGPYEKAFVSYTKEFKQHPLKVQKWREALKPEIEAIGNIIQTVERLCPKFSRLSSHSPVGIQSSLAMLEELLKLEDDCVLVLGILGMGGSGKTTHALALYDRIVQEFEGACFIQDVSQVYRVGGATALQKQILCQFFIEELSDMYSPFEMSGFLRRRLYGKKILIVLNNVDVPRQLELLAINRNMLGNGSRIIITTRNRHILTAYGVDEIYEIPLLNKAQARELFLSACPDATNYEVYTELILRVLEYADRLPLAIRVLASFLRSRDVTFWEDTLDRLDRLTRVSSFEIIKILQISFDGLDQEEKEIFLHIACFFHGKKLDFVMEVLNCCGFFAGIGIQALMDKSLIIKNEEEIHMHDLLQEMGKEIVRQECPENPASRSRLWRYEDFSSTLESKNEASKVKAIVLYEDTSISKHEDLNIEGLSKMTNLELLILYNQHFSGKLTSLPNKLRYLLWDGYPFSSLPSLEPYNRLVQLNLPNSRIKRLWNGSQVFRSLERVDLSYSKELKETPNFEGCPSLKRLDLTGCTNLVQVHPSIGHLKELAYLSFRDCDKLVTLNLDHKCKLSSLKVLDLYSCKNLKNTPDFTGLPNLEHIDLGKCEGLSTVHHSIGNLEKLKFLNVPADIDLPELGGVRIIRTP

>arahy.Tifrunner.gnm2.ann1.RYW3P7.1

MAEALVVGALVSGFANVVLDRLISHEFVDLVVGKKLDRKLVDRLKTAILAAKALAADAEQKQFGHELVREWLDSLKDALYTADDLLDRLFIKAQIRNKTRIHRPHFLDLSGRKMVTKIEEVVERIEDLEKRKDTLGLKEIPTGSSSWRPPSTSLVKGNLFGRDGDQQALIKMLNDNNDHQLSVISIVGMGGVGKTALARWLYNNEALMKEFDPKAWICVSENFDVVETTKNVIKEINSGDCSLDSFESLQKDLKKRLSEKKFFIVLDDVWSEDADMWNTFITPFQHGRKGSTVLVTTRKVNVGLTVQHYNSYTLKQLSDDYCWSIFADNASFPESNESSELEGIGRKIVERCDGLPLAAETIGRLLCSERRVEKWNKILSSDIWEFSVANCKIVPALLLSYDHLPTHLKGCFVYCSLYPKDYQFDKDELILLWMAEDLLRPPKRGETLEEVGCECFDDLTSRLFFKQVENDDEKYFVMHDLMHDLATFLAGDLYCRFGEKEDMSILTRHLSYNHSTPEKICSSSKSESLRTILYINDGDCFLKARATLPCDILLKNKYLRVLSFDTLNIFPDSIAKKLIQLRYLDLSSSDVMILPESLCNLRNLQTFKLEGCLELTMLPSSLGDLINLRYLDLSWSSVKILPESLCNLCNLQTLMLEGCSKLTMLPNGMYNLVNLRHLNIRGTPLKKMPKGMGKLKQLHILSKFVVGKQEDNGIQELGGLLYLHASLEIQNLENVIDGNEARSARIIDKKHIDELLLEWFLPSGDVMASDAQTKRDILHSLQPHNGLKELRIRGYKGTIFPDWLGHSSYQNMTSVSLQYCRNCCVLPSLGQLTSLKSLRIERFDQLKSIGKEFYKNEGHQHSSPIAPFPSLETLVFDGMSCWEEWSLPDSEAFPQLKSLQITGCPMLNGDMVNQVLMRIVSSSSDVSKVRQLEIQEQRESWENKEMTLDGDSLTISGFECVAECAFKARIIHHLTSLQEIHLSWCSSVVSLGGNCLPKSLRKLTILGCRQIELLQQQHKYDLVHLQIEFSCDSLTSLSLDAFPNLQNLEIFRCSNLESVSMSEPPHAALQRLTISQCYKFVSLPSEMNSLLPNLQSLDIRGCRNICRWPEGGLPANLKELSVGECEELVRGLSWLGNLDNLTHLFIDGSNFESIIKSYPEVGWLPRLPSLTILQISWFPNLETLECSELLHLTSLQQLHISNCYMLENMAGEKLPPCQI

>arahy.Tifrunner.gnm2.ann1.S0ANF2.1

MKSIRNNKKAEVQKWREALTVAANLEGLDSHSYRDEIEFIQNIVKDVFQKLIDHYPPNDSKSLVGISENLEKVESLLSESVEVKKIGICGIGGIGKTTLARLIFEKYSYTFEGSCFLENGKFWQNNTKKSTFVEDRISKQRNFIVLDDVSSLEQLDYLVHKLQWCGAGSKIIITTRDKNVIVRTVETIYEMKILDSHESFKLFSLNAFNEDYPQIGYEELSWKAVGCCKGIPLALISLRSFLHLKSKTEWHSALQKLEKTPNPEIQNILRLSYDGLDEEAKQIFLDIACFFKRELVEYVVNLLDSYGLYAAIGMRSLLDRALIAISHNCVRMHDLIQELGWDIVCQQSSGNSENRSHLWDSNDIQDVLGNNKNLVNLEEVDLTDSQKLVKLPDFSKADNLKSVHLSGCRSLRHIHPSLLSLGKLELLDLLNCAKLERLETKMHSKSLKHLYIKSRTSLSYVSHFSSLQKLLLDGSPVETLPMSIKHLTELKTLSLKGCKMLQYLPELPSSIRHLTALNCIMLQTVTFSSNIPRLQEEKCINISFHNCMKLDVANCIYWYLKDIRKLAYVCESRRGGKGVVRRSDFLKICYLDYRVPEWFMHRTKGTSITFEVSSPSSYGFSSLLCVVLPKYSLDYELDIKCRCYLEDGINMHKYSFGILFLNHIPAEGCSDHVYMAYNYGGIFDVIKLDRLNNKIASSGKNPKLTFEFFVSSGDTGSKQDDNLLIKECGVYPLNDSNFRIE

>arahy.Tifrunner.gnm2.ann1.S37TL9.1

MESVLFQTGIVSASLQVILDRFTSFAHKELSLLFGFDNNDLNKLERTLFKVQSLVDNVRAINHSSSCSSHNKAQHLWLRDTQNALHEAEDFLDELALEISKLASADVNLDFTNKDQVRNLVFSKFKFSIPSQMVKMQNKLEELAREMDGSFVDELHKLGMPCSTVGNFQTSSLLDESVVIGRESDKREVIKLLLDEEMGGRSNLSVIAIVGMGGVGKTTLAQLVYNNVDVDSNFDLKIWVSVSVGYNIERVTRSIVECACREKVKLSDLEPIQMRLEEILNGKKFLIVLDGFWDEDEHNWDVLCLPLRVAARGSRVLVTTRSMLVSRIVATASPYQYHLKTLSGEDCWELLKQRAFSNMRHDTNKQLELRETGFKIAQKCDGLPLAARVLGSALRFRSDEMEWDAVLKSNTWDIPENRSQVIPALKLSYDLLDACLKRCFAYCSIFPASFEFKKDDLVQLWVSEGFVQPRGTRRAEDIGKQYFDILFQRSFFQSSHQDLQTQPVYKMHKLIHDLAQFVSDEVCLRMEDGKSLPWGDLRCVRHSSLVCKDIKVETLKAFLKCGRLRTFILLSQGVCQMDQIPYEFFLNLRYIRVLNLSFSNISELPNSIGTLKHLRHLDVSETHVEKLPESVTDLNGIEILKLNNCFKLLQLPKKMKNLTNLRHLELDIKRQISSMPKEIGKLTNLQTLSAFIVGKEKGQCIEELKDMRFLHGSICLVNLENVTNMREAVAAKLEQKPCLEGLELQWNEFKDGLEEQQILAGLQPHNRIKWLTITGYGGFMFPSWLGNPTFCKIERILLQNCRYCKVFPSLGQLPMLKHLYVEEMHDLTRVDDESQGPNSFPSLQSLTFHNLQNLQQWKDLKAGDMPNLTHLTVVDCPSLNFLPSLHYLTRLESLEISQCPKLQCLPAEGLPDSLECLIIIDCCVLKERCRVHEGEDWNKIQSIPKVEIDYEDIFW

>arahy.Tifrunner.gnm2.ann1.S6W881.1

LSDDKFVNWIRGKKLDQDPCGRLENILNVVEAVLNDAEKKQITDNAVKRWLKNLQDAVYDAVDLLDEICTKAATQKDPSNFLSHIFNSQDKDIVVTTIEEVIARLEDIAKYKDILGLEKIAAKNMSGRIPSTSLVKKSDMFVGRDRKKDTLVKLLLDDTKDGEISVIPIVGMGGIGKTTQAKLVYNDAEVKQKFHVKSMDTVQNHLKDKLEGKKFLVVVDDVWSNNREGWESFLTPFECGSDGGHTVVTSRLDSVASMVKTNHIQPFNLSLLHEEDCWLVFAKHAFFPTESRDRSALEITGRKIVEKYKRLPLTVQTLGSLLRTKDHEKSLLRTKDHEREWIDVLYNEIWEFSEDESSILPGLRISYYYLPSNLKRCFVYCSLFPKHYEFERDELVLLWMAEDLL

>arahy.Tifrunner.gnm2.ann1.S844Z0.1

MANPIPLPDFLQQRVSDFAFEKLKNVWKKHVRNRVDYALNCHKKVKKLGKAVEELKEDGKKVHDKAEEDEGLYGREVYHVKAWLRQVDEIISEYGKLKDEHSKHVVINLKKRYLCSKRAEEIKELVMELQKEKQDNISYWQQQQQQQQQWPSSGFMGVTFSDSDYMEFKSRRTTMKKIMASLEDSNSRLVGVYGPAGIGKTSLVIKAAKQAQEIDKLFDMVIMVNVTKRPDTKKIQGQIAEMLGMKLEDESEEARATSIQERLKNEKDNILIILDDLYAKLDLNALGIPLQTKEESSAIVVHNQNPELEDNERIGIPGAADKKMMKAKTSFIDGSSKLKEHNWGYKILLISELRQVLTEMDVKATSIFSINVLNVMEAEKLFKSMVGVGAKNSELETLATEIAKKCNGLPMSIVTTARALKNQLNPLVWKDTLVKLEKLAVENPNAAPEYSTKLSYEILENEELKLTFLLCARMDHDALVADLVRYCIGLGFLQGVYSVWEARDRVQMLLAKLKDSGLLSDSYSSHHFTMQNLVRNAALSISSTERHTFMMTEGKLNKWPGMDELESCNVISLKRCDFIGRFPESIKCPKLSIFHIENNDPSLKISDSFFQEMKELSVLILTGVNLSSLPSSIKCLTKLRMLCLEQCILGEKLEIIGELKNLRILSFSGSDVKSLPHELSHLSNLQIFDISNCYKLRVIPHDVMSNLTRLEELYMRNIPFQSEVDDGKQSENASLSVLGNLNQLTNLDLQIPNASSLPNNFFFDKLYSYKIIIGISISEAFIHCKKS

>arahy.Tifrunner.gnm2.ann1.S8SU5Z.1

MAGAKVLENGKSHEVVNRRMWSRAREVLLAVLDVIEEAEKKQARDPEVKLWFEGVKDICYELTDVSEEFELVQQAKKLRFVGLRLLQHLKGRRKMKSILREFEALREEVGKLKLGSSSELPVPVNLHAPITPSNISGKELILGREAVIQDVISKLTDRHVYHPCISLVGESGIGKTAIARHVYESVRGKFHFATWVTVSKQFNVKRIVKSMLKGSGHEPVAAEMELLEAEIQNLTVDARCLIVLDDVFHLDPNRWFHLLSVLGPCSSMFLITTQMERVAEVAESTIVFMRSLLDEDCWSIIKHHAFGDLKNESPVEWIFGQVGREIAKKCQGKPAVAKSVGNMLRGRSYEEWHQVLQIDSQWSDLRSSSMWNFSLMPPALRQCLLYCSIFPKNYSIQEDKLIKLWMAQGFIASDEDMMEIEGRKYVKQLRDCSAFQDGDGGLIMDEGMTDFIQDLAQNECRIMFLNDGTTAEEAGENRALTKAPHYRHCTLCLEDQTTFPDSIANAEKLHTLMVLSESSDIDSTNLASLLSRMKRIRALDLSSCTIQQLPLKAAELLHLRYLNLSFNHELKKLPSAISNLLNLQTLNLNGCNSLRKLPKSIRKLIKLRHLEILWTTSLSYLPKGIATLTLLRTLNRFFGSSGGASRSKACSLGDLENLNNIQEWITIDGLGGETEISEAKRAGLKNKKNLFGLELWFSIAGSEGNHQILLDNLKAPSQLQSLGIFHYGGSSFPNWIIELNGLKQLLLVNCSECNVLPPLGKLPCLESLEIKNMPKVEMVGSEFLGIGLNHDDAGNEGSSSAIAFPRLQKLHFITLGRWKGWTGINGNGGDEKVMPLLSSLSVVNCESLRSLPDYIKKKENLKPVIEGCSRLPRPRRSKNR

>arahy.Tifrunner.gnm2.ann1.SF2K3E.1

MAESFIFSIAESLTVKLASRAYEEASRVVGVYDDLQELKNSLSYVKAVLLDAEQKQEQSHELQEWLKQIKLIFYDAENVLDQVDCQTLRKQVIRDYGTPKDKVGRFFSSSNPLVFRYKLAHQIKEIKKRLDKVAADRDKFGLQVIDVDRRVVHKREMTYSHVVESDVIGRAHDKKKIIKLLMEPILDNNAGYKHISVIPIVGFGGLGKTTLAKLVFNDKRVTKSFPLKRWVSVSDDFNIKSLILKIINPLSDFASSTGQQNLRDLEIEQLQHRLRNVLEGQKFLLVLDDVWNEDRVKWVELQHLISVGAQGSKVIVTTRSQSIASMMGTVANPYHLKGLSPKDSLRLFVRWAFKEGEEGKYPDLIEIGREILKKCKGVPLAVRTLGSSLFSKHDIQEWESLRDKEIWNLPQKEDDILPALKLSYDEMPSHLRQCFALLSLYPKNHHFNSFGVASLWGAAGLLPLQSKDKTMVDVAHQYLRDFMARSFLHDVSDCGTFYFFGIHDLVHDLAVYVAKDVCQLVNSNTQDISENVLHLSFVENGLPYSSIKTSLQGVRSILFPVNNQGASEAFLDAWVLNCKYLRYLDLSDSTCETLPESIGKLKHLRYISLCNNKRIKRLPNSICQLQNLQVLDLEGCSNLETVPKKLRKLISLQRLEITTKQSILPESDIAKLNCLEVLCVQNCVNLESLFFETRLPKLRTLEVGGCASLKSLPLDTHHFPQLETLAISGVGNEDWLHRSEDTNAVLRLKTIVLSEMVTLPHSLQQYASTLQTLVIVGCYELEVLPEWLSNLSSLKFLFMAGCPKLKSLPSDIHRLTSLQVLRIINCTKLYGKYEPQVGECWPMISHIKHTDIRNCWSRKCCYS

>arahy.Tifrunner.gnm2.ann1.SGT4H9.1

MVDTVVSFVLDNLSRVLVSEVTLLSGVKDQIKPLSEELKFMNIFIKSSEGKHDDPVVREVVNQIRDVAYEAEDVIDTYVVNVNNQRSRNMLGKLLHSKDHVMTLHEVNDRITTIKRRIDDIYENKSKYGIQQGDFESHFRNKEFAEDSLLARRRDVEEEEVVGLVHDSDEVINHLGSRGDSSRKVVCILGMGGLGKTTLARKIYNSDKIKTMFQCRVWGFVSNDYRAEELLINLLKCLGLSVEECKDLNDQEKMKGEVRKRMSGKKYLLVLDDIWKTQVWDELQEAFPDDDKGSRILITTRVQDISHYTRATFTYKLPFLDETQSWELFCRKVFCKKNCPLELEPPGKEMASACKGLPLAIVVLAGMVAKKERSPREWHKIKNHVSWYLAQEEEYRIVTNILKLSYDDLPQTLKPCFLYLGVYPEDYEIHVRTLCQLWIAEGFIQKKEVGPSNSPEVEDIADMYLDKLVERSLVQVASRRTDGGVKTCRVHDLLRDLCISESSENKFMEVCTTLDANKCNPRRMSLQYRGNLRLTKDNQSSARSLLVFGERTIWKSKSEGWKQIENGFKLARVLHMNLVELHSQPSGLKSLIHLRYLKLIVGSLRIAGDVLTCICNLCNLEMLHLEFHRDSDHLALPSKIWKLESLRRIYFRQLYAKWDCTGVSMLSVENGETSMENLQTLGFIHLDSQLASVLNKGMFPNLTKLSLWQHLNKSNDEILEKILQCLNKLRTLKVHNISRLPLDPNVYPRSLSKITIRRHCEIDSRLIKTLGQLVNLQILNLELASMHYDVNCAAGDFPQLQVLRVKLYKRLPGRFMIR

>arahy.Tifrunner.gnm2.ann1.SH571D.1

MAESLLRMVIENLQAFVQDELATLWGVHSQIQELSGNLAVIHAVVQDAEEKQIRERAVKLWLQKLSDAAHVLDDILDECSIESNRLHSDQCLTRLDPVTIMFRRDIGKRMKEMVDRFRQIDEERRRFELRGRVPERQQEDEAWRQTCSGITEHNIYGREQDTENIVEFLSRSADSSNDLSVYPIVGMGGLGKTTLVRWVYNDKKVIEHFDLRIWVCVSTEFNTMRILESIVESTSGHNPNLSTLEALKNKVQEILLGKRYLLVLDDMWSTDKWEDLKSVLLCGGGTKGAAILVTTRVESVASVMGTCPAHHLSPLSEDDNWLLFKYHAFGSDKVERTELVAIGKEIVKKCGGSPLASKALGSLLRNKKEEIQWVNVLESKFWDILEDDAIIVRALKISYFHLKLSLRQCFAFCAIFPQDFGMEKEQLIHLWMANGLIKSKGKLEIEDVGNEAWEELCQRSFFQEVEIDELGRTTFKMHDLFHELAQSIMGEECRVYDESASLTNLSTRVHHVTCLKPERKVNMDPFKKAESLRSMINLLPFDDYHNLNGLPPFNSLRALRTNASQLSALKSLTHLRYLNLRRSGITTLPECVSRLQKLQILKLEDCLNLSCLPKHLTQLKDLRHLLIEECHSLVEMPPNIGELKCLRTLNLFIVDKKEGHGLSELRDLQLGGKLRIKGLENVLNEGDARDANLSAKKKLENLYLSWDSSDSRRGANAERILEALEPPSNLKSFGMNGYSGVELPSWMQNTSILSSLVTVILYDCKNCKHLPPLGKLPHLTVLYVSGMKDVKYIDEDSYDGVDEKAFKSLKYLTLSKLPNLEGMLRDERVEMLPLLSKLEVSCVPKIKLPLLPSLEYISIQGTWSDSDHSDSEGMASILEAIGQNMQHVKTLFISGFPKLKALPHELSSLSSLQKLEIYGGDELESFSENVMQGLCSLRSLKIGSCKKLKSLSEGVGHLTCLESLDIVNCPELVLPSSMNKLVSLQRVYIYSCGTMPEGLQHVPSLQSLDARNLHSIPEWLGDLTSLQKLDLYCKGLRSLPSSFRNLTNLRELSIYGCHKELQKRCTRVTGQDWQAIAHIPQFKLVPIHEETFSDKIRFKWRSWQLRRDRRRHHFAEADTFDYLVDTLFYWYYM

>arahy.Tifrunner.gnm2.ann1.SHX7PA.1

MAGALVGRAFFSGFINVVFDRLLTMDTVNRILGKKLGPGLVERLKISLHAAEAVLDDAEYKQLGDERVRDWLNSLRDAVYDADDFLDAVLTKAATKKEVHSVLLSFFLNRHRKMLDNMEGVVSRIEFLVRQKDILGLQKTTKDNNLSSSSSSSSWRETTCLMEGNIYGREDEQQALIKTINDKSESQLSVIPIVGMGGVGKTTLAKWAYSVAEGFDLKAWVCISETFDVAEITKKTIEEITKTTCSLGSLNLLQNELQKILSGKKFFIVLDDVWSDDADKWKQFITPFHCGANGSTILLTTRNQEVASVVQTCPSCTLNELSEESCWLLFAANACFPESNGNPTLEDVGRKIVKRCKGLPLAVETLGHFLRGKDDVKEWNVVLMNEIWELKNSKIIPALLISYFQLPAYLKRCFVYCSLFPKDHYFKKNELVLLWMAEDLLRLPKRGESLEEVGSQCFEELASRLFFKPAAEDFPEEYVMHDLLHDLAIFLAGDFYCTIQEFGEQEEKKVLIRHLSHLPYRSLDHPISKVLSIVKSESLRTSLYIDDLLSKKSRASKLKYLIVLSFCNLYALPGSIGKLIHLRYLNLSCTYVKTLPESLCNLYNLQTLILYKCLCLTMLPNGMHKLVNLRHLDLRGTSLKEMPRGISKLKHTPILDYFVVGKHKDNGIEELGGLSNLEGSFEIKKLENVADGRQARSARMLEKNHIDKLSLEWCSDDEMVSNTETQRYILDGLQPHTGLKELTILRYKGKRFPDWVGHCSYNNMTSVTLGSCNNCCVLPSLGQLPSLKSLGIYGFLELKRIGDEFYKNDSDHHSSPIAPFPSLEELVFNKMPCWEEWHVPHPEAFPRLRTLDIWNCGMLKGDILNGIFWIRDCCLREDDEGRSDEMVGGGDALSIRACPSSNARNINYCISGCPSIVSFPGNCLPKSLKISRCPKFEFLEKRQHRYDLVEIQIEDSCDSLTSLSLDVFPNLETLDIYECRNLESVSMSEAPHAALQGLTIKGCSKLVSFAGEGLAAPNLTYLQVSFCSKLEALPRDMKSLLPSLLQLKTVICWKNTARTSIN

>arahy.Tifrunner.gnm2.ann1.SK6LYR.1

MAATHKFSENYPHDLGQIQQSNAQLSELKRRKPVVLSNEVVGEDFERNVEKMWELVREEKVLMIGIHGMGGVGKTCLATHLETQIKRKGTFNHVIWVTVSREYTISKLQEDIAEAIGVKLGRDQRTRAAHLSSALSEKGKWVLILDDVWKFIDLQKVGIPRCGSKLILTSRLKHVLRQMDCPTLNIITMHPLSCSEGLELFLVRVGEDHKTHILKIAESIARECDGLPLAINVMARTMKDIDDIHQWRHALNKLRQKMEEVVFQVLKLSYDSLRHKSMQNSFLLCALYSEIHRDDKMIMMLVDSGAINGRRSLGEIFCEGHTIFNELEDHSLLLPHSKILRMKNSMRNMACHILNESQRCIVRYDQRLFGGIPHLLEWSADLDIVSLDTNRIEQIPAGISPNCPRLSTLILSNNHIISIPECFFTHMKSLAILDLSQNRSLTSLPDSLSDLSCLVSLLLHGCKALKKVPPLGRLQKLSRLVISGTSVEVVPGLEMLTNLTWLDLSYNKKLRLKAGSVLRDLTNLQCLNLFDSALLNVELEDVQGMISLEYFVAGFHDSKSYNNYVASIWNRGSTPKCYLLYLGDISDSEWILKAYDVIDPSDDNNQFLHLSDCVDLTHLLPKDITKLSIDFNTCWKSLCEALSNNASSSLMNIEVEGCTKMKSLFCLFGRCSFCSNLNNLQSLQLDDLESLTVICKEDVPVTNTITQPLKSNSIFSHLRRLNNSIFSHLRRLNVSYCHGLTILVTAALLPQLQNLQTLSAVRCYSLKEIFDERVSTISLPNLTTLELCHLTELEILCKGTIISTYLPKLEIDECPKFKNHHHFQVSSS

>arahy.Tifrunner.gnm2.ann1.SL8FFE.1

VPLIDSSMAEVVSSVASTLLANLATKSFQEIILACGLKDDIKKFESSLRTINAYLIDAENKQAKNHSIDDWLKQLREAFDDAGDILDEIEYEAKRNEVVKMYGSISTKVHRFFSYTSNSLAFRIKMAHKIKDMKQKMDEKIRQGRKLGIIEQHVNTPVLEHNLAWRETASSLPFRLCGRLEEKEEIMKSLMTQKSEANSIDVISIVGIGGLGKTTLVQMVYNDTQVKANFDTLMWVCVSDDFDVKKLIQKIIHAASKRENVVDANSSLEYMISLLNQNLHGKRFLLVLDDVWNENHNKWDELRNHLLEAGGGKGSIIIVTTRSQKVVEIVGSNLVMKLEGLPENECWRLFVKCAFQVEKEEEKYPRLKQIGEQIVKKCKRVPLAITTLGCLLRSKSHDENEWRKIRDSEVWNLNQEETDILPSLKLSYNHLPPQVKQCFSYCSCFPKDYDFHVIELIMFWMAHGLLQPTREEEDAEDIGELYIKKLVSTSLLQIDDGDDHFRLFDFQNLMTFKRLKMHDLVHDVAQLTMKESSKTRTIVQEGQQEASIEWTSNKFNYMRVLQLKKDMELSSFPNDCFVKMKKHLRYLYLENCPSLKKQTDSICKMQNLQSLYLDEFPKNMKNLIYLQYLFLMGIKITSMSSMNIGCFQQLKFLYLQCPKLVSVPSAVGRLTTLKKLGFLWCEELMNFEDEEEEGKQQMVVNNLNLQLFLIIGSKKLNALPKWLERATKLQYLSISITGIKLLPTRMPMTSLEELYVYHCKNLSSLPNMDQTHNLQYLVVYDCPALHARYNKETGPDWSKIAHIPYCKIDEDDDDDNDDDDDDDEEFMMMMKMMIE

>arahy.Tifrunner.gnm2.ann1.SMD16A.1

MGARGAILVAIAASIGNFIQGWDNATISGAIVYIKTELALQTSEEGLVVAMSLIGATLITTCSGAVSDWLGRRPLLIISSVFYFLSGLVMLWSPNVYVLCIARLLDGFGIGLAVTLAPIYISETAPPDIRGSLNTLPQFTGSAGMFLSYCMIFGMSLEASPSWRLMLGILFVPSLLYFLIAIFFLPESPRWLVSKGKMLQAKKVLQKLRGREDVSEEMALLVEGLGIGSETSIEEYLIGSAADQEPSEKDYKVLLYGSEGAGLSWVAKPVVASGHSSMKPASRLGSIIGNQSMHLMDPLVTLFDSIHEKIPETGSMRSVLFPHSGSMFSSTVENHHHHAKNEQFDEENIEREGDQDYYPSDAVDGVSDDDLRSPLISARGSSMFNHLRSGGDGEAGIGGGWQLAWKWSEKEGGLKRVYLHQEGSRRGSLVSVQGQGEGVHVAALVSQPALYSKQTIGPAMVHPSEVASKGSIWKDLTEPGVKHALIVGIGIQVLQQLAGINGVLYYTPQILQEAGVEILLKDIGISSKSTSFLLSAITTLLMLPCIALSMRLIDISGRRQLLLATIPVLIVSLVLLVVGNAIEFNSIVHALISTISVVVYFCCFKMAYGPIPNILCSEIFPTRVRGVCIAICALVFWIGNIIVTYTLPVMLSSIGLAGVFGIYAVGCFISWIFVFLKVPETKGMPLEVITEFFAVGARIIYIMTGALVGGAFLSGFINVVFDRLLTVDAVNLVLGKKLGSDLVERLKISLLAAEALVDDAEYKQLGDERVREWLNCLKDAVYIADDLLDDVLTKEATQKKVLSFLPSFLDRKRKMLMVNKMEEVVTRIEFLQKQKDLLGLEKSTKRNFLSWRIPSTSLVEGSIYGREKDQQEIIKILNETREHQLSVISIVGIGGVGKTTLAQWLYNNEDLMDRFQIKKWVCISEDFNIAKVTKNIIGQDGCNTDDFNCLQHELKKTLLGKKFFIVLDDVWSNDGDVWKKFITPFQYGANGSIILVTTRAKEVASLIQTCPPYILNELSEDGC

>arahy.Tifrunner.gnm2.ann1.SY9UAE.1

MAEALLEIVLTNLFPLVQSEFAAFFGIKEKAEELSKNLELIKAFLDDAEEKQWSNRPLKVWLQQLKDAMYEMDDILDQLPTESSQLGCLSSLNPKNVMHRRELGQRLNEIIGRLNGITQAGSNFGLRQVVRERQSEVVEWRQTSSTIAVPQVYGRDEDKARVVEFLLSPSRSFEFLSVYPIVGLGGLGKTTLVQLVYNDHQVGNNFDLKIWVCVSENFTIKSILRSILEAIKKDKSEVLDLEVMEEKVKELLQSKKYLLVLDDAWKRSQEMESGLTQDKWDKLRSVLSCGSKGSSILVSTRDNHVATIMGTCQAHHLDRLSNDDCWSLFKLRAFGADKEERAELVAIGKEIVKKCGGSPLAALALGGVMQSRSTEKEWLEVQNSKLWSLPDENDIMAVLRLSYSCLTPTQKQCFSFCAIFPKDAEIMKQELIYLWMANGFISSRPDLEVEEVGNMVWKELYQKSLFQDVRSDDFSGEIYFKMHDLVHDLAESISGQECICLEKQDLNDSSINPRHVVFHGIDKEQFKKRAFEKAESLRTLYQLNSDEFPFTSRLIPTNNSLRVLCIYSRKIPSFGSLSCLRYLELRDLDIKSLPASICNLHRLEILKLKKLSSLRRLPKHLTRMQNLRHLVIDECSLLSRMFPDAHKLRDLRTLSVYVVKSEEGHSLAELRGLNLGGKLSIEGLGNVGSISEDENANLTGKQDLRELILSWSNSGKRKSVVGAEEVLEALQPHSTLKLLTIEYYEGLQWPTWMQNNSATHNLVSLRLVKCGKCGHLPPVGKLPFLKKLVVKCMDEVQYIEEDESYDGVEAMPFPSLEKLYLSYLPNVERLMKRETTHMFPSLSILFIEDCPKLQLPCLPSVKDLTVWYCSNEQLKSISNLNALNQLHLCDSDQVSCFPEGMMDNMTSLATLEIYSFRELKELPSDITKLTALSDLTINDCGKLECLPEQGLEGLSSLRKLFIHSCKSLGSLPDGVRHLTSLQSLTIGGCPMLKERCKQGTGEDWHKIAHVPHLILDASYIVQFLL

>arahy.Tifrunner.gnm2.ann1.SYAW3D.1

MVDWTGSLVSAVLQVLFDRIARRELIDFFRVNHLNQSLLEKLKMLLLSVTAVLDDAEEKQLTDQLVKEWVDRLKNAVFDADDLLDEIATRALQDMMEPGPRTTLDQVRDYASSLNPFAERVKSKVERIVERLKSIIEHKDLLGLKEGAAGVNKNKPLSLPLPTTSLVDEQRVYGRNDDKEKIIDSLLSGELLHGGIEGVPVVAIVGMAGVGKTTLAQILYNDIRVRNHFHLRSWASISEASGVYEITKKIFESCAPKYSNIIDLNVLQVKLQDILARHRFLLVLDGFSSVNSLEWDMLRRPFQSGKSWES

>arahy.Tifrunner.gnm2.ann1.T13XGJ.1

SSSTENTNRDYSGLHPGAADNLELYMFCAPLKIYRGEDTRHGFTGHLYDALCRNGINTFIDDENLRTGETIRPQLLQSIEASKISIIVFSTNYAASTWCLDELVKILQCHRERNQLVFPVFYKVEPSDVRHQRNTYKEAMDAHEIRFGCHTQKVKEWKEALAETSNMKGFHLKQGYEFKFIQEIVSKALTHIPPRQLLIEDRMIGLQTRVVEVESHLYSSYSTSKYKILSNIKSPKPKLYNNNTMLGIVGIGGSGKTTLAKALYNSICGRFECACFLFNVRKISDQEEGLVRLQQTLLSKLLGEGEIKVRSVEEGISMIKEKLSKKRALIVLDDVDKIEQLKAFAGECDWFSDETRIVMTTRDKYLLTAHKVEKIYKMKLLSDPESLELFCWNAFKMTRPEANYEGLSNQAIHYAQGLPLALKVIGSNLINKNLKEWKSALDKYEKNPPKDIQSVLRVSYDSLEGNEKDIFLDIACFFNGKKCEYVKNVLDGCGMFTEDGIRVLVDKSLLTINDGYLRMHDLIQNMGREIVKQEAPKDVSKRSRLWFHEDVLKLLTEDKENKKIEGIKLVQCEEDDWTDTAFVKMKQLRILILRNTNLSCGTIHLPKQLRLLDWRGYPSNSIPSDLKEIVAFSLRHSPLTLEKPFQNFGHLTYMNFSHCESITHFPNVSEAQCLRKLILNGCINLVRFDESVGFLPNLTYLRASKCIKLTKFLSRICLPSLEHLSFNWCRRLGLFPDIVGKMDKPLKICLKATAIQELPDSFVDLVGLHYLDLTSCEKLGYLPSCLFMLPNFVTLKVGGCPQLGGSFARFRESLSTNAECRPSLETLHFSHASLCDEDLHVIMLSFPNLEVLNVSLNNFVSIPACVQELSYLTSLDLSYCLNLQEIPKLPSSVRKVDLRHCDSLSASTTSMLWSQVREEIHKLQVVMPTSNTKIPKWWDDRTSWKHDPQDLNFWARGKFPVVALAFVFGEMNYQSVGLDLSIDSGDVNSTYQPSHNFRVAENHVLLCDLRLWFSDEEWKRLDAHVEHGNKWKTVKVRCVPDIIPVHWGVYVYKEETSMKDVQFQEWEAVNLSWTEPSVKKRLYSAEELYIASFSESLKTVVKNLKRLMAPREEQSFCLMQHDRDKDKDEAEDEDEDEEGESDMEA

>arahy.Tifrunner.gnm2.ann1.T2FAFC.1

MFSSASENNKKAEVQKWREALTAATNLEGLDSRSYRDEIEFIQNIVKDVLQKLINHYPPNDSKSLIGISKNLEKKIRWCGAGSKIIITARDKNVLVPTVETIYEMKILDSHESFKLFSLNAFNEDYPQIGYEELSWKAVSCCKGIPLALIALGSFLHSKSKTEWHSALQKLEKTPDPEIQNILRLSYDRLDDEAKQIFLDIACYFKGELVEYVVNLLDSCGLYAAIGMRSLLDRALIAISHNCVRMHDLIQELGWDIVCQQSSRNPENHSHLWDSNDIQDVLGNNKGTDSIESIVFDMSQIADLQLNADTFKKMPKLIFLKLYIPSKSDGKLNKLQLPVGLKLFPSKLRYLEWDAYPLPSLPLNFCPEKLVTLRFRNIKLKRLWDGVQ

>arahy.Tifrunner.gnm2.ann1.T30XJM.1

MAGALVGGAFPSGFINVVFDRFLTMDAVNLVLGKKLGPDLVQRLKTALLGAEALVADAEMKQFGNPSVRKWLDHLRDAVYCAEDLLDTVLTKAATRKEESFSWSLSFYINRDRDDMVDKMEGVVRRIEDLGRQKDFLGLEKIPTGSSSWRTPSSSLVKGNVYGREDDQKALVQMLNDNNEYHLSVIAIVGIGGVGKTTLAQWLYNNAEFMEGFDLKAWVCVSEKFEVVETTRNVIKQIHGGTCSLDDFSSLQNALKEELSNKKFFIVLDDVWSNDGDKWSNFMNPFQQNGKKGSIIFLTTREENVASTVQNCQPYFLRKLSEDYCWSVFAENASFPQSNGRAAIEEIGRKIVKKCDGLPLAAETLGRLLRTKHDVEEWNKILMSDIWEFSVEKSKIIPALLISYFHLSPYLKRCFVYCALFPKDFEFVKDVLIFLWMTEDLLPPPKRGESLEEVGCECFDELTSRLLFTKSEGFDDYFVMHDLLHDLAIFLAGDFYCNSEELDLSHCSSKLYNSISKVESLRTLLLFGDFWSPNCNIEAATCEILSKCKYLRVLSLRKLDEVPNLIGELIHLRYLNLSWTDDIKTLPESLCNLCNLQILKLYQCSELTTLPSGLHNLVNLRHLDIRGTSLEEMPRKMSKLNQLHVLSSFVVGKHKDNGIQELGGLVHLHGSVEIKKLENIVDVNKAKRAKIMDKKHIDELCLKWSSGDDLVSSTQKERDILDKLQPQNGLKELKIKGYKGTIFPDWLGNYSYENMTRVSLKSCKNCCMLPSLGQLPSLKSLRIEGLDQLRSIGEEFYKNEGAHHSSHIAAFPSLETLLFDNNITWHVGRCGTYLSRELFLNLGSFK

>arahy.Tifrunner.gnm2.ann1.T9QY5T.1

MKNLVGIDSRVEGVINLIGLGLNDVRYMVIWGMGGIGKTTIARAVFETIRSRFEVSCFLADVREHCEKKDTVHIQKQLLDQMNISSYAVYNKYDGRRIIQNSLCLKKVLLVLDDVNHEKQLEDLAGEKDWFGPGSRIIITTRDVEVLKGPEVHEIYKVEGLVESEALNLFCLKAFKQQEPTEGFLDLSKEVVKYSGGLPLALKVLGSYLNGRPTIAVWYSAIERIKKSSHSEIIDVLKISYDGLDLMEKNIFLDIACFFKGQRKDDVTKILKGCGHDAEIGLDILINRSLVTIEYDSLGMHDLLEEMGKQIVIQESPNDASKRSRLWCYEDVDFVLTQKKVLEKLEHLDLSWCKQLKQTPDLSGAPNLKKLNLQGCKKLDYIHPSLTHHKRLVELDLGYCERLETLGDKLEISSLEKLDLYRCSSLRRLPEFGECMKQLSILDLEKTGIEELPPTLGKLAGVSELDLTGCYKLTSLPFPLGCFVGLKKLKLNRFVELSCVPYTTHGLESLEAWDFSDSPILVGLLCSLSRLTSLSSLKLHGEFSRSREVSTLYYDLGHLTSLADLDLGYSDFLRVPICIHALPRLTRLDLCYCYNLEVLPELPSSLRELQAKCFDSLVASNVNAAISKACCGFAESASQDREDLLQMWISRKKMPAWFKDQEKDNGISVSFPHNCPSTETIALALCFLLQGVMDLPEQPSVICNGKEFINKSVFHGDLRLKSKYLCIVCVNGYYFSNMLLPRQSLPTAISQ

>arahy.Tifrunner.gnm2.ann1.TFI3EY.1

MGRALLSSLLHDLMDNLLYLTSQTKHFMMKPHKYQRKLMDKLLILDALVDDAEQKQFTEGSNAQAVKGWLNELIHASYQLEELLMQIMATIKSPHSQGQQFFKVITTTKKPDKYIMARISESLGNLESLVKKKDVLGLTEHATWRESLQGLPSSSDVLDNYFYGNYPKDYFYGREEEEKSILENLLSASESDEHIKVINIVGNSGAGKTALADVVYFNHKVRESFELRAWITVPYKATATFIAKKILQAVTEEFVAGEDFDVLWKRLQESLDGKKFLLVLDDIRIEDELHKWSKLIASLESSAAKGSSIILTSSPHDDPKLLMLPANHDVHVSLLSIHNCWSIFLVHAFGQIDLHQHPELAAVGKEIVNKLGNLPLAAKMIGSLLQDKLHINQWVQILRCELLDAGDVDLPIPSFLVLCYLDLPAQLKRCFAYLSLFPKGYEFKQKEVILLWMAQGFLNDSDSKSNGKSMEDIGDEYFGYLIMRSFLQPFGSGVSFIMHNLVHDLASYAFGESYKHHLSYLRSTEDFPELSFYKHGELSRTILPIYLPLEGAPRWFDSSLLEQIIMKVNPHVFRVLSLSHYDITHLPASIGRLSCLSYLDLSHTALQTLPDSICDLLSLQTLRLTNCTSLTSLPKRVCNLVNLRYLDVRDSGLQEMPLEMHKLTSLRALTDFIVSKYVPRFGDLAGMYNLKTLTISKLENVVYAKNASDAKLKEKKTLDELMLQWSNGKHSNGNEMEVLESLEPHKDLKRLTVEYYNGASFPNWLGDKSYWDLQLVDLRHCENCNSLPTLGMLPFLKDLFIEGFTQVSSIGAEFYGVATASQKPFQSLERLQFQDMLEWKEWSILEGIEFPCLVELYIIRCPKLVRDLPKQVLSLEKLEIFGCYALEAPLPKVSDTCQVFVHDSNDIVMRNIAKTHSQPSLRRVKISEEVHEITVQSFSGFPSTKYTIGSSLGEIEEIAHVERMESESTRSRWPLDSSSSDTLRIPKLKETSSRIISHQVEANIPNIKQQKDPQSSDTKFNTPITTQAATKVETSSSKEDLDDQRSSFEMLKISTVSQLKSLPTKLHSLKIEGCESLESLPNDLLEGIITLEDLYLISCSSLRSLPSLGSVTTLYIRNCRRLENLPSLESRKQLACLHHLFIGSSCDSLTSLTLDLFPKLKILCIWDCPNLQSFNVTKEYKGDLPSLESLEIRDCPGLMSFPEGGIHAPNLESIFLSNCKNLNNLPDAMNSLTSLKTLFLHKCPEIESFPYGGLPSSLILLSVAYCDKLTPQKNWRLDTLESLNRLELEGGCMGMDSFPEDMILPPNINSLCISTLKSLKKLDYSGFQHLNALQTLEIHCCGMLCSLPDQGLPSSLNQLCVQECPLLTPRLKPNSGKEWHKVARIPHIQIDHQILYLGNQCQS

>arahy.Tifrunner.gnm2.ann1.THV0ST.1

MAANDVFLSFRGGTRYRFSDHLYGALRQNRIETFRDNENLRVGDELEPILMKEIENCEMAVVVLCENYASSTWCLRELVKIIDCHEKQGKQVLPIIYRVNPSDVWDQKGCYETAMAKHENREDPQKVKAWRLALSKVEKLGWVHCKEDMSEPEFIKVIVRDTAGRLPLPEPTDHVVGLDTRCEEVKSRLDIESYENVCMLGIYGPGGIGKTTLAKCLFDKIKRQFEASCFLGNVREKSESRESLESLQKTLLDDMGEETITEFGSEFKGGSEIKRRLRHRRVLLVLDDVNSITQLESLAGGHDWFGSGSRIIITTRDTDMVDKHVMGDVVTKKYKMEELNDDDSLELFCWHAFNSKEPAENFENVSRNAISYAKGFPLALEVIGSHLGGFESVDSWEEELDQYRIDPSIQGVLKRSYDSLYELDQKTFLDIACFFKGEKWEYVKRVLKACDFYPVVRVFISKCLITVNQNGCLEMHDLVQDMGKEIVTSDSPTNPGERSRLWSHKDILQVLKDDTGSSSIEGIMLHPATHEEVDHWITTAFNKMENLRILIVRNTIFSTAPSCLPKSLRLLEWKGYPSESSPLDFHPNRIVDLKLPHSALKLEKSFQIFEDLTFINLSQCQSITQIPNVSGAKSLRVFILDRCHKLVKFDESVGFLPNLVYLSASECRVLRSFVPRMYLPSLEVLSFYFCKKLQNFPDVMQKMDKPLKIYLANTAIKEFPDSIGNLIGLEHIDISICRGLKDLPSSFFKLPKLFTLTMEGCYHLRRSFKRFKESHSTLDDCPNIKRLNFTGANLSDEDLHPTIQVLQKLEDVNVSHNDFVSLPNCIERSVHIKCLDVSYCKNLKTIPELPSSIQKVDARYCLSLTSESSTELWSKVLLETERIQIVMPKKEIPNLFDCDSSEGIPLFWARRKFPVVALAFMFGRSTVSNSVAETRTDILGFFPQIGSSLSFIVRLHLFIGGKQIFPKDSKYFSVGEDHVLLFDLRALFSDEEWHDLDAYLGVGDDGWKAIQVQCESPLTLSHWGVYVYKQDTNSADIQFKNPNPKSPLSDLVPKRSPHEPQESMMRQVAENLNPRELLGDYLPLVELTEVPSFTSALLRSLRTGKAEATRPESSAYGASLKQEHEESNWNVSRVMDMIKDDVPTHIADAYSNEIREGRRFVEELMRARMELLKEKGQERMDIDMAIVLEQPRSGRPPSRRYWGRLHIKYEEITAKAIVRKTTQLAWRDWNPGRTTTKEKATAVLLKCTGQGESSEEENNDPVLAELLSQIEEDAMRFNTSYGKMKACIILTDDAYALISEEYVPEFMLIRGKENAEIKGMSQWGALELLIYGFMRASGENTFGSVESEPRFEKTPYGKIRVEN

>arahy.Tifrunner.gnm2.ann1.TKZD77.1

MADKLYGGAYLSPFVNTVLDNMSSILEDDSVLYGNDSALELLGRLQNCLYDVGPVLDDAELKQFTDKKVKKWLVDLHDALYMADDLLDELSTKAAISANQRESGNSSSWSRLVDSYIEDSGDLEKIVRRLESVVARKKYLRLKESAKVDMSWRIPSTCLVEPSEICGRKEDKEAILKLLLDDDDAADGDLSVIPIVGMGGIGKTTLAQLLYHDDKVKENFKFQAWVCVSEEFDIVKVTKTIIEAITSSSCNLADLNLLQLHLKEILLRKKFLIVLDDVWNENYEDWNKLLKPFQRRVKGSKILITTRNKNVASVVQTVSPHELRSLSDEDCWLVFAKHARLSTVSIENPTLEKIGRDIVKKCDGLPLAAQALGGLLRENLDIRSWNHLLKSEIWELSDDKINVIPALRVSYYFLPSYLKQCFIYCSLYPKDYEFSKDELILLWMAENFLQPVGKKTVEEVGDEYFDELIARSFLQPHSTKENKFVMHDLVHDLAMTCAGEFYFRAEELRNAVEVDIKTRHLSHNAKSNYPMSKLLGICDRLKHTRTFLEINLDSLIPFNMENAPCIFLSQLKYLRALSFNSFPLESVPDSIESLGNLYNLQTLKLSSCSNLKMLPVSMKDLVNLRHLDIGGIGLHEMPKGMSKLKSLQFLSGYVVGKHEENKIKELGALANLHDSIWIDKLEDVVDSSEALEARMFDKNSINSLSLWWSVNKDENTVDSQMESDILDKLRPHTNLKELQIRGYRSTTFPDWLGHSLYHNIITMKLGDCRNCCKLPSLGQLPSLKHLSISDFGSVEIVGAEFYFYHYDESCLETPFPNLETLSFVSMRCWKEWRSLEYNAFPRLRELTISSCPMLRGDLPSQLPSLQSLQIKCCKQLSSCLPRAPALTSLSIEDGNKARIEELPPLLRELSIAGKHEVEWVVEAIMHMQLTCLTSLWIENCSSHISFPVSCIPASLQELTIQHCRKLEFQMDGQHYSLKELSIHSSCDSVTSFSLLDSFPNLVRVDIRNCKNMECIVVSRSLSCLRSLIIRYCRSLKSVSTLWMAASQLEHLTVLECPEIELCPTGDGDPHRSLRSLSISYCEKLGNSEAFMNSQFHGLTHLNIEGGSGESVKCFPKEGWLPASLESLSLERIQSVETLQCKGLAHLTSLQKLSIEYCPKLENIEGEKLPASLIRLFINRSRLLGKRLETKDPQVWPKISHIPGIQVNYTWIW

>arahy.Tifrunner.gnm2.ann1.TNU8Q0.2
[truncated: 4,000,726 more chars]
