# Supplementary figures and images for: Evolution of NLR genes in genus Arachis reveals asymmetric expansion of NLRome in wild and domesticated tetraploid species
Source: Sci Rep. 2023 Jun 8;13:9305. doi: 10.1038/s41598-023-36302-1 (PMC10250334; doi:10.1038/s41598-023-36302-1)

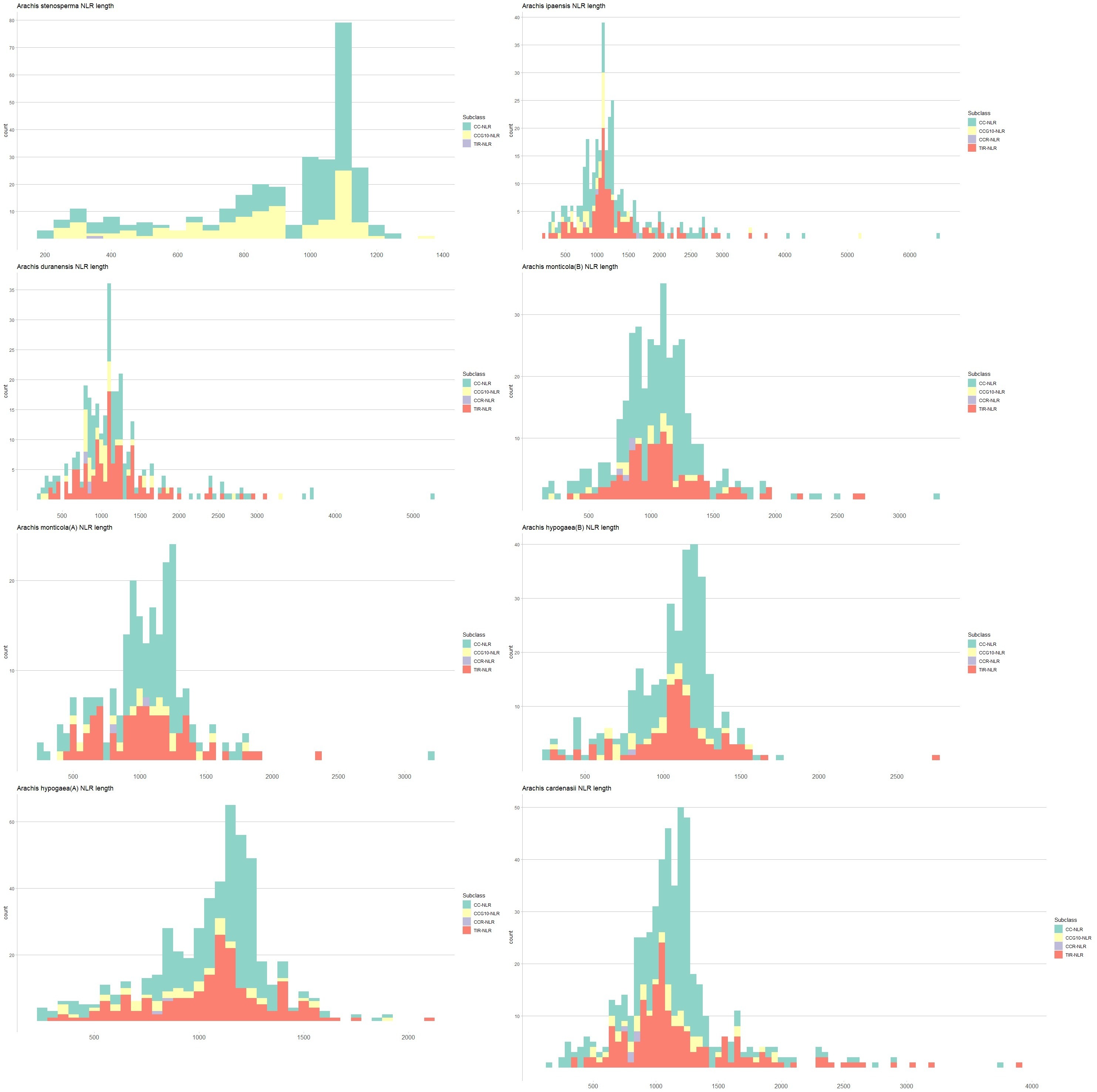

Supplement: Supplementary file 2 — Supplementary Information 2. [file 41598_2023_36302_MOESM2_ESM.jpg]

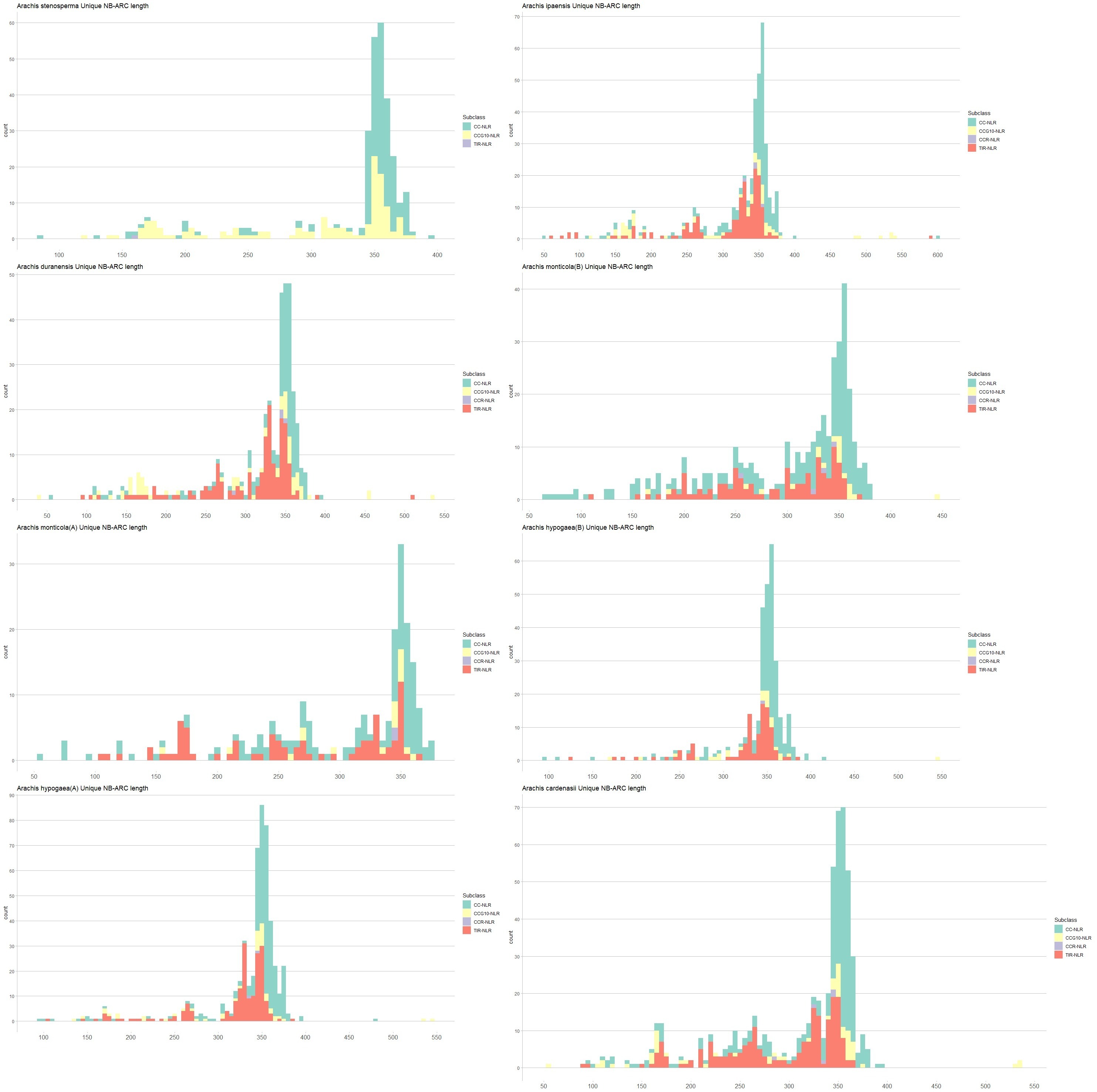

Supplement: Supplementary file 3 — Supplementary Information 3. [file 41598_2023_36302_MOESM3_ESM.jpg]

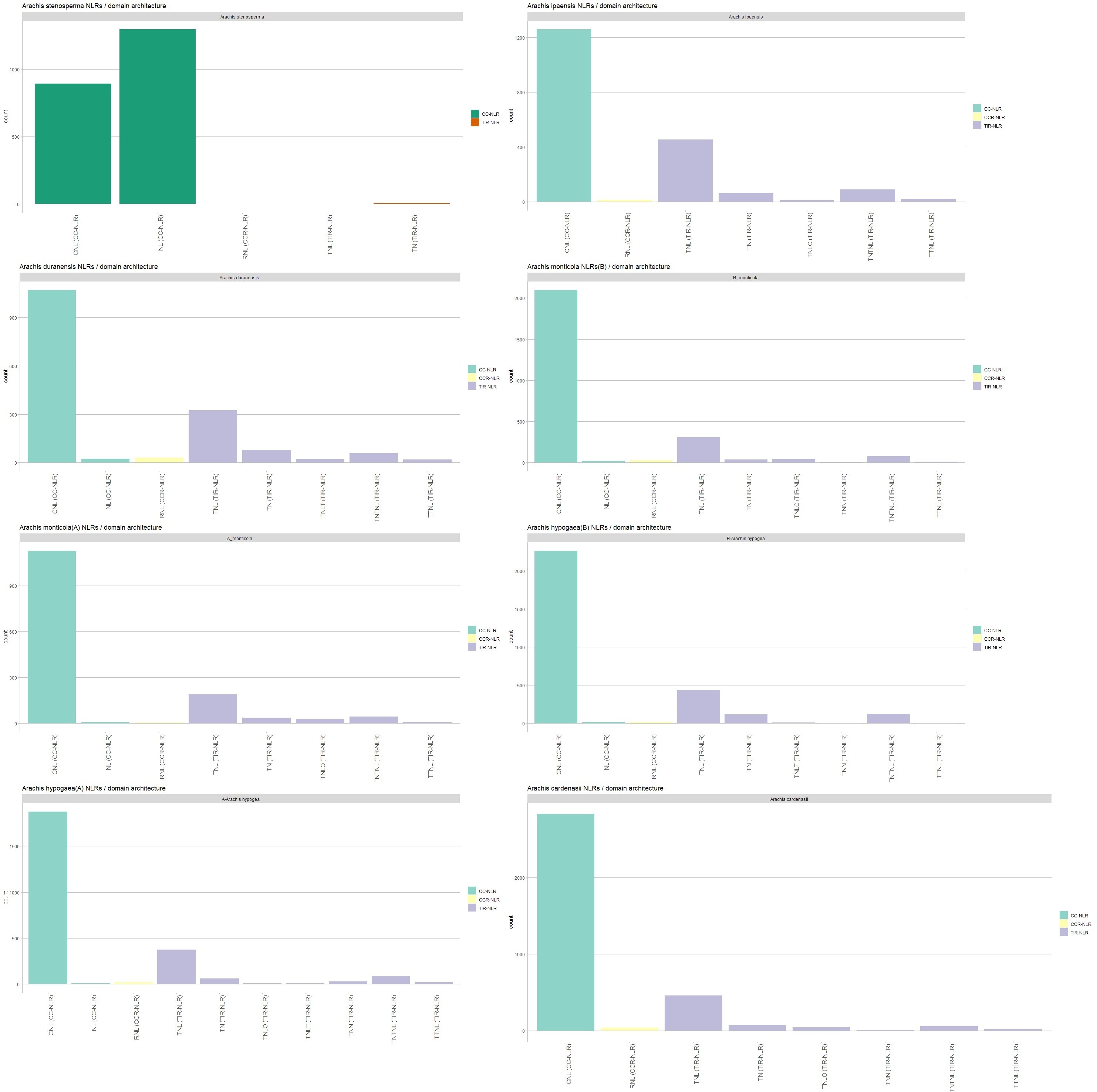

Supplement: Supplementary file 4 — Supplementary Information 4. [file 41598_2023_36302_MOESM4_ESM.jpg]

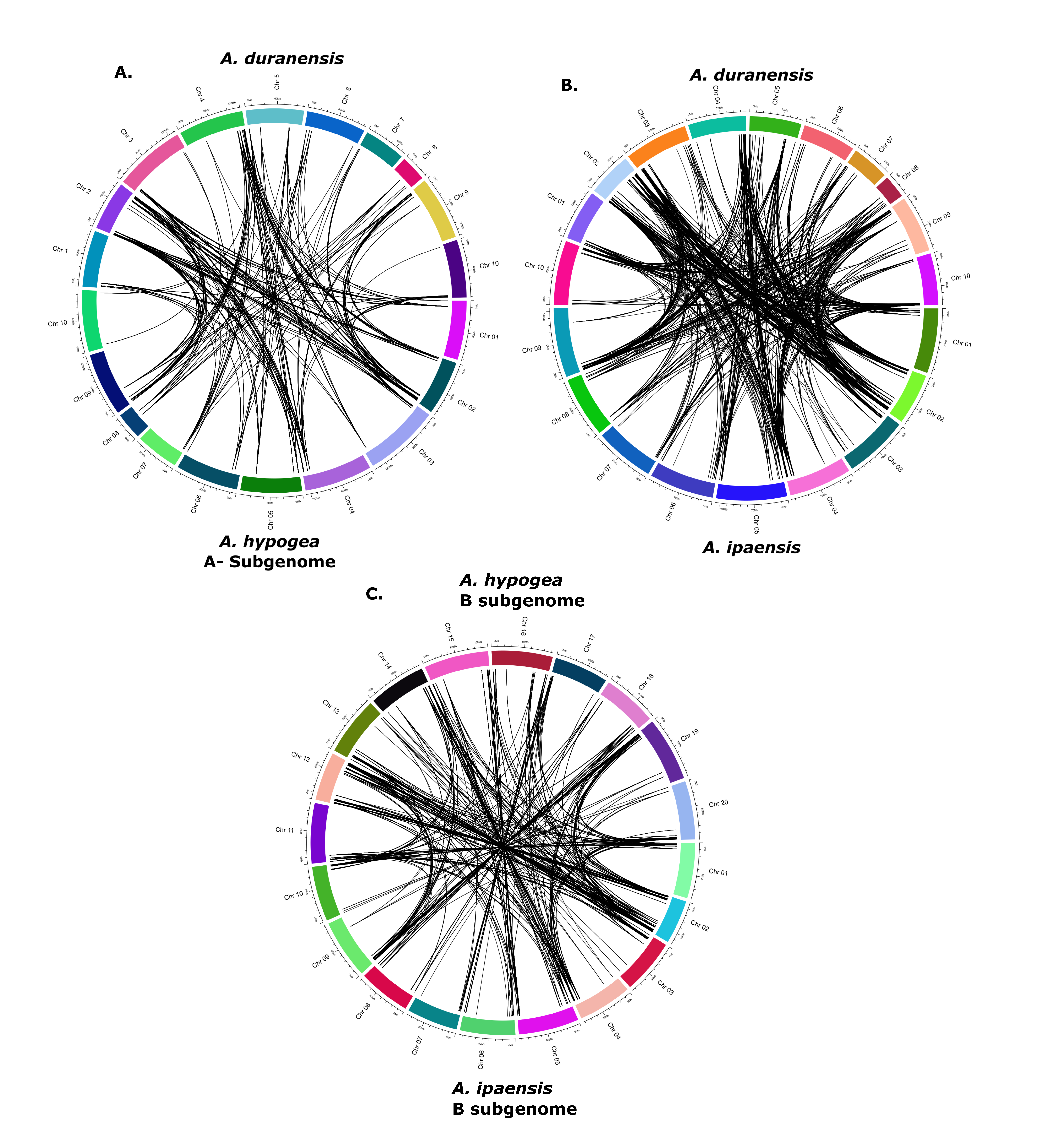

Supplement: Supplementary file 5 — Supplementary Information 5. [file 41598_2023_36302_MOESM5_ESM.jpeg]

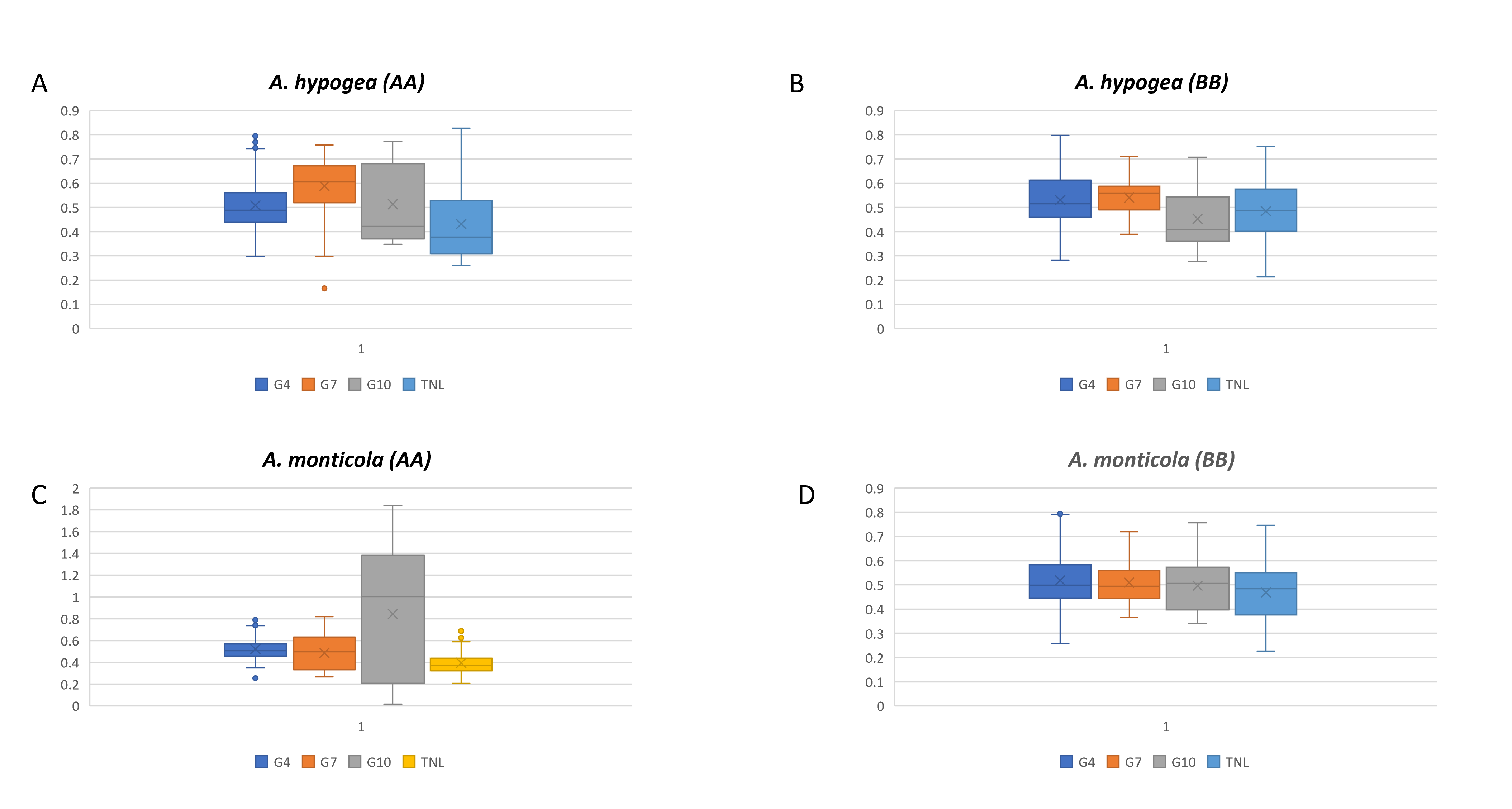

Supplement: Supplementary file 6 — Supplementary Information 6. [file 41598_2023_36302_MOESM6_ESM.jpeg]

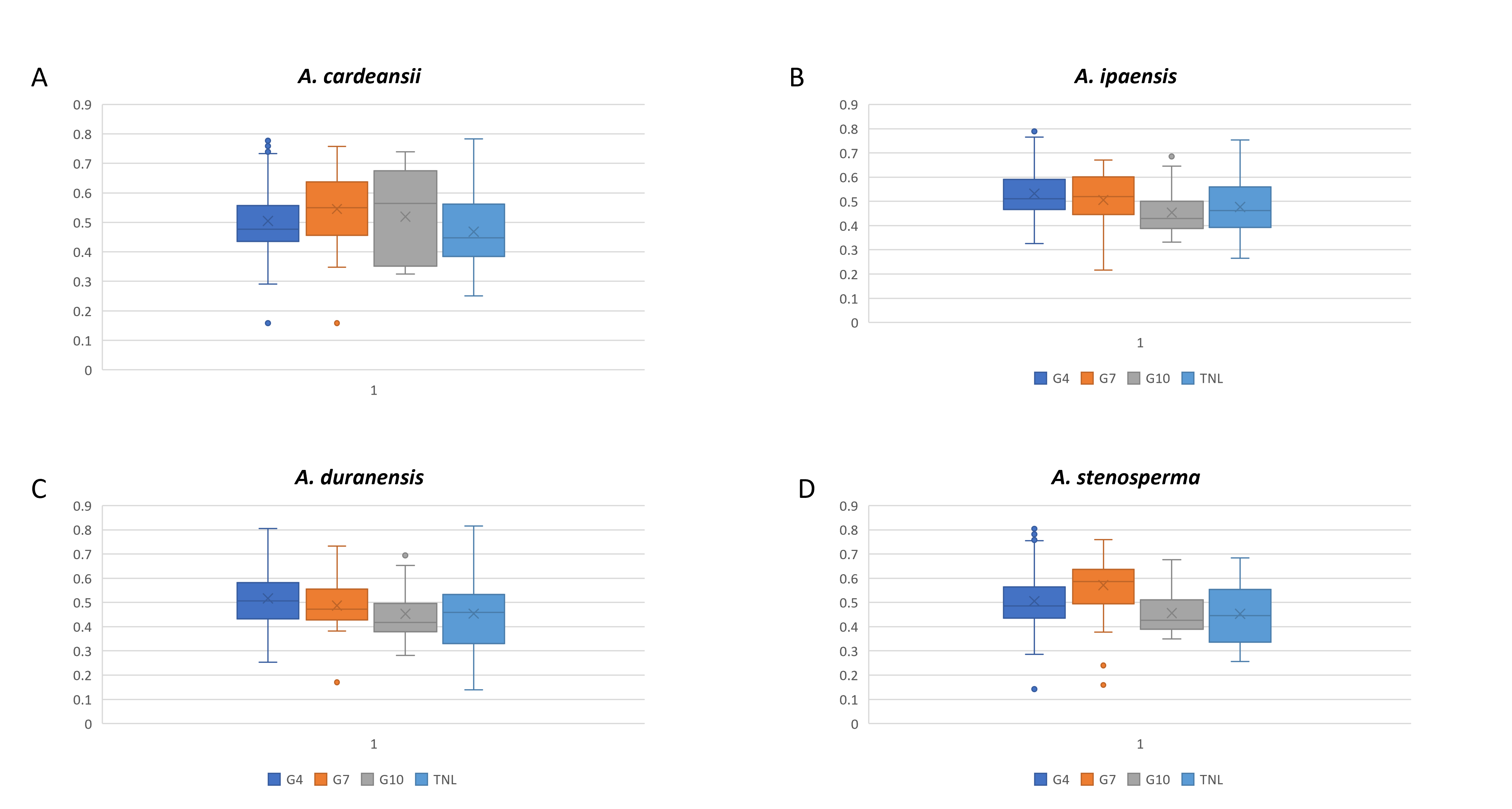

Supplement: Supplementary file 7 — Supplementary Information 7. [file 41598_2023_36302_MOESM7_ESM.jpeg]

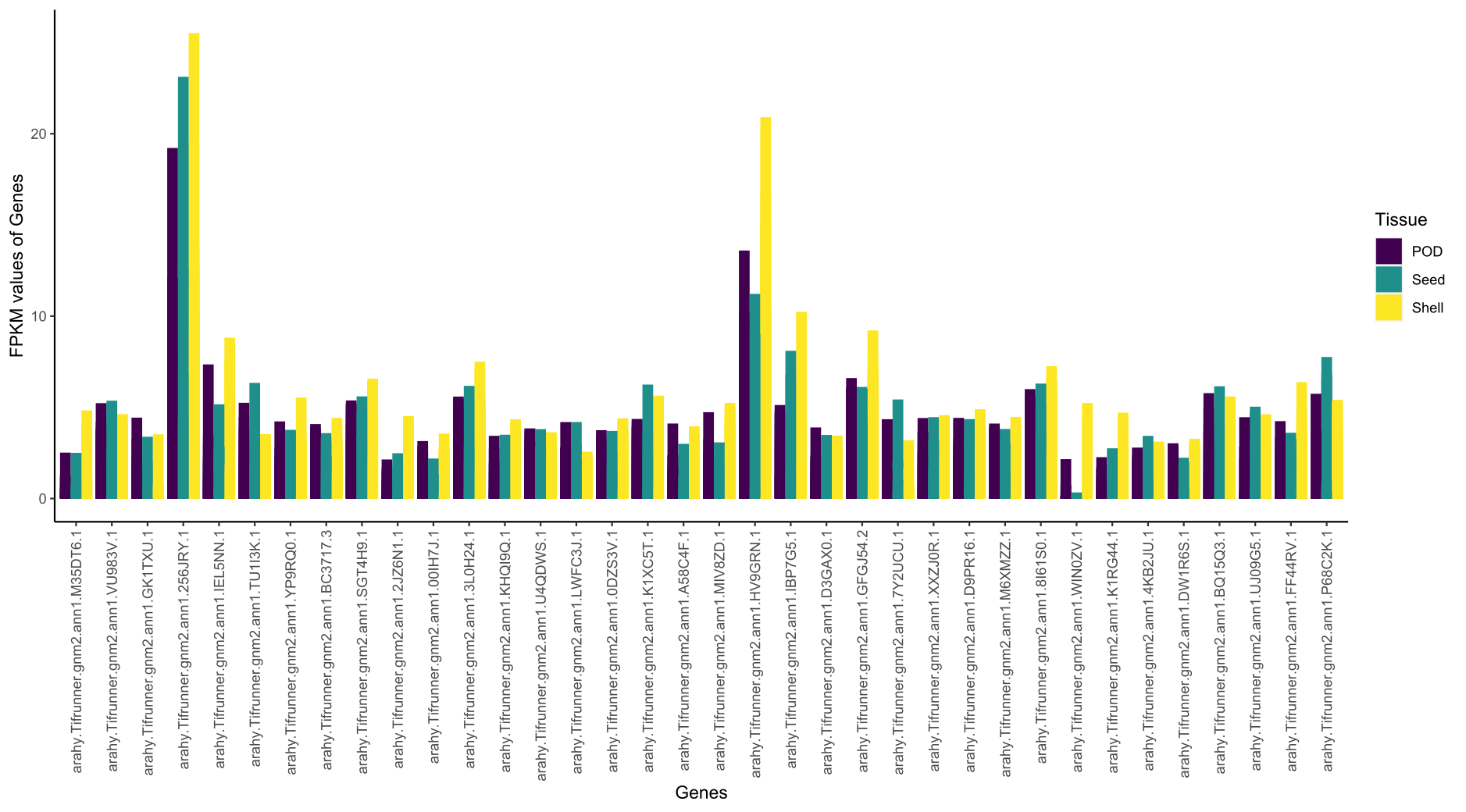

Supplement: Supplementary file 8 — Supplementary Information 8. [file 41598_2023_36302_MOESM8_ESM.jpeg]

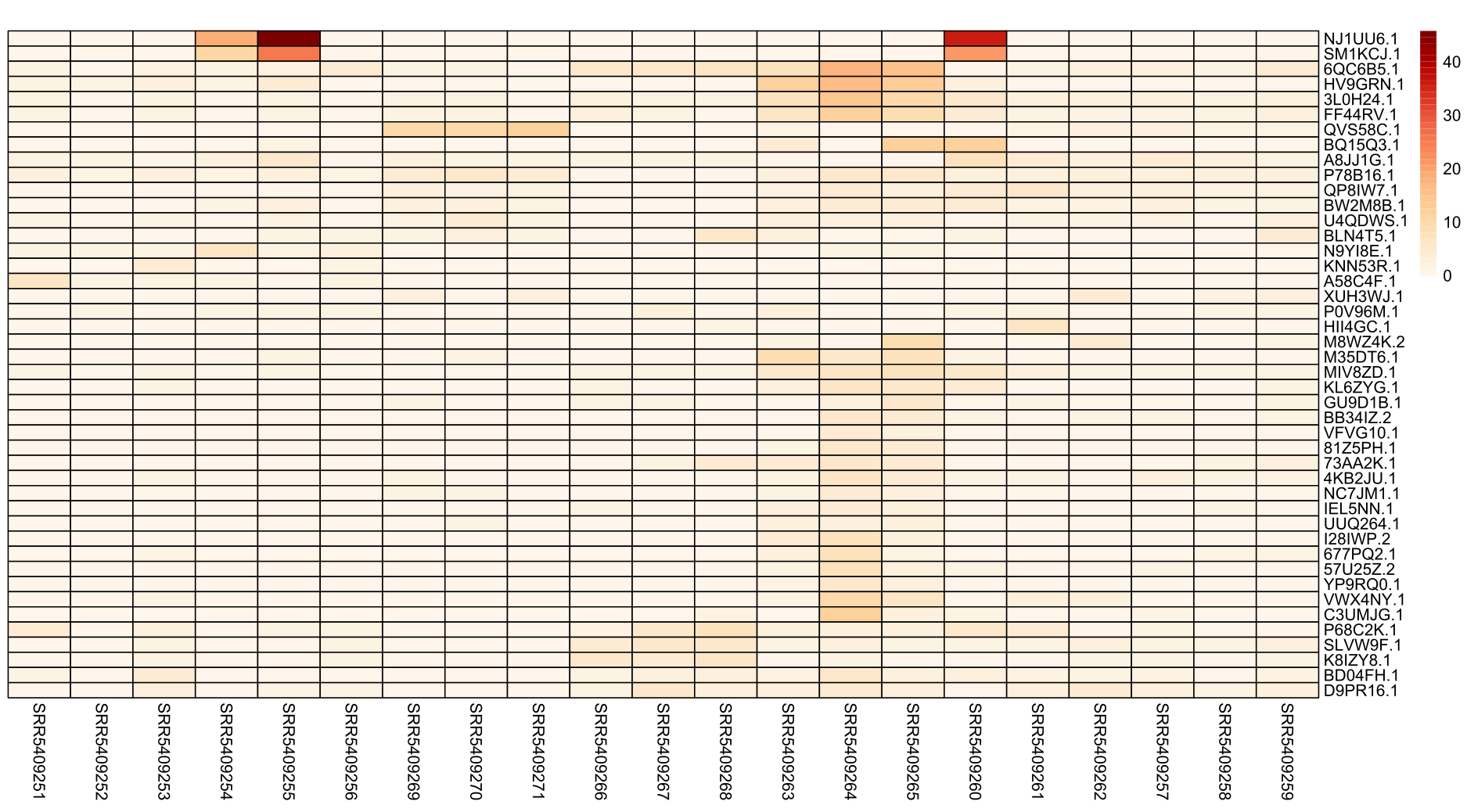

Supplement: Supplementary file 9 — Supplementary Information 9. [file 41598_2023_36302_MOESM9_ESM.png]

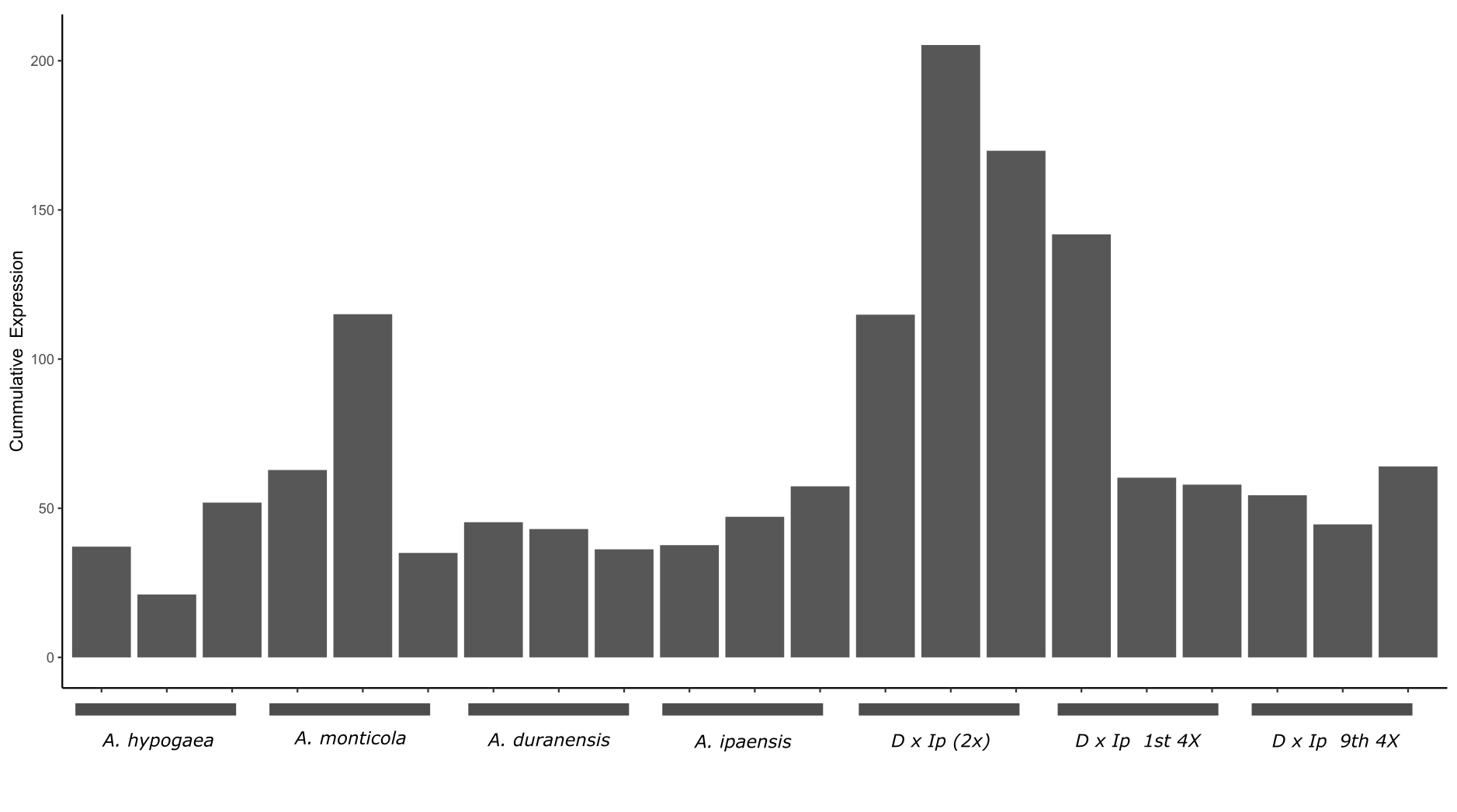

Supplement: Supplementary file 10 — Supplementary Information 10. [file 41598_2023_36302_MOESM10_ESM.jpeg]

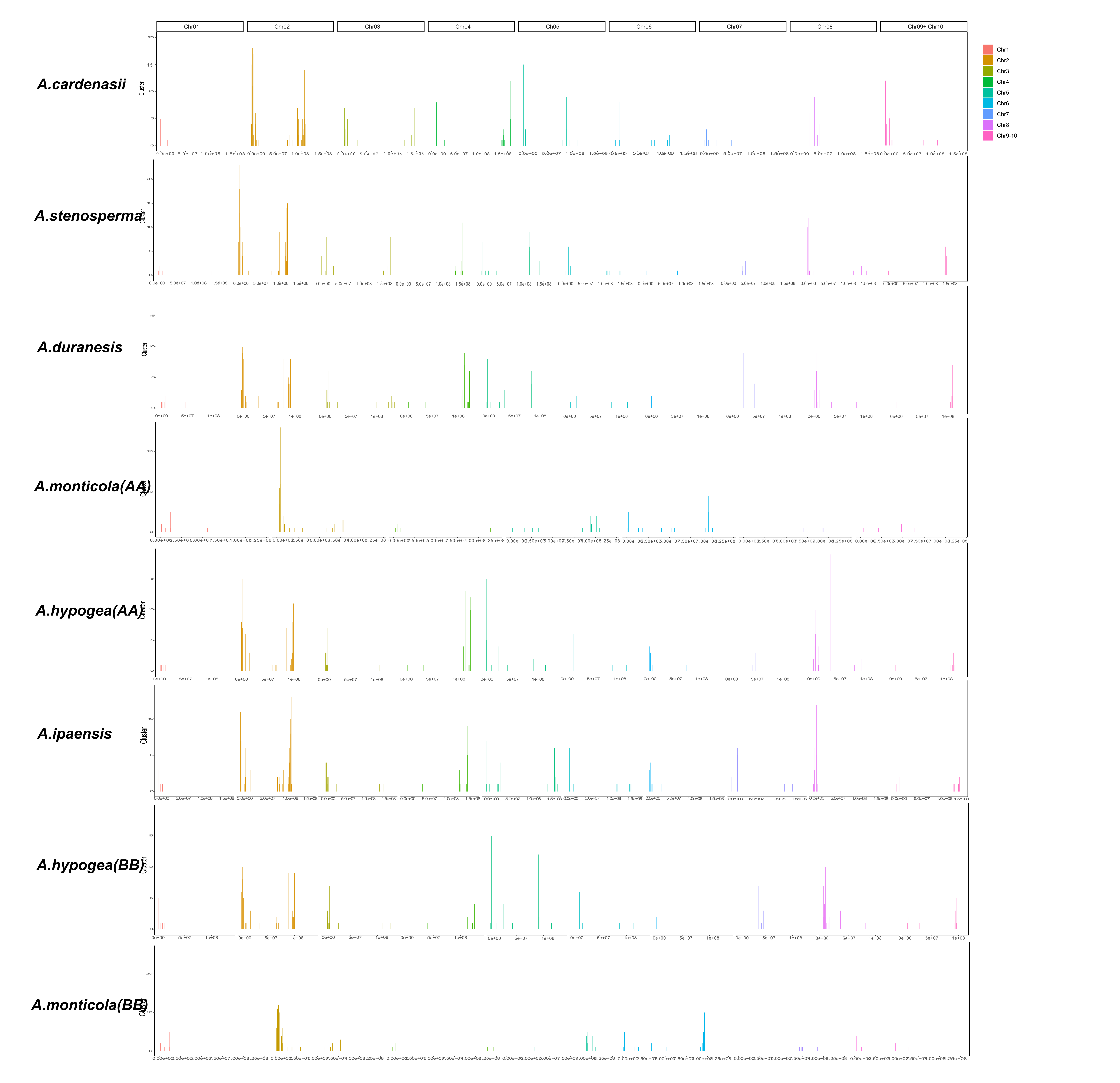

Supplement: Supplementary file 11 — Supplementary Information 11. [file 41598_2023_36302_MOESM11_ESM.png]
